# Deafblindness in French Canadians from Quebec: a predominant founder mutation in the *USH1C *gene provides the first genetic link with the Acadian population

**DOI:** 10.1186/gb-2007-8-4-r47

**Published:** 2007-04-03

**Authors:** Inga Ebermann, Irma Lopez, Maria Bitner-Glindzicz, Carolyn Brown, Robert Karel Koenekoop, Hanno Jörn Bolz

**Affiliations:** 1 Institute of Human Genetics, University Hospital of Cologne, Kerpener Str., 50931 Cologne, Germany; 2 McGill Ocular Genetics Laboratory, Montreal Children's Hospital Research Institute, Tupper, Montreal, PQ, Canada, H3H 1P3; 3 Unit of Clinical and Molecular Genetics, Institute of Child Health, University College London, Guilford St, London WC1N 1EH, UK

## Abstract

**Background:**

Usher syndrome type 1 (USH1) is the leading cause of deafblindness. In most populations, many private mutations are distributed across the five known USH1 genes. We investigated patients from the French Canadian population of Quebec (approximately 6 million people) that descends from about 8,500 French settlers who colonized the St Lawrence River valley between 1608 and 1759. We hypothesized that founder mutations in USH1 genes exist in this population.

**Results:**

We have genetically characterized 15 patients from different regions of Quebec who were clinically diagnosed as USH1. Of these cases, 60% carried mutations of the *USH1C *gene, a genetic subtype that is rare outside the Acadian population. We have discovered a founder effect of the c.216G>A mutation, which has previously been designated the 'Acadian allele' because it accounts for virtually all Acadian USH1 cases. It represents 40% of disease alleles in Quebec, and a carrier of c.216G>A was identified in the general population. Mutations in other genes, except *CDH23*, are very rare.

**Conclusion:**

Based on our findings, approximately 0.5% of congenitally deaf children in Quebec are at risk of developing retinal degeneration due to homozygosity for c.216G>A. Although the Acadians and French Canadians from Quebec are descended from French ancestors, they have always been considered genetically distinct. The genetic conditions common in Quebec are generally not found in Acadians, or they are due to different mutations. Our results, however, show that carriers of the c.216G>A allele haplotype belonged to the early founders of both the Acadian and the Quebec population.

## Abstract 2

Genetic characterisation of 15 French Canadian patients from different regions of the province of Quebec who were clinically diagnosed as USH1 reveals that carriers of the c.216G>A-allele haplotype belong to the early founders of both the Acadian and the Quebec population.

## Background

Usher syndrome (USH) is an autosomal recessive condition characterized by sensorineural hearing loss, variable vestibular dysfunction, and visual impairment due to retinitis pigmentosa. It is the leading cause of deafblindness, with a general prevalence of 2 to 6.2 in 100,000 [1,2]. Three clinical subtypes are distinguished, with type 1 (USH1; MIM 276900) representing the most severe subtype with profound congenital deafness, vestibular dysfunction, and prepubertal onset of retinitis pigmentosa. To date, five USH1 genes have been identified [3-10]. In a recent study on USH1 patients from the US and the UK, 39% of patients had mutations in myosin-7A (*MYO7A/USH1B*) or cadherin-23 (*CDH23/USH1D*), 11% had mutations in protocadherin-15 (*PCDH15/USH1F*), 7% had mutations in *SANS *(*USH1G*), and 7% had mutations in *USH1C *(non-Acadians) [11]. These proportions are in line with most investigations of other populations where *MYO7A *is the most commonly mutated gene in USH1. However, in the Ashkenazi Jewish population and the Acadian population of the Southern United States, founder effects for *USH1F *and for *USH1C*, respectively, lead to locally high incidences of these genetic subtypes [12,13].

The current French Canadian population of Quebec of approximately 6 million people descends from about 8,500 French settlers who colonized the St Lawrence River valley between 1608 and 1759. The 2,600 settlers who arrived before 1680 contributed about two-thirds of the current gene pool [14]. We hypothesized that one or more founder mutations in USH1 genes may exist in this population. In order to investigate this, we have evaluated 15 USH1 patients (from 15 separate families) from different parts of Quebec for mutations in all known USH1 genes. Several founder mutations were identified, including an *USH1C *mutation that has previously been described almost exclusively in Acadians, where it is responsible for virtually all USH1 cases. In our patients from Quebec, this mutation accounts for 40% of disease alleles, a finding that will have a major impact on diagnostic and clinical management of deaf children in Quebec. Although Acadians and Quebecers are both French Canadian, Acadia was founded four years prior to Quebec and in a geographically separate area now corresponding to New Brunswick and Nova Scotia. Acadians to a great extent came from different parts of France than do Quebecois. (the meaning of the sentence has been altered by editing; what we want to say is that Acadians came from other regions of France than Quebec people ("Quebecois" is just an alternative expression for "Quebecers") Consequently, the two populations are considered genetically distinct and do not share the same propensity for genetic disorders. However, our data for the first time provide evidence for a genetic link between the population of Quebec and the Acadians, a link that has previously been regarded as unlikely.

## Results

### *USH1C*: novel mutations and a wide-spread founder mutation

**Figure 1:**
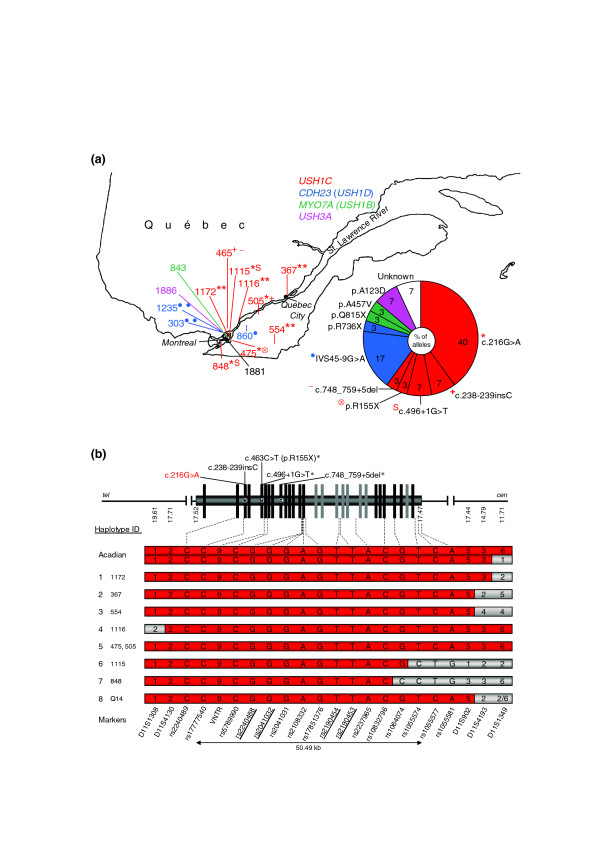
USH1 gene mutations in Quebec and haplotype analysis of the *USH1C *founder mutation c.216G>A. **(a) **Distribution and proportion of USH1 gene mutations in Quebec. Numbers designate patients, colors indicate different USH1 genes. Superscript symbols indicate different mutations that are listed beside the diagram. Note that c.216G>A is present all along the St Lawrence river, suggesting that it was present from the beginning of colonization by French settlers. In sum, *USH1C *mutations account for 60% of USH1 cases investigated in this study. In the case of patient 1881, no mutation could be identified in any of the known USH1 genes. See Additional data file 4 for the precise origin of each patient. **(b) **Genomic structure of the *USH1C *gene and haplotype bearing the c.216G>A mutation in different patients. Constitutive exons are given in black, alternatively spliced exons in grey. Mutations identified in this study are given above. Asterisks indicate novel mutations. Designations of intragenic SNPs and polymorphic microsatellite repeat markers are given below (SNPs in bold are referred to in Figure 2). The corresponding UCSC map positions are indicated below the scheme of the *USH1C *gene. 'VNTR' designates the 45 bp variable number of tandem repeat polymorphism in intron 5, which is in complete linkage disequilibrium with the c.216G>A mutation. Presence of the 9VNTR(t,t) allele is indicated by '9'. Alleles of microsatellite markers are represented by numbers indicating different repeat lengths. Slashes indicate that marker alleles could not be assigned definitively to a haplotype. For biallelic SNPs, the respective nucleotide is given (according to the genomic *USH1C *sequence in 5'-3' orientation). Haplotype IDs and respective patients are given in the left column. Haplotypes associated with c.216G>A are in red. For patients who are compound heterozygous for c.216G>A and another mutation, only the c.216G>A-associated haplotype is shown. Recombination events are indicated by grey background. Acadian: for comparison of the c.216G>A-associated haplotype ('Acadian allele') with haplotypes in our sample, we have genotyped the family of a previously described patient with homozygosity for c.216G>A (see Additional data file 1a). As there is a recombination event in this patient for marker D11S1349, both alleles are shown. 1-4: haplotypes from patients 1172, 367, 554, and 1116 who are homozygous for c.216G>A. In the case of a recombination event, only the corresponding allele is shown. 5-7: c.216G>A-associated haplotypes from compound heterozygous patients. 8: c.216G>A-associated haplotype from healthy carrier (Q14).

Mutation screening in exons 1, 2, 3, 5 and 6 of the *USH1C *gene revealed the previously reported exon 3 mutation c.216G>A in a homozygous state in four patients (one of the two corresponding families is shown in Additional data file 1b). Another four patients with the c.216G>A mutation were found to be compound heterozygotes, carrying different *USH1C *mutations on the second allele: c.238-239insC in exon 3 (see Additional data file 1c), and two novel mutations, c.463C>T in exon 5 (see Additional data file 1d), and c.496+1G>T in intron 5 (two patients; see Additional data file 1e). One patient was compound heterozygous for c.ins238-239insC and a novel deletion in exon 9, c.748_759+5del (see Additional data file 1f). All novel *USH1C *mutations are predicted to be truncating: c.463C>T creates an in-frame stop codon (p.R155X), whereas both c.496+1G>T and c.748_759+5del probably lead to aberrant splicing. In c.496+1G>T, the transversion affects the invariant consensus sequence of the exon 5 donor splice site. A G-to-A transition at the same position has been reported previously in an USH1 patient [15]. In c.748_759+5del, the twelve last nucleotides of exon 9 and five intronic nucleotides, including the donor splice site, are deleted. In sum, *USH1C *mutations account for 60% of disease alleles in our French Canadian USH1 patient cohort, with c.216G>A alone accounting for 40% (Figure 1a).

The silent mutation c.216G>A has previously been shown to result in aberrant splicing of the *USH1C *gene [16]. It has been described as a founder mutation restricted to the Acadian population ('Acadian allele'), where it accounts for virtually all USH1 cases [5,8,17] and is in complete linkage disequilibrium with the 9VNTR(t,t) allele of a 45 base-pair (bp) variable number of tandem repeat (VNTR) polymorphism in intron 5 of the *USH1C *gene [17].

In our cohort, the c.216G>A mutation accounts for 40% of disease alleles. While all other mutations identified in our study were absent in 100 French Canadian control samples from Quebec, c.216G>A was present in a heterozygous state in one out of 227 healthy control individuals, suggesting a carrier rate of about 0.44% in the Quebec population. In order to elucidate the c.216G>A haplotype, we analyzed genotypes of 16 intragenic single nucleotide polymorphisms (SNPs), the intron 5 VNTR, and locus-specific microsatellite markers of the *USH1C *locus. The results were consistent with a common ancestral c.216G>A-associated haplotype in our eight patients from Quebec, a previously described Acadian *USH1C *patient [5] and the heterozygous healthy carrier from the Quebec population (Figure 1b). Moreover, we found evidence for historical meiotic recombinations of both intragenic SNPs and a closely flanking microsatellite marker (haplotypes 1 to 4 and 6 to 8, respectively).

The insertion c.238-239insC has previously been found in several USH1 cases from Europe, Asia, and North America [11,15,18]. Despite the overall low prevalence of *USH1C *in most populations, c.238-239insC has been found in 14% and 12.5% of USH1 patients in the UK and Germany, respectively, which may be due to founder effects [15,18]. Haplotype analysis in our patients carrying c.238-239insC was in line with haplotype data available from European patients with that mutation (see Additional data file 2).

**Figure 2:**
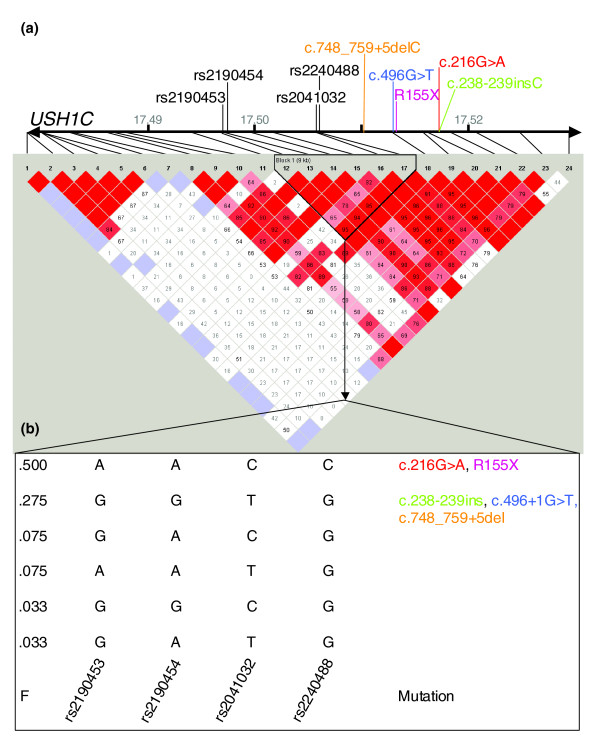
Haplotype structure of the *USH1C *gene locus in Europeans. **(a) **Structure of the *USH1C *gene with relative positions of mutations identified in this study and SNPs typed by the HapMap project [30]. Rate (D') of linkage disequilibrium (LD; visualized by haploview program) is represented by different colors (highest rate of LD in dark red and lower LD in light red/white). The marked block defines potential haplotypes between selected SNPs. Note that the orientation of the graphic is opposite to Figure 1b and that SNP alleles are given in reverse complement. **(b) **Potential haplotypes in the European population (CEU) for four selected SNPs that have been genotyped in our study. F: putative frequency of these haplotypes in CEU as determined in the HapMap project. The 'Acadian allele', c.216G>A, and p.R155X are both located on the most prevalent haplotype predicted to account for 50% of haplotypes. c.238-239insC, c.496+1G>T and c.748_759+5del are located on the second most common haplotype.

Using data from the HapMap project, the *USH1C *mutations identified in our study could all be assigned to *USH1C *haplotypes predicted to be present in the Central European population (CEU), supporting the results of our analyses (Figure 2). Both c.216G>A and the novel p.R155X mutation originated on the most common *USH1C *CEU haplotype. The mutations c.238-239insC, c.496+1G>T and c.748_759+5del probably occurred independently on the background of the second most common CEU haplotype. While the haplotype associated with the insertion in our patients is centromerically restricted because of different alleles for the marker D11S1349, probably indicating an older mutation, the haplotype bearing the c.496+1G>T mutation is identical over the whole range of analyzed markers (7.9 Mb) in both patients with this mutation.

### *CDH23 *mutations

Two patients were homozygous for the *CDH23 *mutation IVS45-9G>A and showed the same haplotype as previously reported for German patients with this mutation [19], suggesting a common origin of this mutation (see Additional data file 3). Another patient was compound heterozygous for IVS45-9G>A and a novel nonsense mutation, c.2206C>T (p.R736X). Thus, *CDH23 *mutations are responsible for 20% of USH1 cases in our study (Figure 1a).

### *MYO7A *and *USH3A *mutations

Only one patient in our study had mutations in *MYO7A*, the most common USH1 gene in most other populations; this patient was compound heterozygous for the previously reported missense mutation c.1370C>T (p.A457V) [20], and a novel nonsense mutation, c.2443C>T (p.Q815X). One of the USH1 patients investigated here was homozygous for a novel missense mutation in the *USH3A *gene, p.A123D (c.368C>A), which is likely to be pathogenic as p.A123 is evolutionarily conserved (data not shown) and the change was not present in controls (100 French Canadians and 93 from mixed ethnic groups). One patient did not show any pathogenic change in any USH1 gene.

## Discussion

French settlement in North America started in 1608 and occurred in mainly two regions: along the St Lawrence River (the later Quebec) and in Acadia (today corresponding to New Brunswick and Nova Scotia). The Acadians, many of whom were deported to the United States in 1755, gave rise to the later populations of Maritime Canada and to the Cajuns of the southern US. Some Acadians escaped deportation and moved to what is now Quebec. By the English conquest in 1759, French immigration stopped. Linguistic and religious barriers discouraged admixture with the mostly Protestant English-speaking new settlers. Highest birth rates occurred at pioneer fronts, that is, rural regions opened to colonization, which existed until the early 20th century. Quebec has been considered a mosaic of layered founder effects, resulting from the distinct settlers' gene pools of the respective pioneer fronts. Despite modern mobility, secularization, urbanization and immigration in the second half of the 20th century, historical founder effects still have a strong impact on medical genetics and public health in Quebec. As a result, some diseases are found more frequently or exclusively in French Canadians from Quebec than in other populations, or they have special clinical or genetic features (for example, agenesis of corpus callosum and peripheral neuropathy). Frequently, a few founder mutations account for the vast number of cases of a given phenotype [14].

The genetic conditions found in Quebec are generally not found in Acadians, or they are due to different mutations (for example, oculopharyngeal muscular dystrophy) [14]. In the case of USH1, our results challenge this point of view: we have shown that the *USH1C *mutation c.216G>A, the 'Acadian allele', is the main cause of USH1 in Quebec, accounting for 40% of USH1 disease alleles and being found all along the St Lawrence River (Figure 1a). In Acadians, c.216G>A is responsible for virtually all USH1 cases (only one out of 44 Acadian USH1 cases has been shown not to be homozygous, but compound heterozygous for c.216G>A and c.238-239insC [17]). The 9VNTR(t,t) in intron 5 and the c.216G>A mutation are in complete linkage disequilibrium and are almost exclusively found in Acadians, raising the possibility of a recent origin of c.216G>A in this population [17]. Our data now strongly suggest that carriers of c.216G>A belong to the early founders of both the Acadian and the Quebec population (Figure 1b). This would explain its wide distribution all along the St Lawrence River valley, following the direction of the historical colonization movement (Figure 1a).

*USH1C *has so far been considered rare outside Acadia and peoples descended from that region, accounting for 7% of USH1 cases in a recent study on US and UK patients [11]. Roux *et al*. [21] have performed extensive mutation screening in USH1 patients from France and found *USH1C *mutations in only 6% of cases. Of note, c.216G>A was not detected; this resembles the rhodopsin p.P23H mutation, which has been found in 12% of Irish-American families with autosomal dominant RP, but not in Europe [22]. Strikingly, *USH1C *mutations account for 60% of USH1 in the patients investigated here, followed by *CDH23*/*USH1D *(20%). *MYO7A *mutations (*USH1B*), which are responsible for the main proportion of USH1 cases in other populations, only play a minor role (one patient). To date, only six USH1-causing mutations have been identified in the *USH1C *gene [5,8,15]. In our collective derived from a founder population, however, we met unexpected allelic heterogeneity: five different *USH1C *mutations, including three novel (p.R155X, c.496+1G>T, and c.748_759+5del), were found. These novel changes, although probably rare in most cases and likely to be of recent origin at a given pioneer front in some, could clinically manifest because of the high prevalence of c.216G>A. Although digenic inheritance of type 1 has been described in USH1 [23], we did not find this pattern of inheritance in our patients. Thus, there is no indication that *USH1C *is involved in digenic Usher syndrome, at least probably not in combination with the other USH genes found to be mutated here.

We show that the Quebec population is the second population in the world in which *USH1C *is the major genetic USH1 subtype, which is rare in all other populations studied to date. USH1 is a severely disabling disorder, causing major communication handicap due to congenital deafness and progressive retinal degeneration resulting in legal blindness in most cases. There are no official numbers describing the incidence of Usher syndrome in Quebec. However, the carrier rate of approximately 0.44% solely for the *USH1C *mutation c.216G>A in the Quebec population suggests an incidence of Usher syndrome type 1C of 0.5 per 100,000 (assuming random mating and complete penetrance), based on c.216G>A alone. Assuming a minimum incidence of 1 per 1,000 for children with congenital profound hearing impairment [24], 0.5% of these children may develop additional retinitis pigmentosa due to homozygosity for the *USH1C *mutation c.216G>A. Since 60% of USH1 cases in our study are due to other mutations (20% of which also affect the *USH1C *gene), and because of local founder effects (also for other USH1 subtypes such as USH1D), the incidence for USH1 may (regionally) be even higher.

While routine testing for USH1 gene mutations is hampered by the number and size of the genes involved in most populations, our data should facilitate molecular diagnosis of deafness and Usher syndrome in Quebec (>60% of cases have a mutation in *USH1C *and >90% of cases can be explained by ten mutations). Knowledge that a mutation in a profoundly deaf child will result in severe visual impairment in later life is important for rehabilitative strategies: parents may be more likely to choose cochlear implantation for their child rather than visual modes of communication such as sign language.

Our findings suggest that the *USH1C *mutation c.216G>A is one of only a handful of common single USH1 mutations, along with founder mutations in *USH3A *in the Finnish and the Ashkenazi Jewish population and a *PCDH15*/*USH1F *founder allele in Ashkenazi Jews [12,25,26]; moreover, it is predominant in two populations. The recent development of a mouse model carrying the c.216G>A mutation in *USH1C *is an important step in the development of a specific therapeutic approach for the treatment or amelioration of this devastating condition in affected individuals with the c.216G>A mutation [27].

## Conclusion

Our study sheds new light on the colonization history of two North American regions and their populations, French Canadians from Quebec and Acadians. Moreover, the finding of a wide-spread founder mutation for an otherwise rare genetic subtype of deafblindness is of great importance to the medical community as this knowledge should strongly influence diagnostic and therapeutic management of congenitally deaf children in Quebec.

## Materials and methods

### Patients

French Canadian subjects from Quebec with USH1 were identified through the McGill Ocular Genetics Laboratory, Montreal Children's Hospital Research Institute, McGill University Health Center, Montreal, Quebec, Canada (see Additional data file 4 for precise origin of each patient). The study was performed according to the Declaration of Helsinki and approved by the institutional review boards of both institutions involved (ethics committees of McGill University and the University Hospital of Cologne). Written informed consent was obtained from all participants. All patients met the diagnostic criteria for USH1.

The healthy control individuals (all had negative family history for Usher syndrome) came from all regions of Quebec.

### Detection of mutations and haplotype analyses

Genomic DNA was extracted from venous EDTA blood samples. To identify founder mutations in USH1 patients from Quebec, we followed a combination of different strategies. We performed genotyping of polymorphic microsatellite markers closely flanking the seven USH1 loci in parallel with a mutation screening strategy. In the case of homozygosity for marker alleles of a specific USH1 locus, the coding region of the corresponding gene was sequenced. Where these approaches did not lead to the identification of the genetic subtype, we sequenced the entire coding regions of all USH1 genes, in the order of mutation prevalences in other populations: *MYO7A *(*USH1B*), *CDH23 *(*USH1D*), *USH1C*, and *PCDH15 *(*USH1F*). Once a mutation was identified, all other patients were screened for this change. As *USH1C *mutations have so far only been reported in exons 1, 2, 3, 5 and 6, these exons were sequenced first. The small genes *SANS *(*USH1G*) and *USH3A *(which was screened because it has previously been reported to be causative in some USH1 patients) were also analyzed by direct sequencing in parallel with microsatellites.

PCR was carried out following standard protocols and using gene-specific primers that amplify the coding exons and adjacent intronic sequences. For amplification of locus-specific polymorphic microsatellite repeat markers, we used fluorescent dye-labeled primers. For the amplification of several markers, we applied the tailed primer method as described previously [28] (primers and protocols available on request). Purified PCR fragments were sequenced using Big Dye Terminator Cycle sequencing (Applied Biosystems, Foster City, CA, USA) and analyzed on an ABI-377 DNA sequencer by capillary electrophoresis. Microsatellite markers were also analyzed on an ABI-377 DNA sequencer and genotypes were determined by GeneScan software (Applied Biosystems). Data provided by the Genome Database [29] were used as references for allele sizes. SNPs were genotyped by either restriction enzyme digest of corresponding PCR fragments or by direct sequencing. Samples were genotyped for the presence of the 9VNTR(t,t) allele of the VNTR polymorphism in intron 5 of the *USH1C *gene as described previously [17]. Screening for the mutations detected in this study in other patients and in normal controls was, in part, possible by restriction enzyme digest (*Dra*III for c.216G>A (*USH1C*), *Bsp119*I for p.R155X (*USH1C*), *Esp3*I for c.496+1G>T (*USH1C*), and *Nhe*I for p.Q815X (*MYO7A*)). Screening for the *USH1C *insertion c.238-239insC in exon 3 was performed by using fluorescent dye-labeled primers for amplification of exon 3 and subsequent analysis of fragment length on the ABI-377 DNA sequencer. DNA mutation numbering of identified mutations was given based on cDNA sequences of GenBank entries given below, with +1 corresponding to the A of the ATG translation initiation codon (codon 1) in the respective reference sequence.

### Accession numbers

*USH1C *mRNA, isoform b3 (GenBank:NM_153676.2); *CDH23 *mRNA (GenBank:AF312024.1); *MYO7A *mRNA (GenBank:NM_000260.2); *USH3A *mRNA (GenBank:AF482697.1).

## Additional data files

The following additional data are available with the online version of this paper. Additional data file 1 is a figure showing the *USH1C *genotypes identified in this study. **(a) **The family of an Acadian USH1 patient that has previously been shown to be homozygous for the Acadian founder mutation, c.216G>A [5], was available for haplotype analysis. *USH1C *haplotypes are represented by vertical colored bars (c.216G>A-associated haplotype in red). See Figure 1b and Figure 2 for detailed haplotypes. **(b) **Two brothers with homozygosity for c.216G>A, which was also found in patients 367, 1116, and 1172. **(c) **Compound heterozygosity for c.216G>A and c.238-239insC in two brothers. **(d) **Compound heterozygosity for c.216G>A and the novel nonsense mutation p.R155X (patient 475). **(e) **Compound heterozygosity for c.216G>A and a novel splice site mutation, c.496+1G>T, which affects the invariant donor splice site of exon 5 (patients 848 and 1115). **(f) **Compound heterozygosity for c.238-239insC and the novel 17 bp deletion 748_759+5del, which removes 12 exonic and five intronic base-pairs, including the donor splice site of exon 9 (patient 465). Additional data file 2 is a figure that displays the haplotypes associated with *USH1C *mutations c.238-239insC, p.R155X, c.748_759+5del, and c.496+1G>T. SNPs in bold are referred to in Figure 2. '∅' indicates absence of the 9VNTR(t,t) allele. European: haplotype associated with c.238-239insC in European patients as published by Zwaenepoel *et a*l. [15]. 1-2: haplotype associated with c.238-239insC in our patients (compound heterozygosity for c.748_759+5del and c.216G>A, respectively). Common haplotypes in Quebec and European USH1 patients carrying the c.238-239insC mutation suggest that the mutation probably has recently been locally 'imported' by other ethnic communities after completion of settlement. Note different alleles for D11S1349 on the chromosome carrying c.238-239insC in patients 465 and 505, respectively. 3: haplotype associated with c.496+1G>T. 4 and 5: Haplotypes associated with novel mutations c.748_759+5del and p.R155X, respectively. Additional data file 3 is a figure that displays the haplotypes associated with *CDH23 *mutation IVS45-9G>A. Homozygosity for the *CDH23 *mutation IVS45-9G>A was found in patients 303 and 1235, while patient 860 was compound heterozygous for IVS45-9G>A and the novel nonsense mutation p.R736X. SNP alleles are given according to the genomic *CDH23 *sequence in 5'-3' orientation. The haplotype associated with IVS45-9G>A in Quebec patients matches with the *CDH23 *haplotype of two German families that we have investigated previously [19]. As in the case of c.238-239insC, this could be due to settlement of ethnic groups other than French Canadians. Note recombination event for marker D10S1759 in patient 1235. N.d. = not determined. Additional data file 4 consists of a table and a map figure illustrating the precise origins of patients investigated in this study. The map illustrates the location of the places given in the table (cities associated with patients carrying c.216G>A in red). See also Figure 1a. Additional data file 5 consists of figures illustrating the haplotypes of patients with *USH1C *and *CDH23*/*USH1D *mutations. Fragment length analysis is shown for microsatellite markers (GeneScan, Applied Biosystems), electropherograms for SNPs, and agarose gel electrophoresis for the VNTR in intron 5 of the *USH1C *gene. Additional data file 6 comprises figures that show all mutations that have been identified in USH1 patients that have been investigated in this study (electropherograms), and figures for genotyping of healthy French Canadian control individuals for these mutations (by direct sequencing, restriction enzyme digest, and fragment length analysis). Additional data file 7 contains figures that illustrate results of mutation screening in patient 1881 in the following genes: *MYO7A *(*USH1B*) and *USH1C *(no mutations found). Additional data file 8 contains figures that illustrate results of mutation screening in patient 1881 in the *CDH23 *gene (*USH1D*) (no mutations found). Additional data file 9 contains figures that illustrate results of mutation screening in patient 1881 in the following genes: *PCDH15 *(*USH1F*), SANS (*USH1G*) and *USH3A *(no mutations found).

## Supplementary Material

Additional data file 1 *USH1C *genotypes identified in this study. **(a) **The family of an Acadian USH1 patient that has previously been shown to be homozygous for the Acadian founder mutation, c.216G>A [5], was available for haplotype analysis. *USH1C *haplotypes are represented by vertical colored bars (c.216G>A-associated haplotype in red). See Figure 1b and Figure 2 for detailed haplotypes. **(b) **Two brothers with homozygosity for c.216G>A, which was also found in patients 367, 1116, and 1172. **(c) **Compound heterozygosity for c.216G>A and c.238-239insC in two brothers. **(d) **Compound heterozygosity for c.216G>A and the novel nonsense mutation p.R155X (patient 475). **(e) **Compound heterozygosity for c.216G>A and a novel splice site mutation, c.496+1G>T, which affects the invariant donor splice site of exon 5 (patients 848 and 1115). **(f) **Compound heterozygosity for c.238-239insC and the novel 17 bp deletion 748_759+5del, which removes 12 exonic and five intronic base-pairs, including the donor splice site of exon 9 (patient 465). Click here for file

Additional data file 2 SNPs in bold are referred to in Figure 2. '∅' indicates absence of the 9VNTR(t,t) allele. European: haplotype associated with c.238-239insC in European patients as published by Zwaenepoel *et a*l. [15]. 1-2: haplotype associated with c.238-239insC in our patients (compound heterozygosity for c.748_759+5del and c.216G>A, respectively). Common haplotypes in Quebec and European USH1 patients carrying the c.238-239insC mutation suggest that the mutation probably has recently been locally 'imported' by other ethnic communities after completion of settlement. Note different alleles for D11S1349 on the chromosome carrying c.238-239insC in patients 465 and 505, respectively. 3: haplotype associated with c.496+1G>T. 4 and 5: Haplotypes associated with novel mutations c.748_759+5del and p.R155X, respectively. Click here for file

Additional data file 3 Homozygosity for the *CDH23 *mutation IVS45-9G>A was found in patients 303 and 1235, while patient 860 was compound heterozygous for IVS45-9G>A and the novel nonsense mutation p.R736X. SNP alleles are given according to the genomic *CDH23 *sequence in 5'-3' orientation. The haplotype associated with IVS45-9G>A in Quebec patients matches with the *CDH23 *haplotype of two German families that we have investigated previously [19]. As in the case of c.238-239insC, this could be due to settlement of ethnic groups other than French Canadians. Note recombination event for marker D10S1759 in patient 1235. N.d. = not determined. Click here for file

Additional data file 4 The map illustrates the location of the places given in the table (cities associated with patients carrying c.216G>A in red). See also Figure 1a. Click here for file

Additional data file 5 Fragment length analysis is shown for microsatellite markers (GeneScan, Applied Biosystems), electropherograms for SNPs, and agarose gel electrophoresis for the VNTR in intron 5 of the *USH1C *gene. Click here for file

Additional data file 6 All mutations that have been identified in USH1 patients that have been investigated in this study (electropherograms), and figures for genotyping of healthy French Canadian control individuals for these mutations (by direct sequencing, restriction enzyme digest, and fragment length analysis). Click here for file

Additional data file 7 Results of mutation screening in patient 1881 in the genes *MYO7A *(*USH1B*) and *USH1C *(no mutations found). Click here for file

Additional data file 8 Results of mutation screening in patient 1881 in the *CDH23 *gene (*USH1D*) (no mutations found). Click here for file

Additional data file 9 Results of mutation screening in patient 1881 in the genes *PCDH15 *(*USH1F*), SANS (*USH1G*) and *USH3A *(no mutations found). Click here for file
