# Supplementary figures and images for: Deafblindness in French Canadians from Quebec: a predominant founder mutation in the USH1C gene provides the first genetic link with the Acadian population
Source: Genome Biol. 2007 Apr 3;8(4):R47. doi: 10.1186/gb-2007-8-4-r47 (PMC1895989; doi:10.1186/gb-2007-8-4-r47)

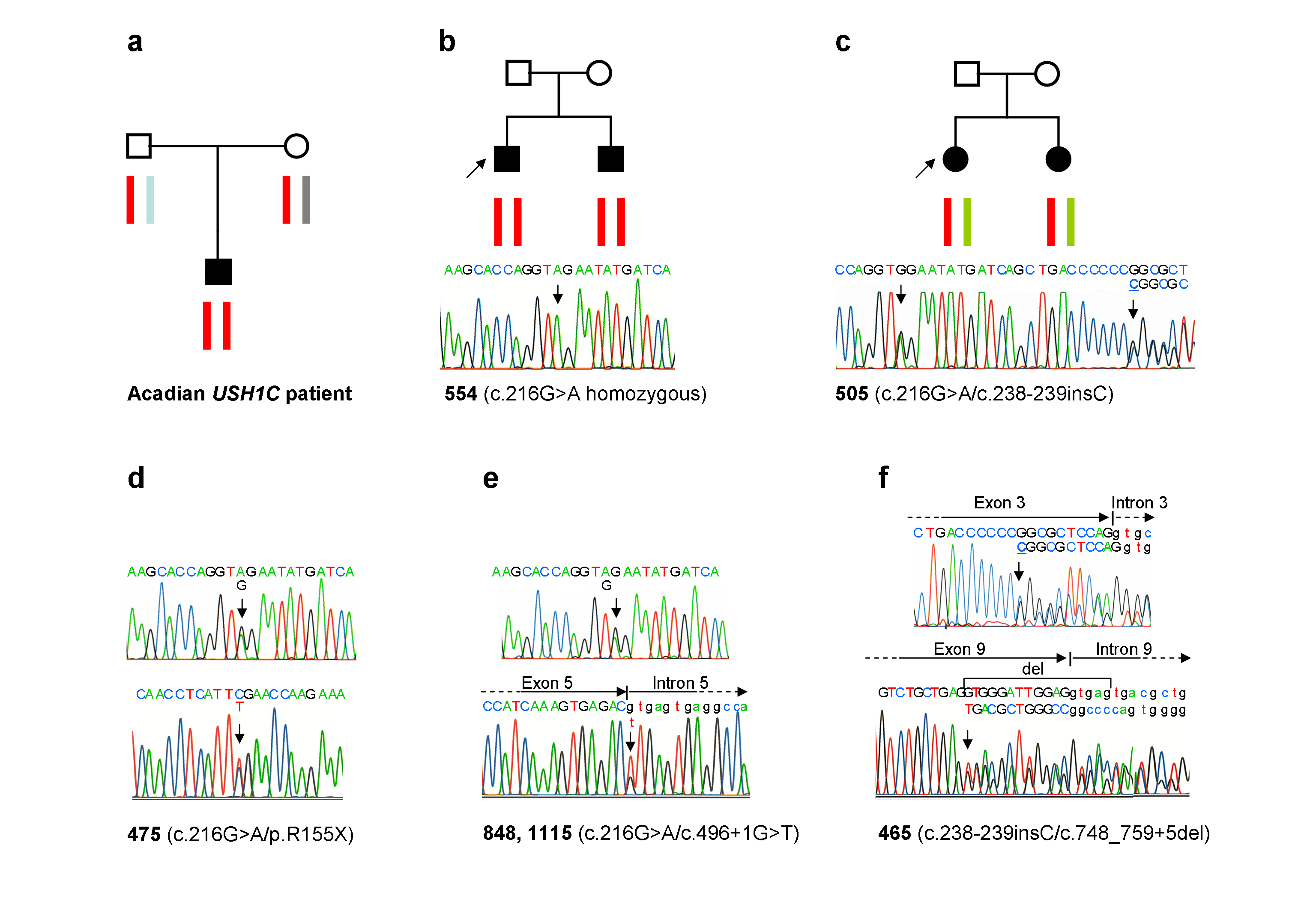

Supplement: Additional data file 1 — USH1C genotypes identified in this study. (a) The family of an Acadian USH1 patient that has previously been shown to be homozygous for the Acadian founder mutation, c.216G>A [5], was available for haplotype analysis. USH1C haplotypes are represented by vertical colored bars (c.216G>A-associated haplotype in red). See Figure 1b and Figure 2 for detailed haplotypes. (b) Two brothers with homozygosity for c.216G>A, which was also found in patients 367, 1116, and 1172. (c) Compound heterozygosity for c.216G>A and c.238-239insC in two brothers. (d) Compound heterozygosity for c.216G>A and the novel nonsense mutation p.R155X (patient 475). (e) Compound heterozygosity for c.216G>A and a novel splice site mutation, c.496+1G>T, which affects the invariant donor splice site of exon 5 (patients 848 and 1115). (f) Compound heterozygosity for c.238-239insC and the novel 17 bp deletion 748_759+5del, which removes 12 exonic and five intronic base-pairs, including the donor splice site of exon 9 (patient 465). [file gb-2007-8-4-r47-S1.tiff]

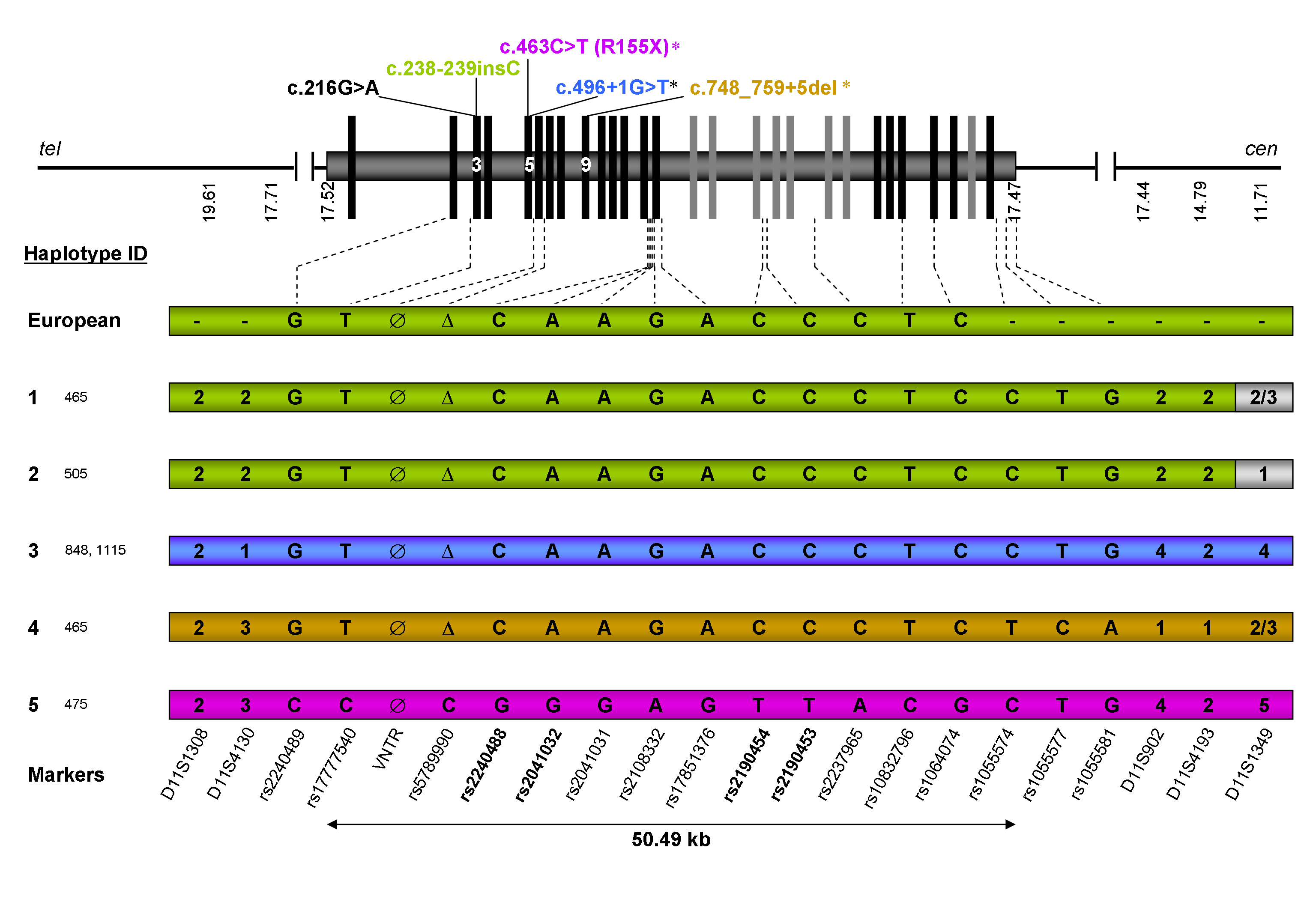

Supplement: Additional data file 2 — SNPs in bold are referred to in Figure 2. '∅' indicates absence of the 9VNTR(t,t) allele. European: haplotype associated with c.238-239insC in European patients as published by Zwaenepoel et al. [15]. 1-2: haplotype associated with c.238-239insC in our patients (compound heterozygosity for c.748_759+5del and c.216G>A, respectively). Common haplotypes in Quebec and European USH1 patients carrying the c.238-239insC mutation suggest that the mutation probably has recently been locally 'imported' by other ethnic communities after completion of settlement. Note different alleles for D11S1349 on the chromosome carrying c.238-239insC in patients 465 and 505, respectively. 3: haplotype associated with c.496+1G>T. 4 and 5: Haplotypes associated with novel mutations c.748_759+5del and p.R155X, respectively. [file gb-2007-8-4-r47-S2.tiff]

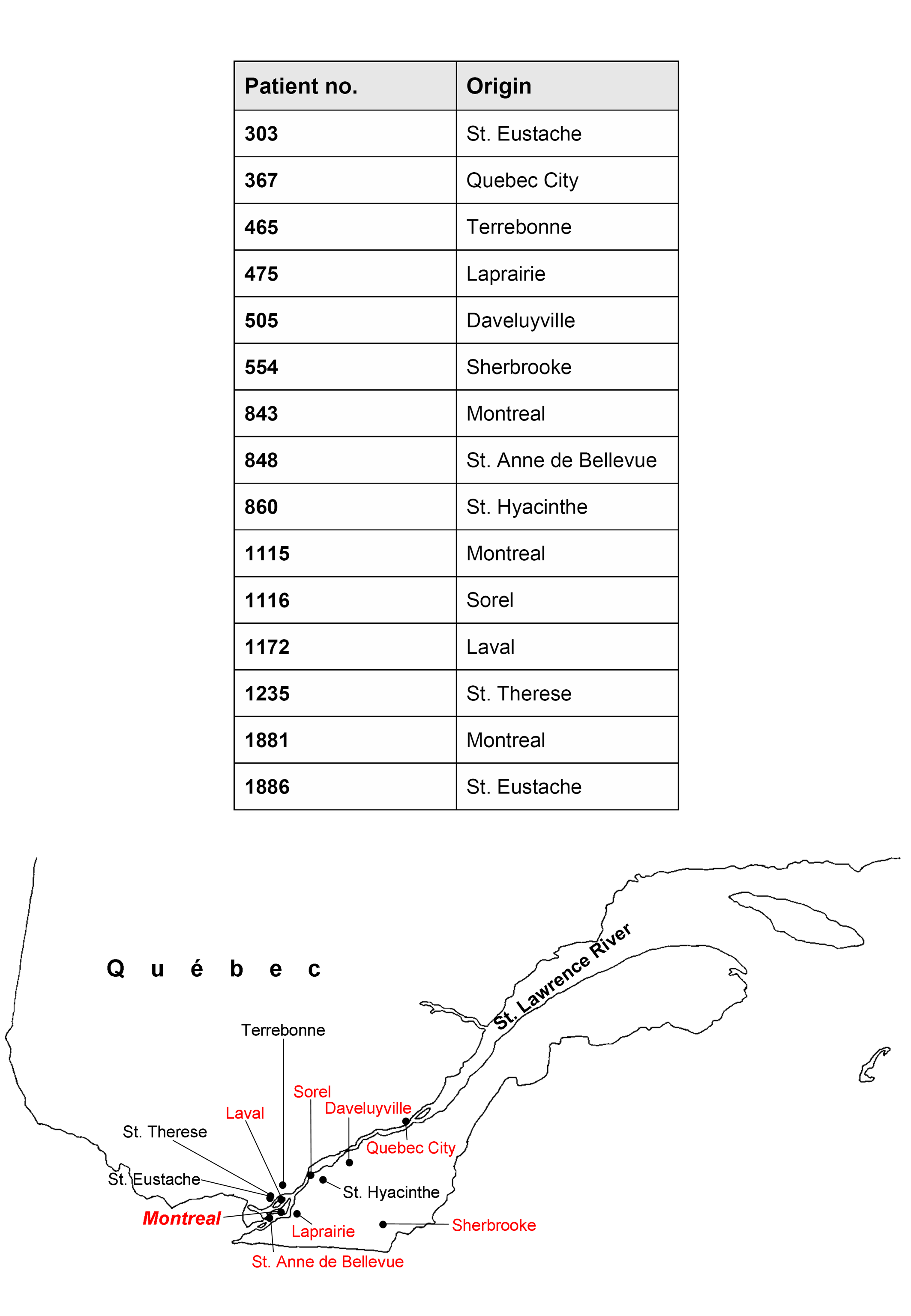

Supplement: Additional data file 4 — The map illustrates the location of the places given in the table (cities associated with patients carrying c.216G>A in red). See also Figure 1a. [file gb-2007-8-4-r47-S4.tiff]
