# Supplementary material for: Deafblindness in French Canadians from Quebec: a predominant founder mutation in the USH1C gene provides the first genetic link with the Acadian population
Source: Genome Biol. 2007 Apr 3;8(4):R47. doi: 10.1186/gb-2007-8-4-r47 (PMC1895989; doi:10.1186/gb-2007-8-4-r47)
Supplement: Additional data file 3 — Homozygosity for the CDH23 mutation IVS45-9G>A was found in patients 303 and 1235, while patient 860 was compound heterozygous for IVS45-9G>A and the novel nonsense mutation p.R736X. SNP alleles are given according to the genomic CDH23 sequence in 5'-3' orientation. The haplotype associated with IVS45-9G>A in Quebec patients matches with the CDH23 haplotype of two German families that we have investigated previously [19]. As in the case of c.238-239insC, this could be due to settlement of ethnic groups other than French Canadians. Note recombination event for marker D10S1759 in patient 1235. N.d. = not determined. [file gb-2007-8-4-r47-S3.doc]

| **Marker/SNP** | **rsSNP** | **German** | | **303** | | **1235** | | **860** | |
| --- | --- | --- | --- | --- | --- | --- | --- | --- | --- |
|  |  | **IVS45-9G>A** | **IVS45-9G>A** | **IVS45-9G>A** | **IVS45-9G>A** | **IVS45-9G>A** | **IVS45-9G>A** | **IVS45-9G>A** | **c.2206C>T**  **(R736X)** |
| D10S529 | - | n.d. | n.d. | **2** | **2** | **2** | 3 | **2** | 1 |
| D10S1688 | - | n.d. | n.d. | **3** | **3** | **3** | 1 | **3** | 2 |
| D10S1759 | - | n.d. | n.d. | **1** | **1** | **1** | 3 | **1** | 2 |
| D10S1694 | - | n.d. | n.d. | **1** | **1** | **1** | **1** | **1** | 1 |
| IVS15-78A>T | rs3802717 | **T** | **T** | **T** | **T** | **T** | **T** | **T** | T |
| c.5712G>A | - | **G** | **G** | **G** | **G** | **G** | **G** | **G** | G |
| c.5996G>C | rs11592462 | **C** | **C** | **C** | **C** | **C** | **C** | **C** | G |
| **IVS45-9G>A** |  | **A** | **A** | **A** | **A** | **A** | **A** | **A** | G |
| IVS48-81G>A | rs7068357 | **A** | **A** | **A** | **A** | **A** | **A** | **A** | G |
| IVS49-16A>G | rs4747193 | **G** | **G** | **G** | **G** | **G** | **G** | **G** | A |
| c.7073G>A | rs4747194 | **A** | **A** | **A** | **A** | **A** | **A** | **A** | G |
| c.7139C>T | rs4747195 | **T** | **T** | **T** | **T** | **T** | **T** | **T** | C |
| c.7572G>A | rs10823849 | **A** | **A** | **A** | **A** | **A** | **A** | **A** | G |
| IVS60+8G>A | - | **A** | **A** | **A** | **A** | **A** | **A** | **A** | A |
| D10S218 | - | n.d. | n.d. | **1** | **1** | **1** | **1** | **1** | 2 |
