# Supplementary material for: Deafblindness in French Canadians from Quebec: a predominant founder mutation in the USH1C gene provides the first genetic link with the Acadian population
Source: Genome Biol. 2007 Apr 3;8(4):R47. doi: 10.1186/gb-2007-8-4-r47 (PMC1895989; doi:10.1186/gb-2007-8-4-r47)

Deafblindness in French Canadians from Quebec: A predominant founder mutation in the *USH1C* gene provides the first genetic link with the Acadian population

Ebermann et al.

RAW DATA

	page
Haplotypes	
USH1C haplotypes	2 – 23
USH1D haplotypes	24 – 29
USH1 mutations	30 – 36
Genotyping of healthy French Canadian Controls	
Controls for c.216G>A (*USH1C*)	37 – 40
Controls for c.238-239insC (*USH1C*)	41 – 58
Controls for c.496+1G>T (*USH1C*)	59 – 60
Controls for p.R155X (*USH1C*)	61 – 62
Controls for c.748-759+5del (*USH1C*)	63 – 64
Controls for IVS45-9G>A (*CDH23*)	65 – 73
Controls for p.R736X (*CDH23*)	74 – 82
Controls for p.A457V (*MYO7A*)	83 – 91
Controls for p.Q815X (*MYO7A*)	92 – 93
Controls for p.A123D (*USH3A*)	94 – 102
Mutation screening in USH1 genes in patient 1881	103 – 204

USH1C haplotypes

Positions of the respective SNPs are indicated by arrows. Genotyping for the presence of the 9VNTR(t,t) allele was carried out as decribed previously [1].

1. Savas S, Frischhertz B, Pelias MZ, Batzer MA, Deininger PL, Keats BB: **The USH1C 216G-->A mutation and the 9-repeat VNTR(t,t) allele are in complete linkage disequilibrium in the Acadian population**. *Hum Genet* 2002, **110**(1):95-97.

USH1C, D11S1308

Acadian

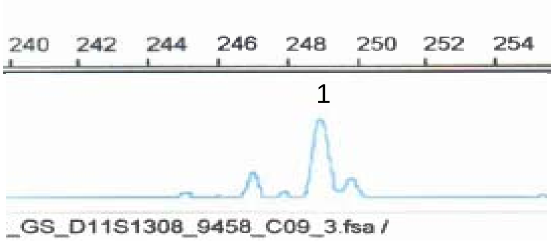

1172

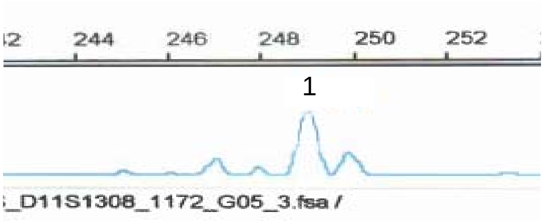

367

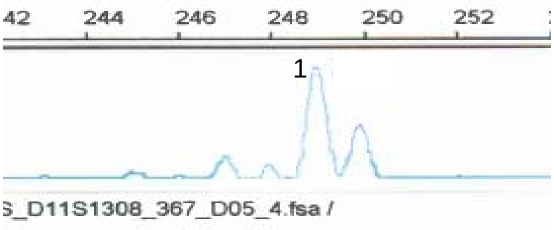

554

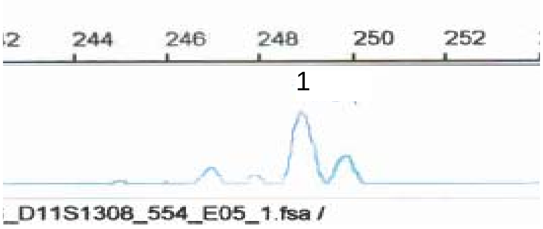

1116

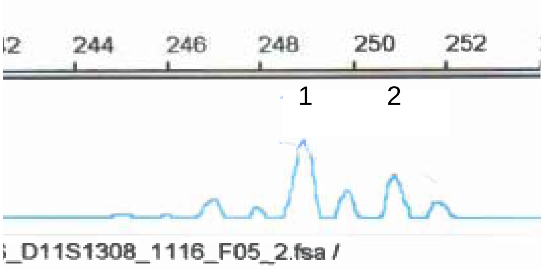

475

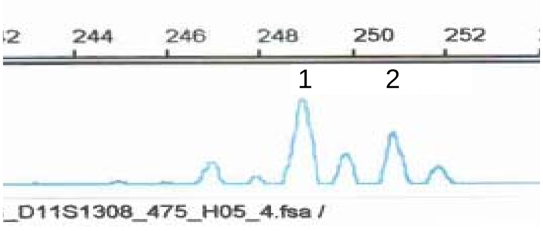

505

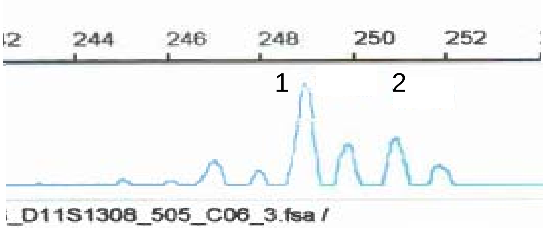

1115

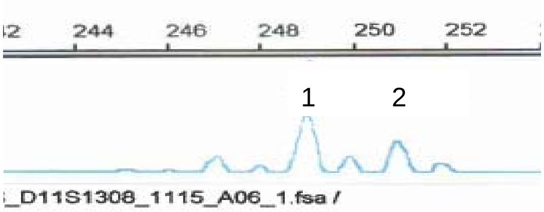

848

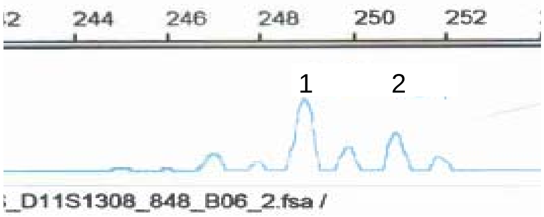

465

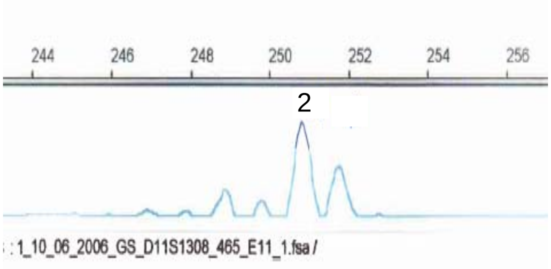

Q14

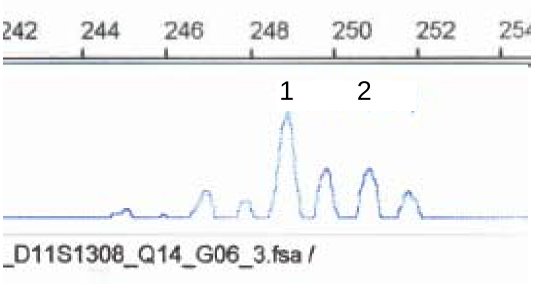

USH1C, D11S4130

Acadian

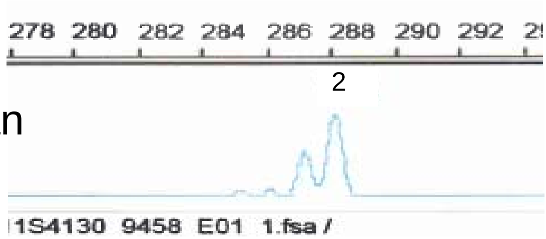

1172

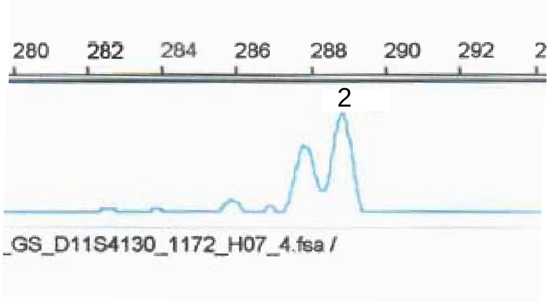

367

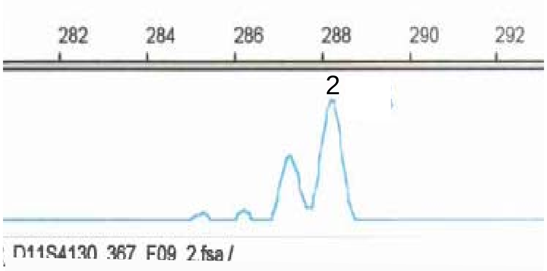

554

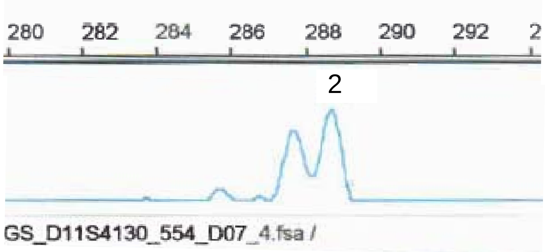

1116

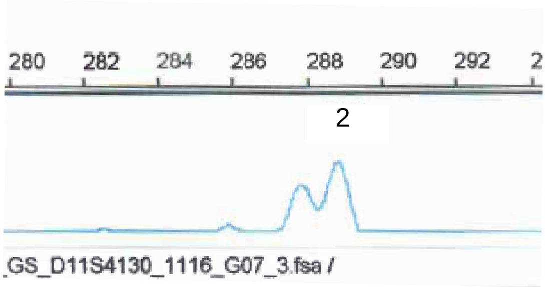

475

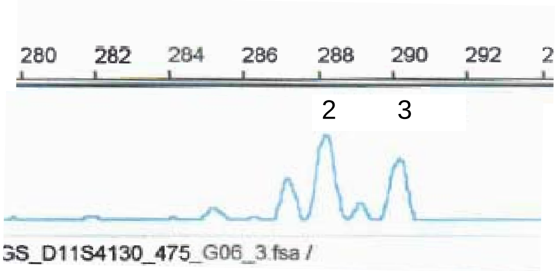

505

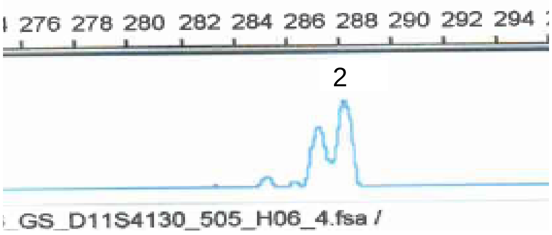

1115

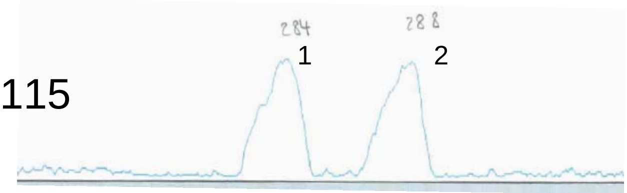

848

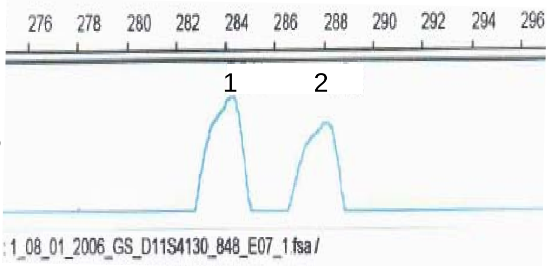

465

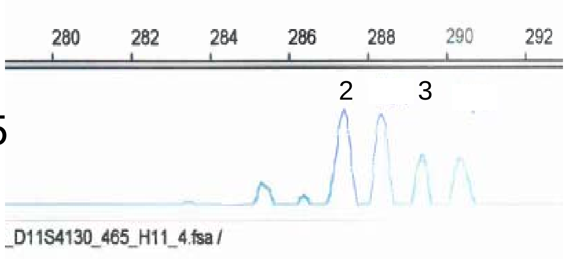

Q14

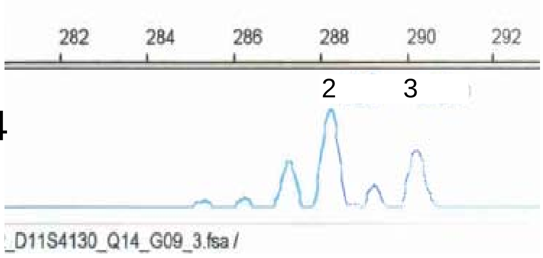

***USH1C*, rs2240489**

Acadian

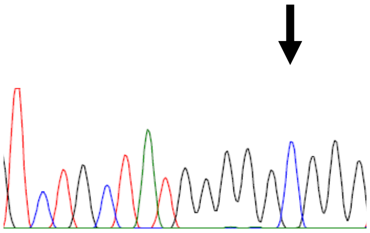

505

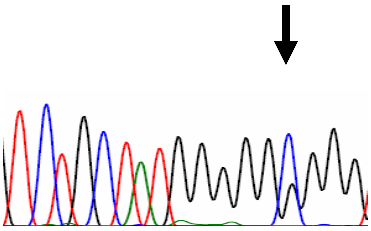

1172

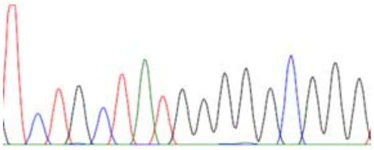

1115

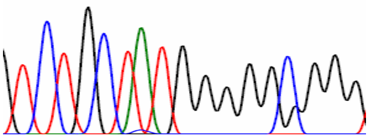

367

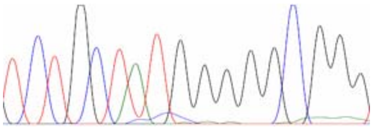

848

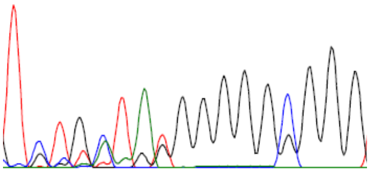

554

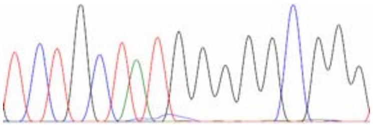

465

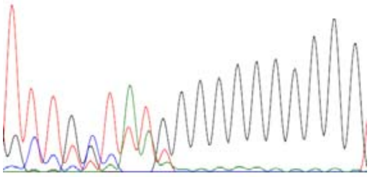

1116

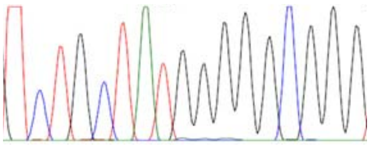

Q14

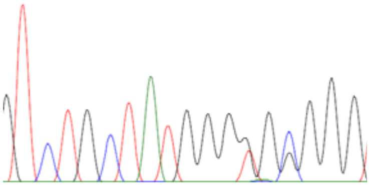

475

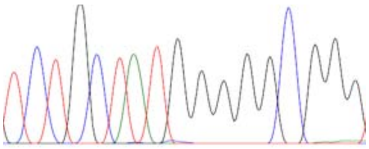

***USH1C*, rs17777540**

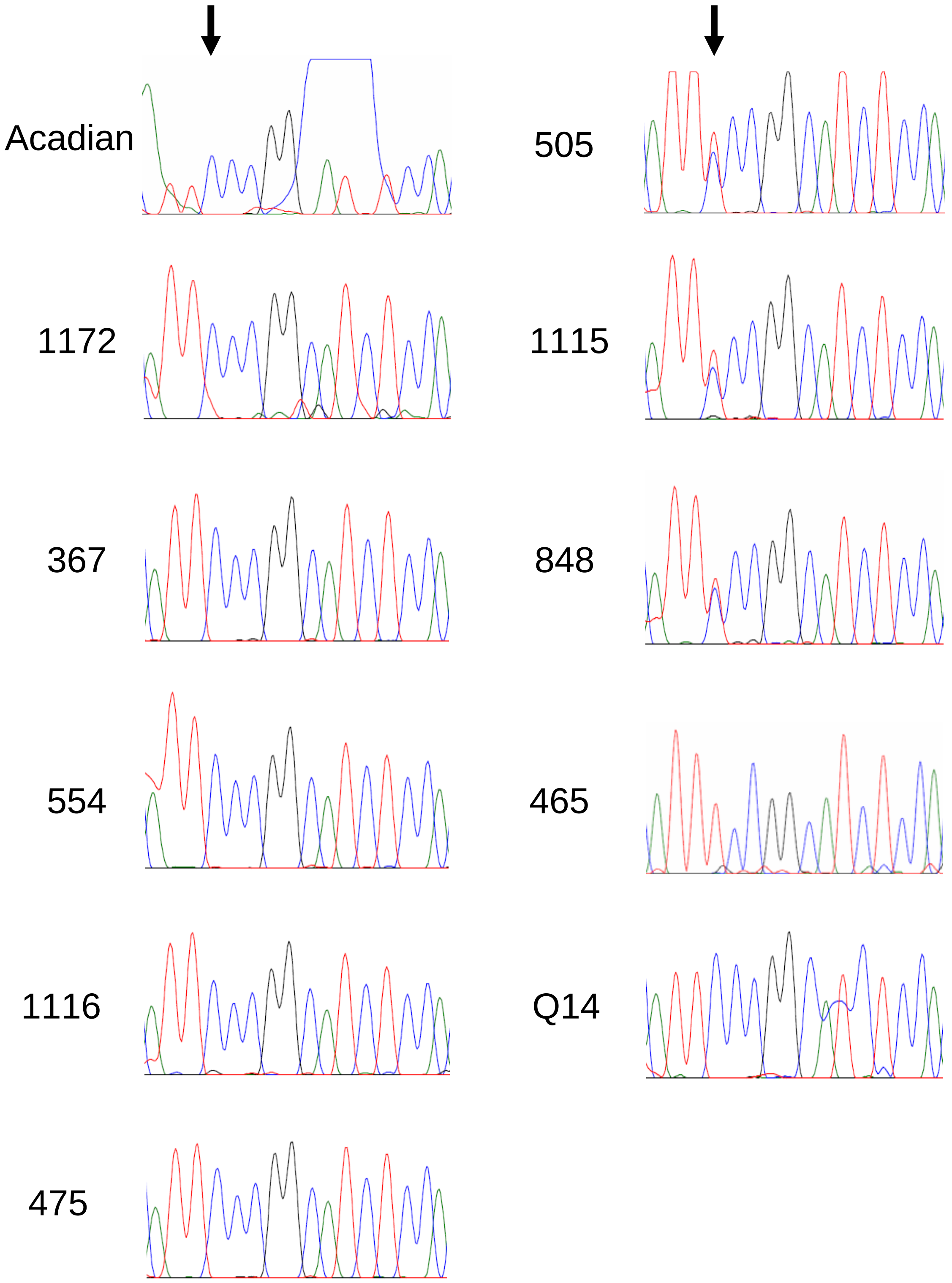

VNTR

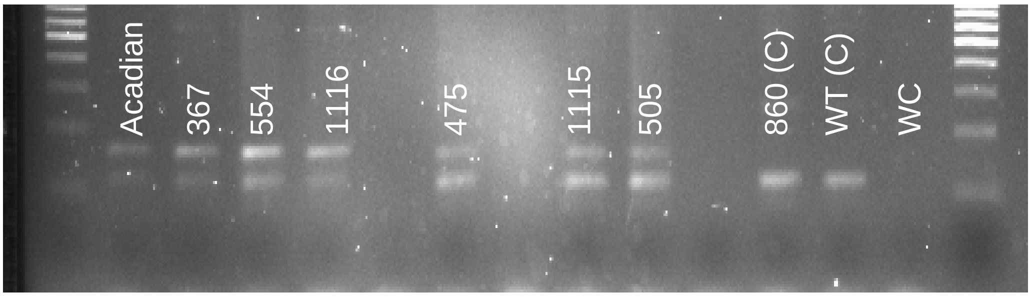

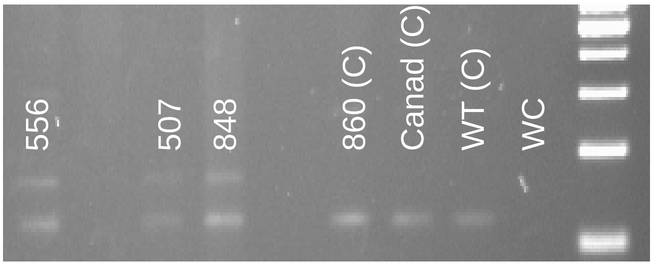

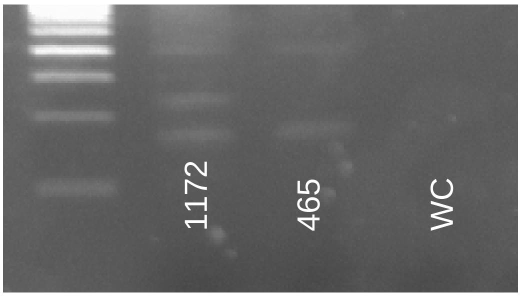

860 (C):	patient with two CDH23 mutant alleles as control
WT (C):	healthy control individual
WC:	water control

***USH1C*, rs5789990**

Acadian

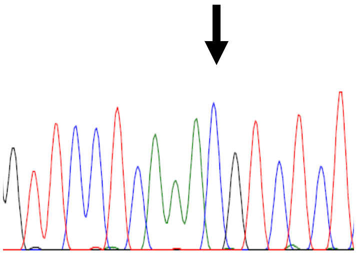

505

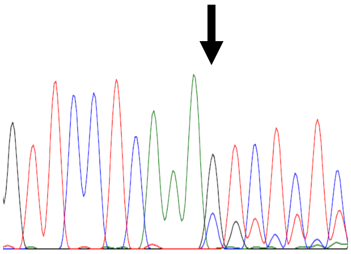

1172

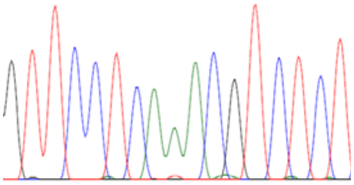

1115

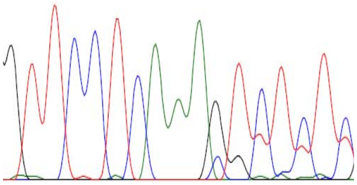

367

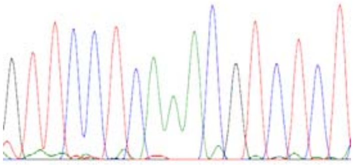

848

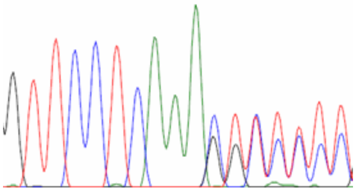

554

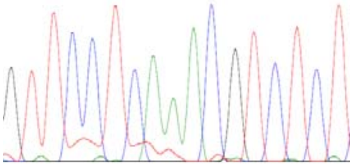

465

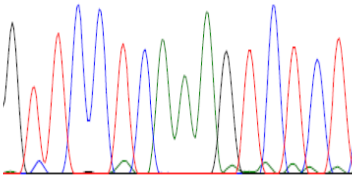

1116

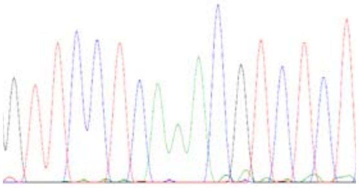

Q14

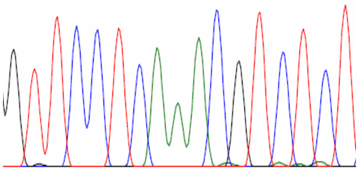

475

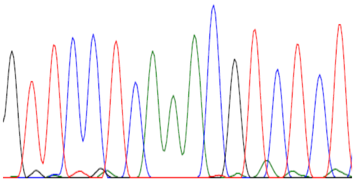

***USH1C*, rs2240488**

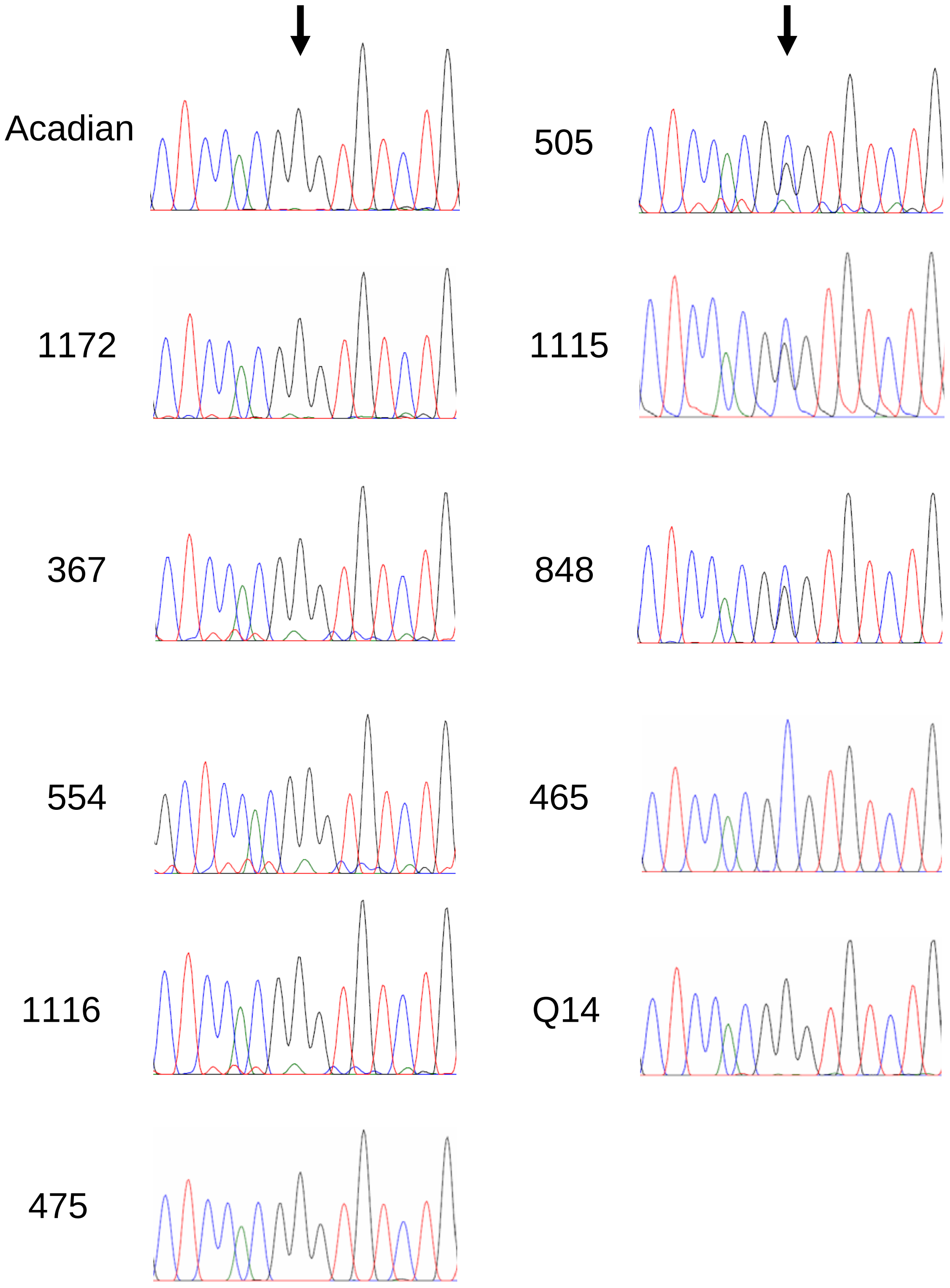

***USH1C*, rs2041032**

Acadian

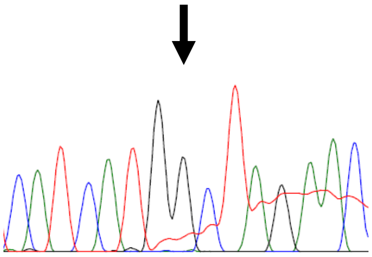

505

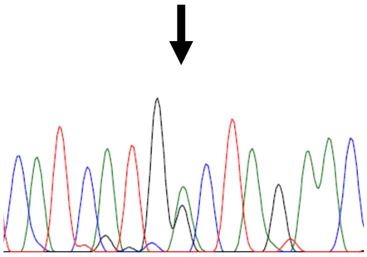

1172

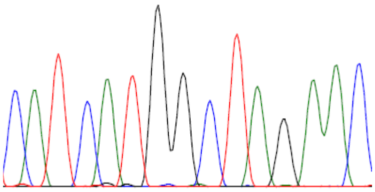

1115

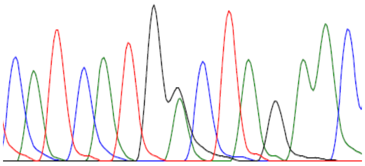

367

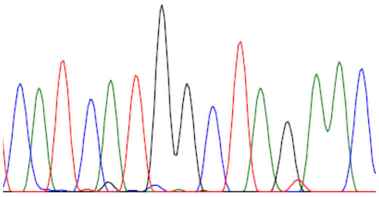

848

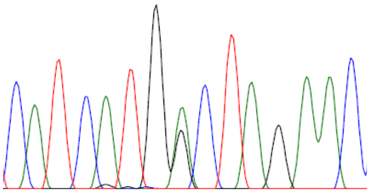

554

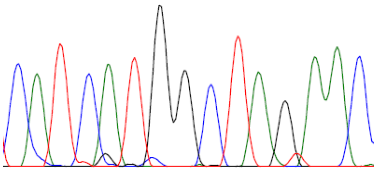

465

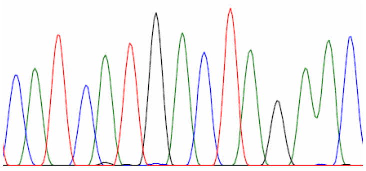

1116

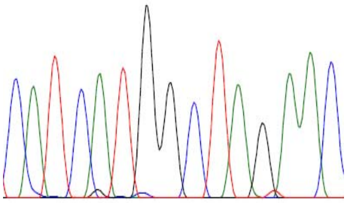

Q14

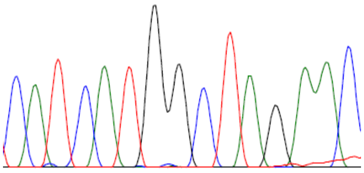

475

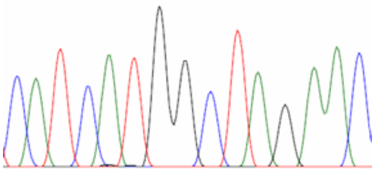

***USH1C*, rs2041031 and rs2108332**

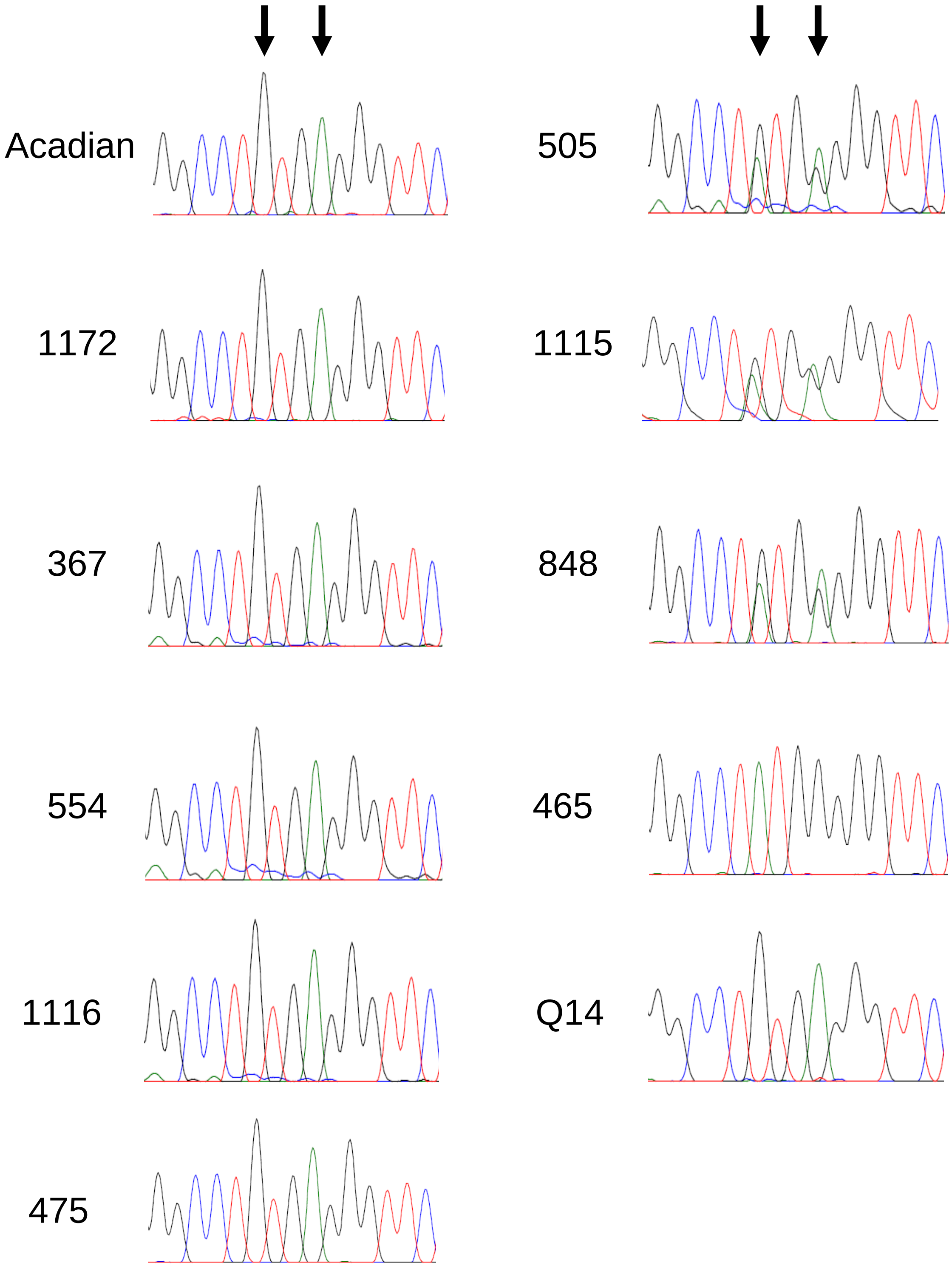

***USH1C*, rs17851376**

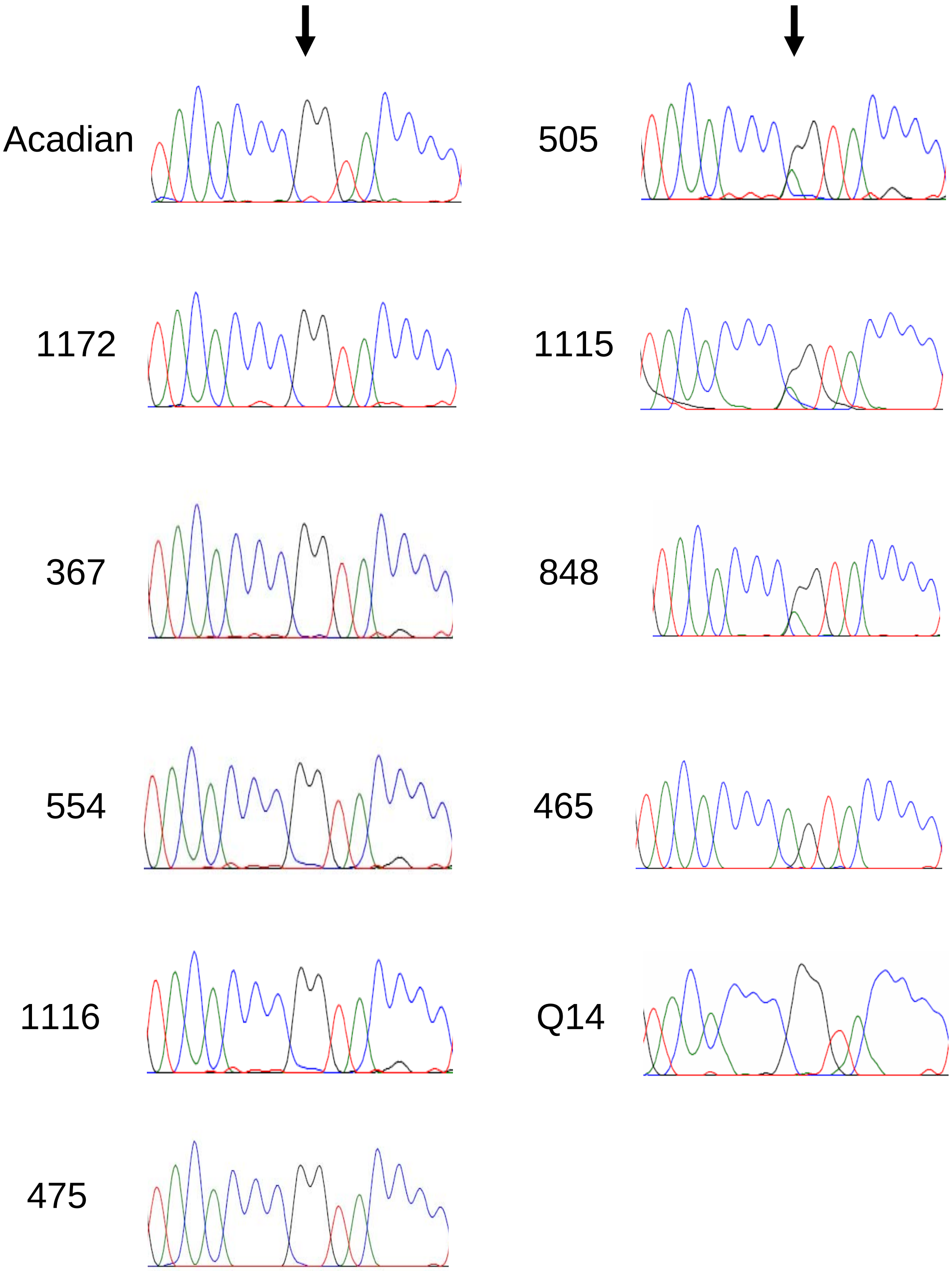

***USH1C*, rs2190454**

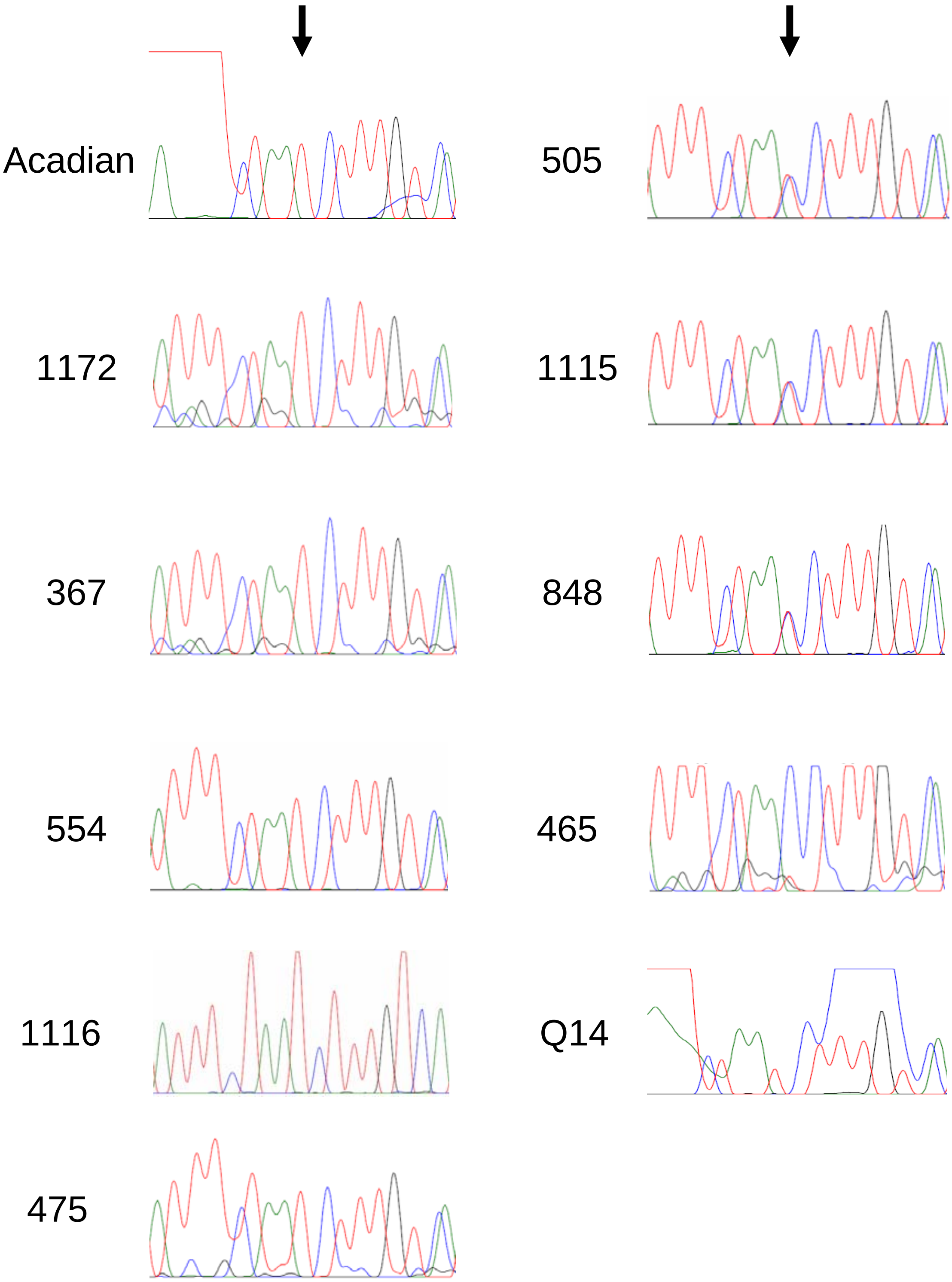

***USH1C*, rs2190453**

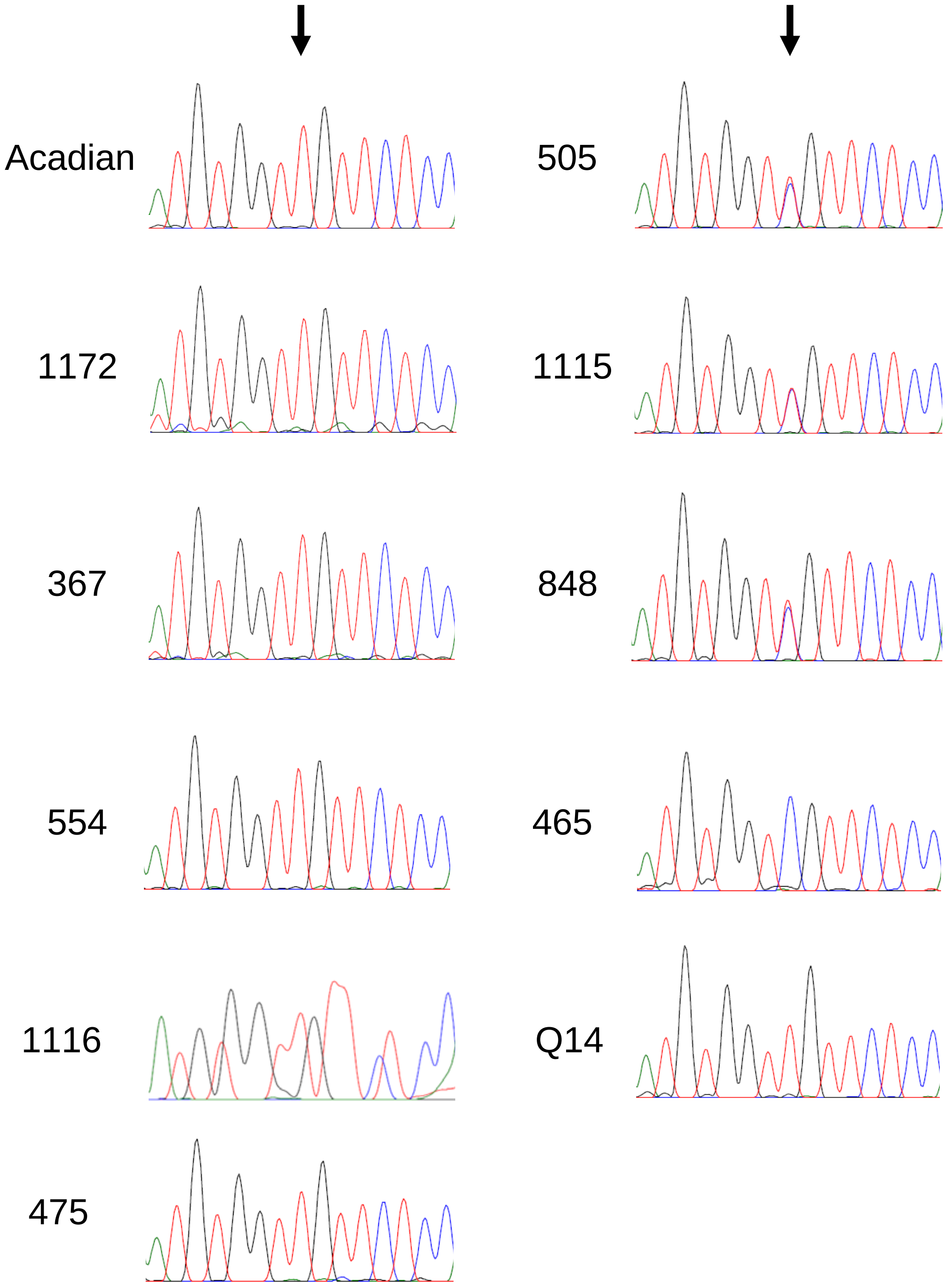

***USH1C*, rs2237965**

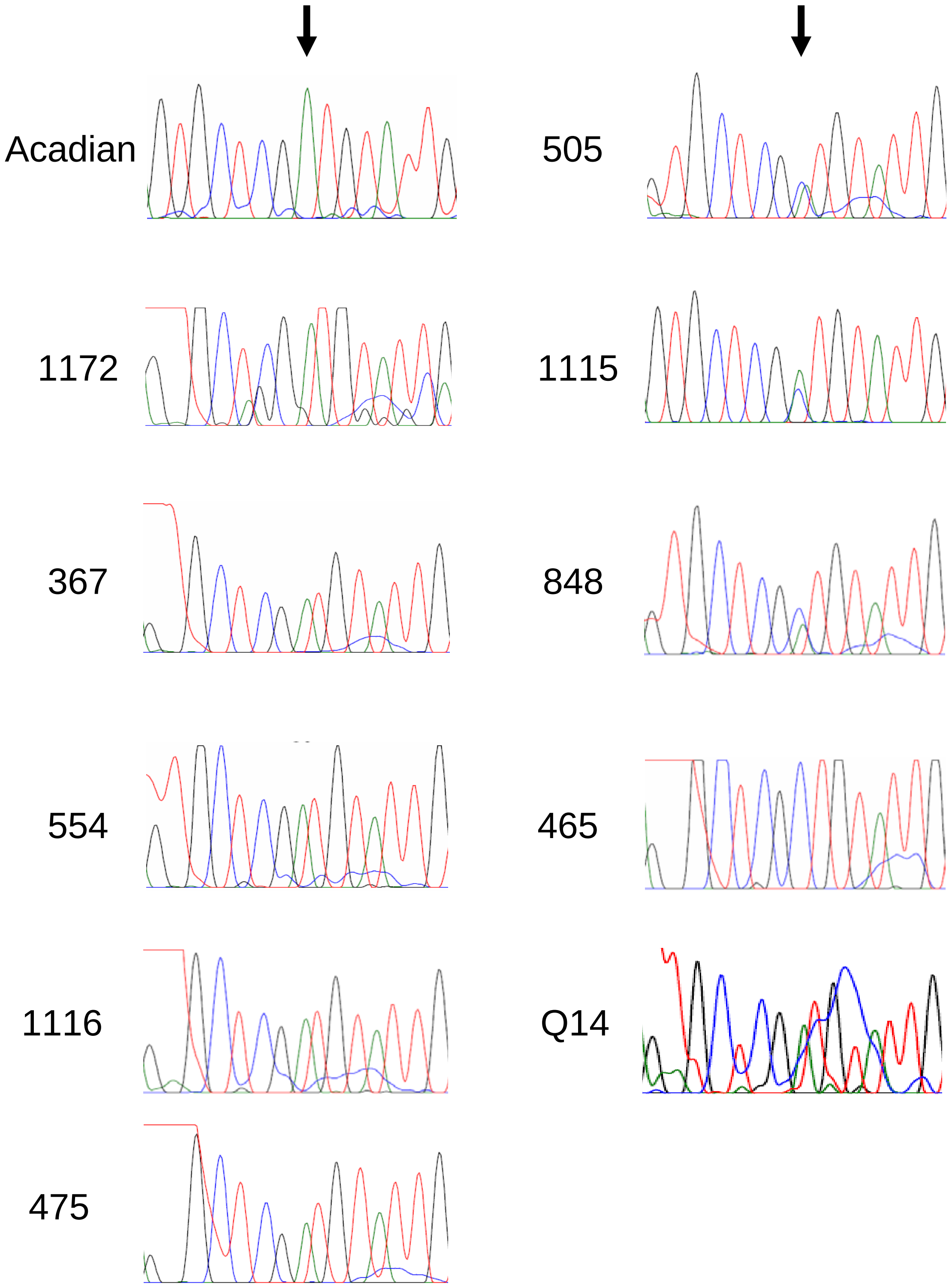

***USH1C*, rs10832796**

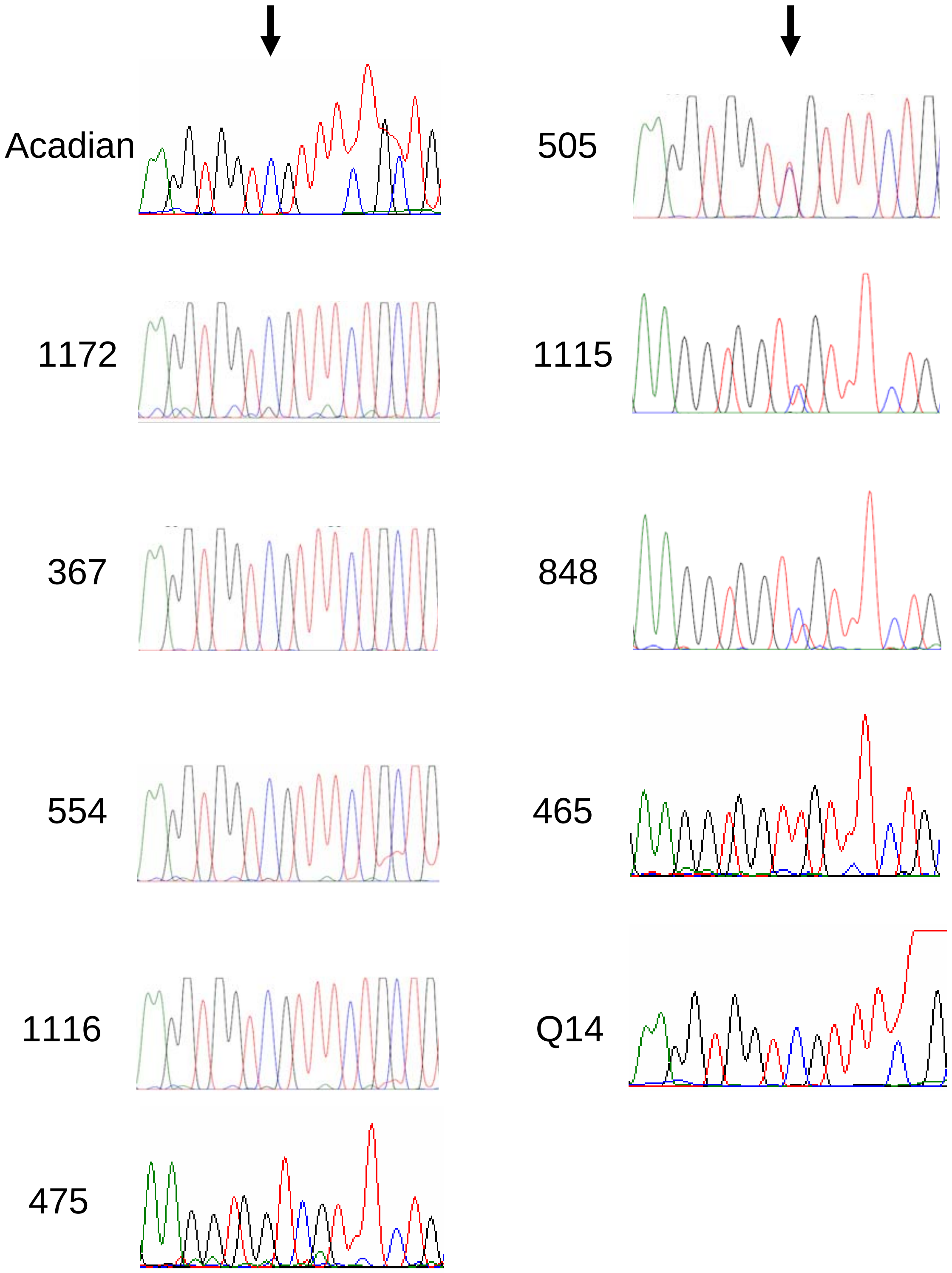

USH1C, rs1064074

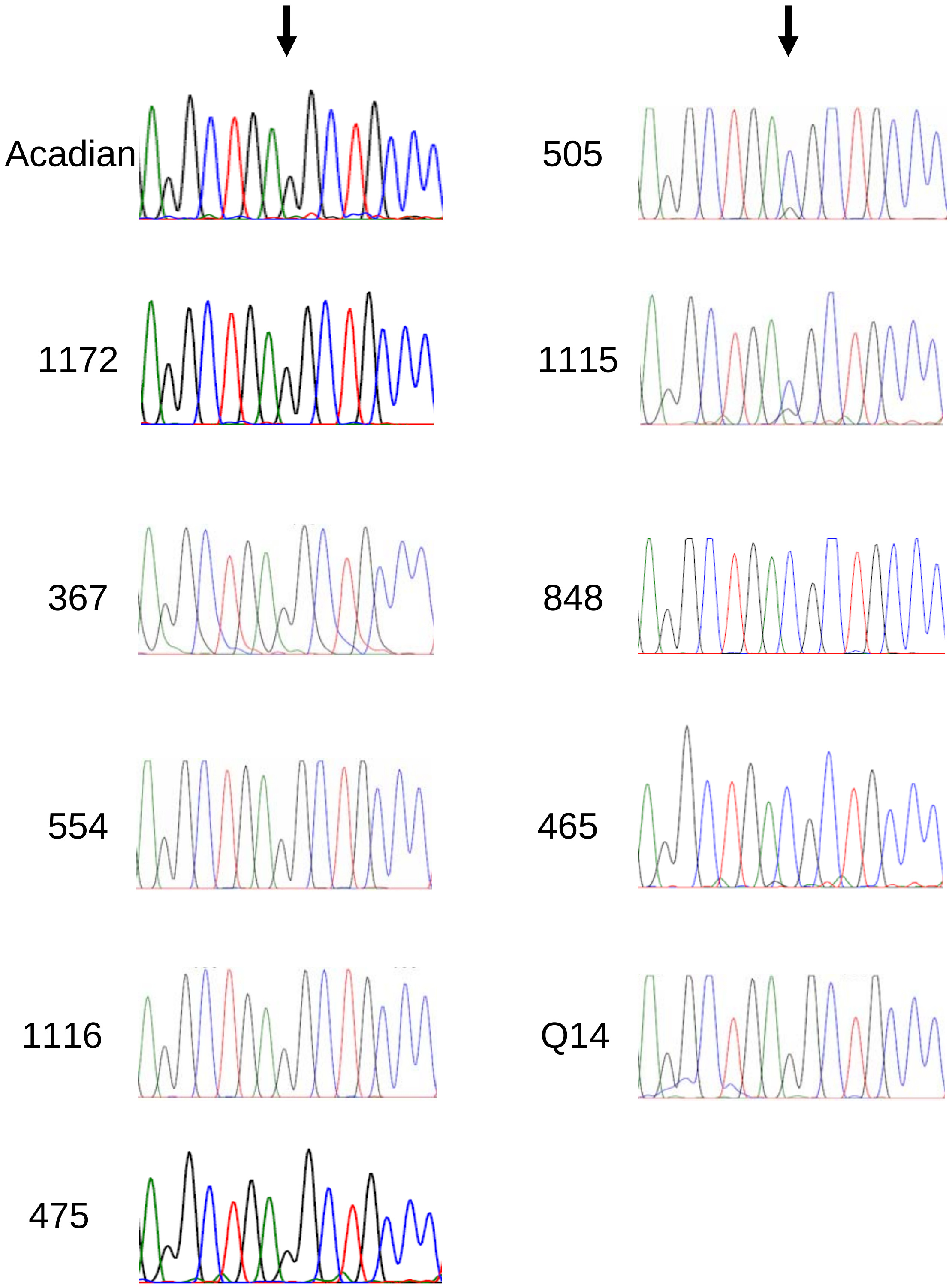

***USH1C*, rs1055574**

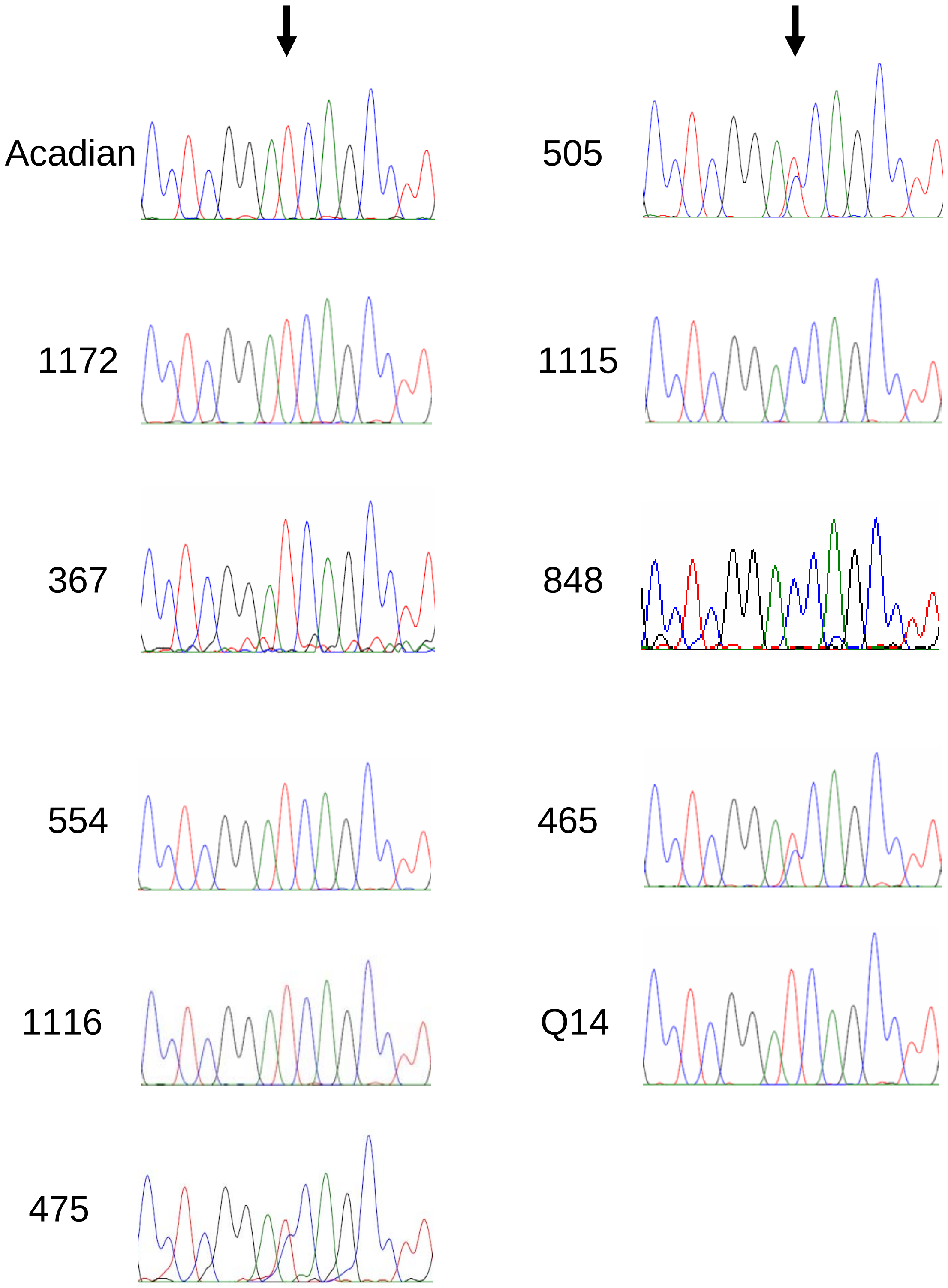

***USH1C*, rs1055577**

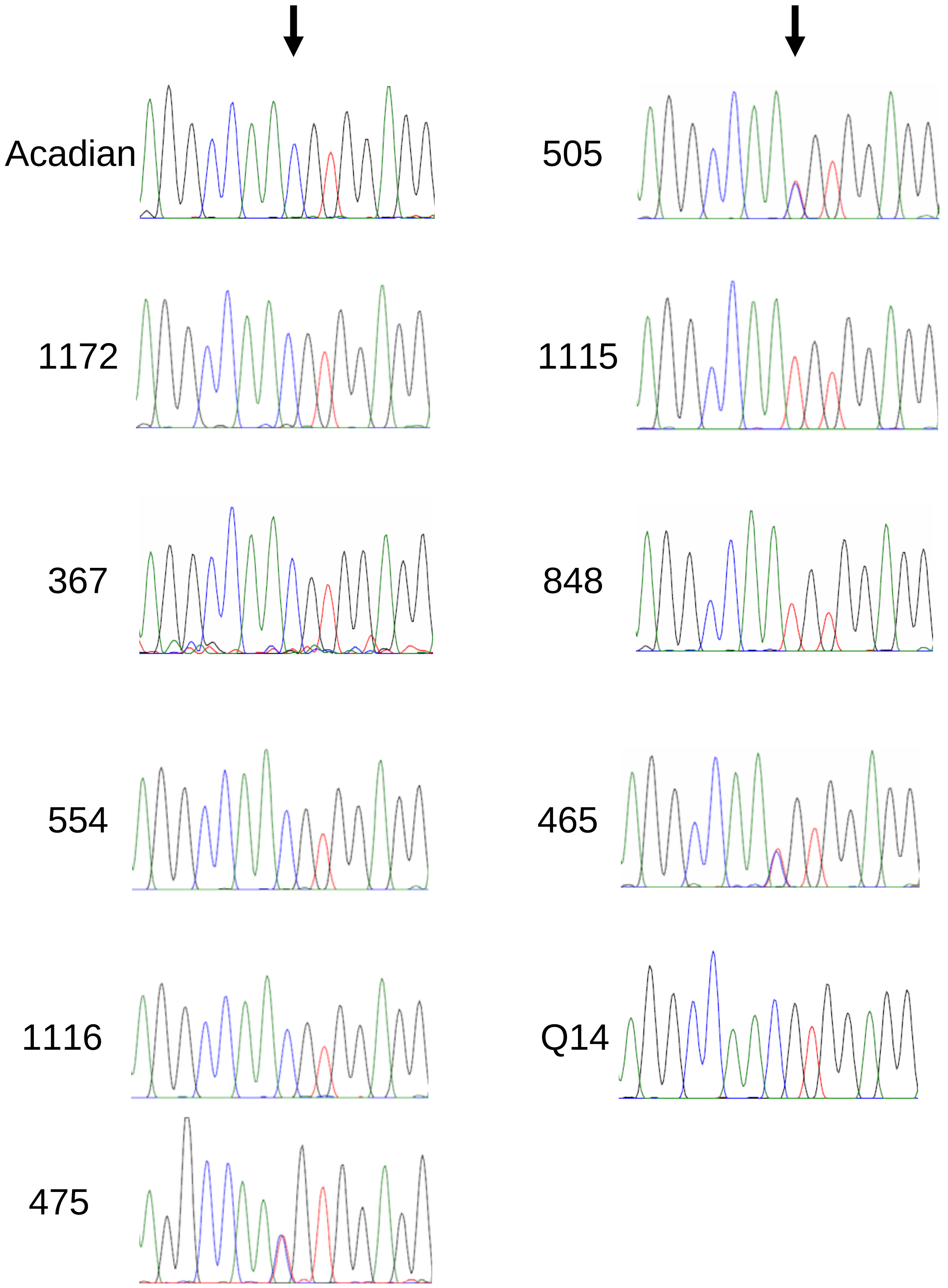

***USH1C*, rs1055581**

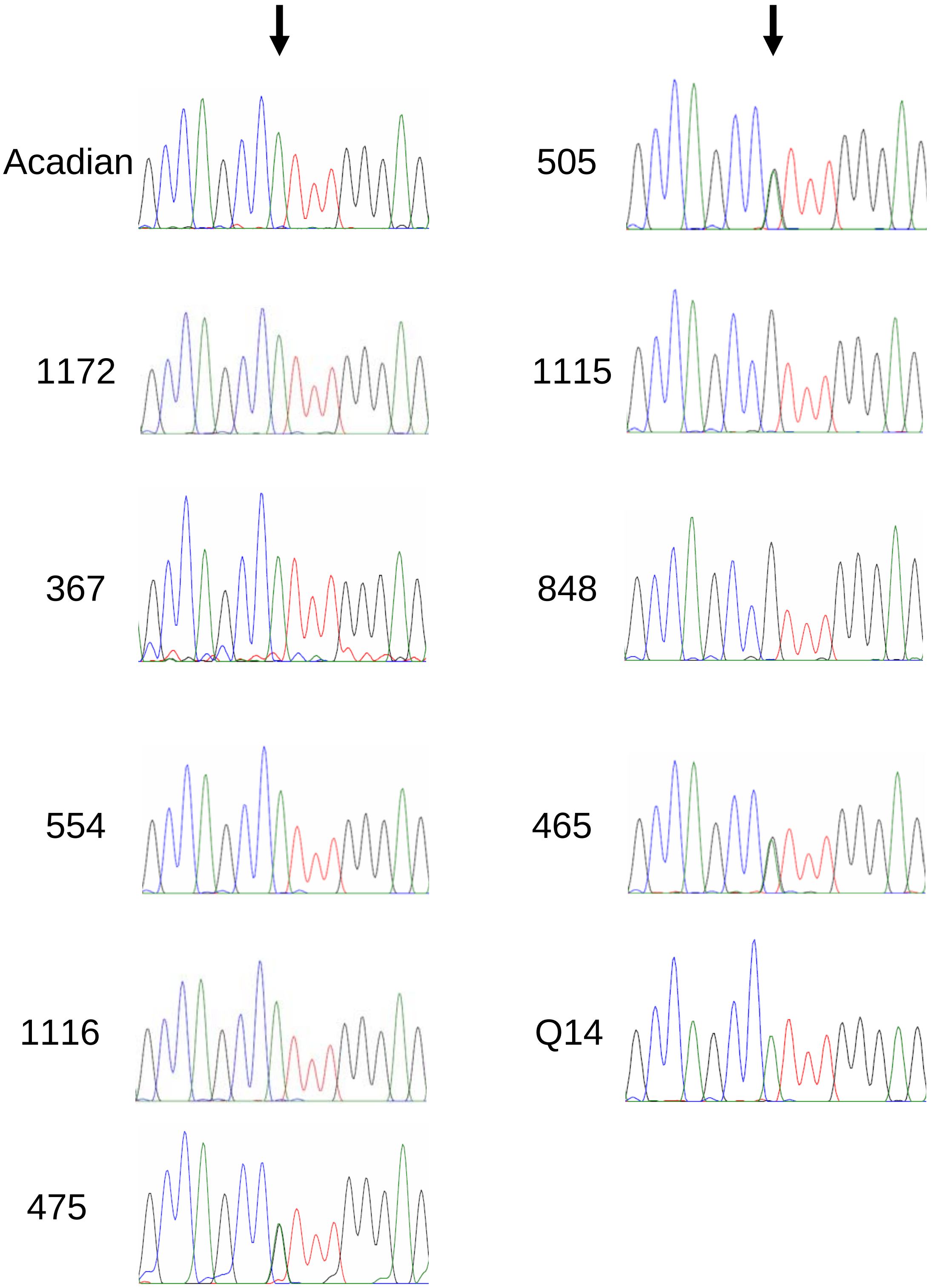

***USH1C*, D11S902**

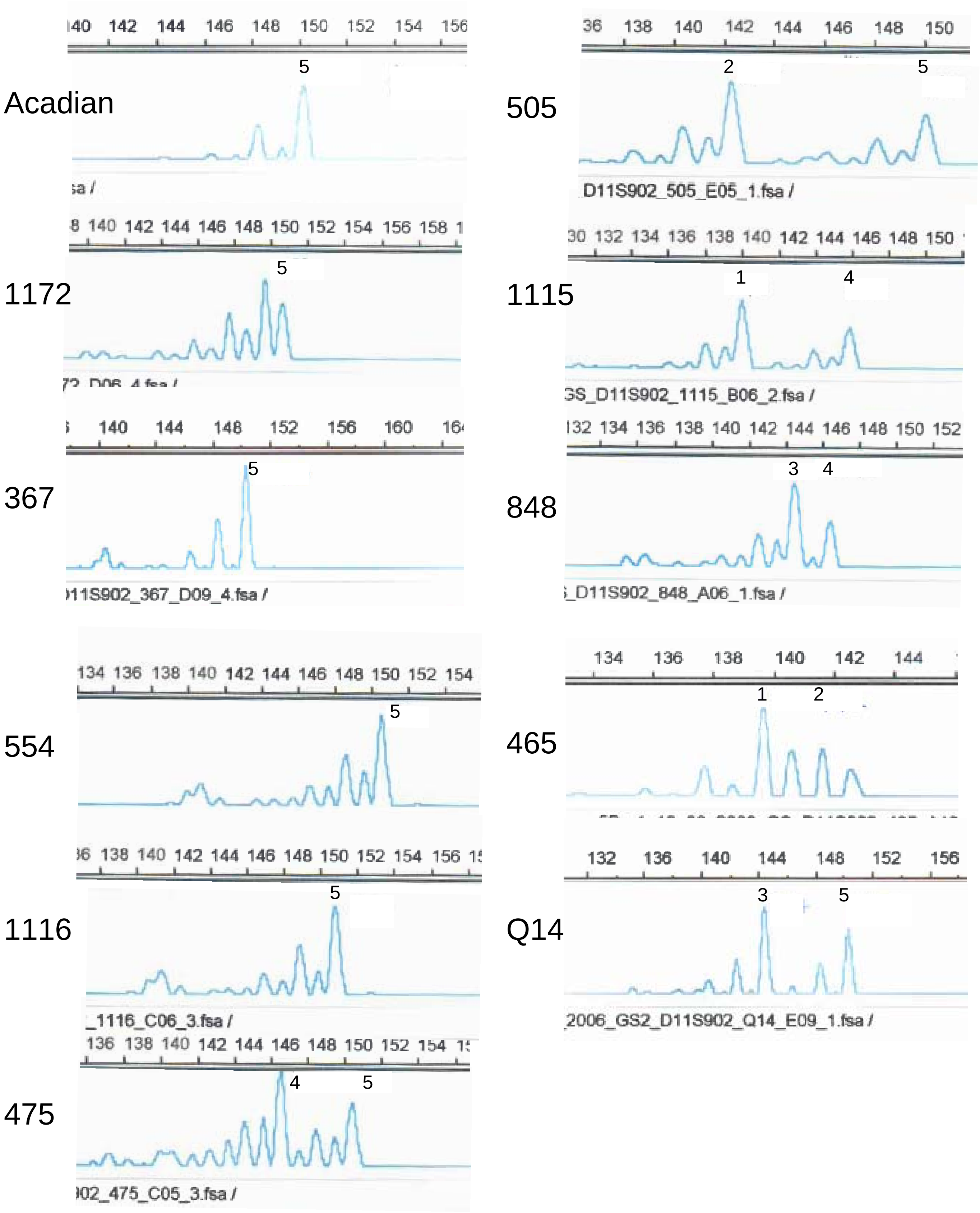

***USH1C*, D11S4193**

Acadian

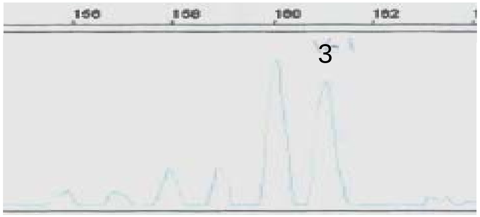

505

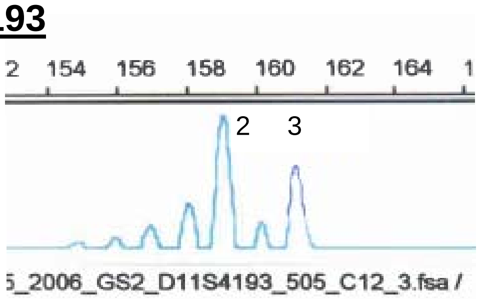

1172

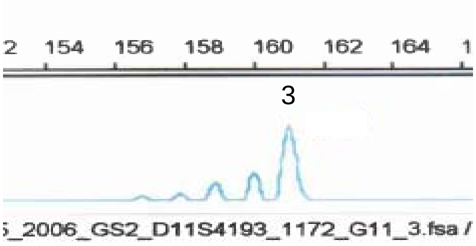

1115

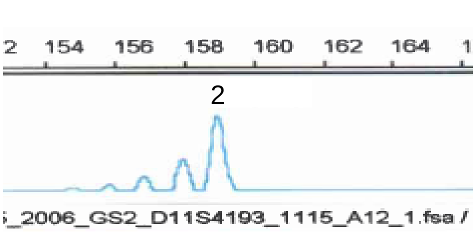

367

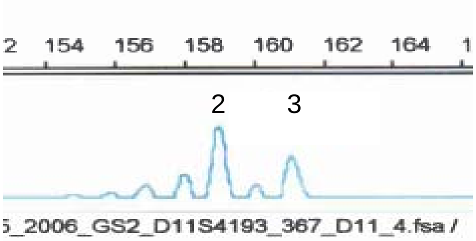

848

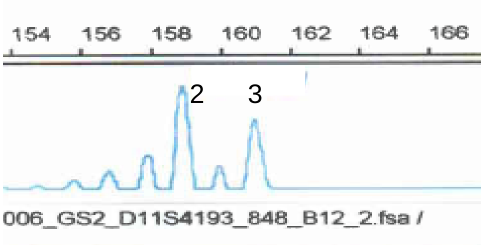

554

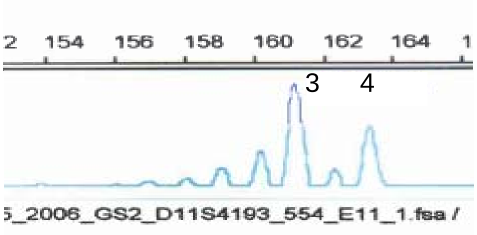

465

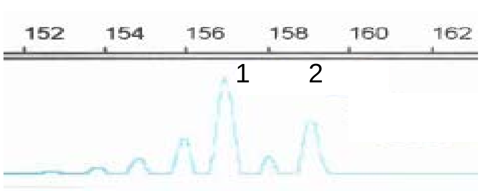

1116

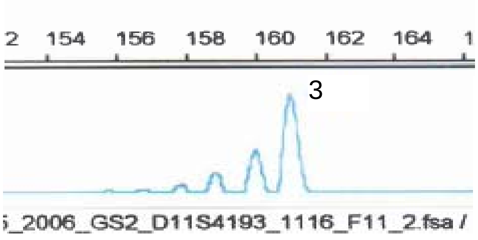

Q14

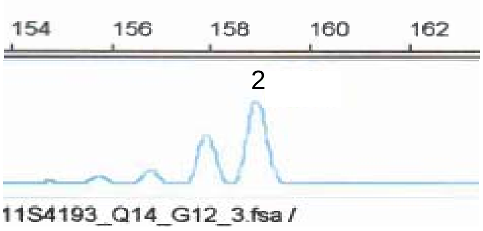

475

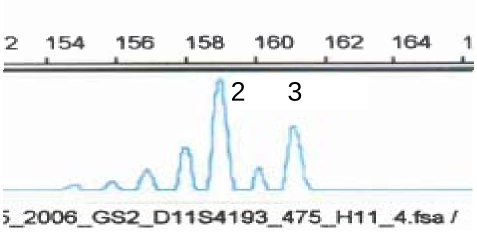

***USH1C*, D11S1349**

Acadian

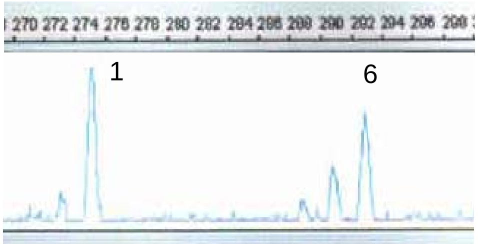

505

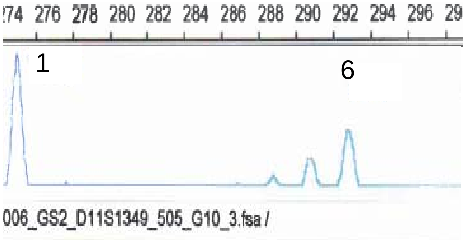

1172

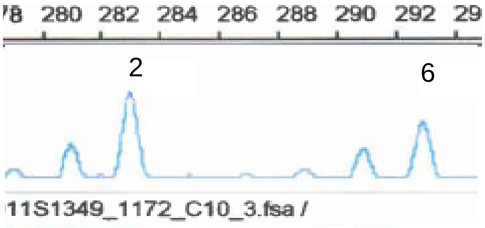

1115

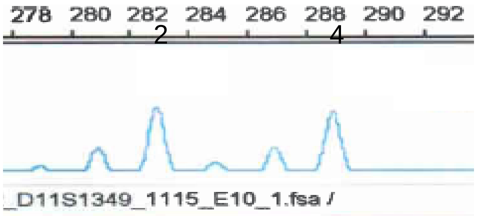

367

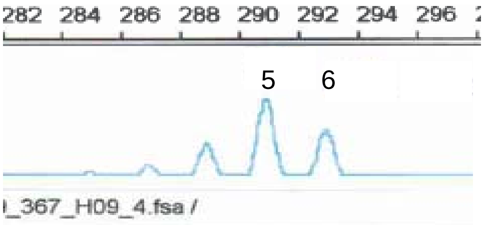

848

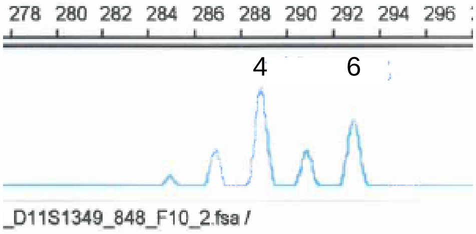

554

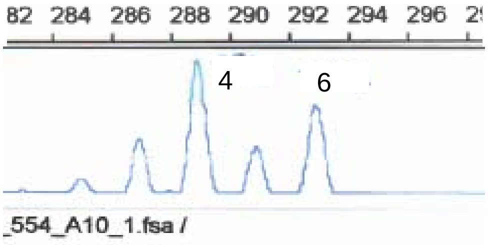

465

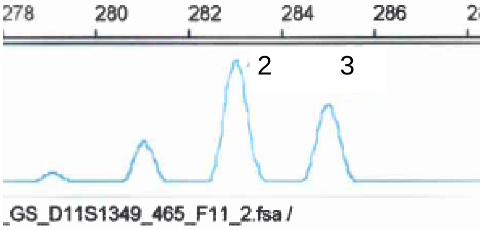

1116

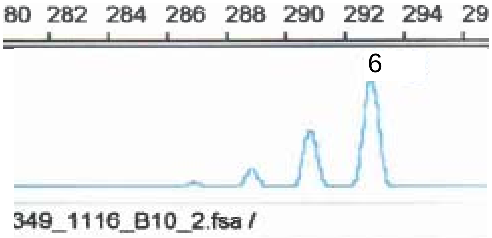

Q14

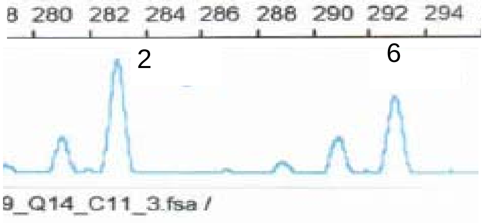

475

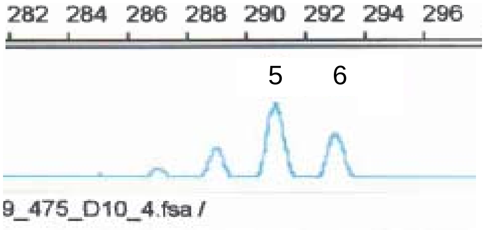

***CDH23* (*USH1D*) haplotypes**

Positions of the respective SNPs are indicated by arrows.

USH1D, D10S529

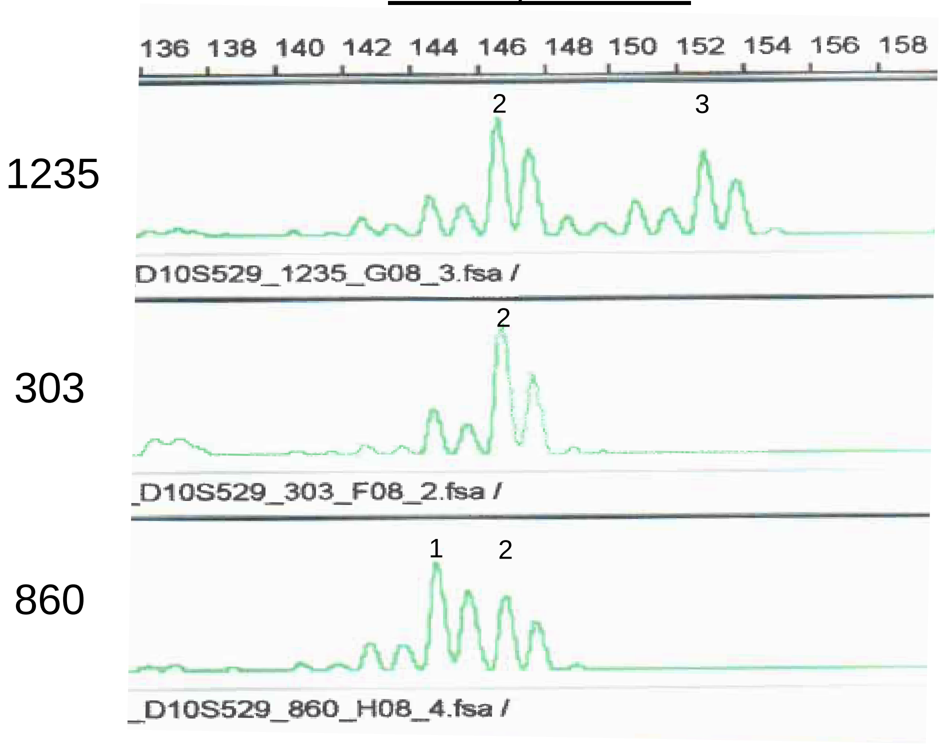

USH1D, D10S1688

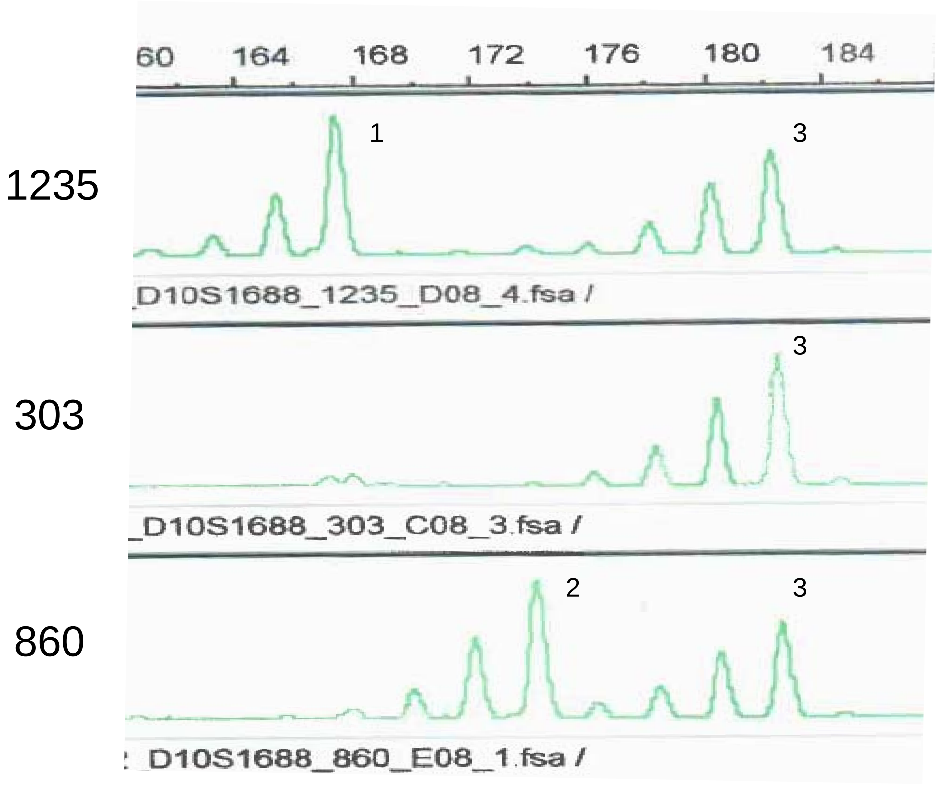

***USH1D*, D10S1759**

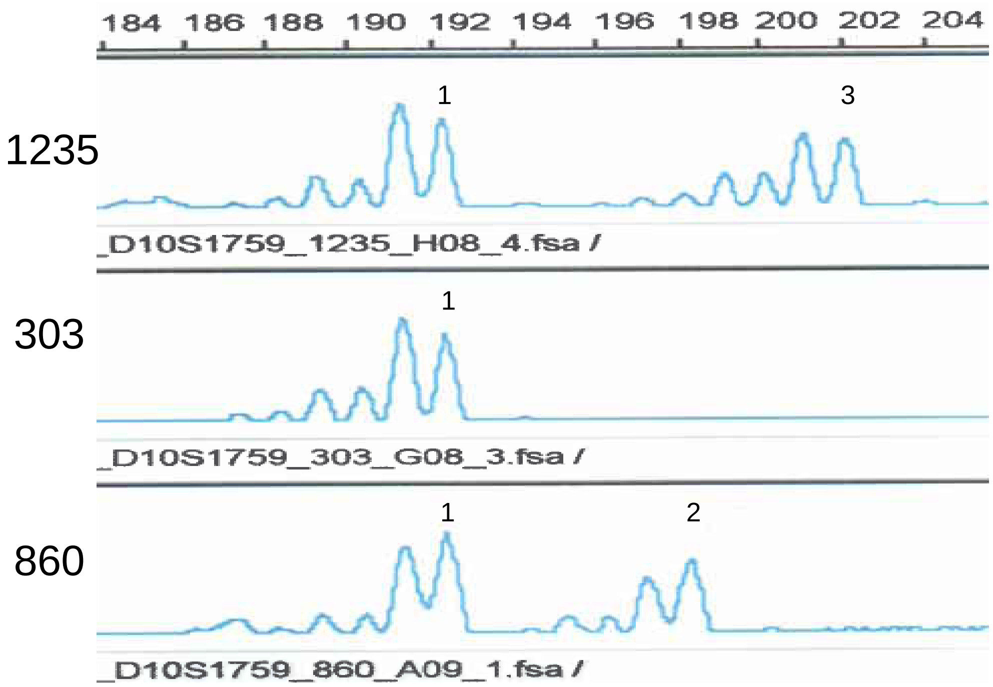

***USH1D*, D10S1694**

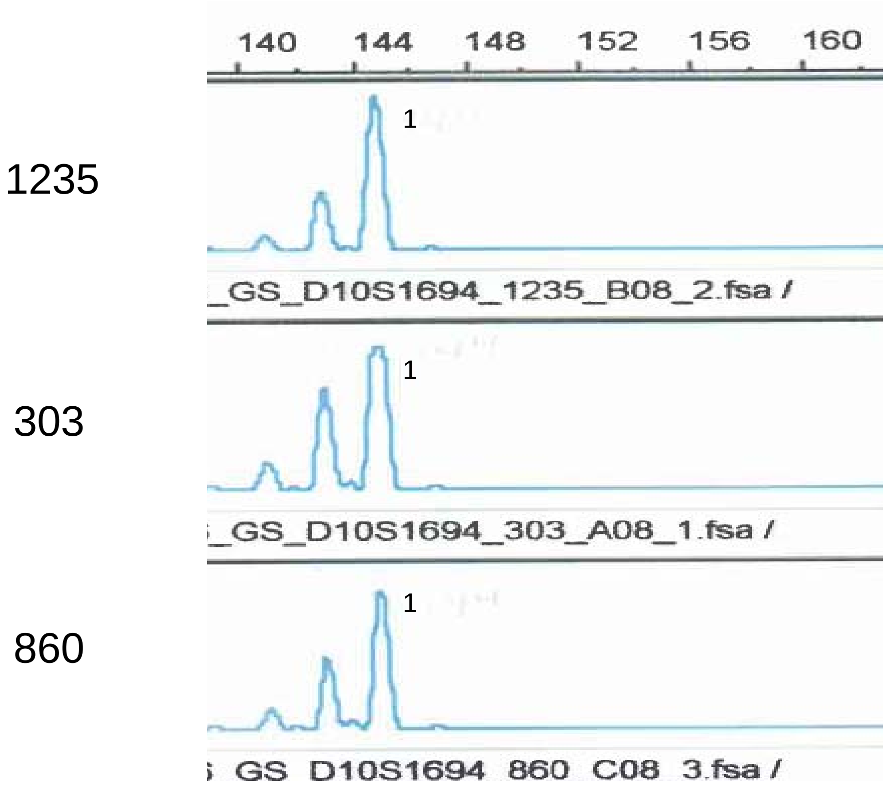

USH1D, rs3802717

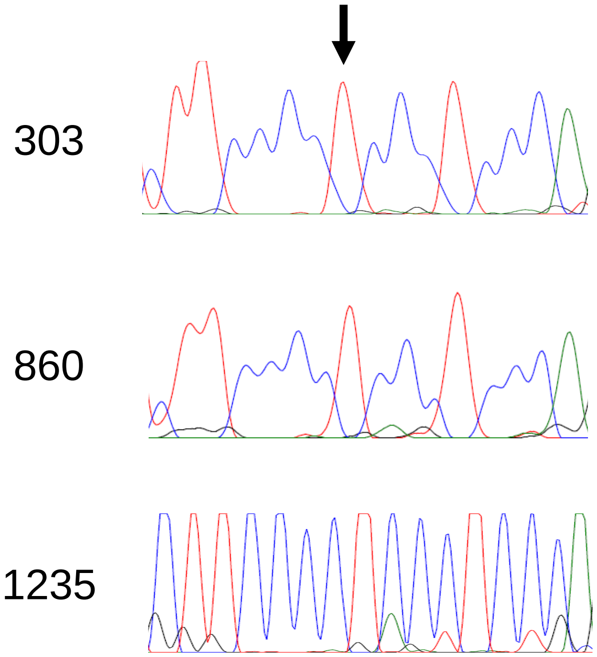

USH1D, c.5712G>A

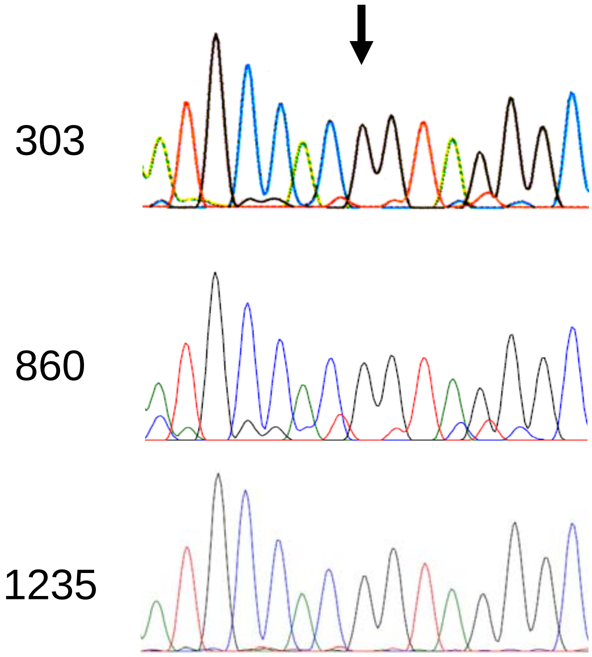

USH1D, rs11592462

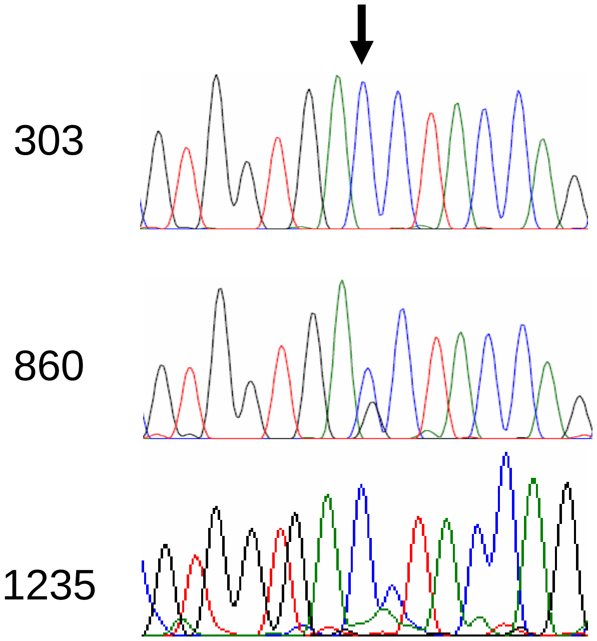

USH1D, IVS45-9G>A

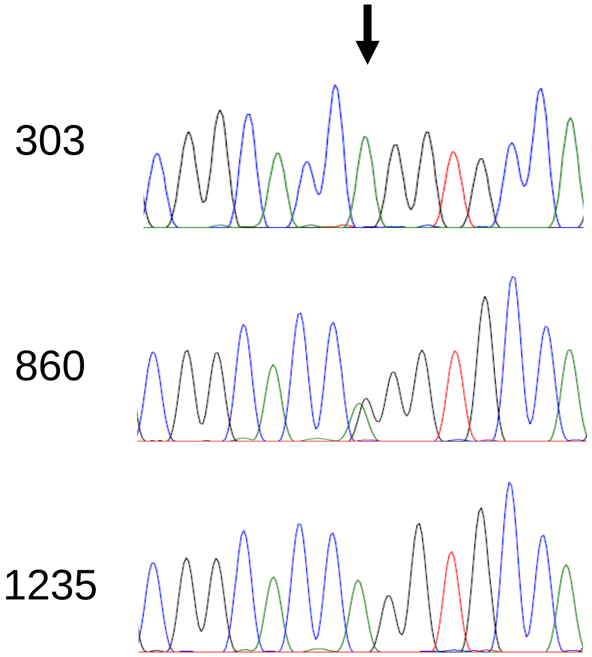

***USH1D*, rs7068357**

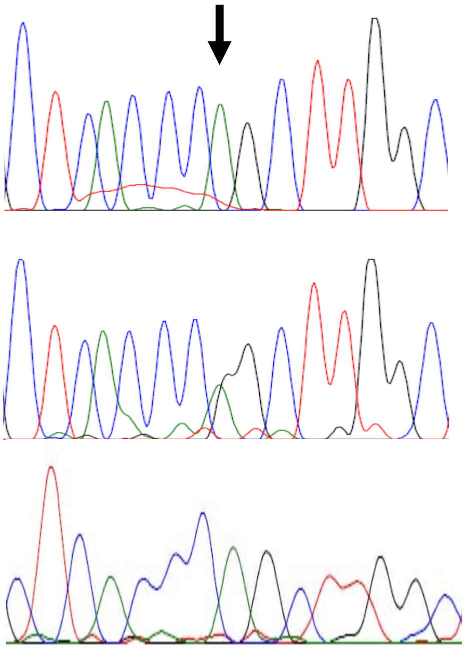

303

860

1235

***USH1D*, rs4747193**

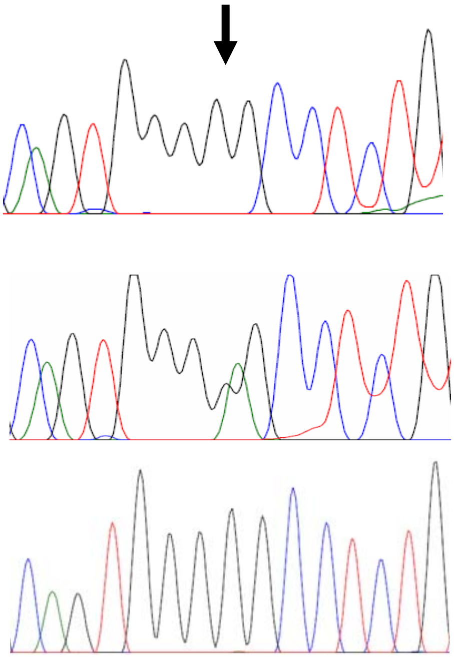

303

860

1235

***USH1D*, rs4747194**

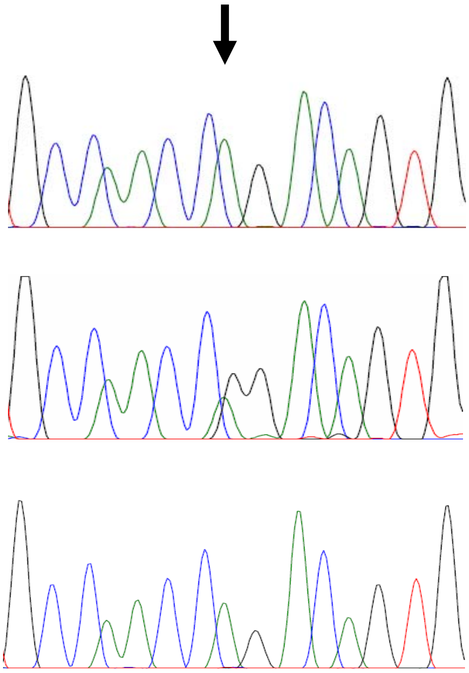

303

860

1235

***USH1D*, rs4747195**

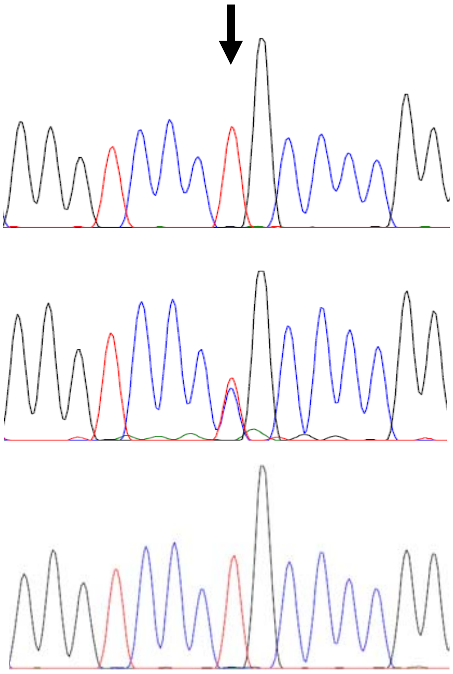

303

860

1235

***USH1D*, rs10823849**

303

860

1235

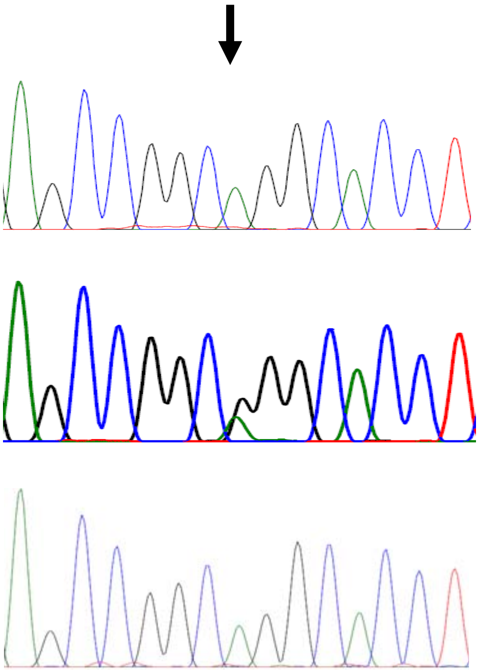

***USH1D*, IVS60+8G>A**

303

860

1235

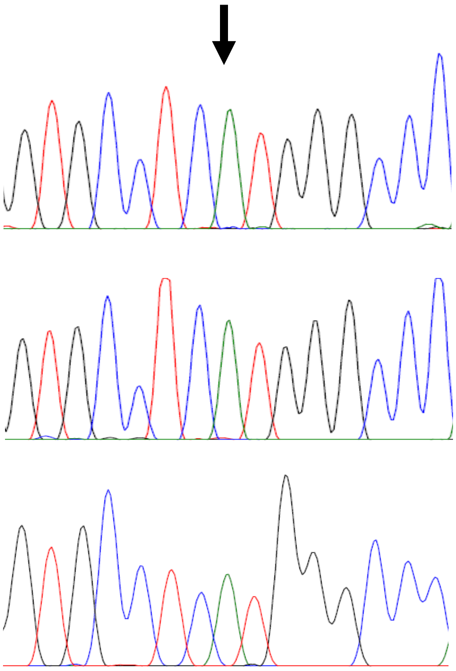

***USH1D*, D10S218**

1235

303

860

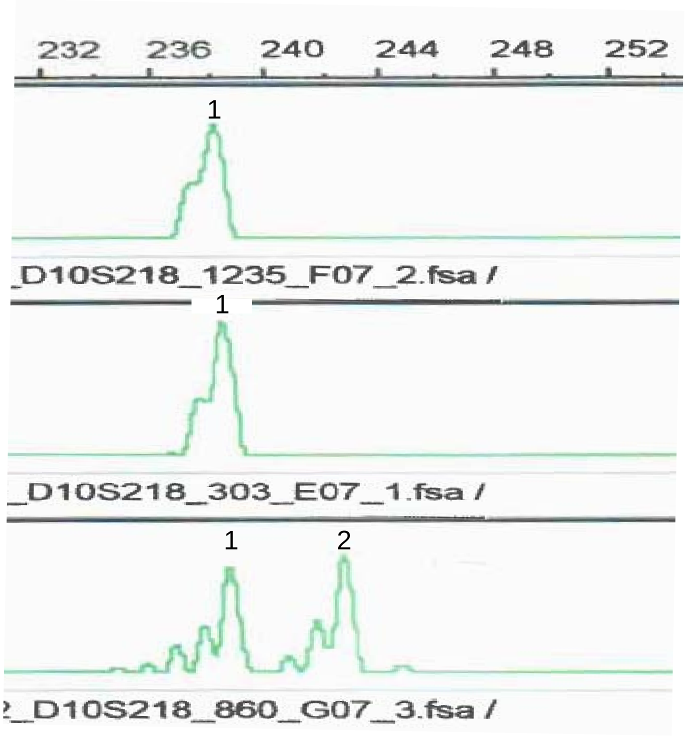

Supplement: Additional data file 5 — Fragment length analysis is shown for microsatellite markers (GeneScan, Applied Biosystems), electropherograms for SNPs, and agarose gel electrophoresis for the VNTR in intron 5 of the USH1C gene. [file gb-2007-8-4-r47-S5.pdf]
