# Supplementary material for: Deafblindness in French Canadians from Quebec: a predominant founder mutation in the USH1C gene provides the first genetic link with the Acadian population
Source: Genome Biol. 2007 Apr 3;8(4):R47. doi: 10.1186/gb-2007-8-4-r47 (PMC1895989; doi:10.1186/gb-2007-8-4-r47)

Deafblindness in French Canadians from Quebec: A predominant founder mutation in the *USH1C* gene provides the first genetic link with the Acadian population

Ebermann et al.

RAW DATA

	page
Haplotypes	
USH1C haplotypes	2 – 23
USH1D haplotypes	24 – 29
USH1 mutations	30 – 36
Genotyping of healthy French Canadian Controls	
Controls for c.216G>A (*USH1C*)	37 – 40
Controls for c.238-239insC (*USH1C*)	41 – 58
Controls for c.496+1G>T (*USH1C*)	59 – 60
Controls for p.R155X (*USH1C*)	61 – 62
Controls for c.748-759+5del (*USH1C*)	63 – 64
Controls for IVS45-9G>A (*CDH23*)	65 – 73
Controls for p.R736X (*CDH23*)	74 – 82
Controls for p.A457V (*MYO7A*)	83 – 91
Controls for p.Q815X (*MYO7A*)	92 – 93
Controls for p.A123D (*USH3A*)	94 – 102
Mutation screening in USH1 genes in patient 1881	103 – 204

USH1 gene mutations identified in this study

***USH1C*, c.216G>A**

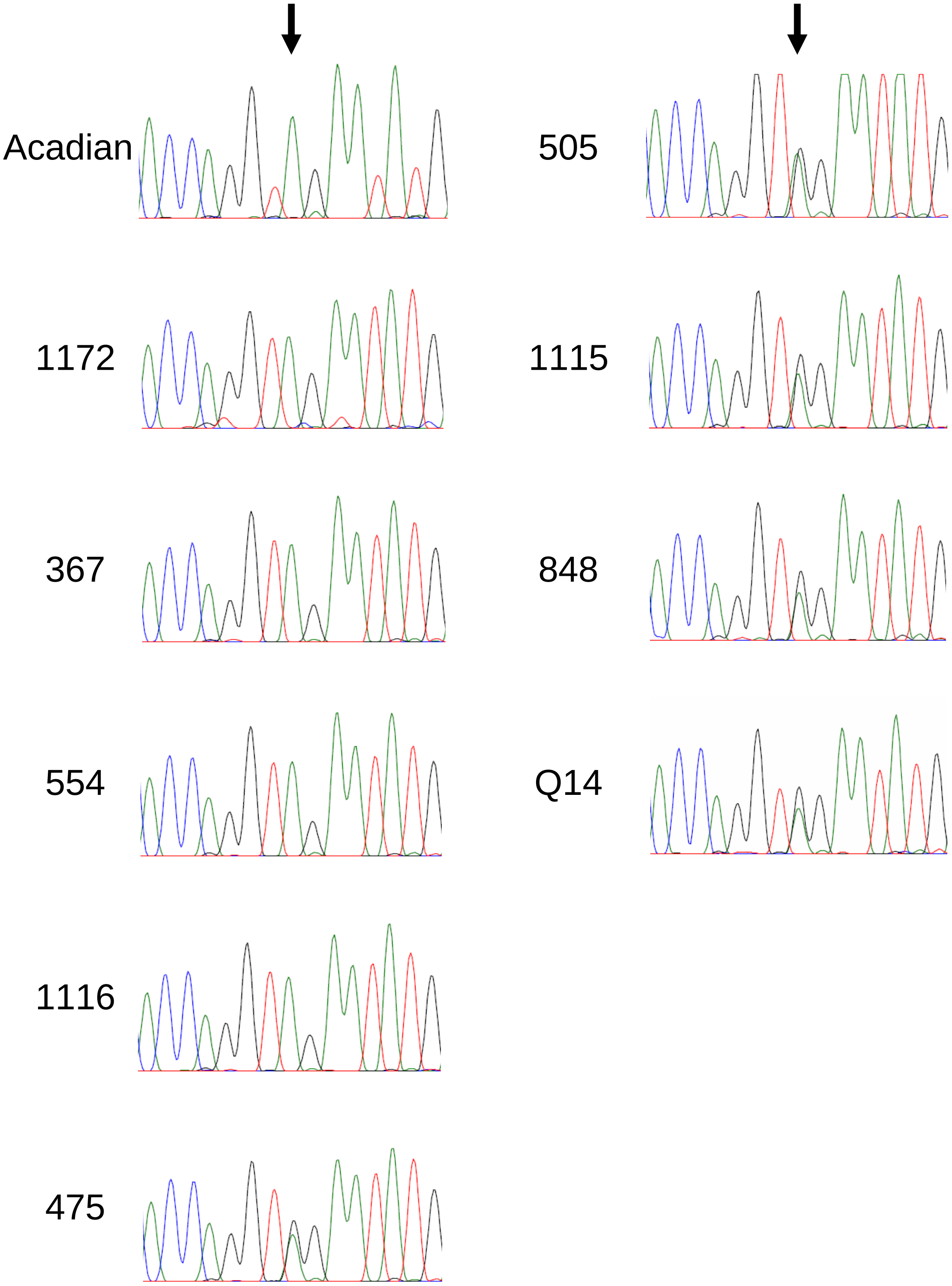

USH1C, c.238-239insC

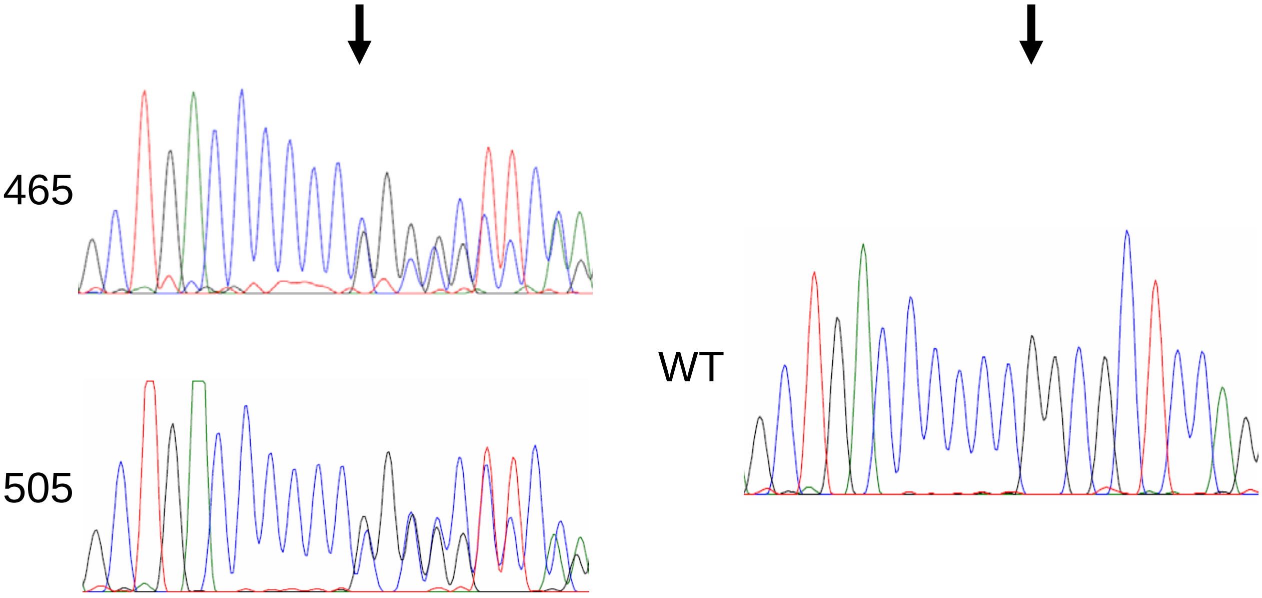

USH1C, c.496+1G>T

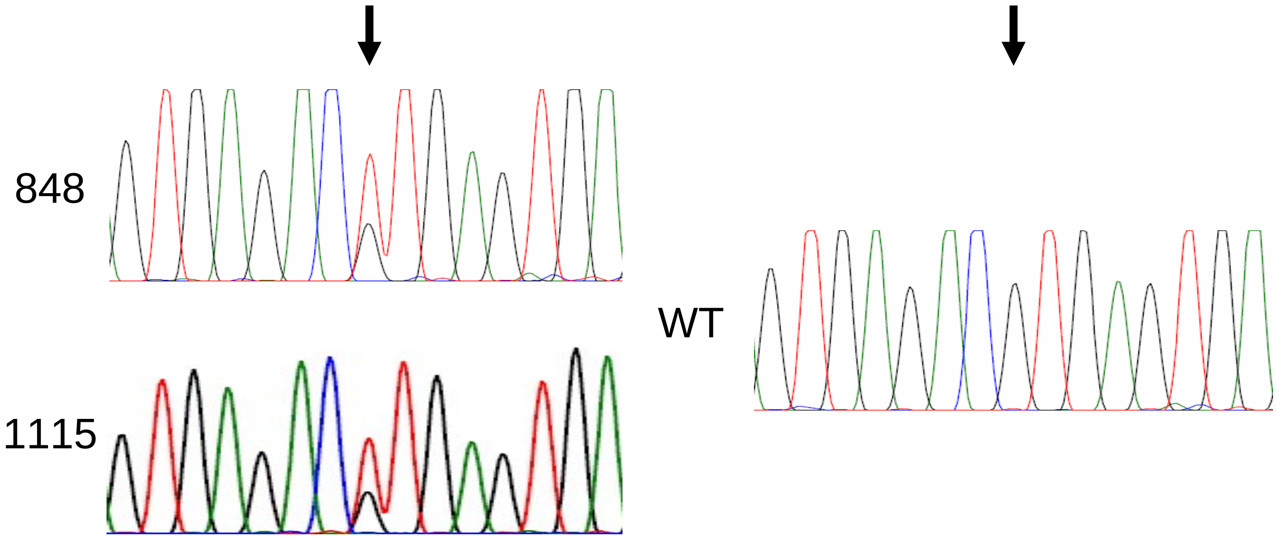

<u>*USH1C*, c.463C>T (p.R155X)</u>

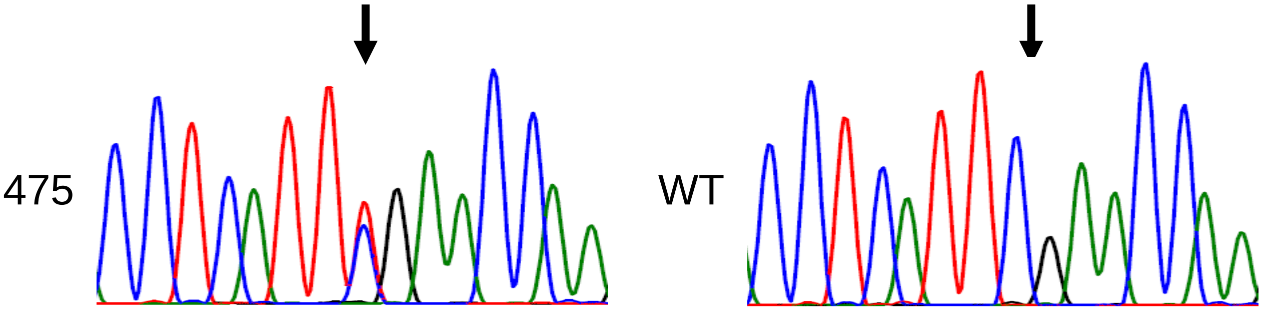

<u>*USH1C*, c.748-759+5del</u>

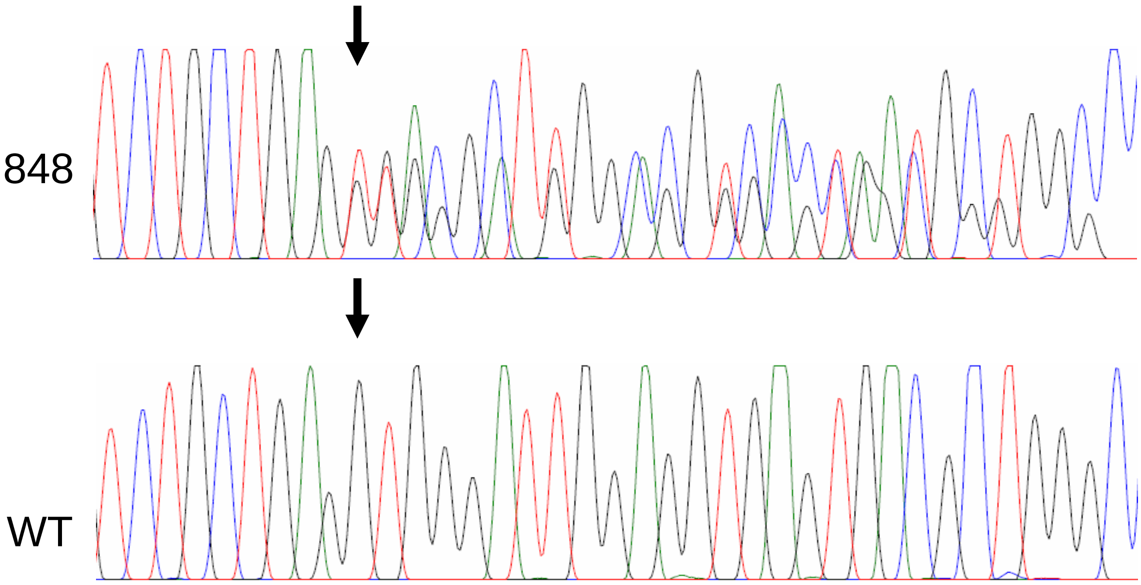

***CDH23*, IVS45-9G>A**

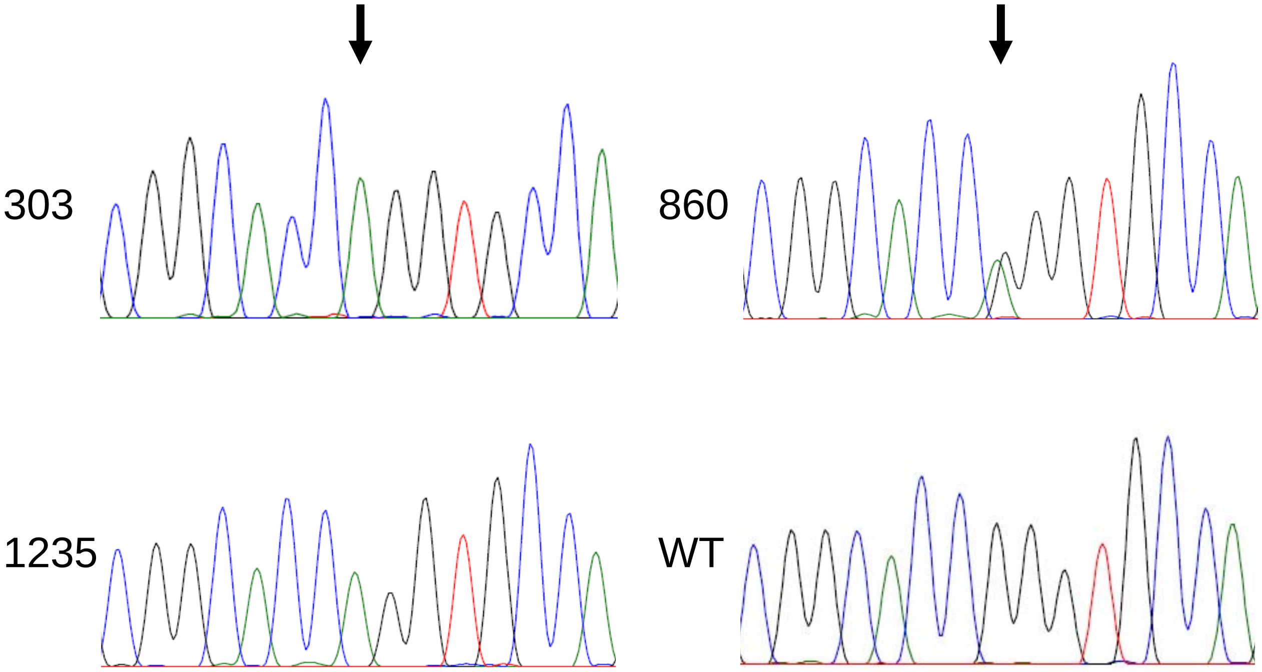

***CDH23*, c.2206C>T (p.R736X)**

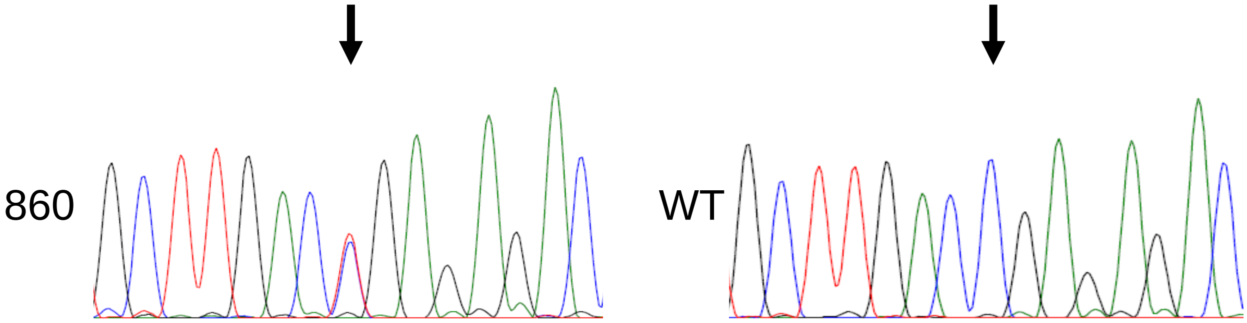

MYO7A, c.1370C>T (p.A457V)

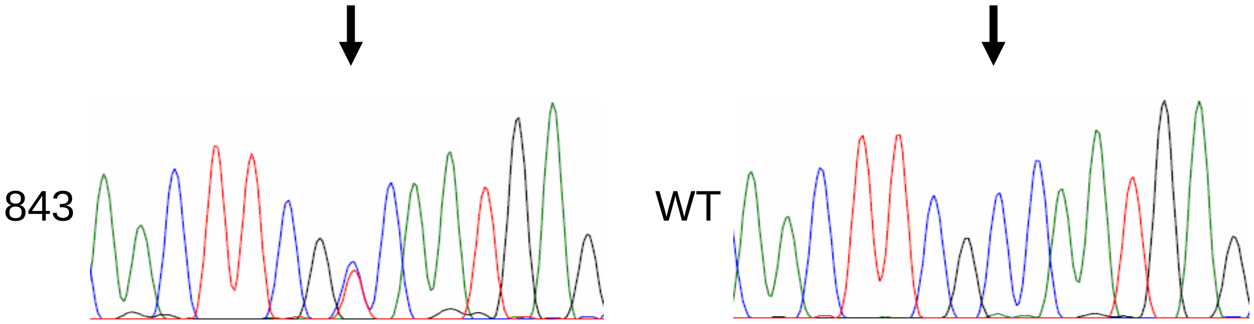

MYO7A, c.2443C>T (p.Q815X)

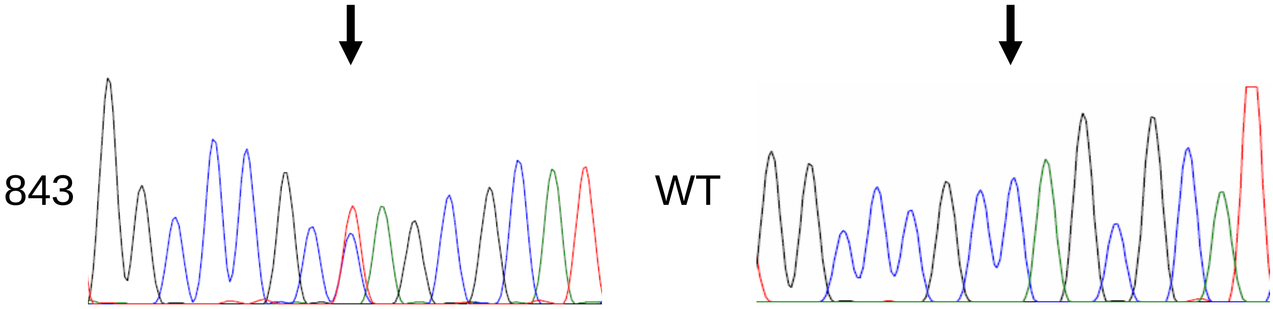

USH3A, c.368C>A (p.A123D)

1886

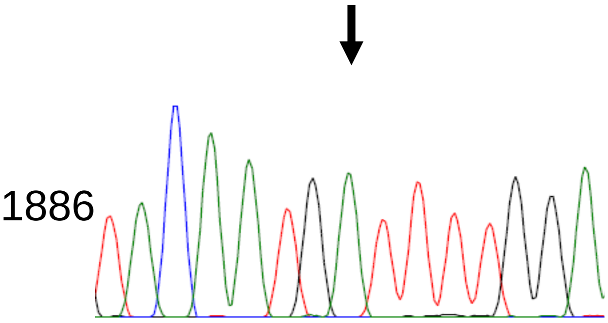

WT

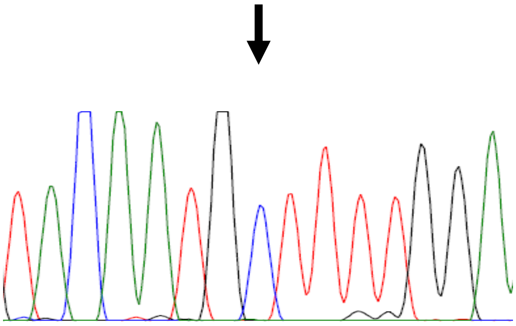

c.216G>A (*USH1C*):
Genotyping of 227 French Canadian healthy control individuals

PCR products (625 bp) were digested with *Dra*III. One *Dra*III site is present in the wildtype, resulting in fragments of 230 and 395 bp. Presence of c.216G>A results in loss of the *Dra*III site.

Controls for c.216G>A (*USH1C*, exon 3) by restriction digest with *Dra*III

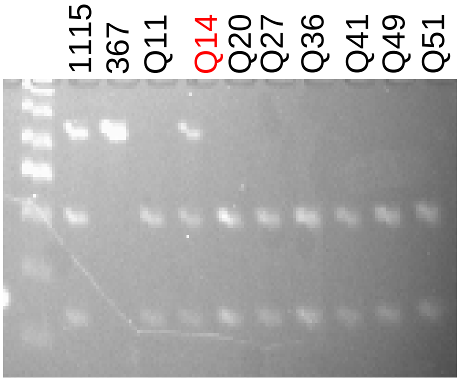

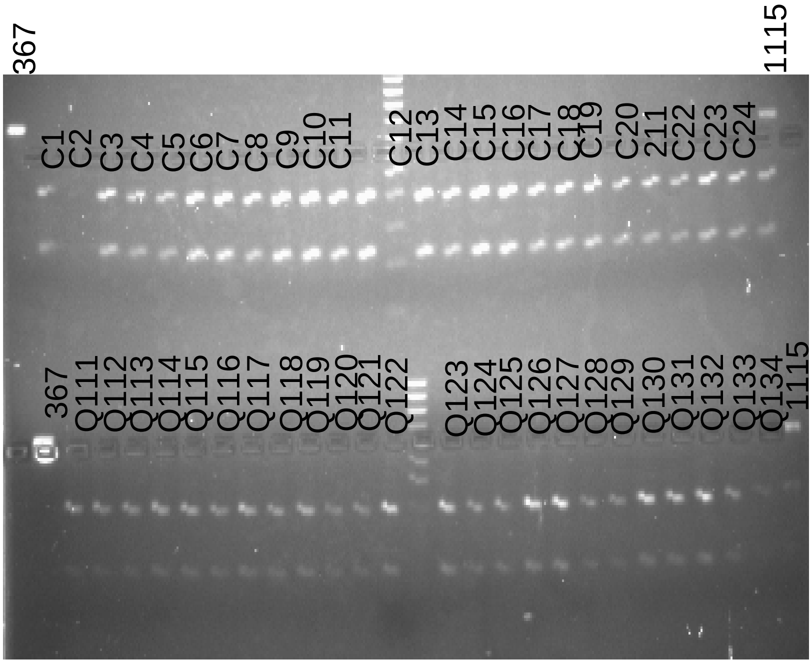

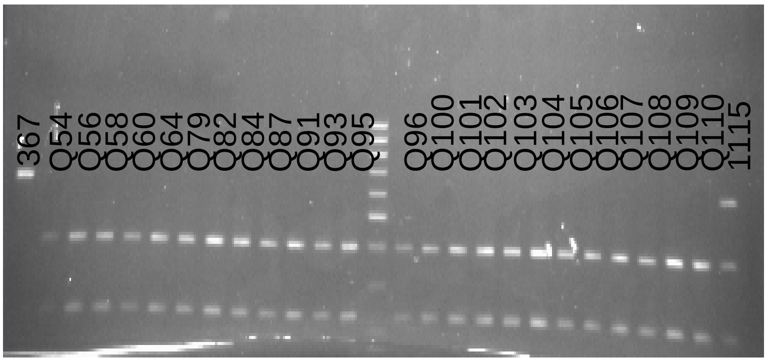

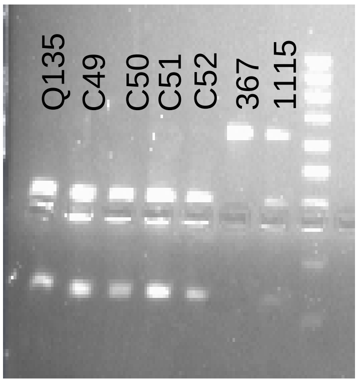

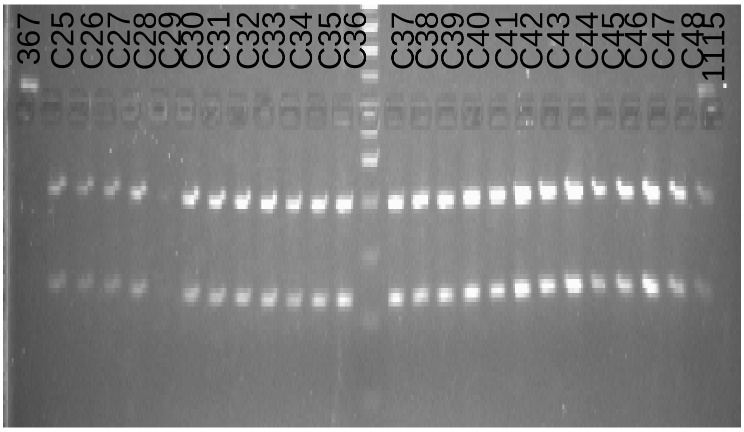

PCR product: 625 bp

367 = c.216G>A (homozygous)
1115 = c.216G>A (heterozygous)
Q14 = healthy carrier

→

WT = 1x *Dra*III restriction site
230+395 bp

Mutant = 625 bp

Controls for c.216G>A (*USH1C*, exon 3) by restriction digest with *Dra*III

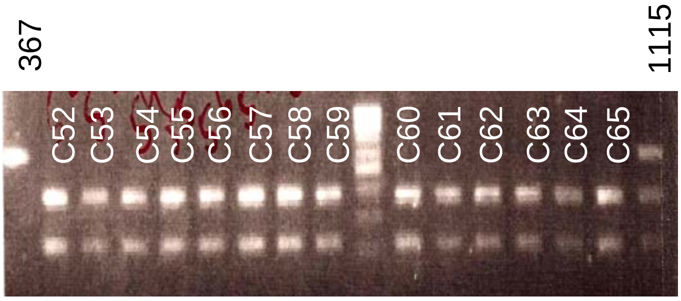

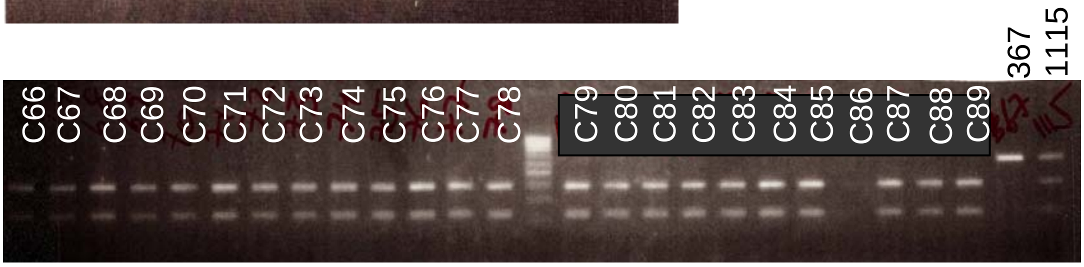

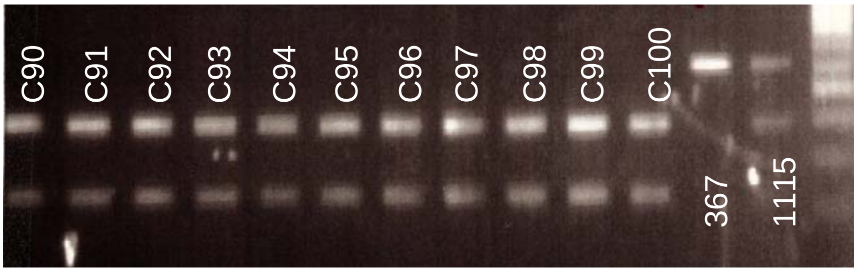

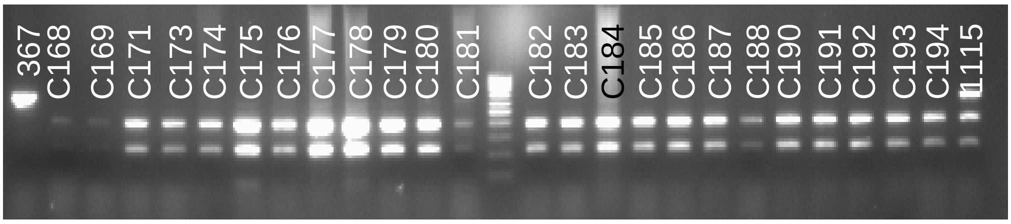

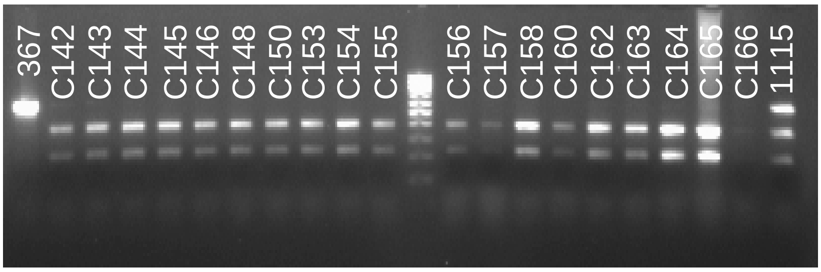

<u>Controls for c.216G>A (*USH1C*, exon 3) by restriction digest with *Dra*III</u>

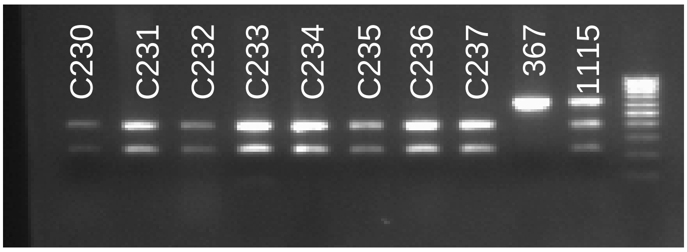

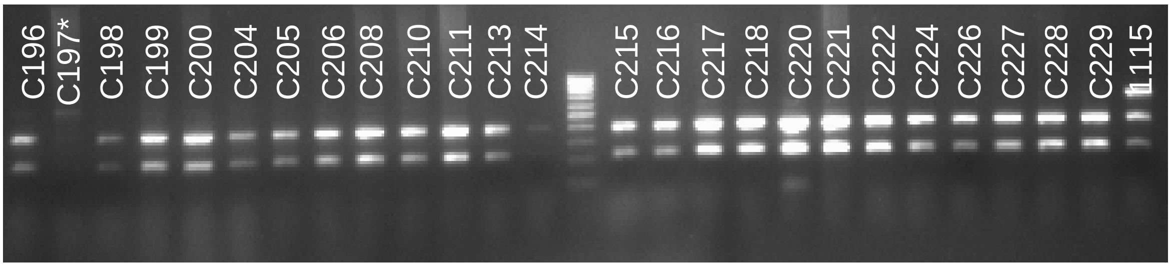

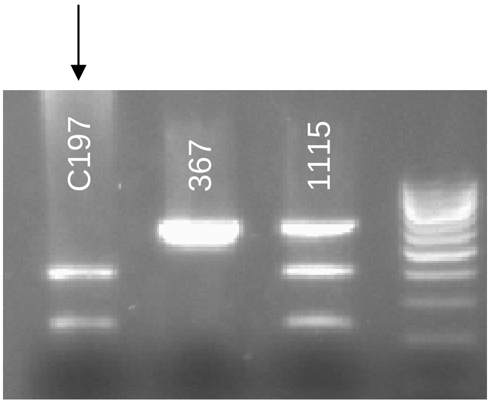

*digestion of control C197 was repeated and shown to be homozygous for c.216G (wildtype)

<u>Σ227 controls</u>

(Q2, Q133,Q134, C29, C66, C67, C86, C166 and C214 not counted (weak signal))

c.238-239insC (*USH1C*):
Genotyping of 100 French Canadian healthy control individuals

- fragment length analysis of exon 3 PCR products (GeneScan) -

***USH1C*, controls for c.238-239insC by GeneScan**

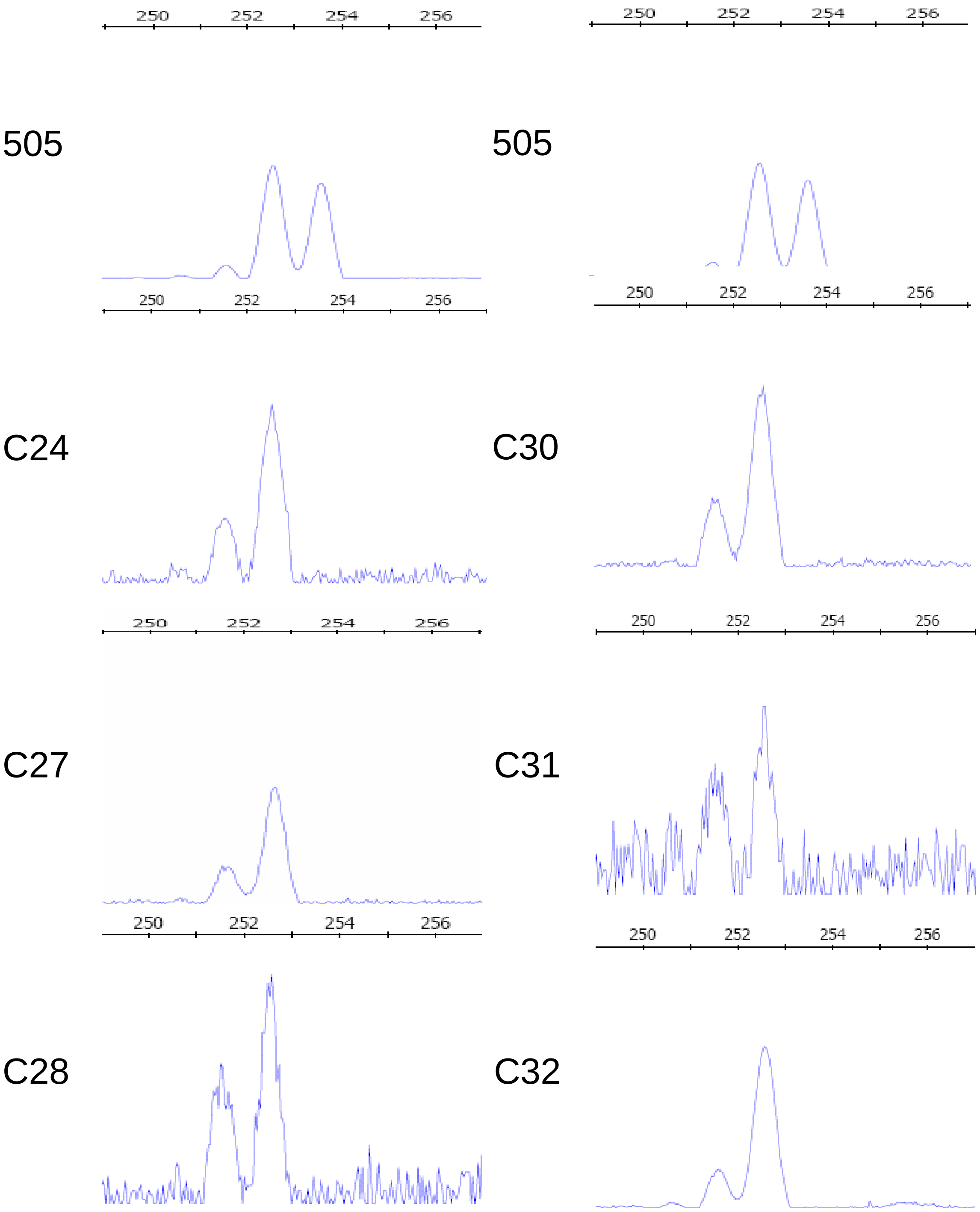

505: patient heterozygous for c.238-239insC (> two peaks)

<u>*USH1C*, controls for c.238-239insC by GeneScan</u>

505

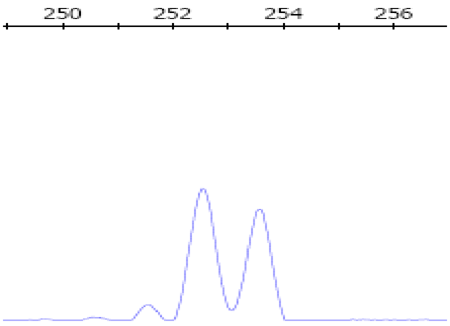

505

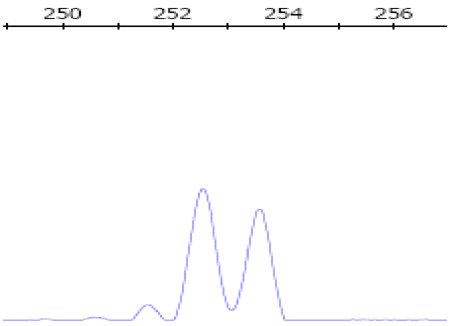

C33

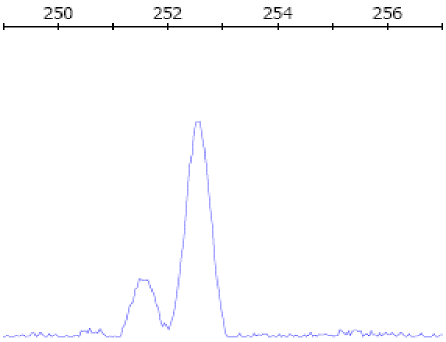

C36

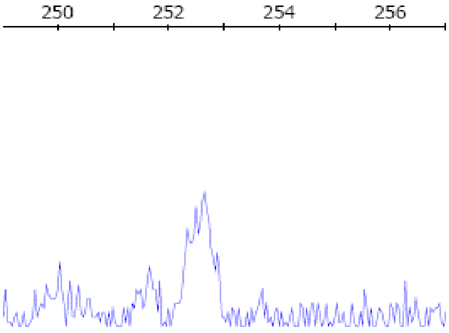

C34

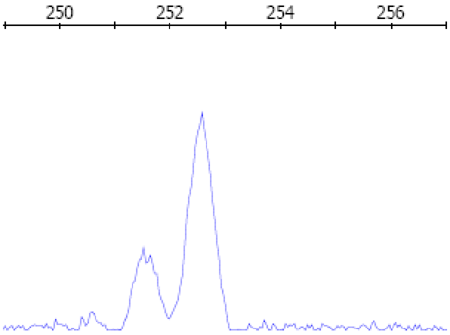

C37

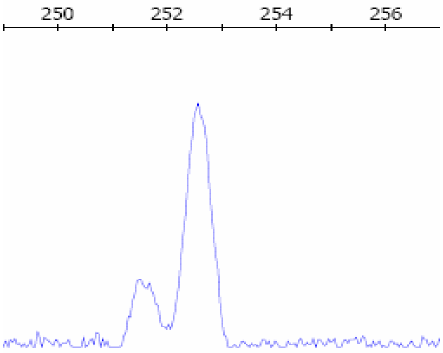

C35

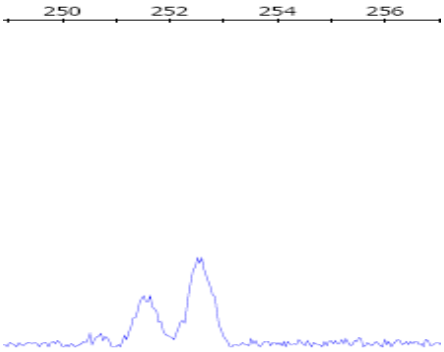

C38

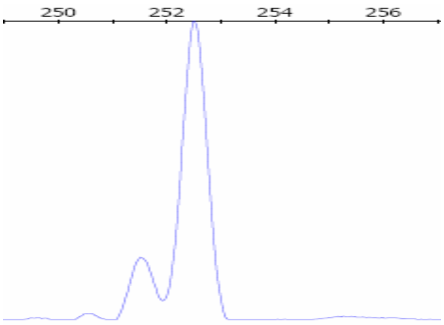

***USH1C*, controls for c.238-239insC by GeneScan**

505

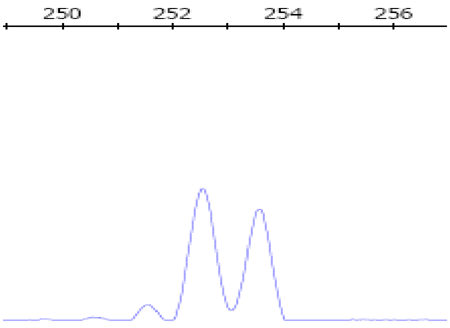

505

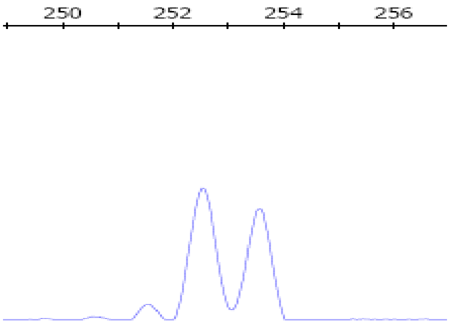

C42

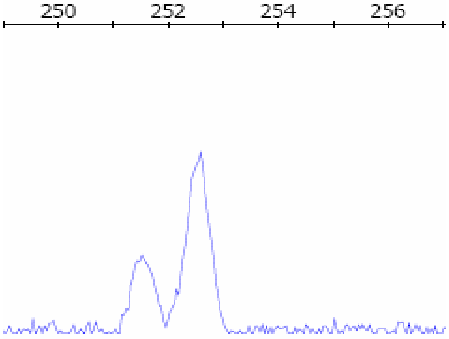

C45

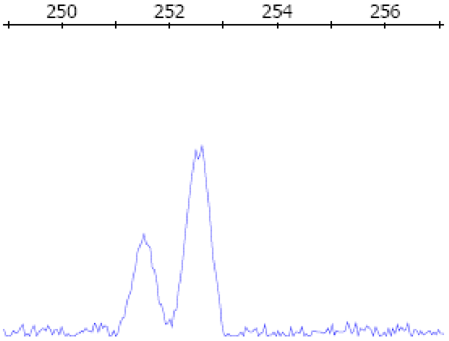

C43

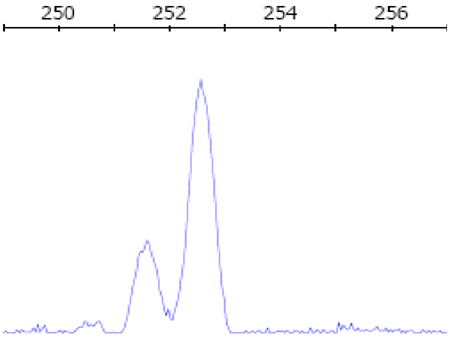

C46

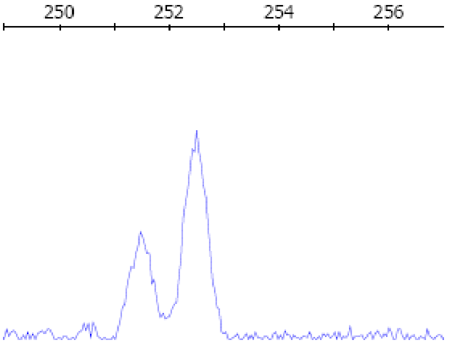

C44

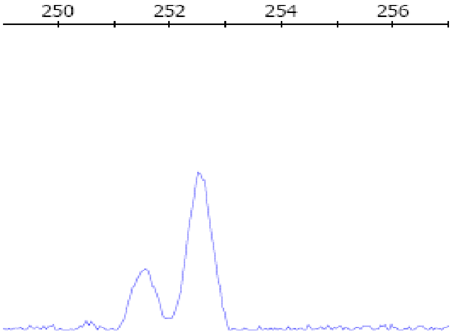

C48

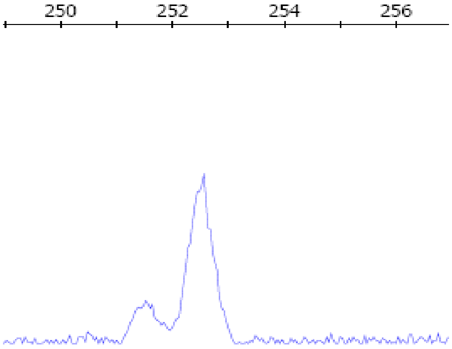

***USH1C*, controls for c.238-239insC by GeneScan**

505

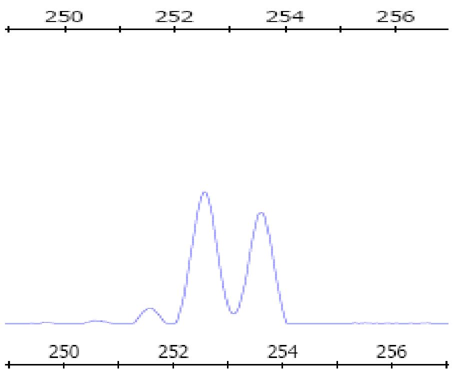

505

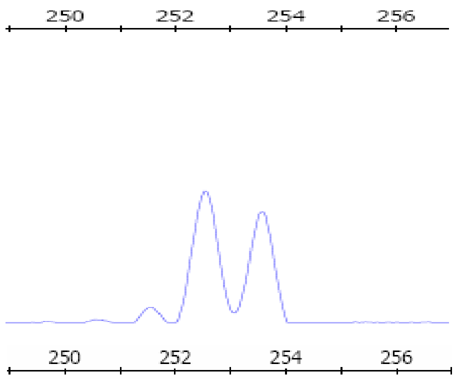

C49

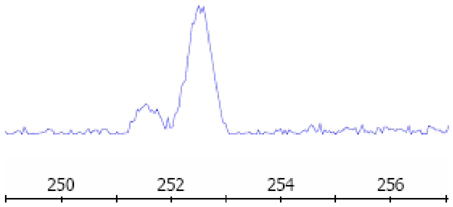

C52

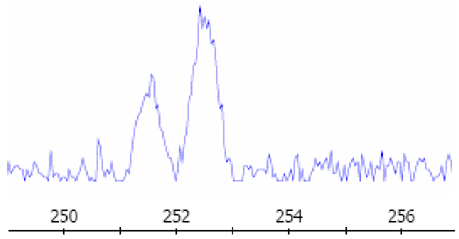

C50

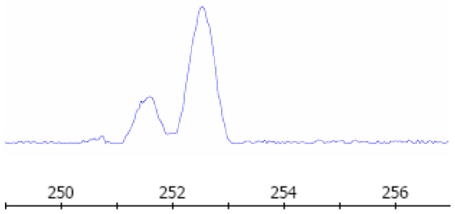

C53

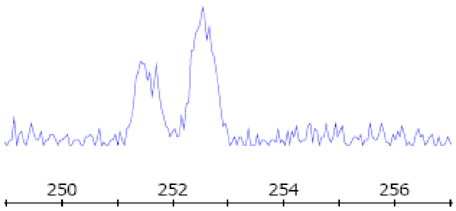

C51

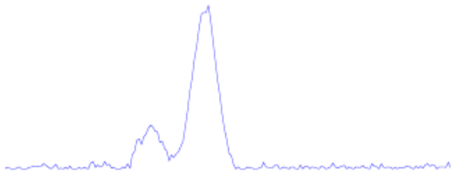

C54

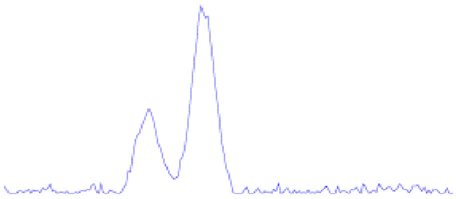

USH1C, controls for c.238-239insC by GeneScan

505

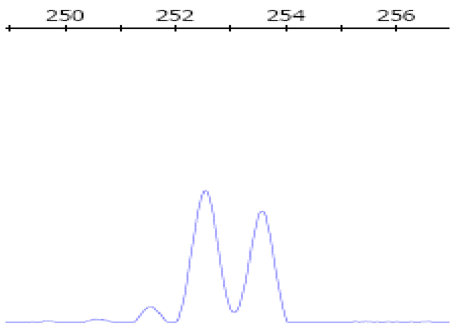

505

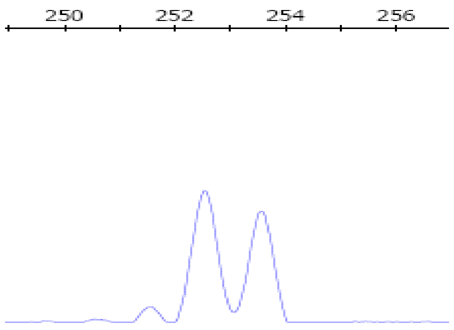

C55

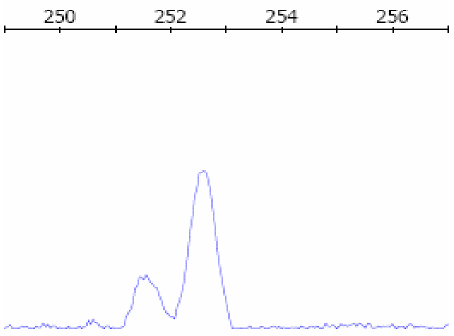

C58

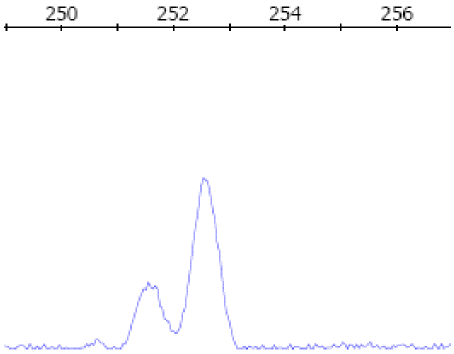

C56

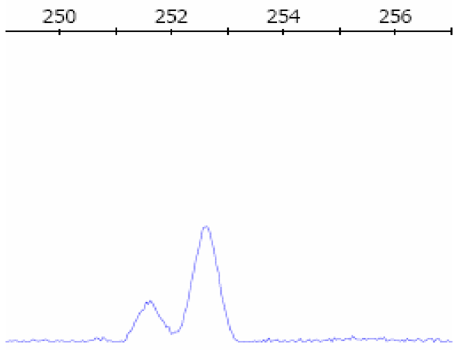

C59

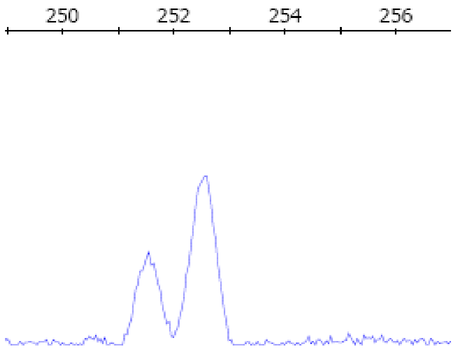

C57

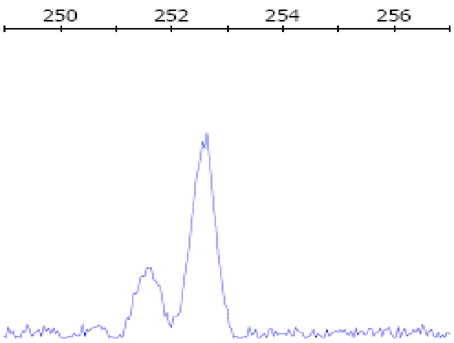

C60

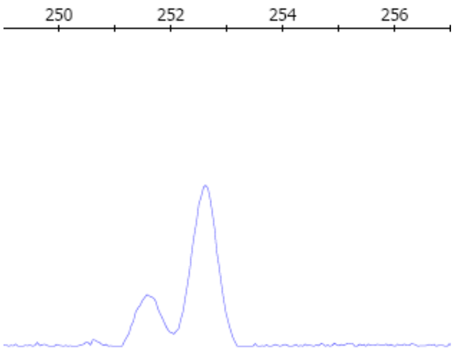

USH1C, controls for c.238-239insC by GeneScan

505

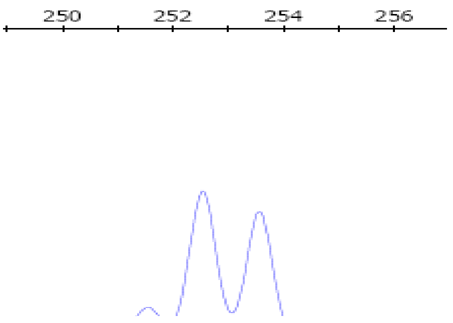

C61

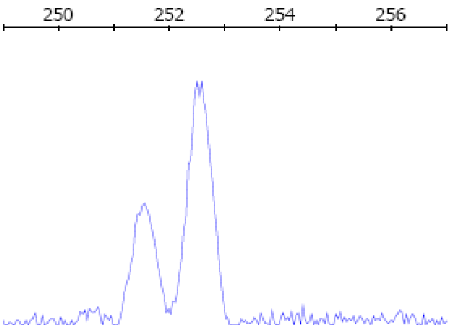

C62

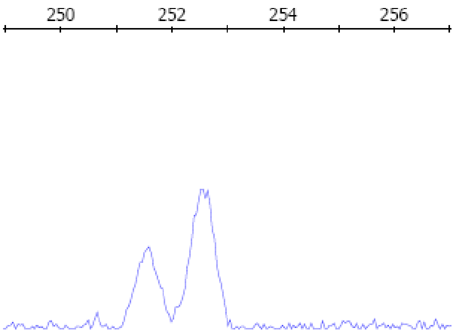

C63

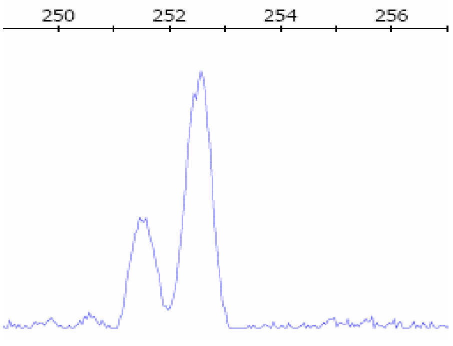

505

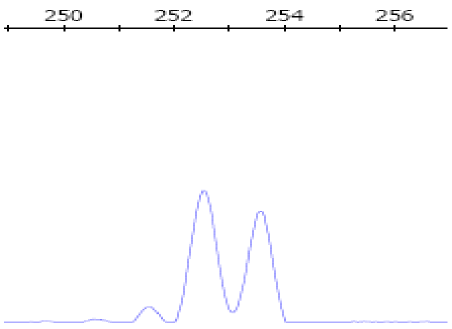

C64

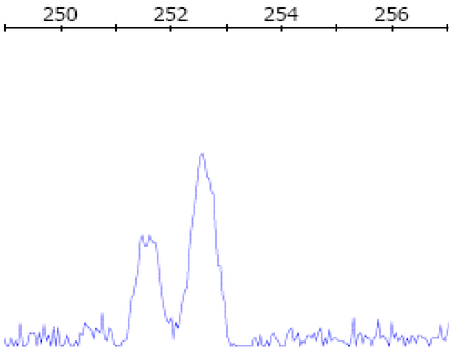

C65

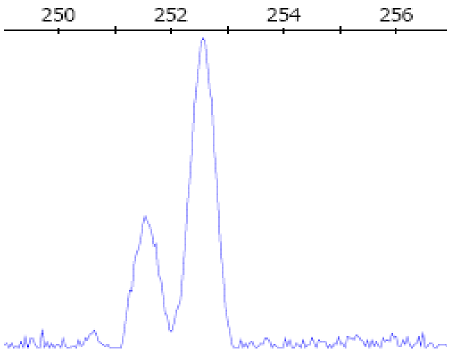

C66

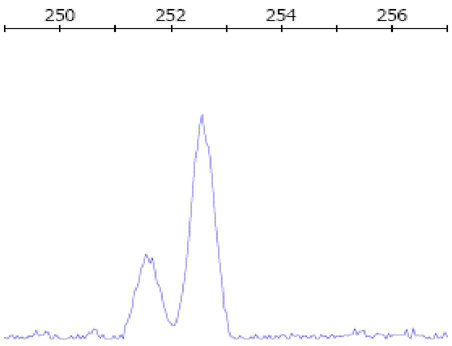

***USH1C*, controls for c.238-239insC by GeneScan**

505

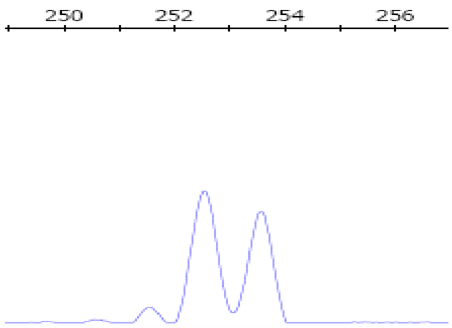

505

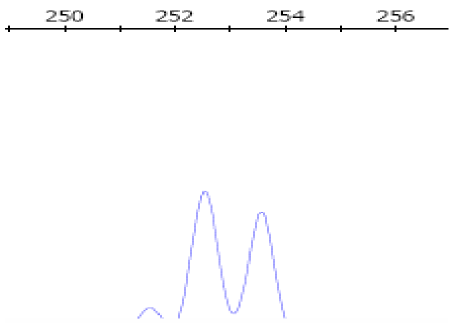

C67

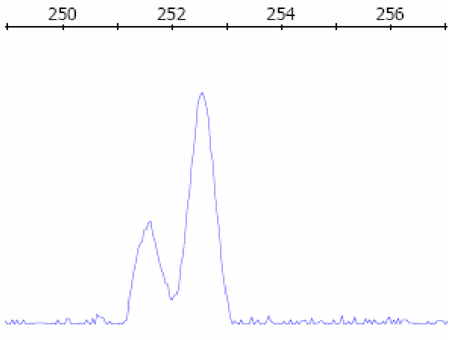

C70

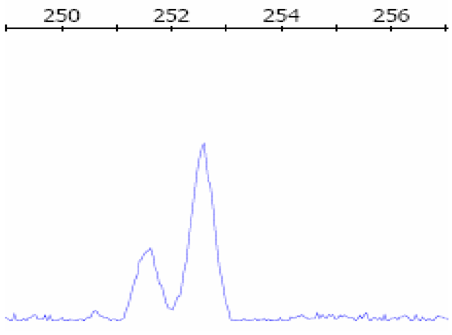

C68

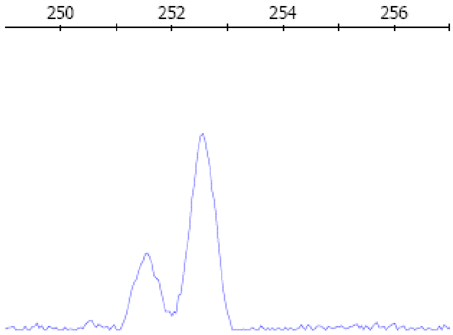

C71

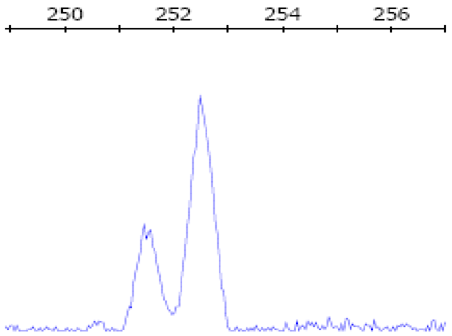

C69

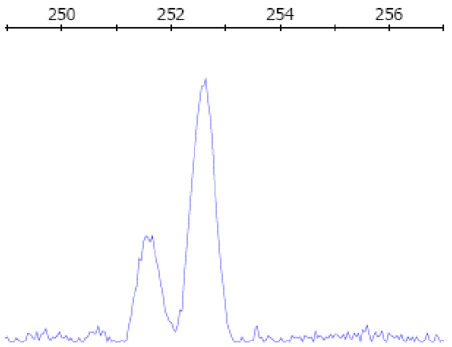

C72

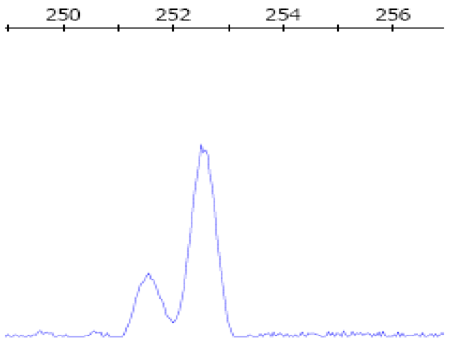

USH1C, controls for c.238-239insC by GeneScan

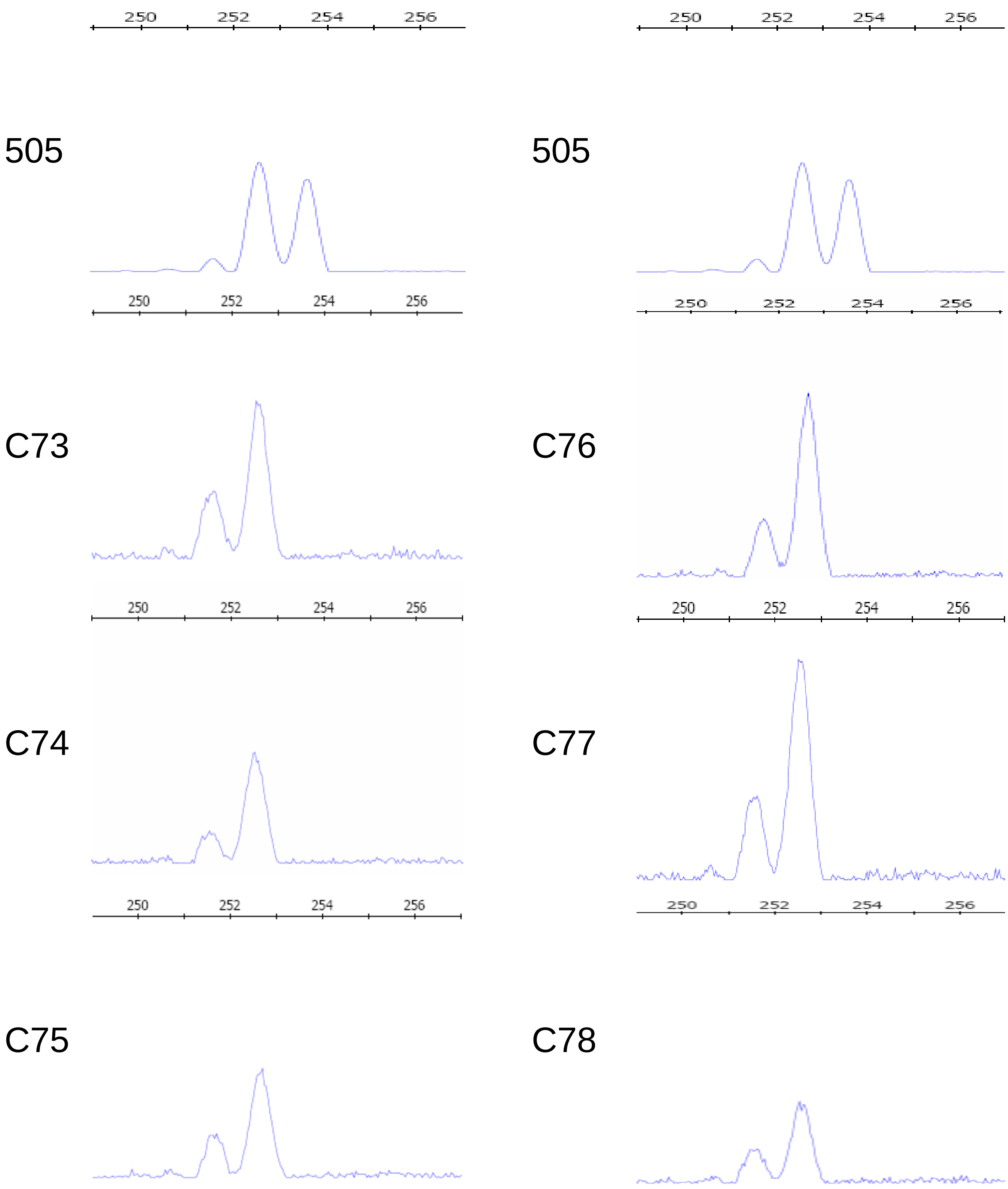

USH1C, controls for c.238-239insC by GeneScan

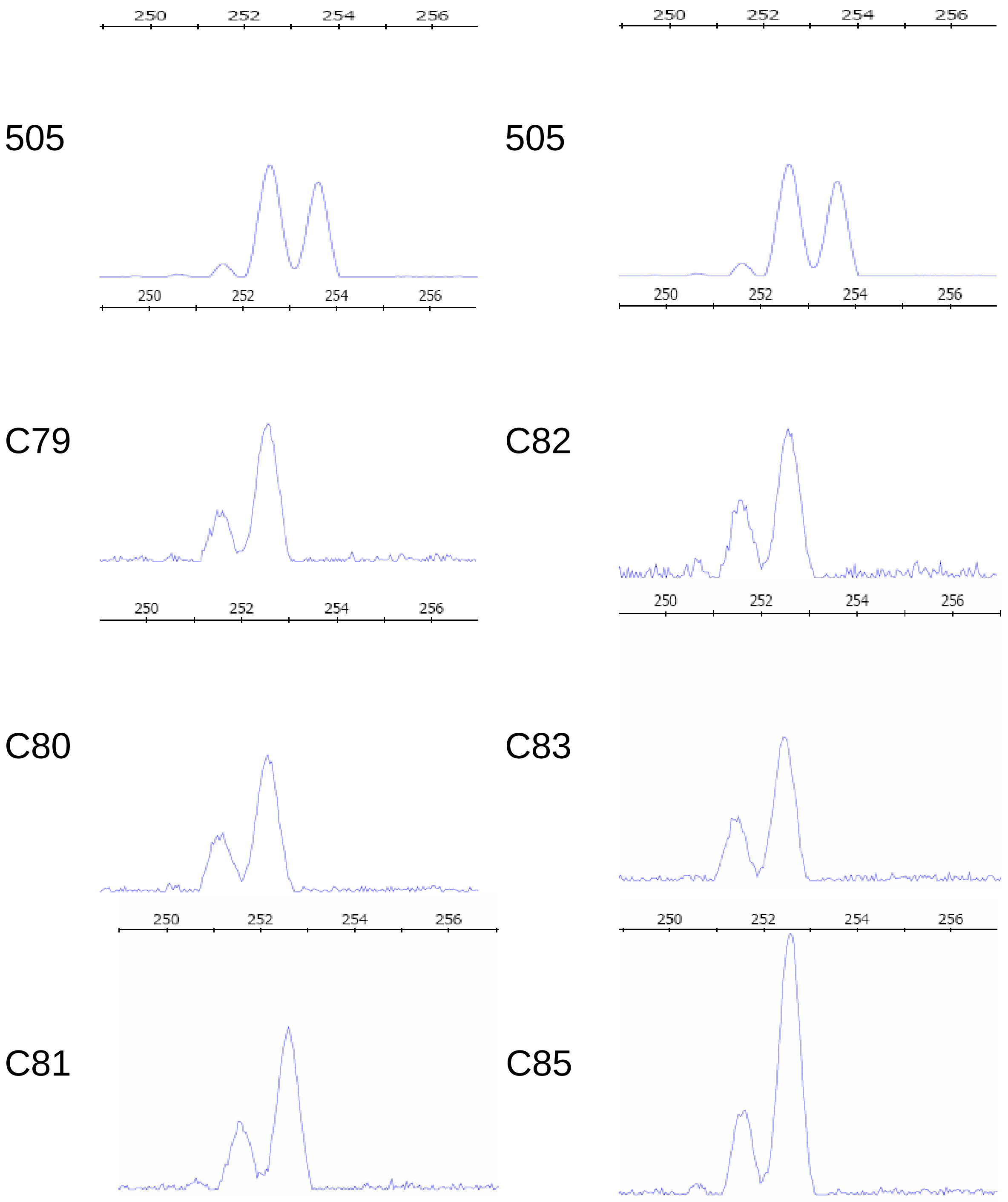

USH1C, controls for c.238-239insC by GeneScan

505

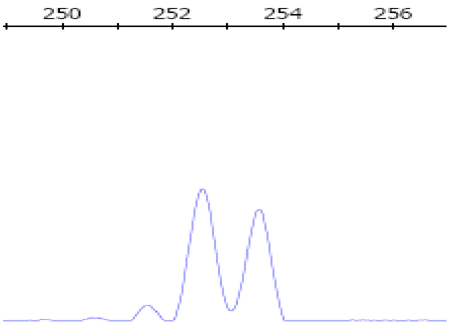

505

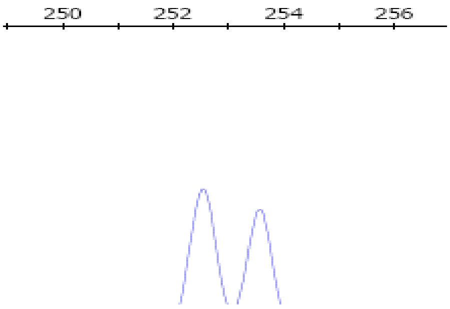

C86

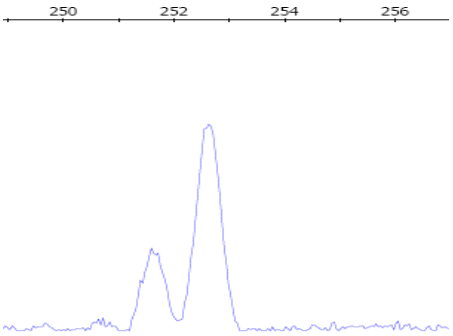

C89

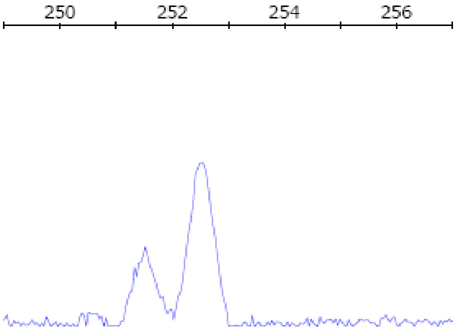

C87

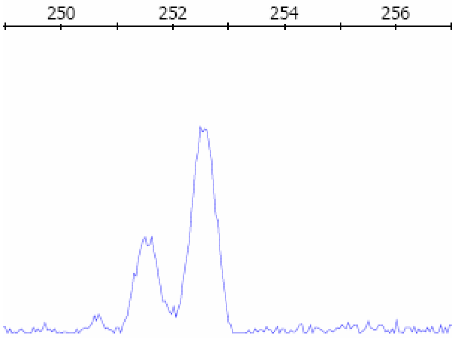

C91

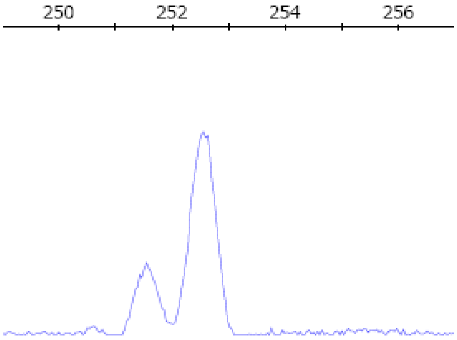

C88

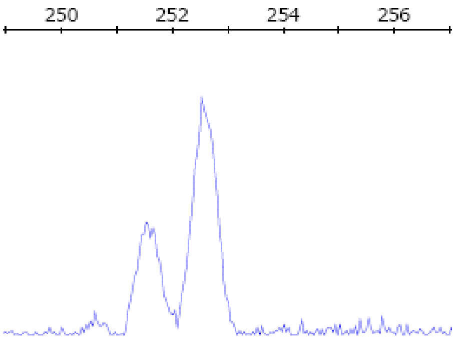

C92

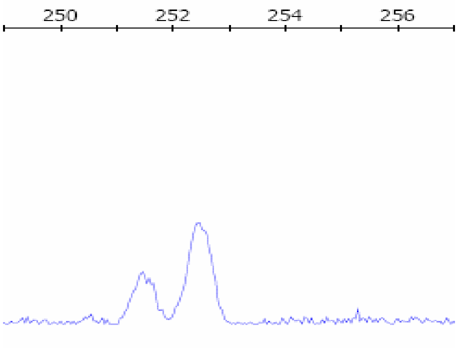

USH1C, controls for c.238-239insC by GeneScan

505

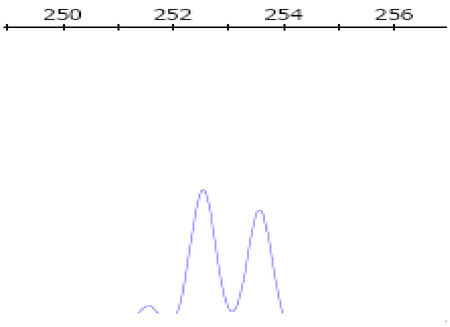

505

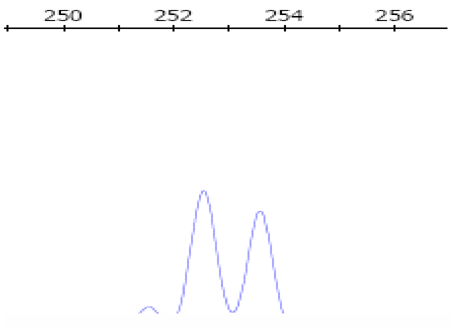

C93

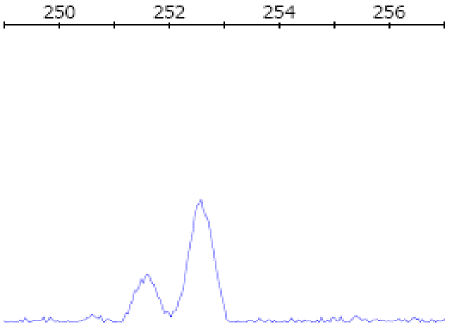

C96

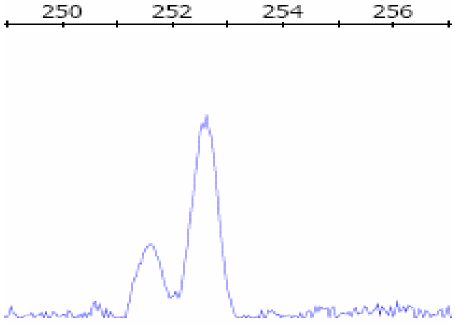

C94

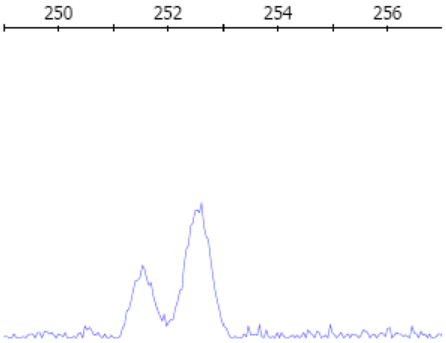

C97

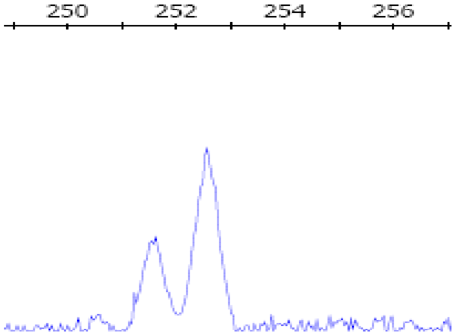

C95

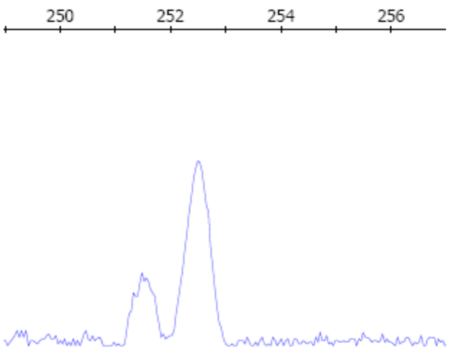

C98

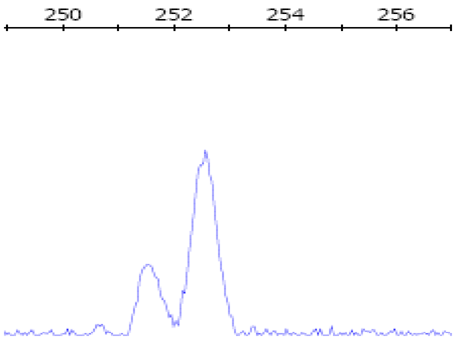

***USH1C*, controls for c.238-239insC by GeneScan**

505

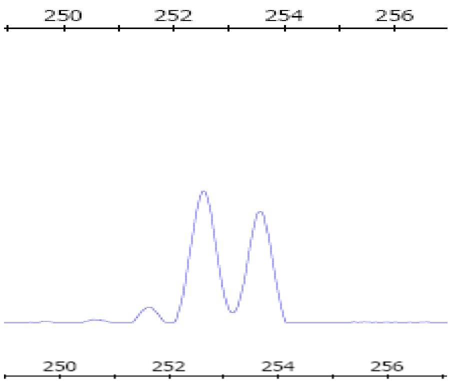

505

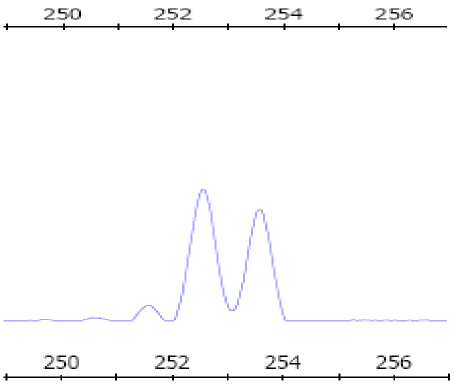

C99

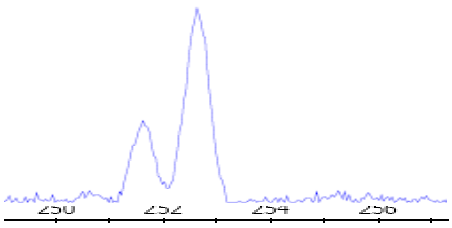

C102

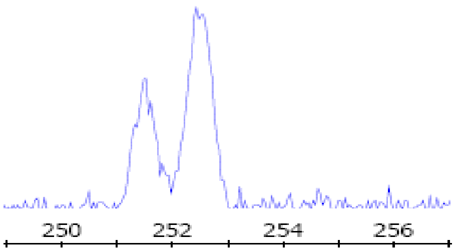

C100

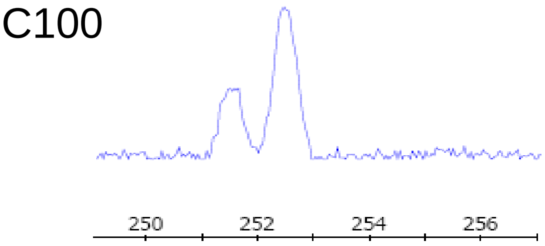

C103

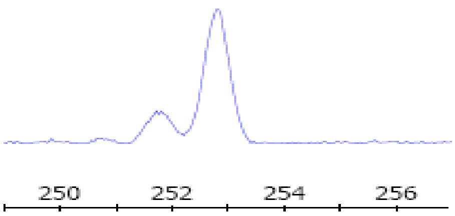

C101

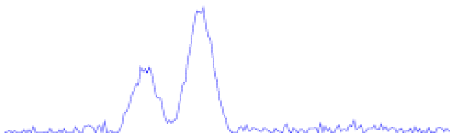

C104

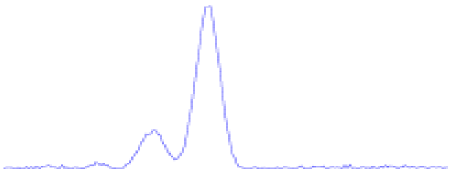

USH1C, controls for c.238-239insC by GeneScan

505

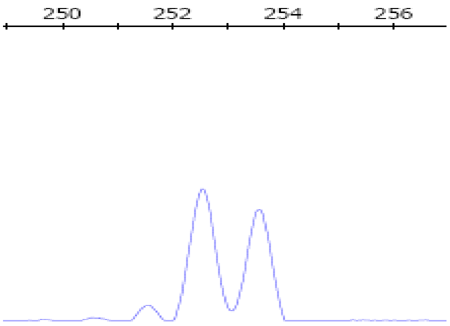

505

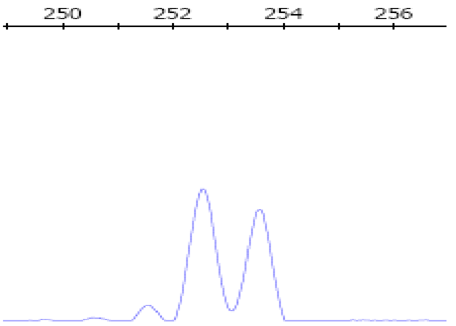

C105

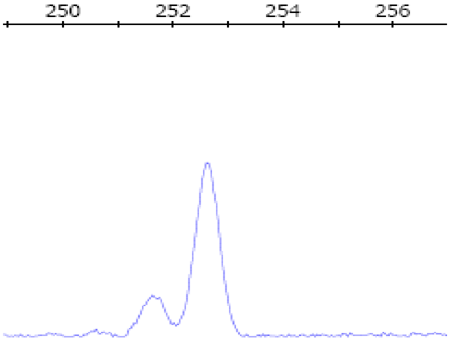

C108

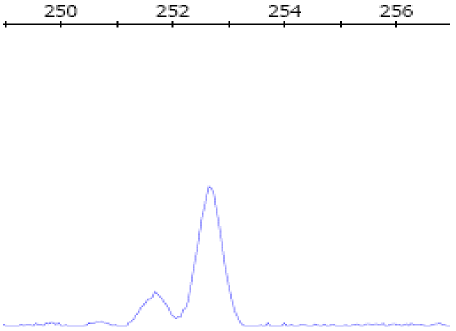

C106

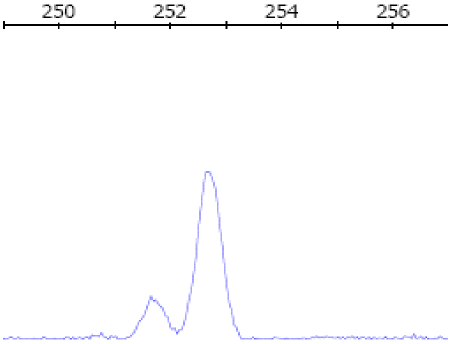

C109

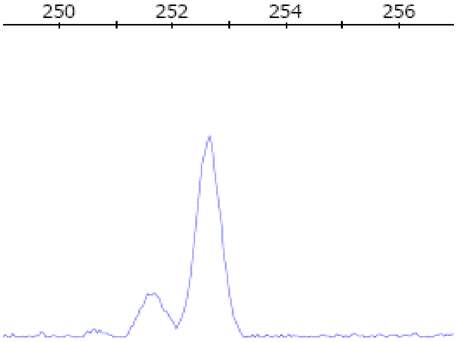

C107

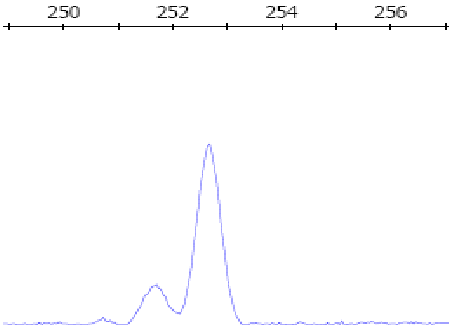

C110

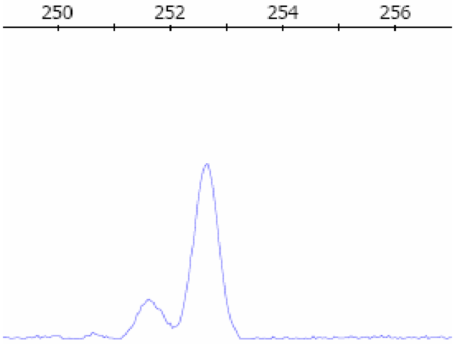

USH1C, controls for c.238-239insC by GeneScan

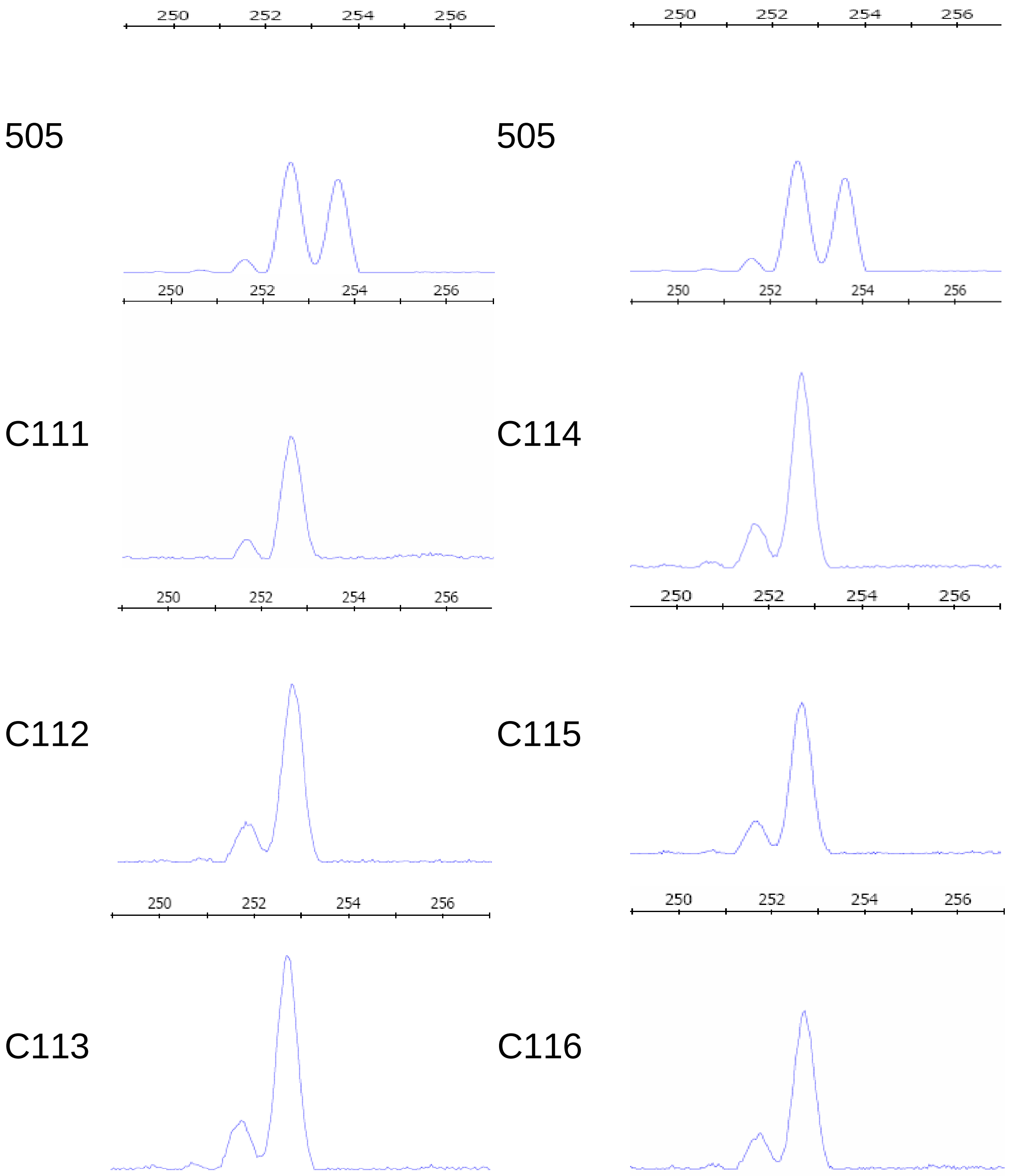

***USH1C*, controls for c.238-239insC by GeneScan**

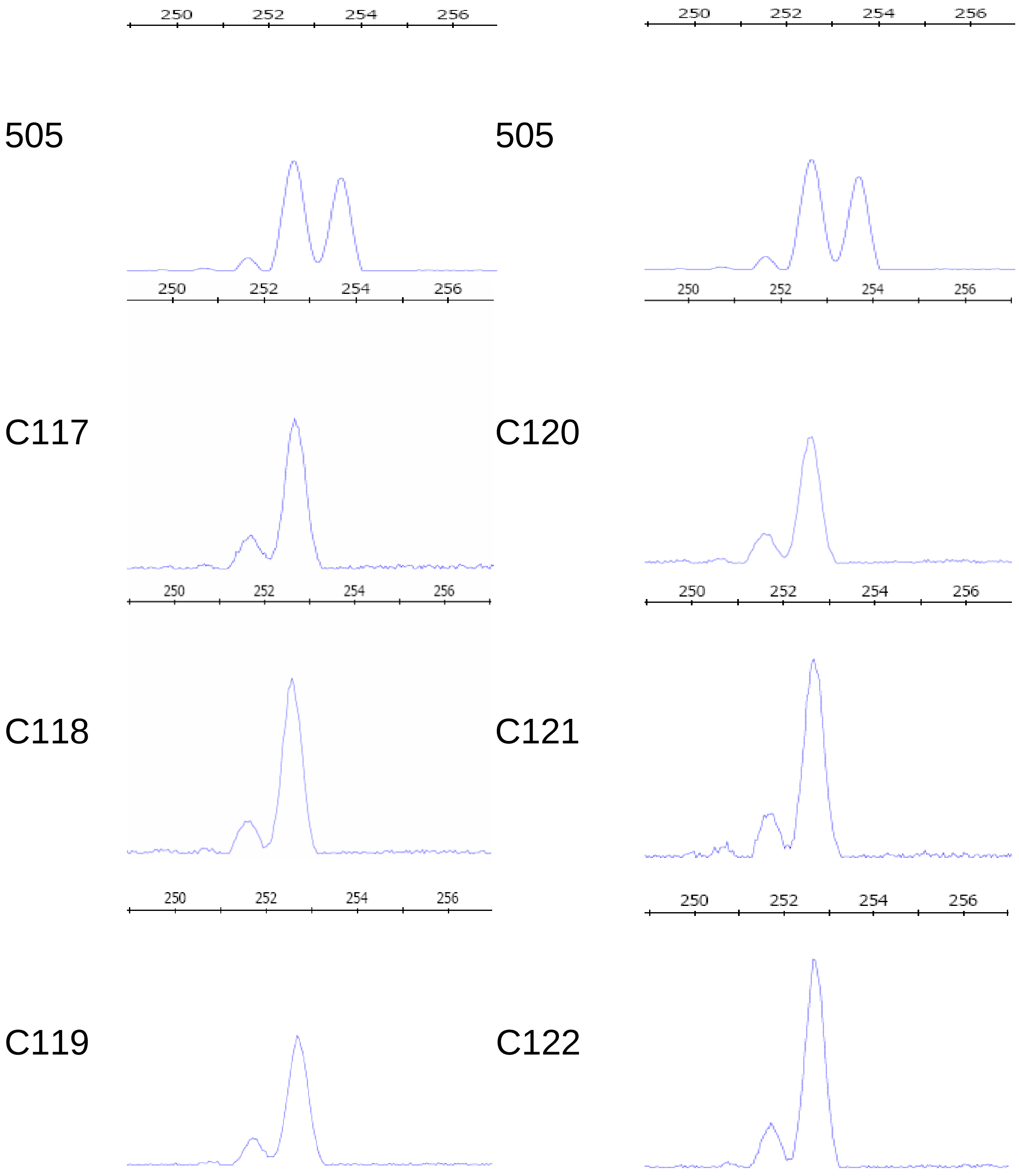

***USH1C*, controls for c.238-239insC by GeneScan**

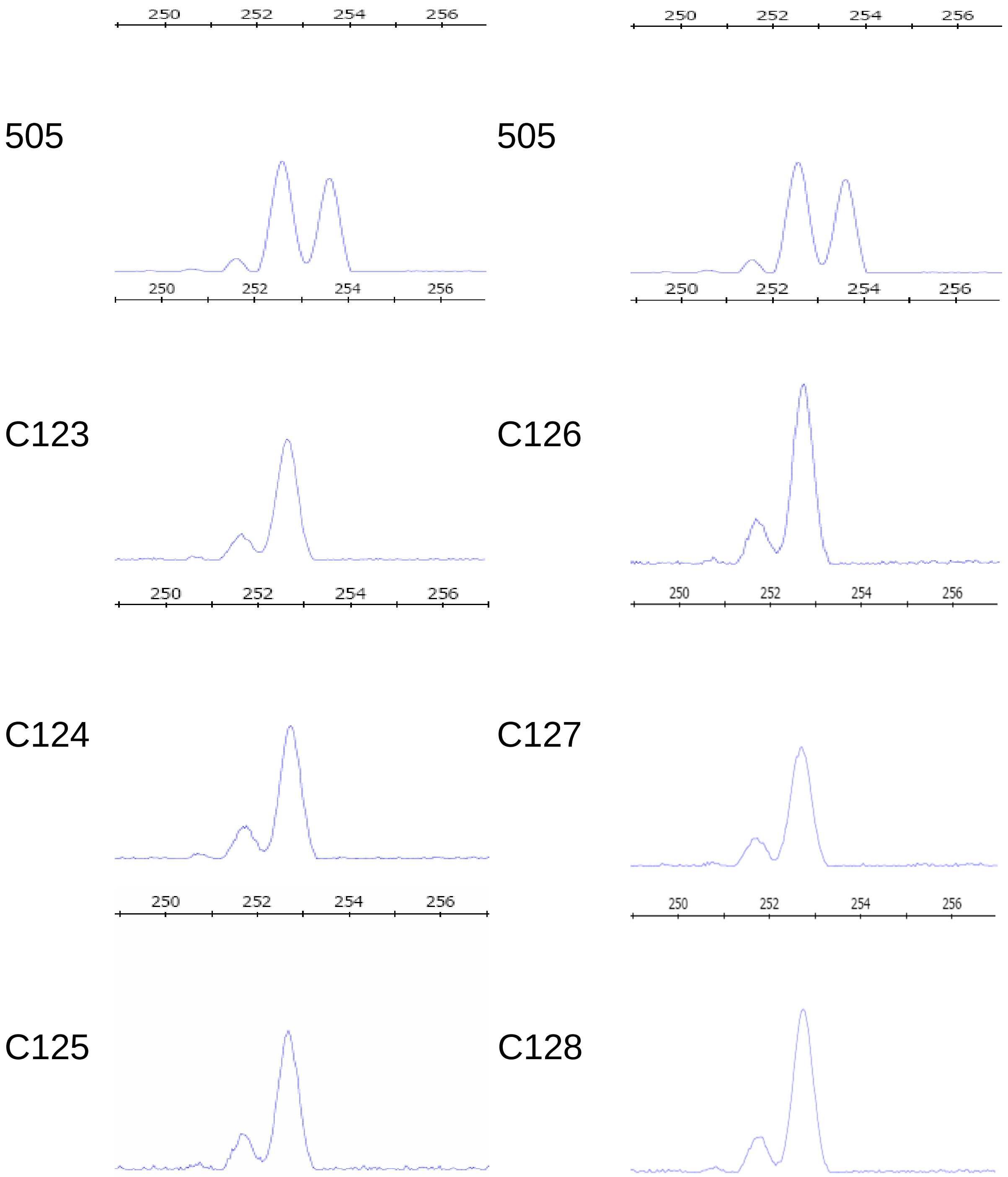

USH1C, controls for c.238-239insC by GeneScan

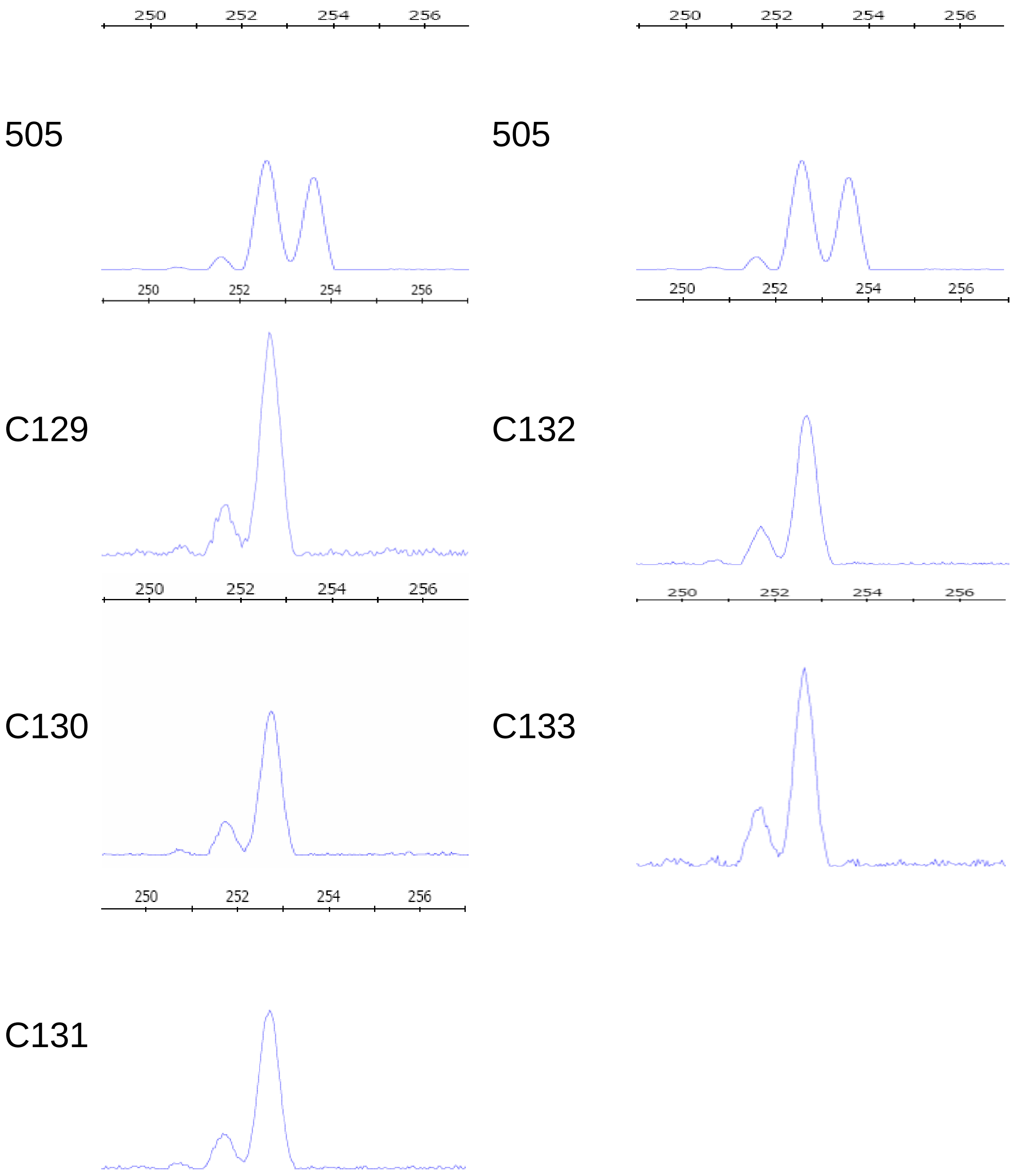

c.496+1G>T (*USH1C*):
Genotyping of 100 French Canadian healthy control individuals

PCR products (297 bp) were digested with *Esp3*I. One *Esp3*I site is present in the wildtype, resulting in fragments of 208 and 88 bp. Presence of c.496+1G>T results in loss of the *Esp3*I site.

<u>Controls for c.496+1G>T (*USH1C*, exon 5)</u>
<u>by restriction digestion with *Esp3*I</u>

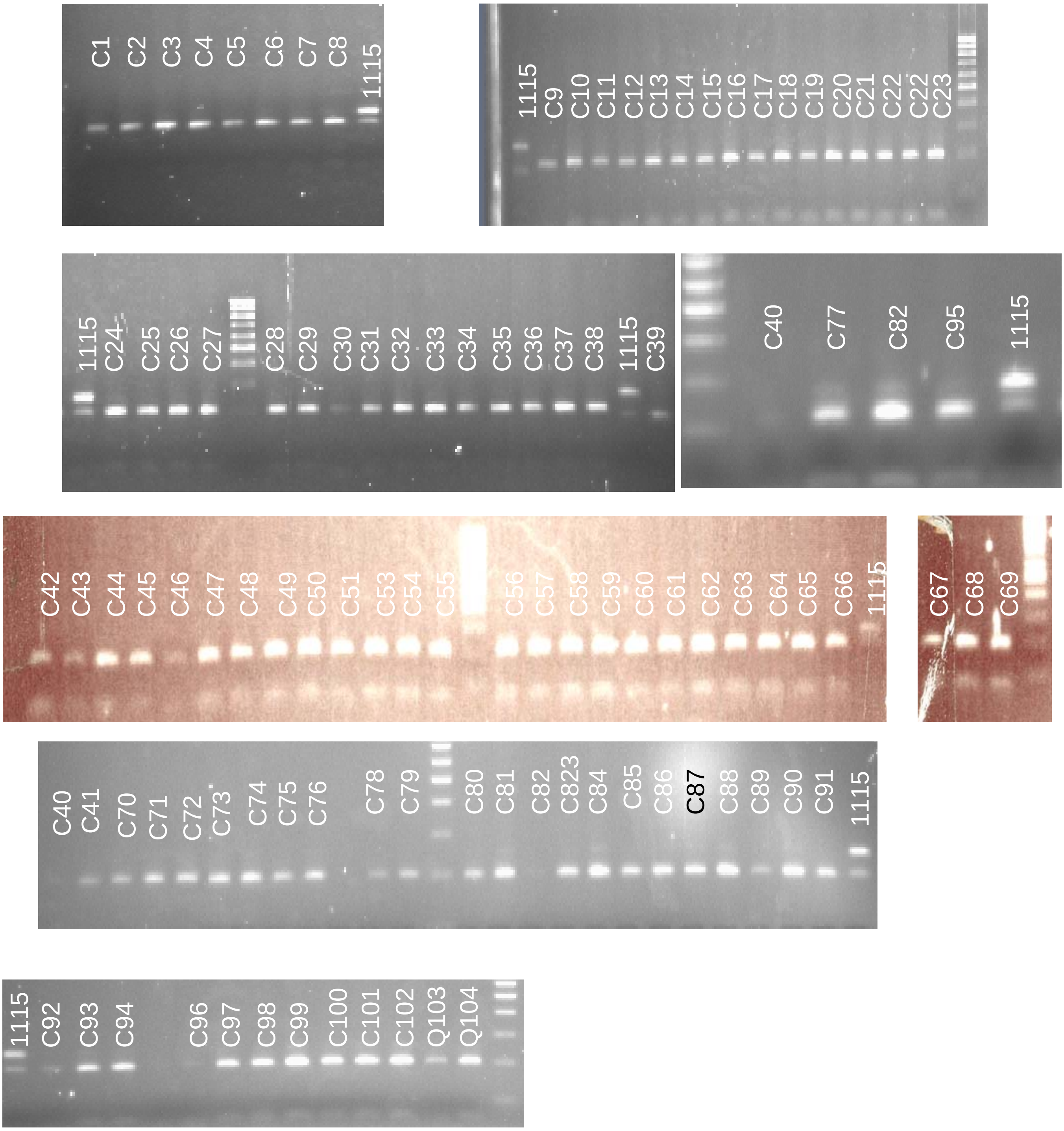

1115: patient heterozygous for c.496+1G>T

p.R155X (*USH1C*):
Genotyping of 100 French Canadian healthy control individuals

PCR products (297 bp) were digested with *Bsp119*I. One *Bsp119*I site is present in the wildtype, resulting in fragments of 174 and 123 bp. Presence of c.463C>T results in loss of the *Bsp119*I site.

<u>Controls for c.463C>T/p.R155X (*USH1C*, exon 5) by restriction digest with *Bsp119*I</u>

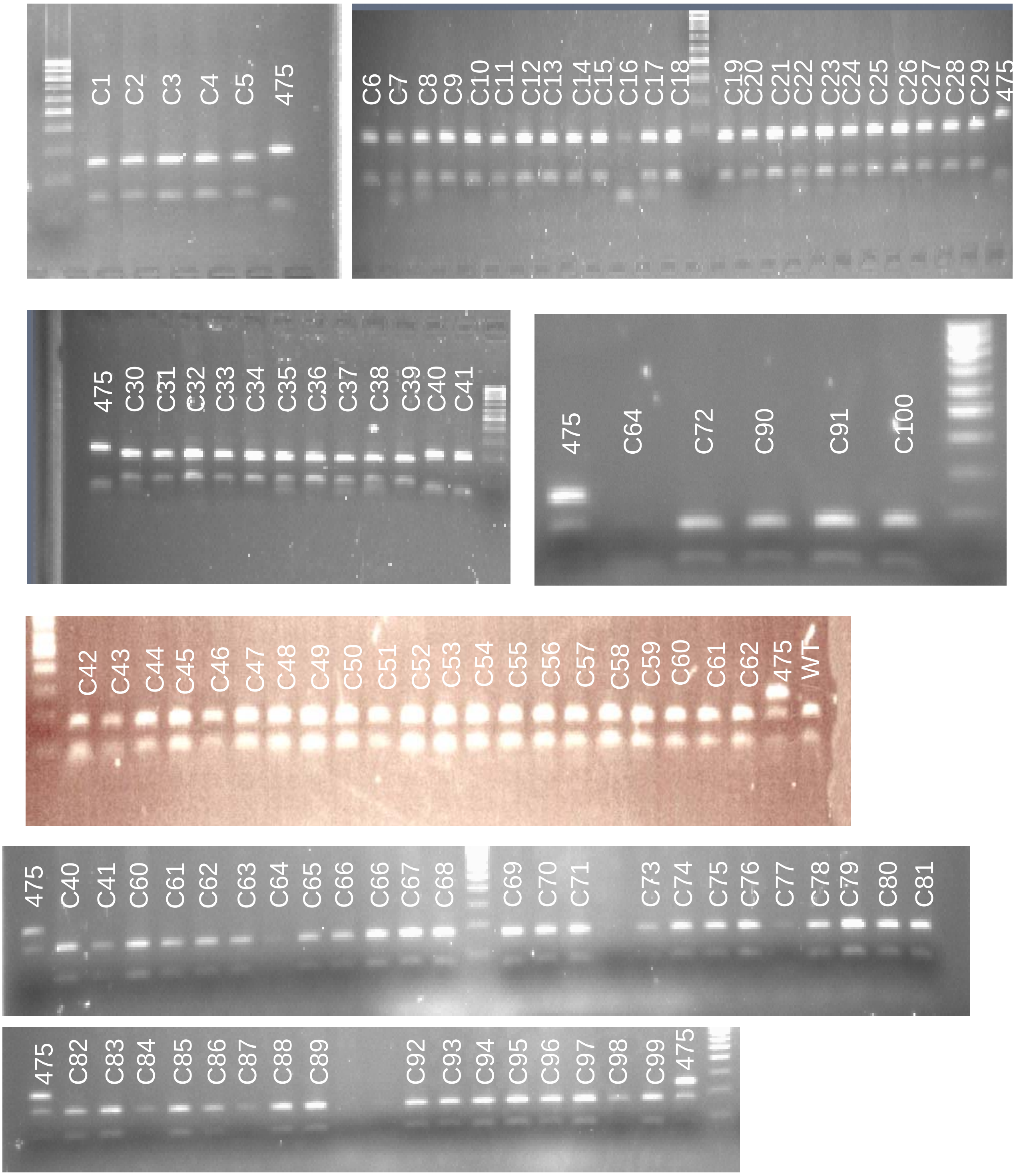

475: patient heterozygous for p.R155X

c.748-759+5del (*USH1C*):
Genotyping of 100 French Canadian healthy control individuals

PCR products (225 bp) were run on a 3% agarose gel. The PCR product heterozygous for c.748-759+5del shows a second band of 208 bp.

Controls for c.748_759+5del (*USH1C,* exon 9) by electrophoresis on a 3% agarose gel

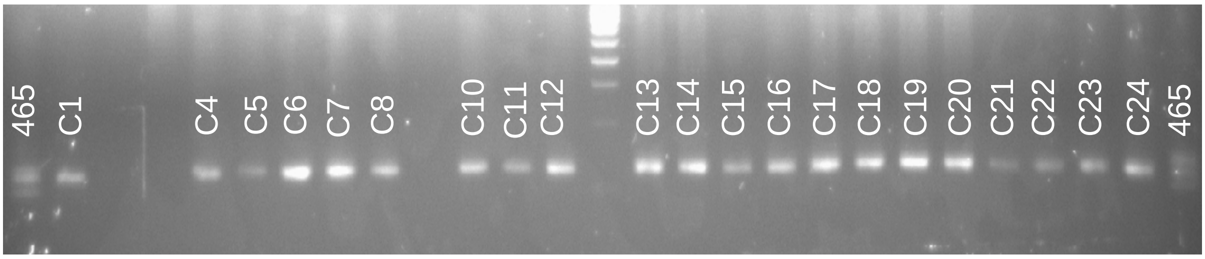

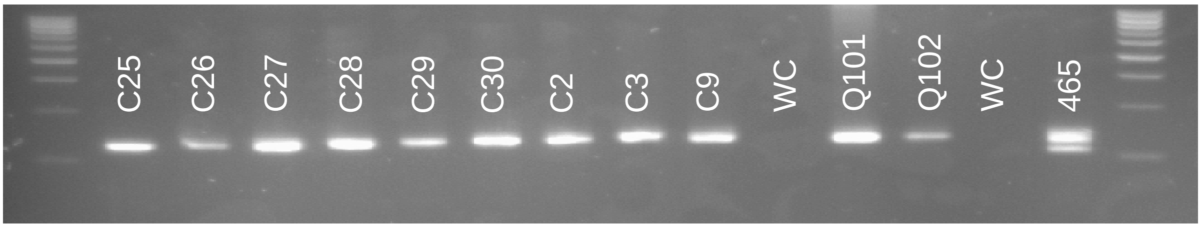

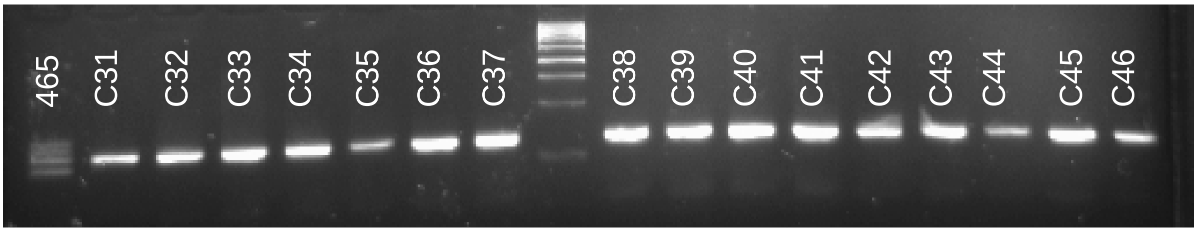

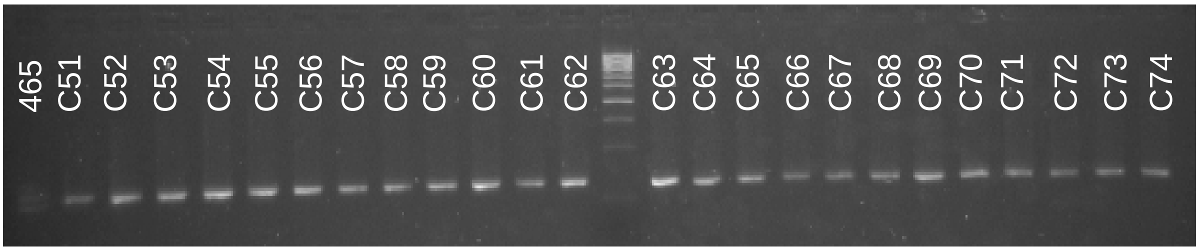

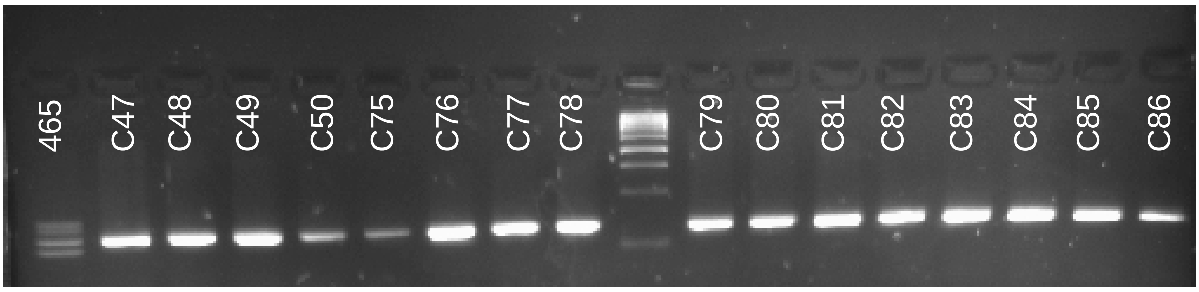

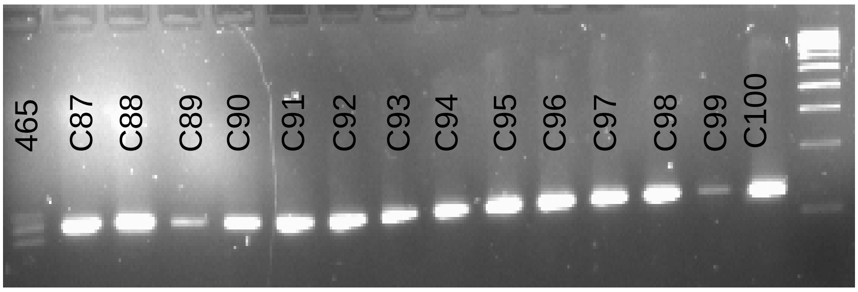

WC = water control
465 = heterozygous for c.748_759+5del

IVS45-9G>A (*CDH23*):
Genotyping of 100 French Canadian healthy control individuals

- by direct sequencing of PCR products -

<u>Controls for *CDH23*, IVS45-9G>A</u>

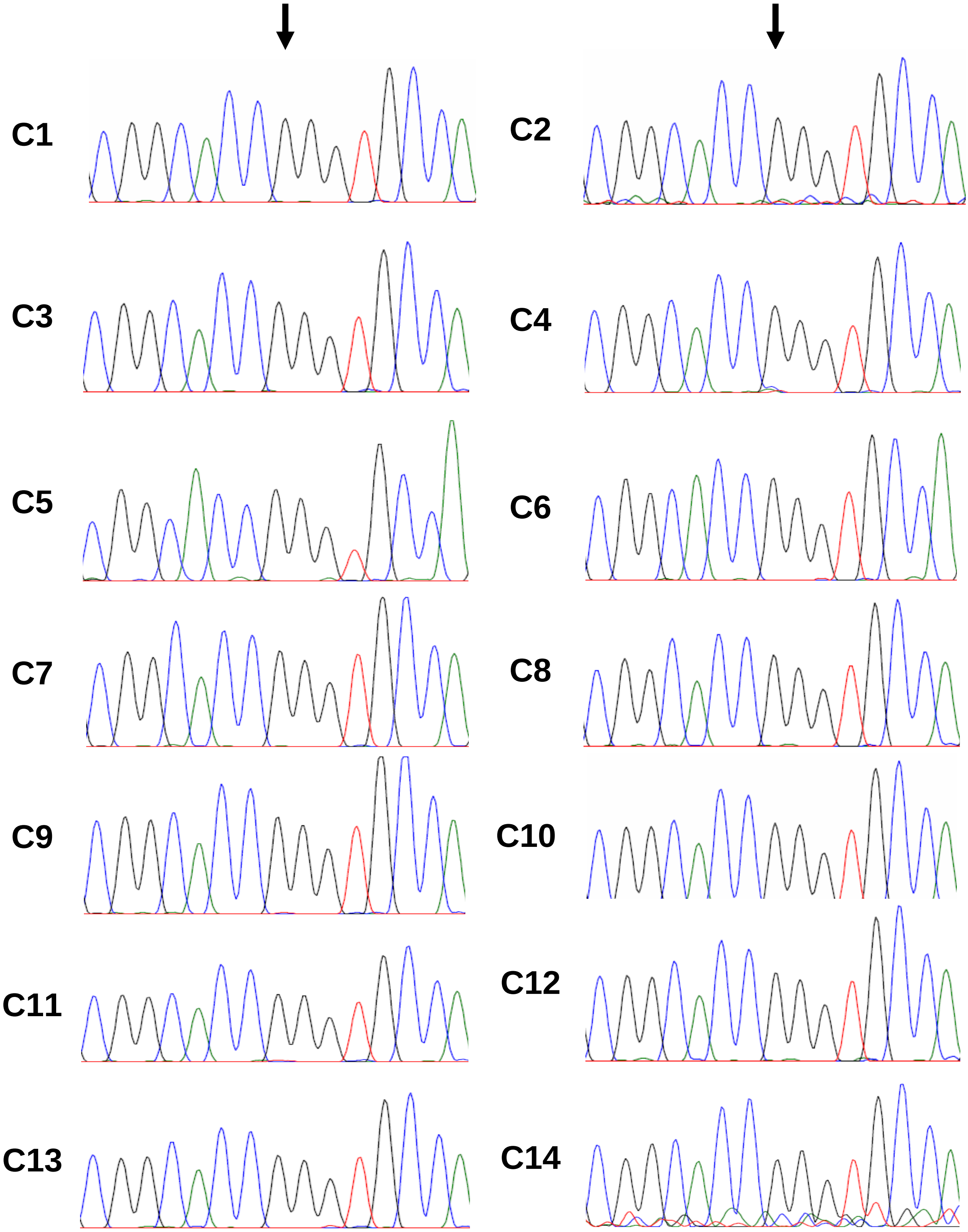

Controls for *CDH23*, IVS45-9G>A

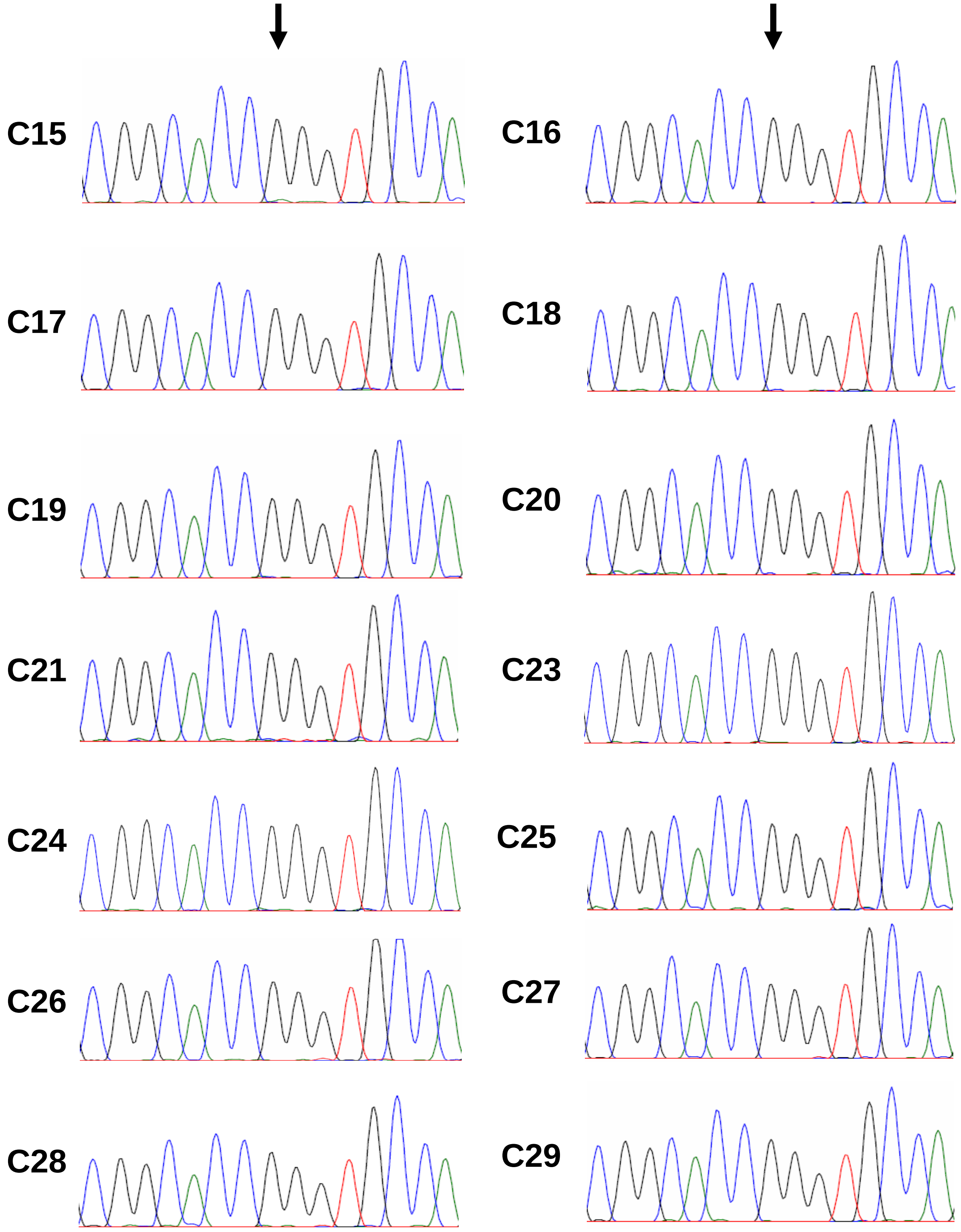

Controls for *CDH23*, IVS45-9G>A

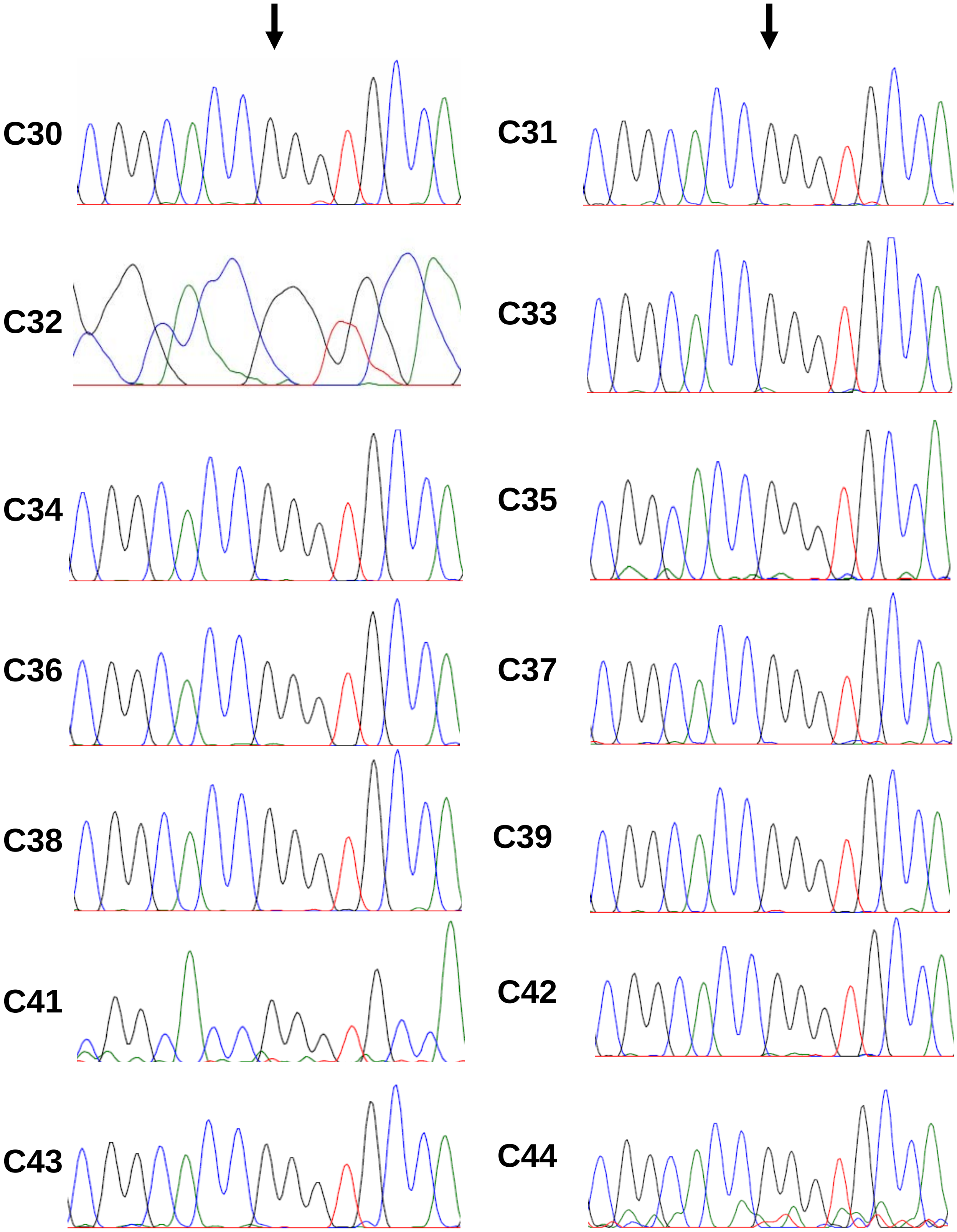

Controls for *CDH23*, IVS45-9G>A

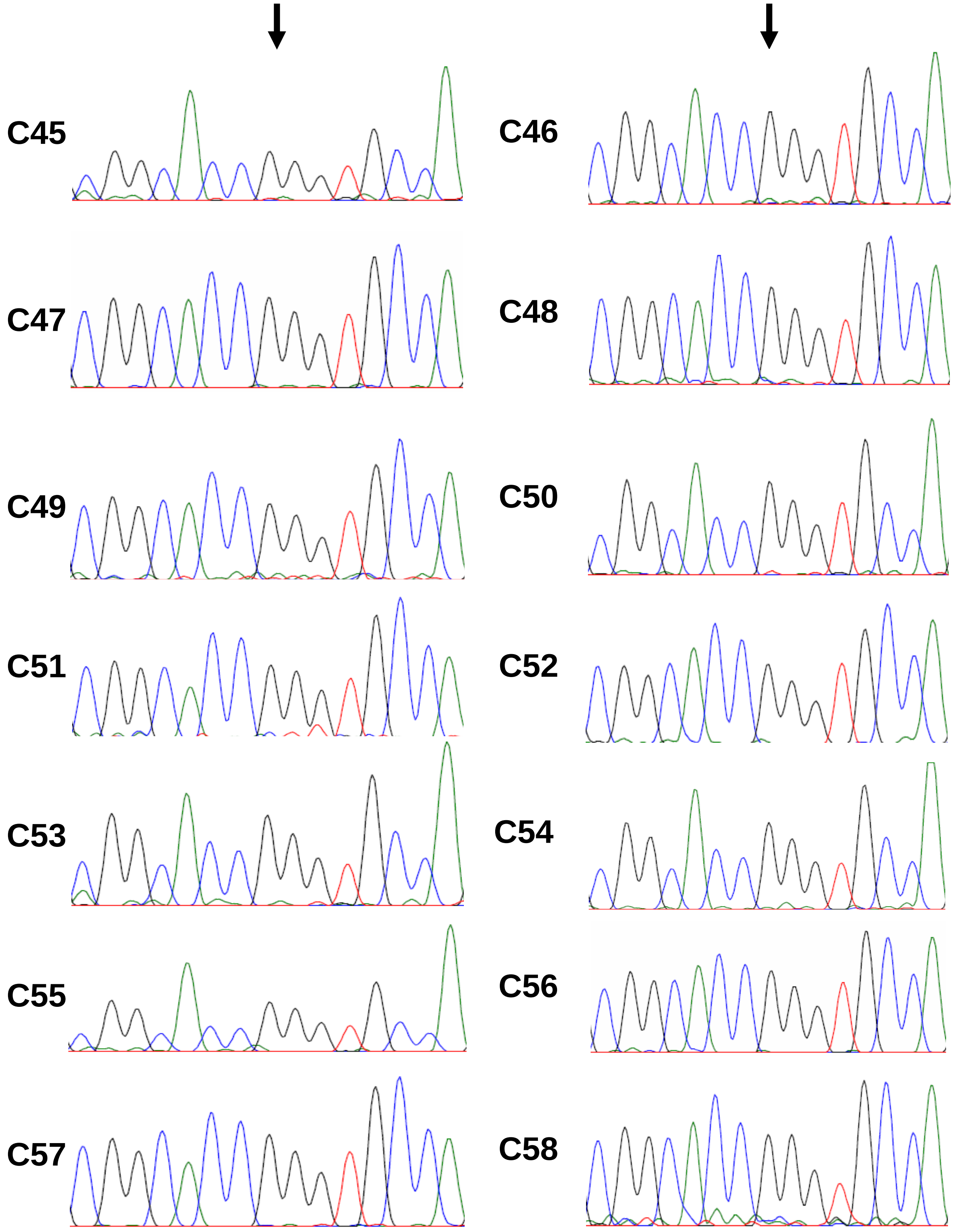

Controls for *CDH23*, IVS45-9G>A

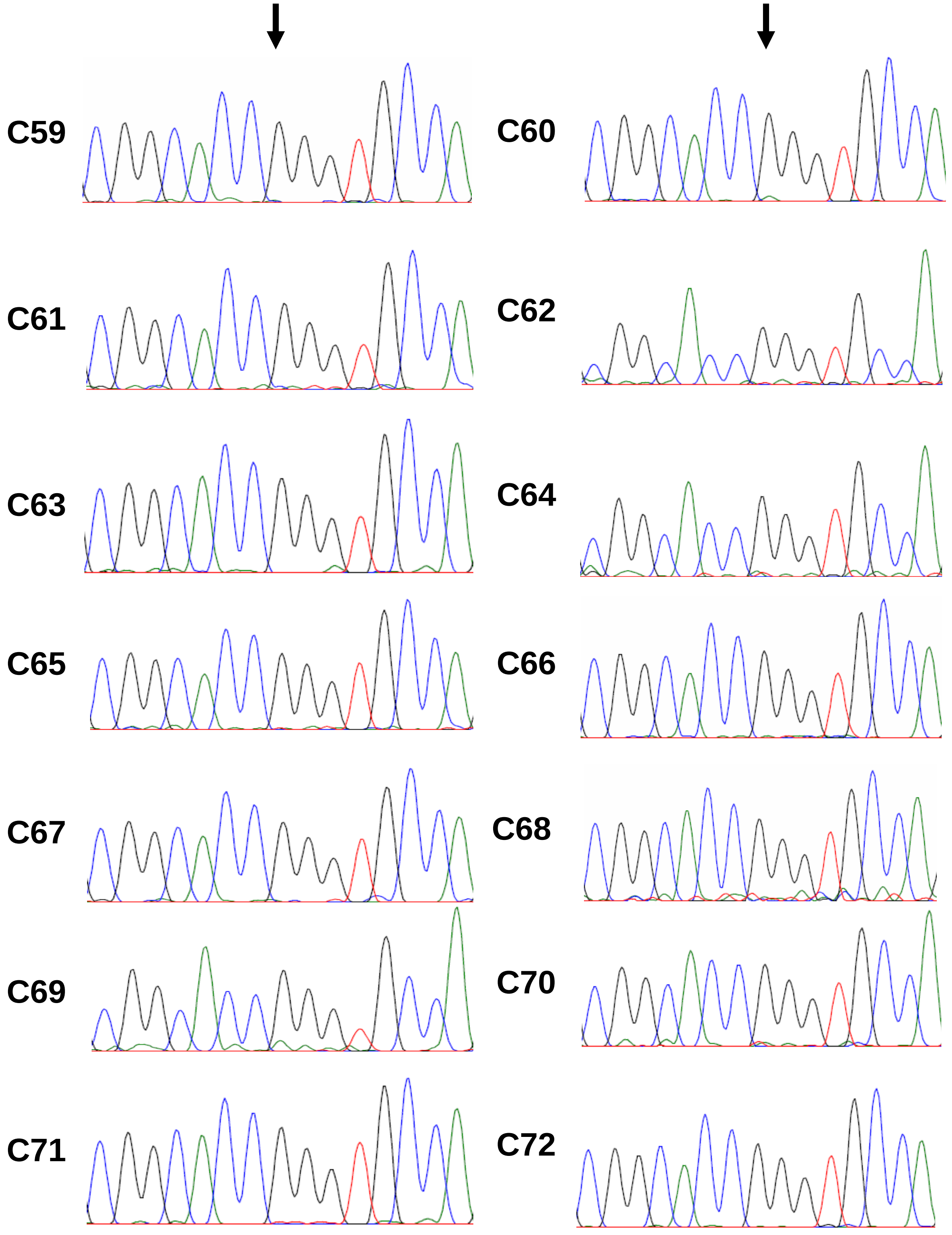

Controls for *CDH23*, IVS45-9G>A

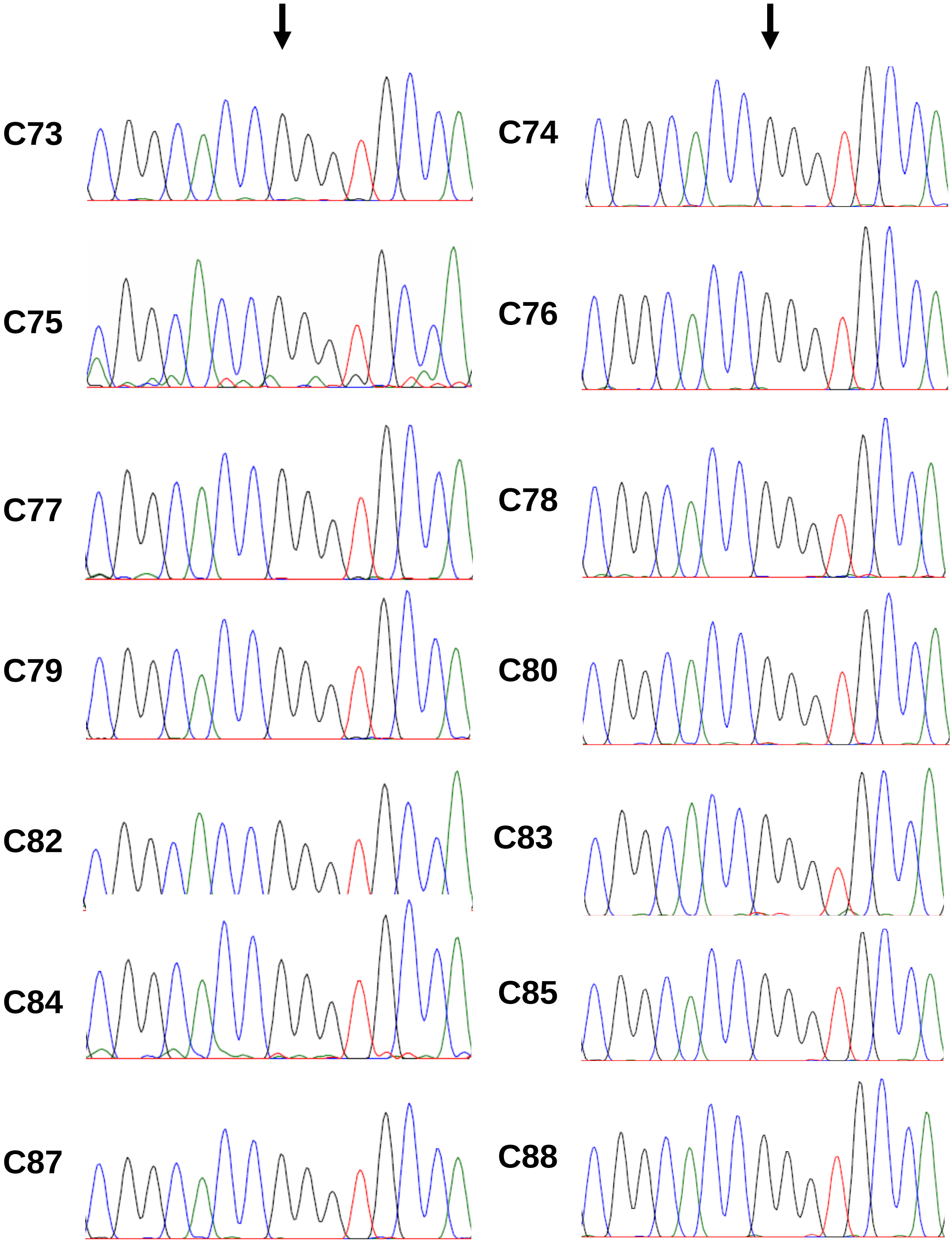

Controls for *CDH23*, IVS45-9G>A

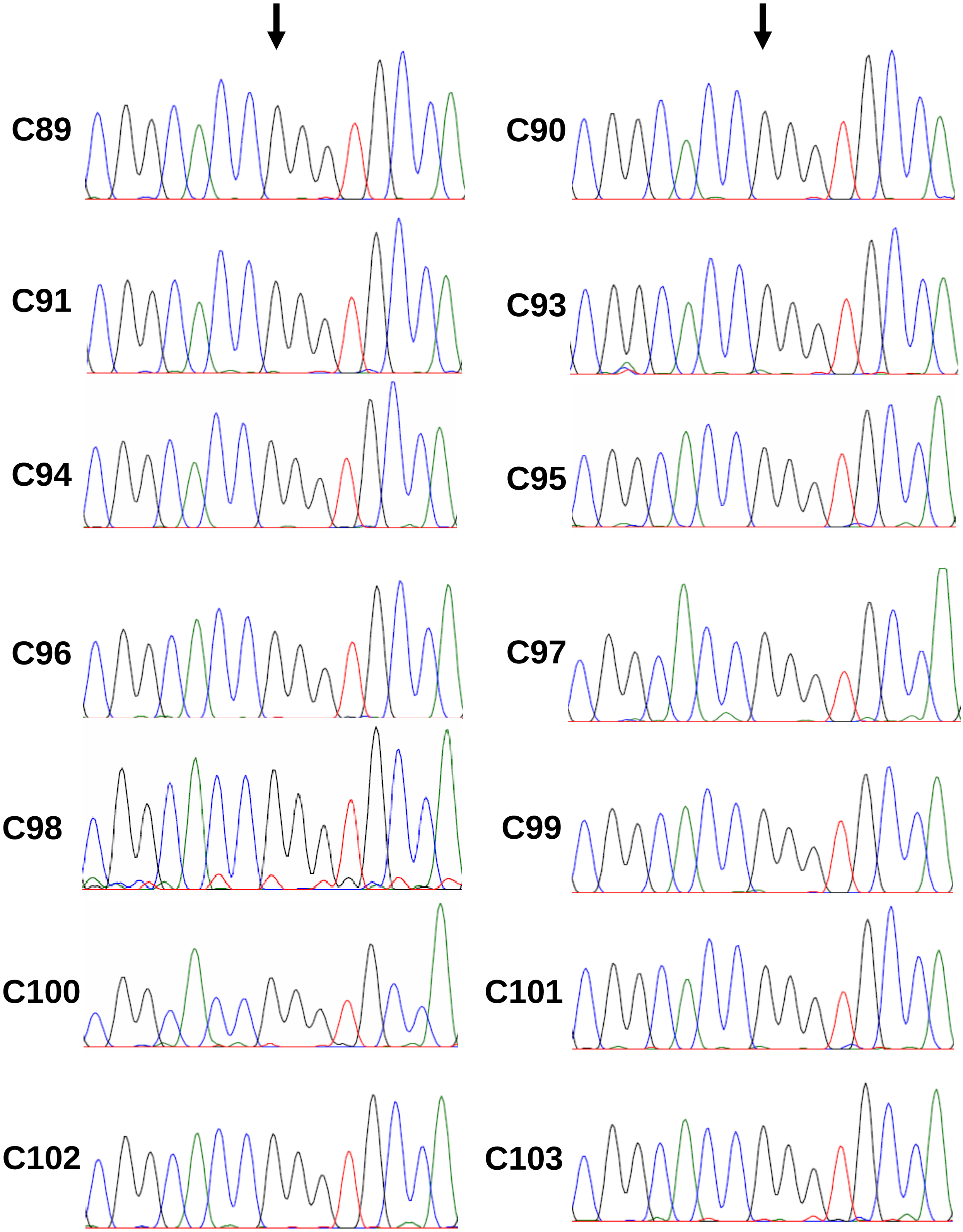

Controls for *CDH23*, IVS45-9G>A

C102

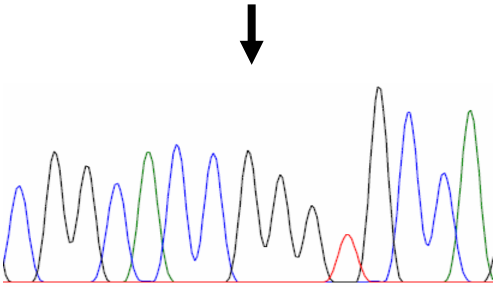

C110

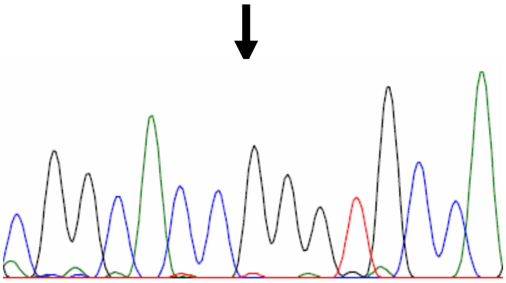

p.R736X (*CDH23*):
Genotyping of 100 French Canadian healthy control individuals

- by direct sequencing of PCR products -

Controls for *CDH23*, c. 2206C>T (p.R736X)

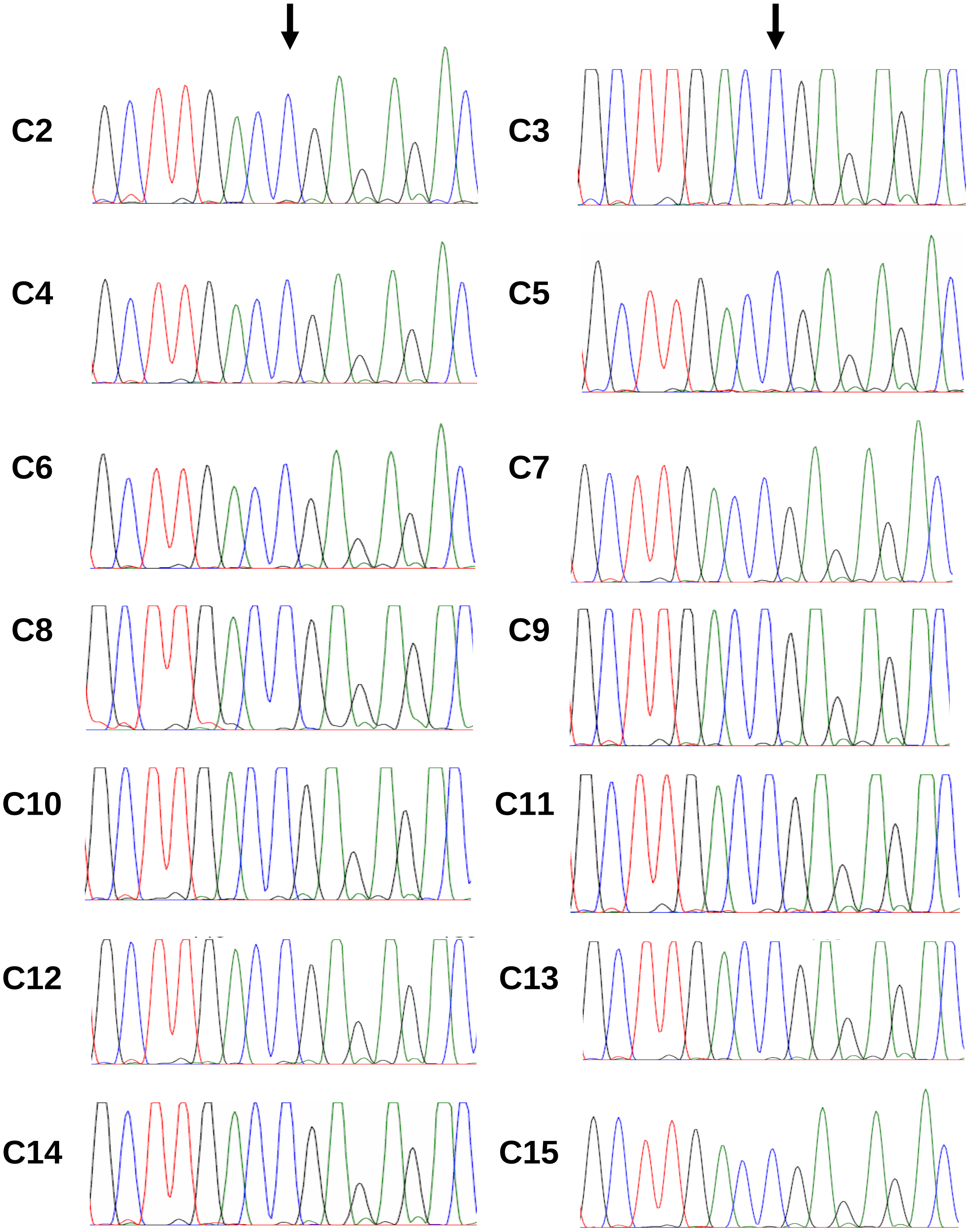

Controls for *CDH23*, c. 2206C>T (p.R736X)

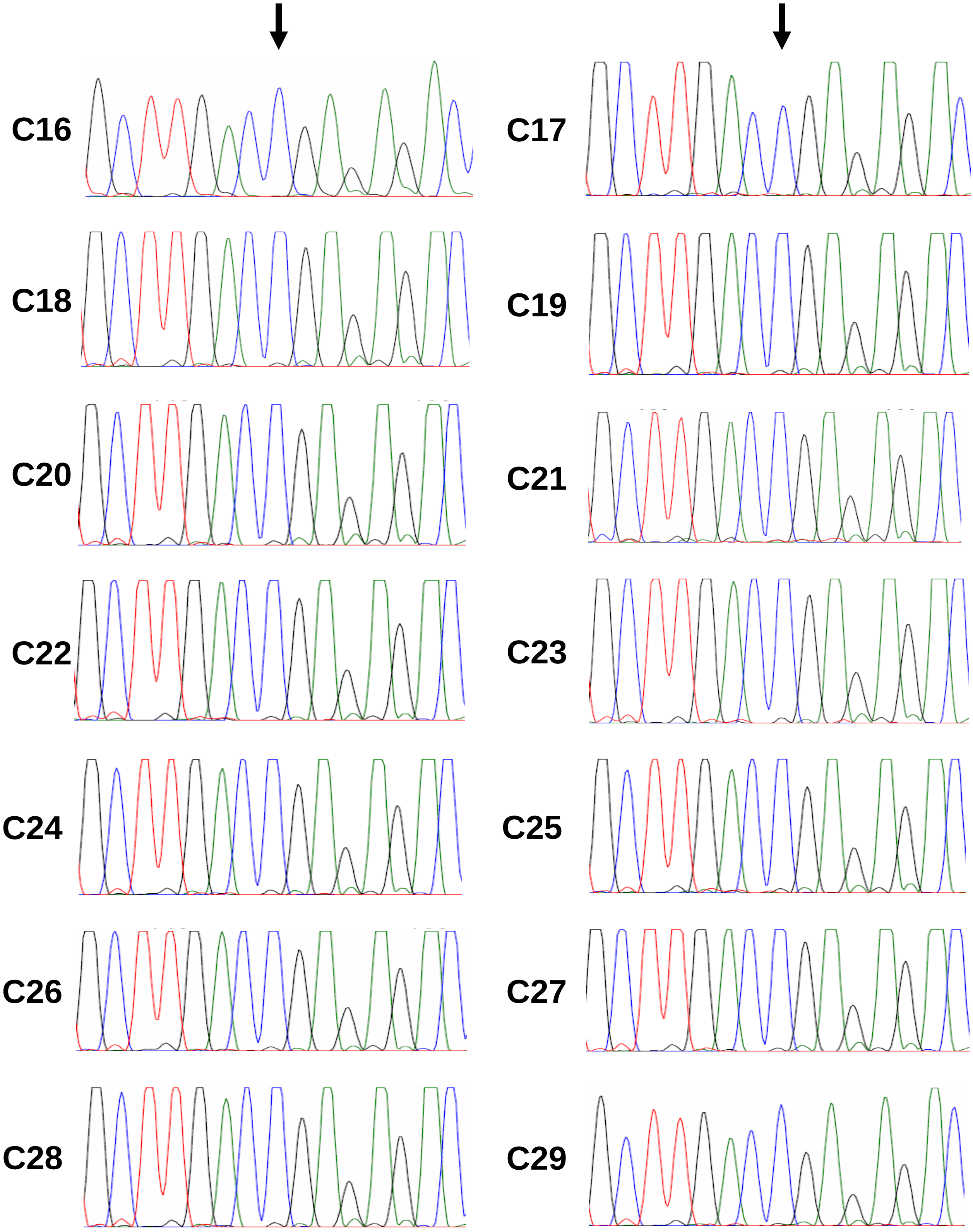

<u>Controls for *CDH23*, c. 2206C>T (p.R736X)</u>

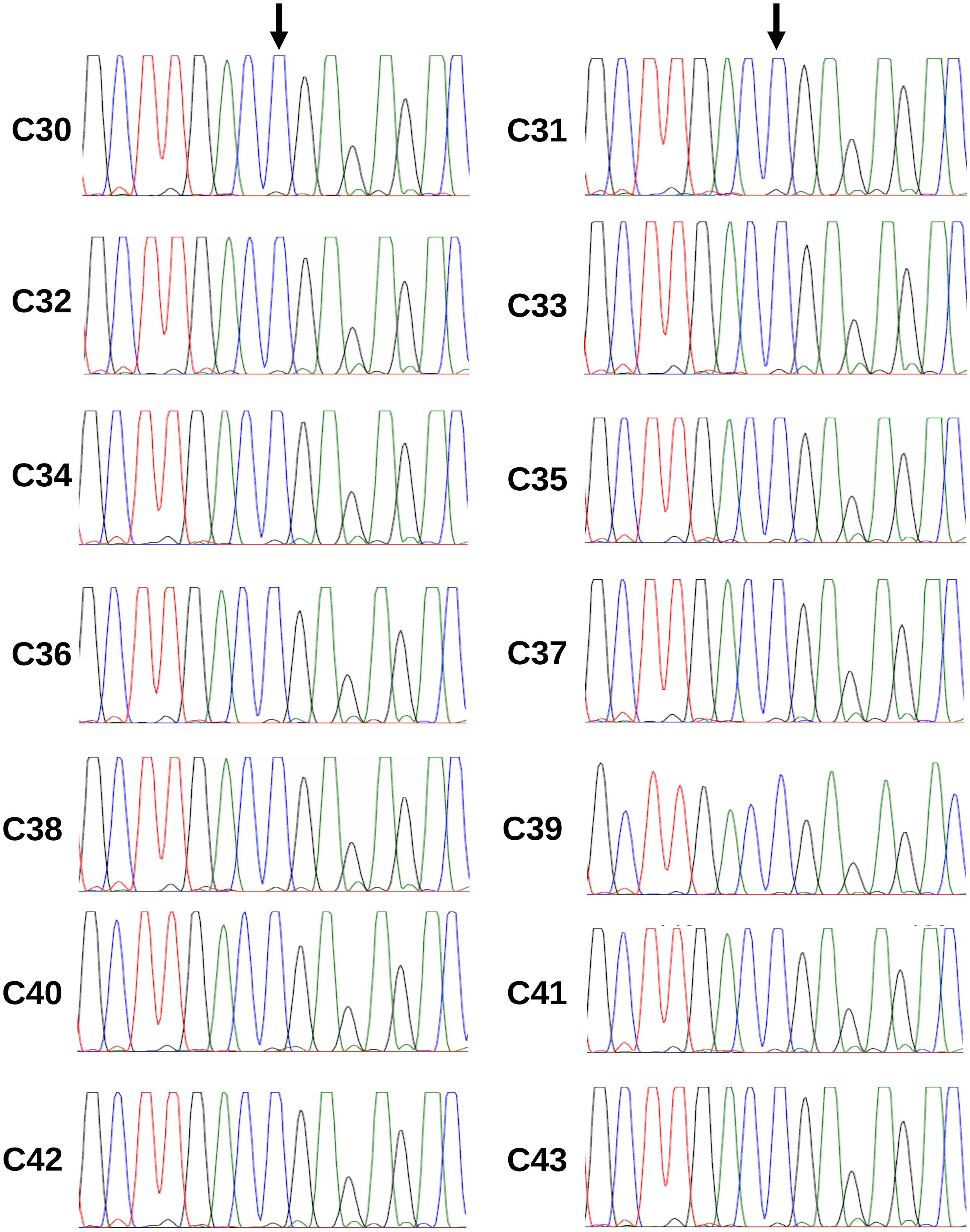

Controls for *CDH23*, c. 2206C>T (p.R736X)

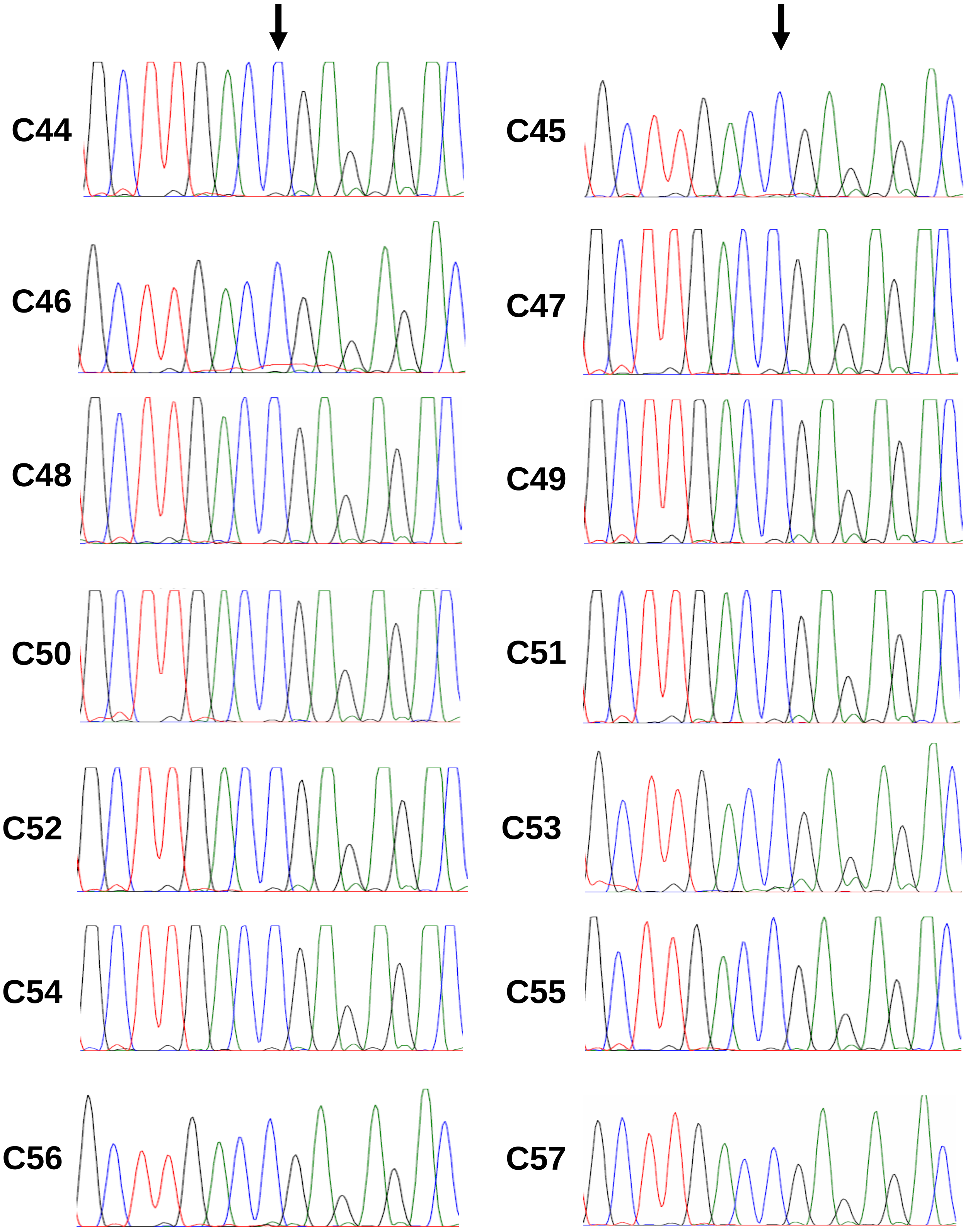

Controls for *CDH23*, c. 2206C>T (p.R736X)

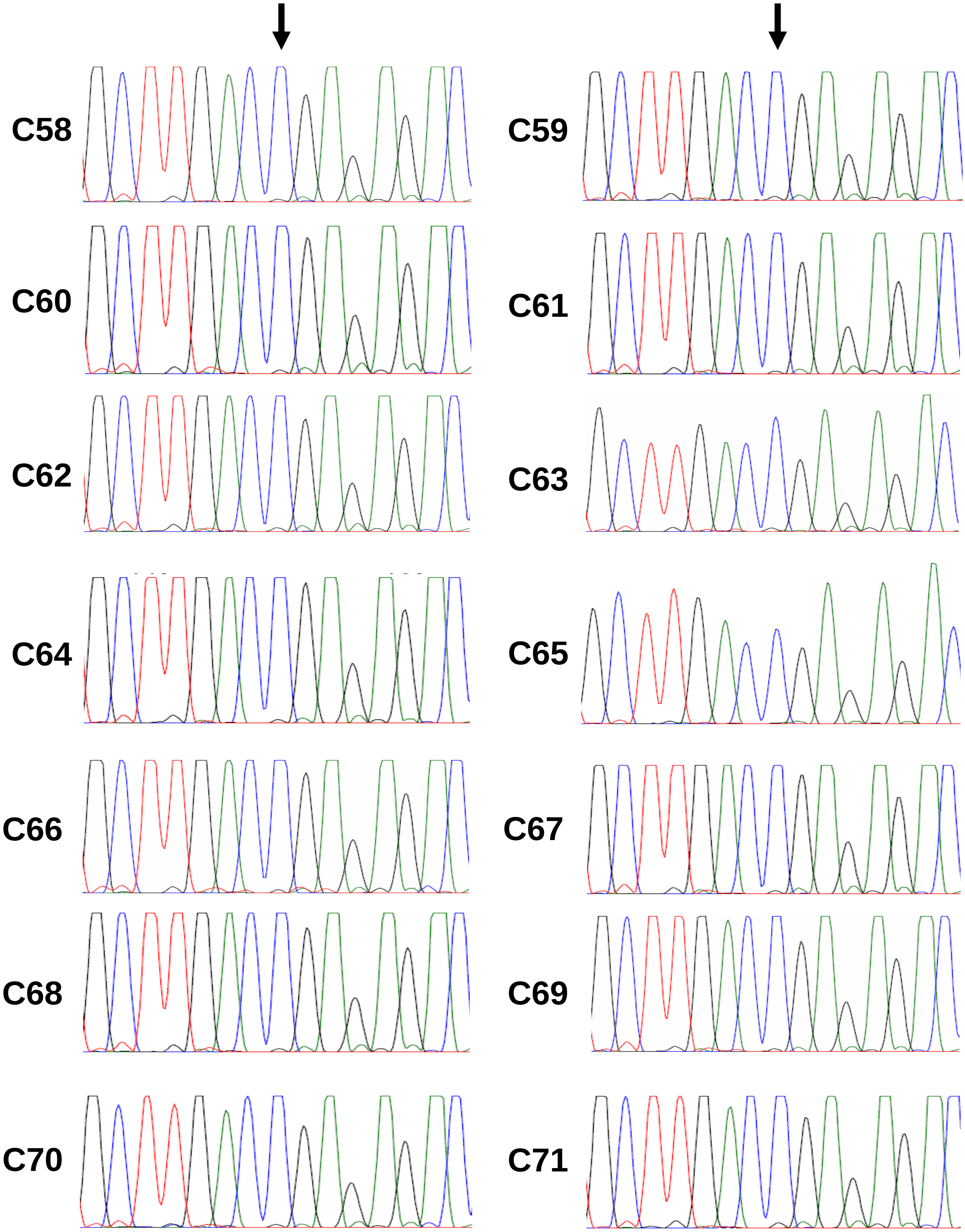

<u>**Controls for *CDH23*, c. 2206C>T (p.R736X)**</u>

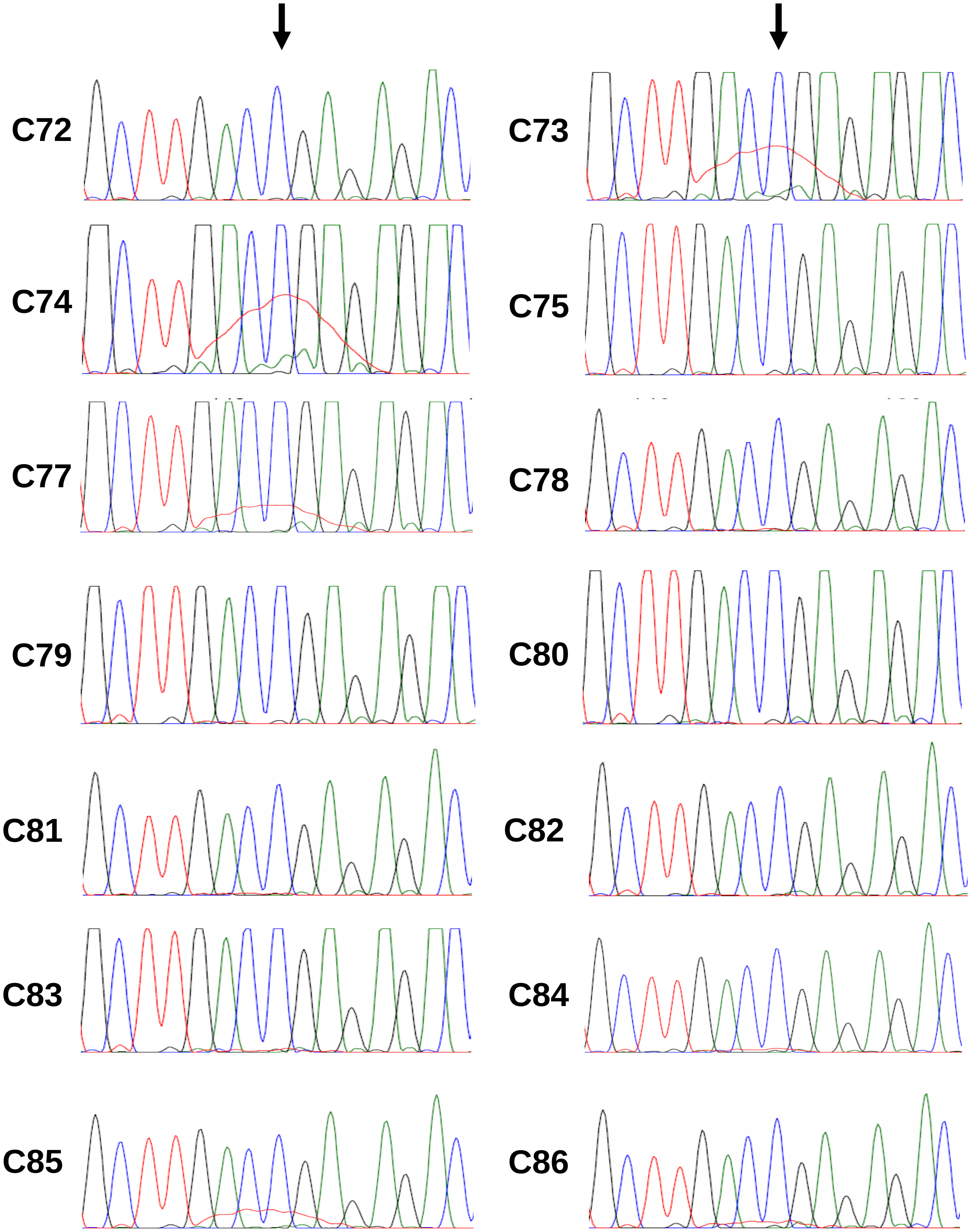

Controls for *CDH23*, c. 2206C>T (p.R736X)

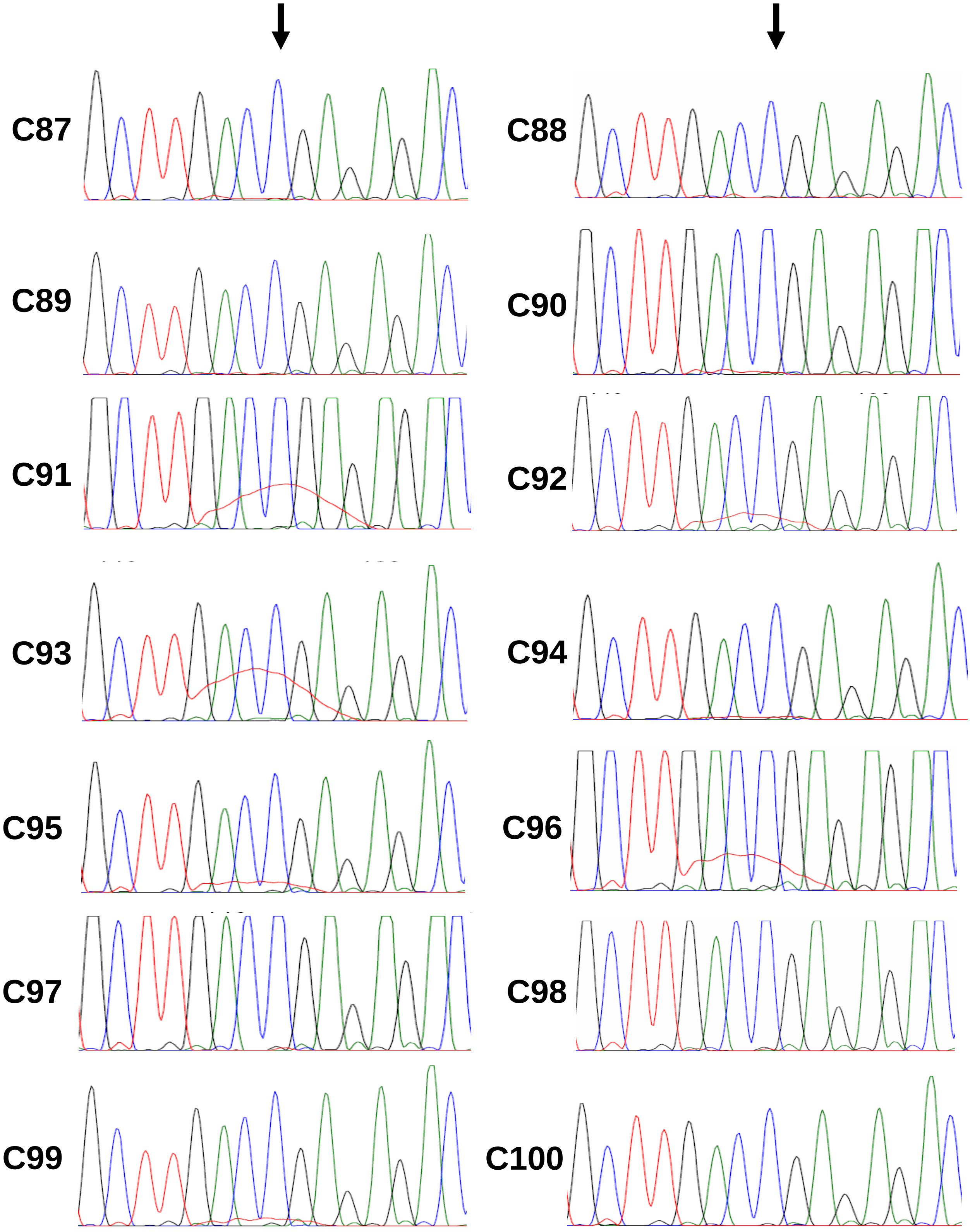

Controls for *CDH23*, c. 2206C>T (p.R736X)

C102

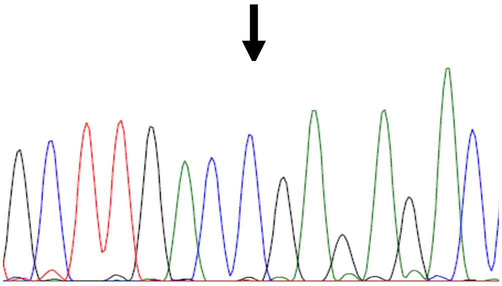

C110

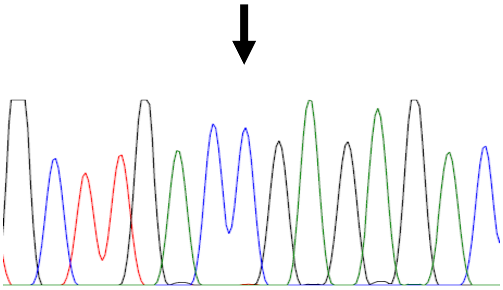

p.A457V (*MYO7A*):
Genotyping of 100 French Canadian healthy control individuals

- by direct sequencing of PCR products -

<u>Controls for *MYO7A*, c. 1370C>T (p.A457V)</u>

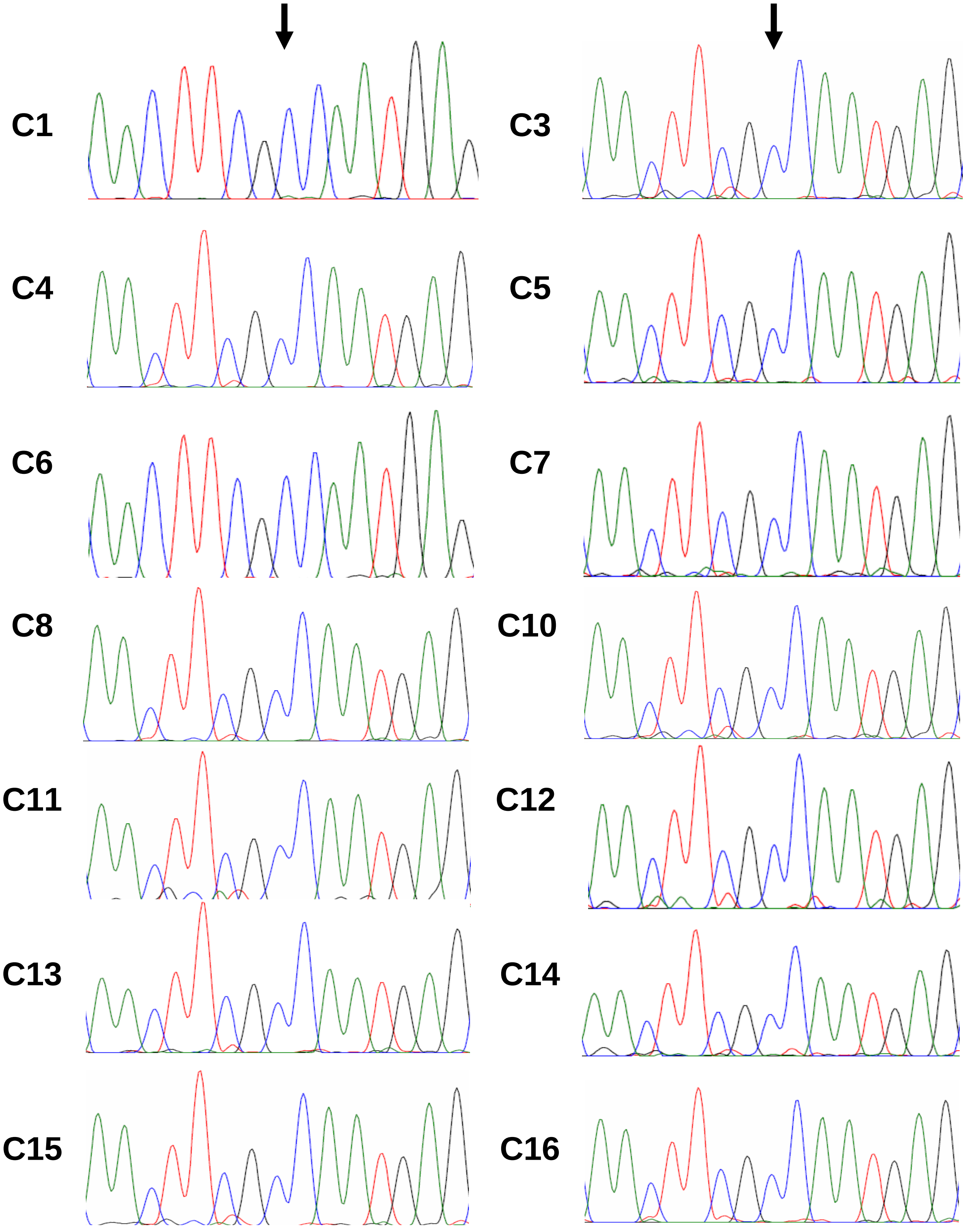

<u>Controls for *MYO7A*, c. 1370C>T (p.A457V)</u>

C17
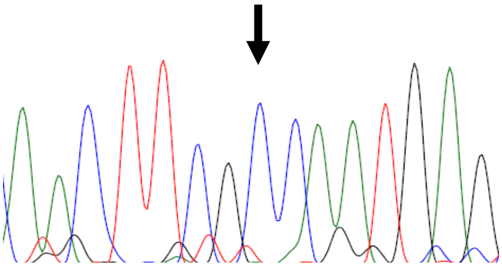

C18
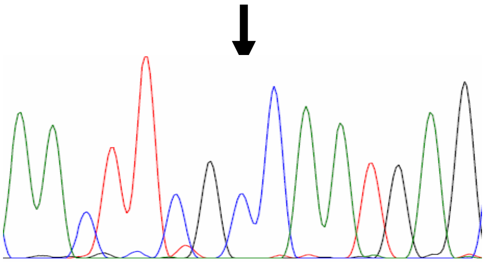

C19
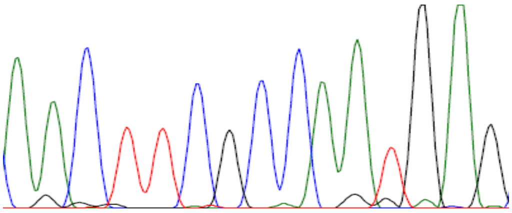

C20
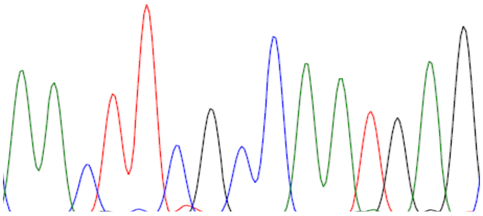

C22
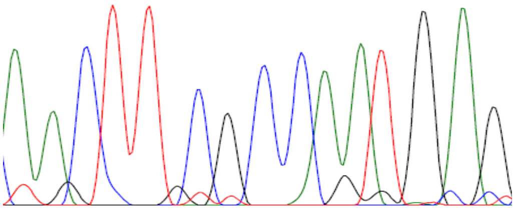

C23
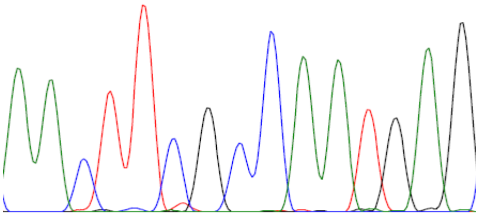

C24
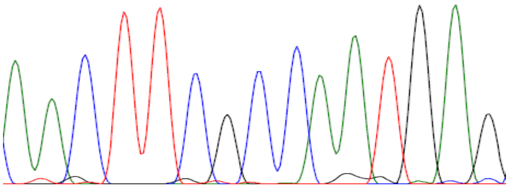

C25
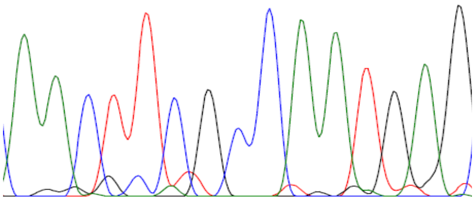

C26
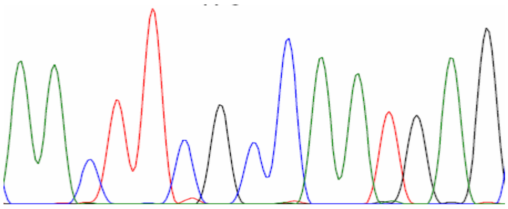

C27
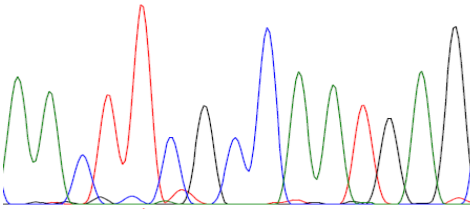

C28
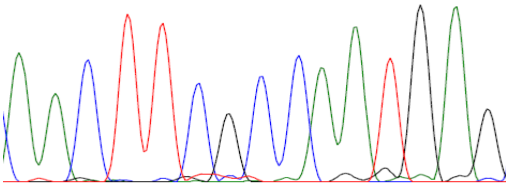

C29
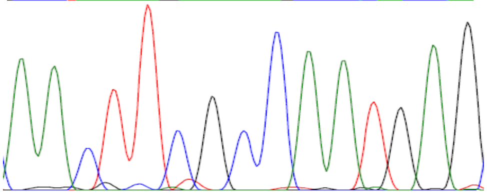

C30
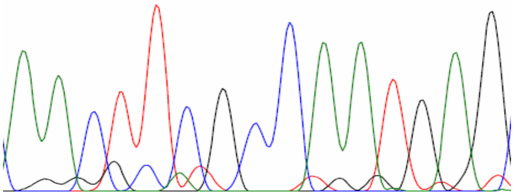

C32
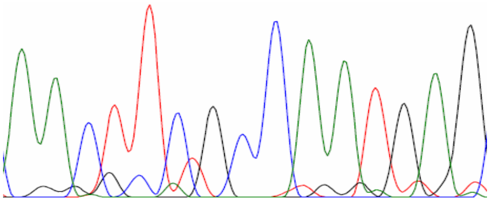

<u>Controls for *MYO7A*, c. 1370C>T (p.A457V)</u>

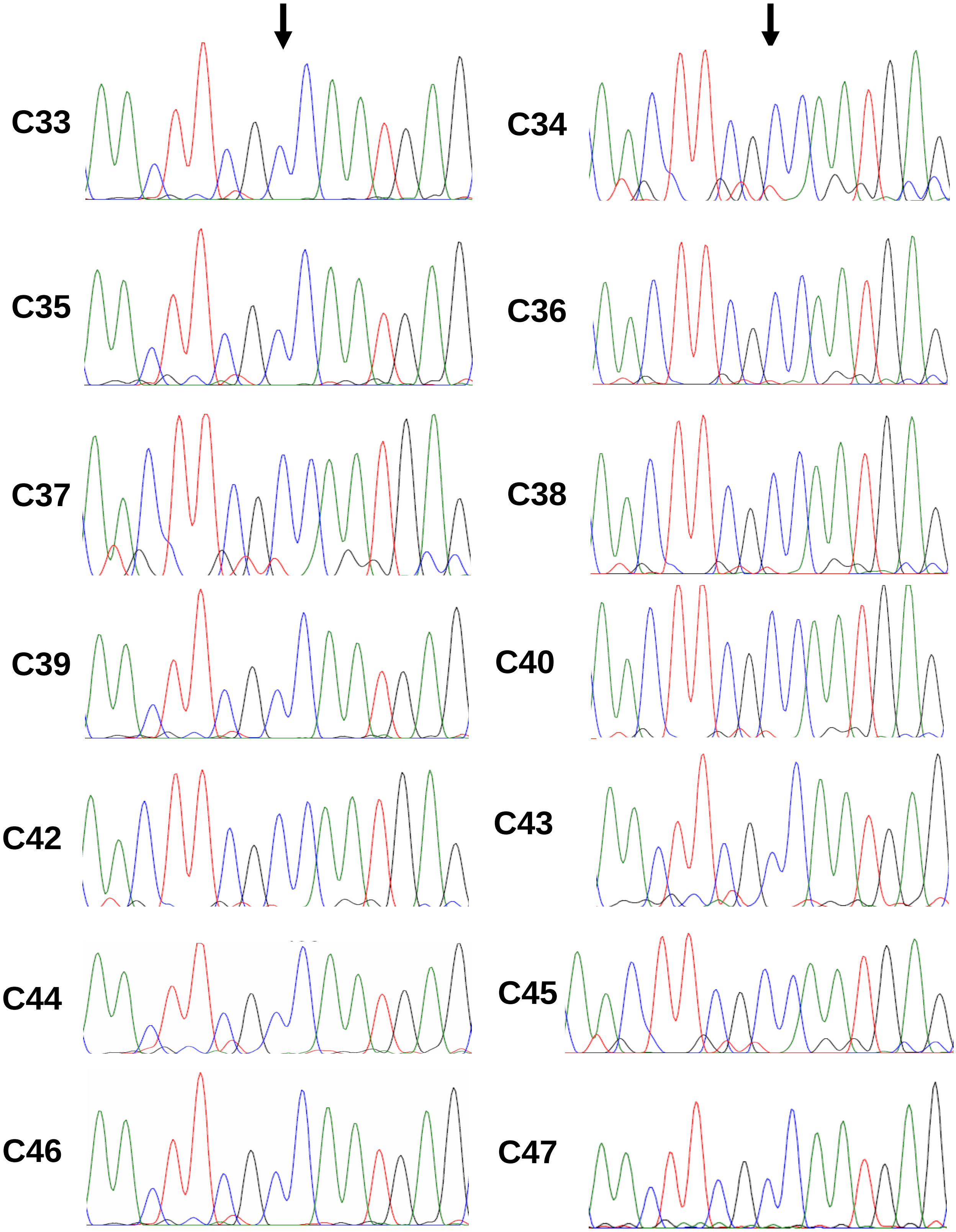

Controls for *MYO7A*, c. 1370C>T (p.A457V)

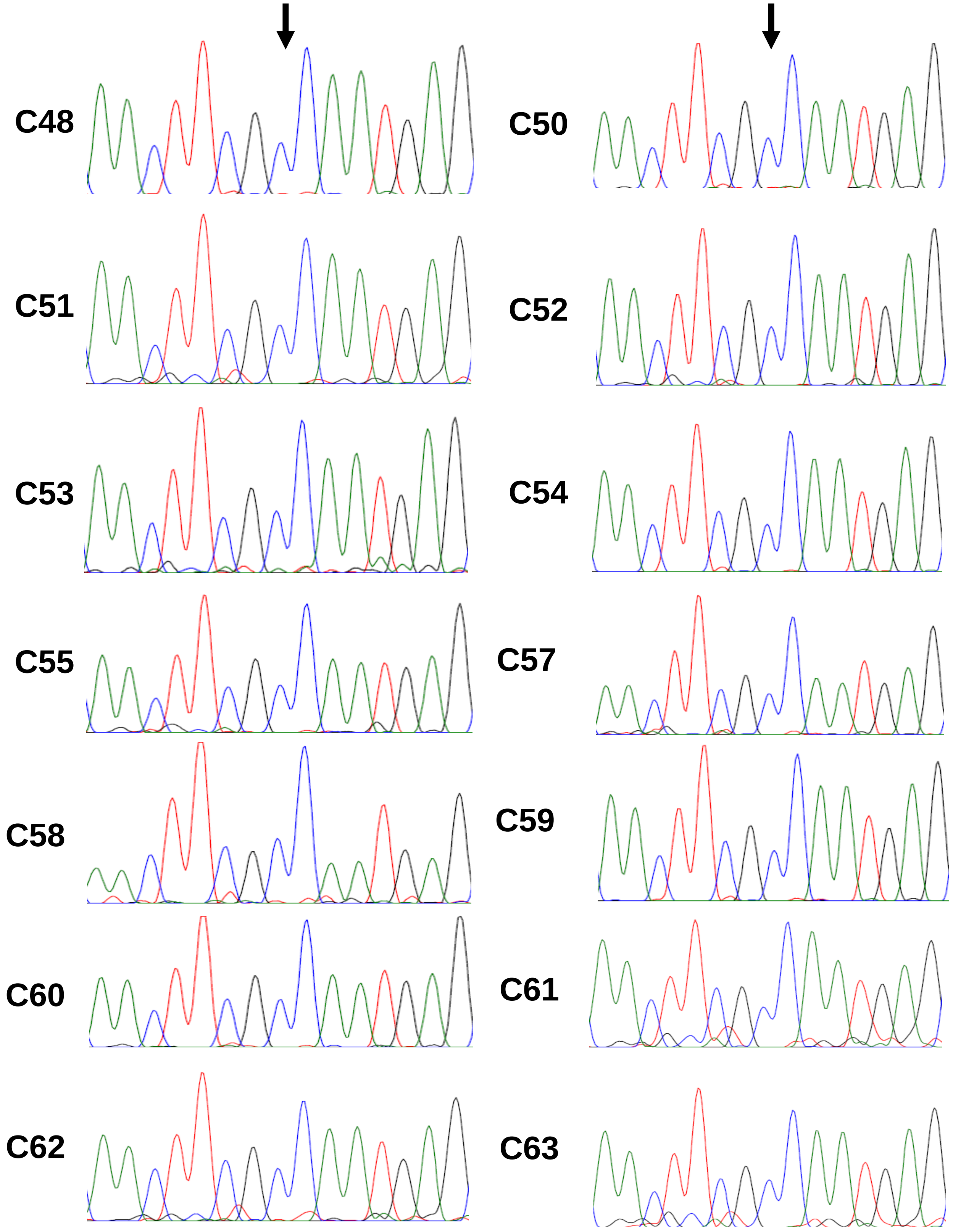

Controls for *MYO7A*, c. 1370C>T (p.A457V)

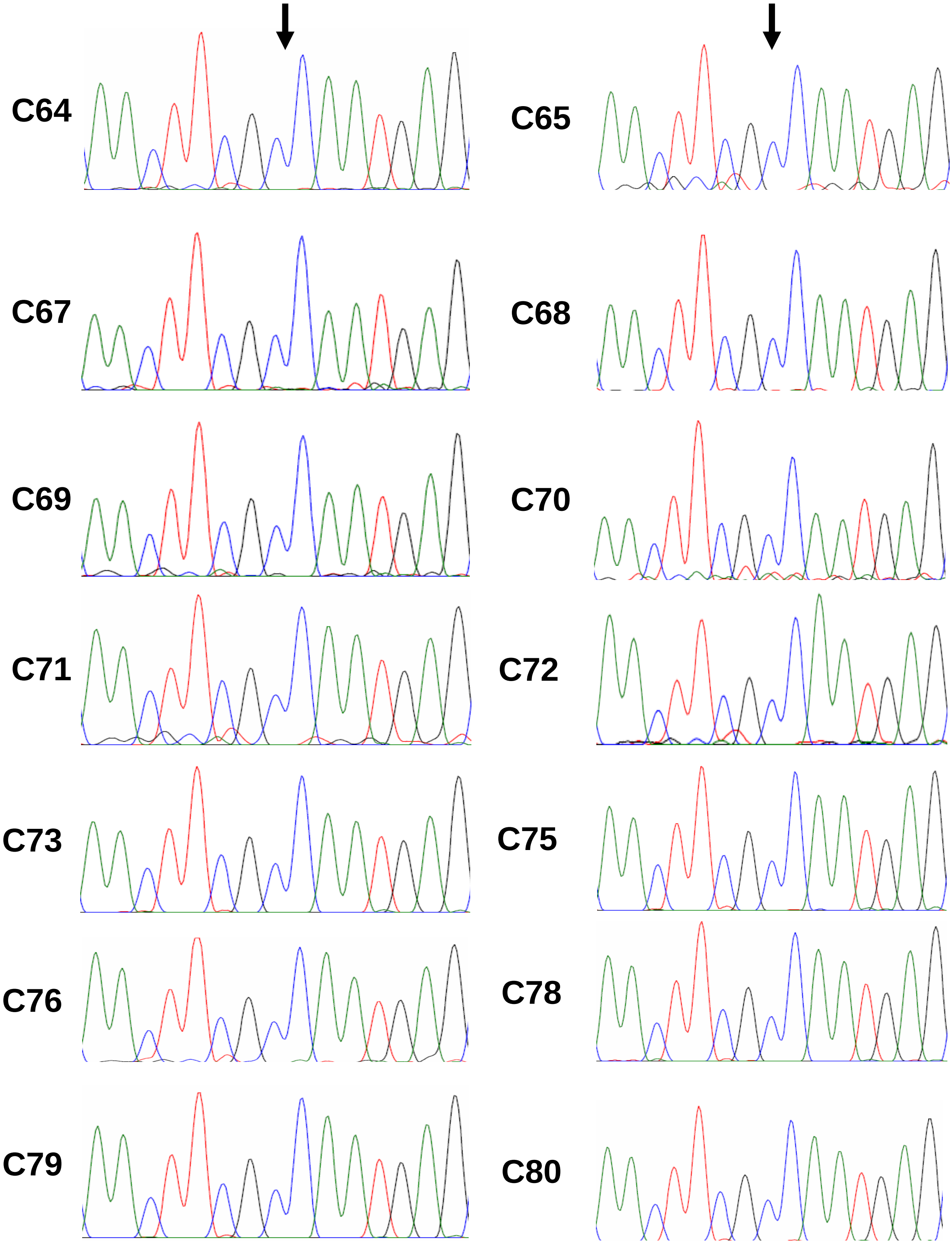

Controls for *MYO7A*, c. 1370C>T (p.A457V)

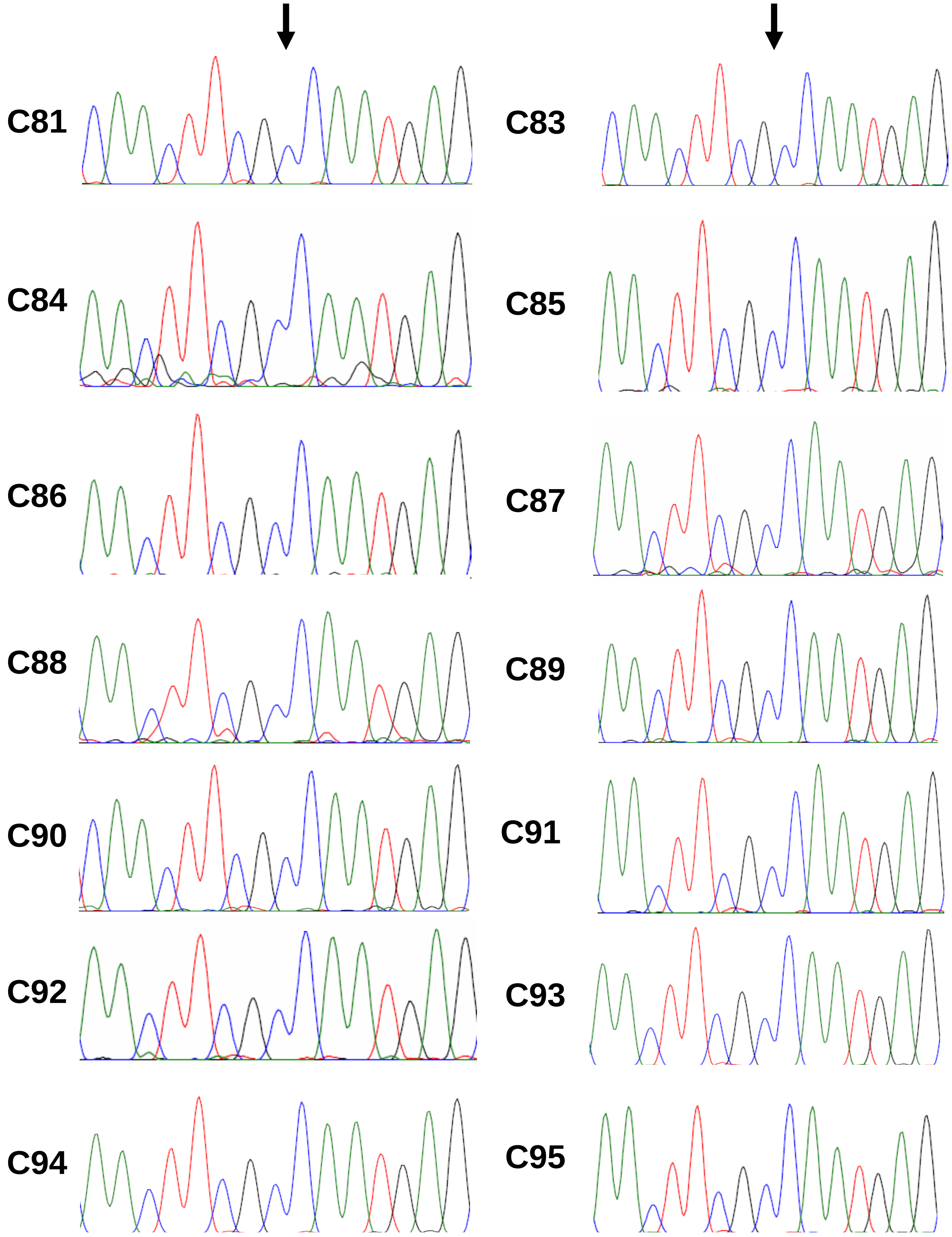

Controls for *MYO7A*, c. 1370C>T (p.A457V)

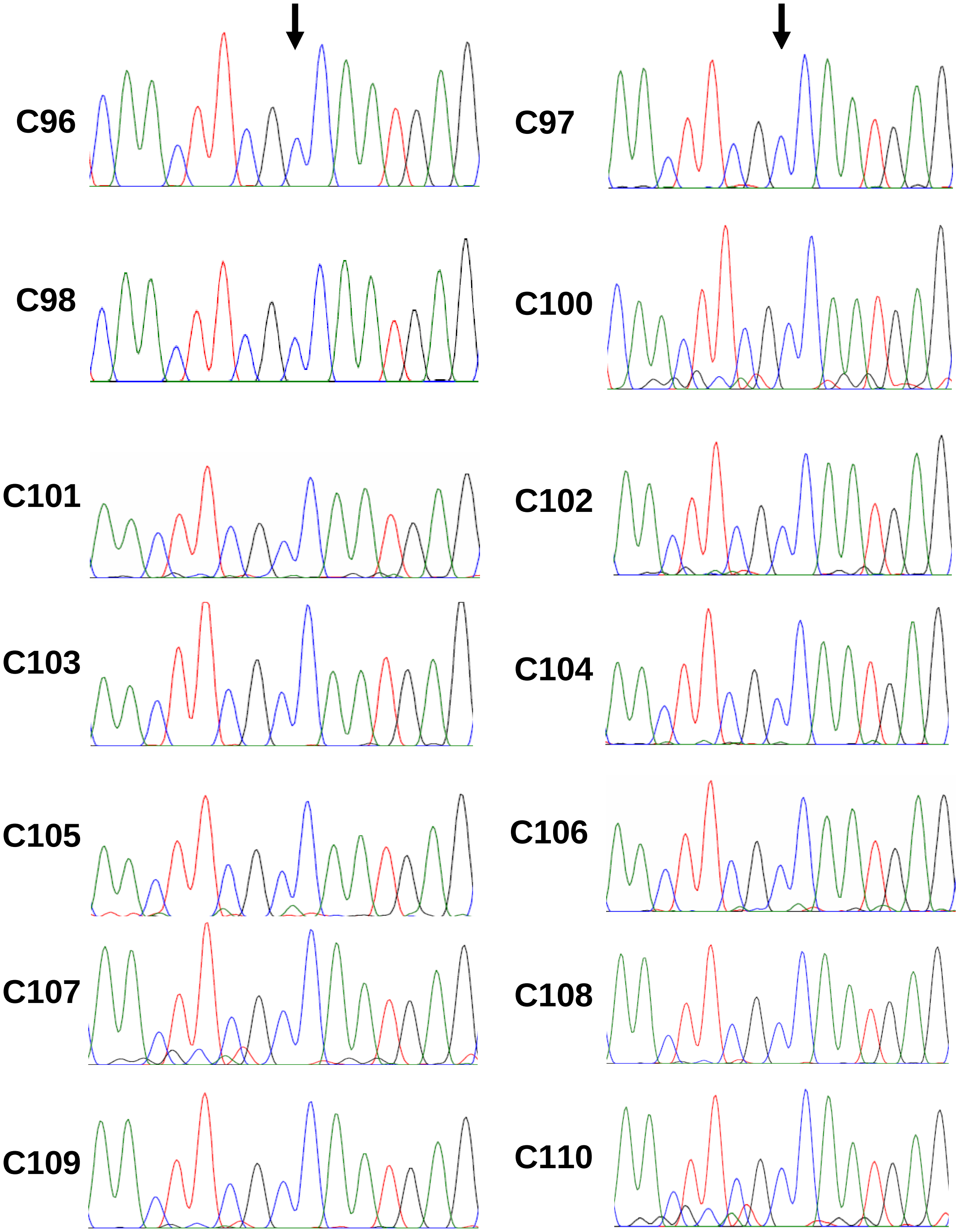

<u>Controls for *MYO7A*, c.1370C>T (p.A457V)</u>

C112

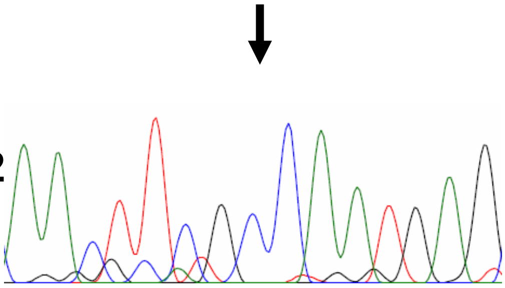

C110

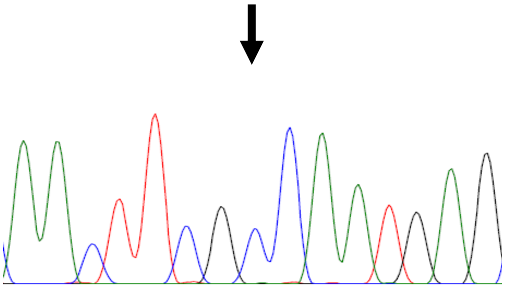

p.Q815X (*MYO7A*):
Genotyping of 100 French Canadian healthy control individuals

PCR products (1199 bp) were digested with *Nhe*I. Presence of c.2443C>T creates a *Nhe*I site in the mutant, resulting in fragments of 295 and 904 bp.

Controls for c.2443 C>T/p.Q815X (*MYO7A*, exon 21) by restriction digest with *Nhe*I

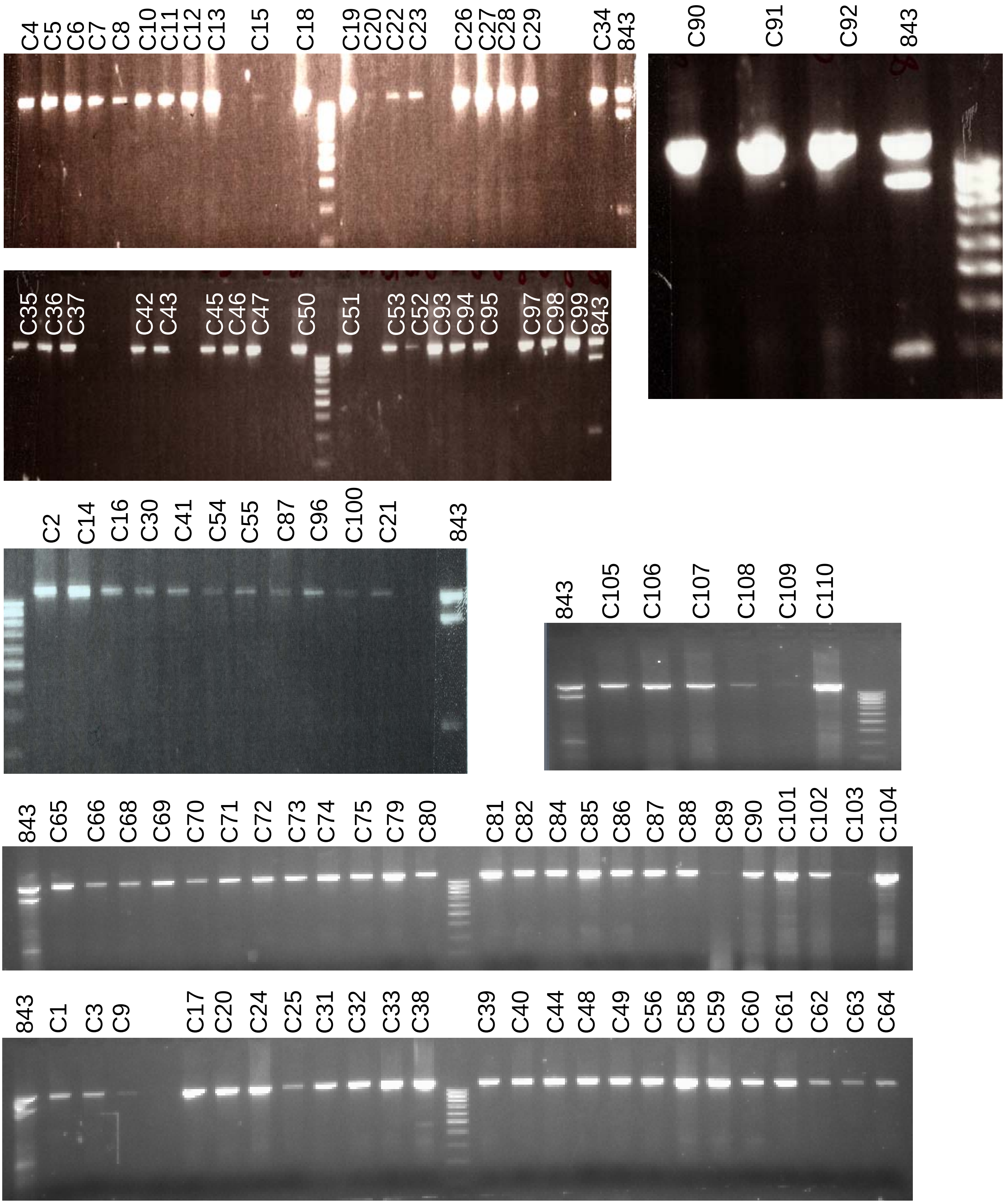

843: patient heterozygous for p.Q815X

p.A123D (*USH3A*):
Genotyping of 100 French Canadian healthy control individuals

- by direct sequencing of PCR products -

<u>Controls for *USH3A*, c.368C>T (p.A123D)</u>

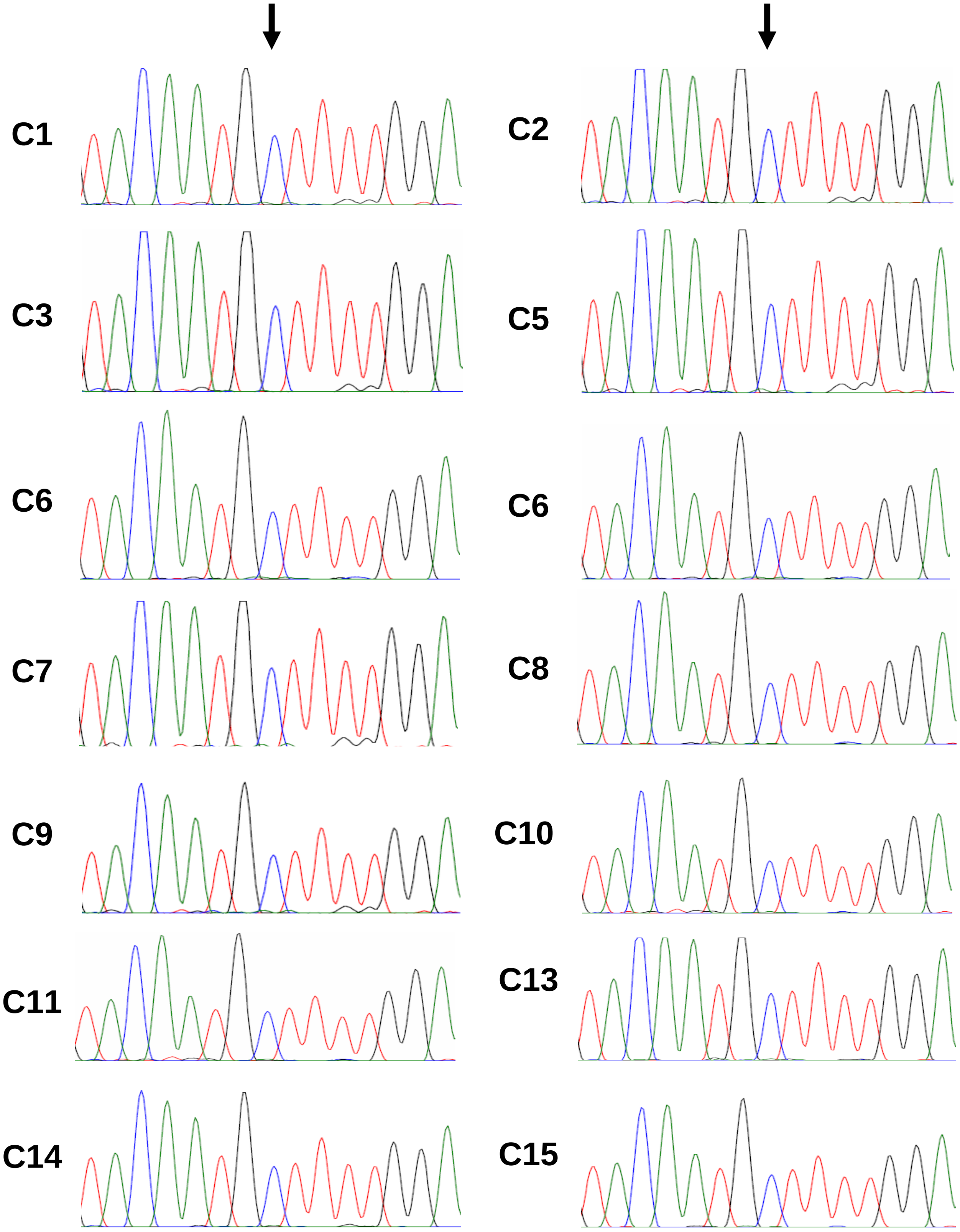

<u>Controls for *USH3A*, c.368C>T (p.A123D)</u>

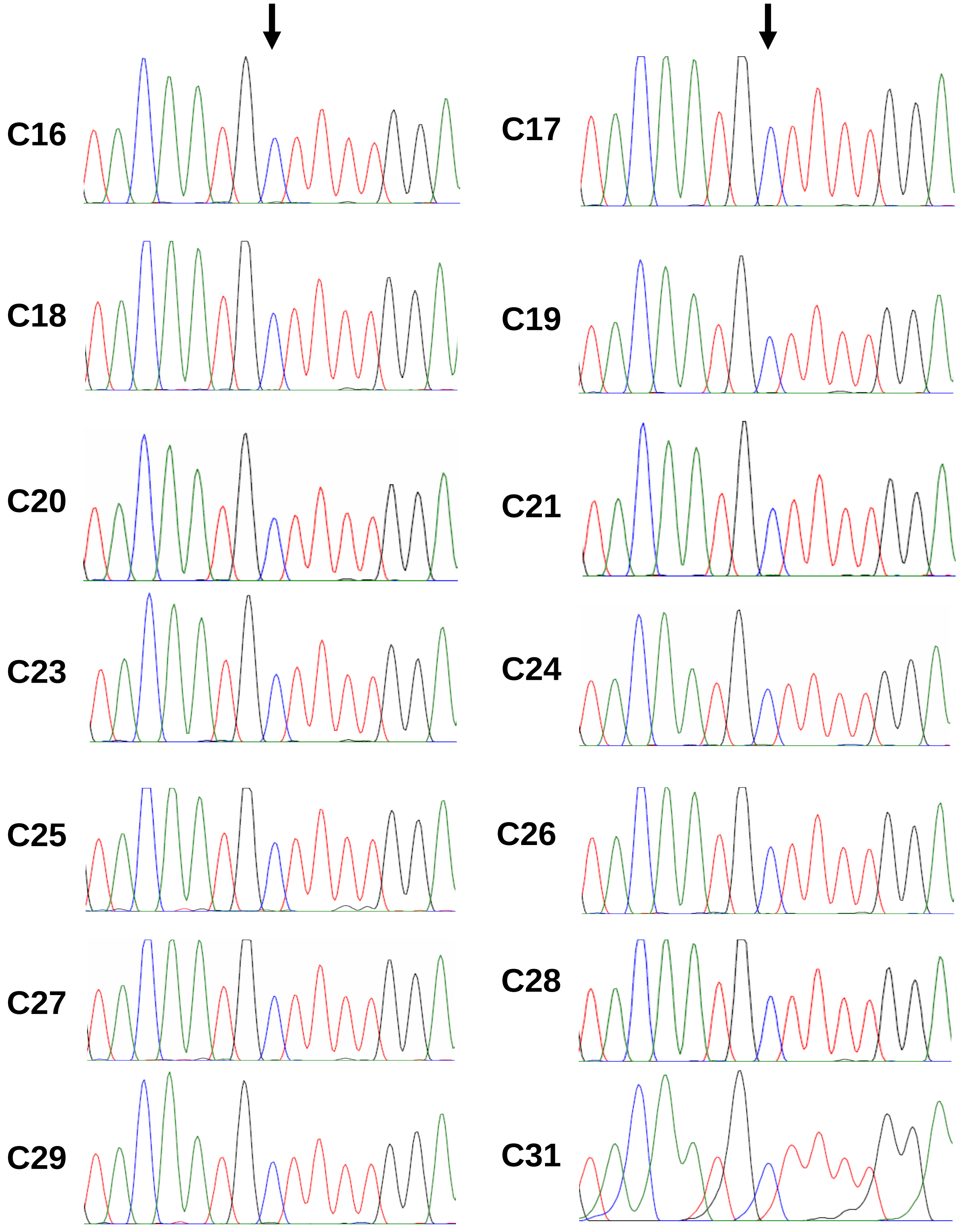

Controls for *USH3A*, c.368C>T (p.A123D)

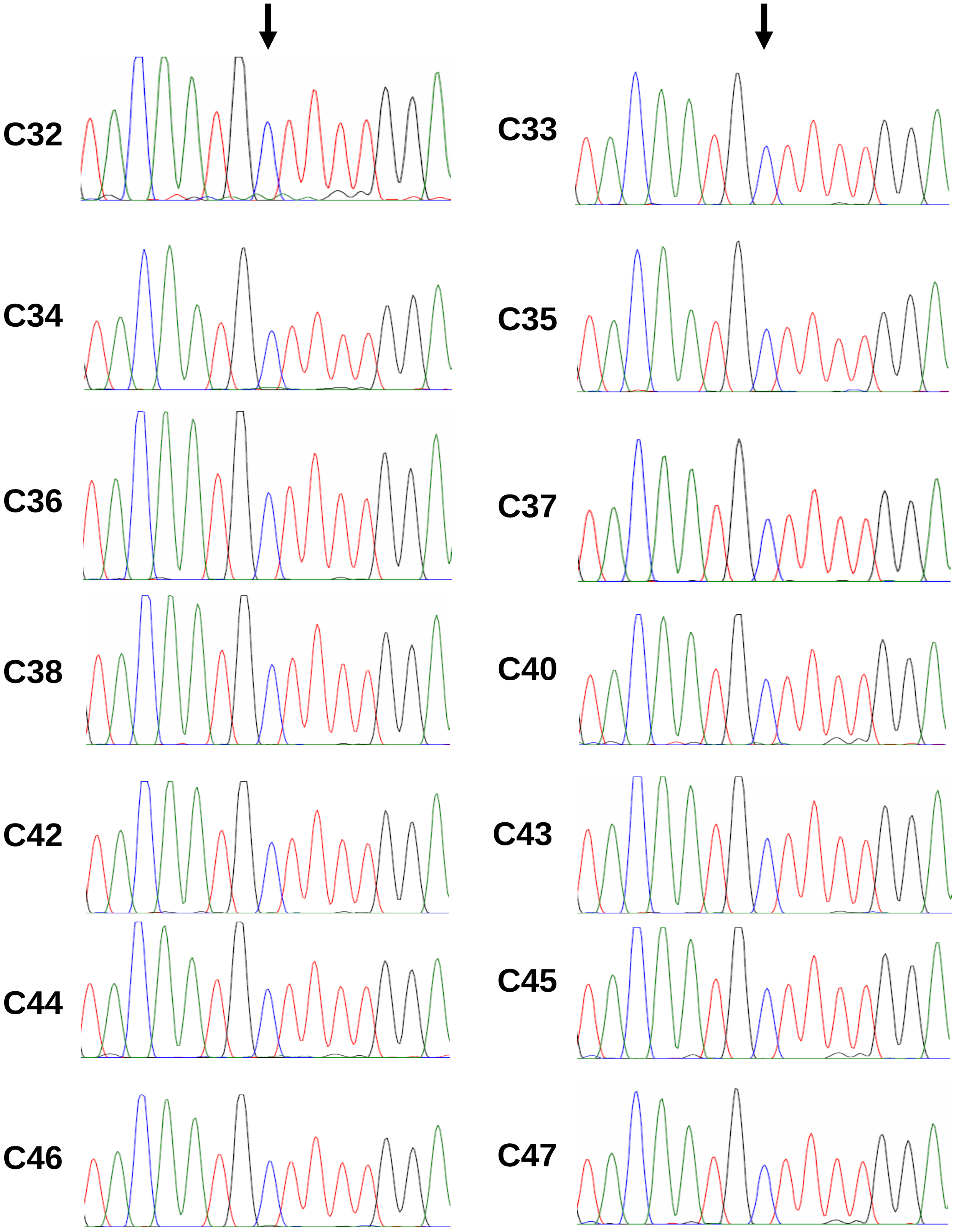

Controls for *USH3A*, c.368C>T (p.A123D)

C48

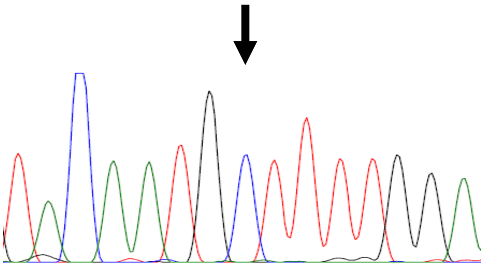

C49

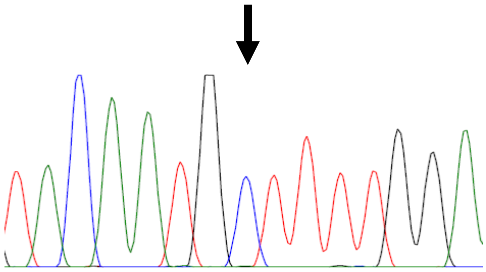

C50

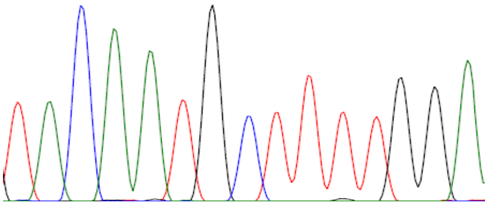

C51

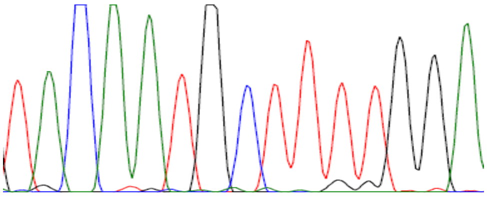

C53

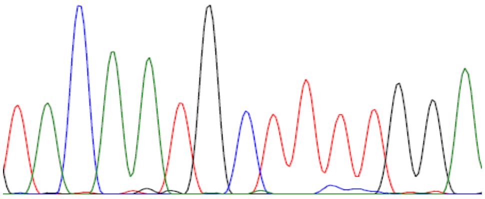

C54

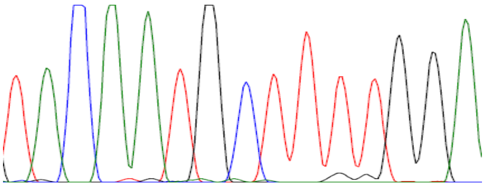

C55

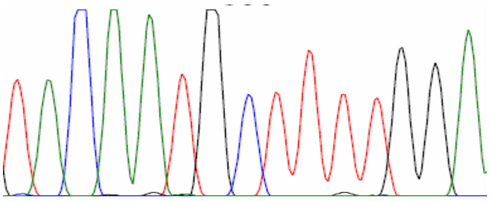

C56

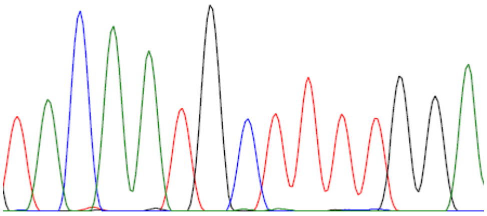

C57

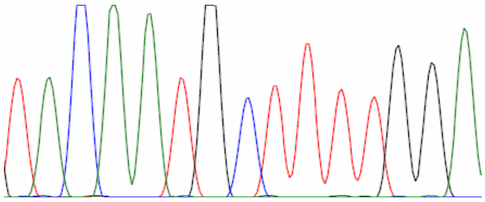

C58

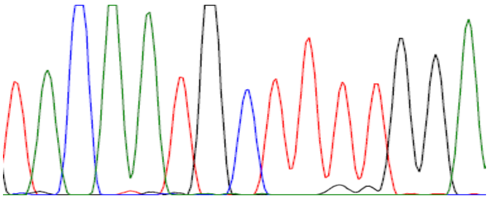

C59

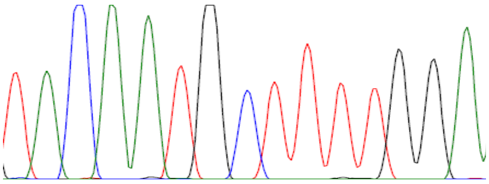

C60

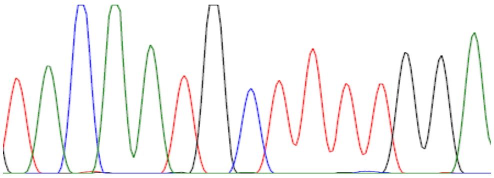

C61

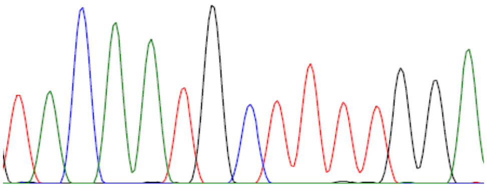

C62

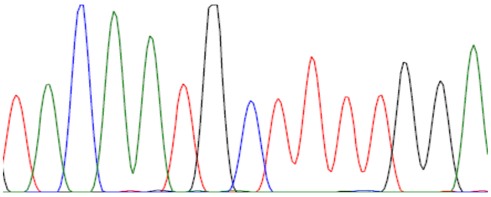

Controls for *USH3A*, c.368C>T (p.A123D)

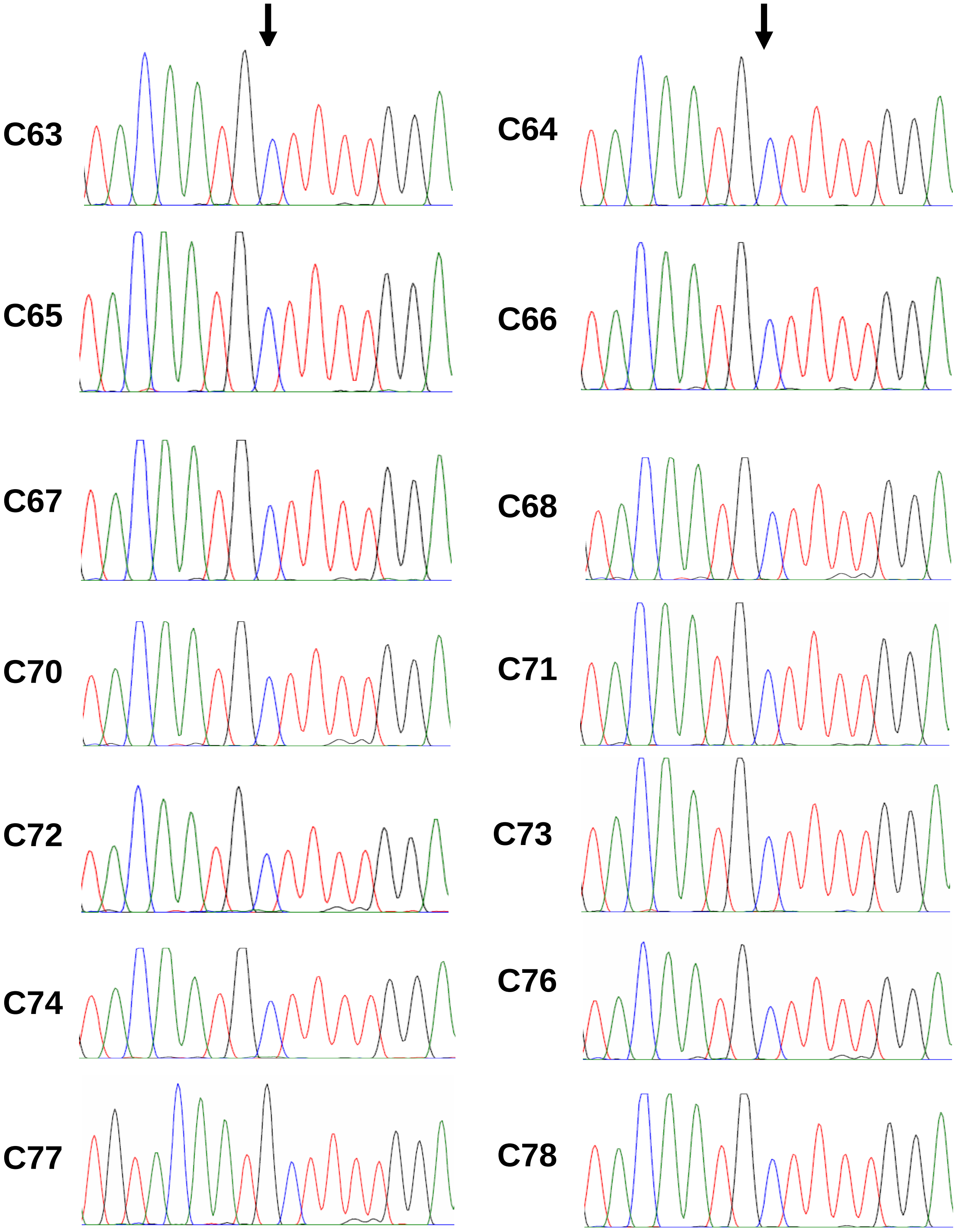

Controls for *USH3A*, c.368C>T (p.A123D)

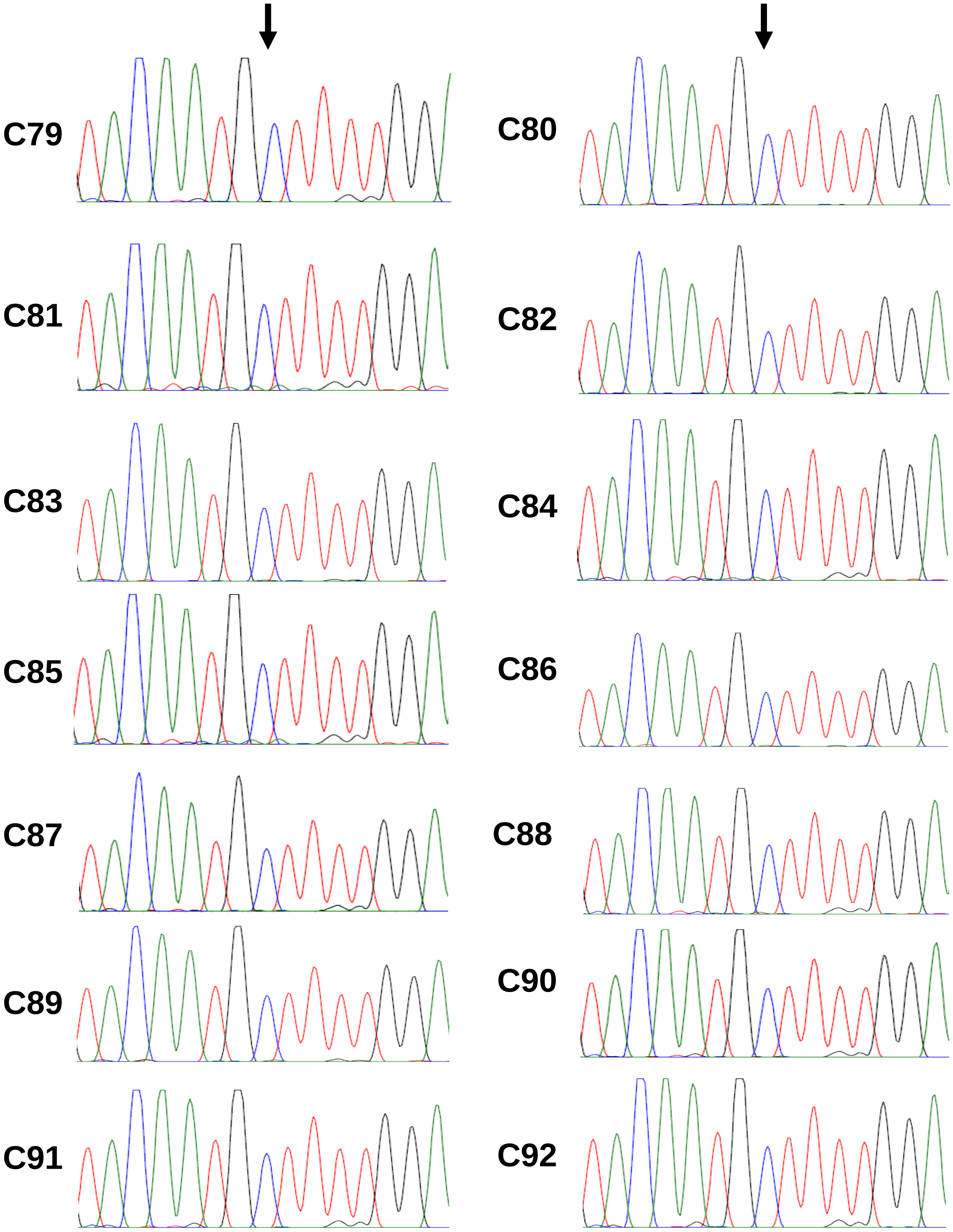

Controls for *USH3A*, c.368C>T (p.A123D)

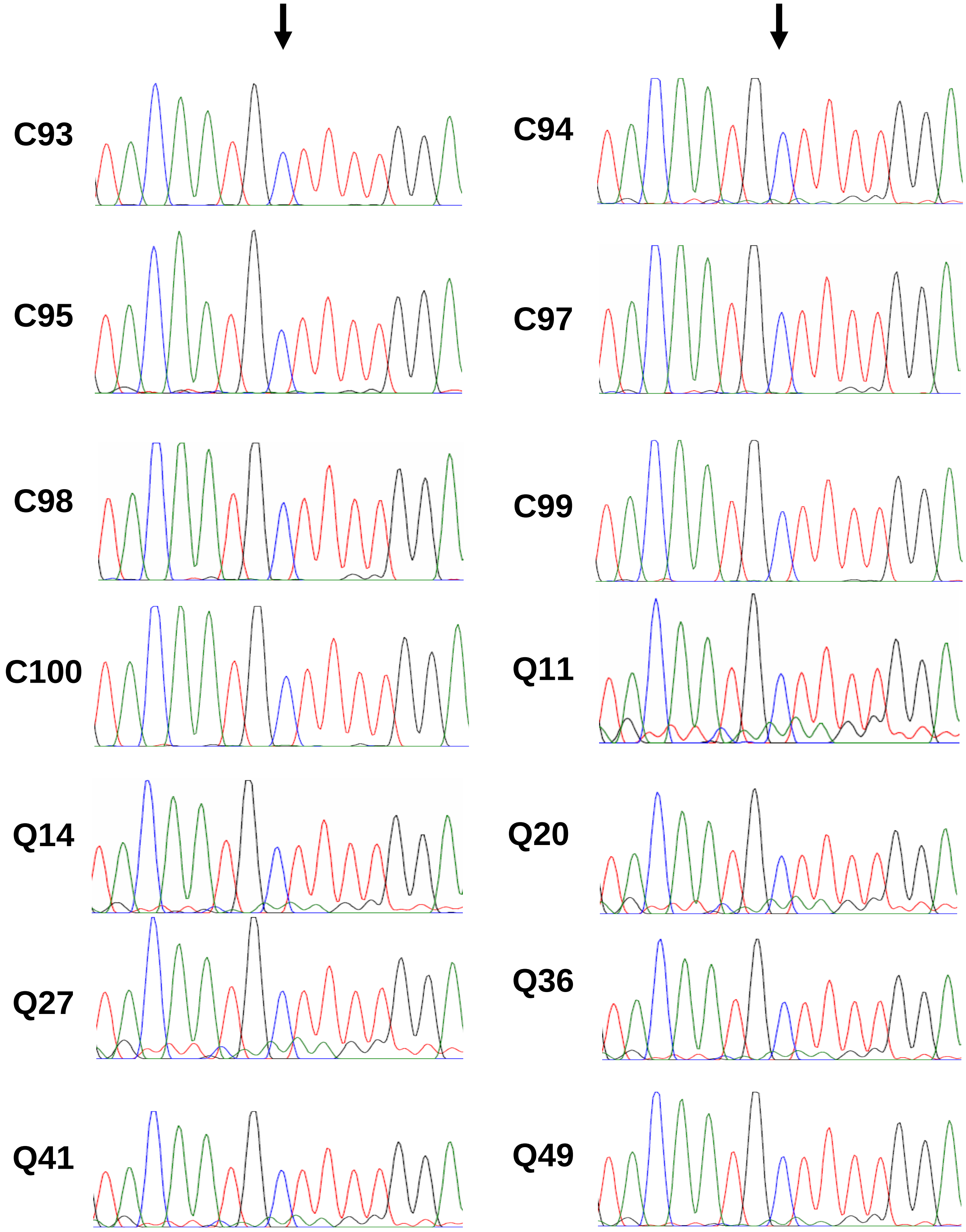

Controls for *USH3A*, c.368C>T (p.A123D)

Q51

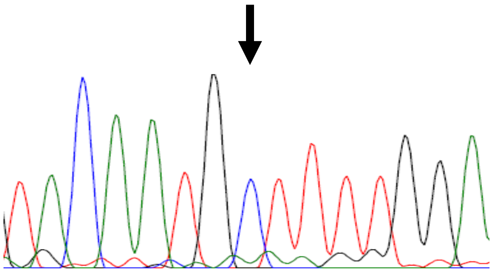

Q56

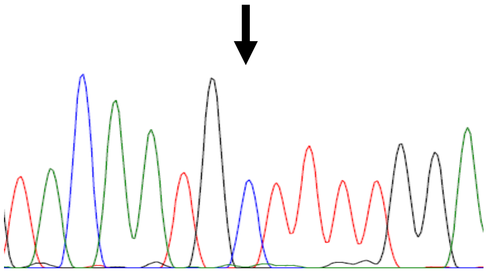

Supplement: Additional data file 6 — All mutations that have been identified in USH1 patients that have been investigated in this study (electropherograms), and figures for genotyping of healthy French Canadian control individuals for these mutations (by direct sequencing, restriction enzyme digest, and fragment length analysis). [file gb-2007-8-4-r47-S6.pdf]
