# Supplementary material for: Deafblindness in French Canadians from Quebec: a predominant founder mutation in the USH1C gene provides the first genetic link with the Acadian population
Source: Genome Biol. 2007 Apr 3;8(4):R47. doi: 10.1186/gb-2007-8-4-r47 (PMC1895989; doi:10.1186/gb-2007-8-4-r47)

Deafblindness in French Canadians from Quebec: A predominant founder mutation in the *USH1C* gene provides the first genetic link with the Acadian population

Ebermann et al.

RAW DATA

	page
Haplotypes	
USH1C haplotypes	2 – 23
USH1D haplotypes	24 – 29
USH1 mutations	30 – 36
Genotyping of healthy French Canadian Controls	
Controls for c.216G>A (*USH1C*)	37 – 40
Controls for c.238-239insC (*USH1C*)	41 – 58
Controls for c.496+1G>T (*USH1C*)	59 – 60
Controls for p.R155X (*USH1C*)	61 – 62
Controls for c.748-759+5del (*USH1C*)	63 – 64
Controls for IVS45-9G>A (*CDH23*)	65 – 73
Controls for p.R736X (*CDH23*)	74 – 82
Controls for p.A457V (*MYO7A*)	83 – 91
Controls for p.Q815X (*MYO7A*)	92 – 93
Controls for p.A123D (*USH3A*)	94 – 102
Mutation screening in USH1 genes in patient 1881	103 – 204

Mutation screening in USH1 genes in patient 1881.

Patient 1881, *MYO7A* (*USH1B*)

Exon 2

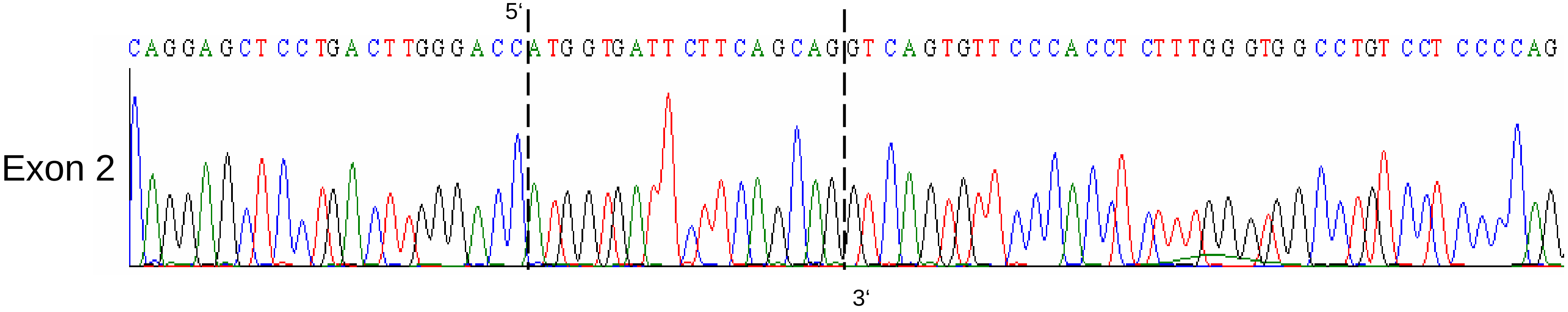

Exon 3

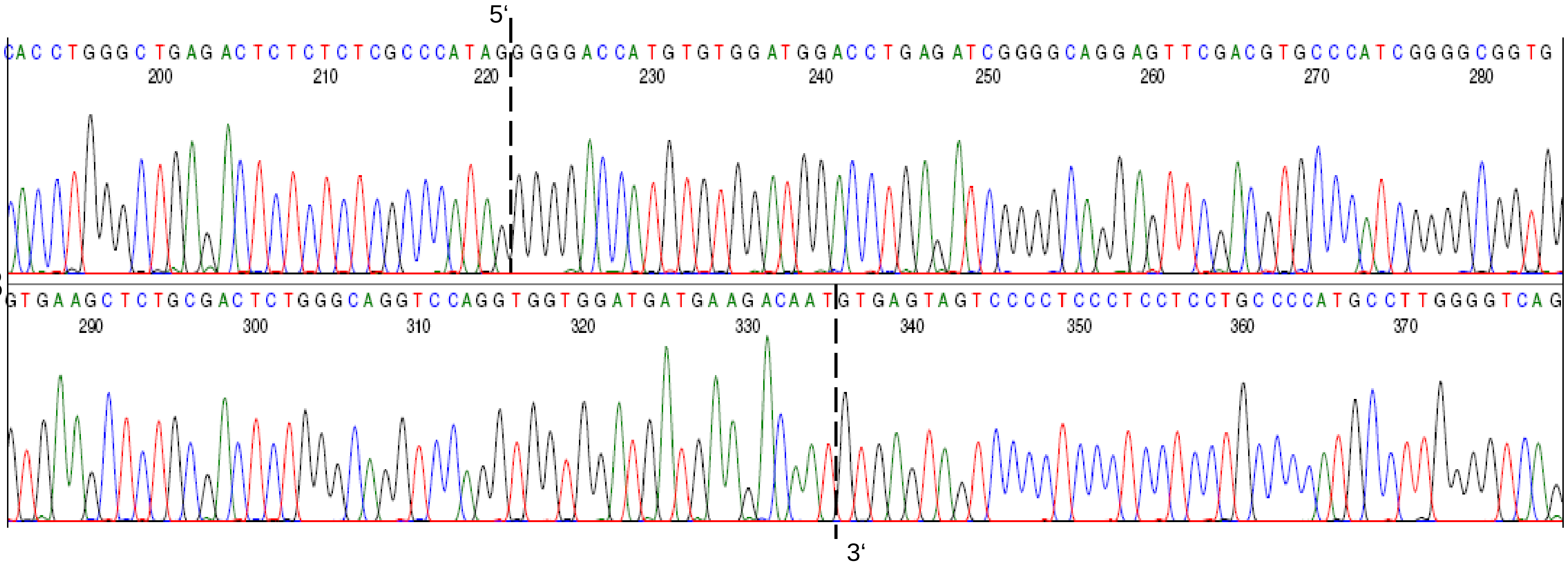

Exon 4

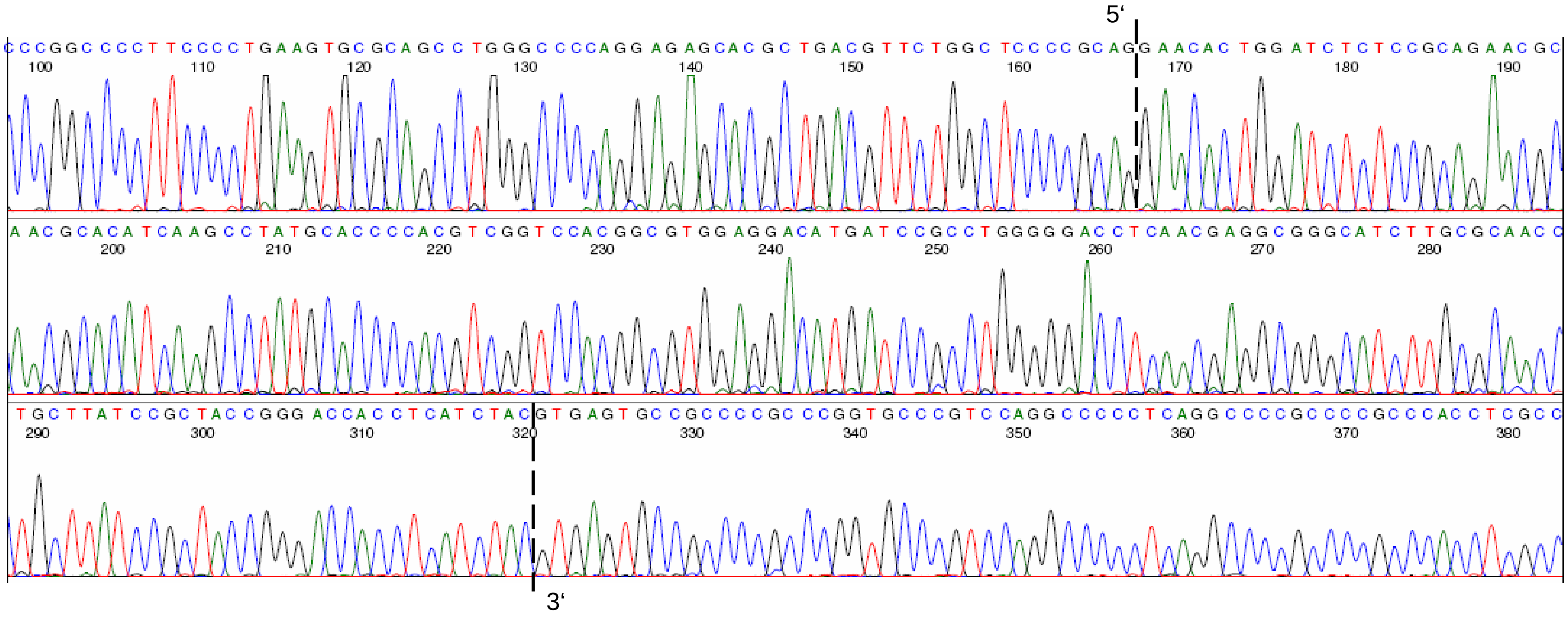

Exon 5

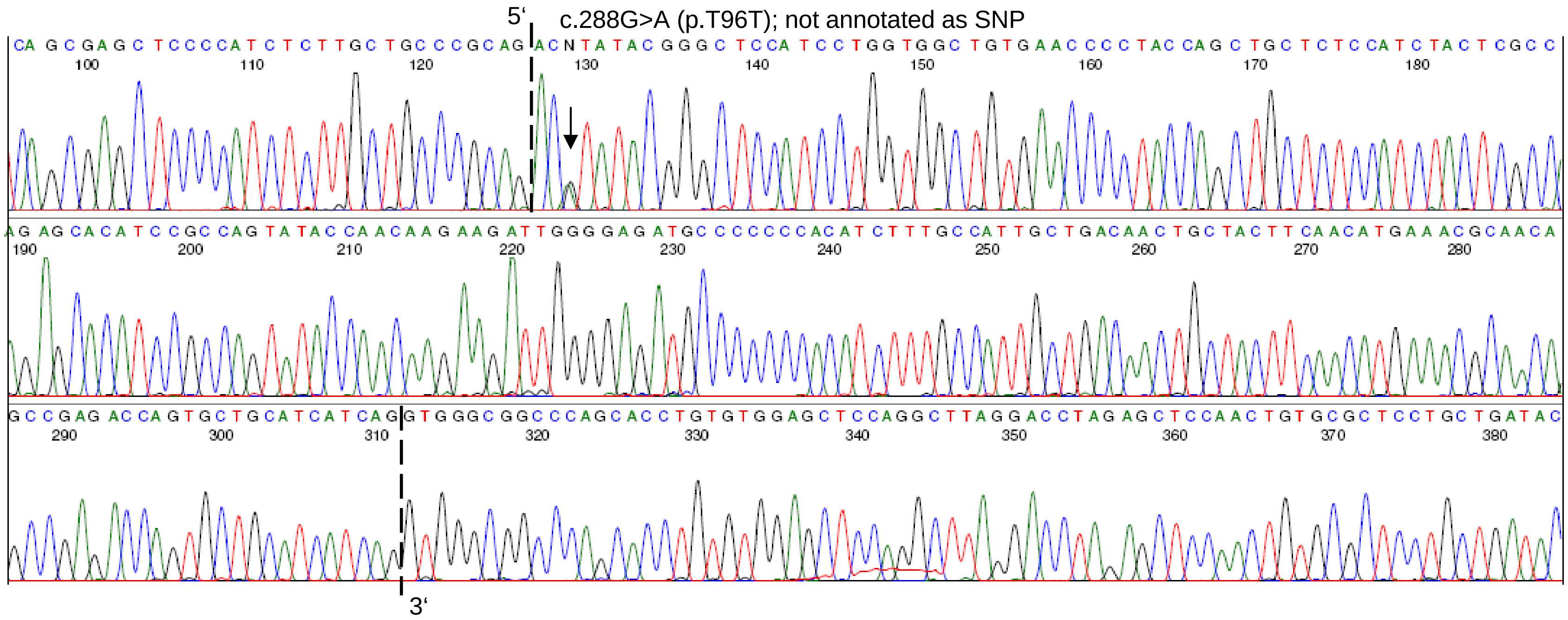

Exon 6

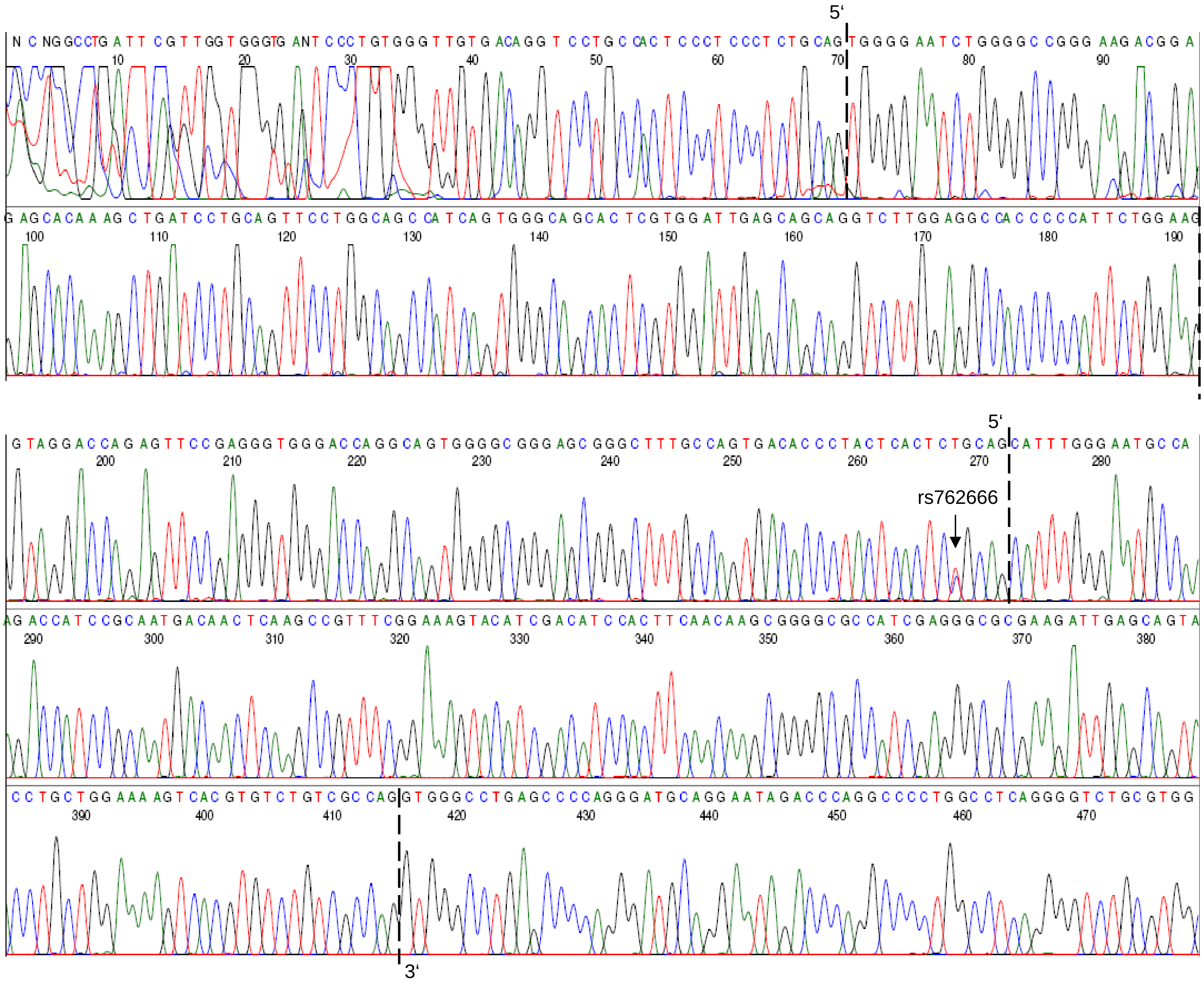

Exon 7

Exon 8

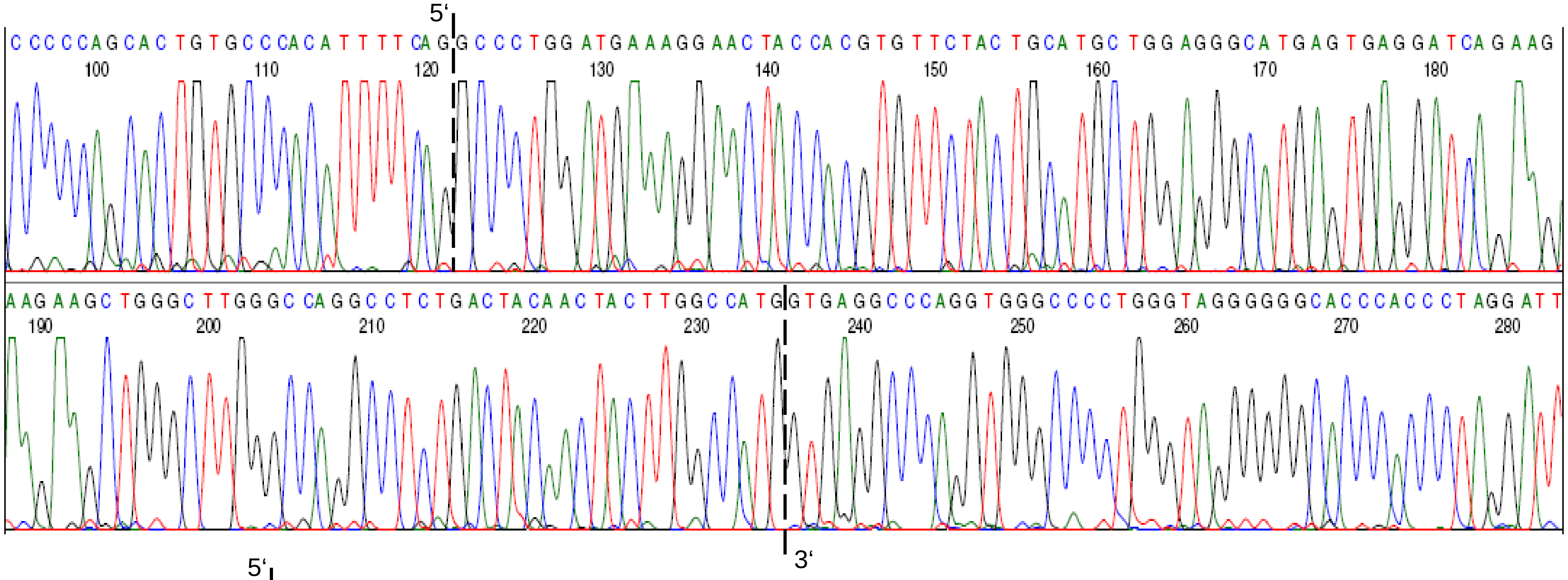

Exon 9

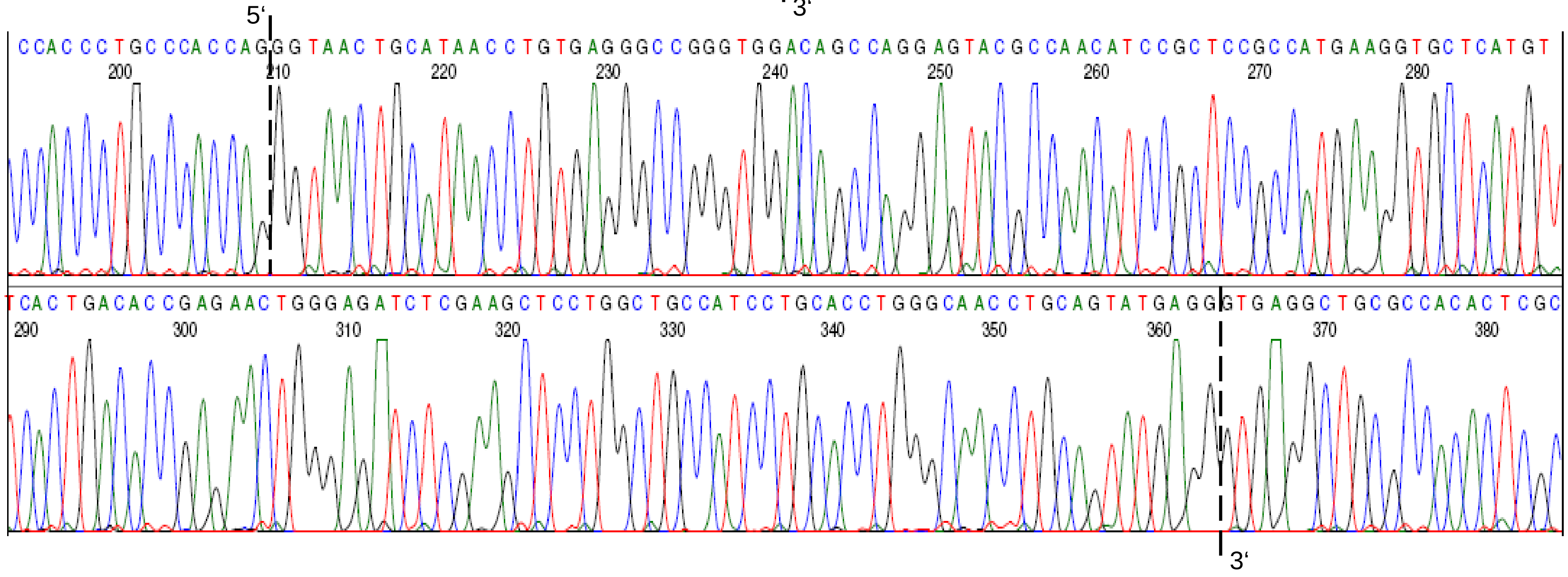

Exon 10

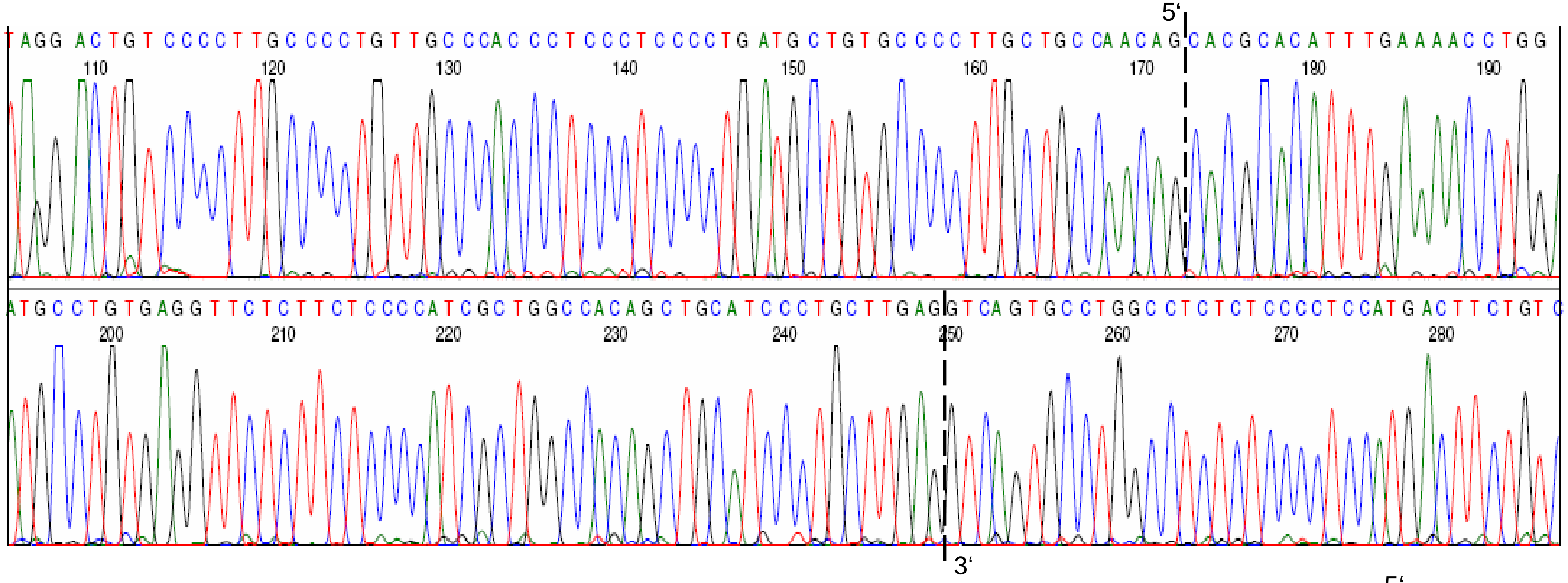

Exon 11

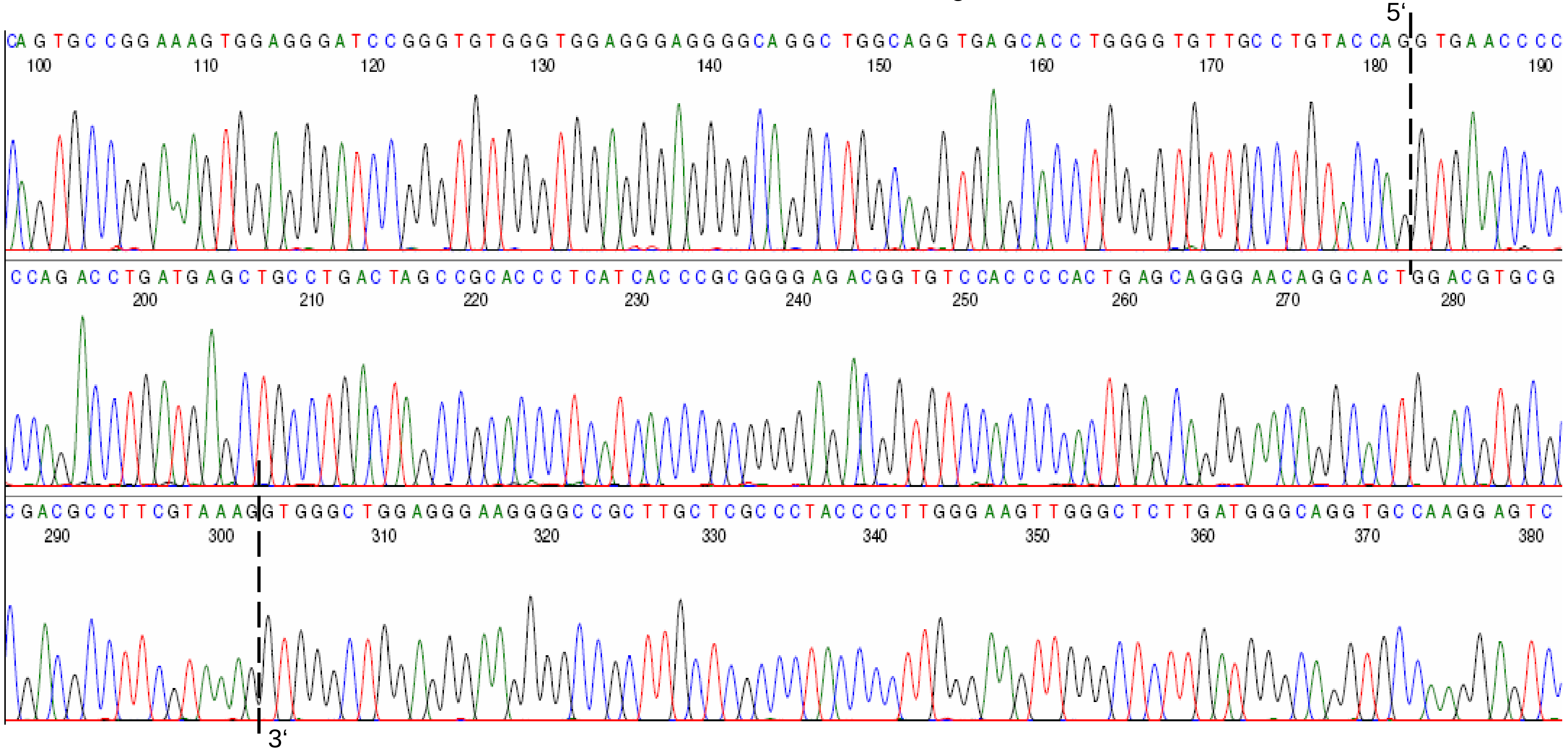

Exon 12

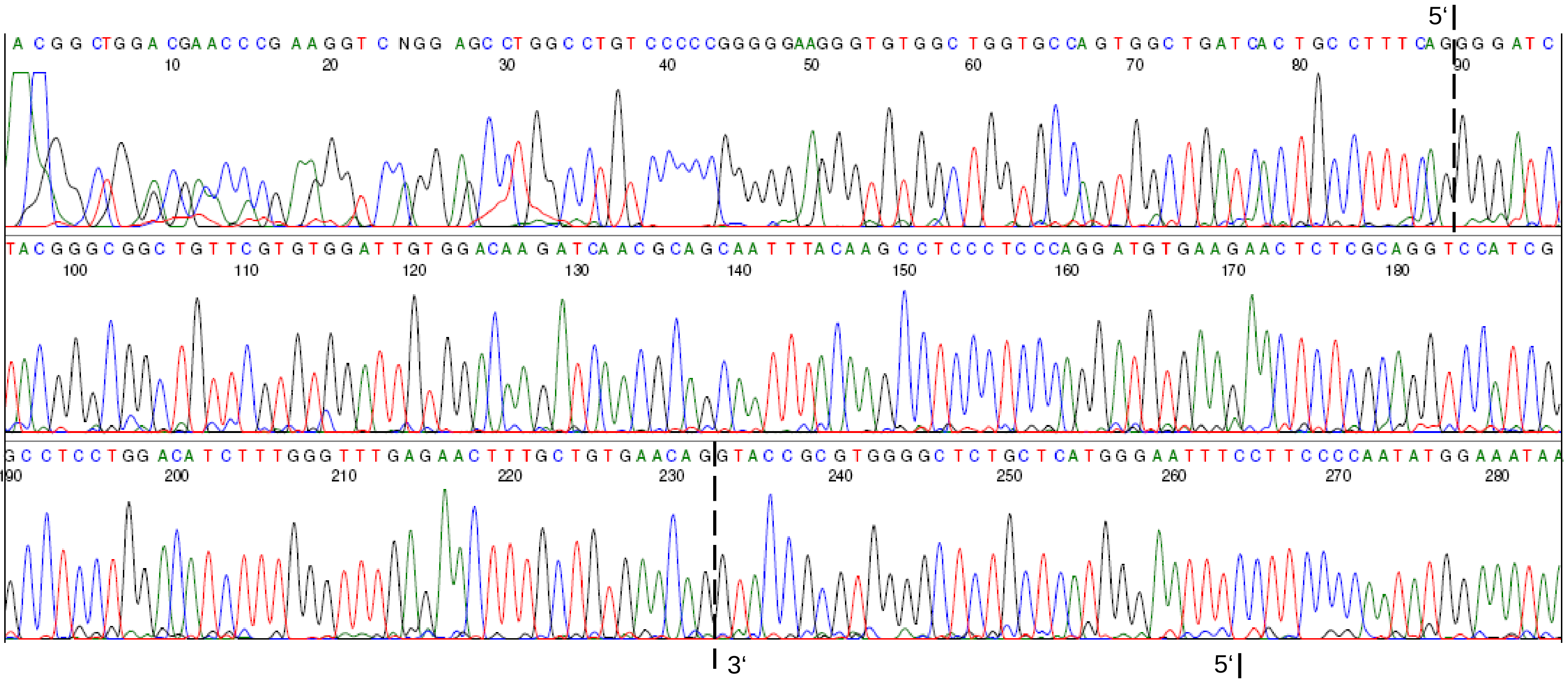

Exon 13

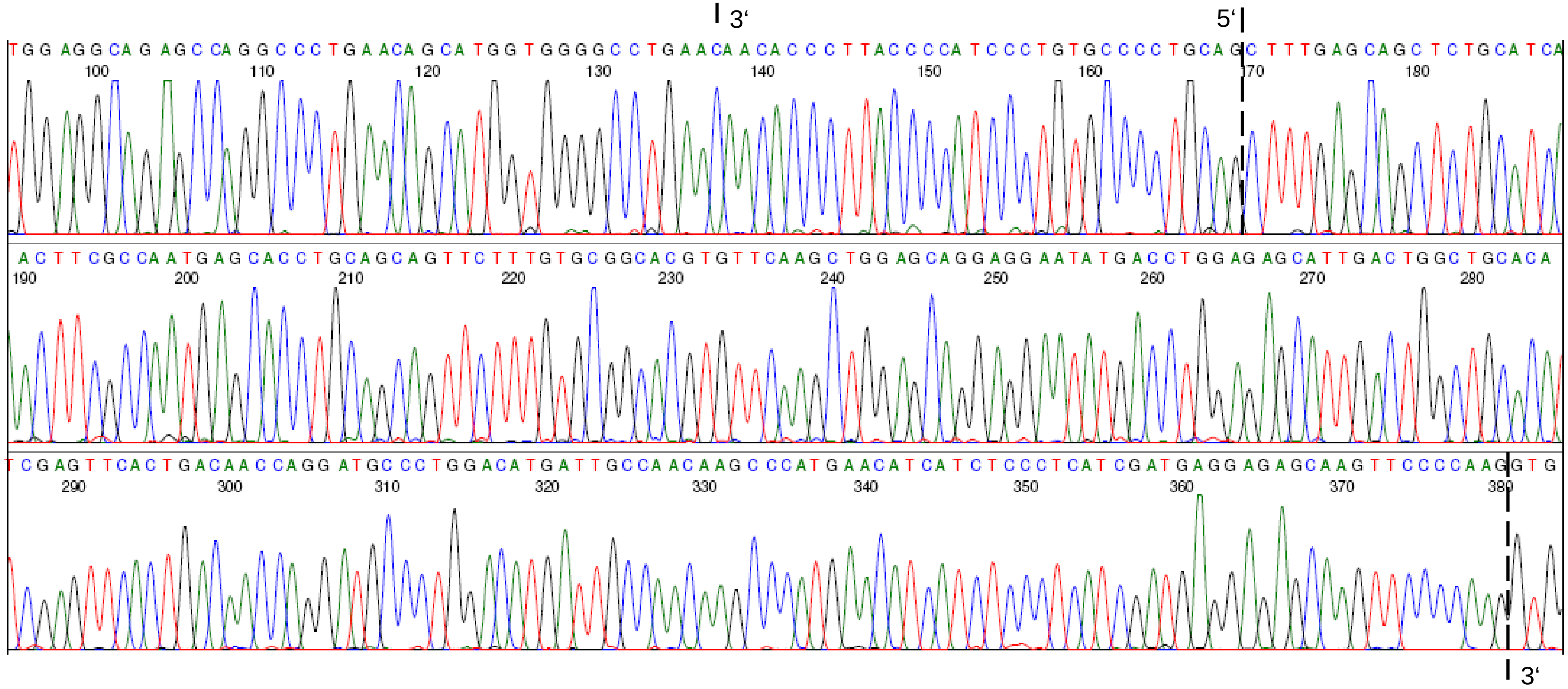

Exon 14

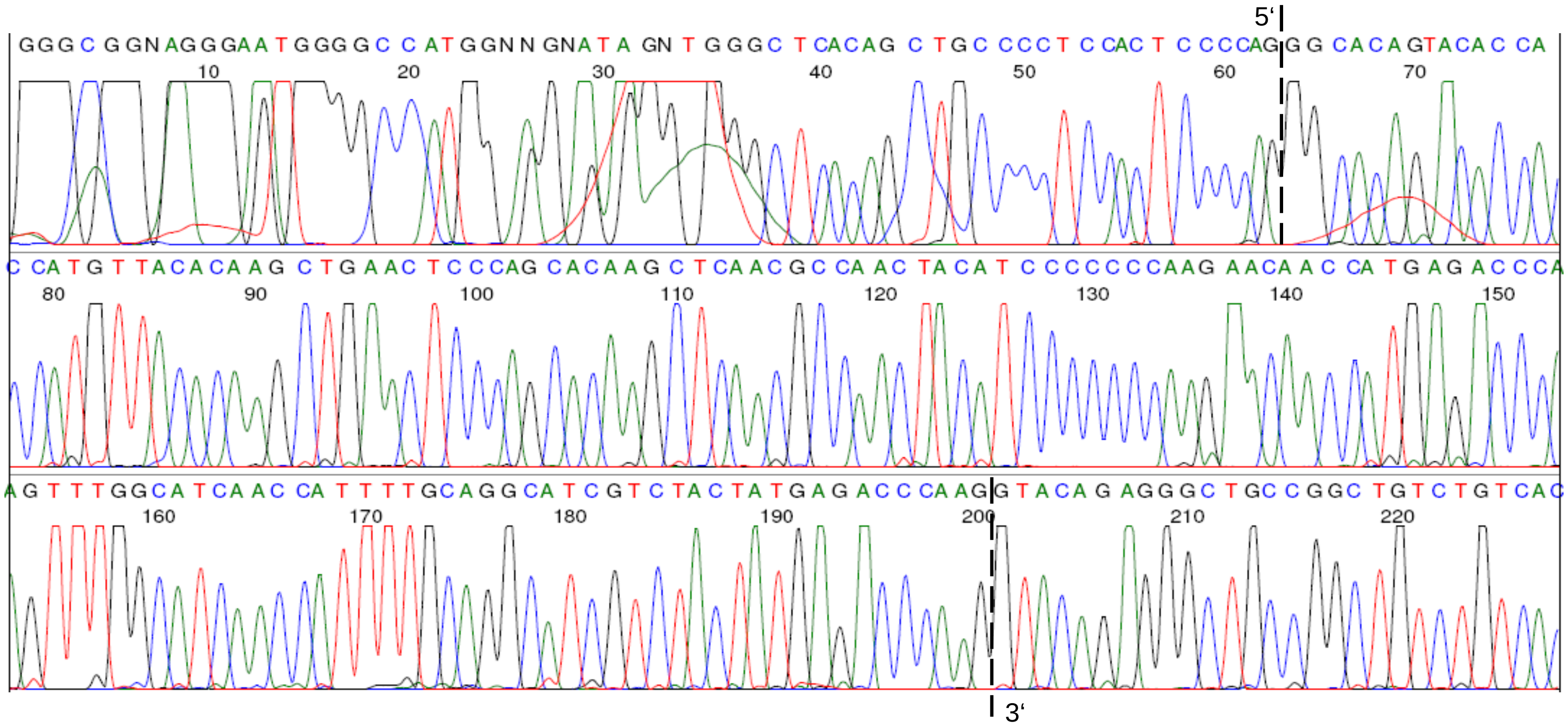

Exon 15

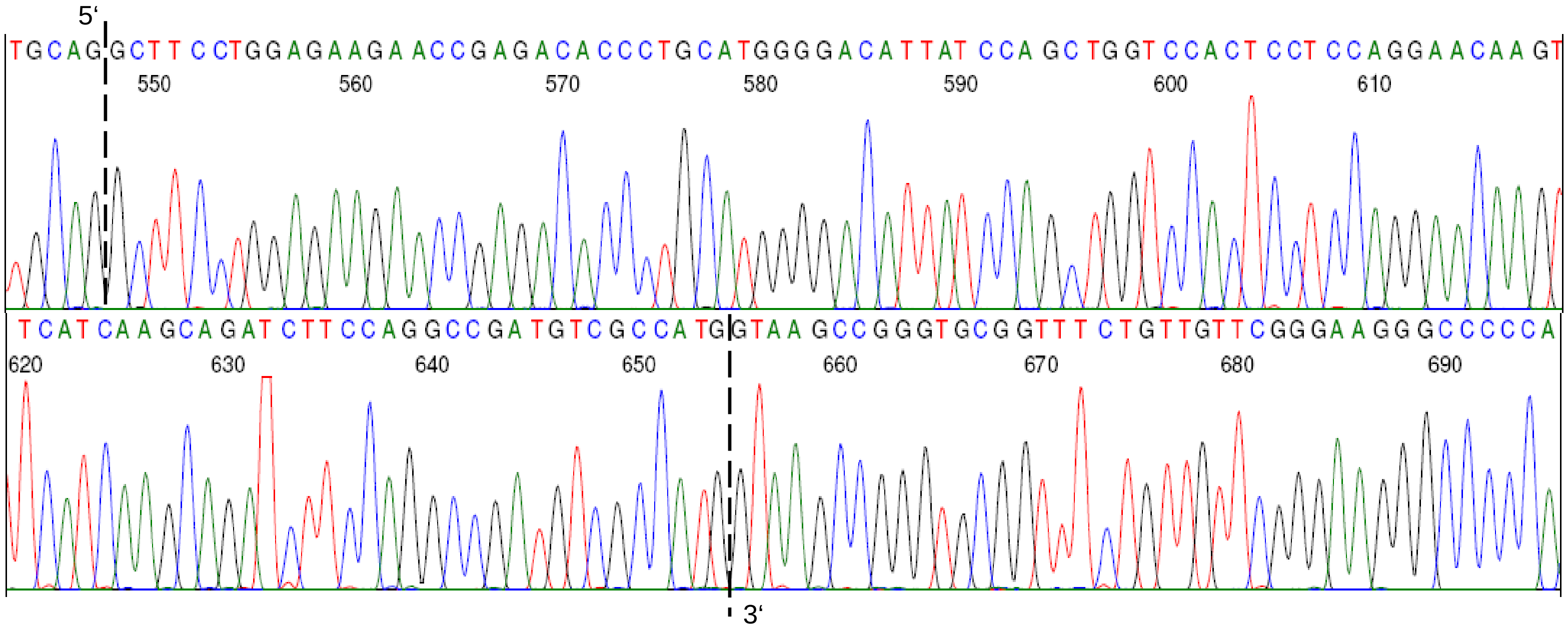

Exon 16

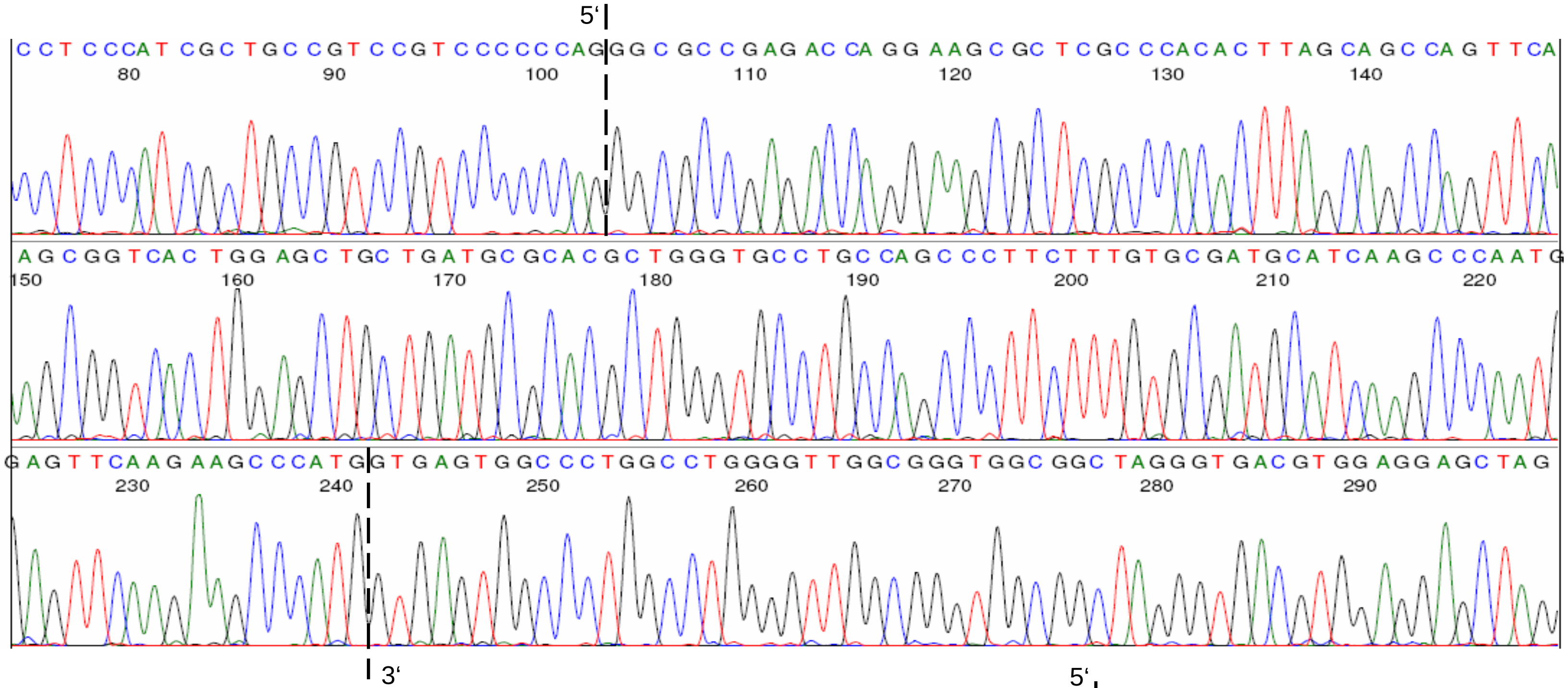

Exon 17

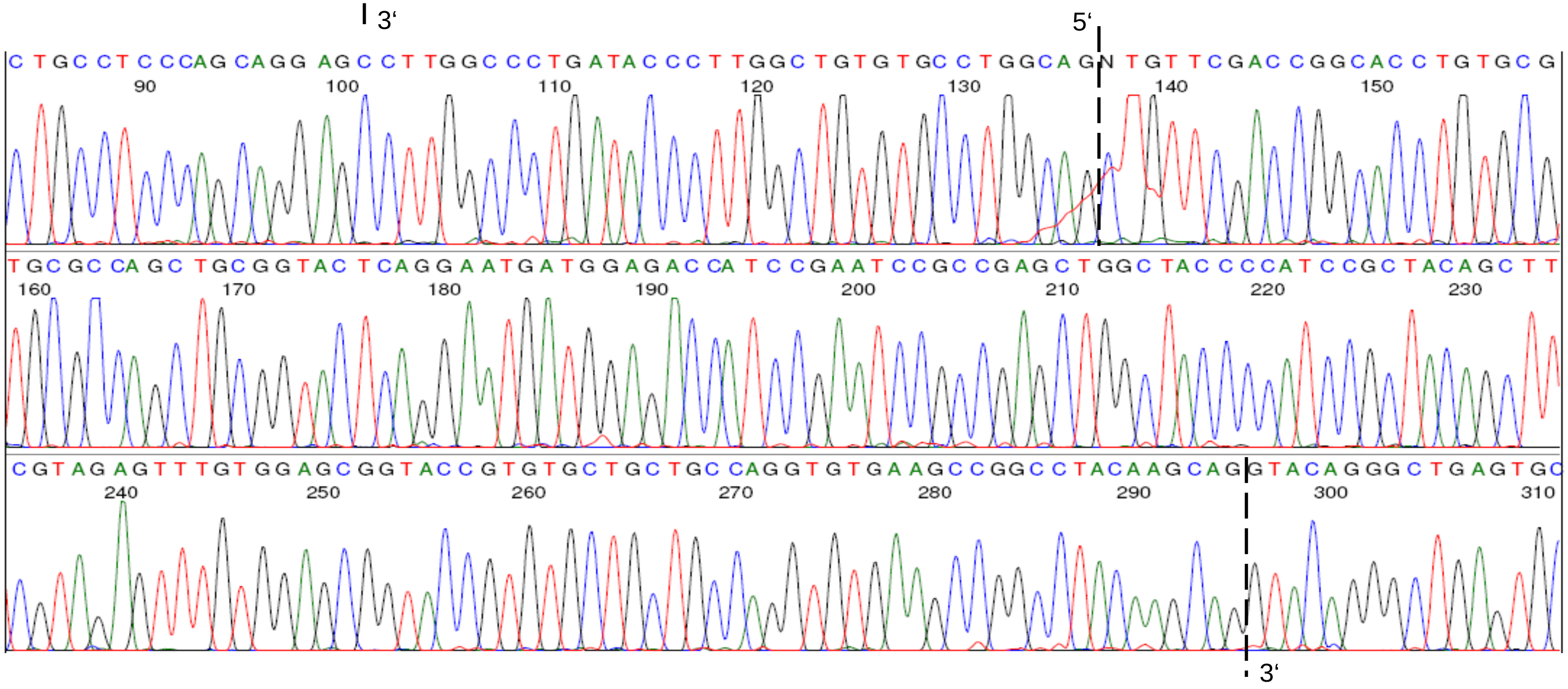

Exon 18

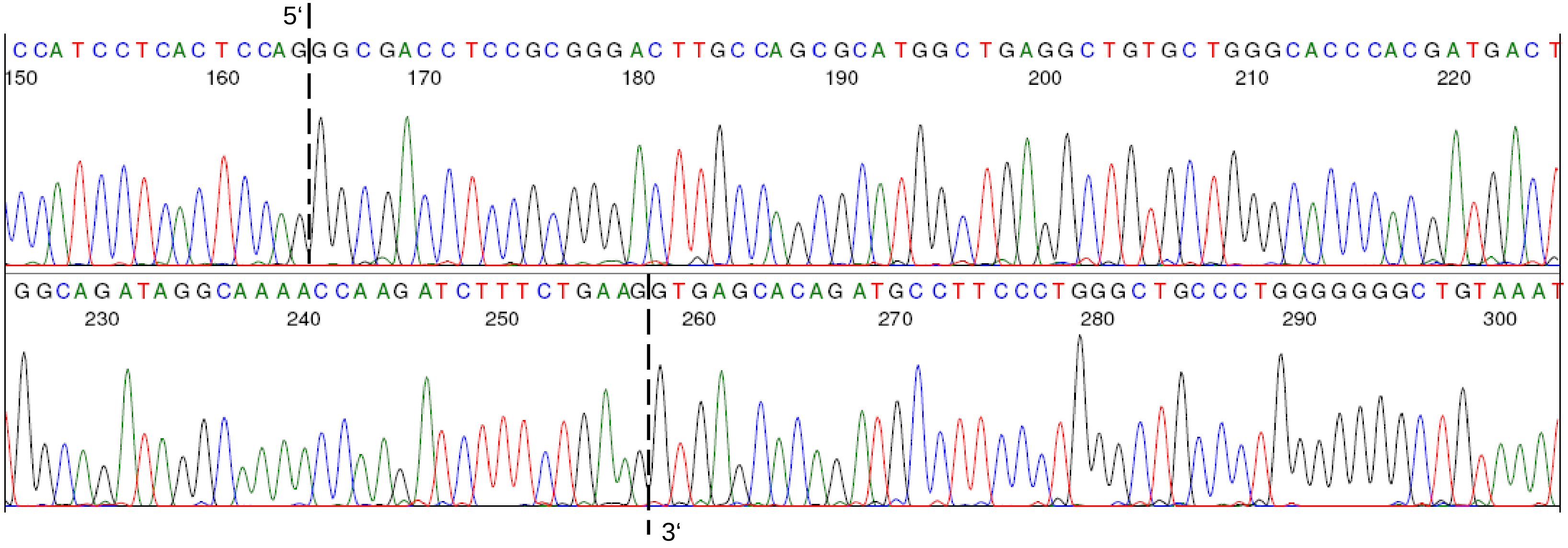

Exon 19

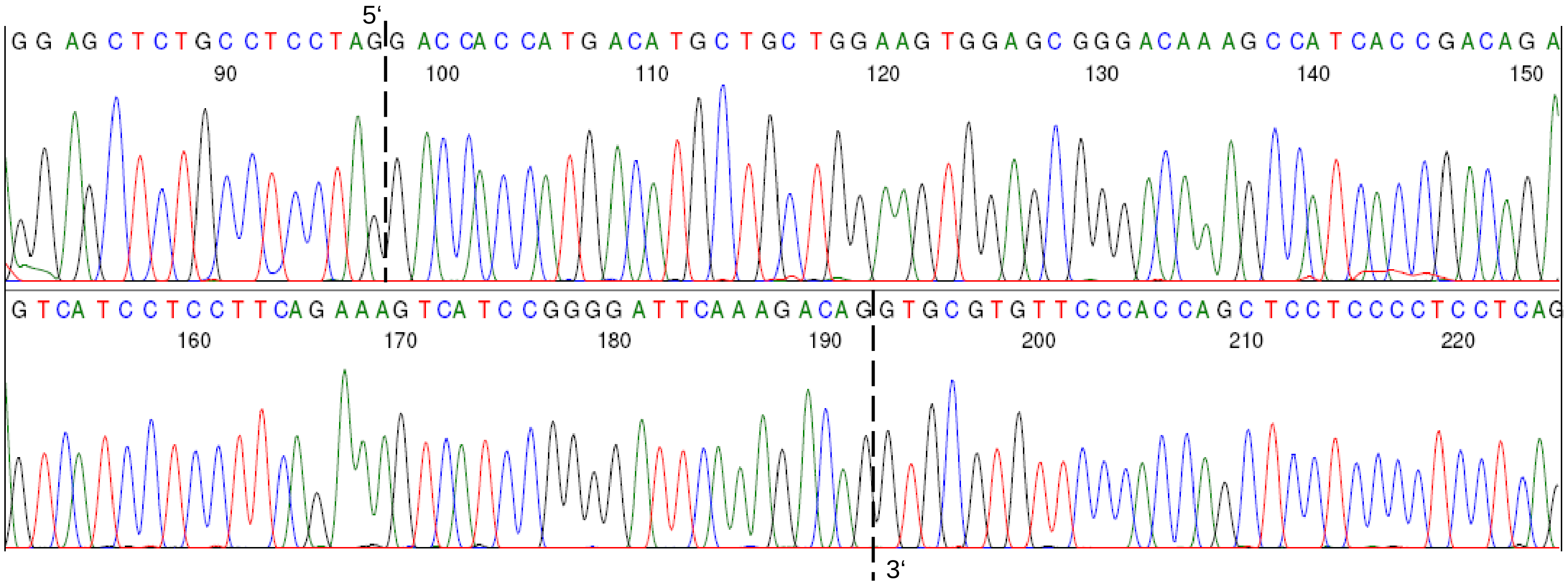

Exon 20

Exon 21

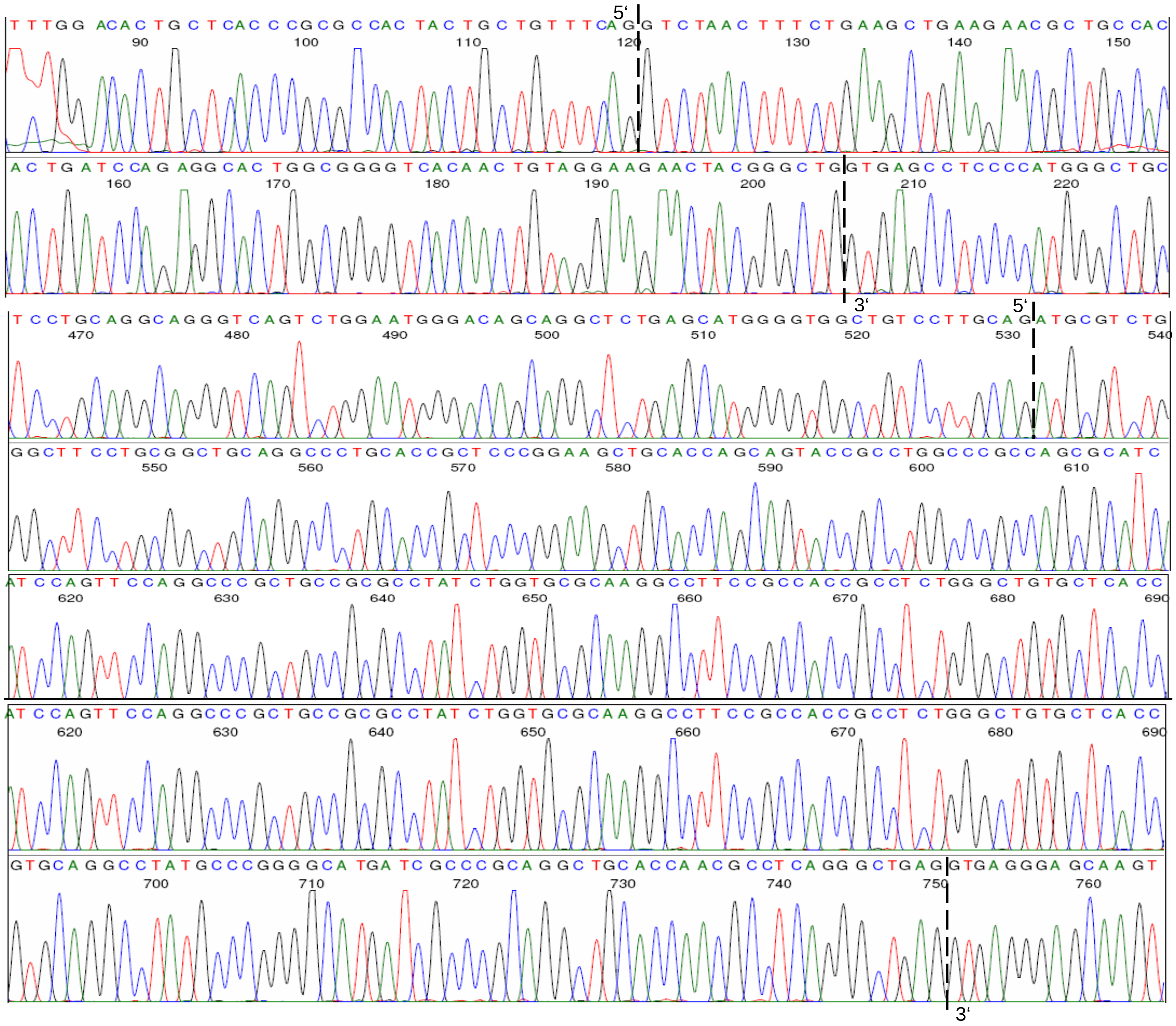

Exon 22

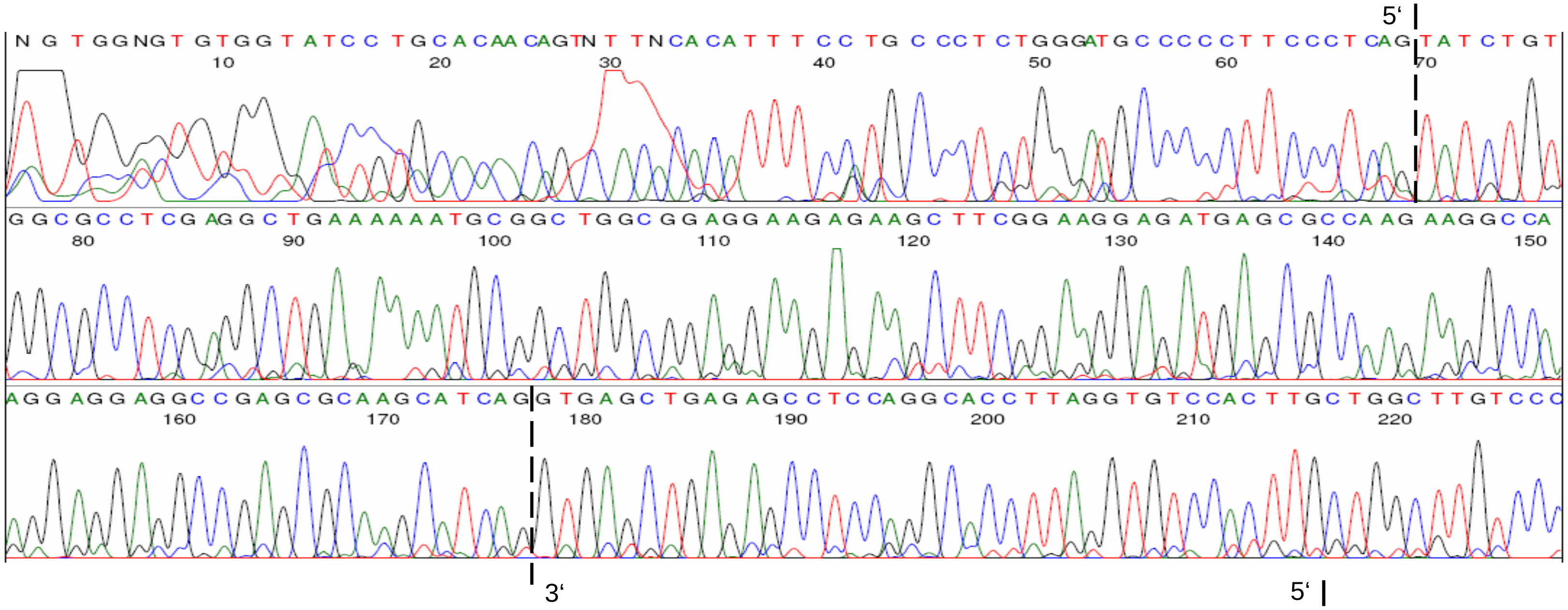

Exon 23

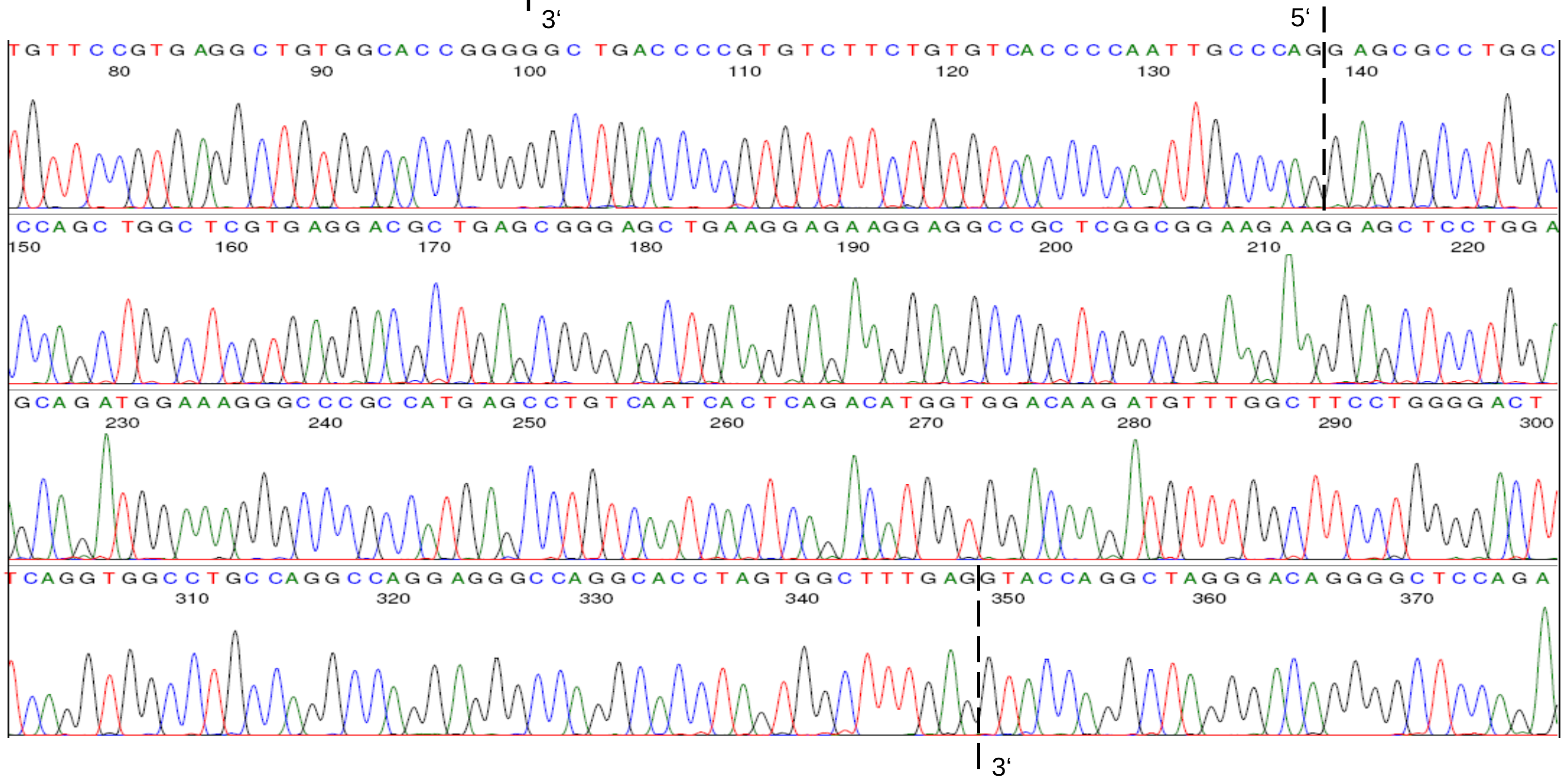

Exon 24

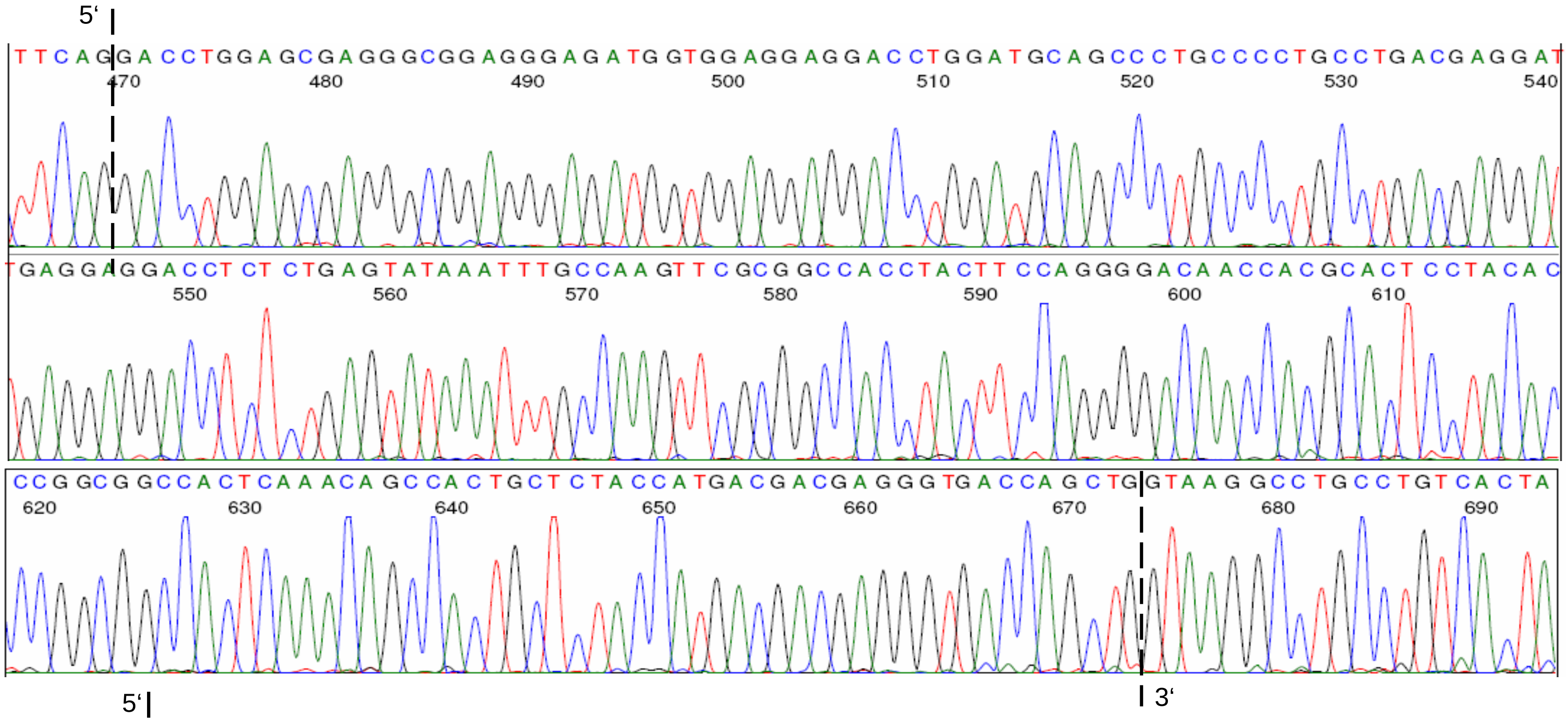

Exon 25

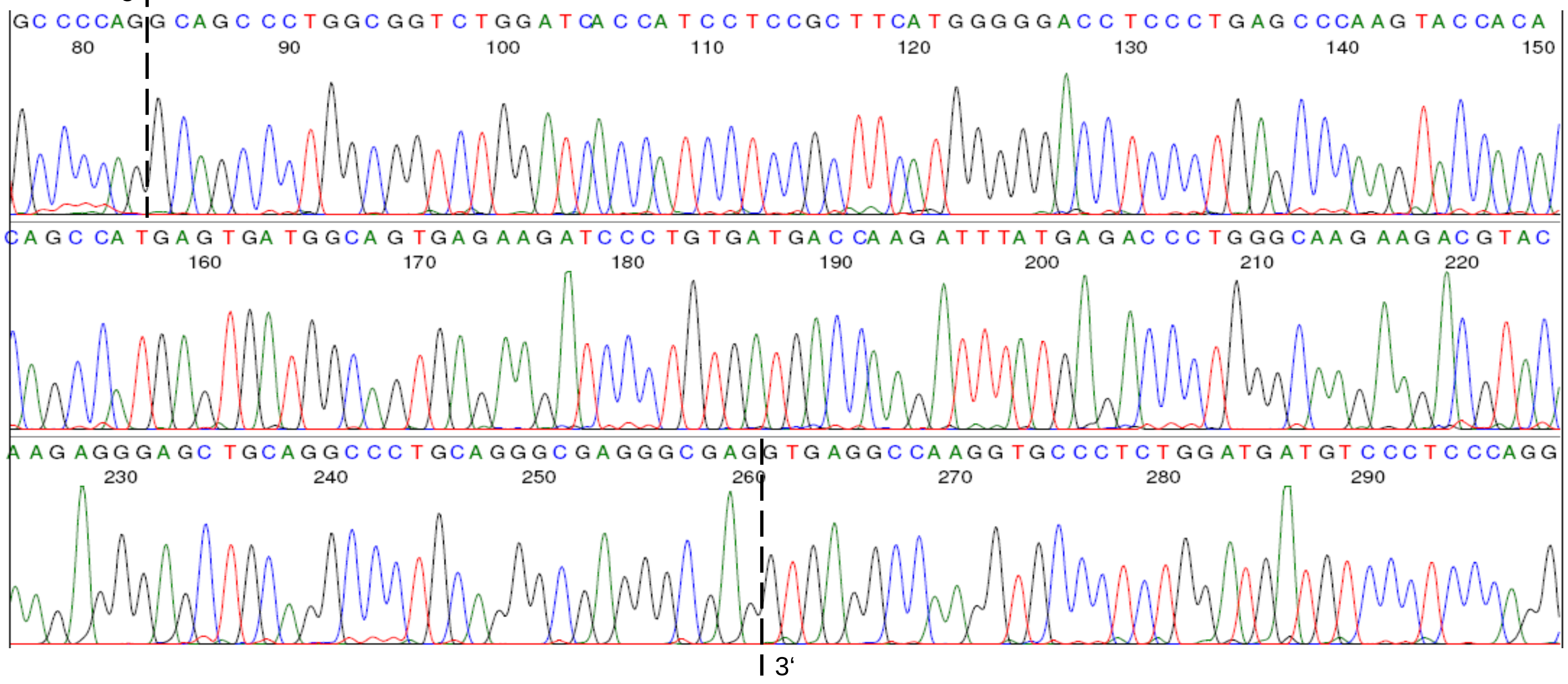

Exon 26

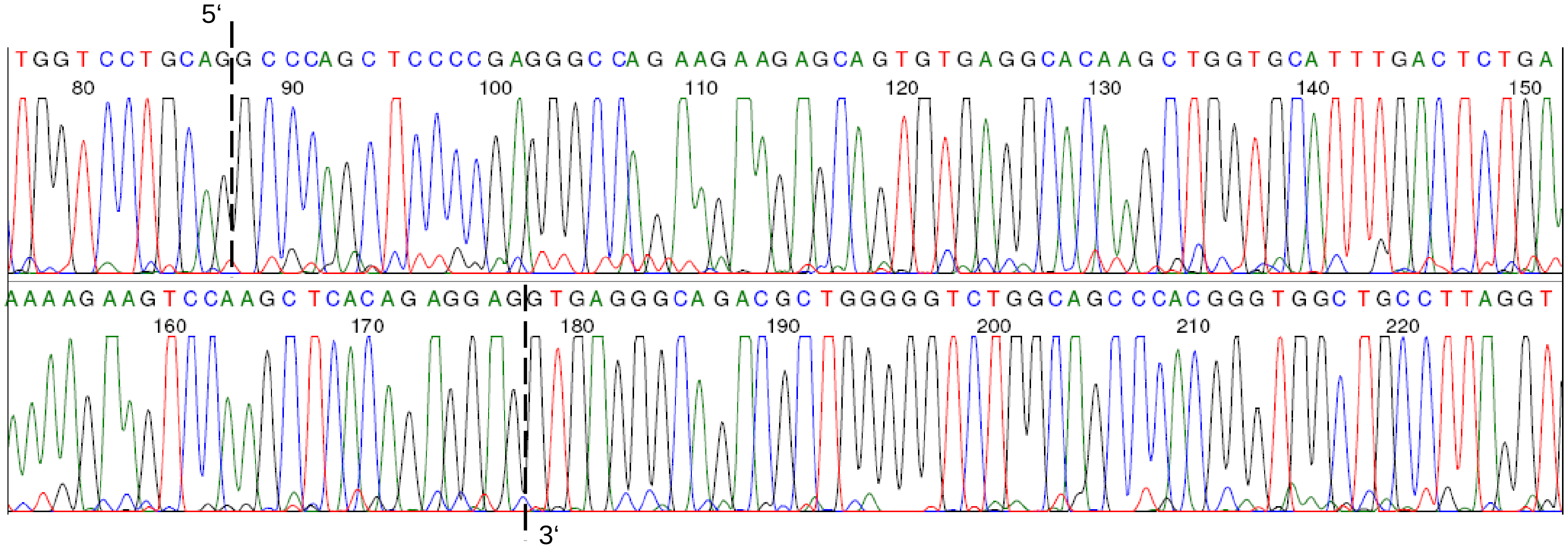

Exon 27

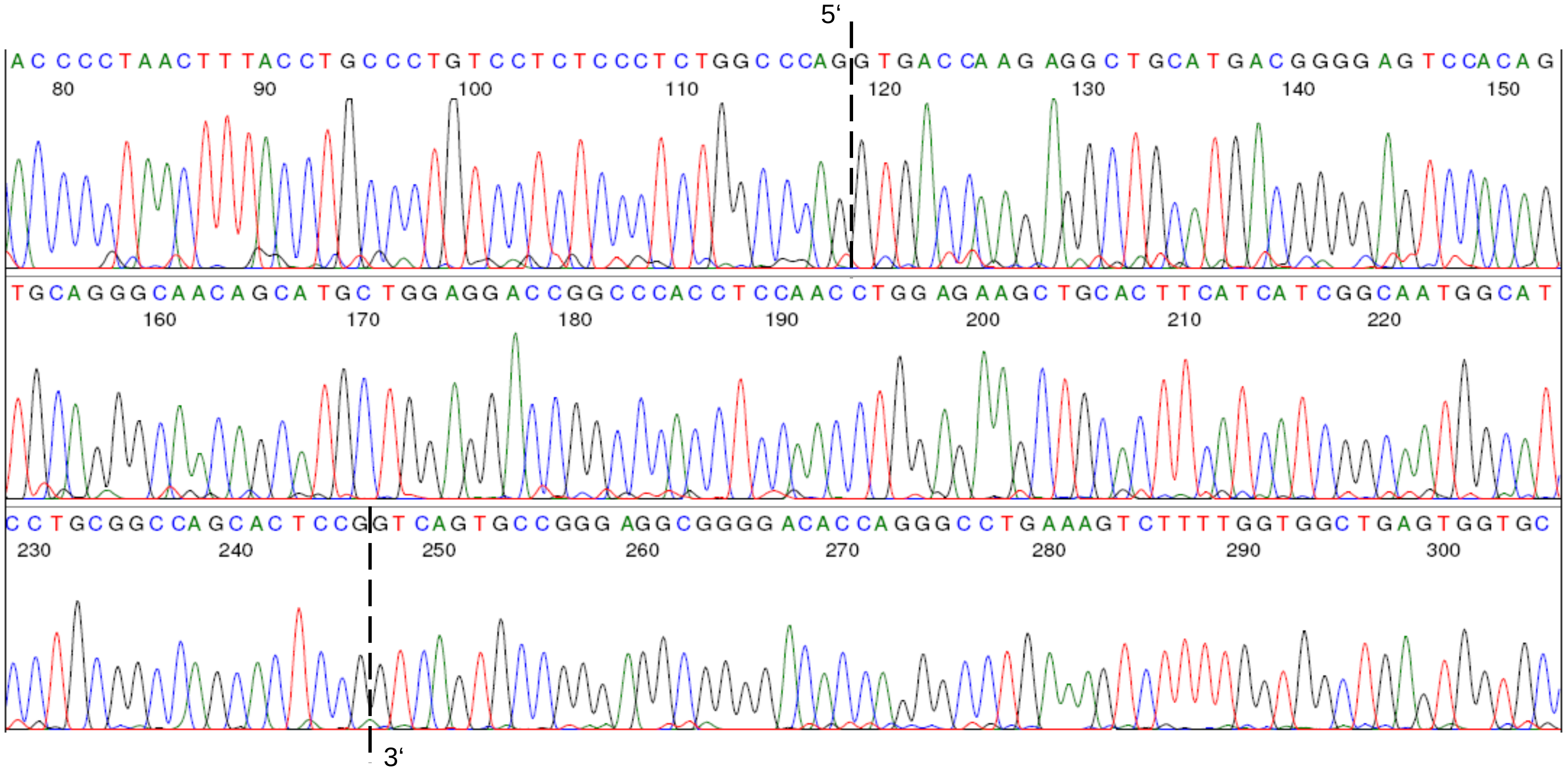

Exon 28

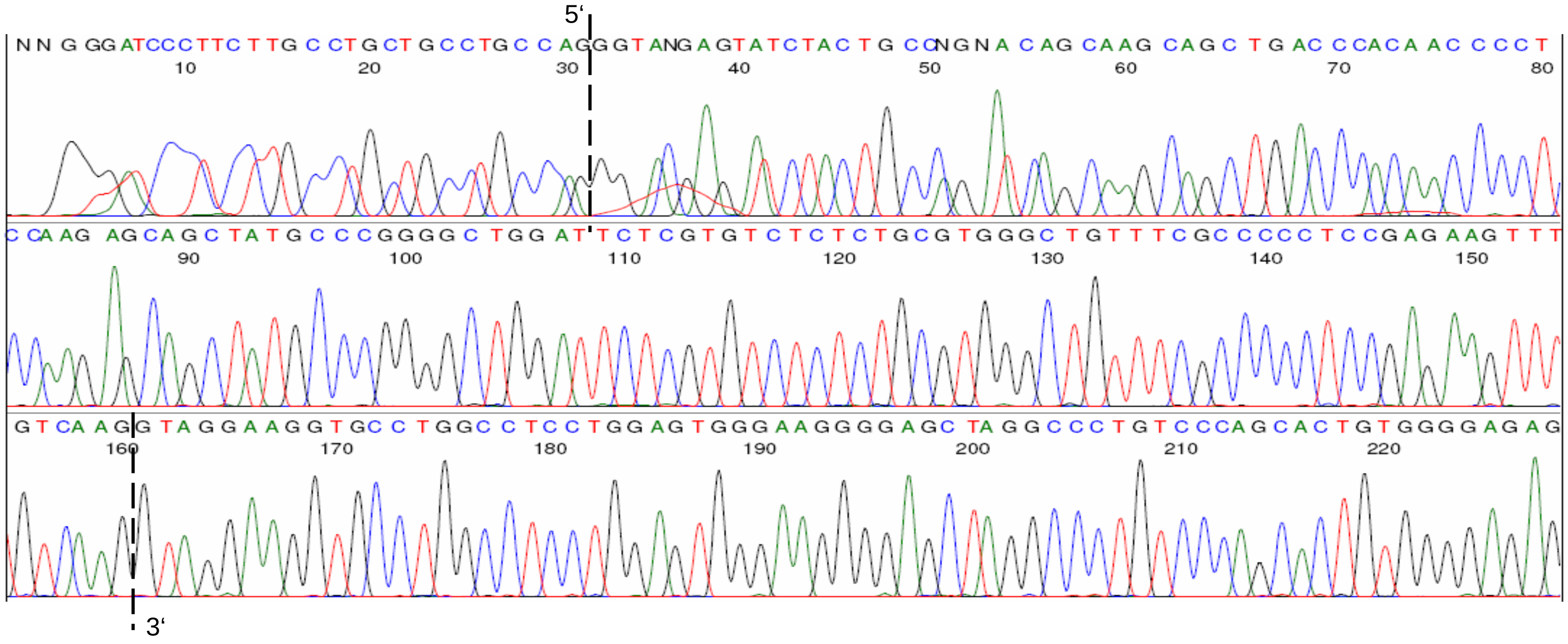

Exon 29

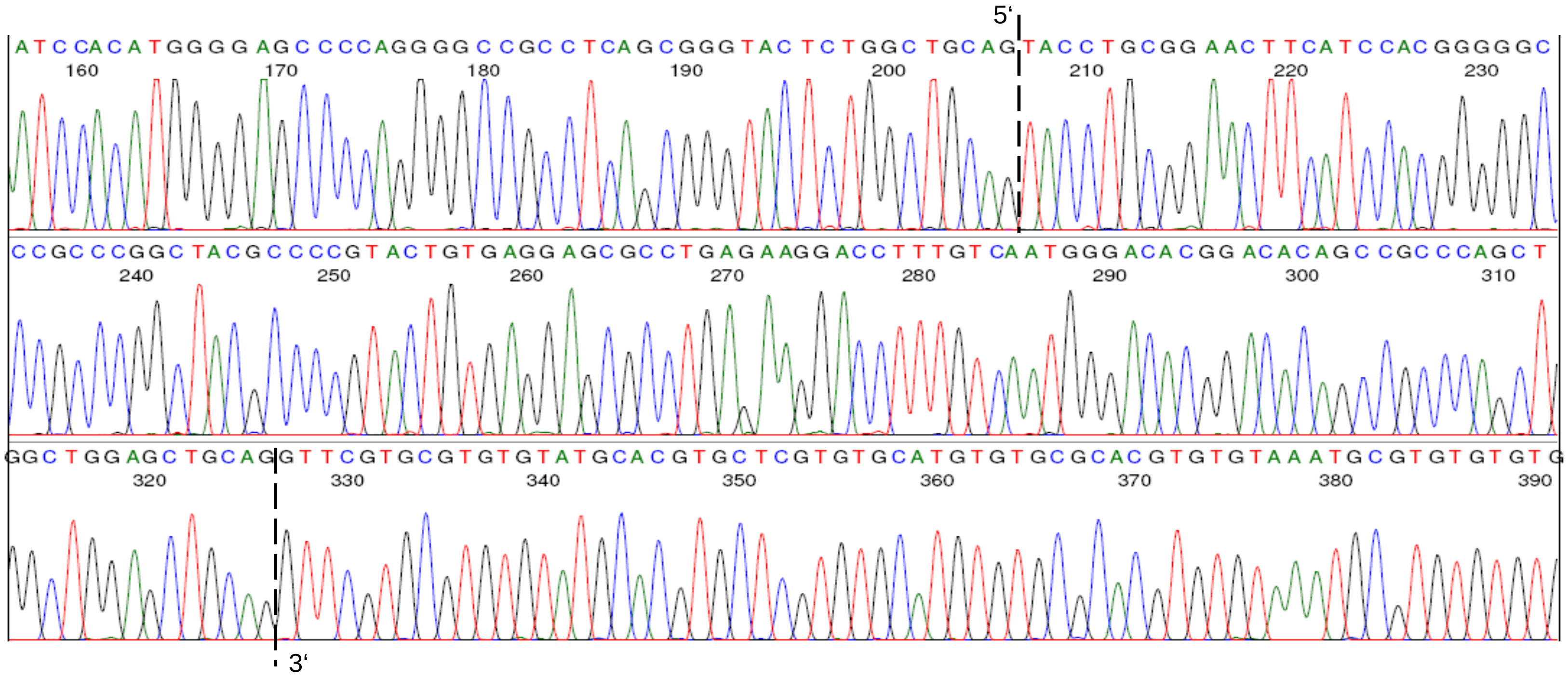

Exon 30

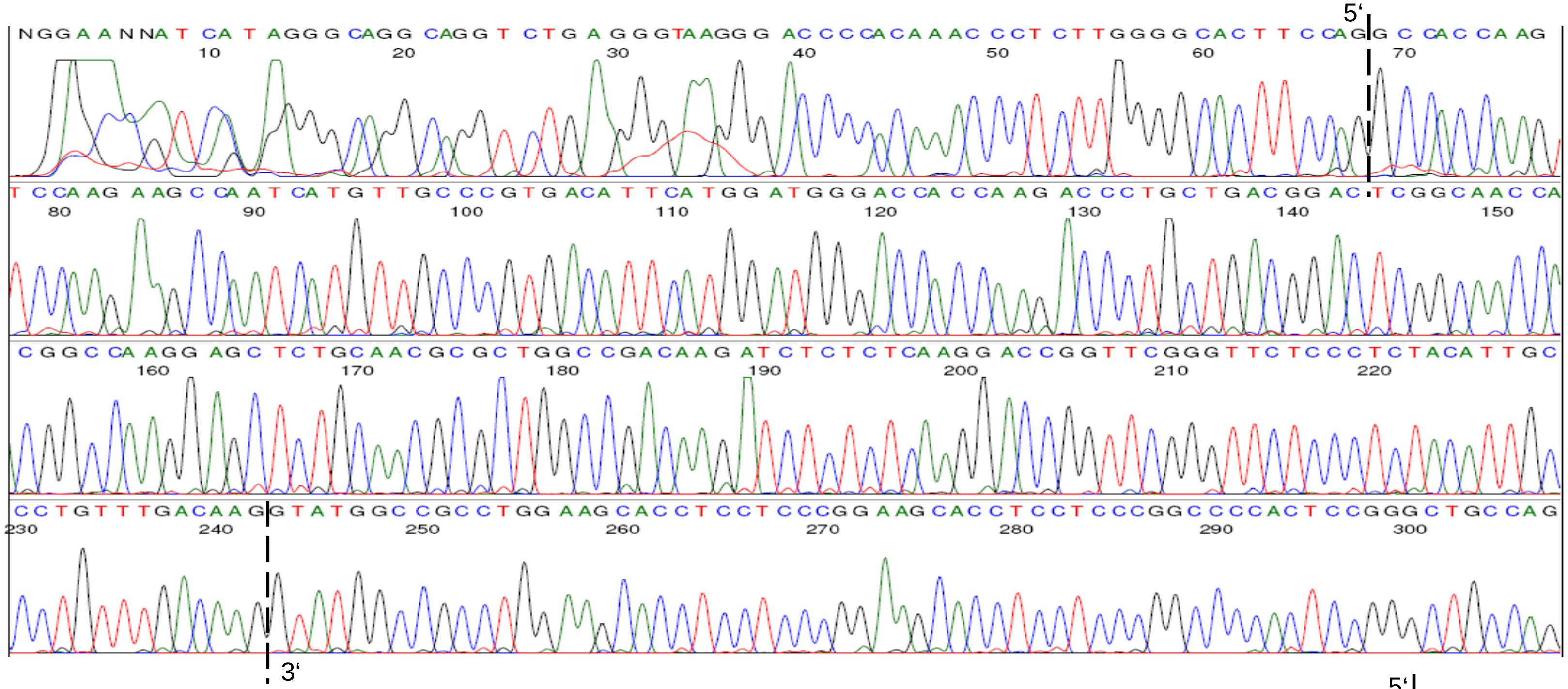

Exon 31

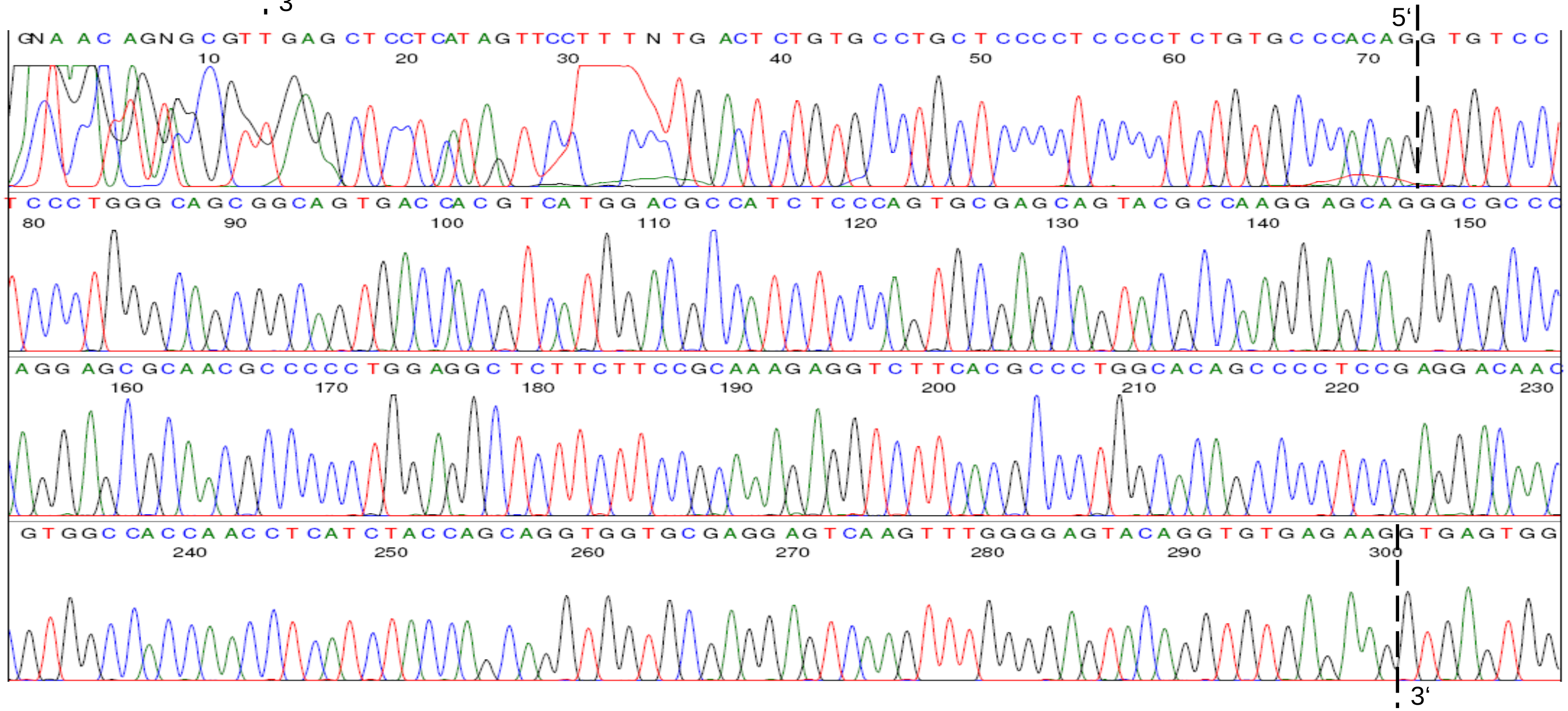

Exon 32

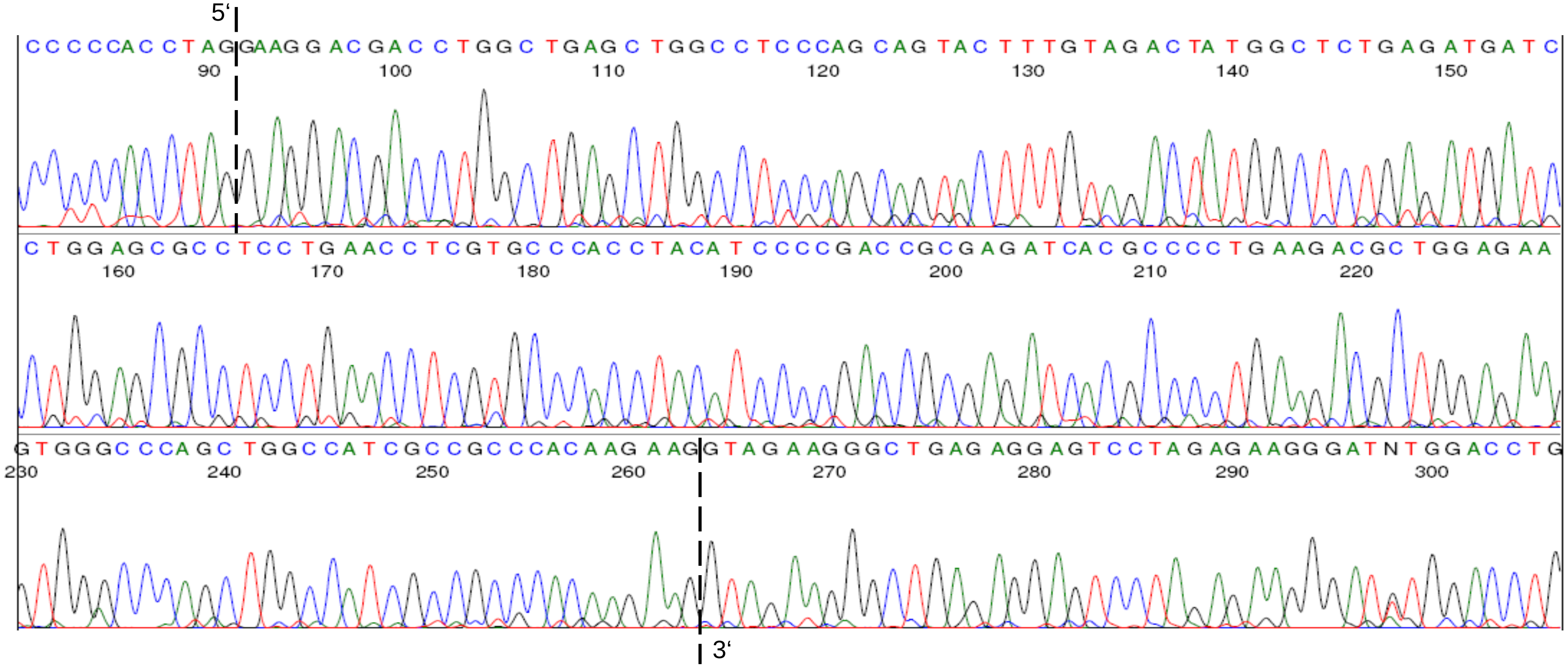

Exon 33

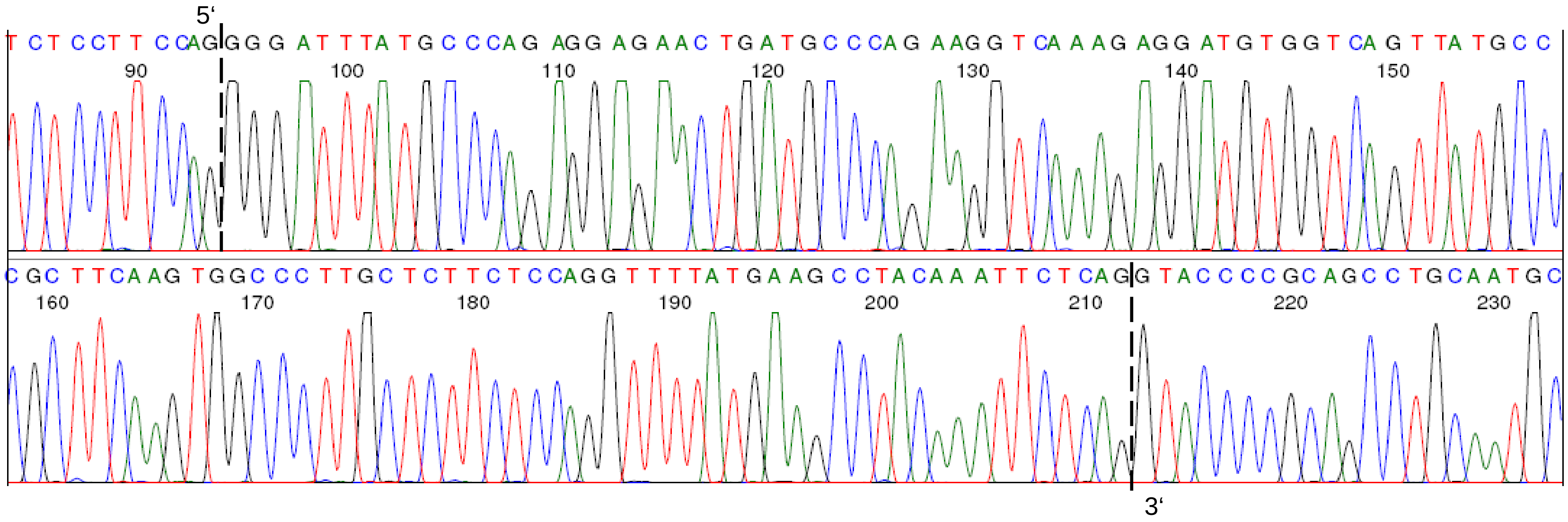

Exon 34

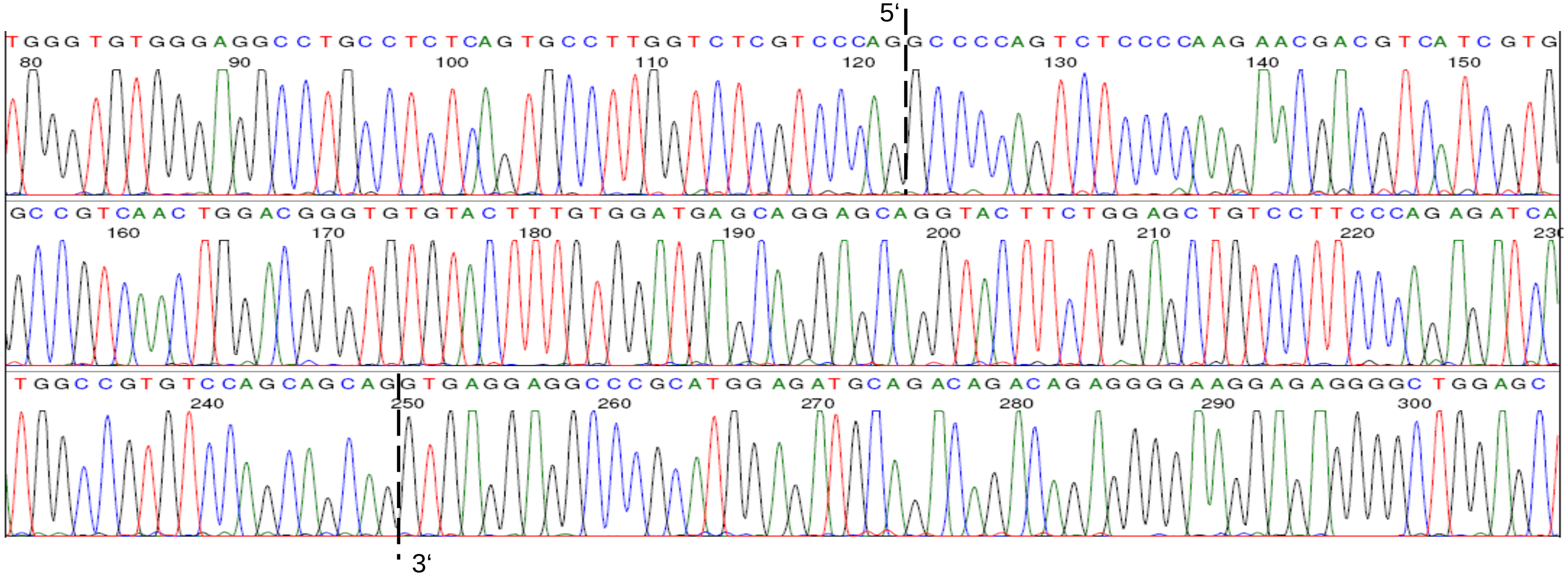

Exon 35

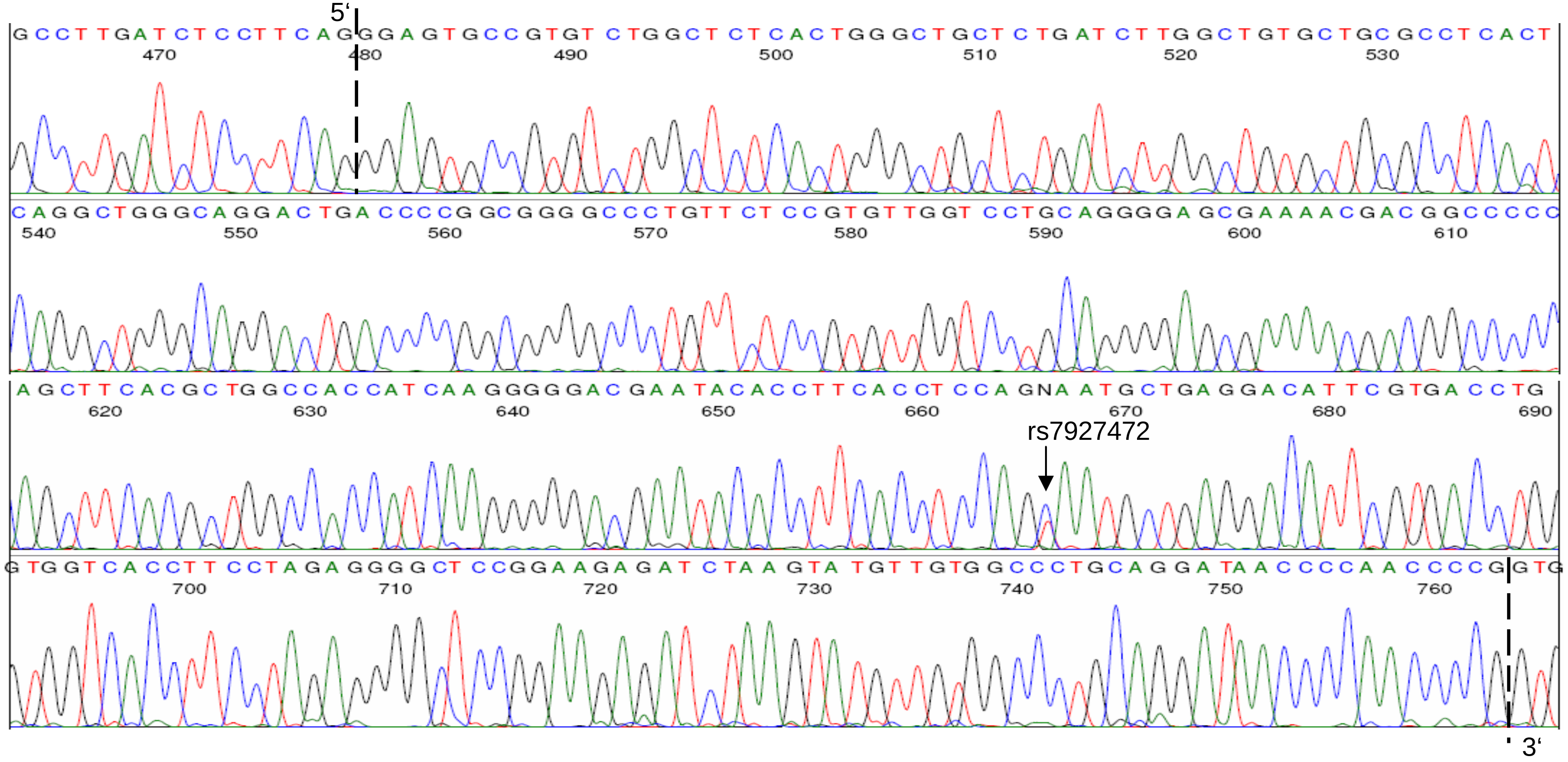

Exon 36

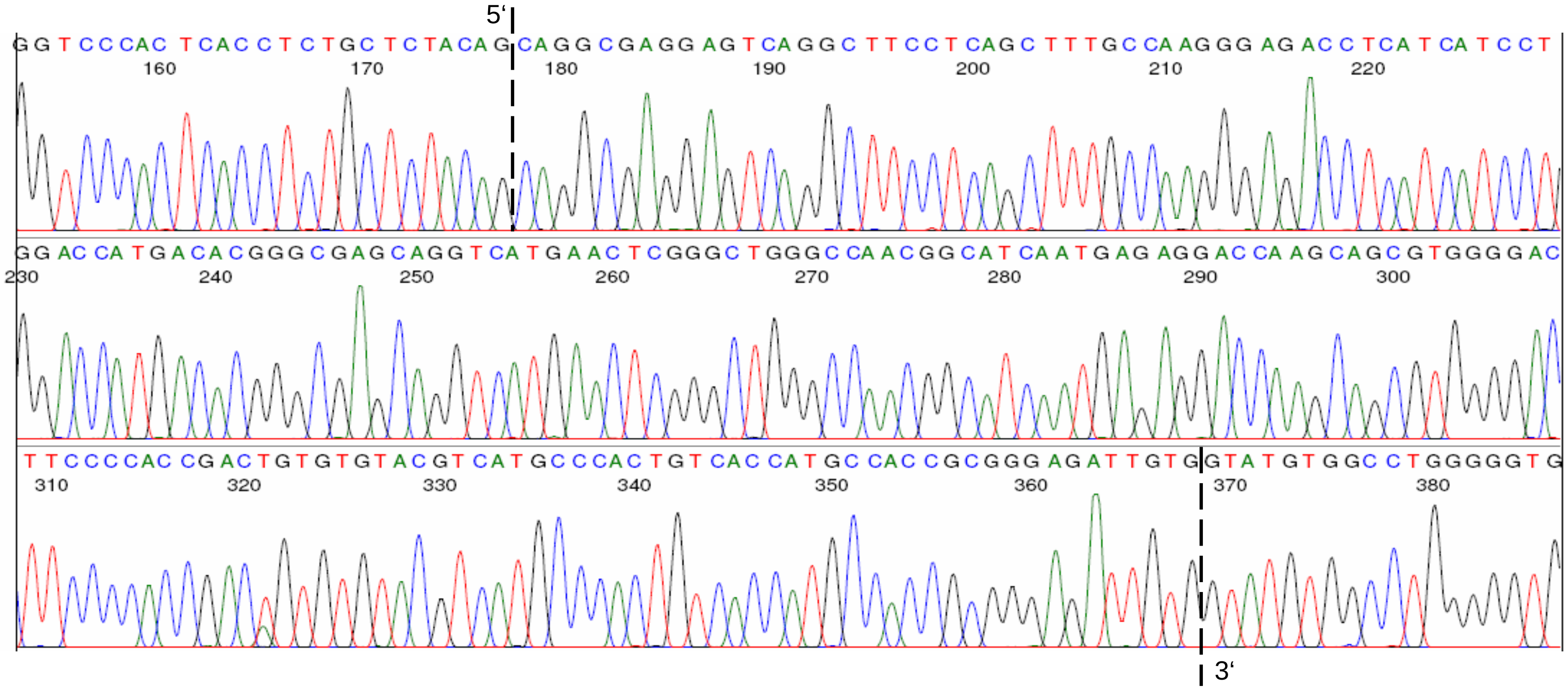

Exon 37

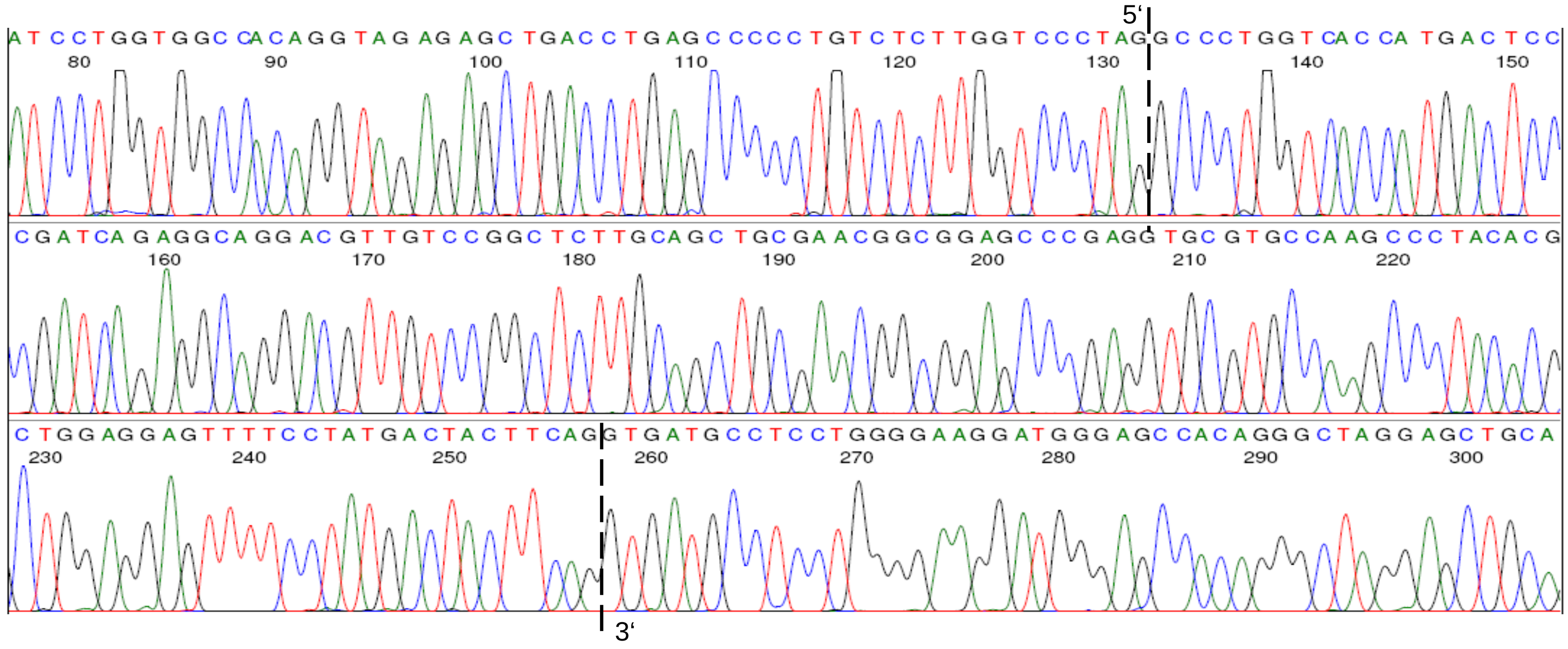

Exon 38

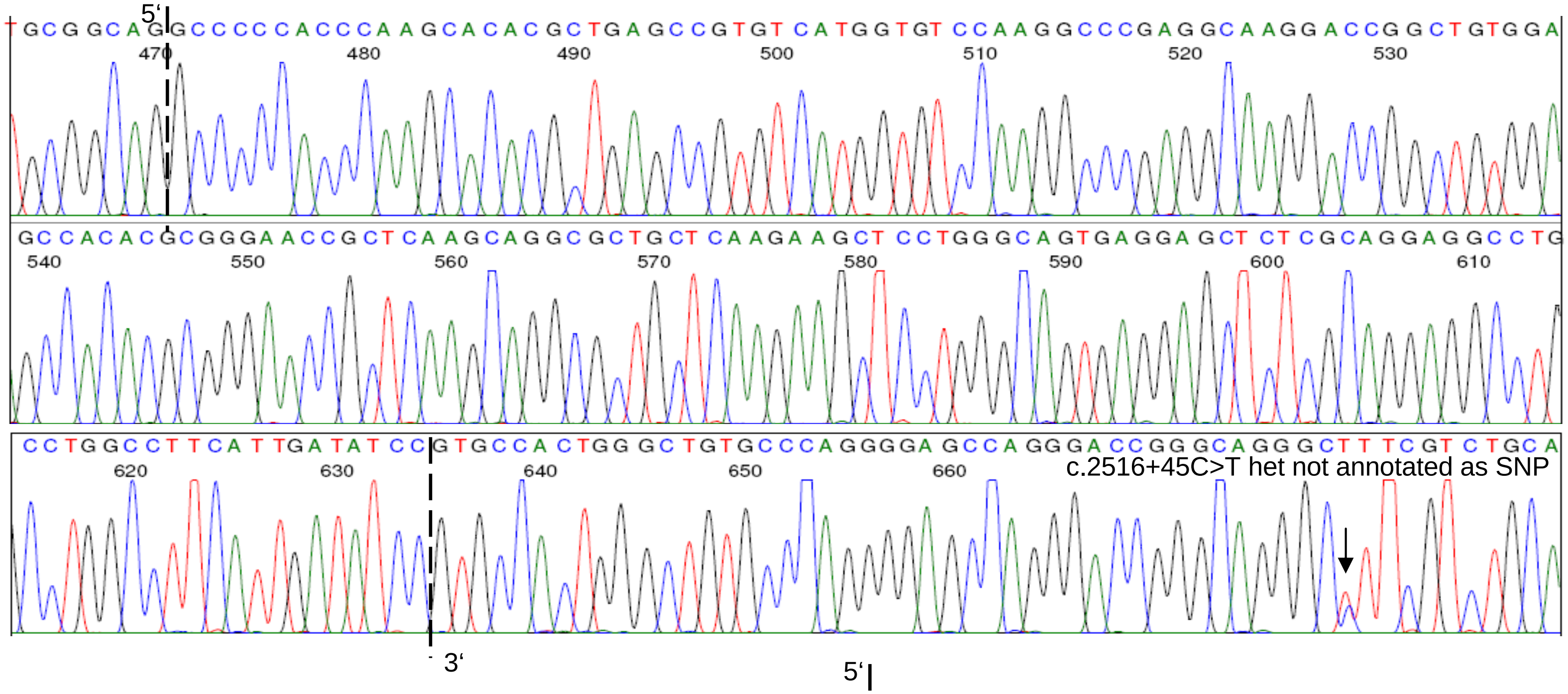

Exon 39

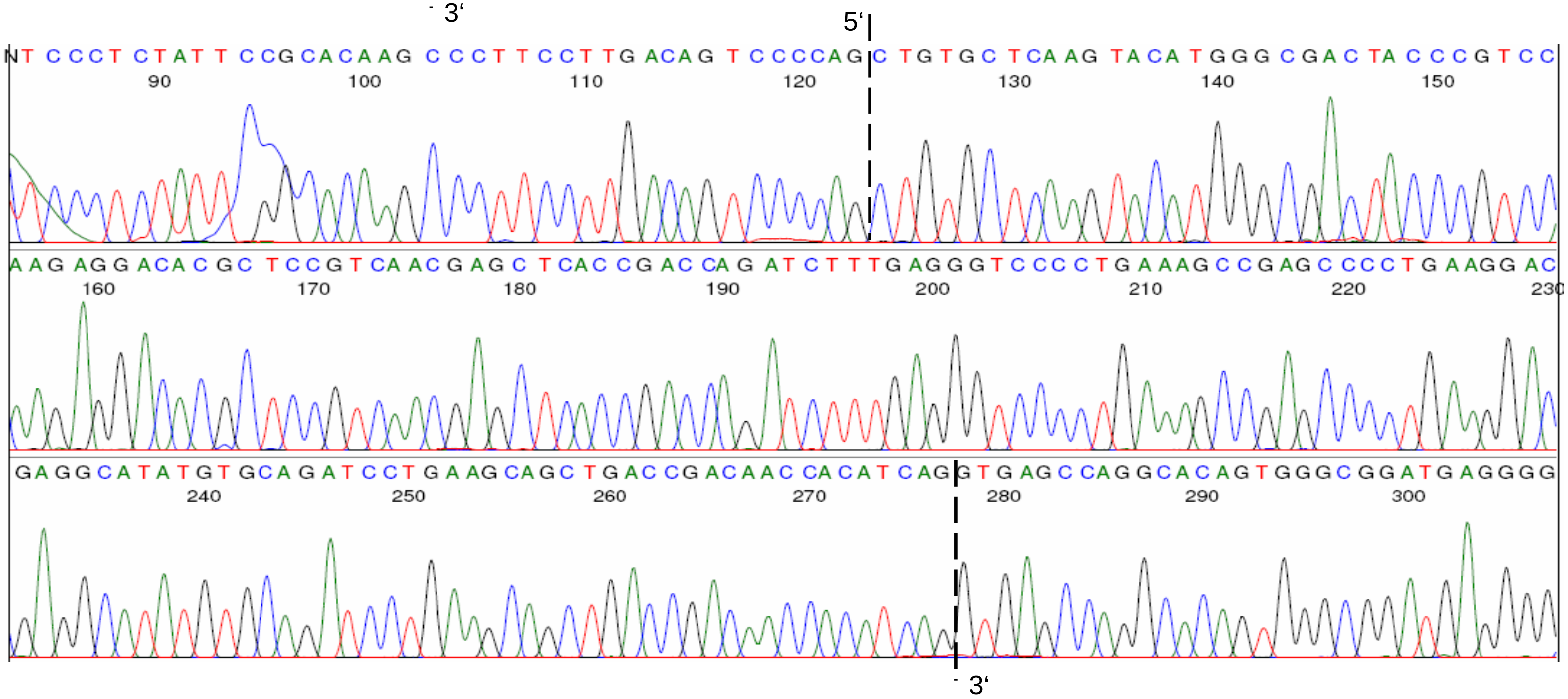

Exon 40

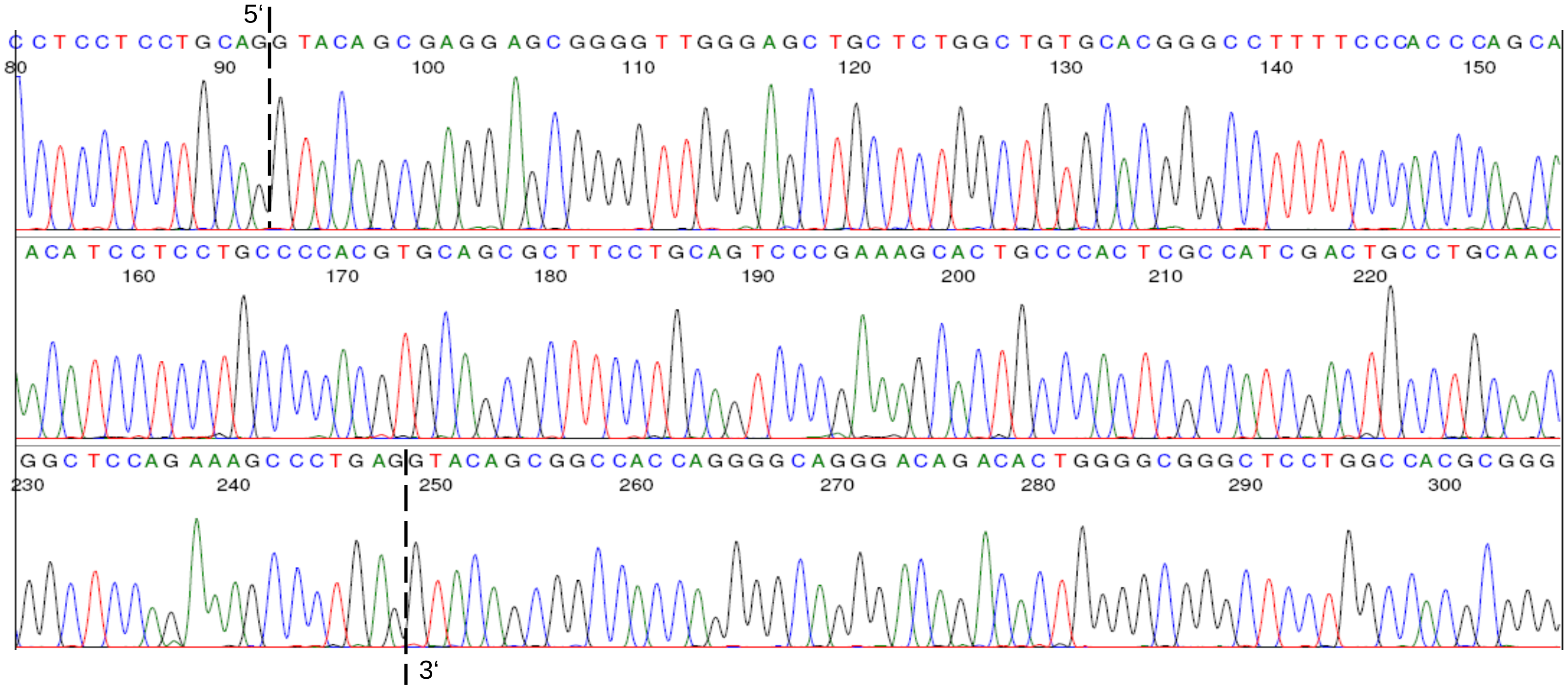

Exon 41

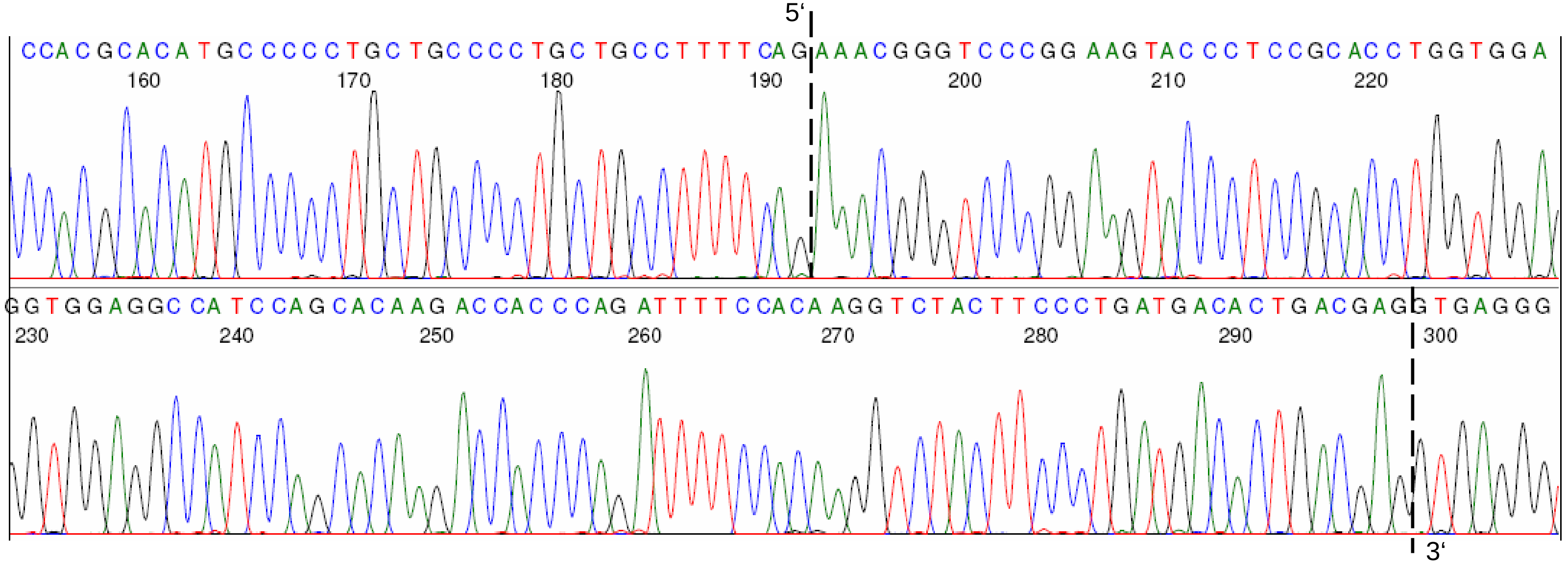

Exon 42

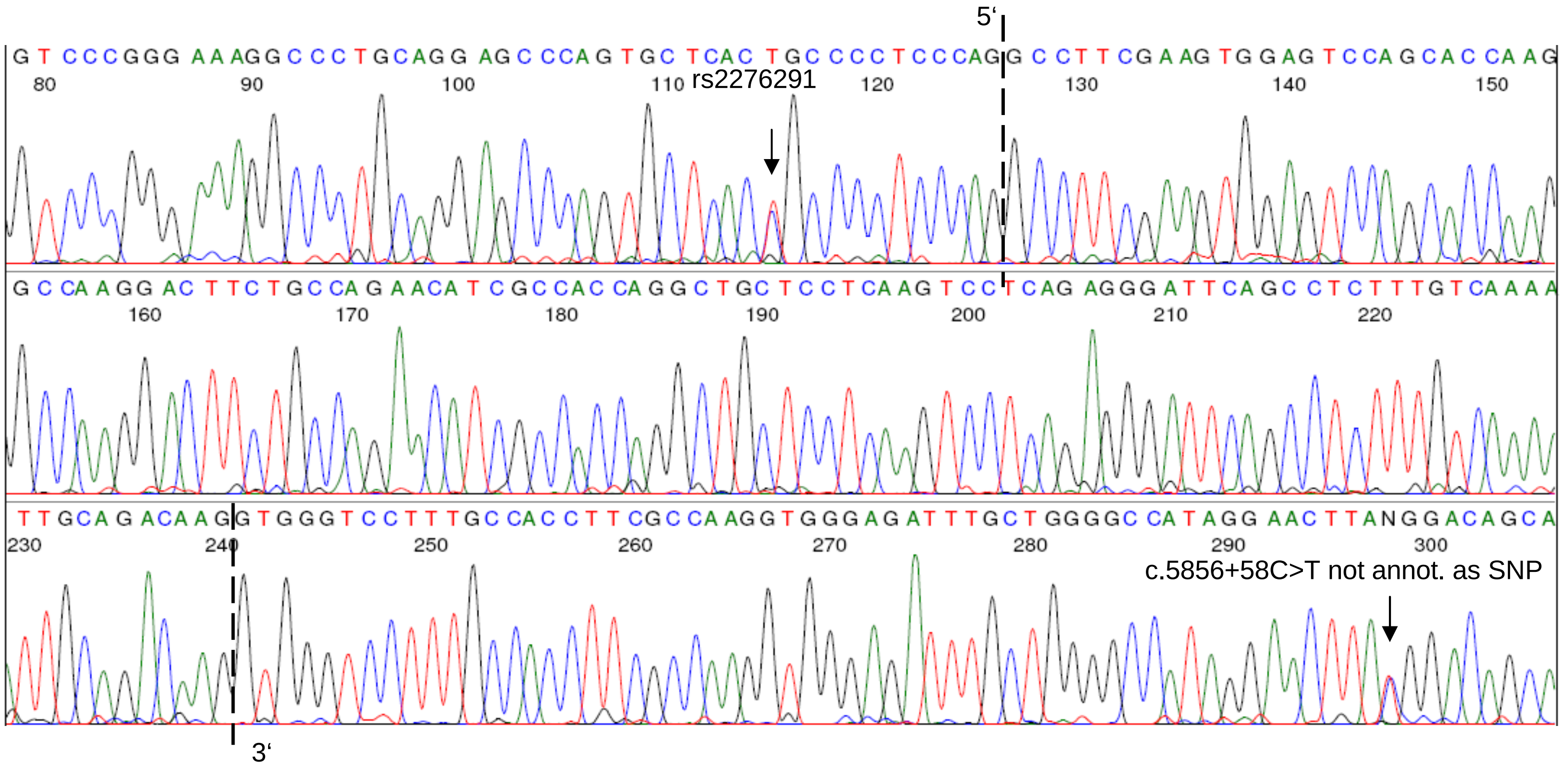

Exon 43

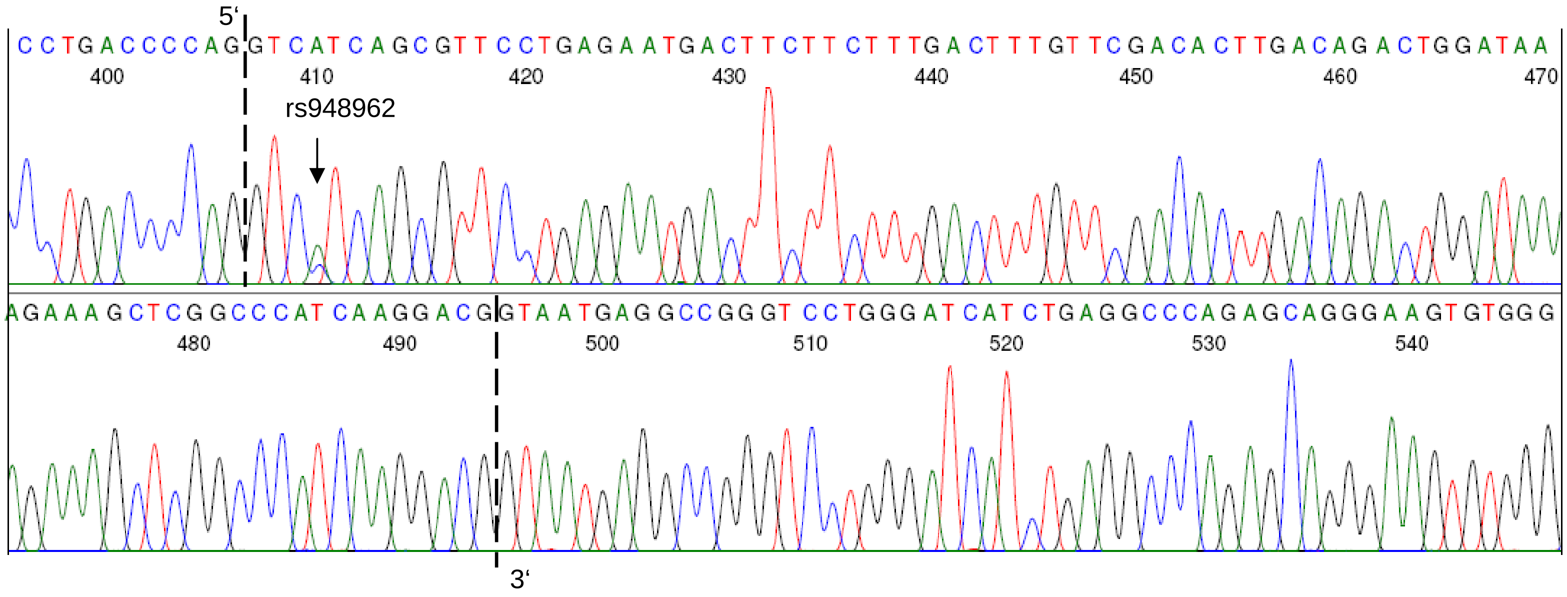

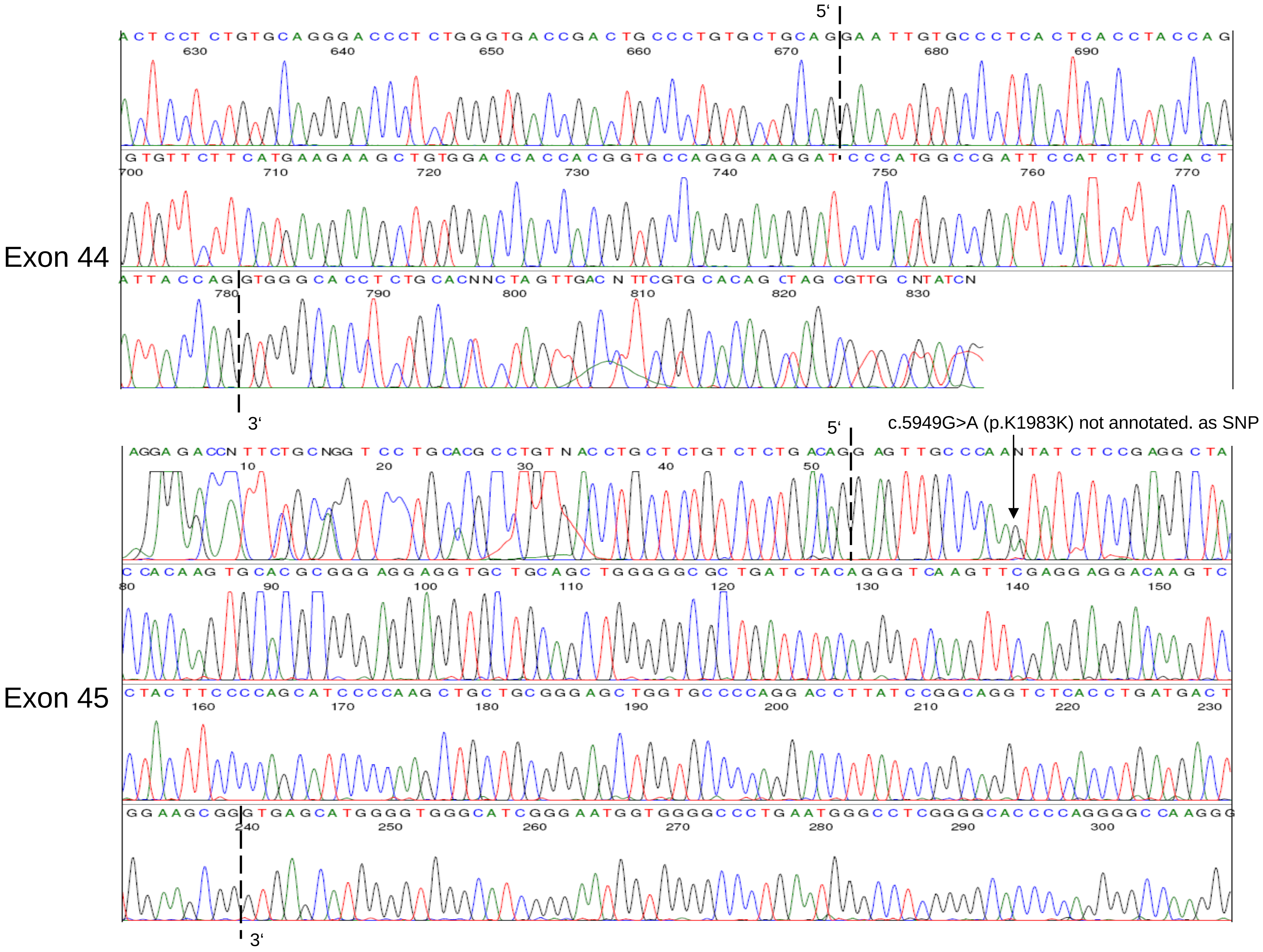
Exon 44
5'
3'
Exon 45
5'
c.5949G>A (p.K1983K) not annotated. as SNP
3'

Exon 46

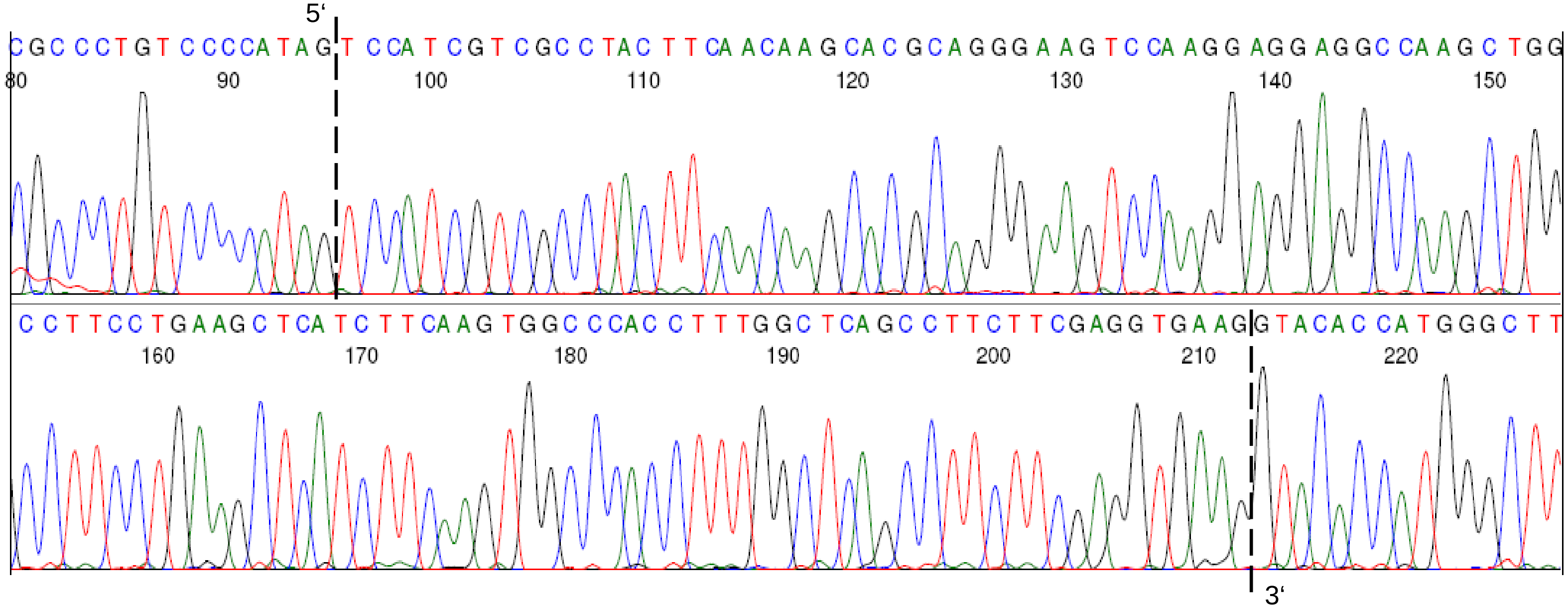

Exon 47

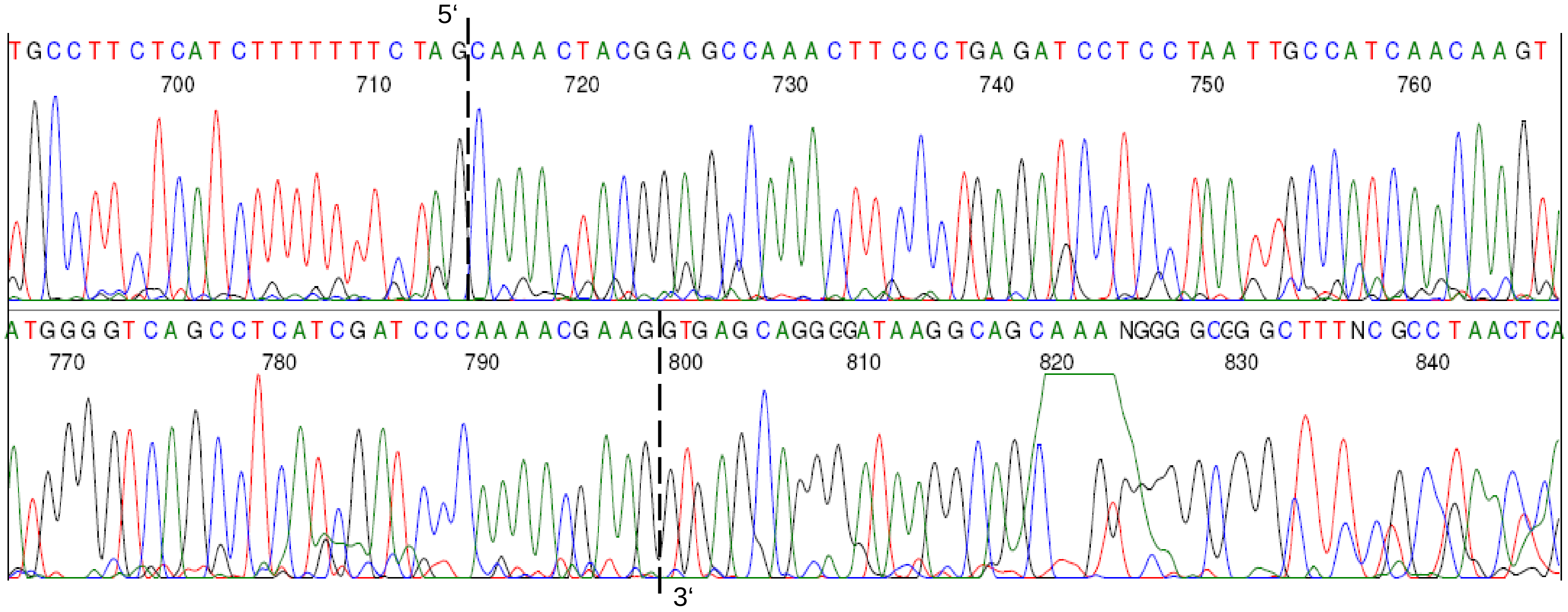

Exon 48

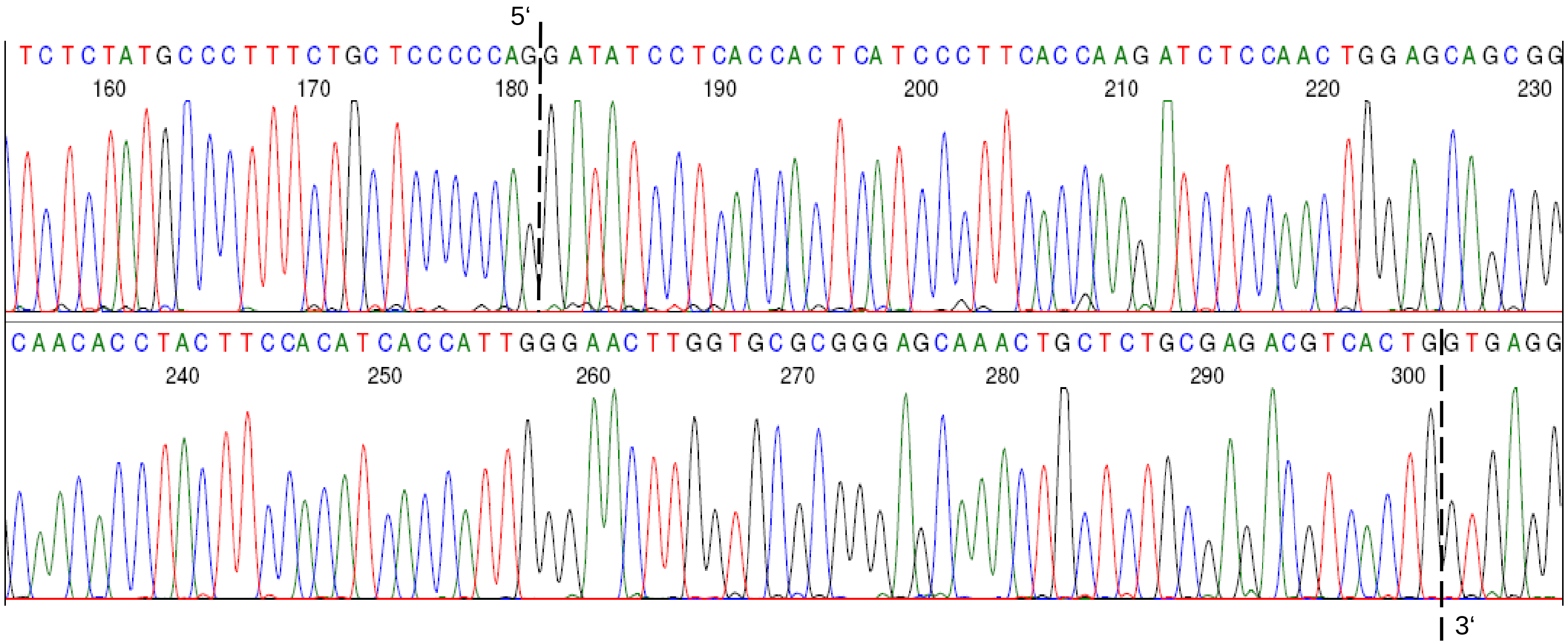

Exon 49

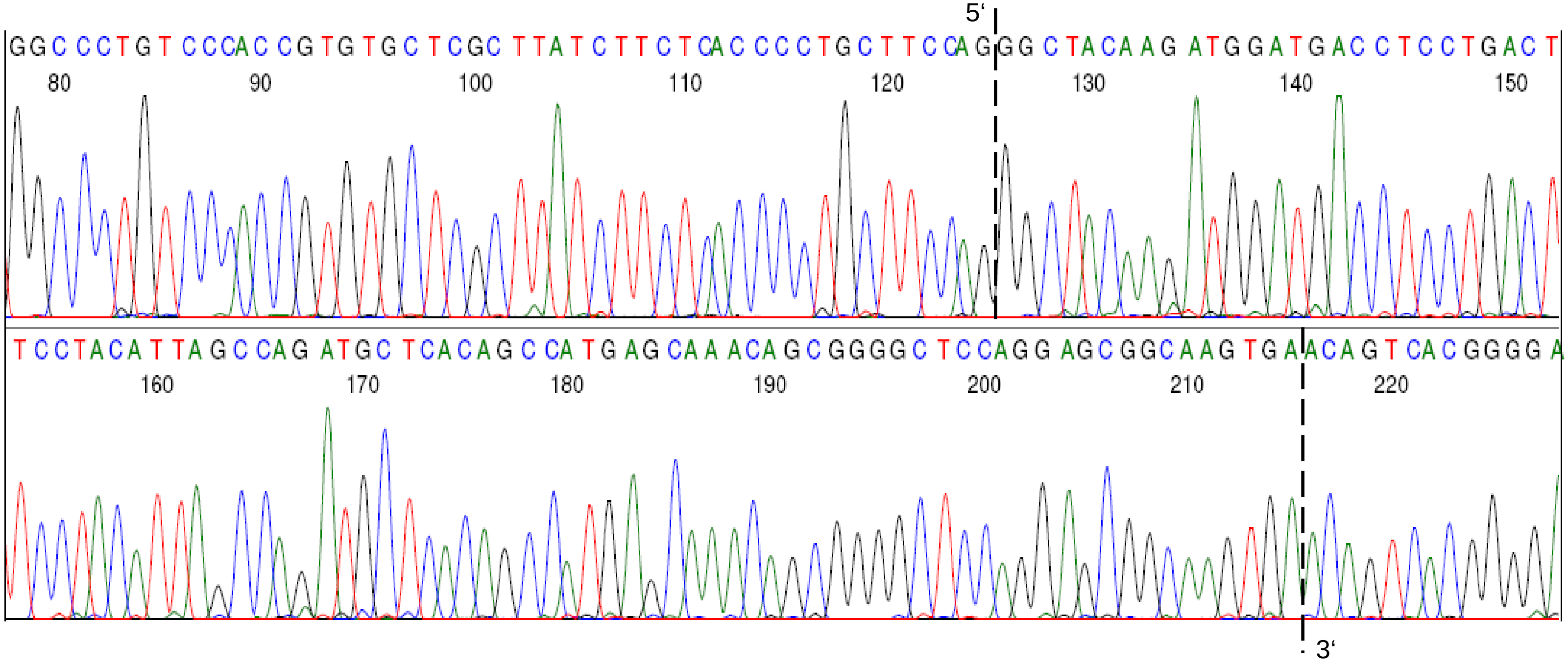

Patient 1881, *USH1C*

Exon 1

Exon 2

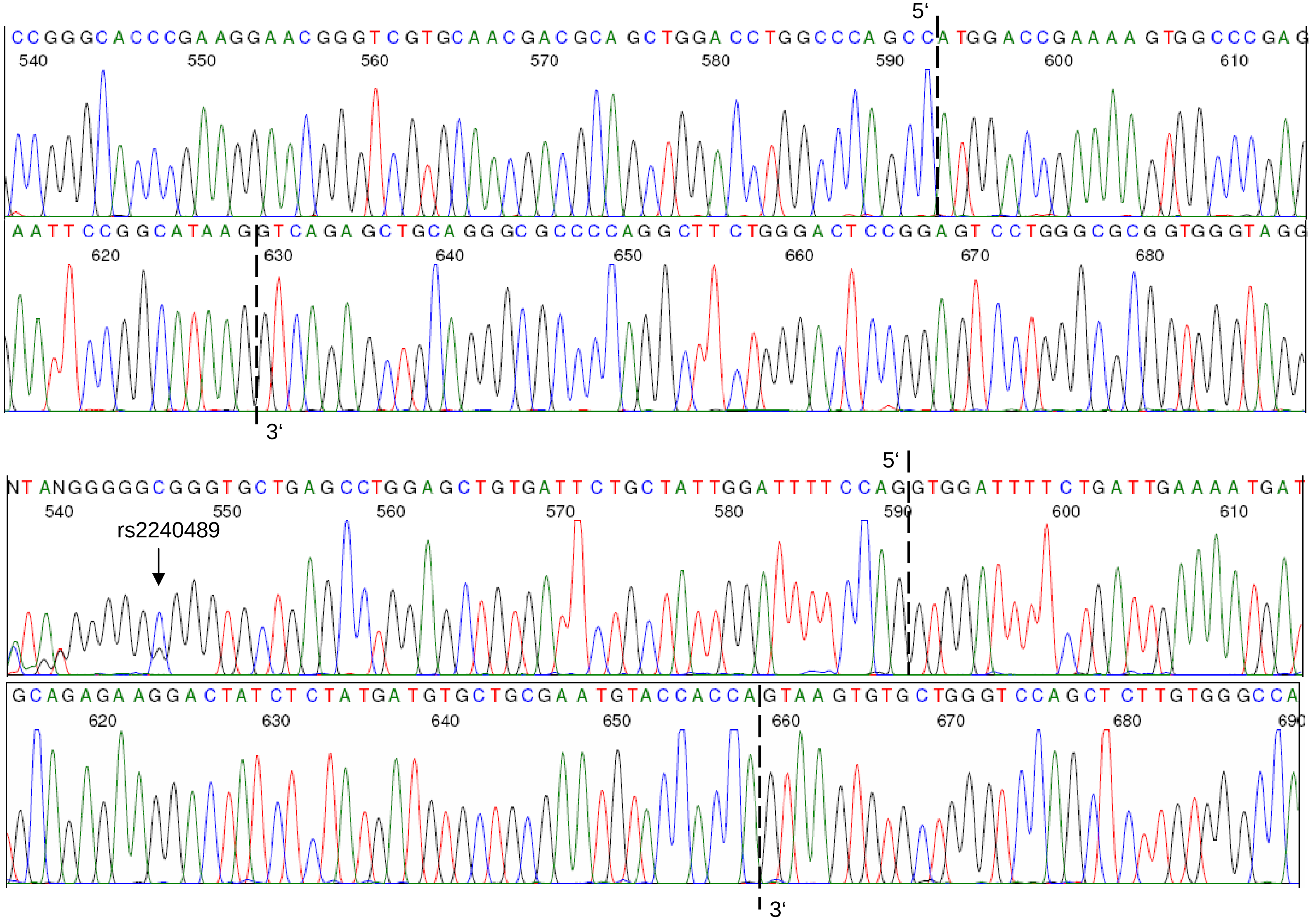

Exon 3

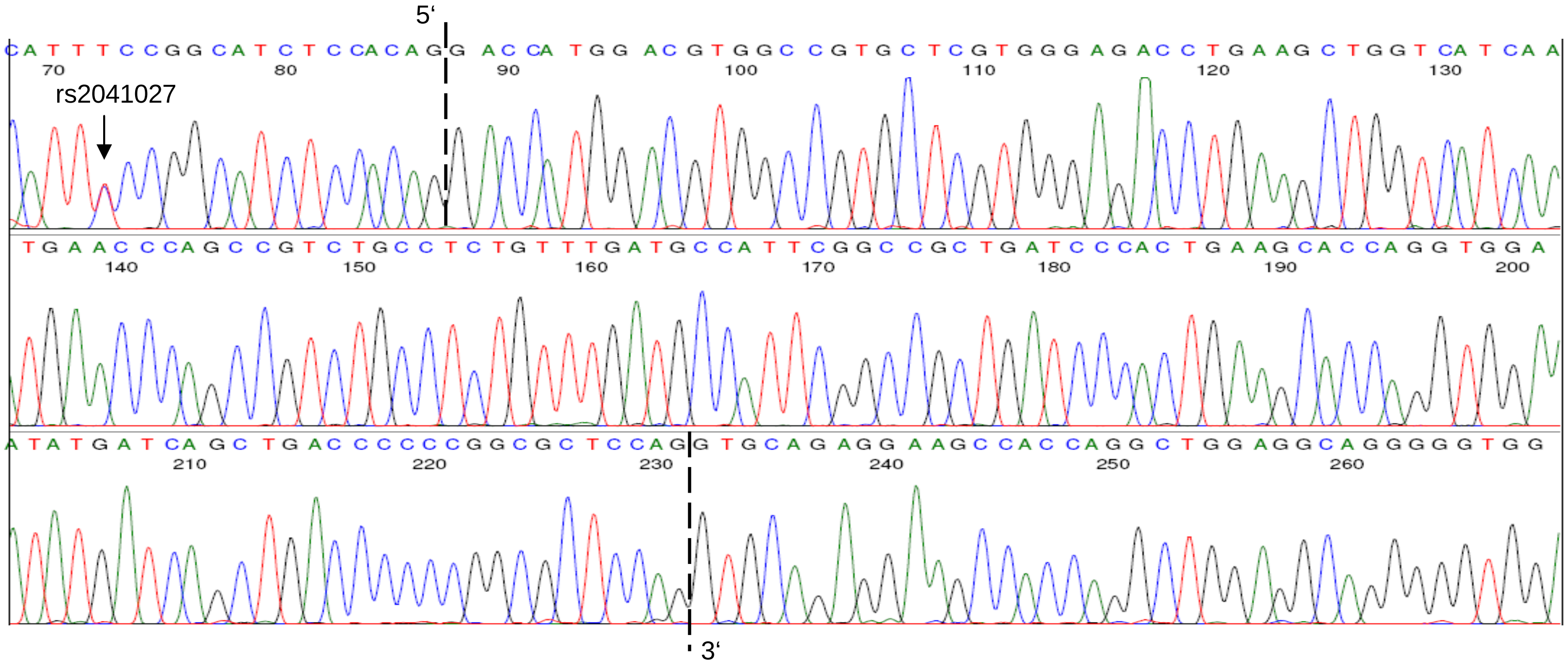

Exon 4

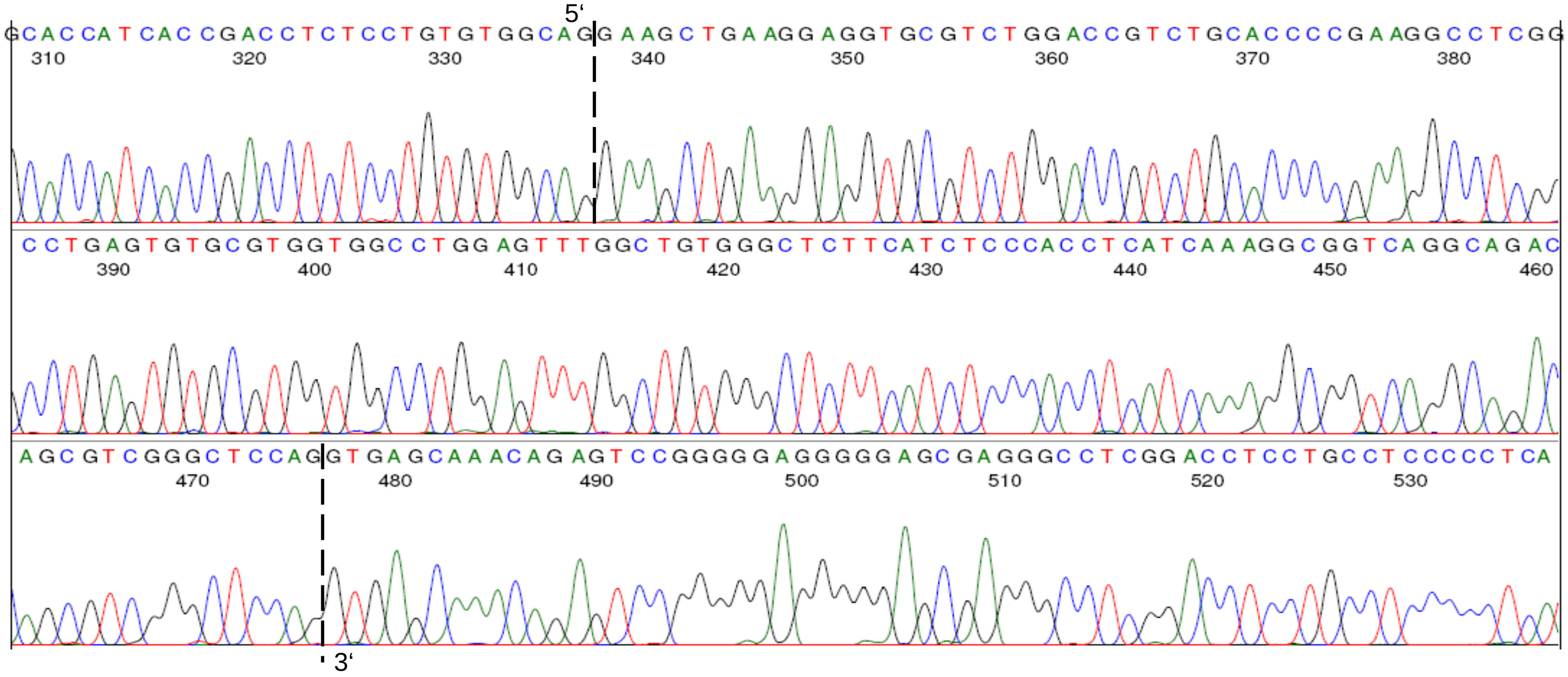

Exon 5

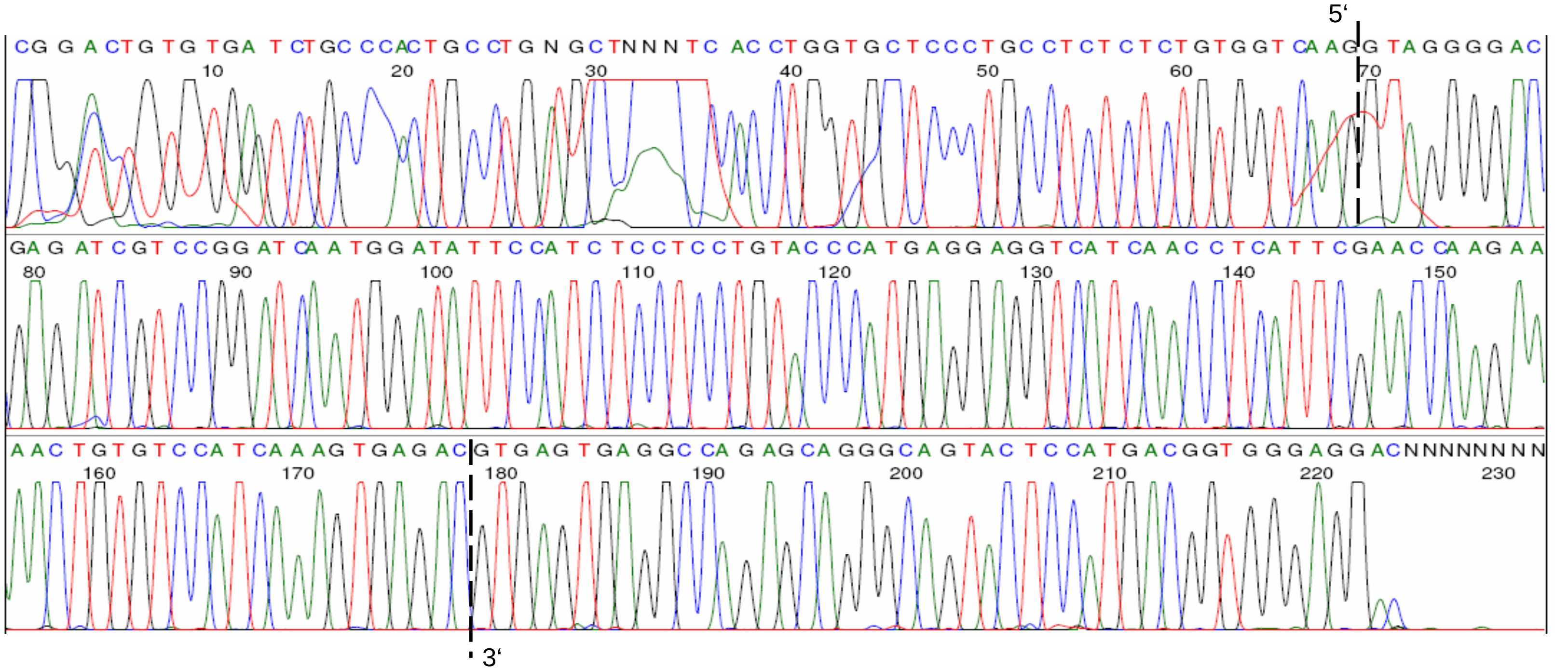

Exon 6

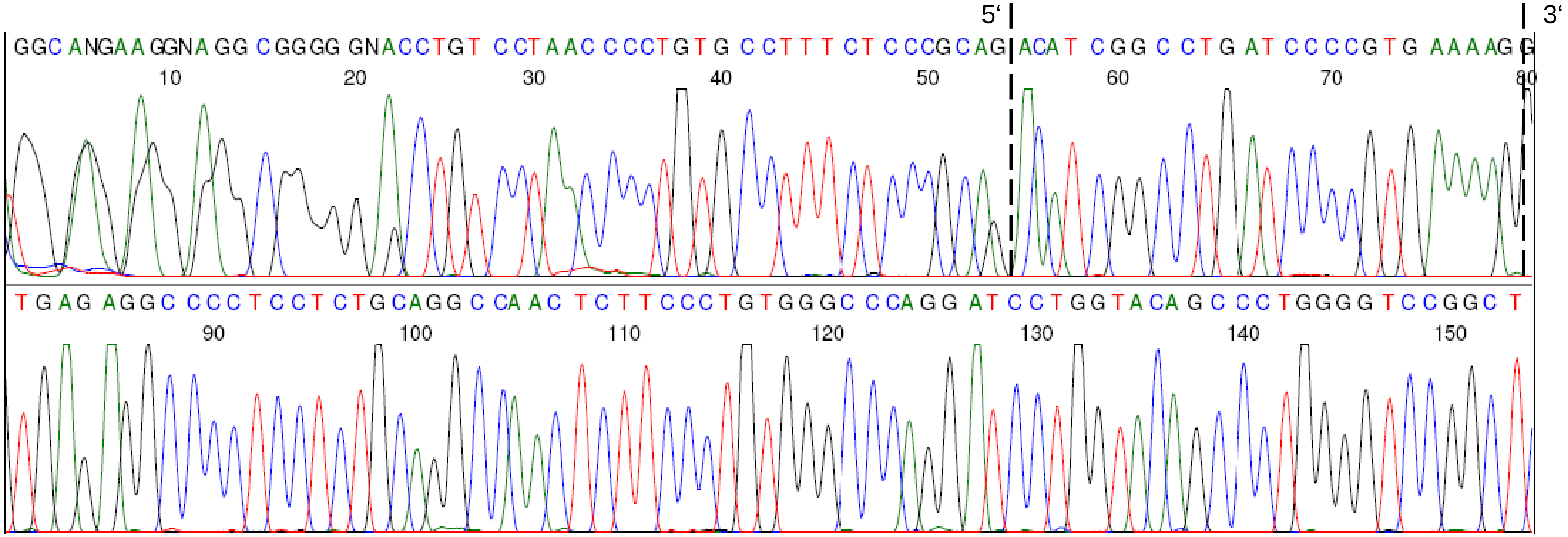

Exon 7

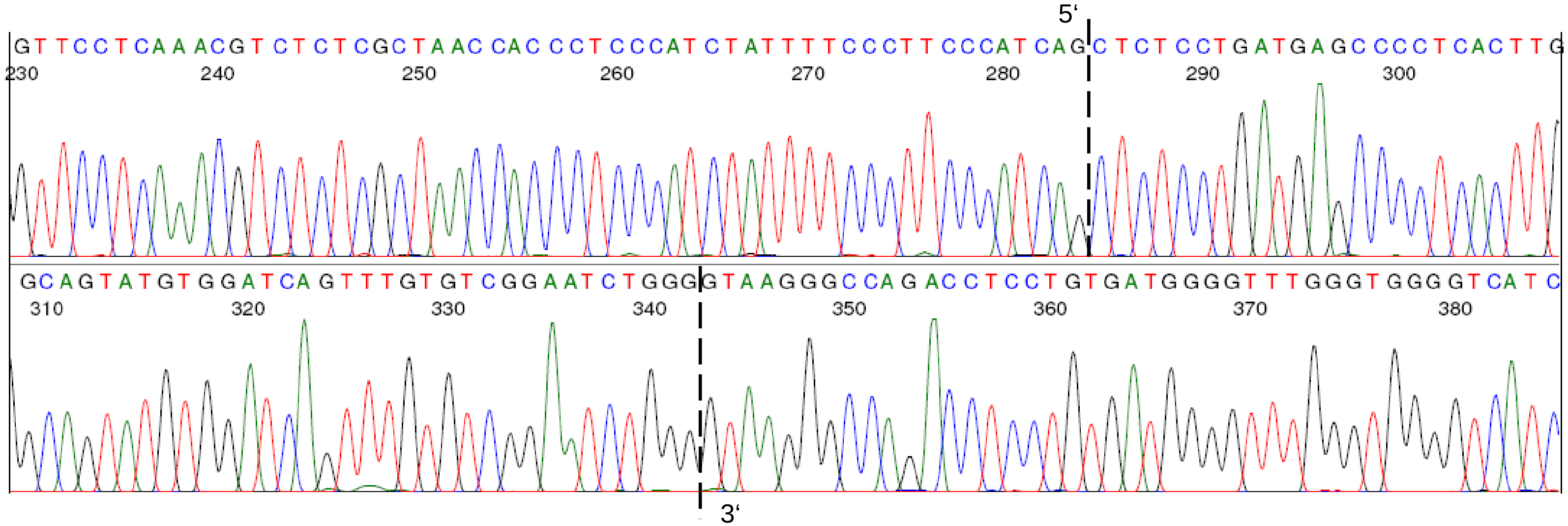

Exon 8

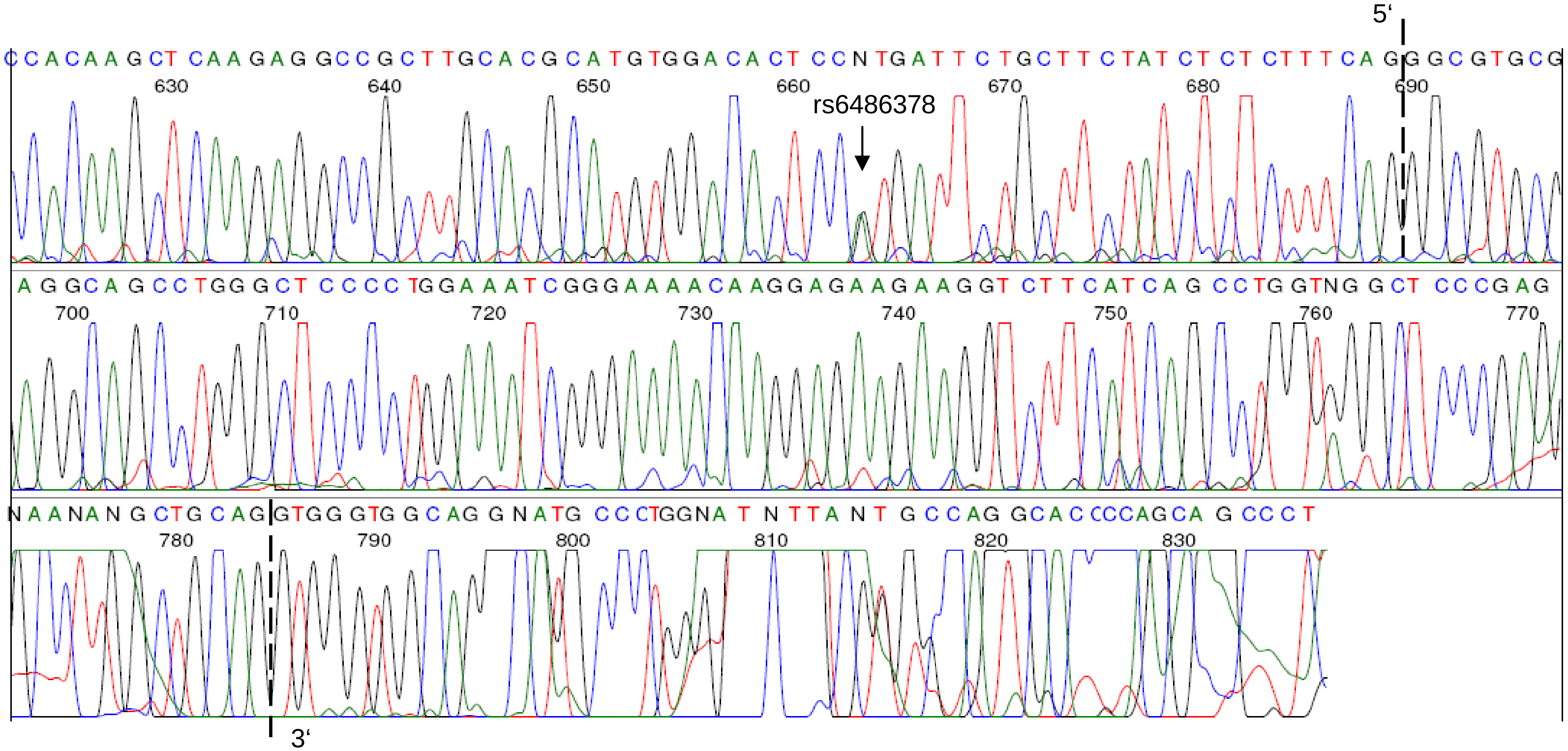

Exon 9

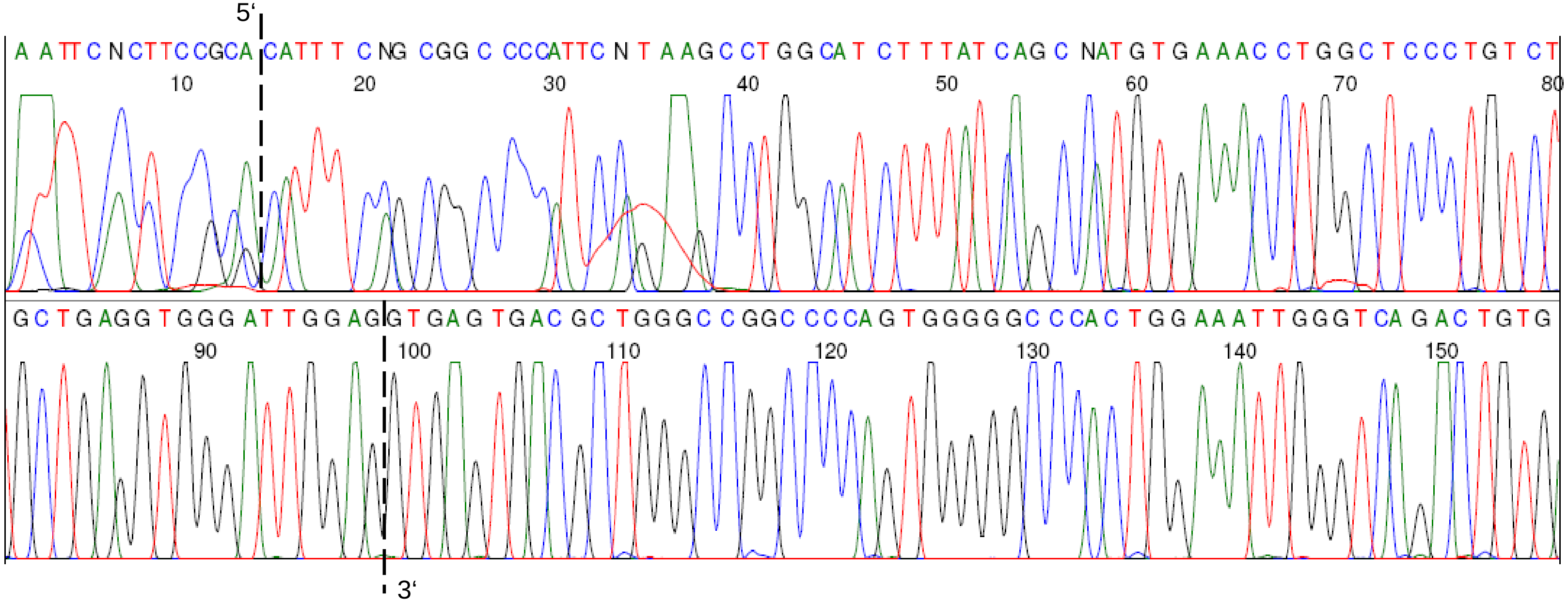

Exon 10

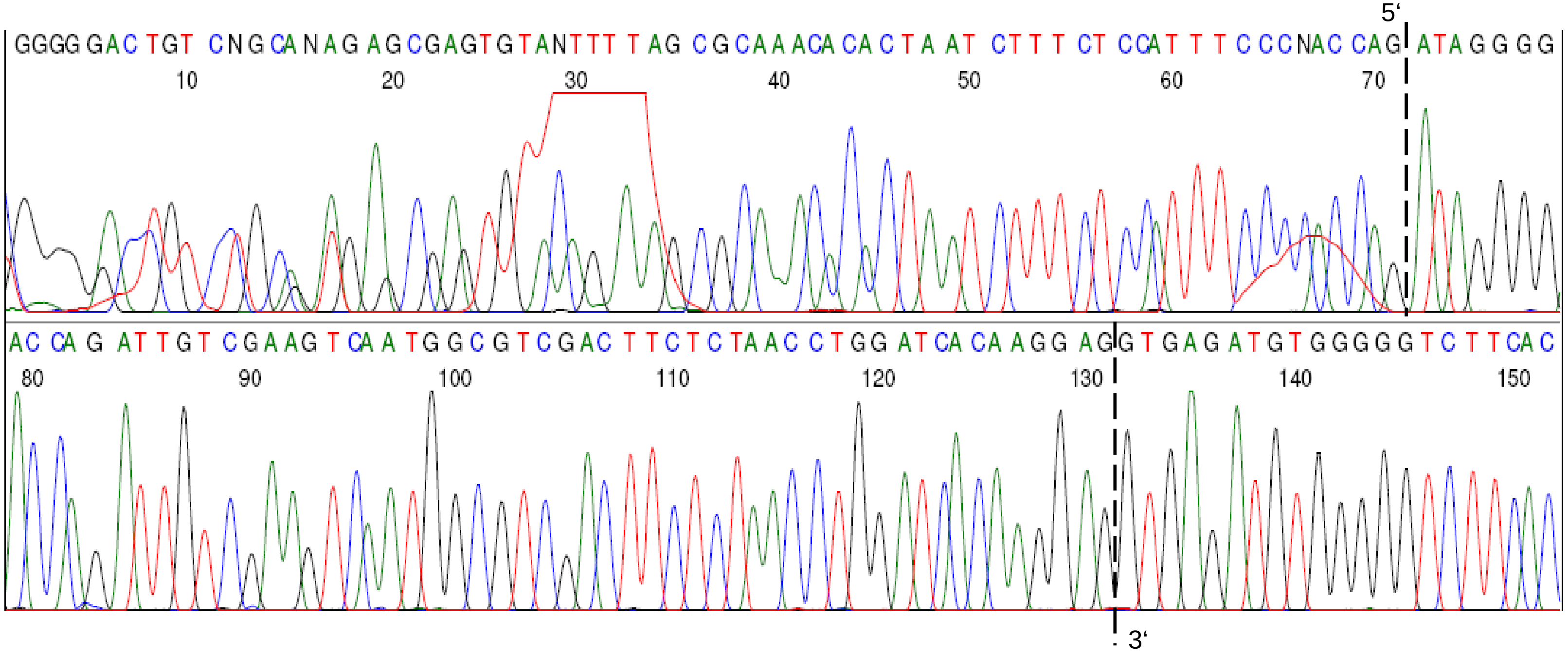

Exon 11

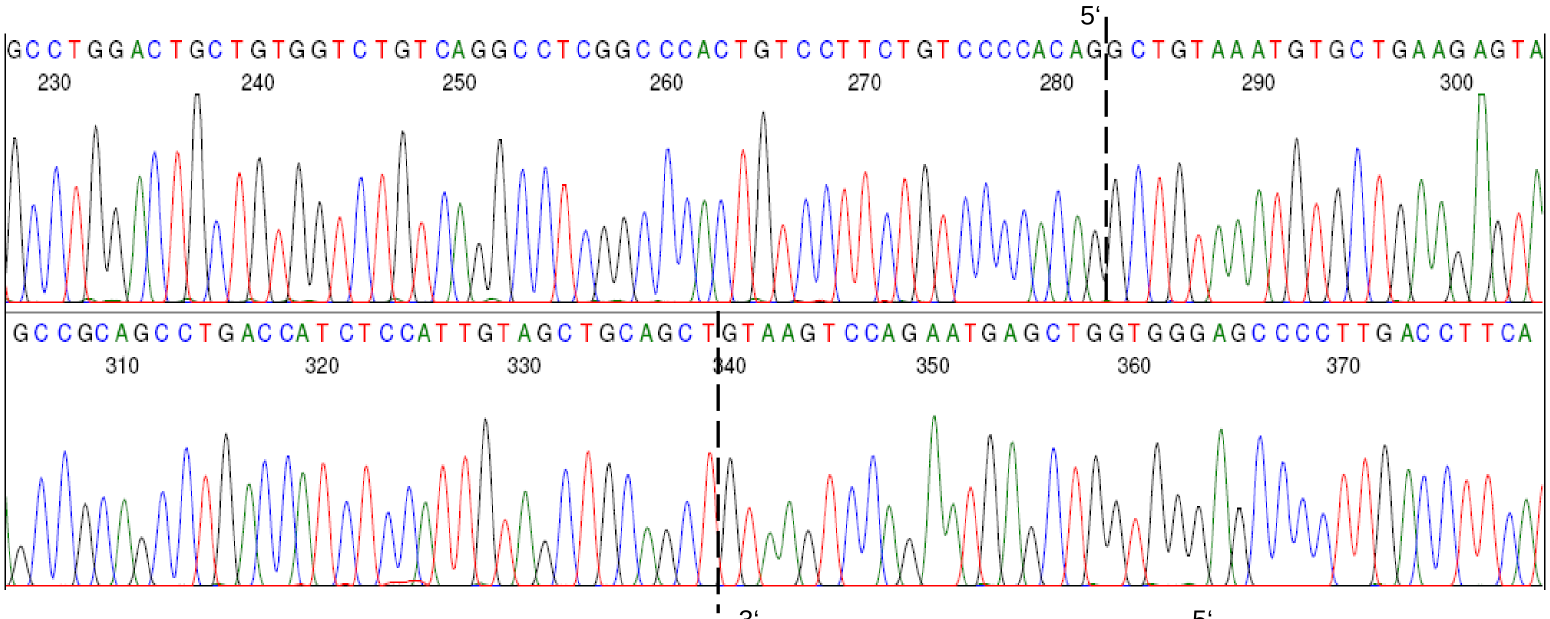

Exon 12

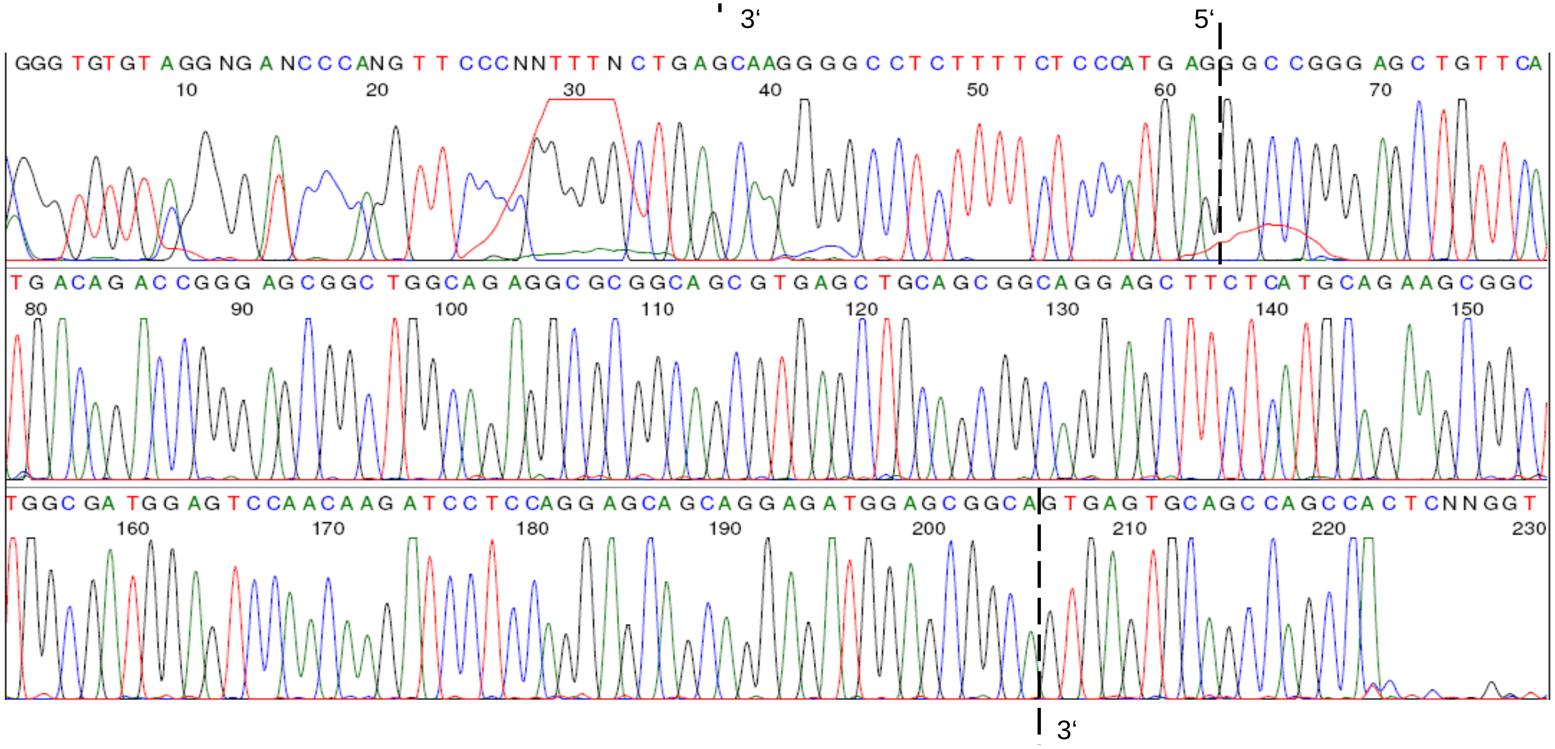

Exon 13

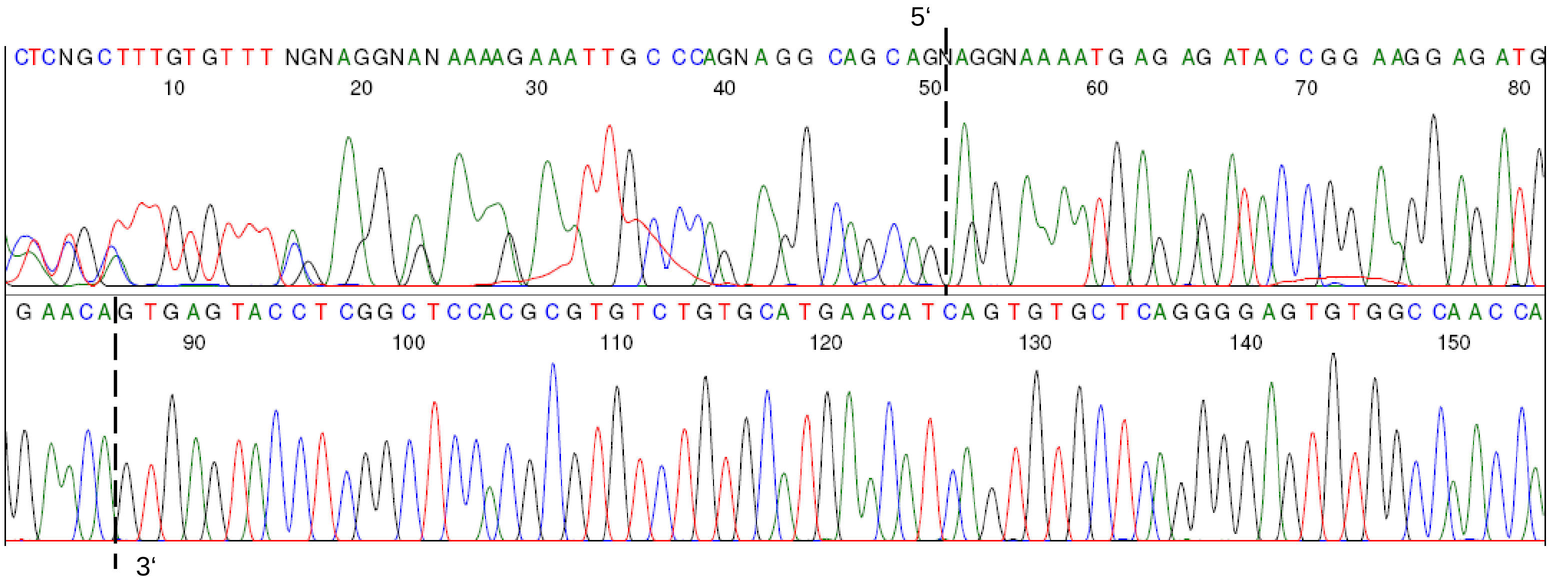

Exon 14

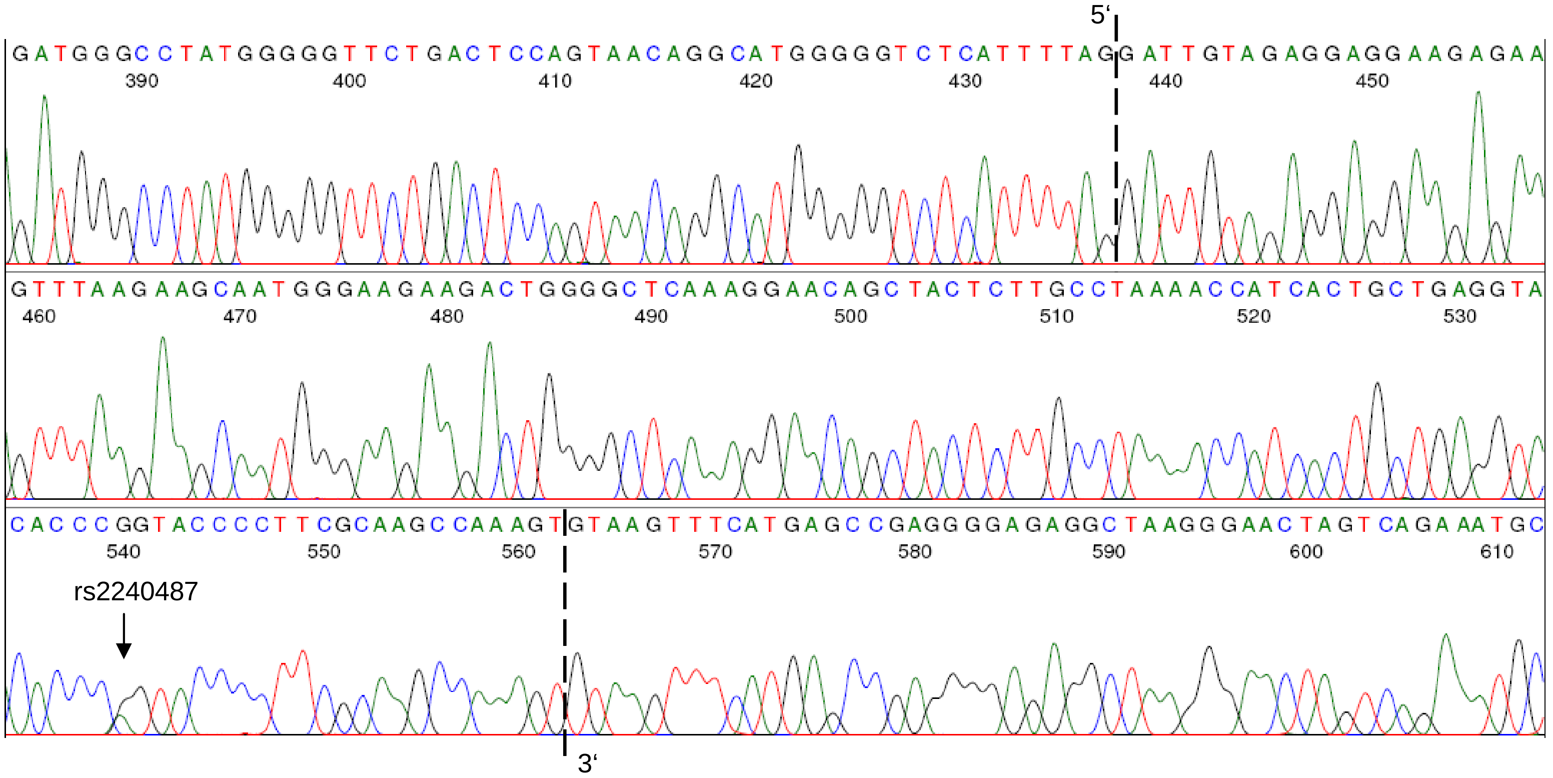

Exon 15

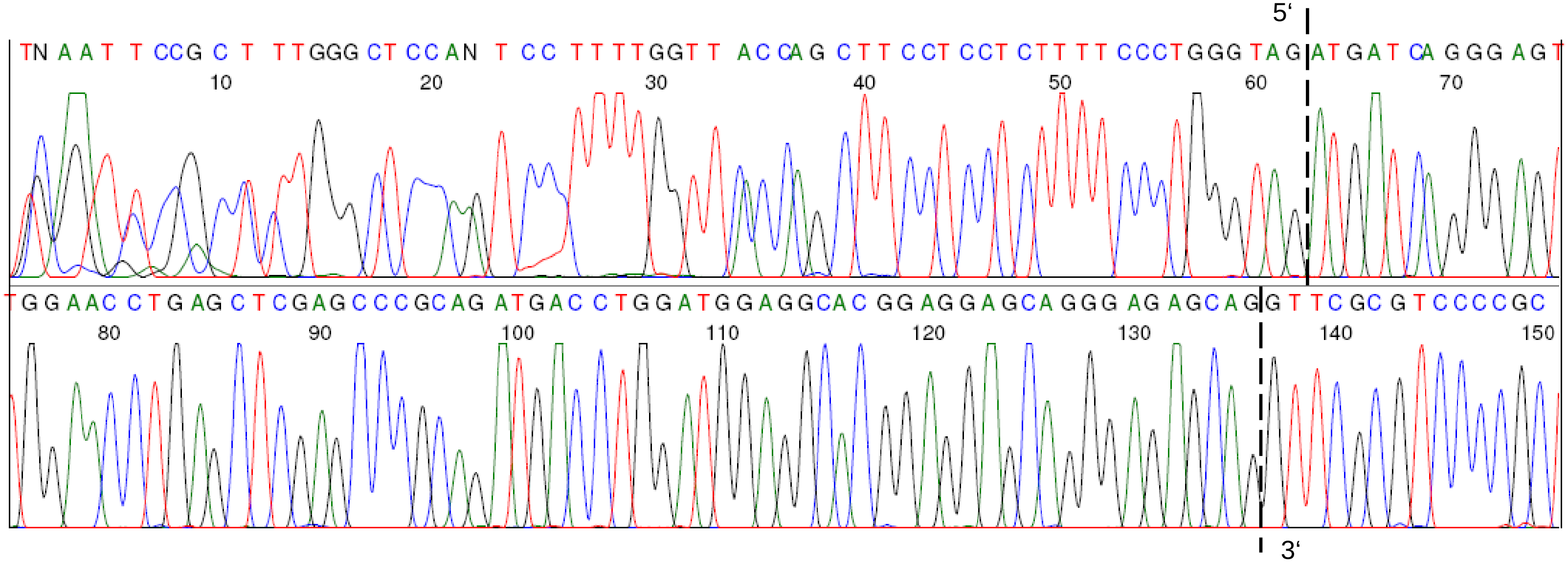

Exon A

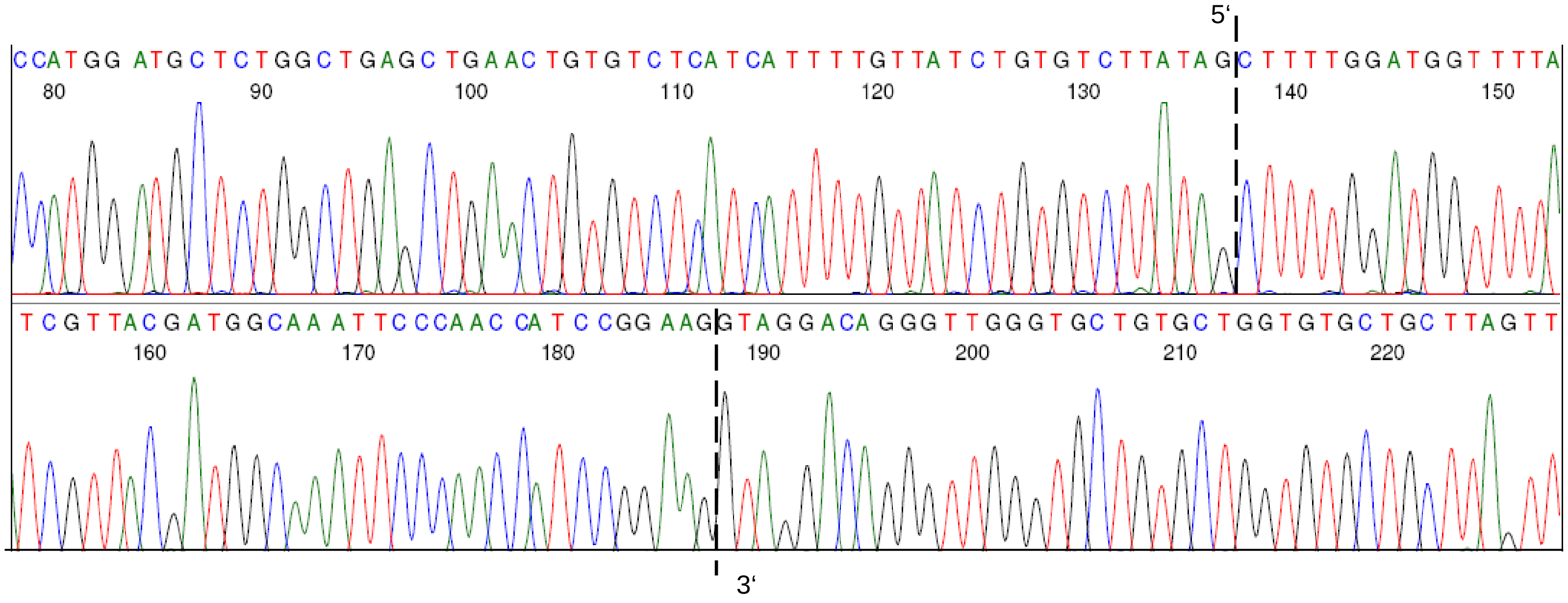

Exon B

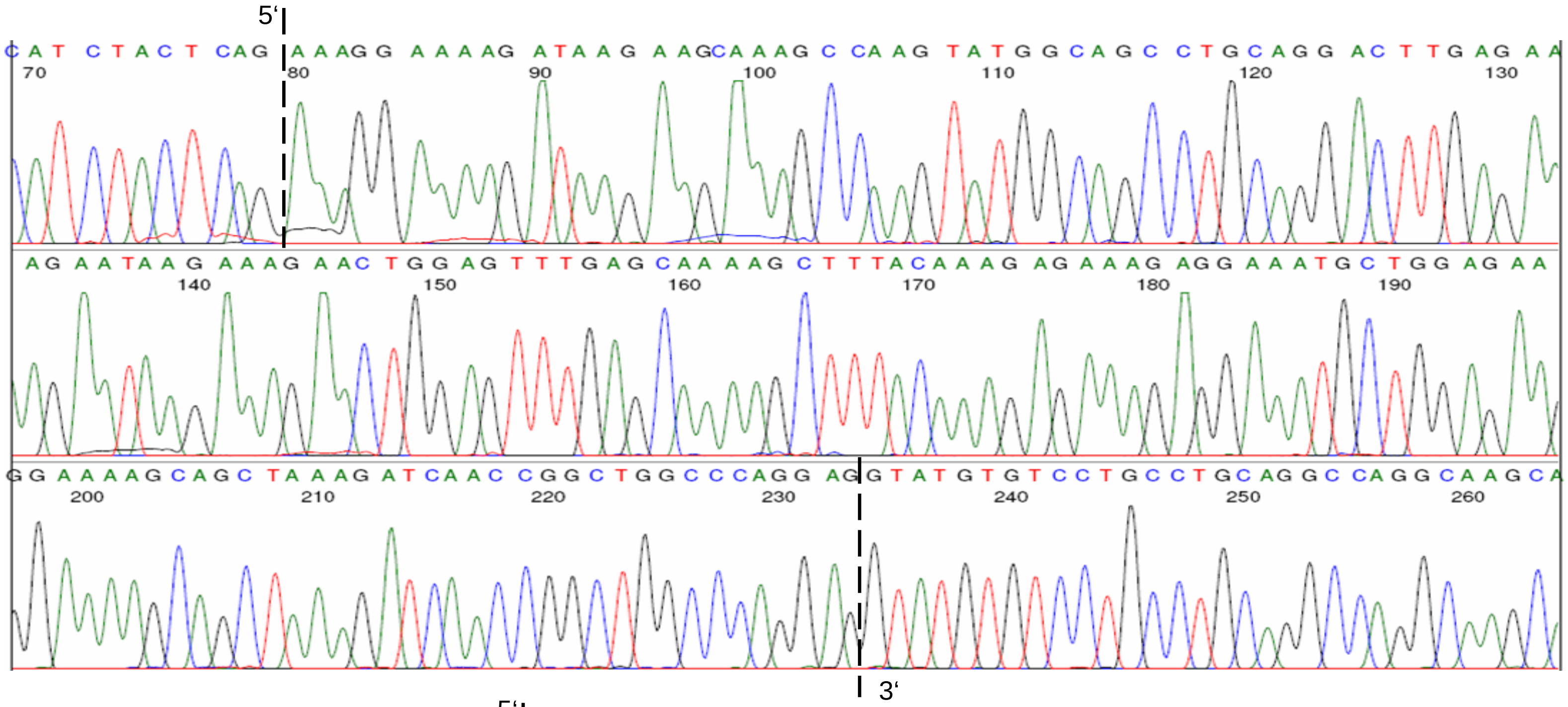

Exon C

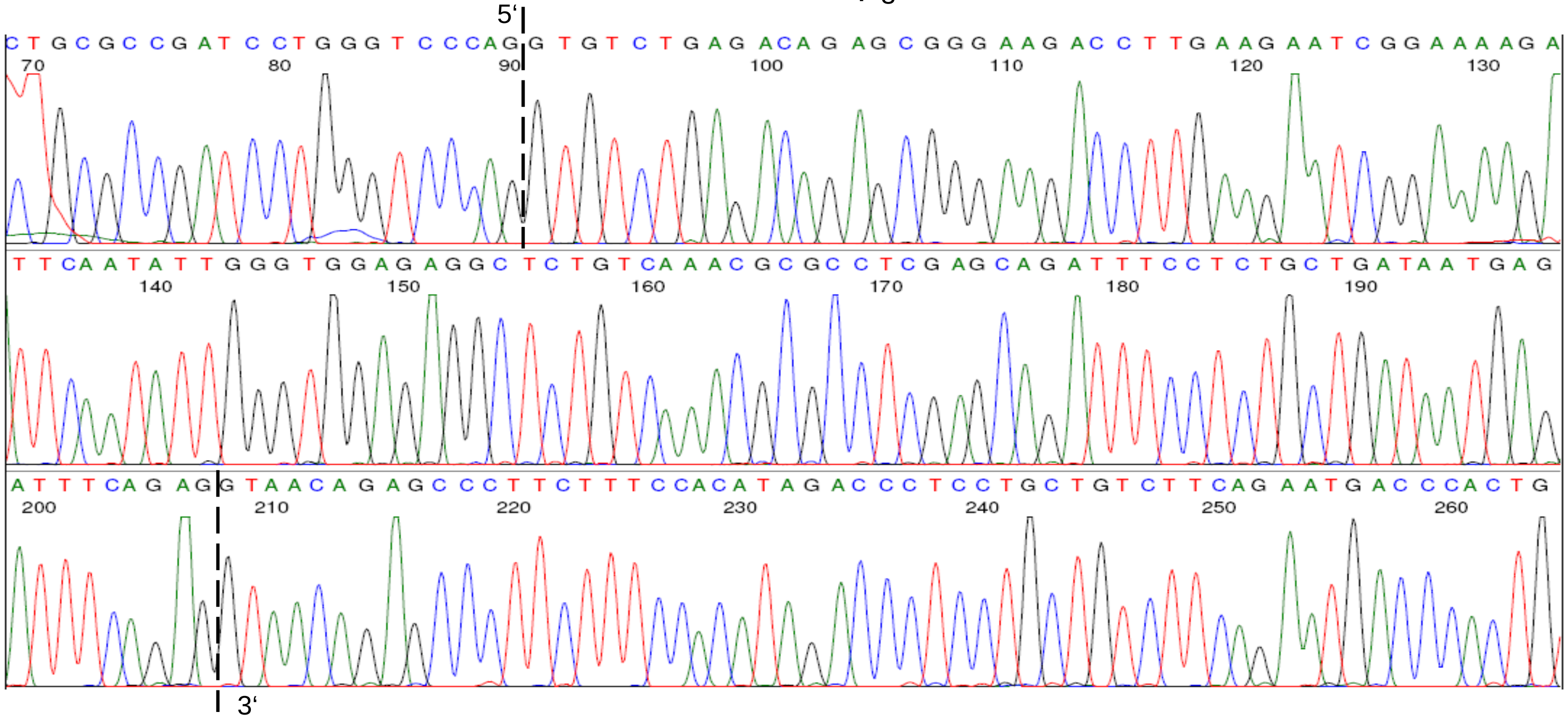

Exon D

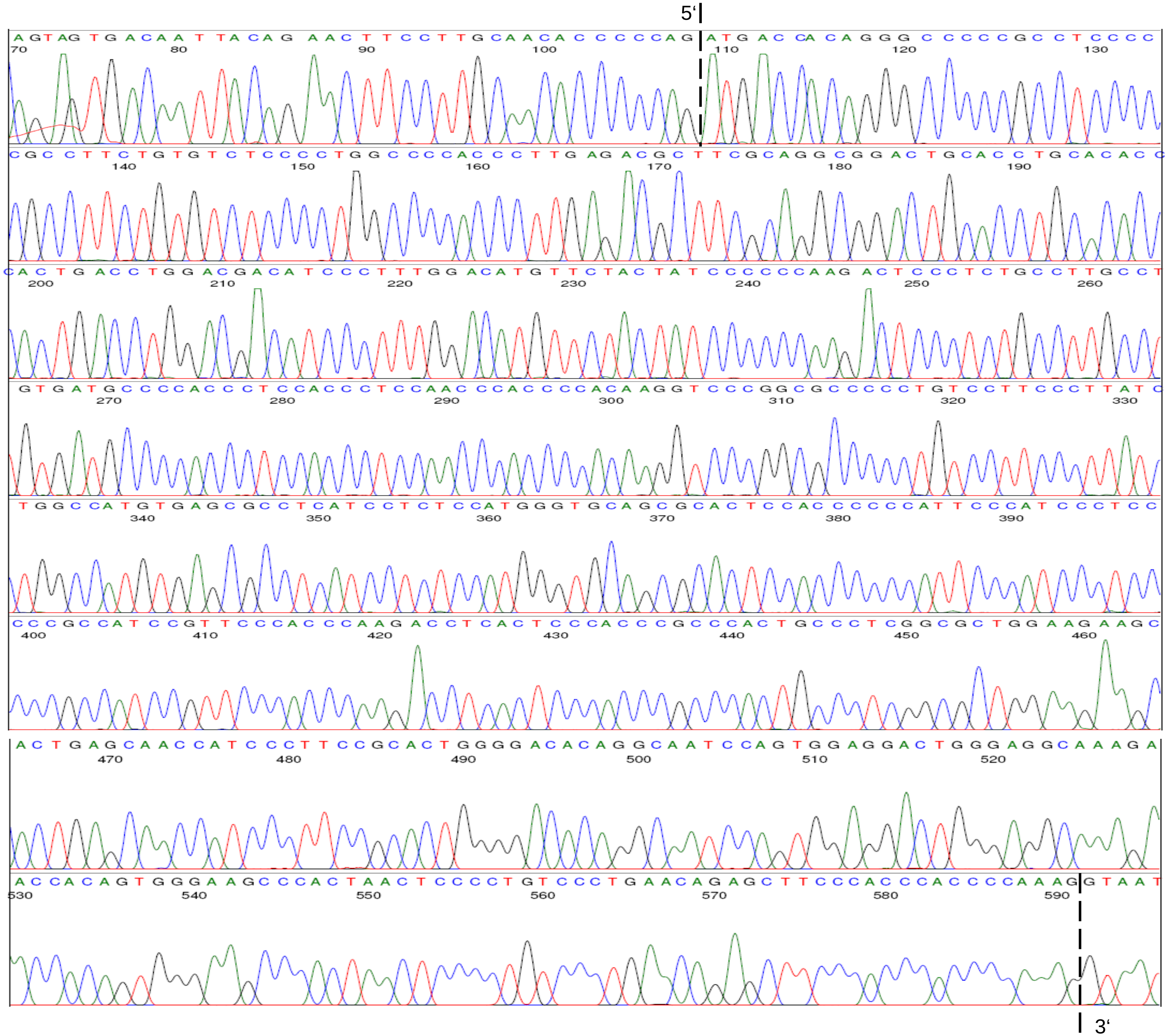

Exon E

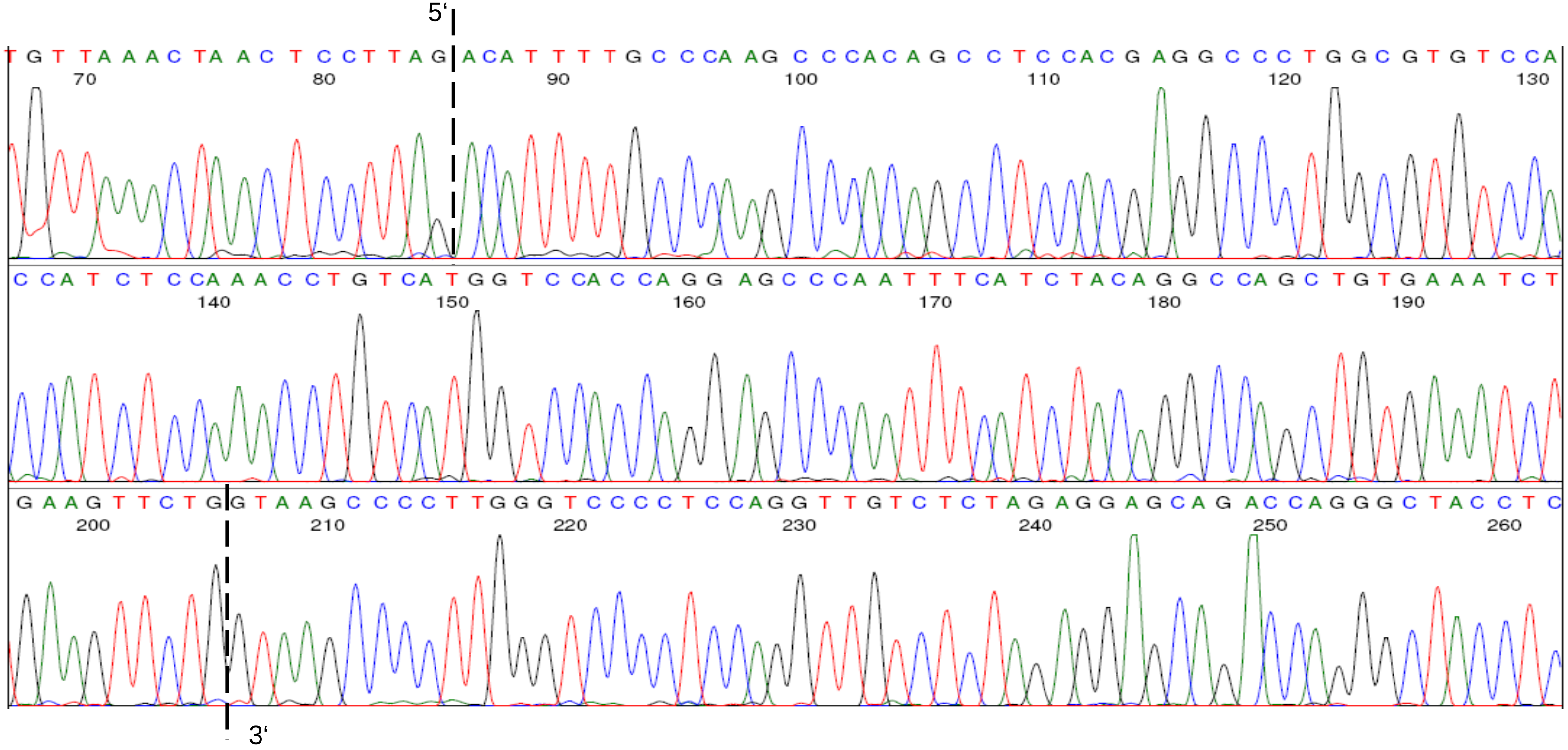

Exon F

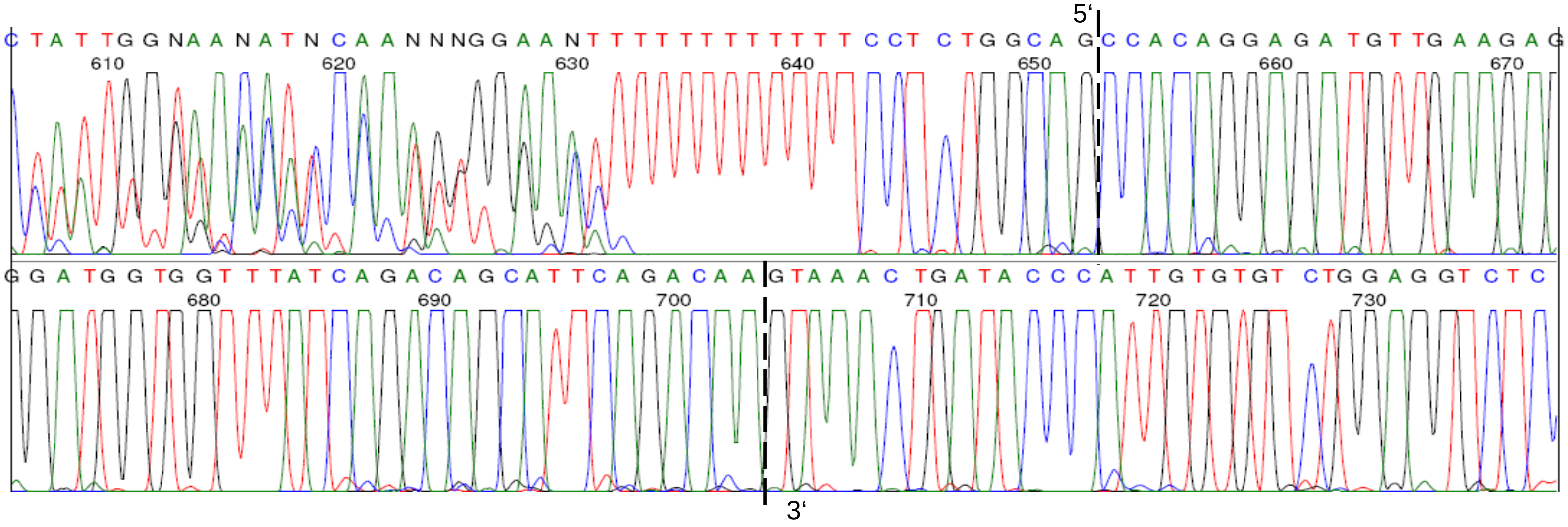

Exon 16

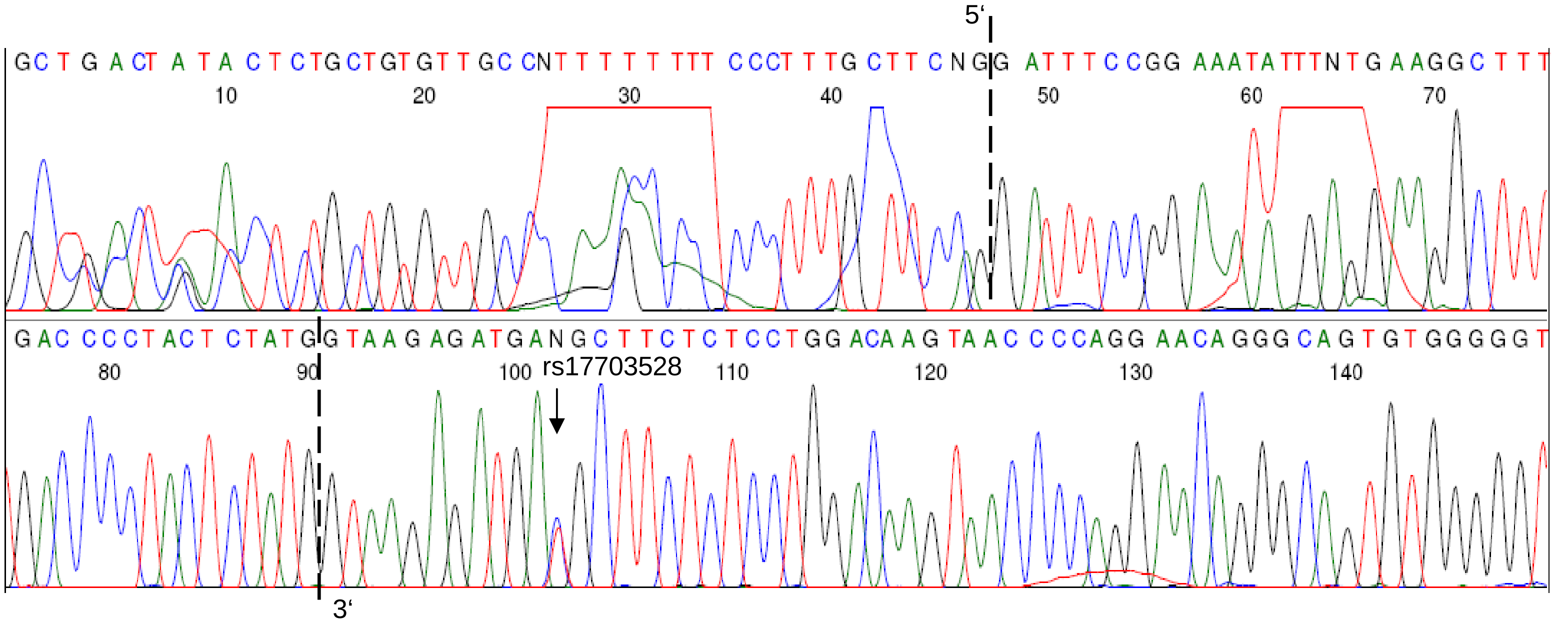

Exon 17

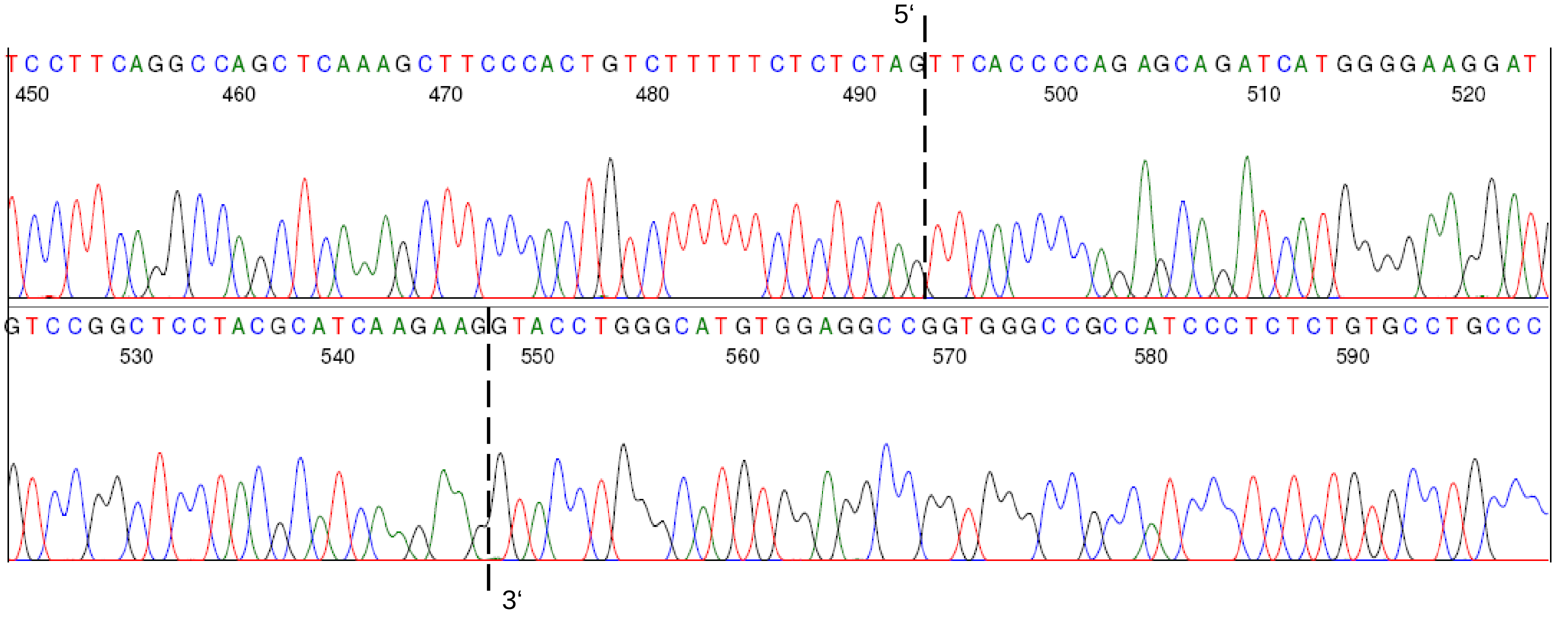

Exon 18

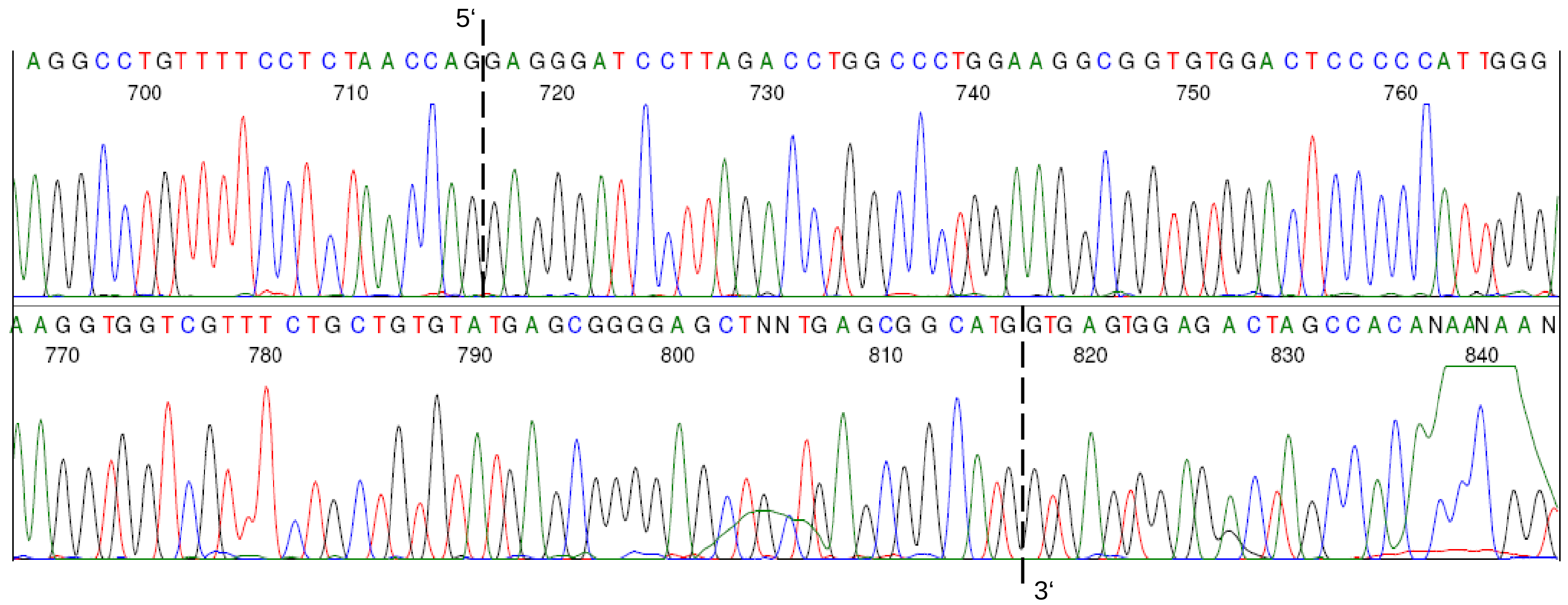

Exon 19

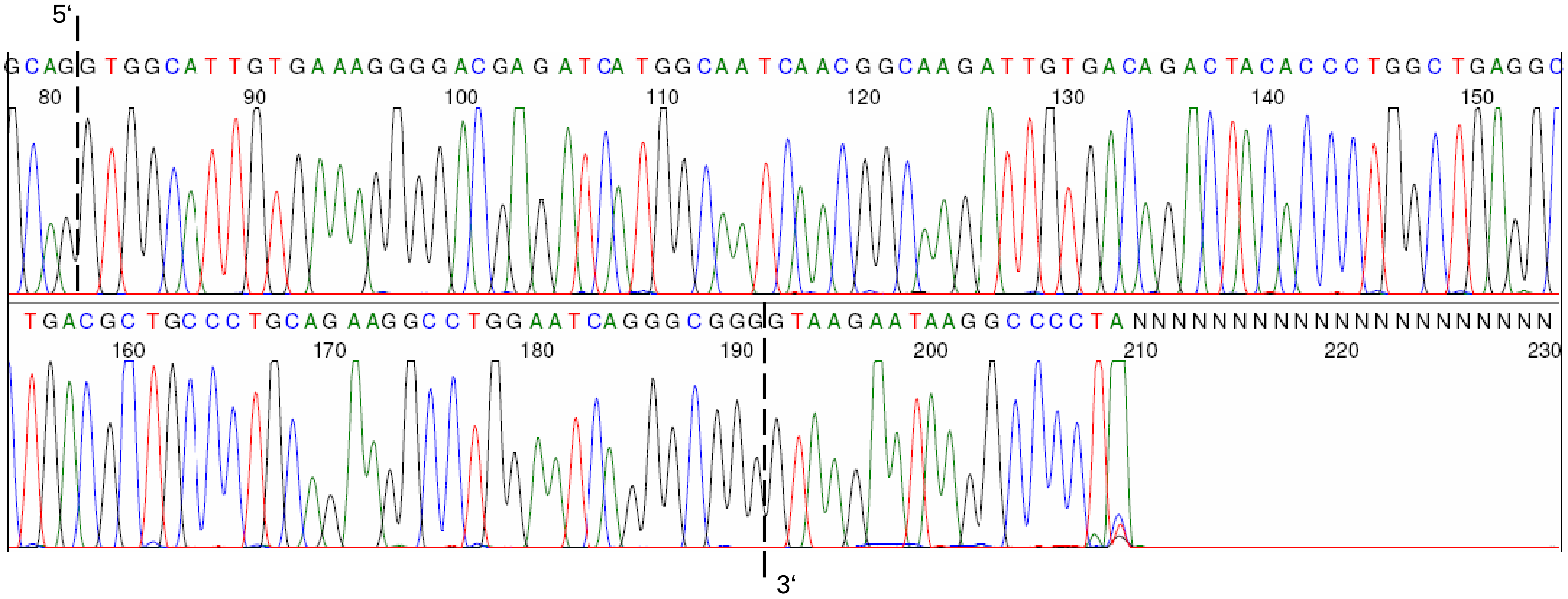

Exon 20

Exon G

Exon 21

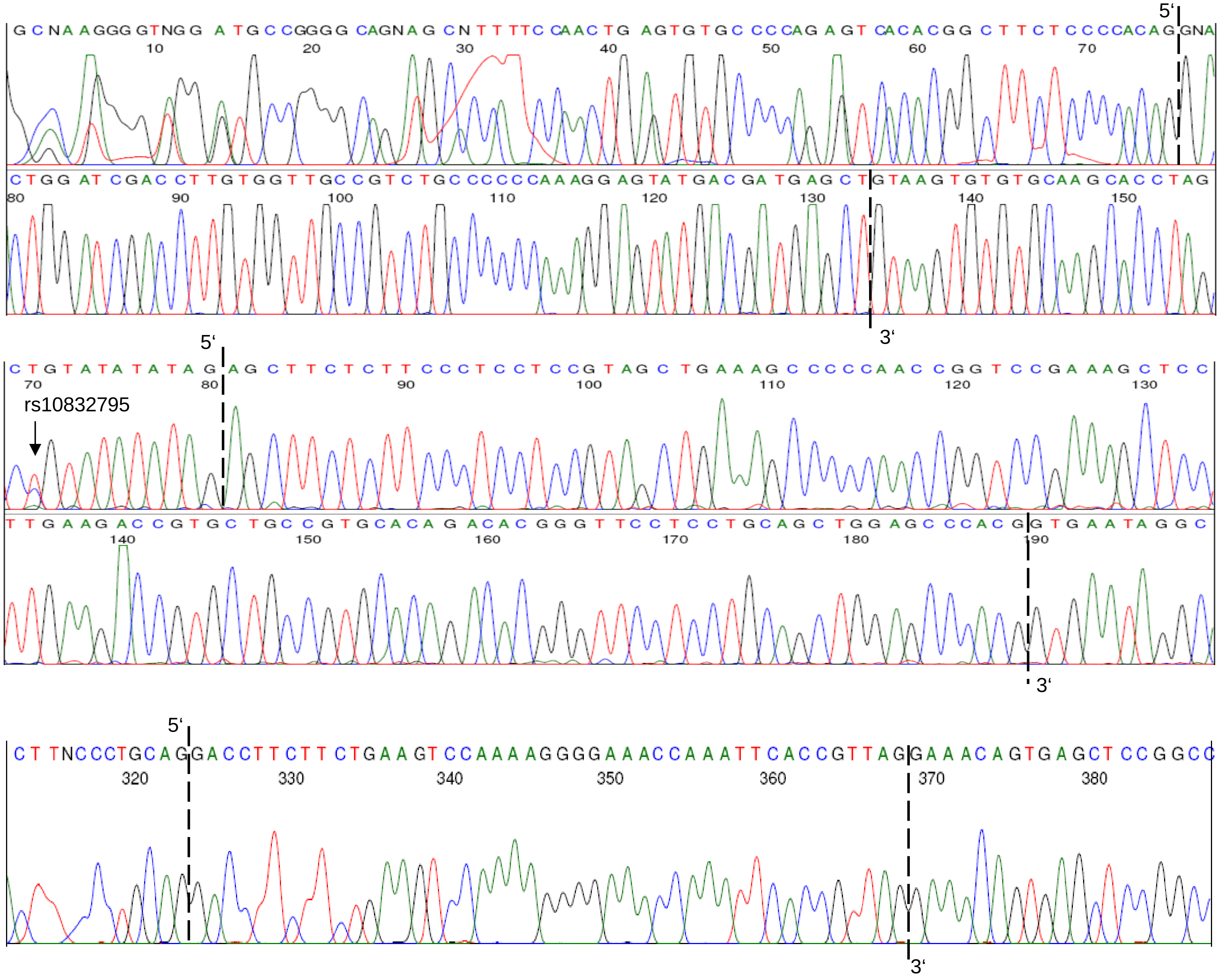

Supplement: Additional data file 7 — Results of mutation screening in patient 1881 in the genes MYO7A (USH1B) and USH1C (no mutations found). [file gb-2007-8-4-r47-S7.pdf]
