# Supplementary material for: Deafblindness in French Canadians from Quebec: a predominant founder mutation in the USH1C gene provides the first genetic link with the Acadian population
Source: Genome Biol. 2007 Apr 3;8(4):R47. doi: 10.1186/gb-2007-8-4-r47 (PMC1895989; doi:10.1186/gb-2007-8-4-r47)

Deafblindness in French Canadians from Quebec: A predominant founder mutation in the *USH1C* gene provides the first genetic link with the Acadian population

Ebermann et al.

RAW DATA

	page
Haplotypes	
USH1C haplotypes	2 – 23
USH1D haplotypes	24 – 29
USH1 mutations	30 – 36
Genotyping of healthy French Canadian Controls	
Controls for c.216G>A (*USH1C*)	37 – 40
Controls for c.238-239insC (*USH1C*)	41 – 58
Controls for c.496+1G>T (*USH1C*)	59 – 60
Controls for p.R155X (*USH1C*)	61 – 62
Controls for c.748-759+5del (*USH1C*)	63 – 64
Controls for IVS45-9G>A (*CDH23*)	65 – 73
Controls for p.R736X (*CDH23*)	74 – 82
Controls for p.A457V (*MYO7A*)	83 – 91
Controls for p.Q815X (*MYO7A*)	92 – 93
Controls for p.A123D (*USH3A*)	94 – 102
Mutation screening in USH1 genes in patient 1881	103 – 204

Patient 1881, *CDH23* (*USH1D*)

Exon 1

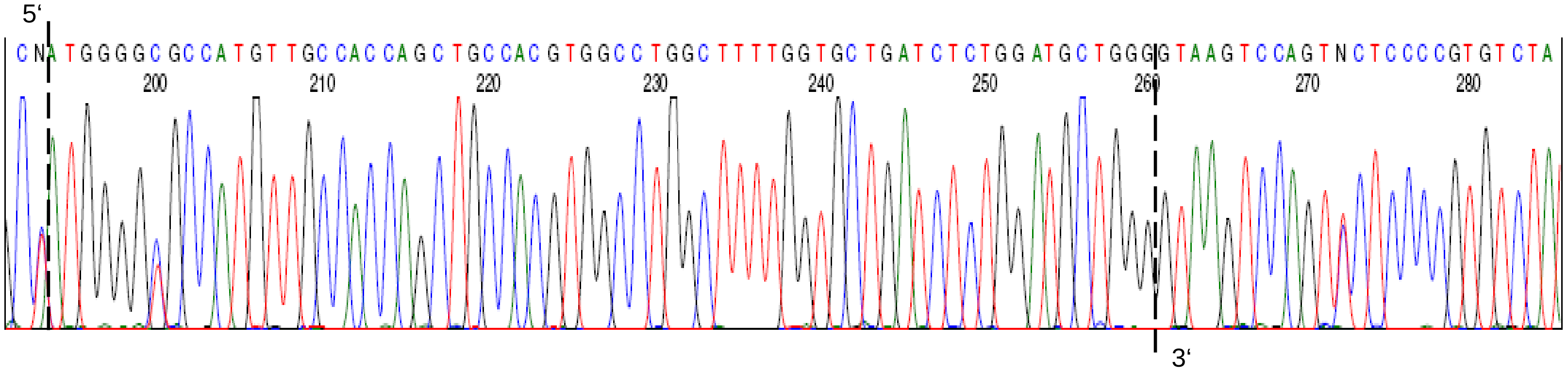

Exon 2

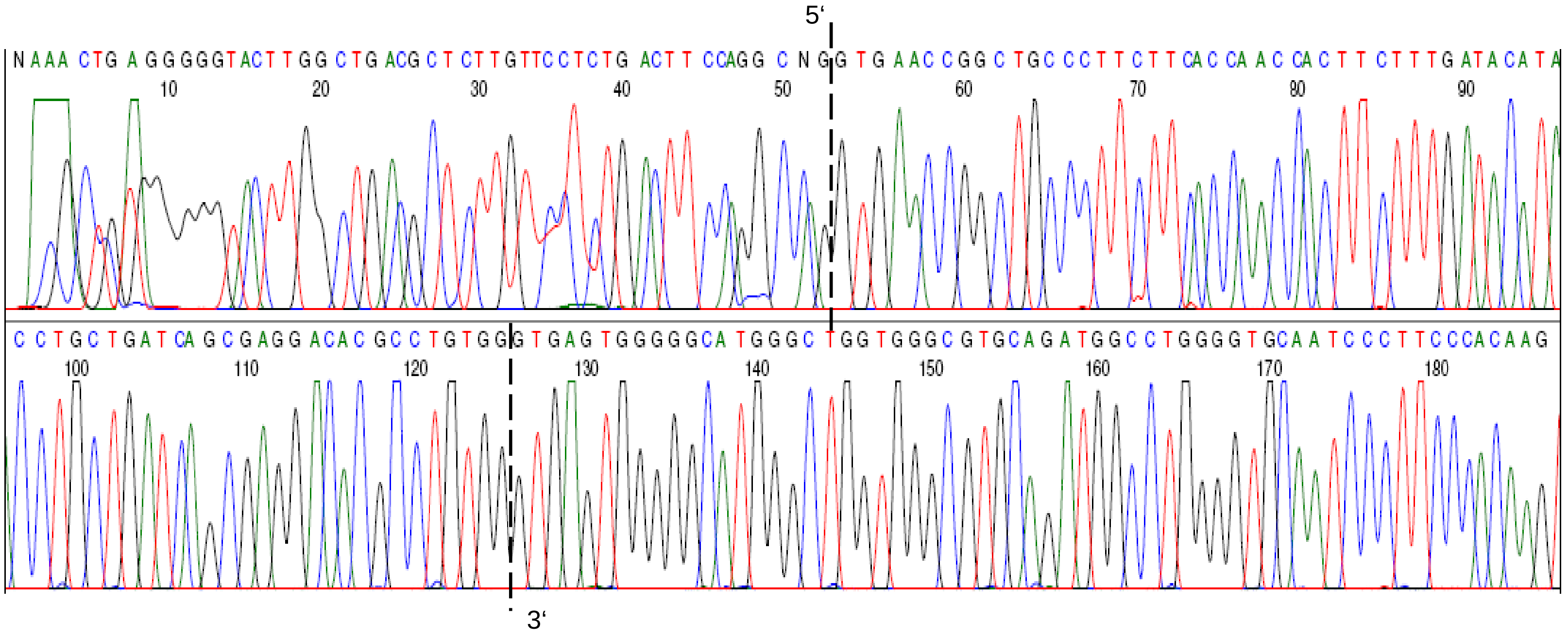

Exon 3

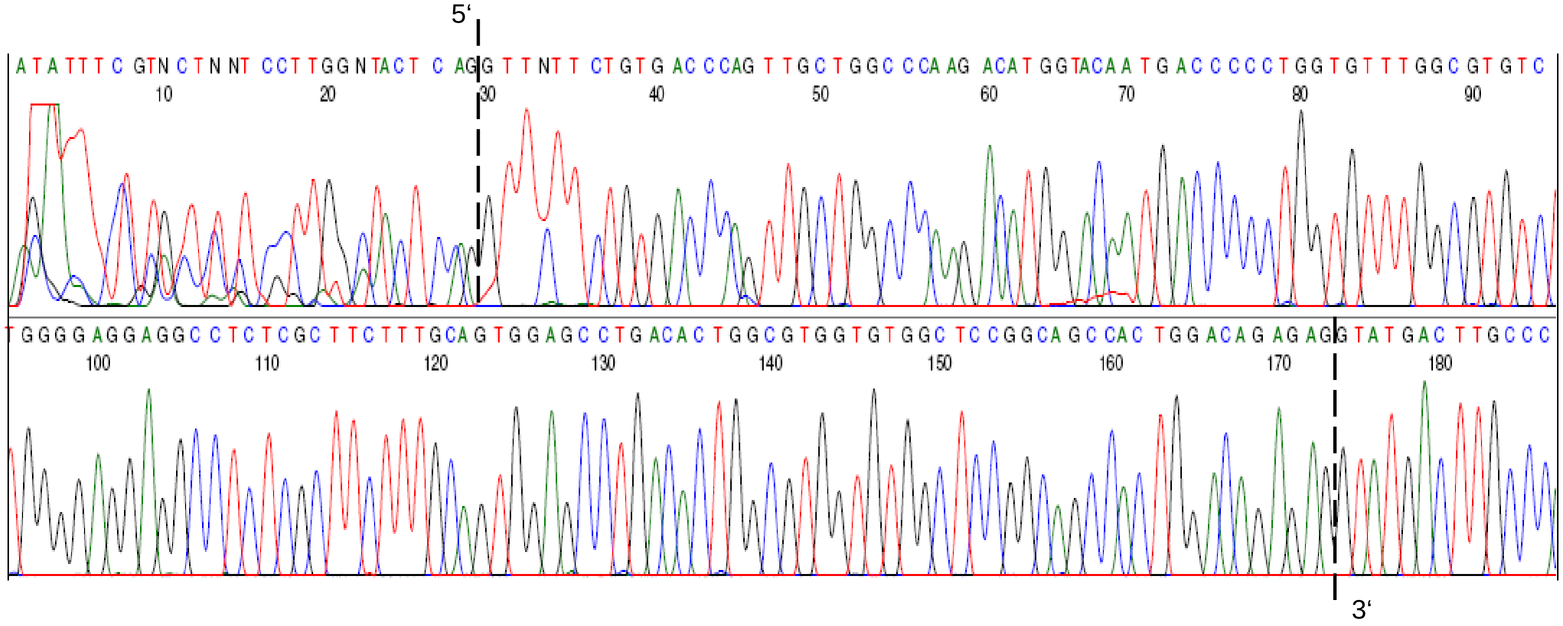

Exon 4

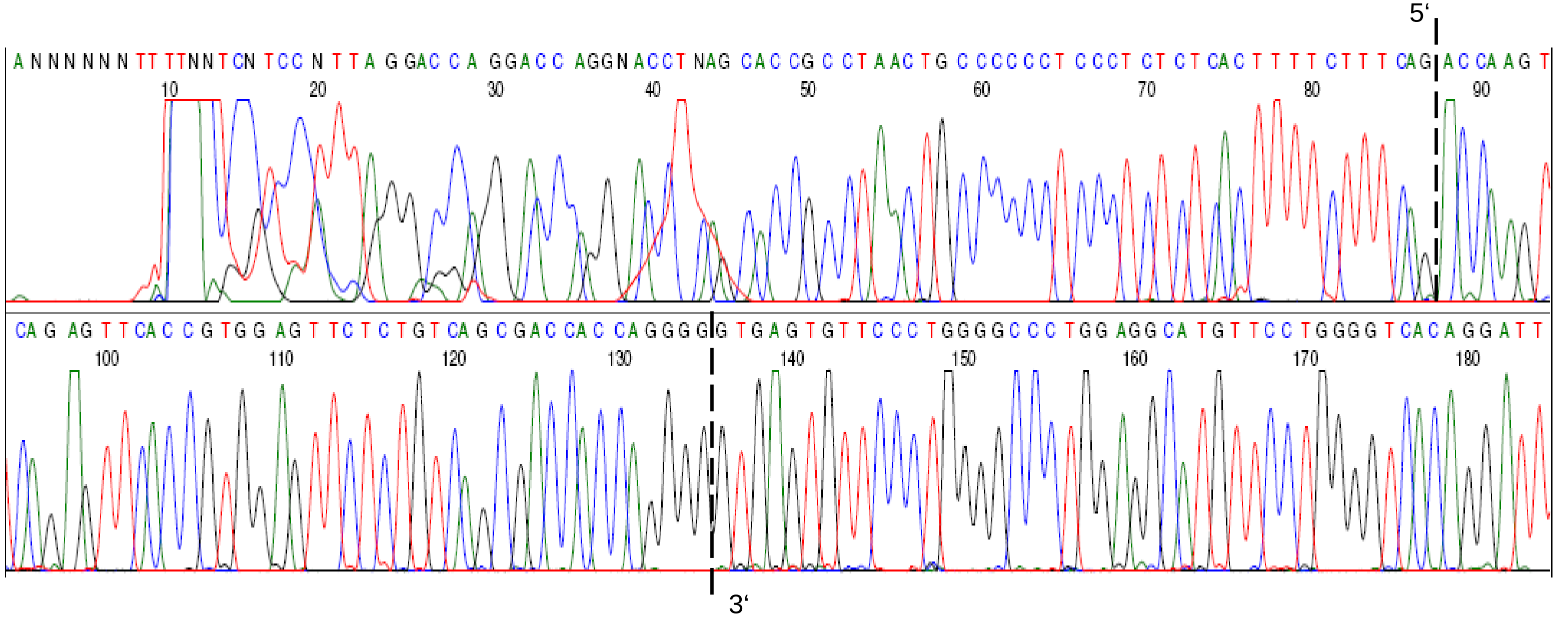

Exon 5

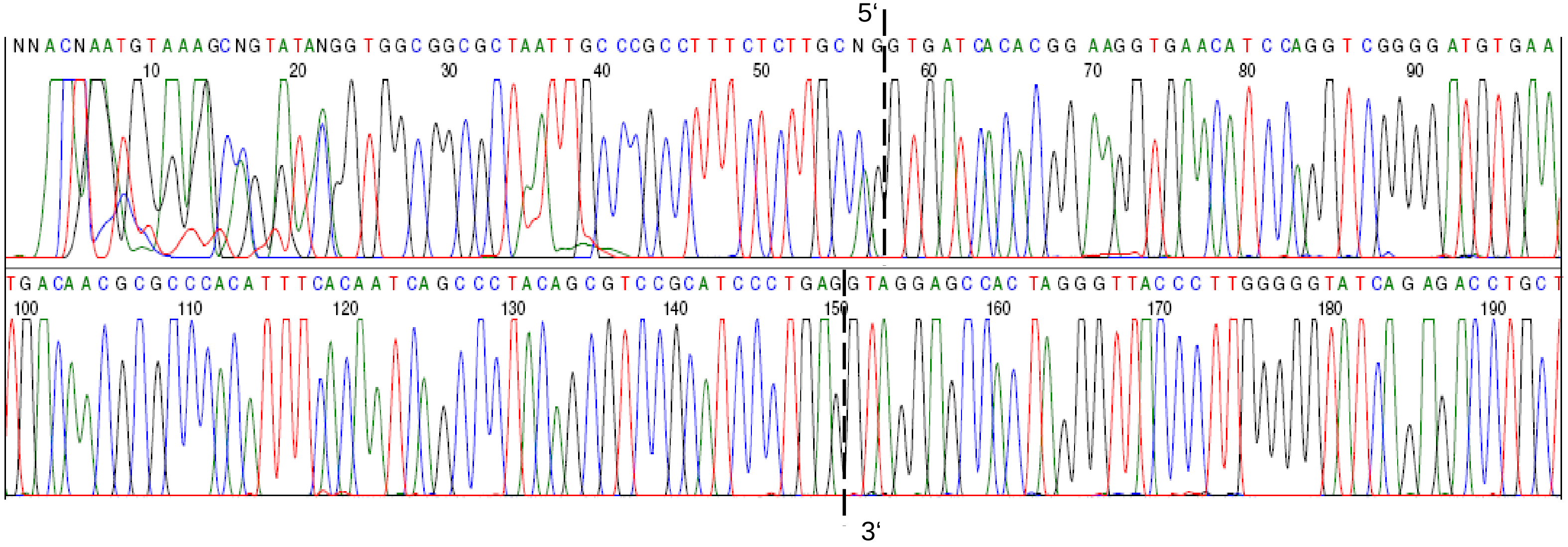

Exon 6

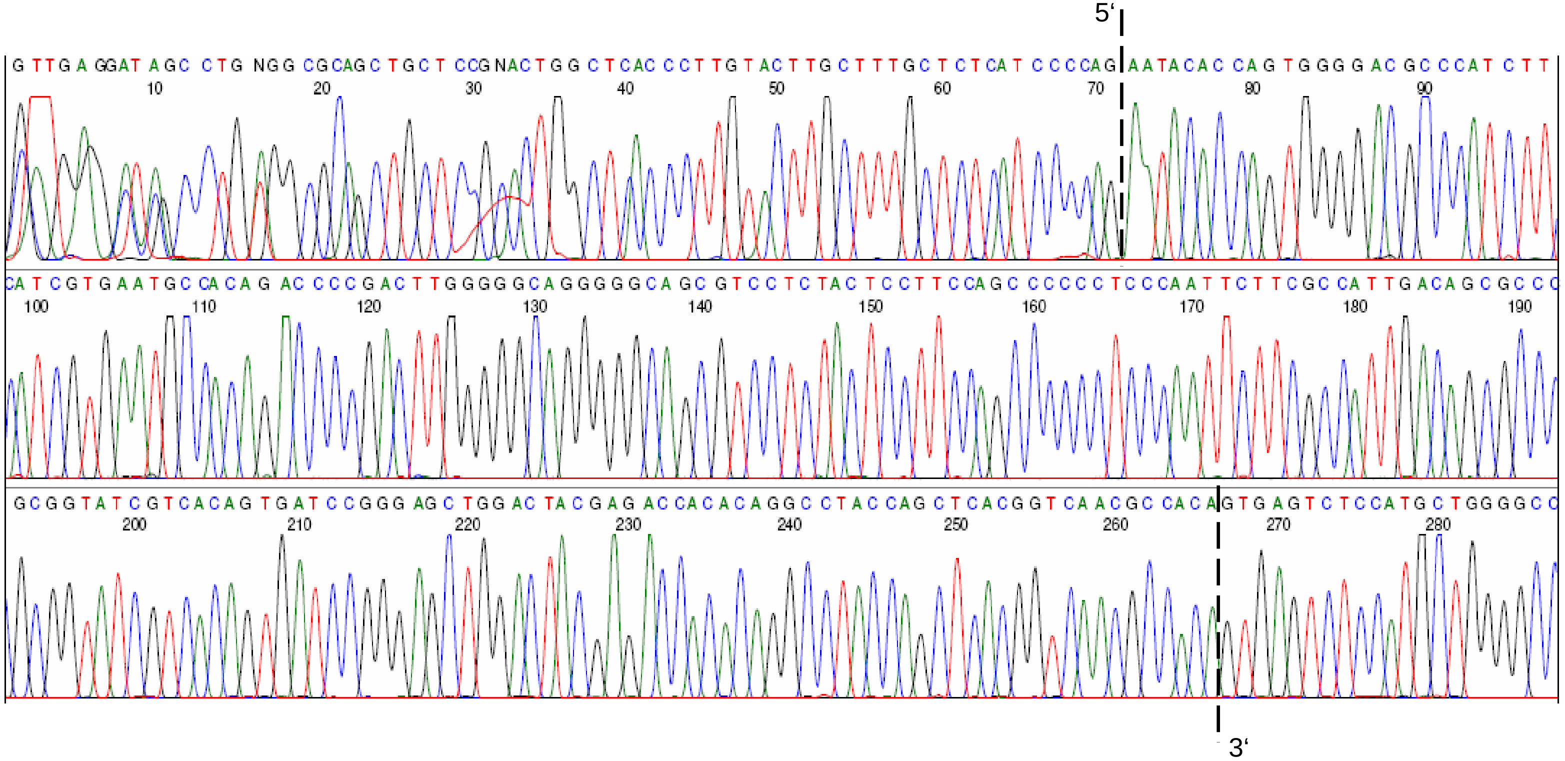

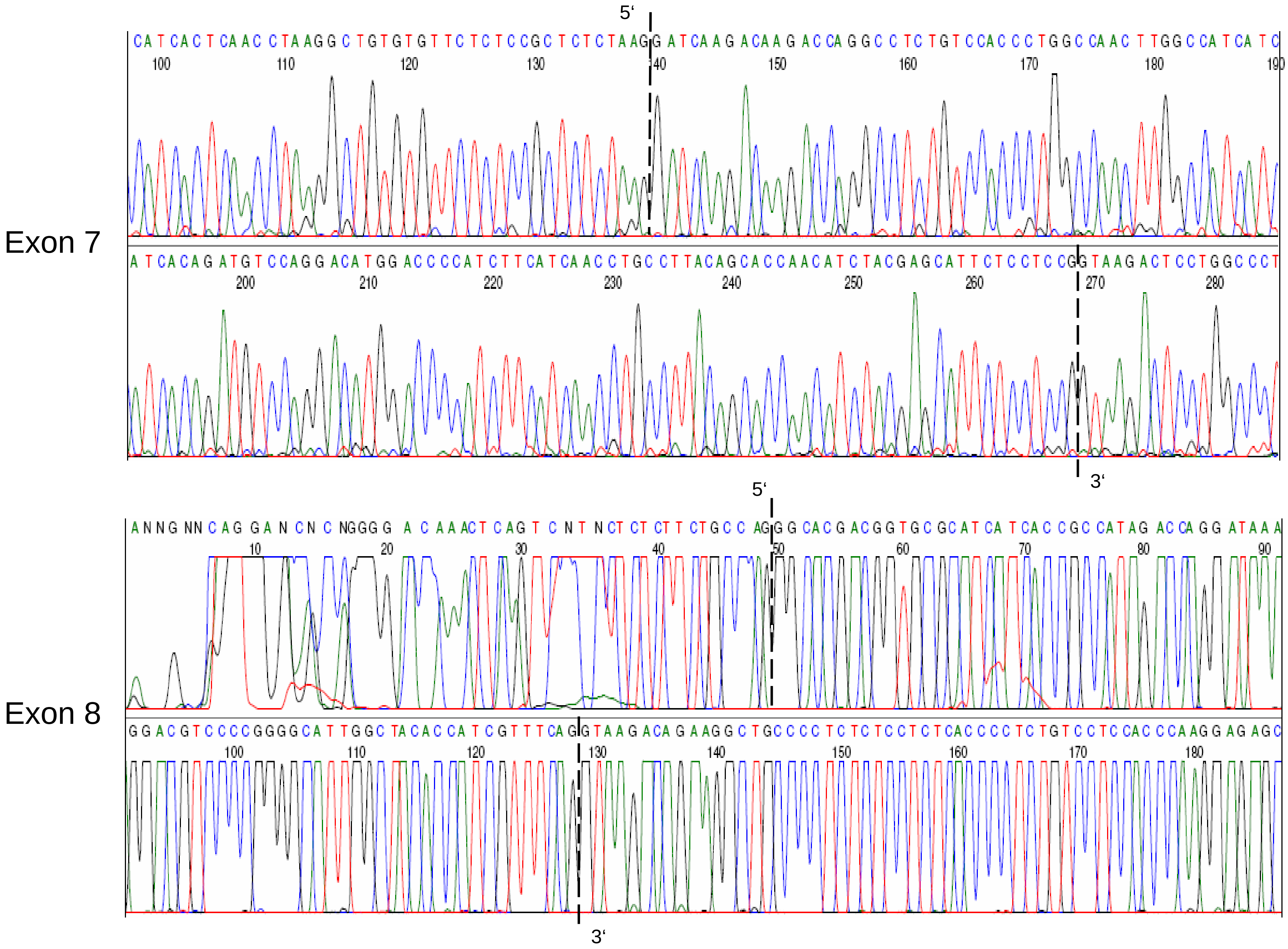
5‘
3‘
Exon 7
5‘
3‘
Exon 8

Exon 9

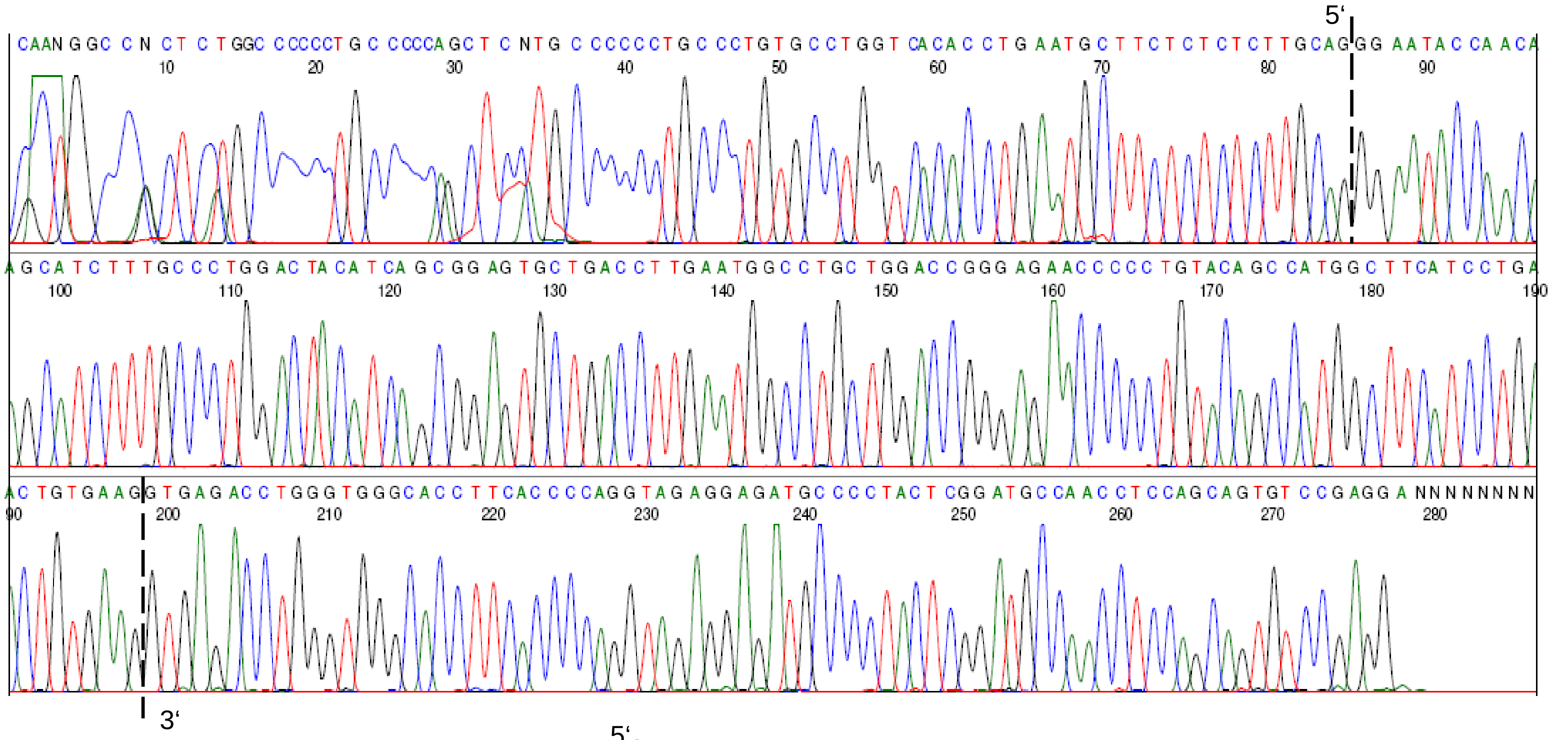

Exon 10

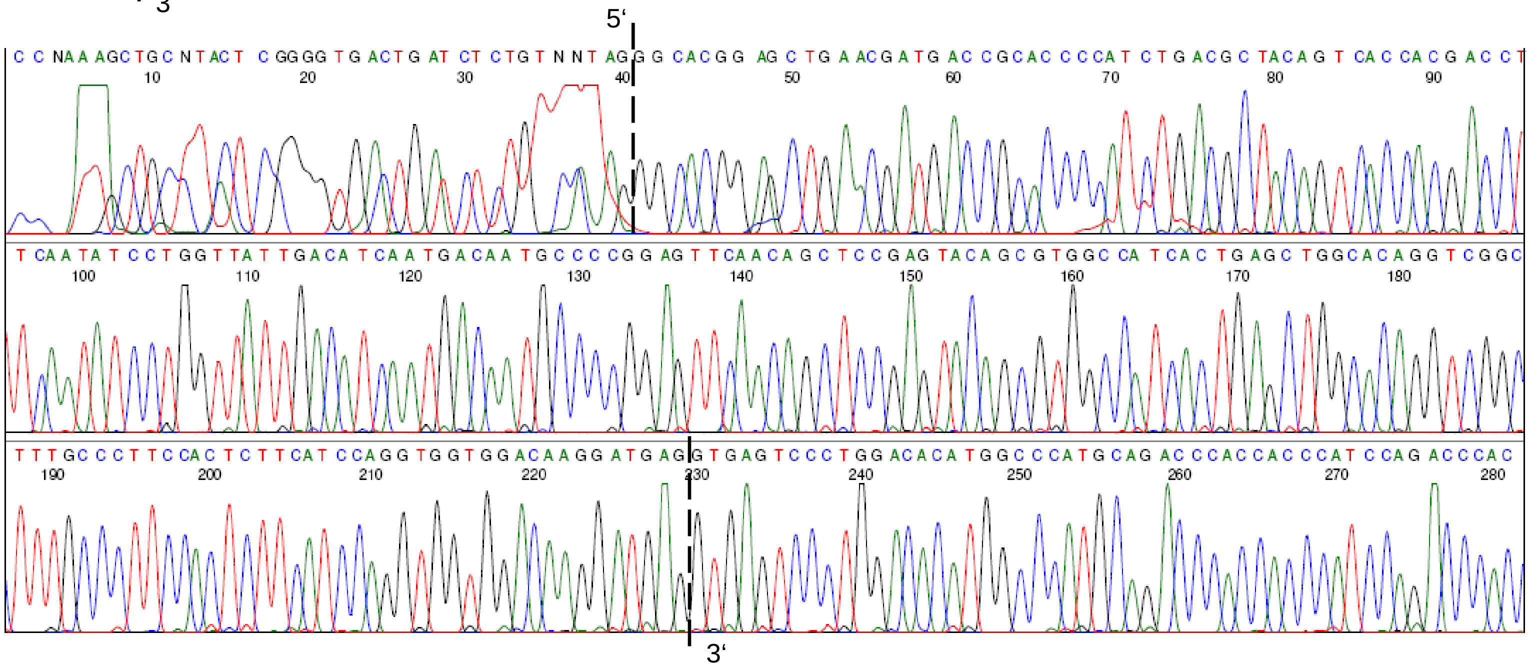

Exon 11

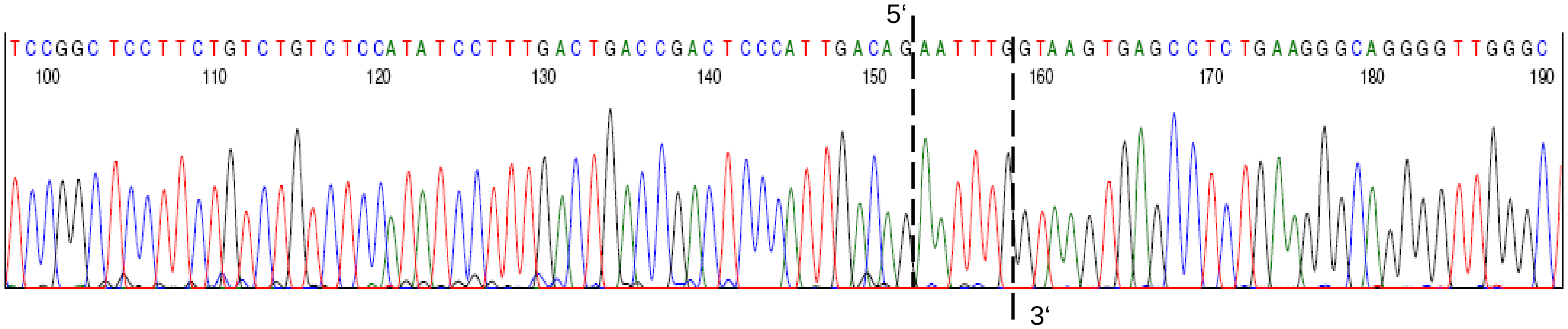

Exon 12

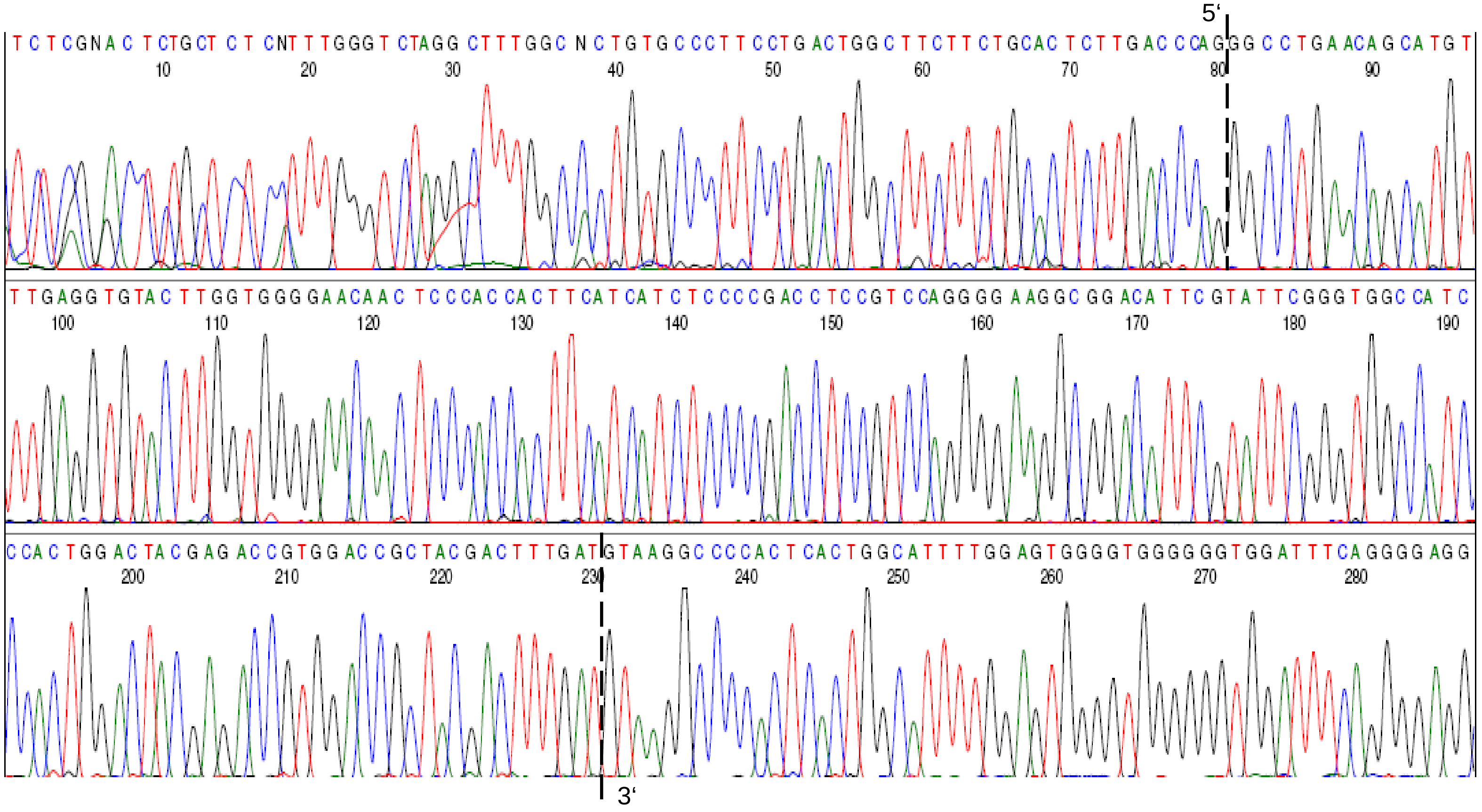

Exon 13

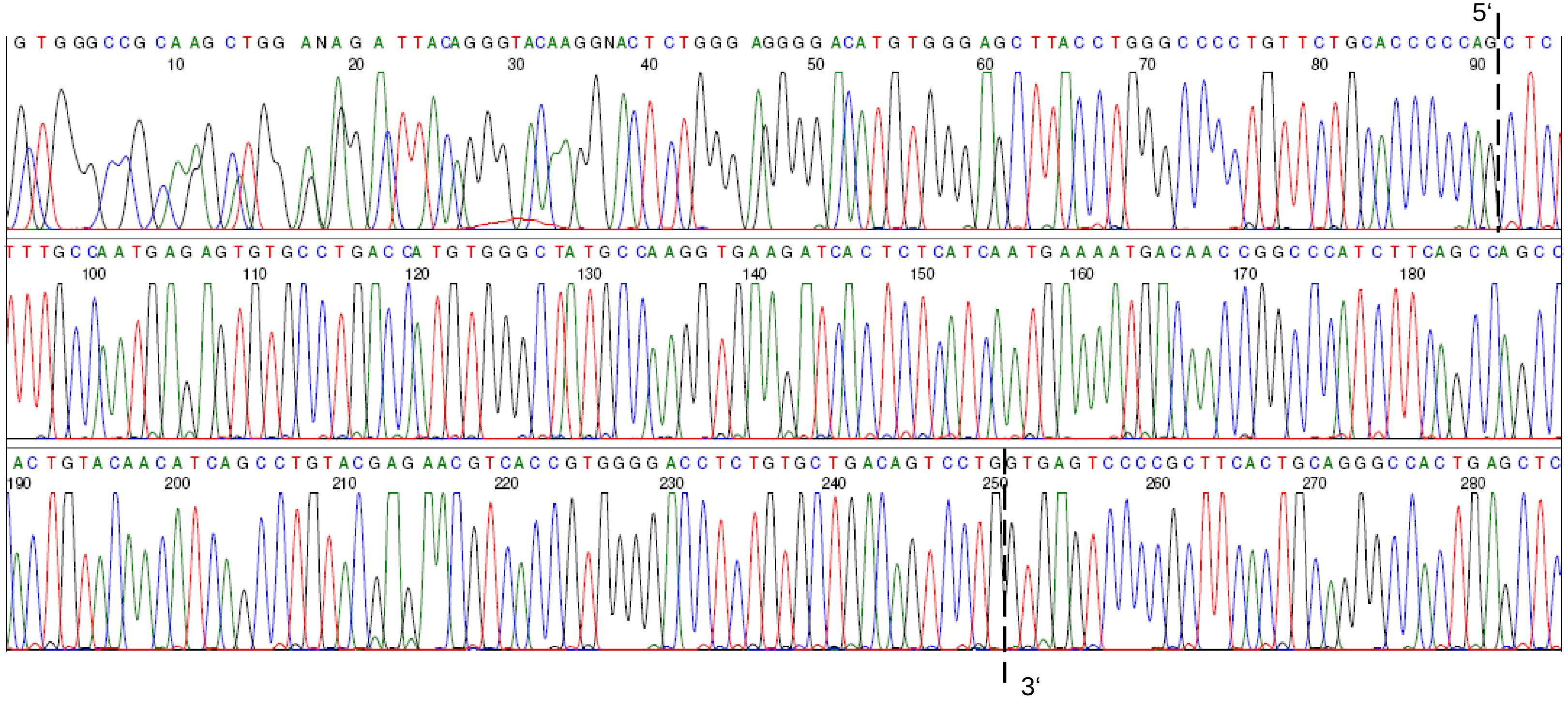

Exon 14

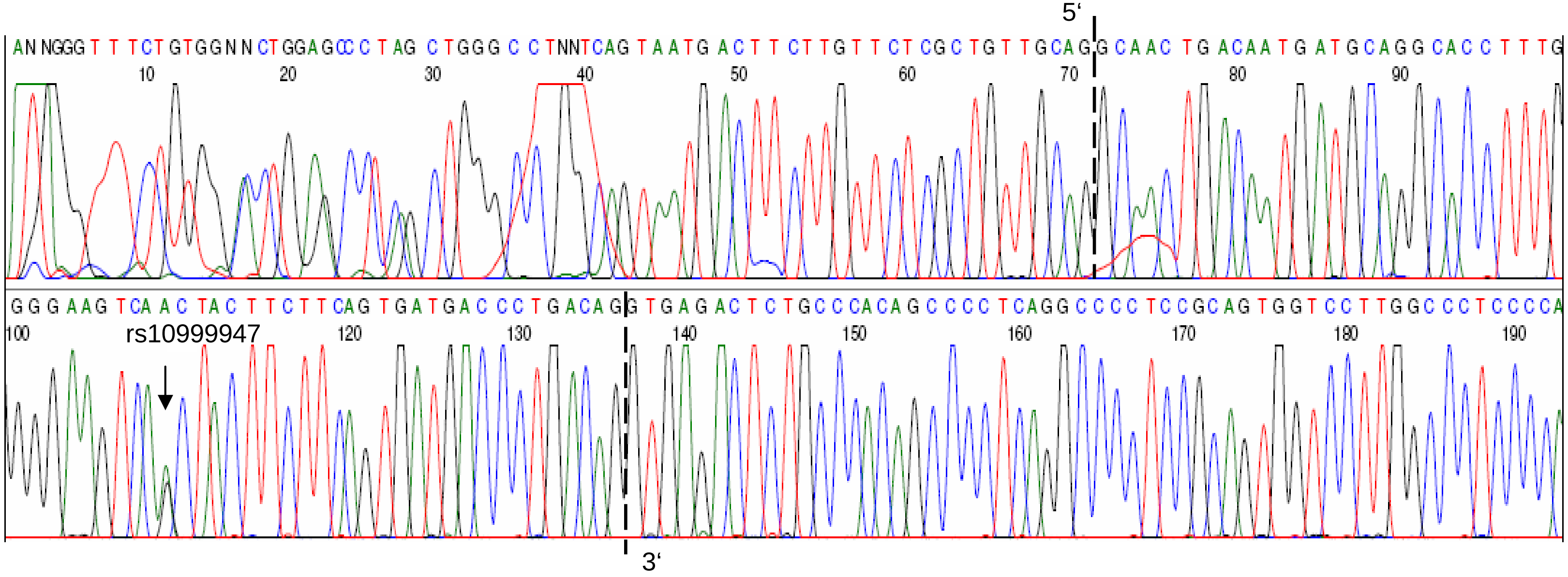

Exon 15

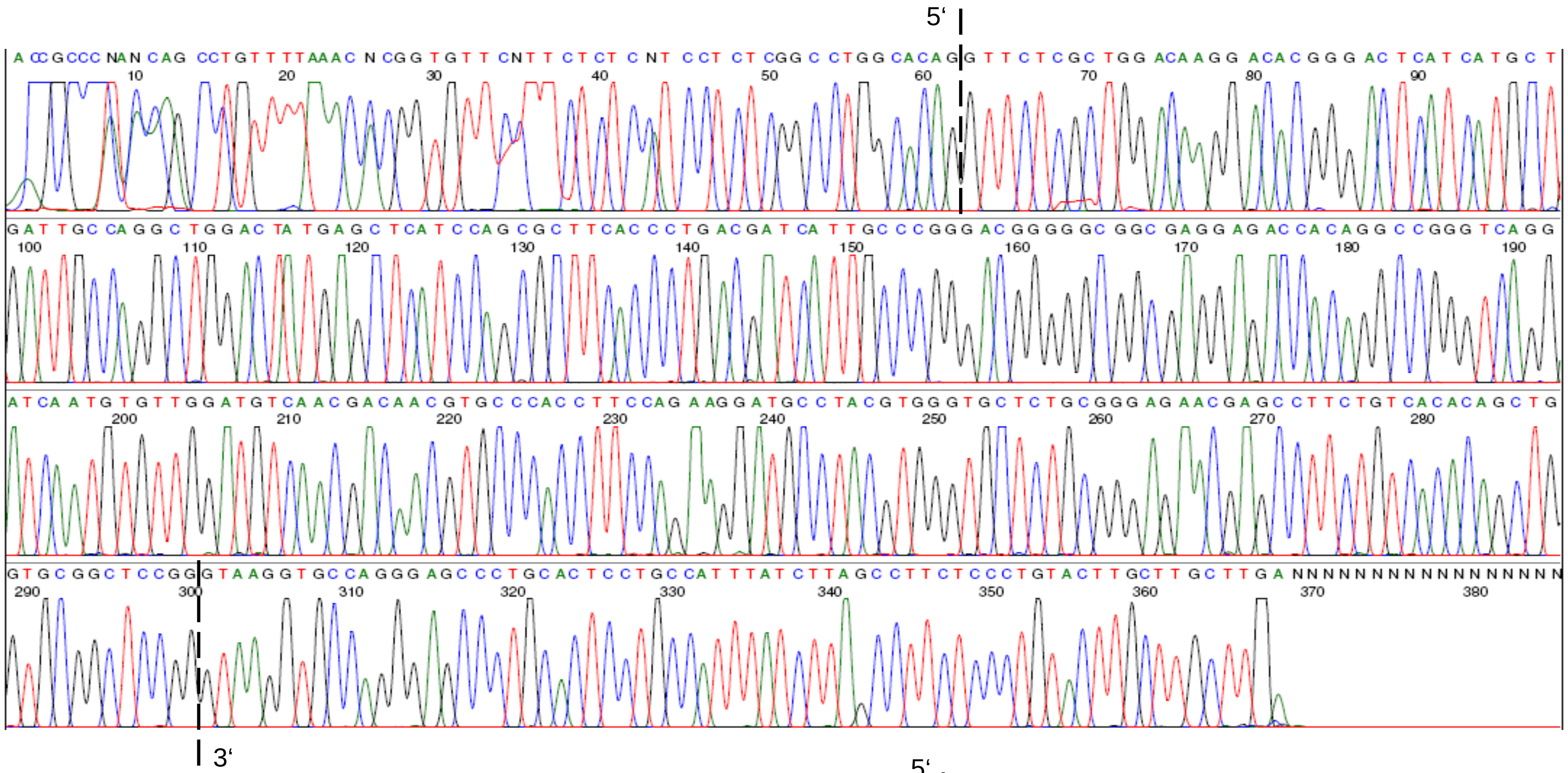

Exon 16

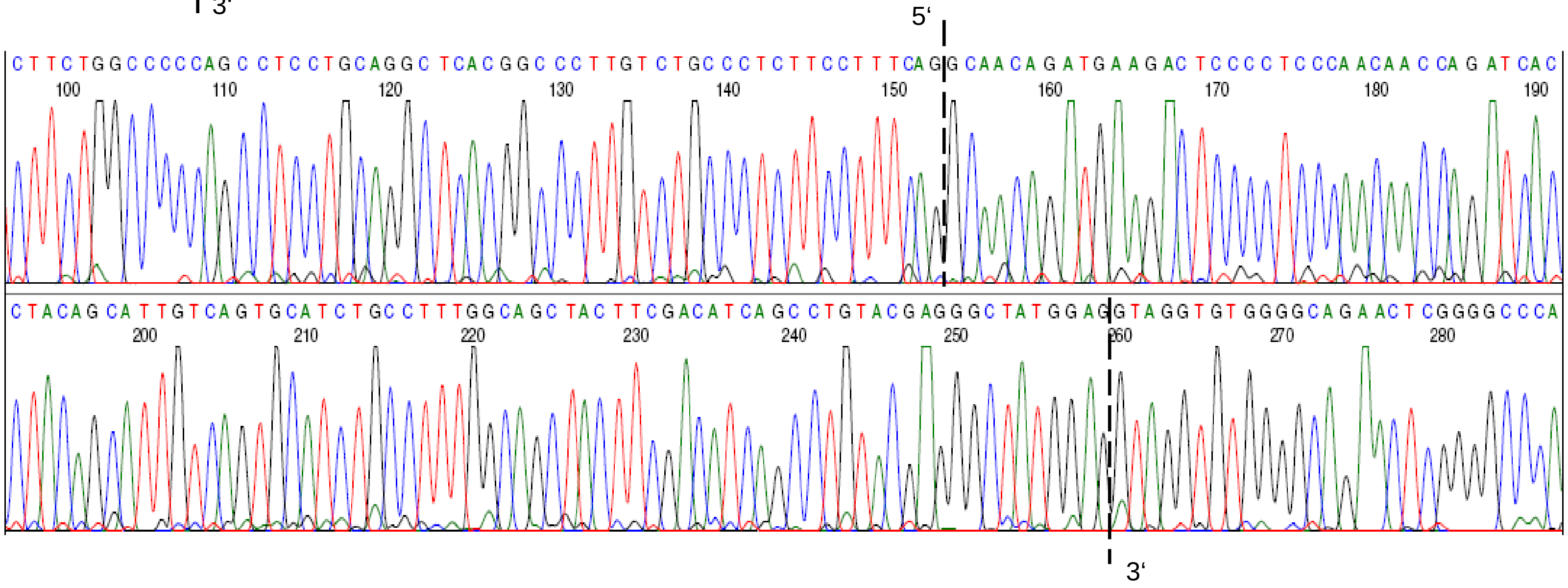

Exon 17

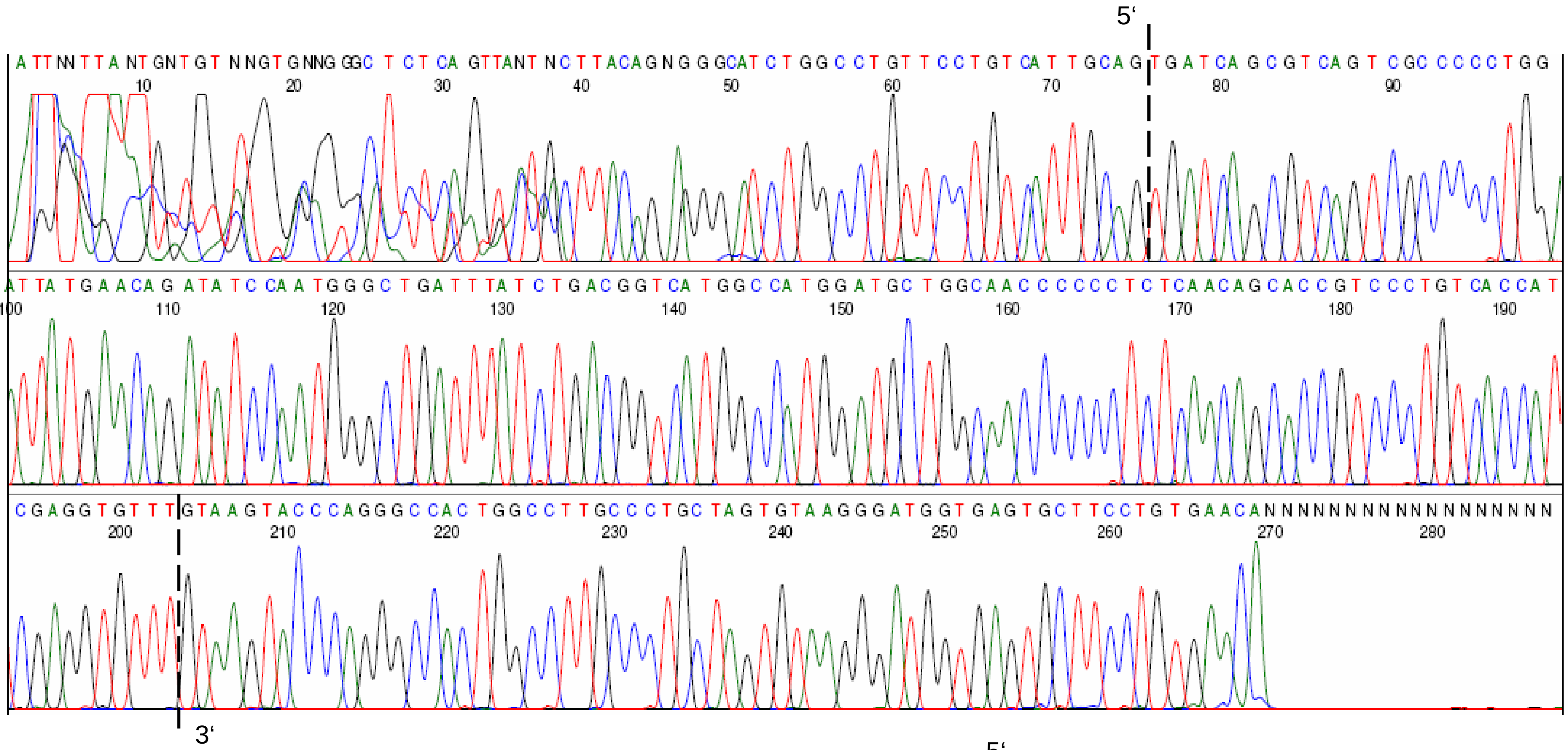

Exon 18

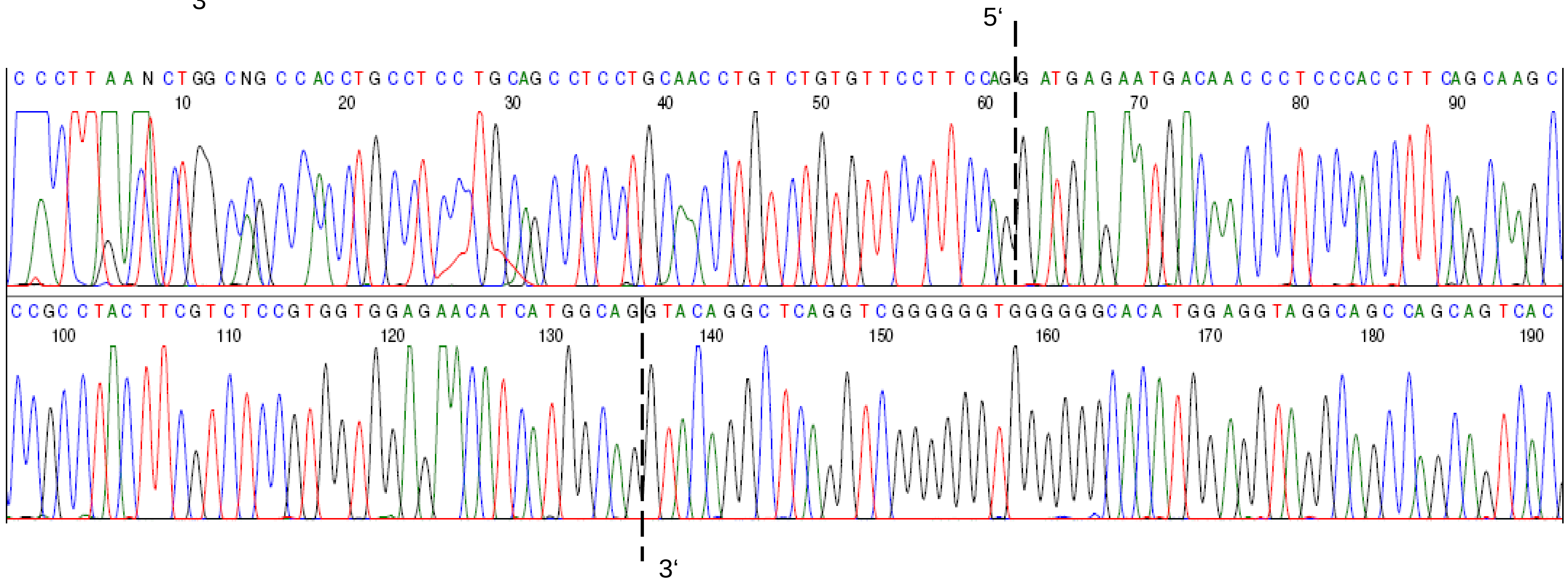

Exon 19

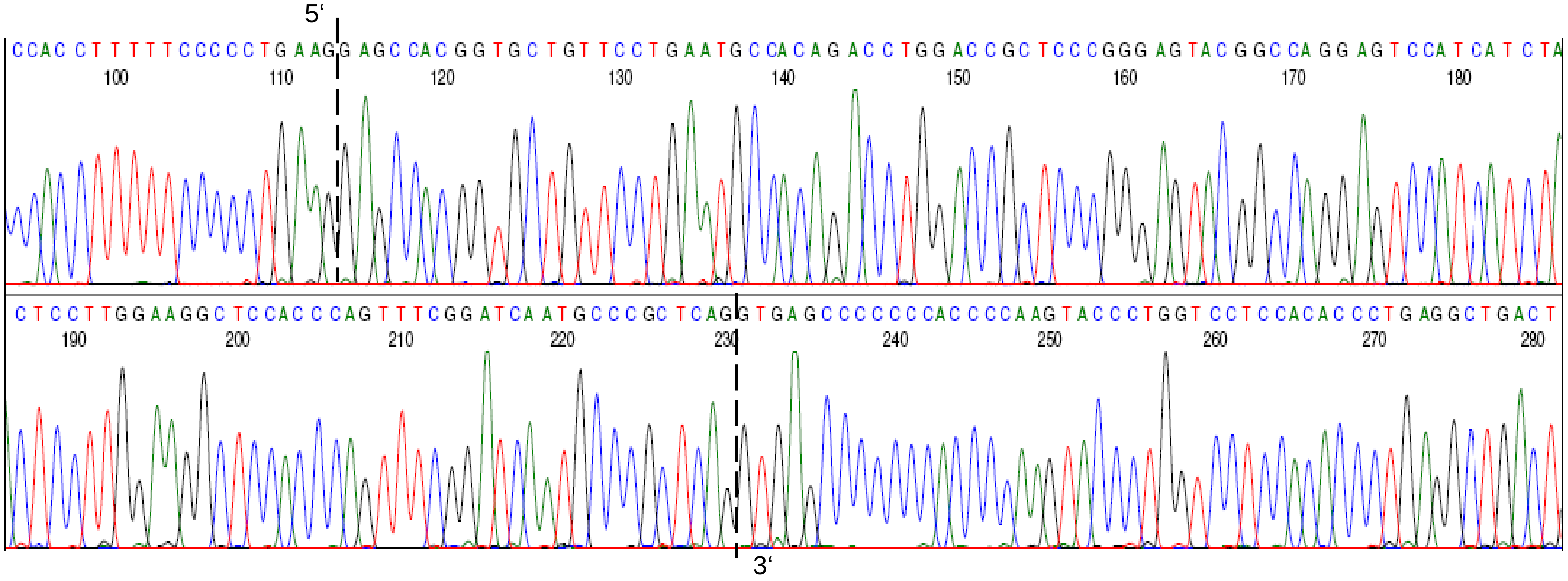

Exon 20

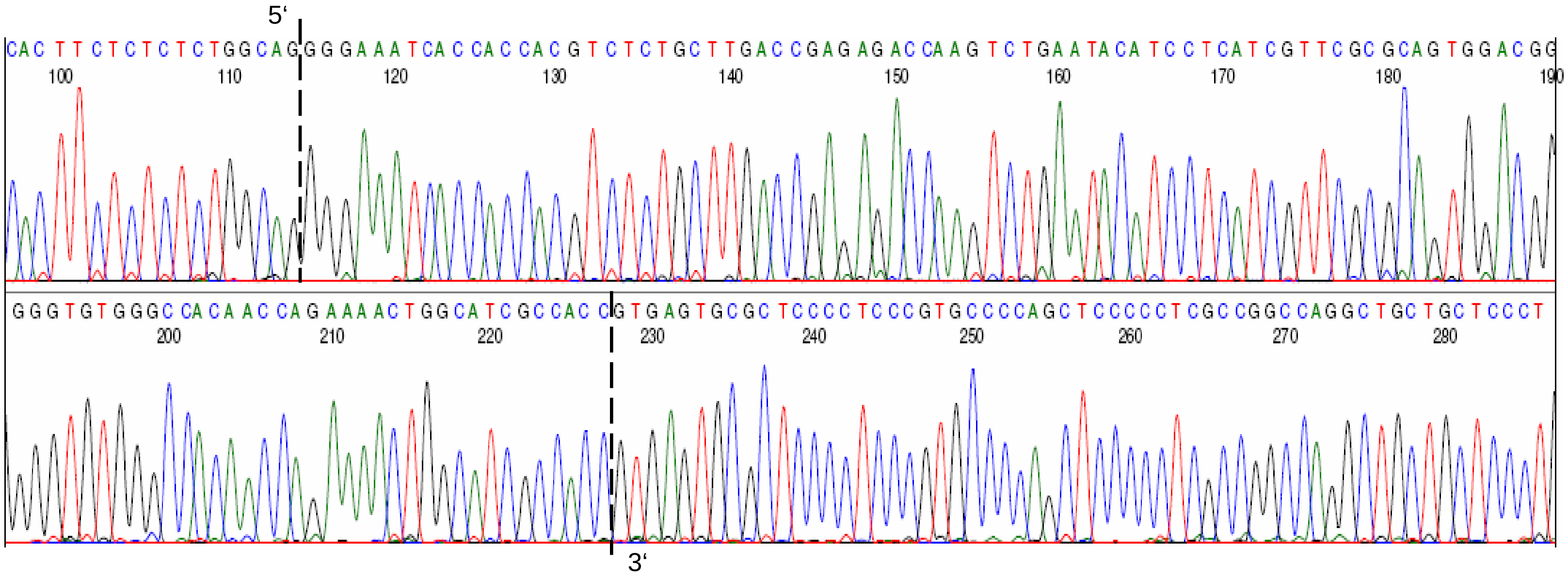

Exon 21

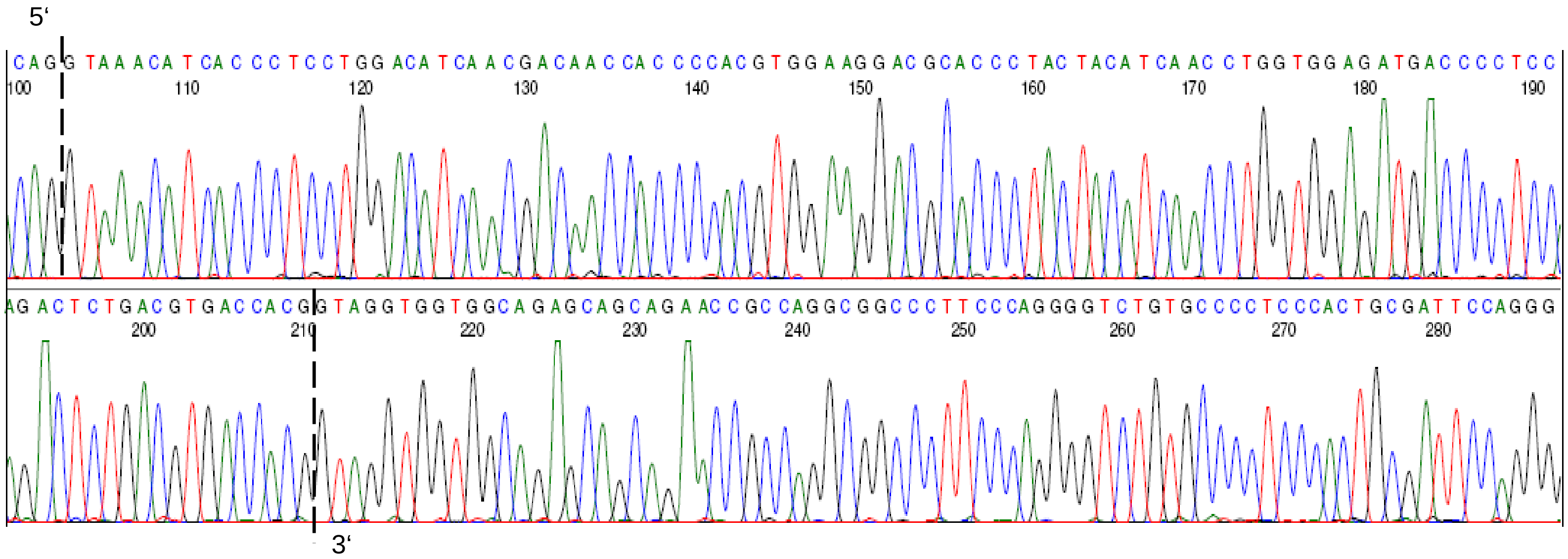

Exon 22

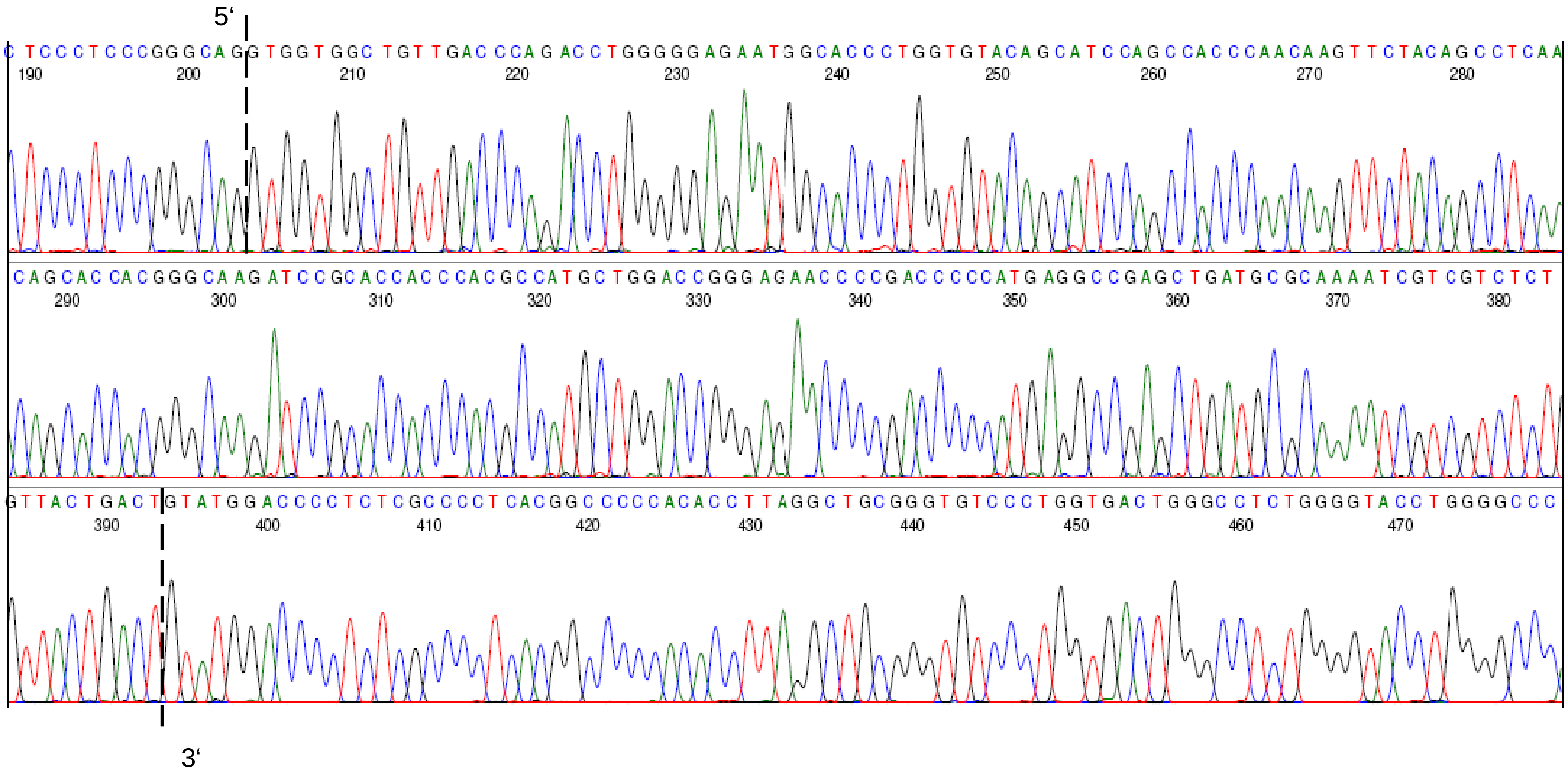

Exon 23

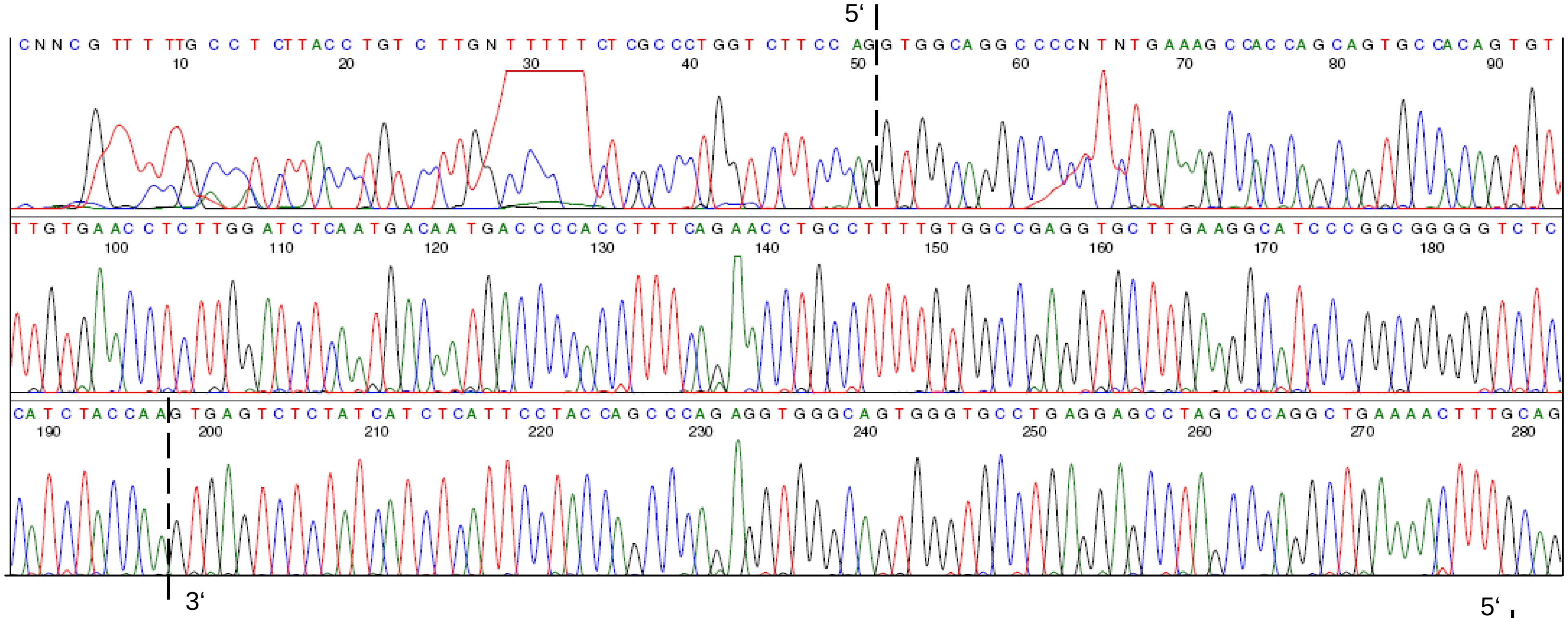

Exon 24

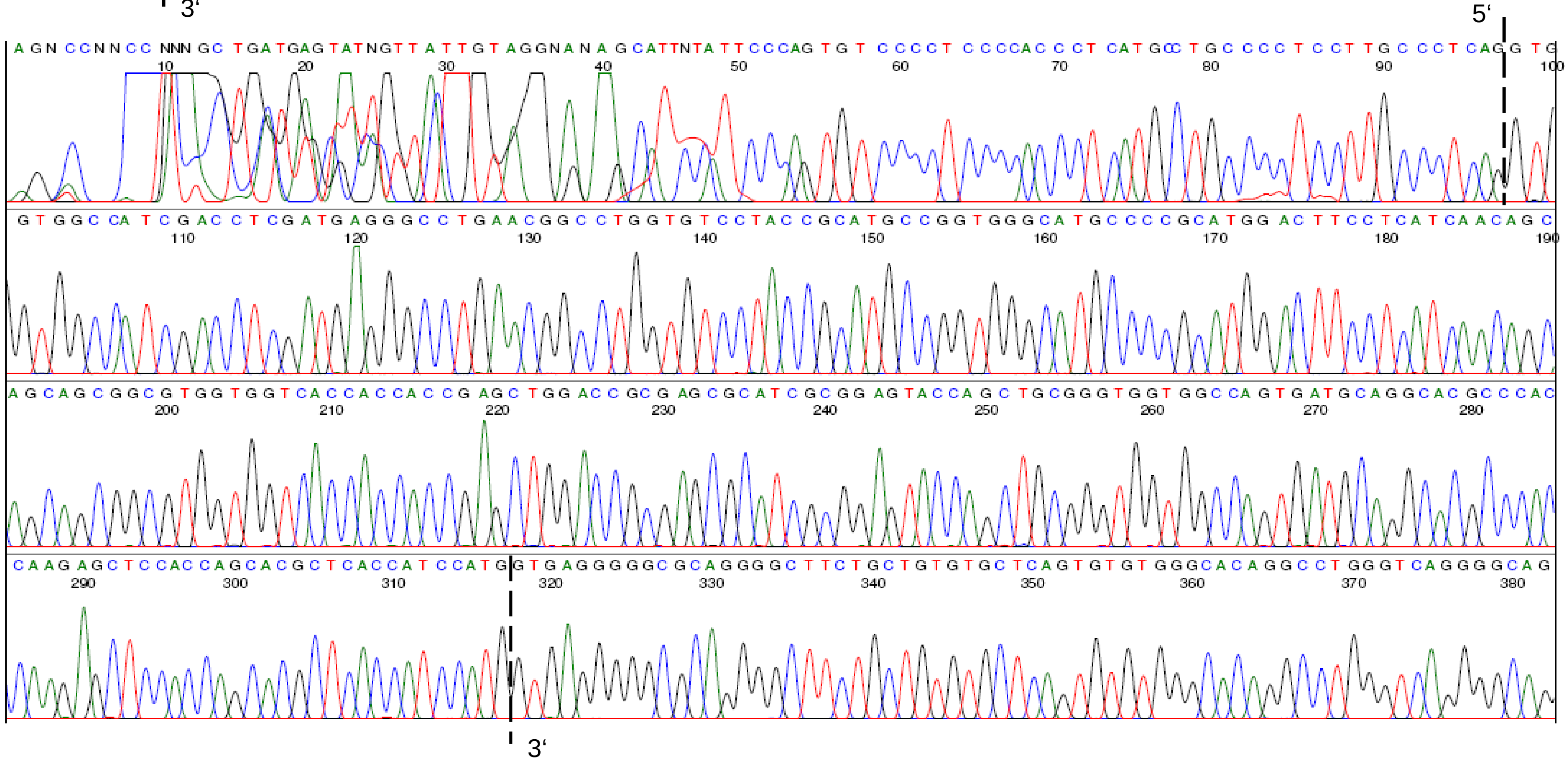

Exon 25

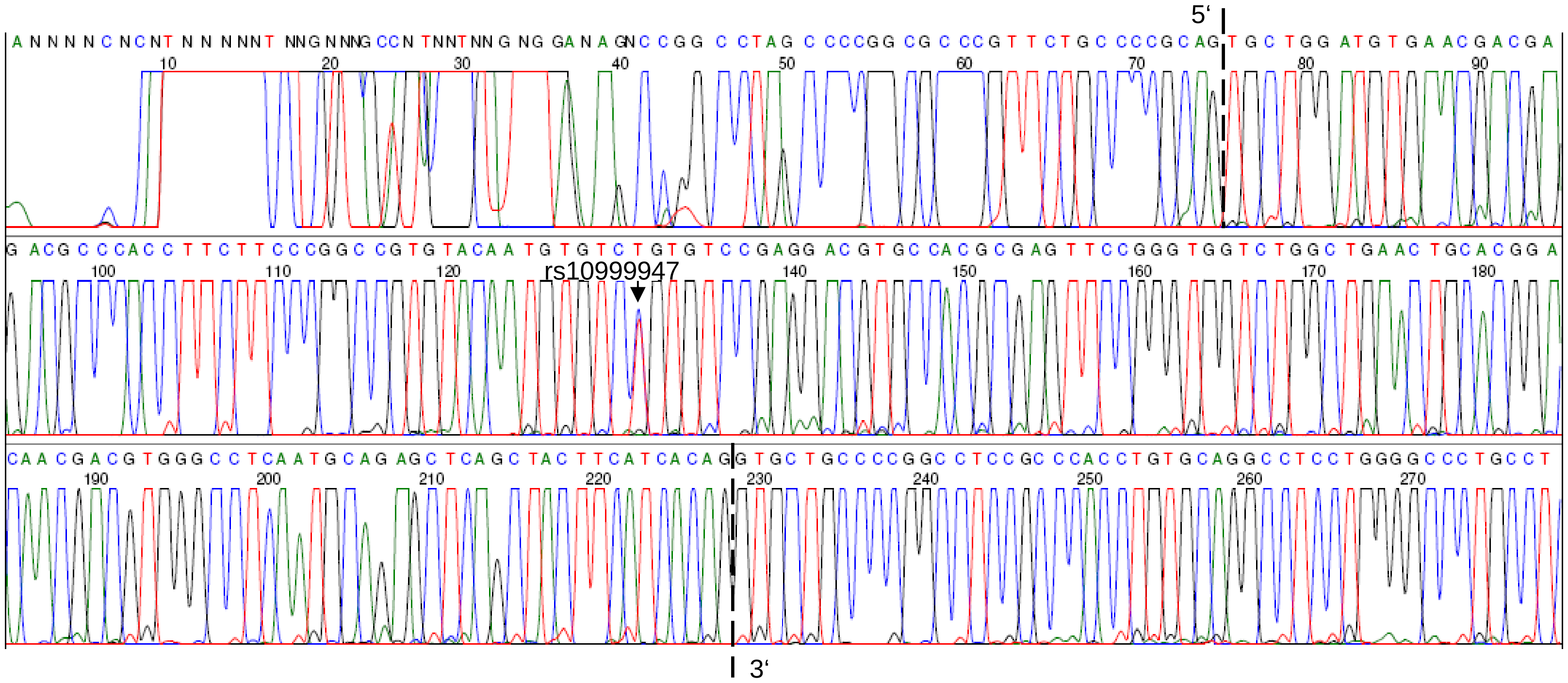

Exon 26

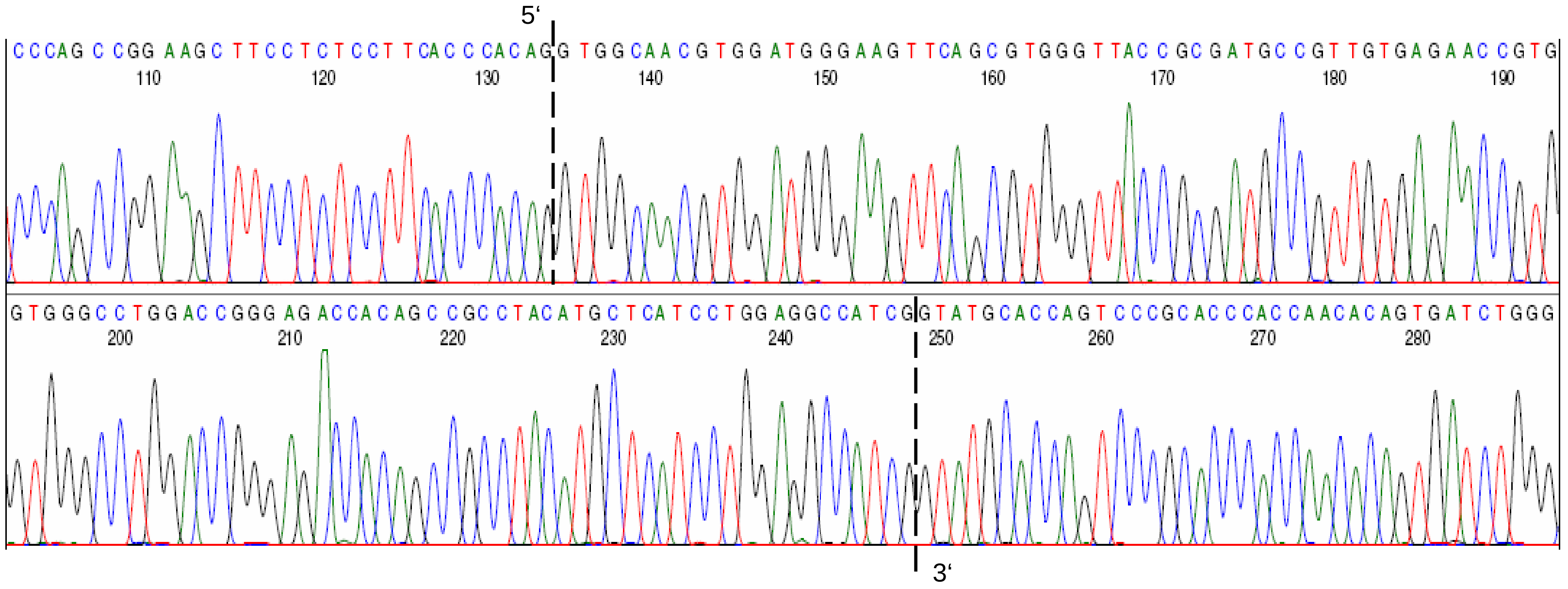

Exon 27

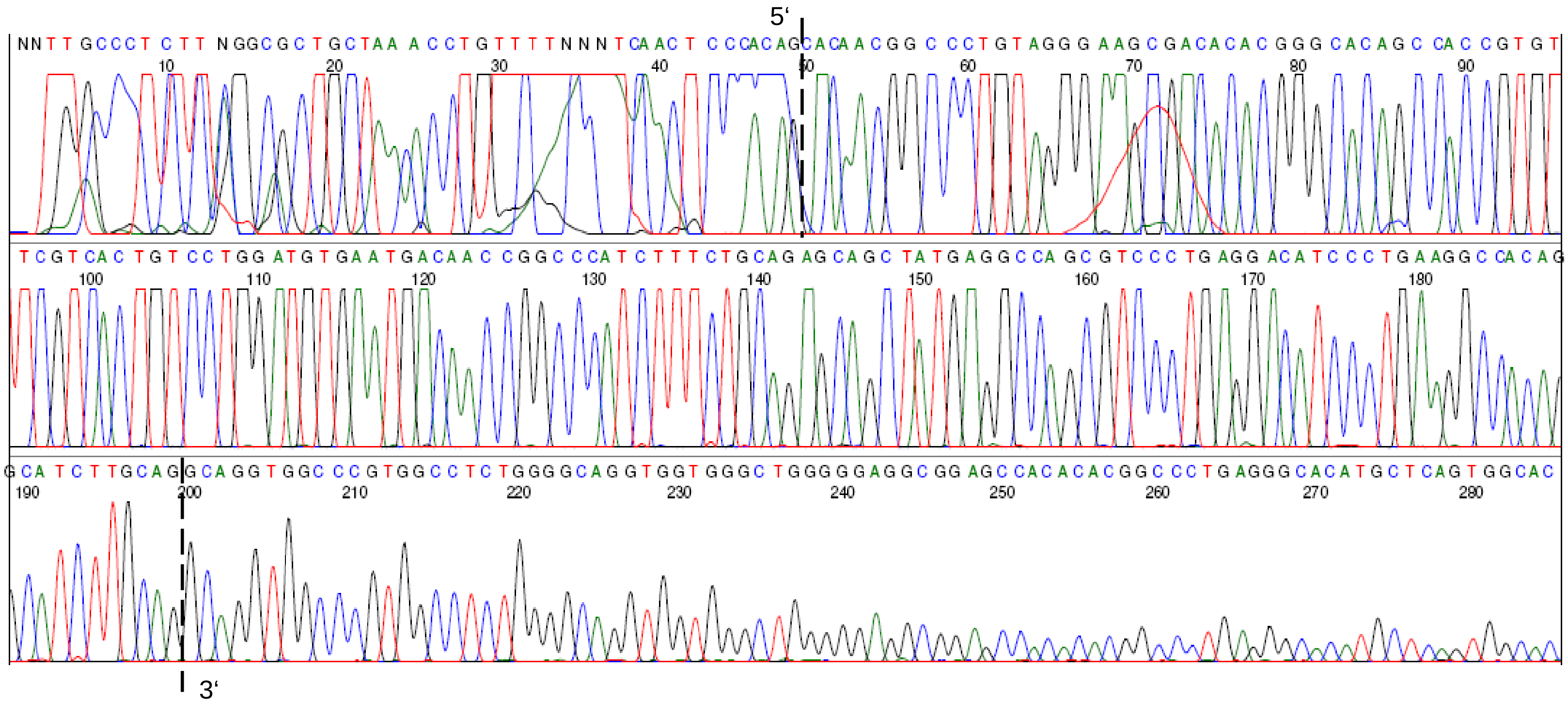

Exon 28

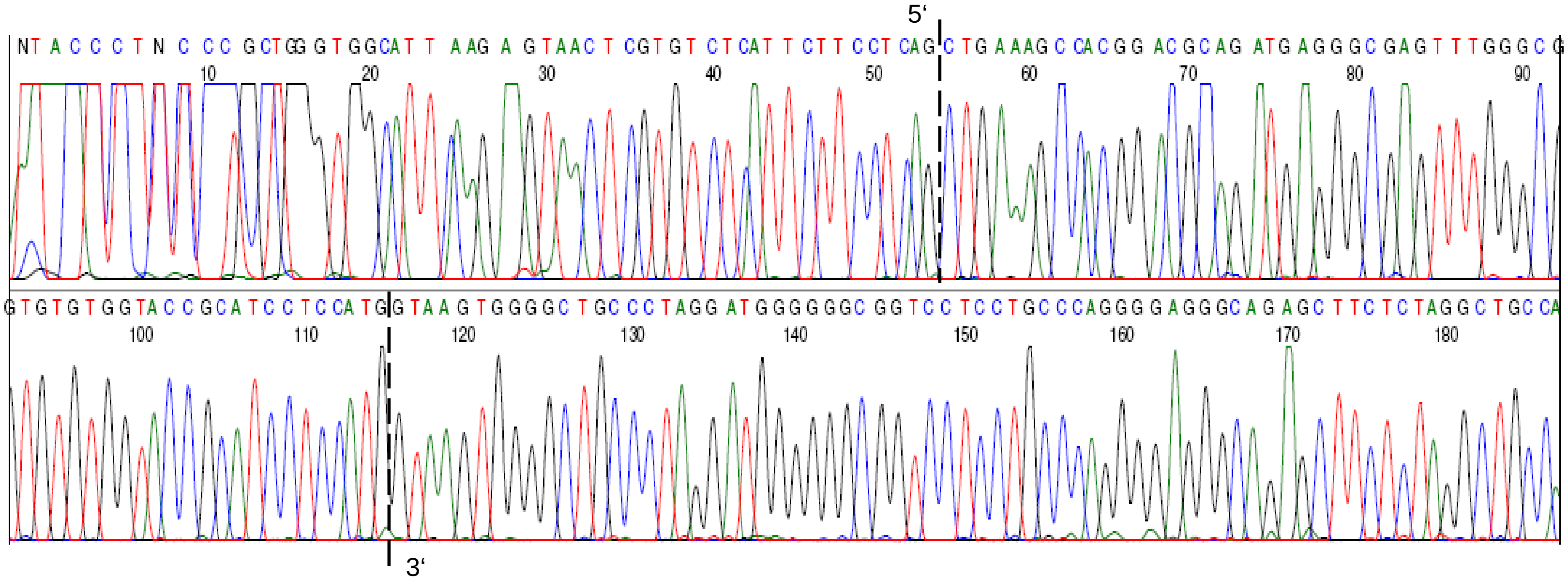

Exon 29

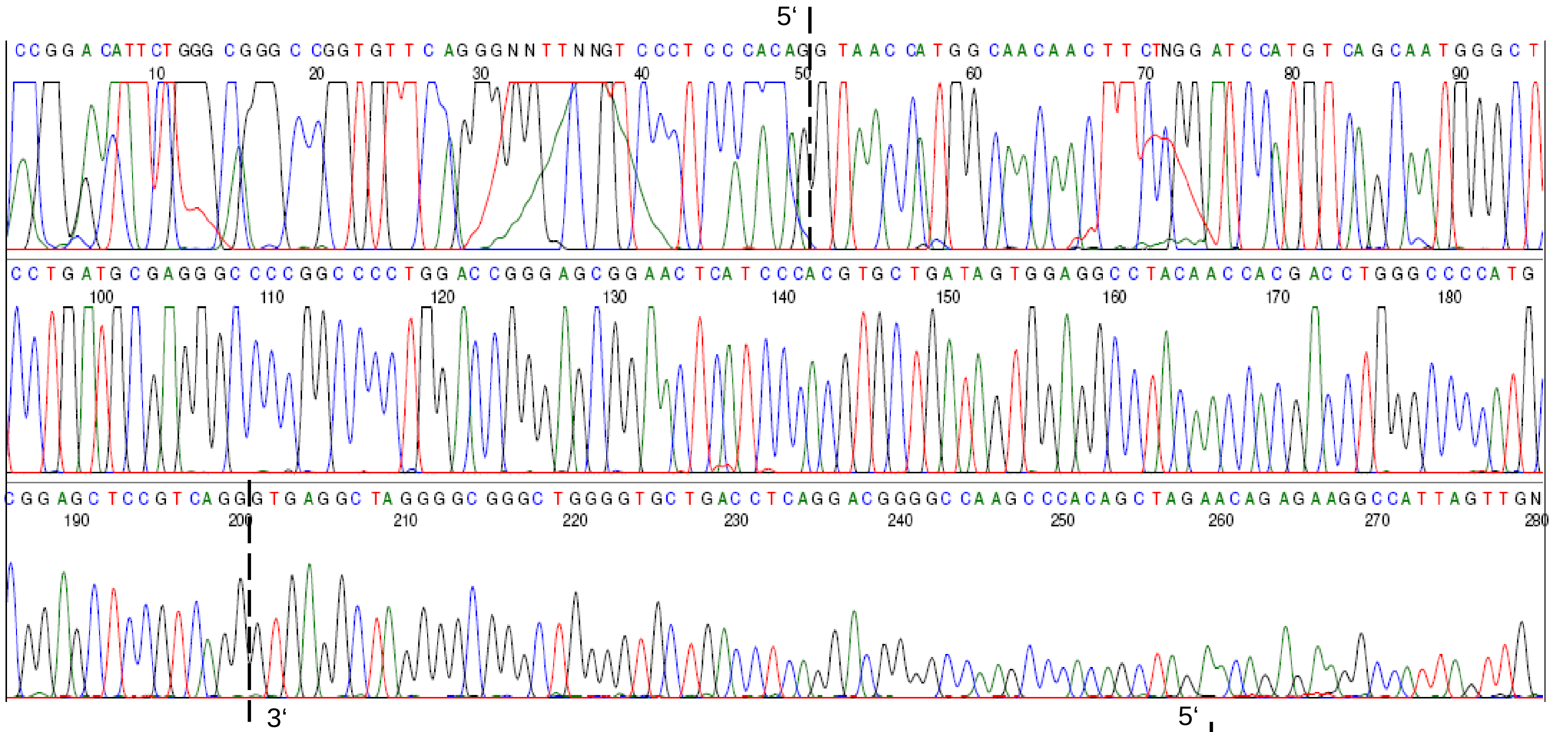

Exon 30

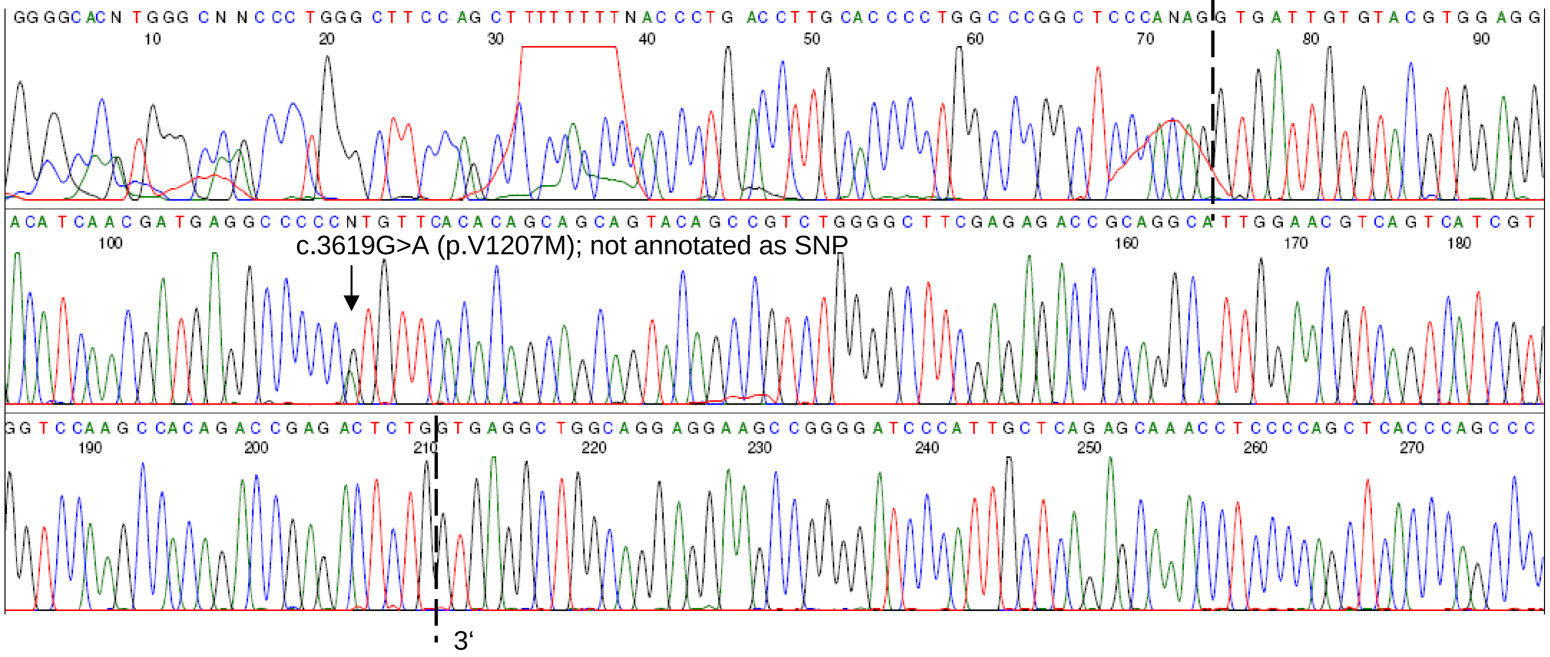

Exon 31

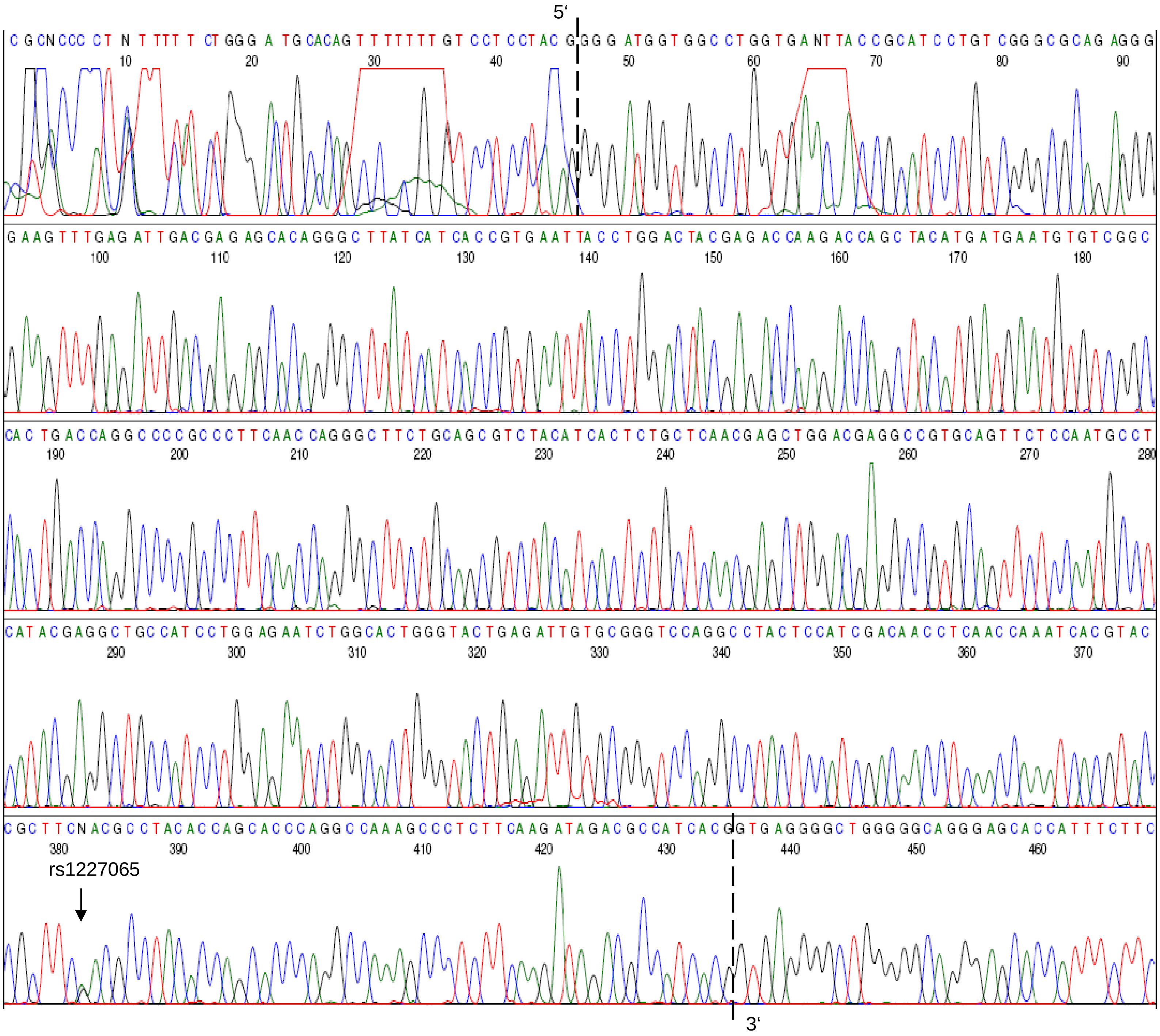

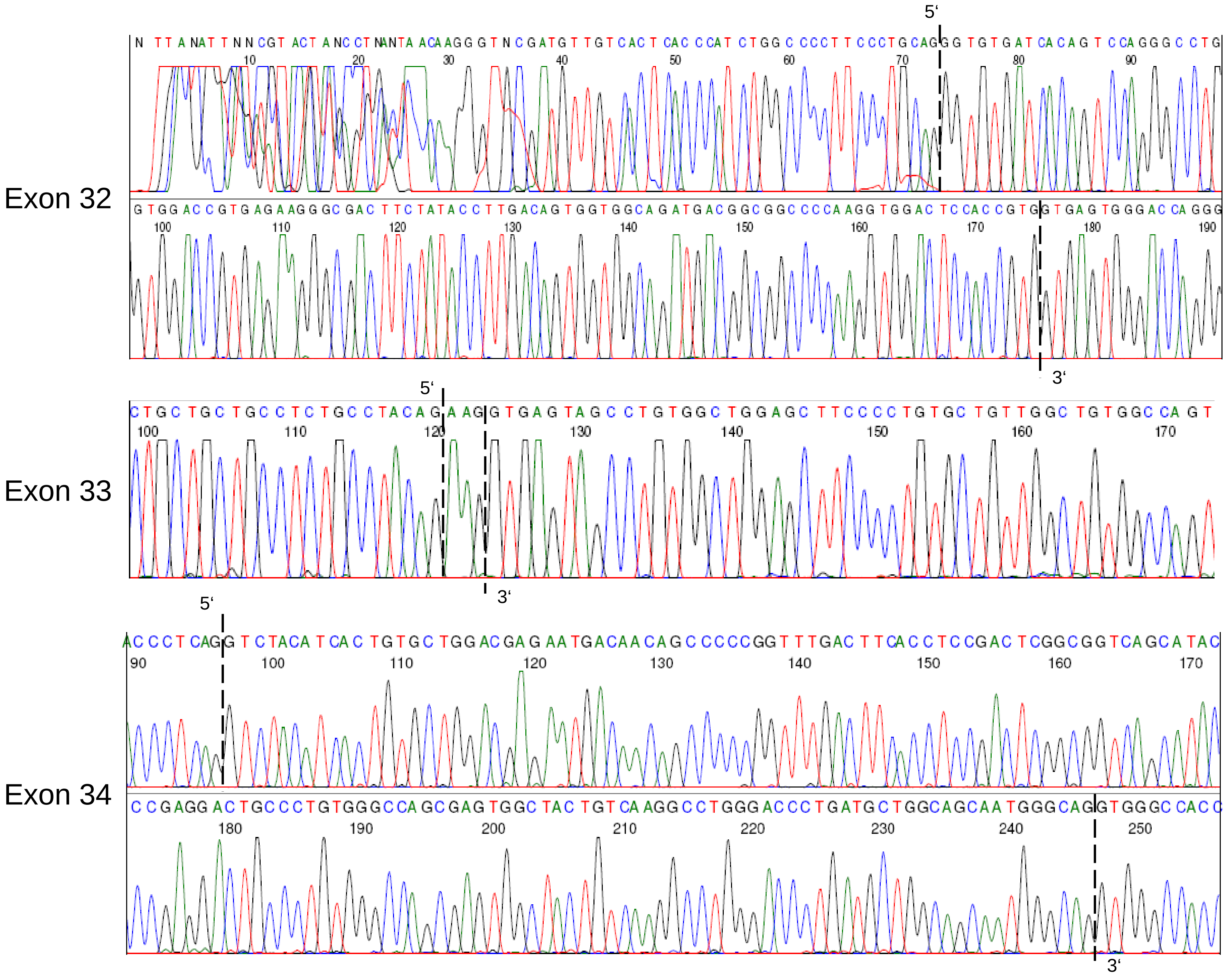
Exon 32
Exon 33
Exon 34
5‘
3‘

Exon 35

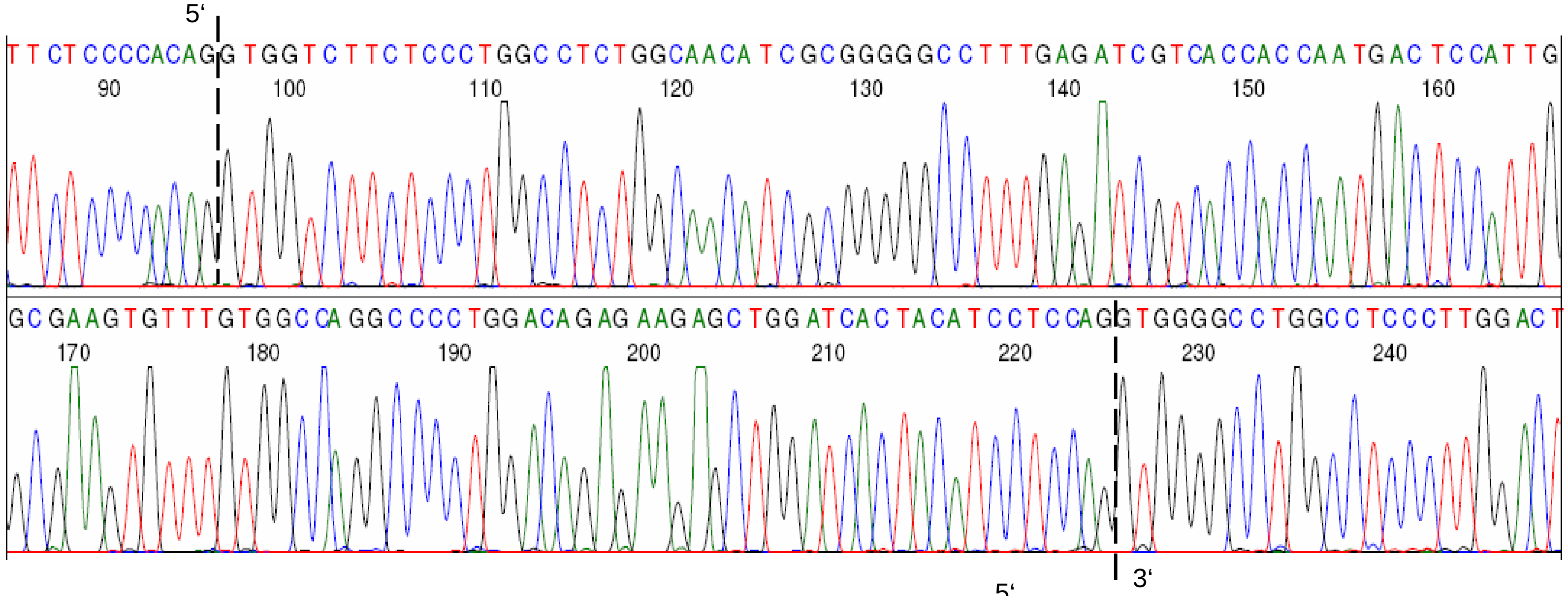

Exon 36

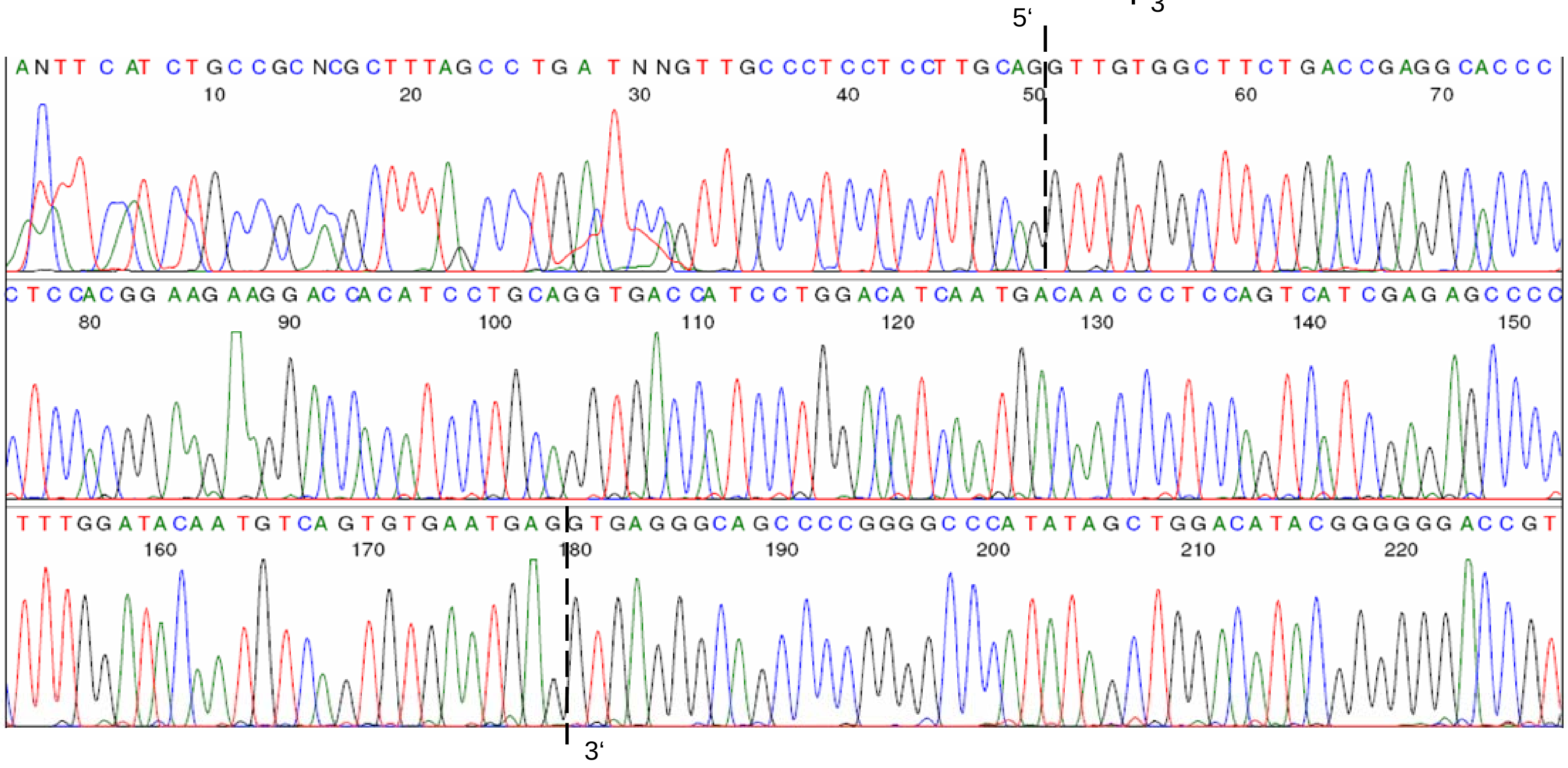

Exon 37

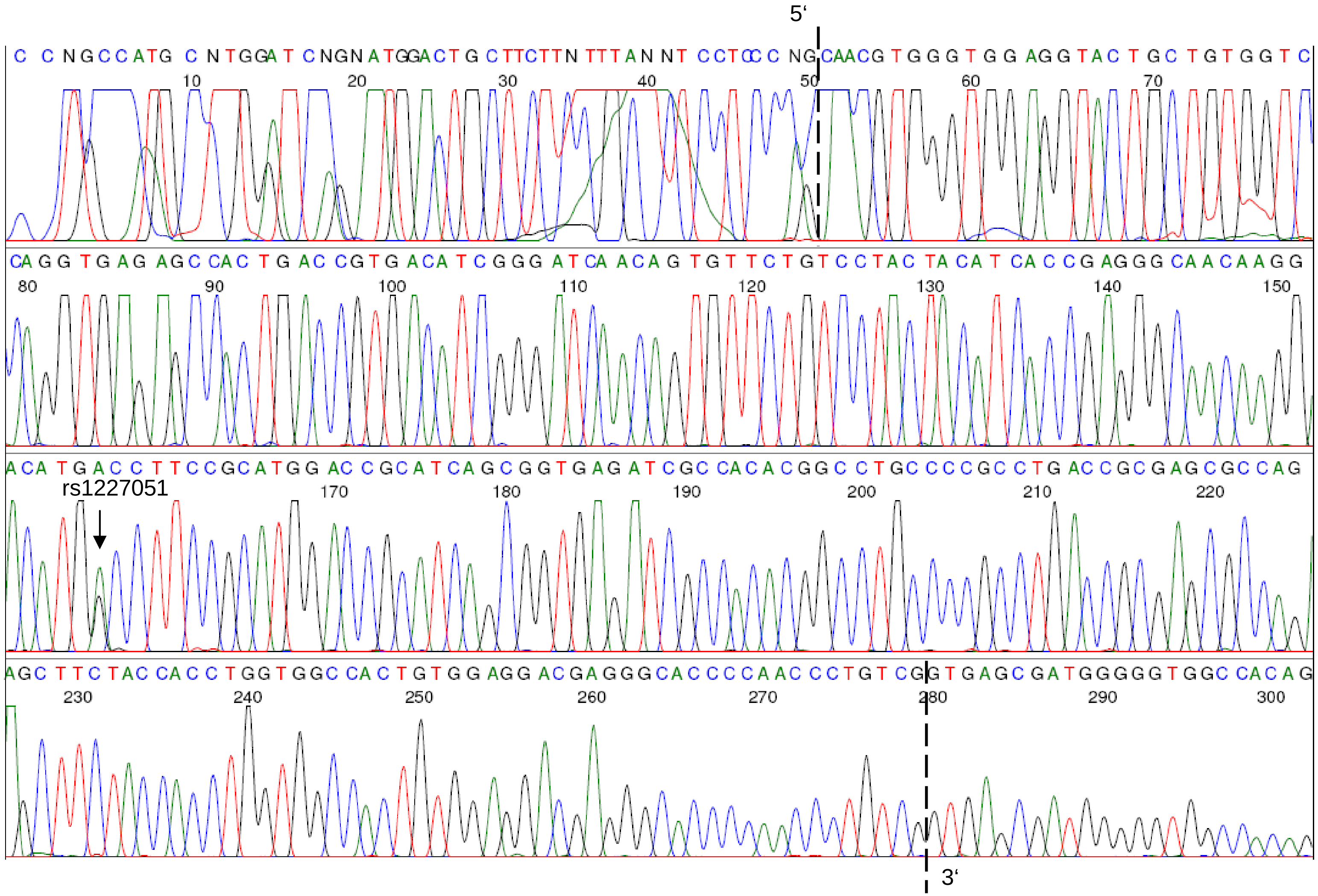

Exon 38

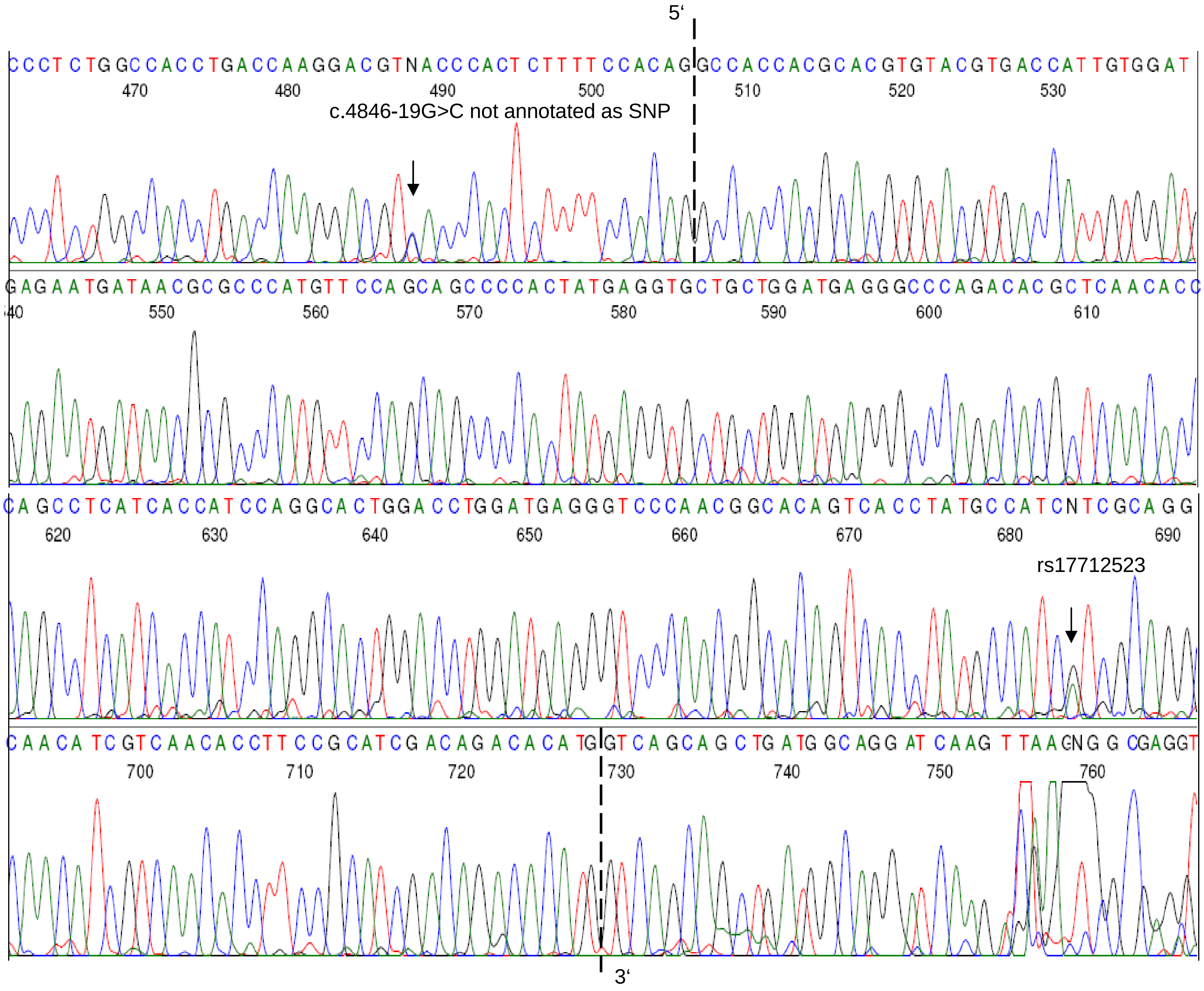

Exon 39

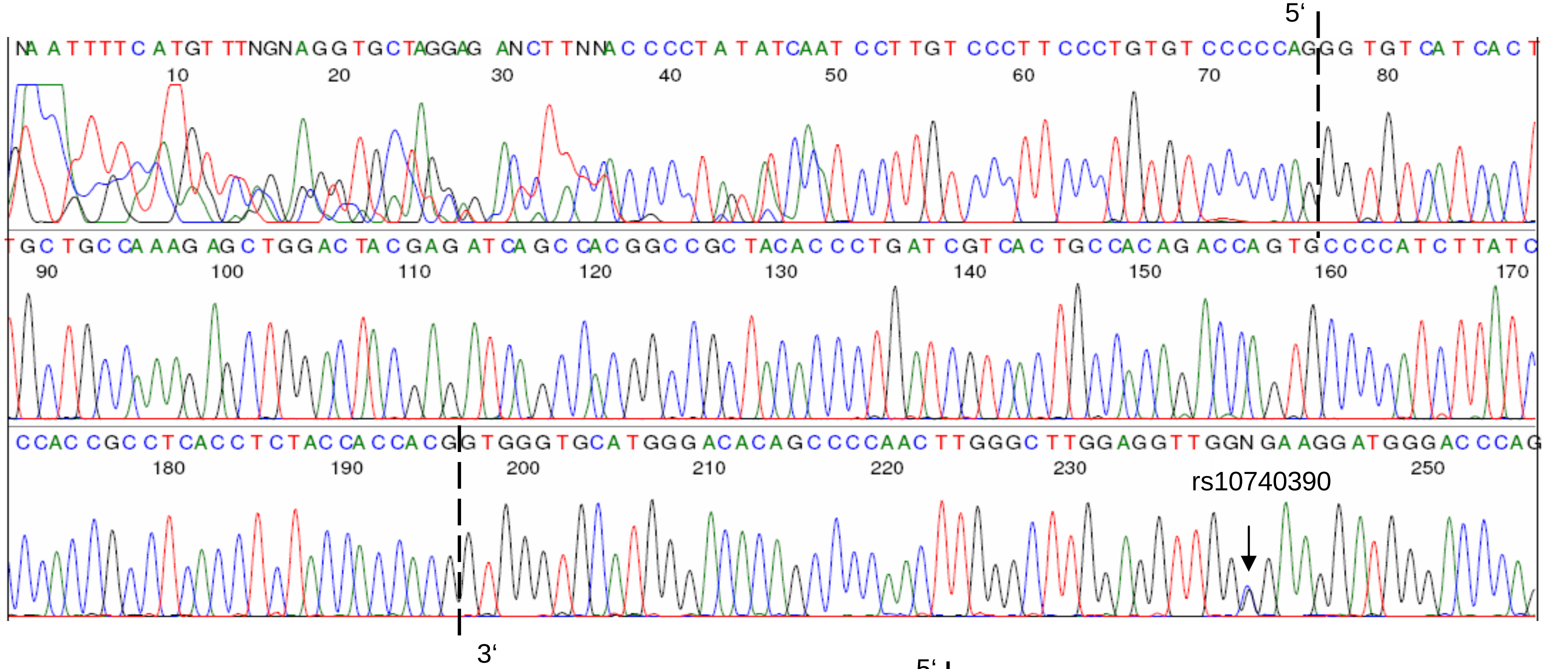

Exon 40

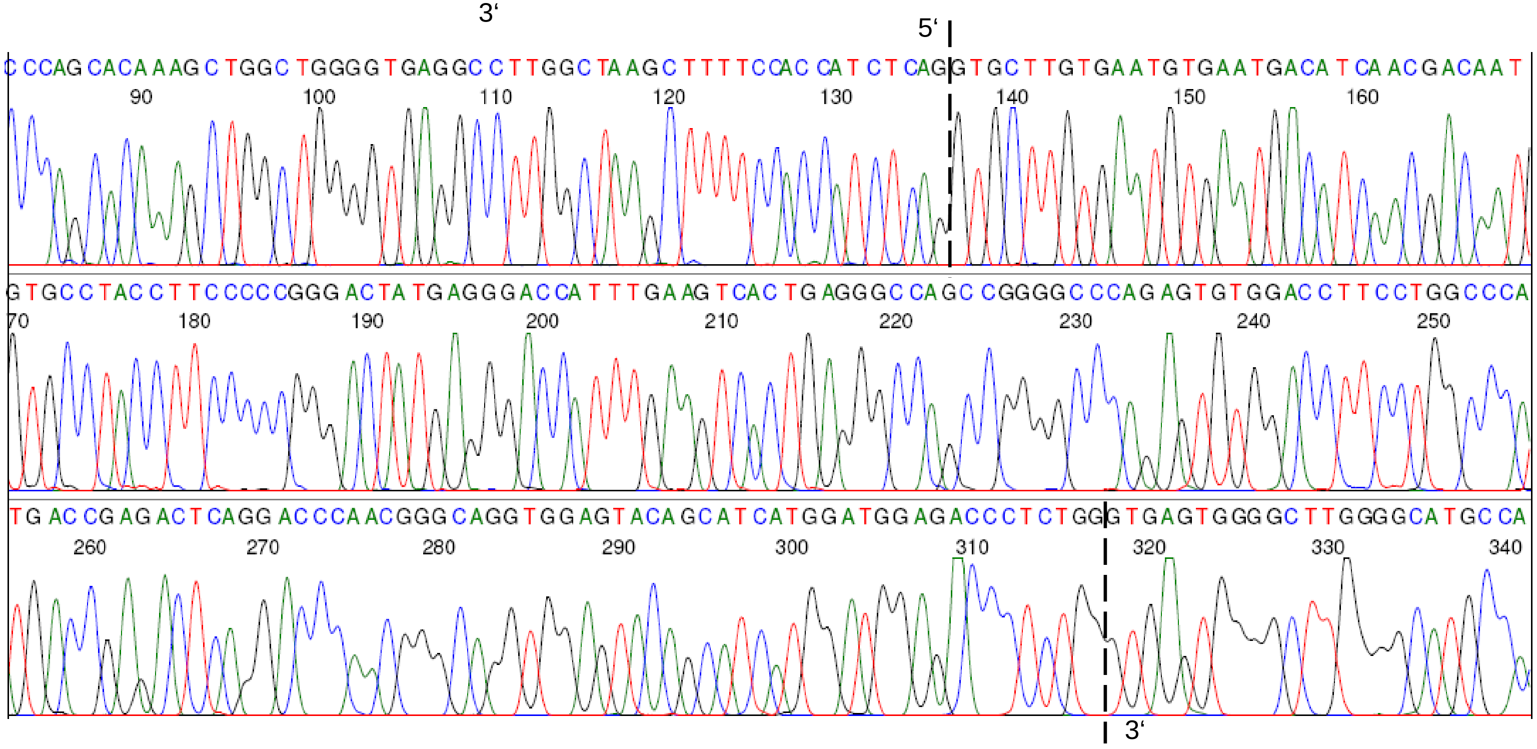

Exon 41

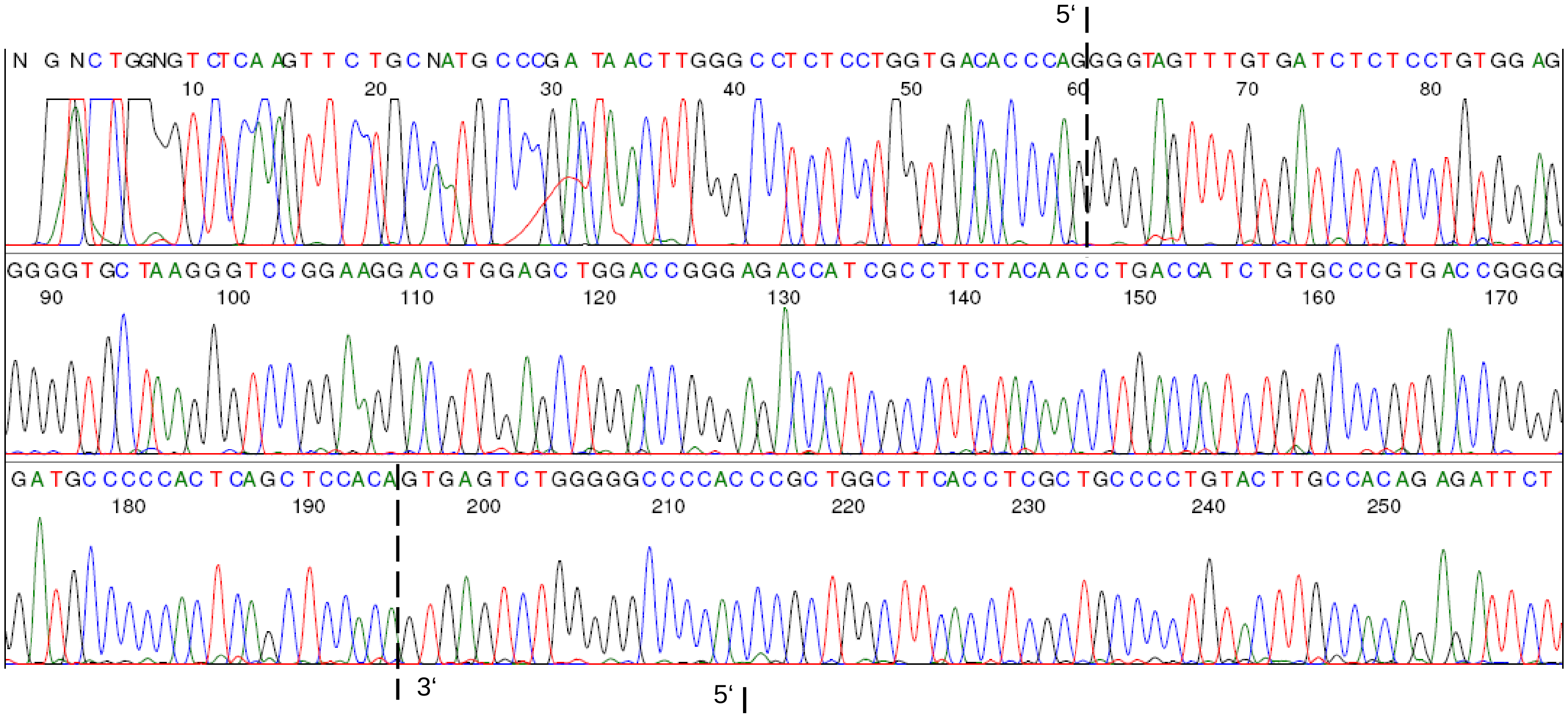

Exon 42

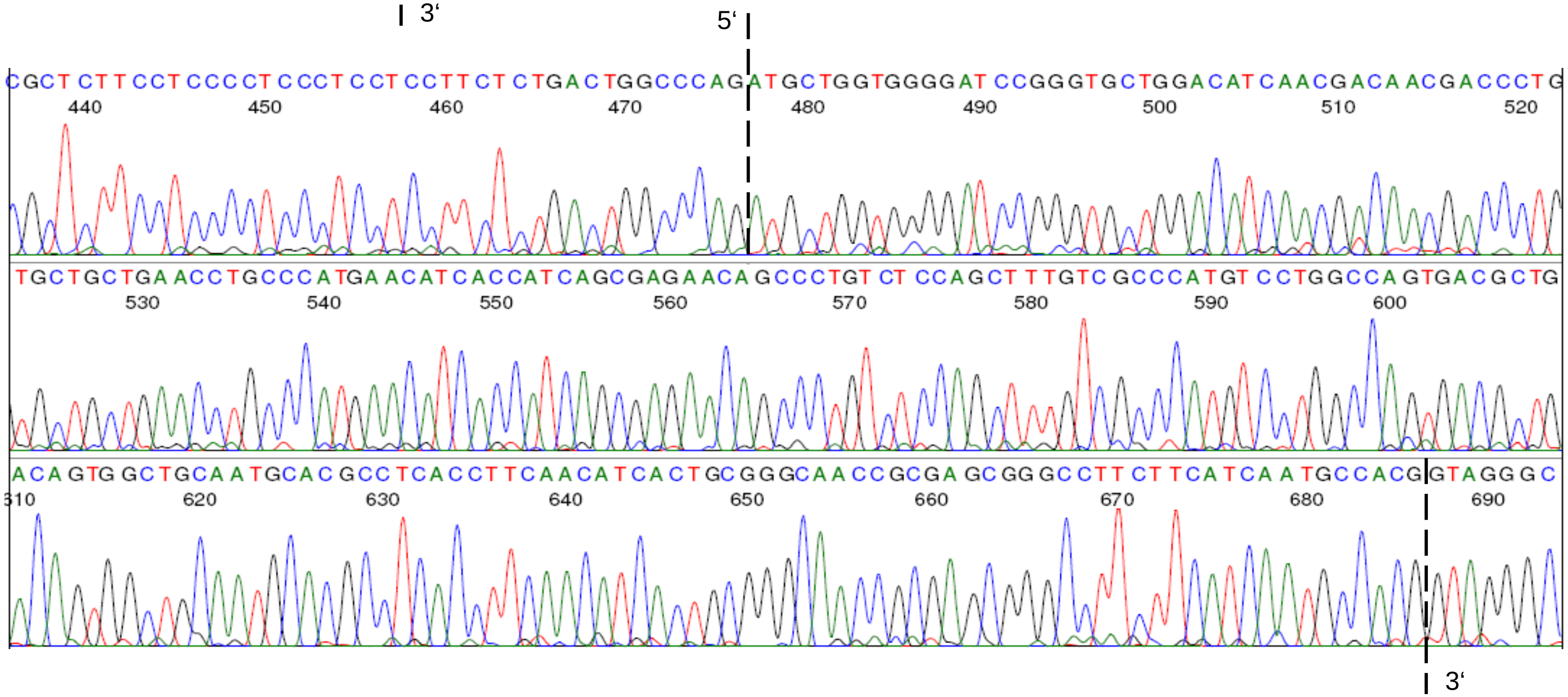

Exon 43

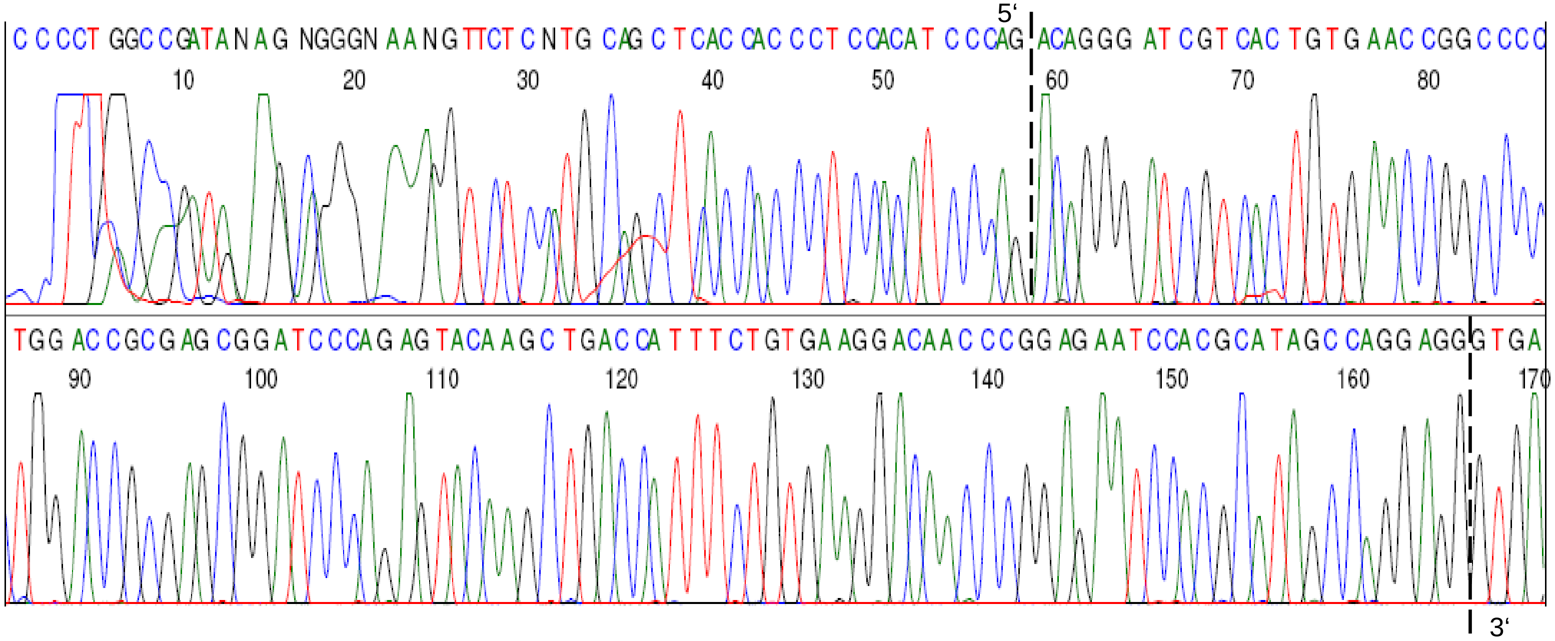

Exon 44

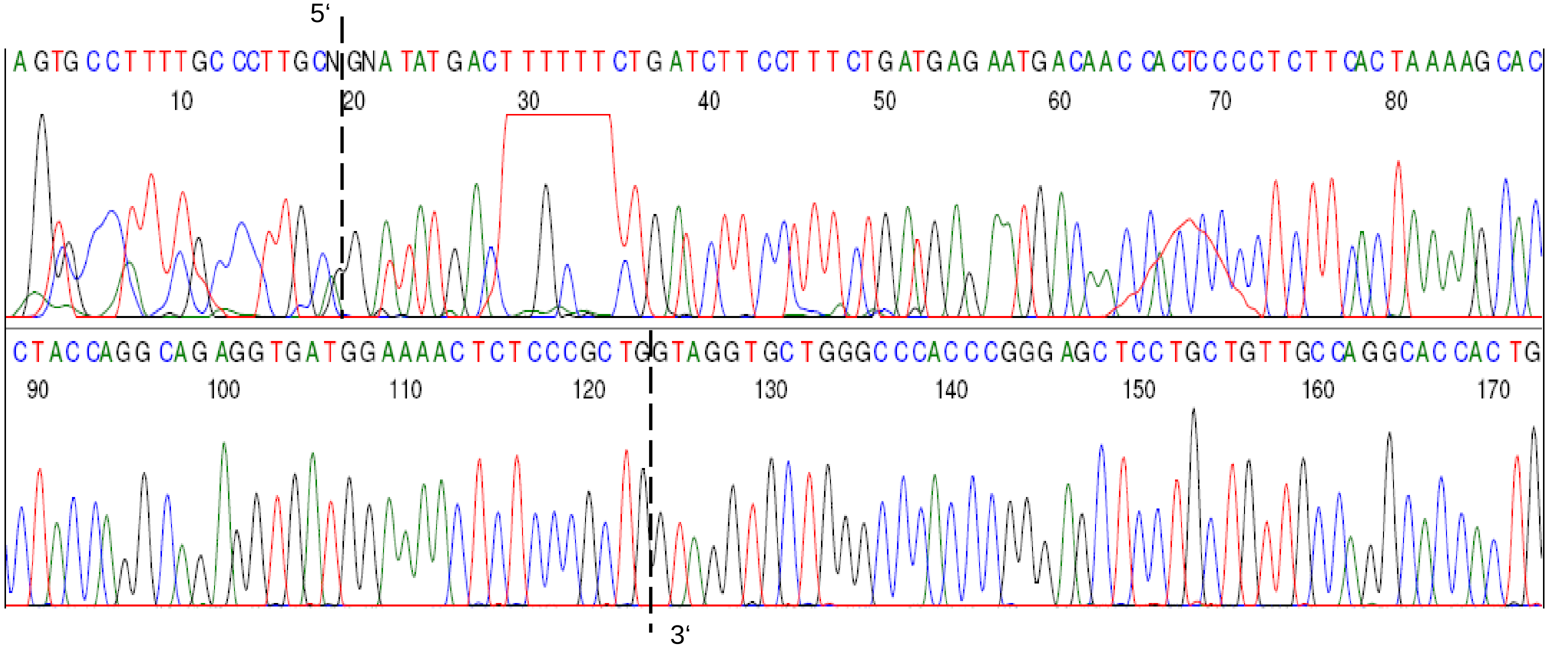

Exon 45

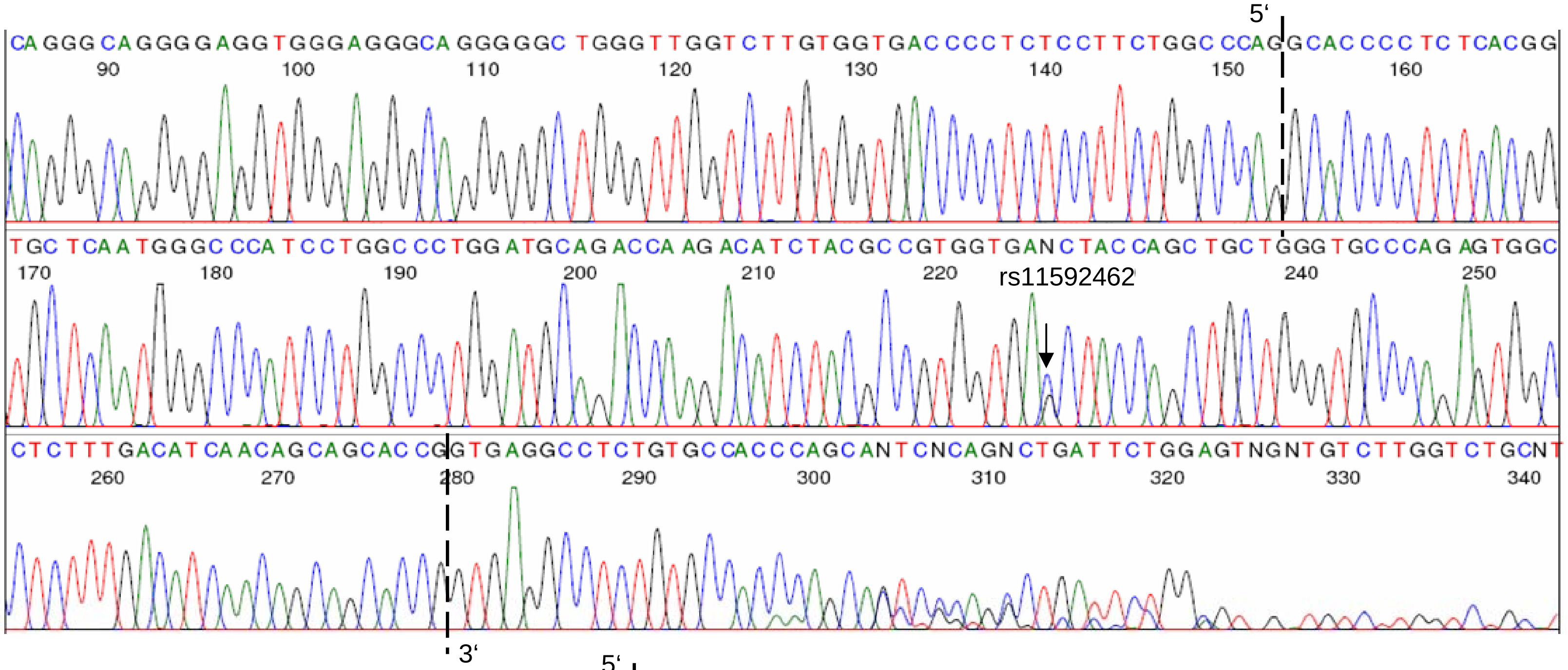

Exon 46

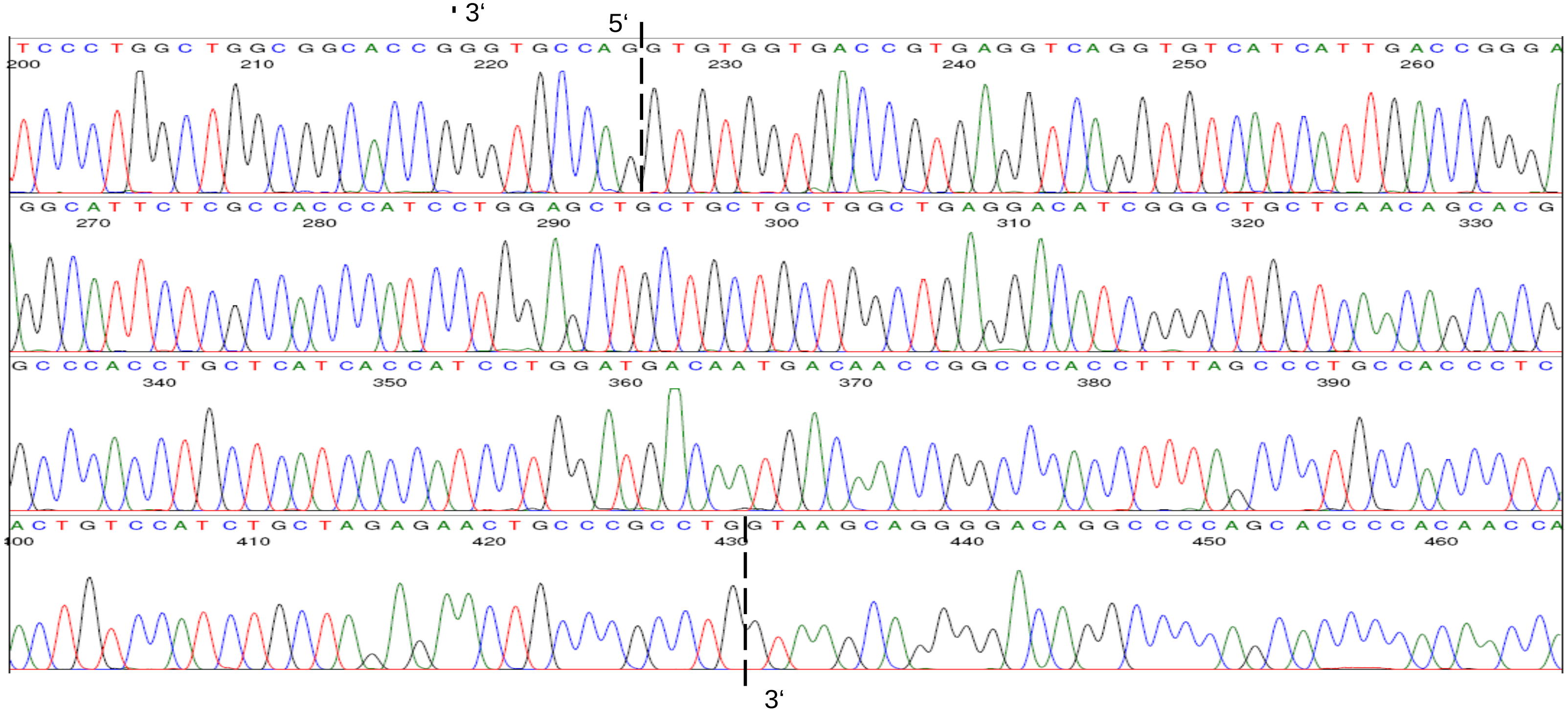

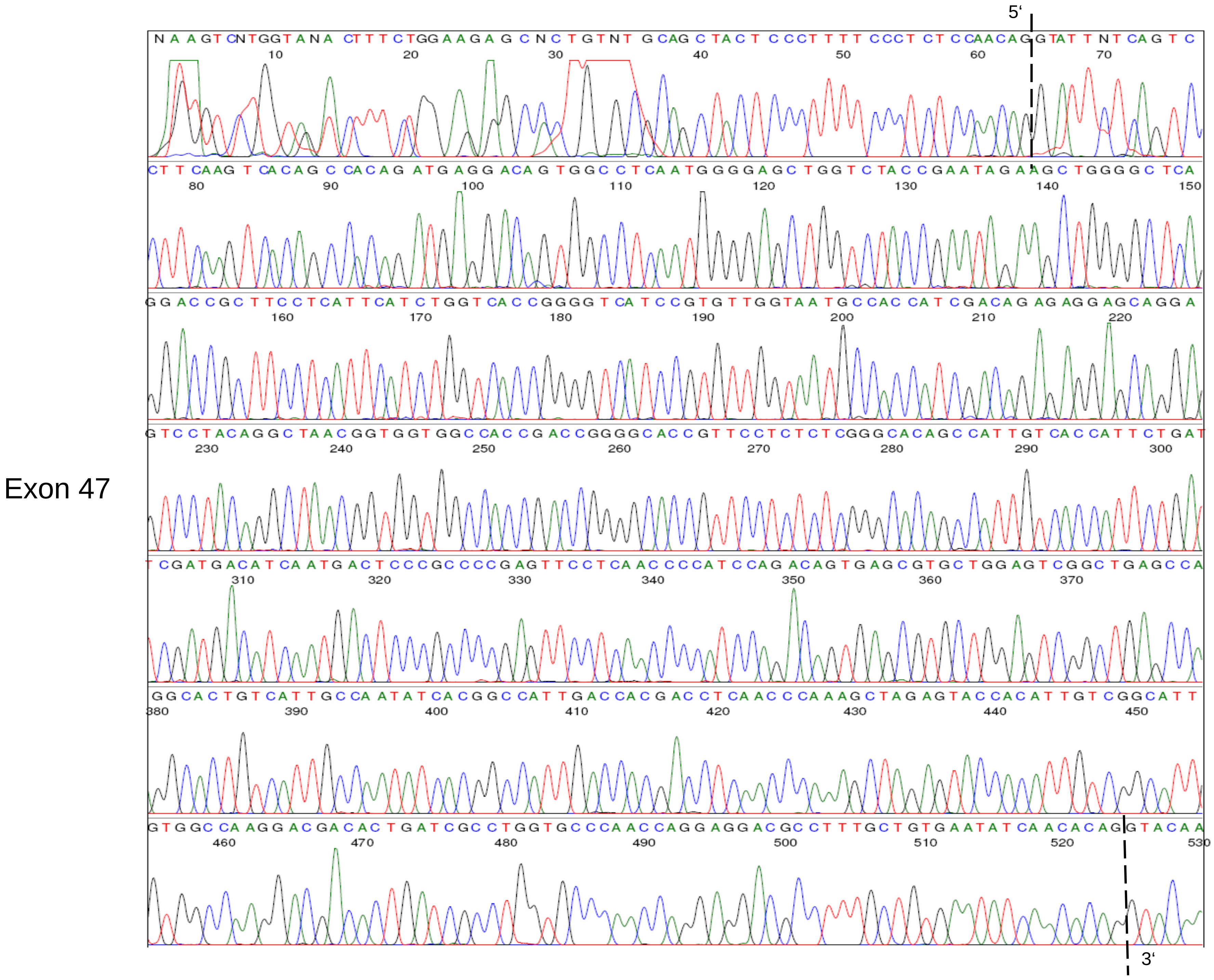
5‘
3‘
Exon 47

Exon 48

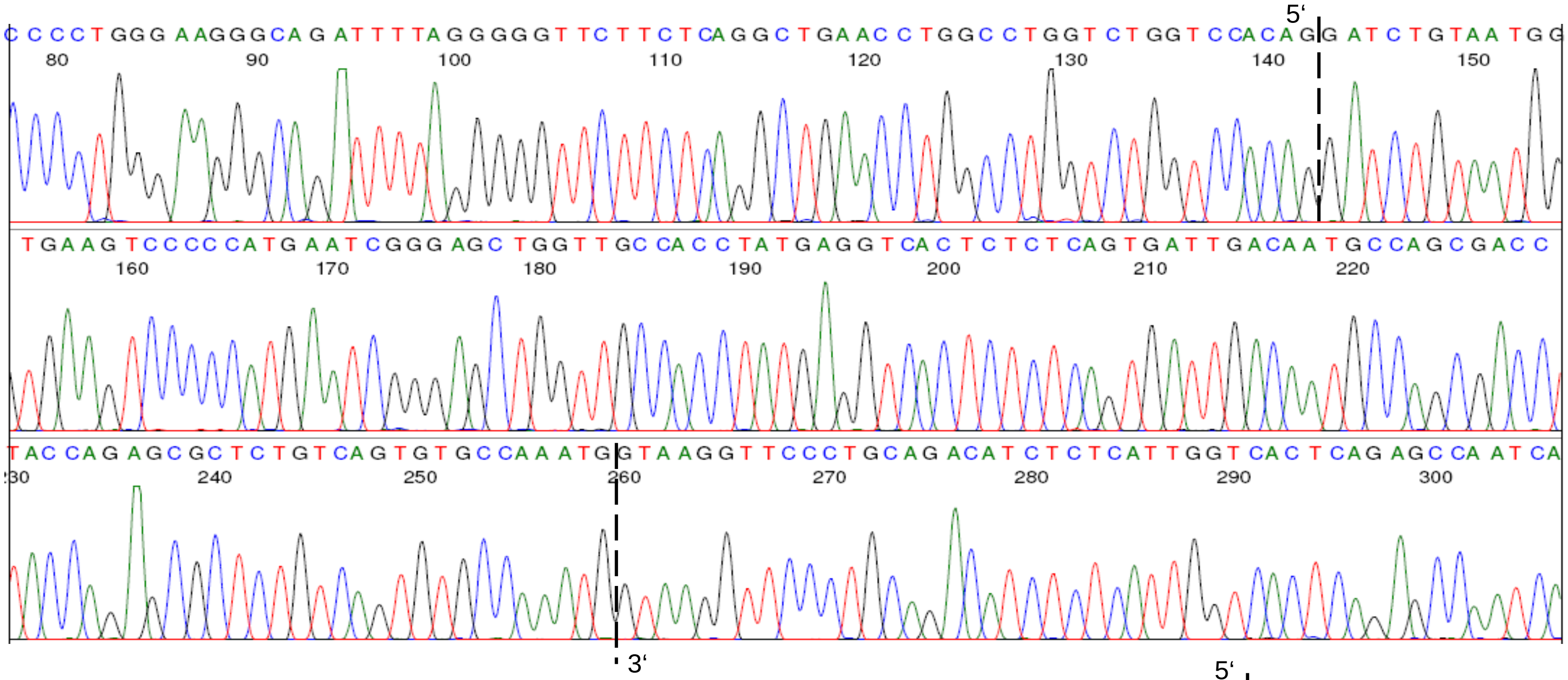

Exon 49

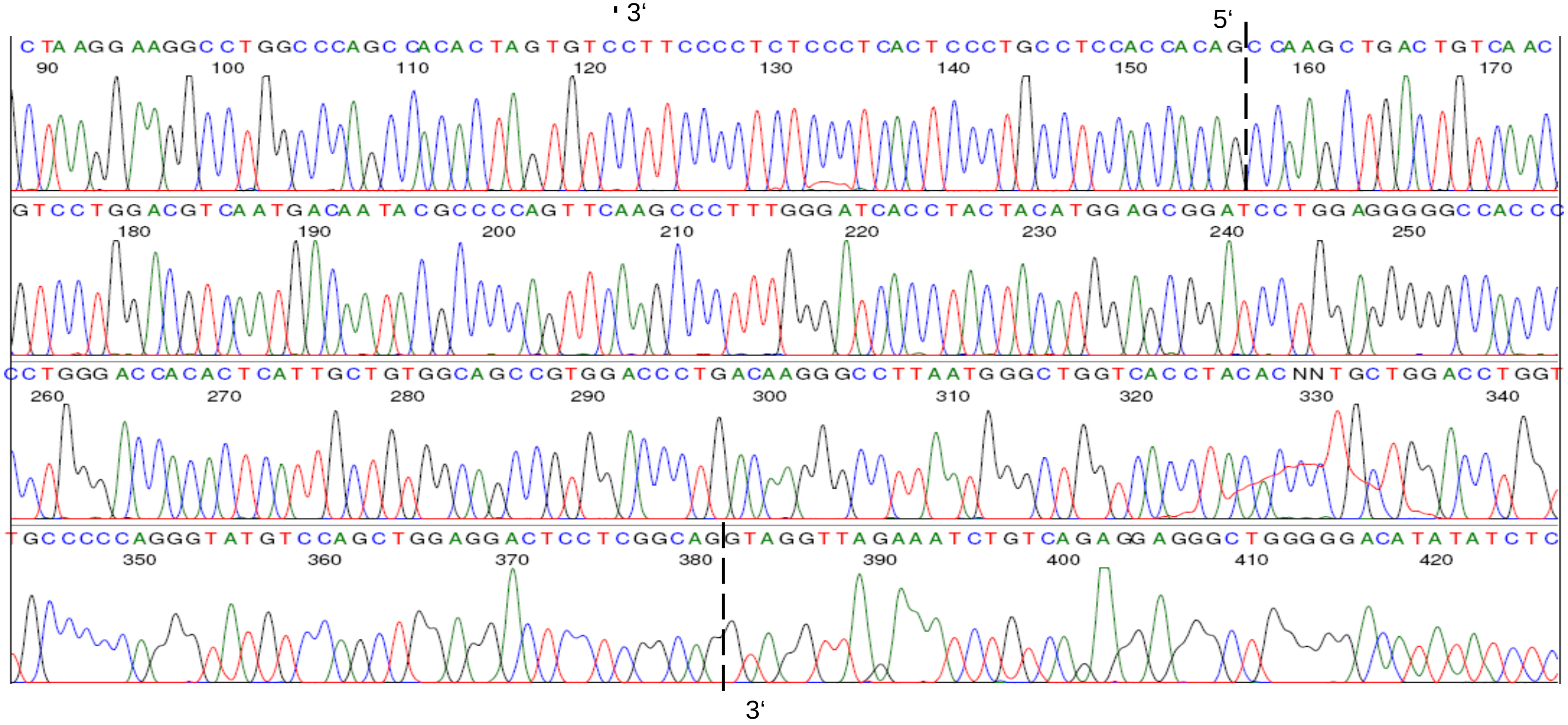

Exon 50

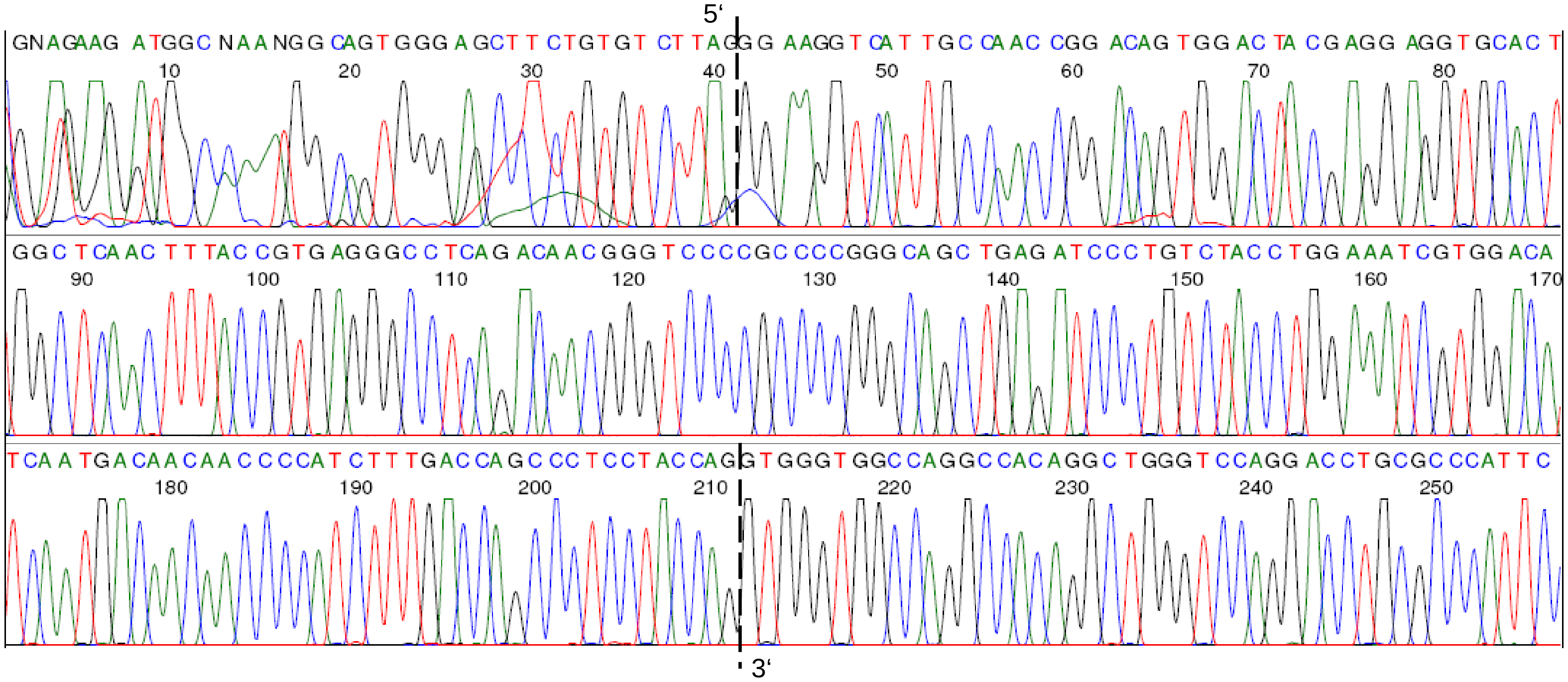

Exon 51

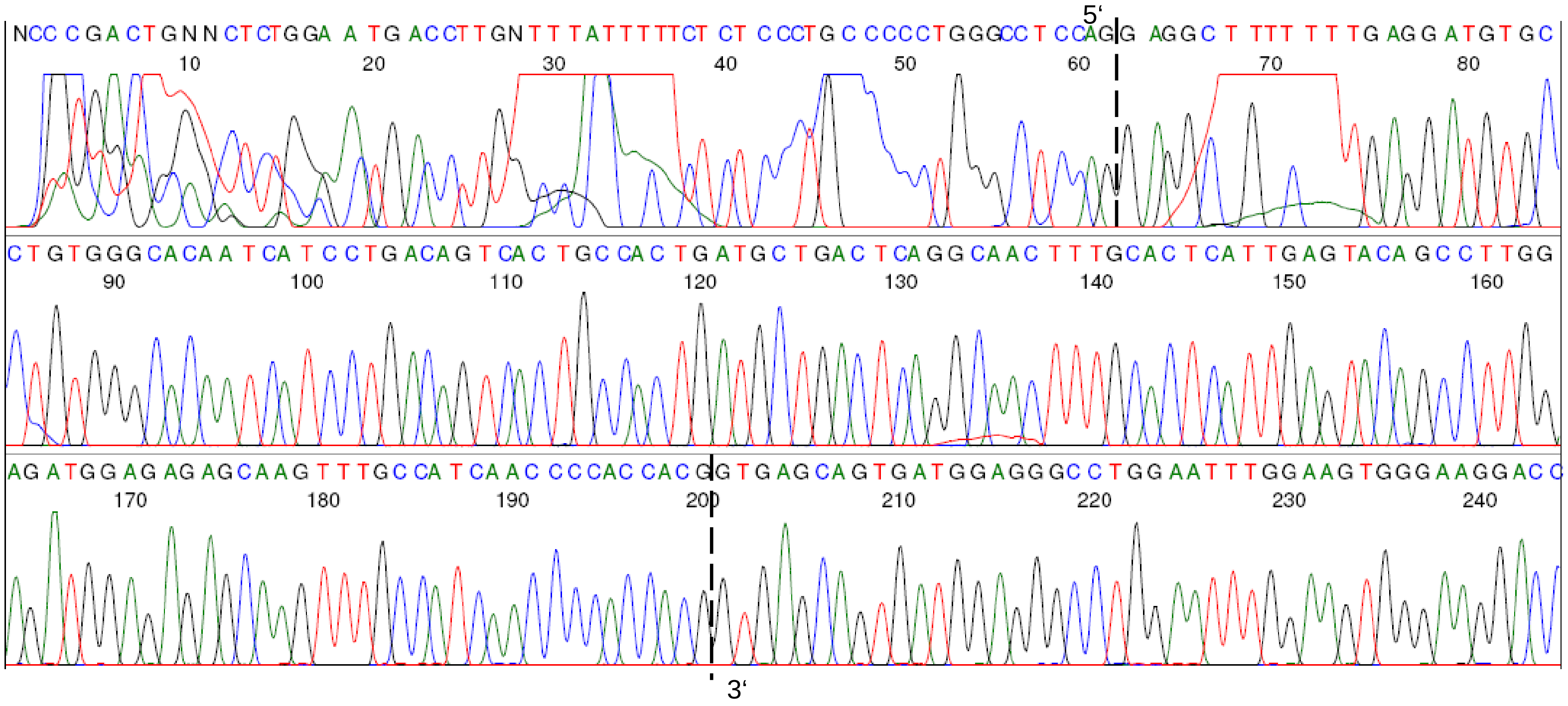

Exon 52

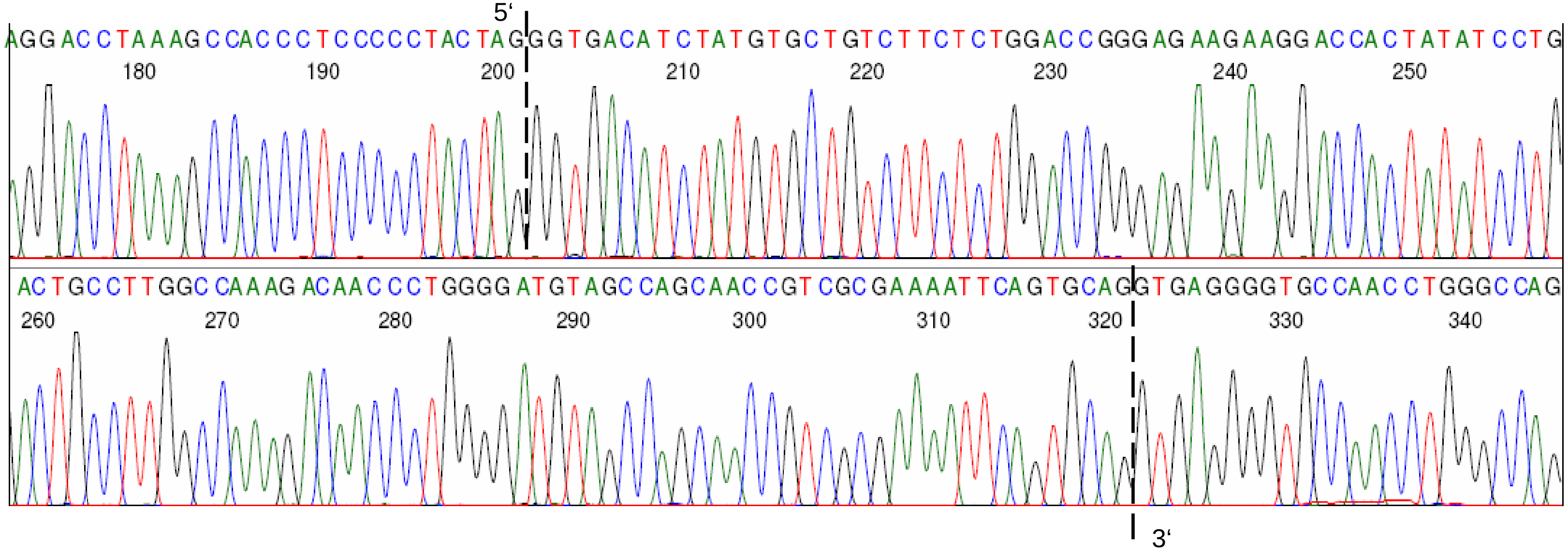

Exon 53

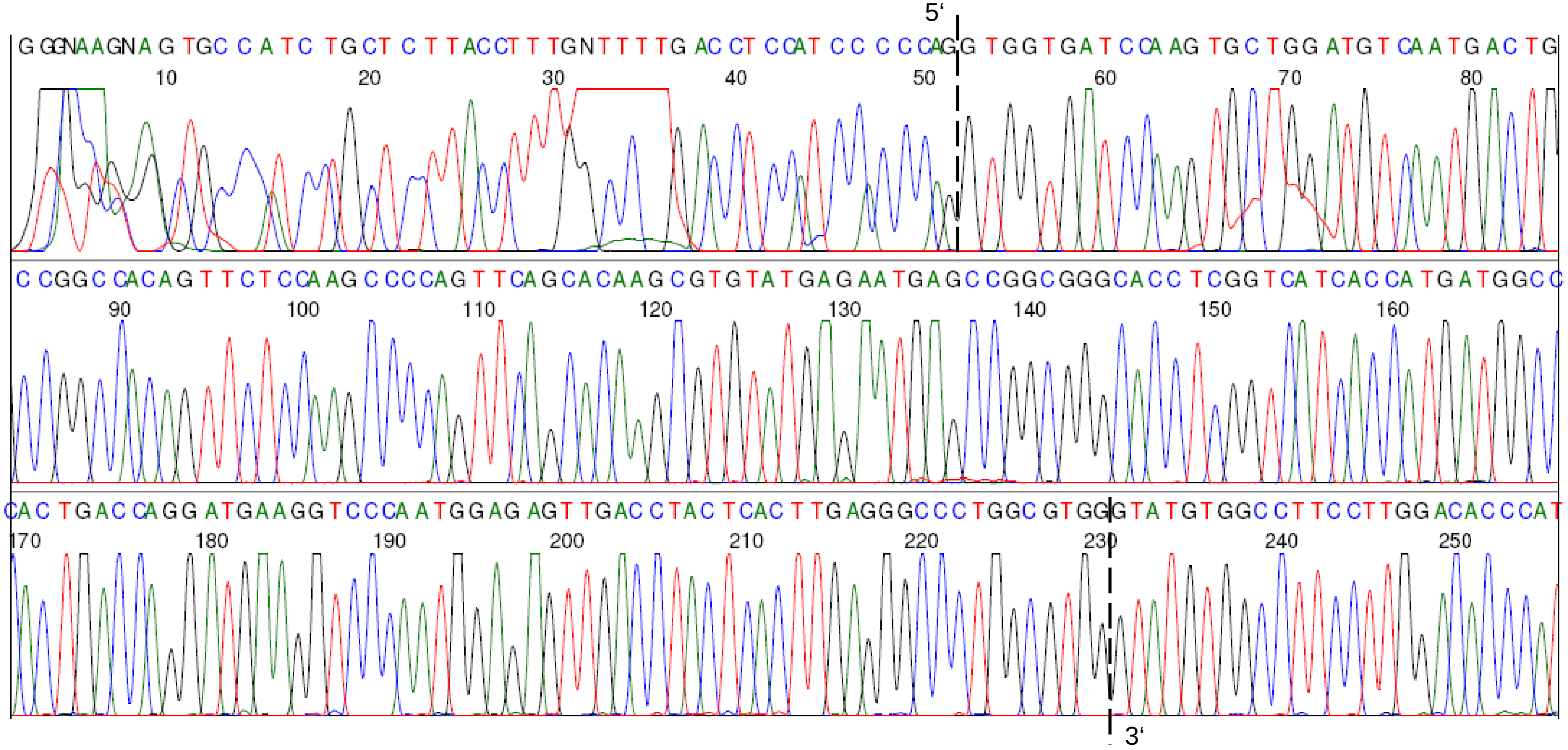

Exon 54

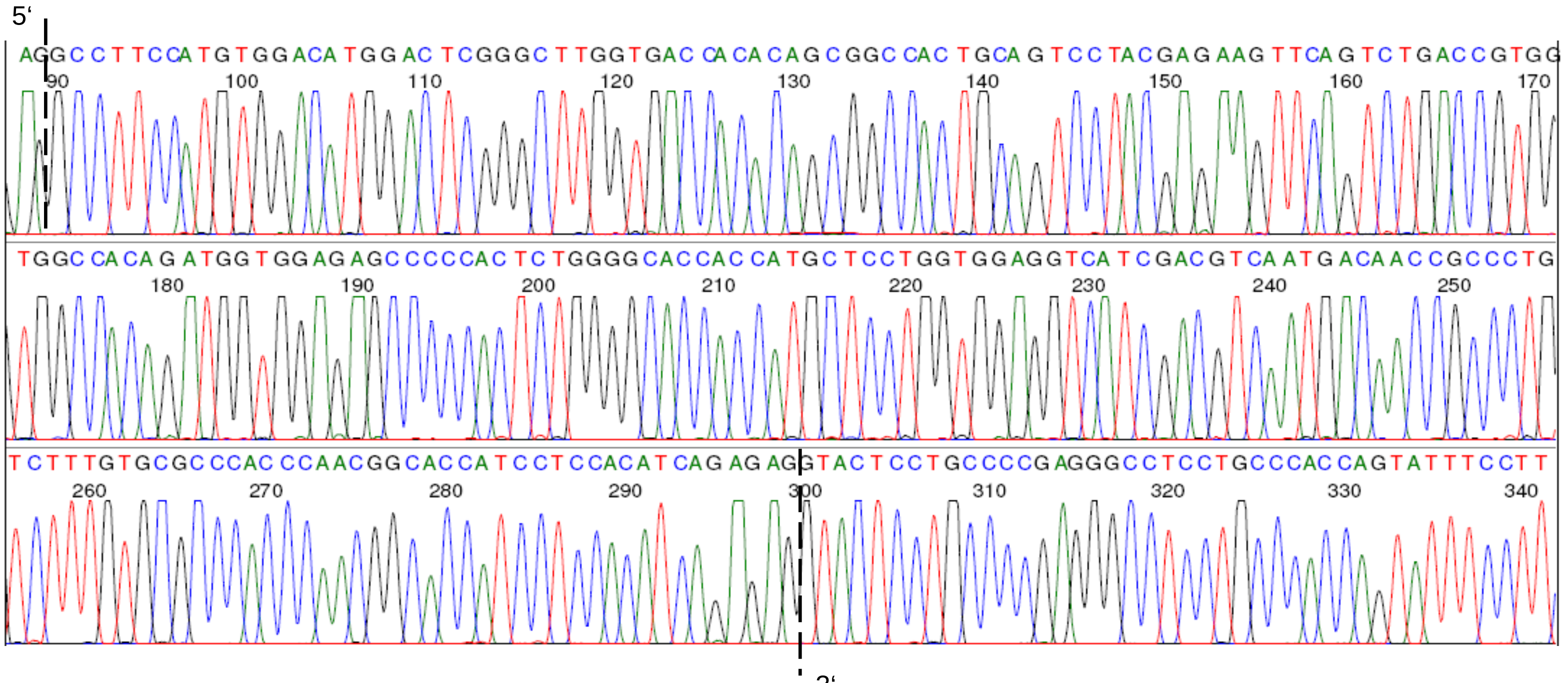

Exon 55

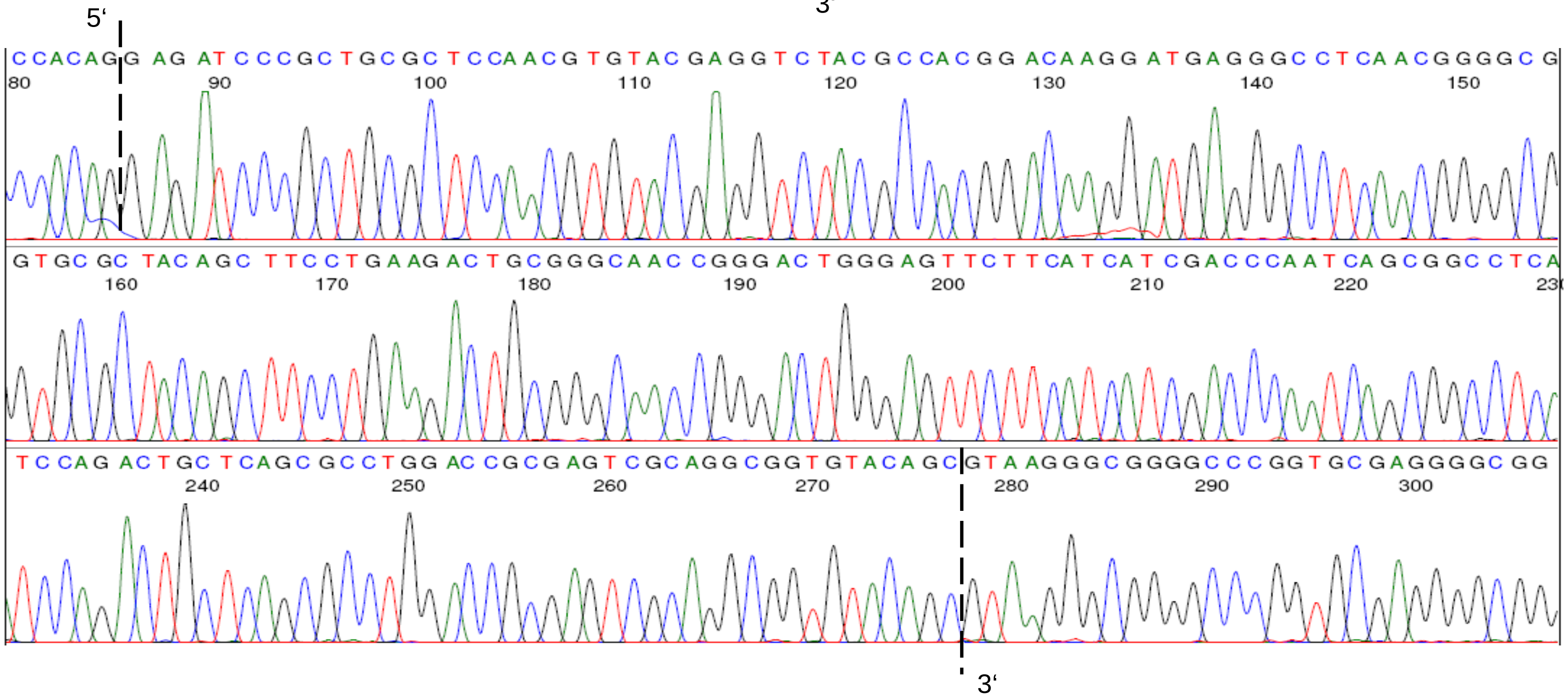

Exon 56

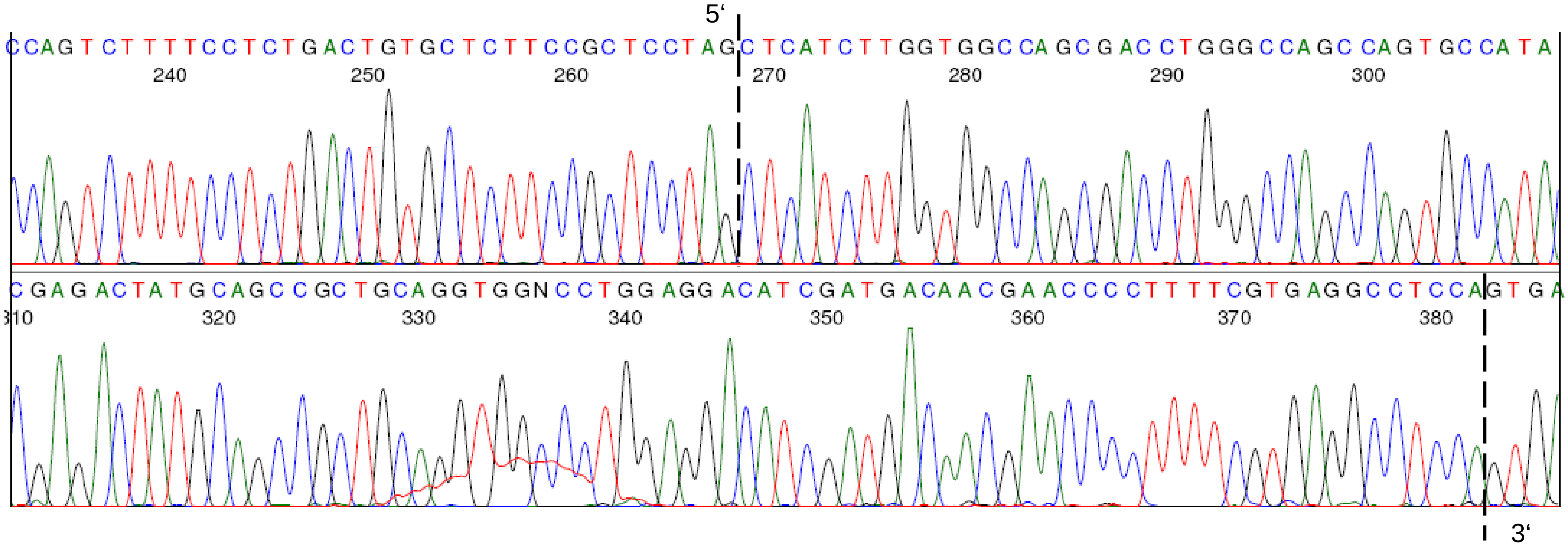

Exon 57

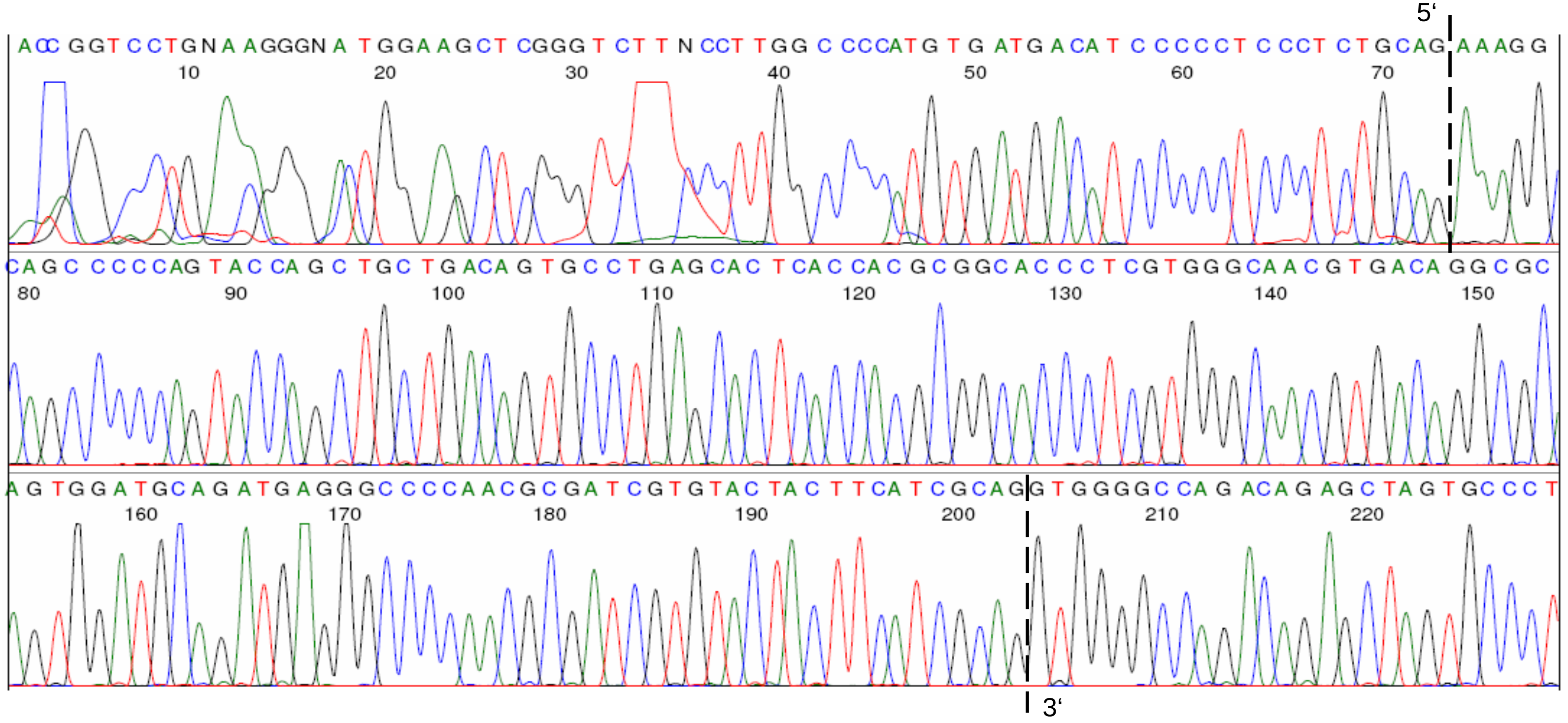

Exon 58

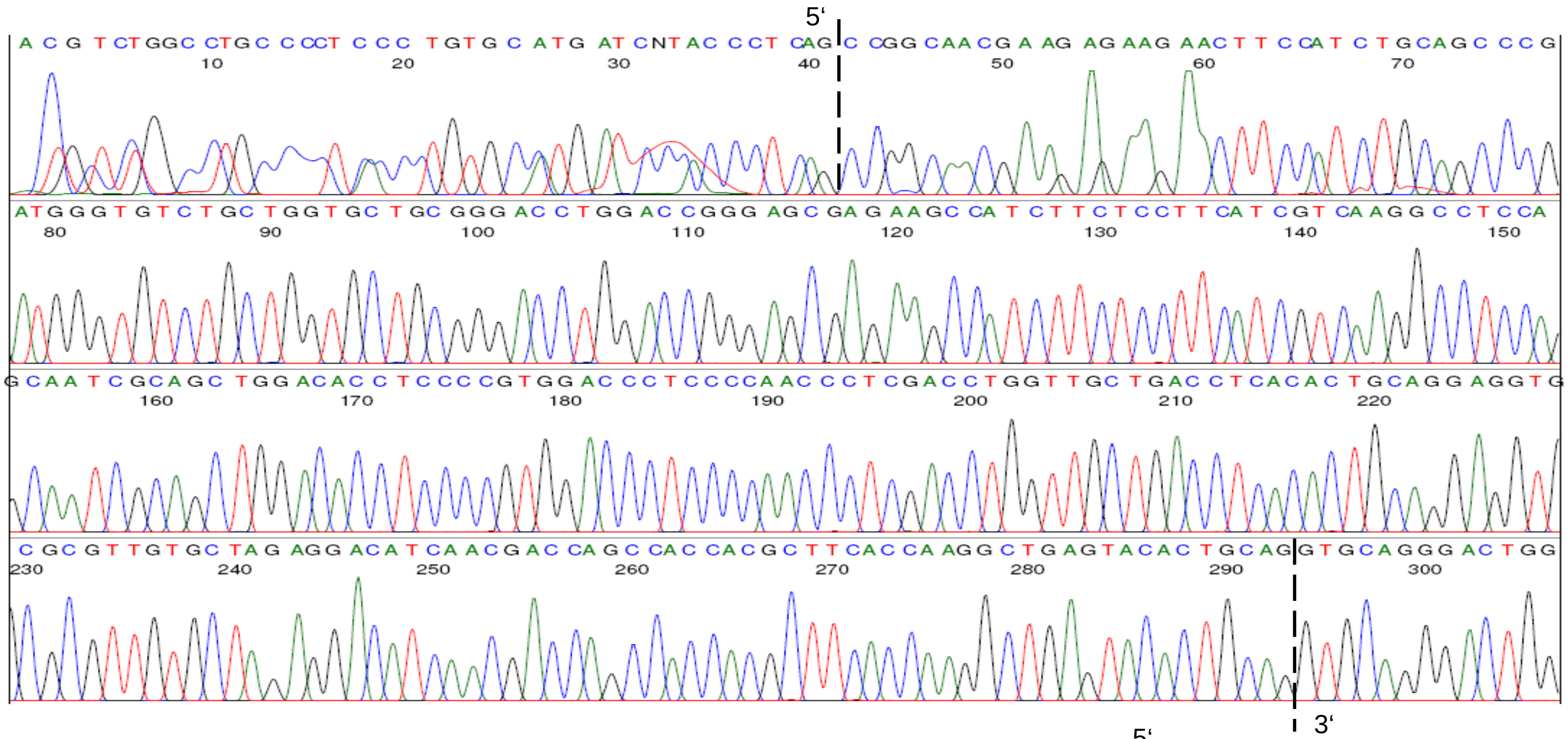

Exon 59

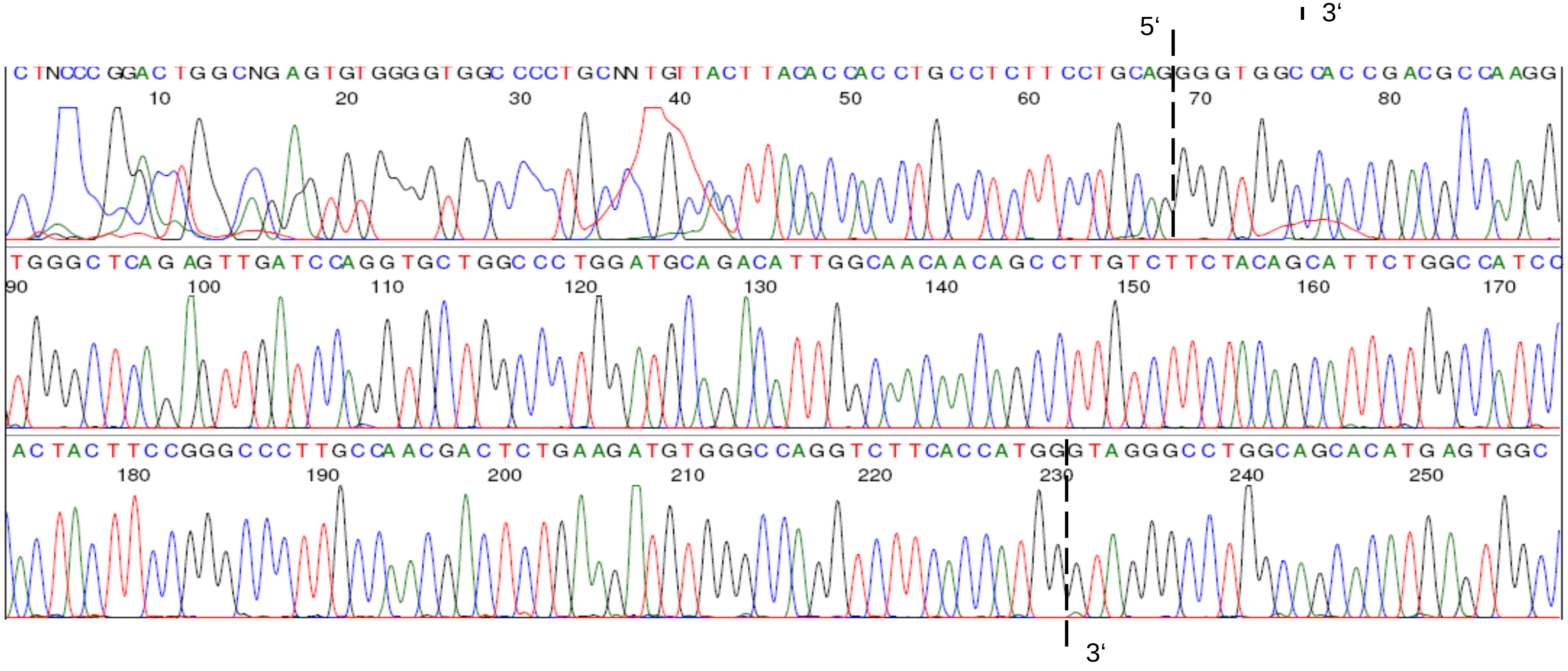

Exon 60

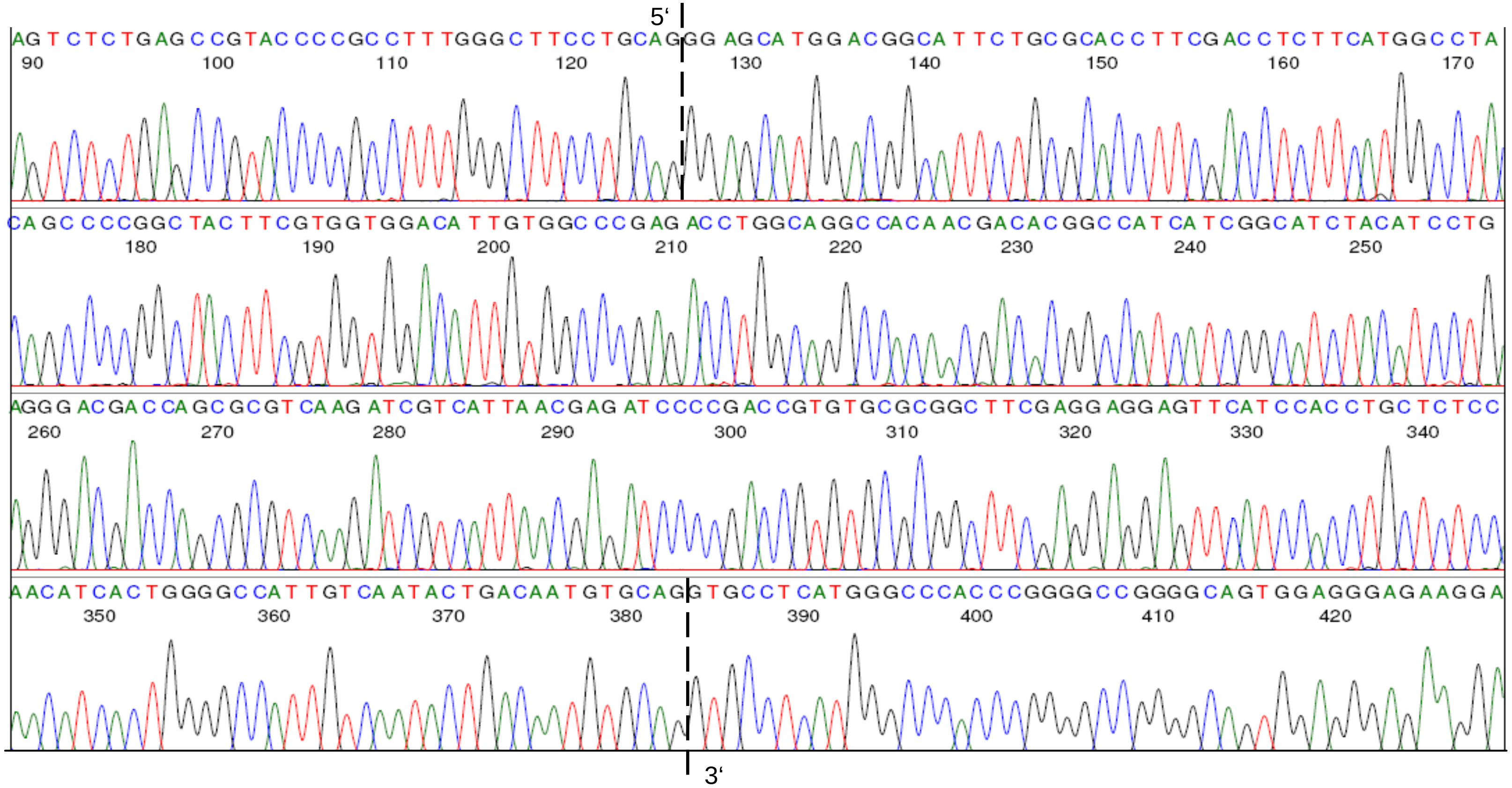

Exon 61

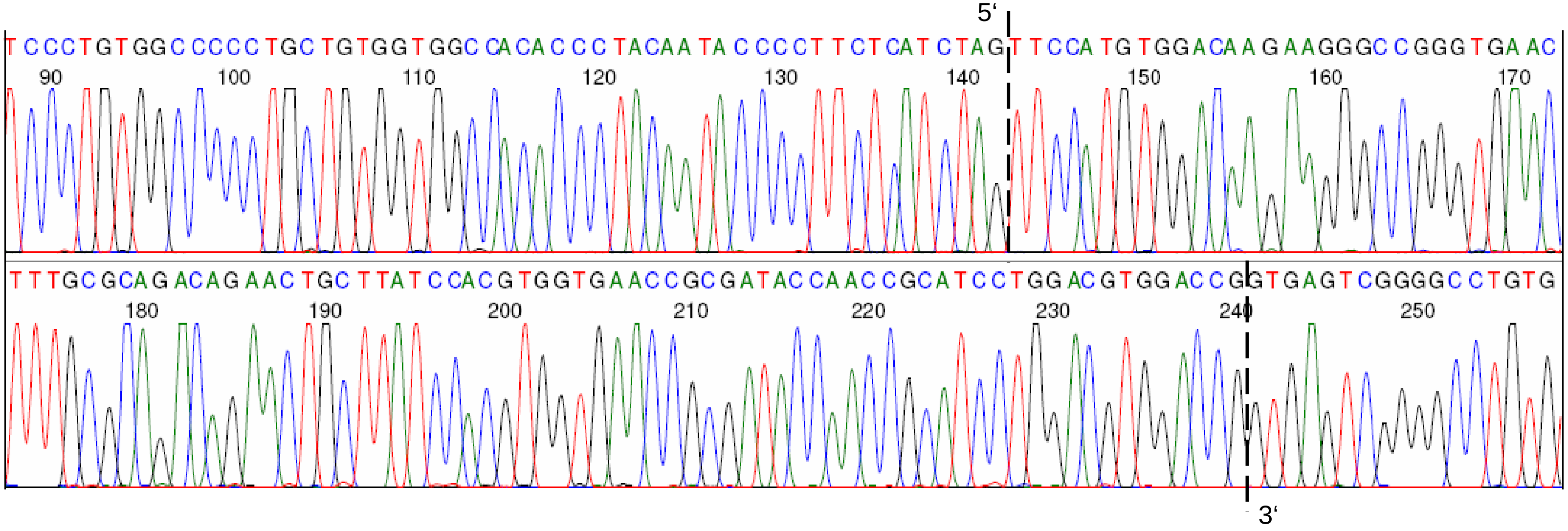

Exon 62

Exon 63

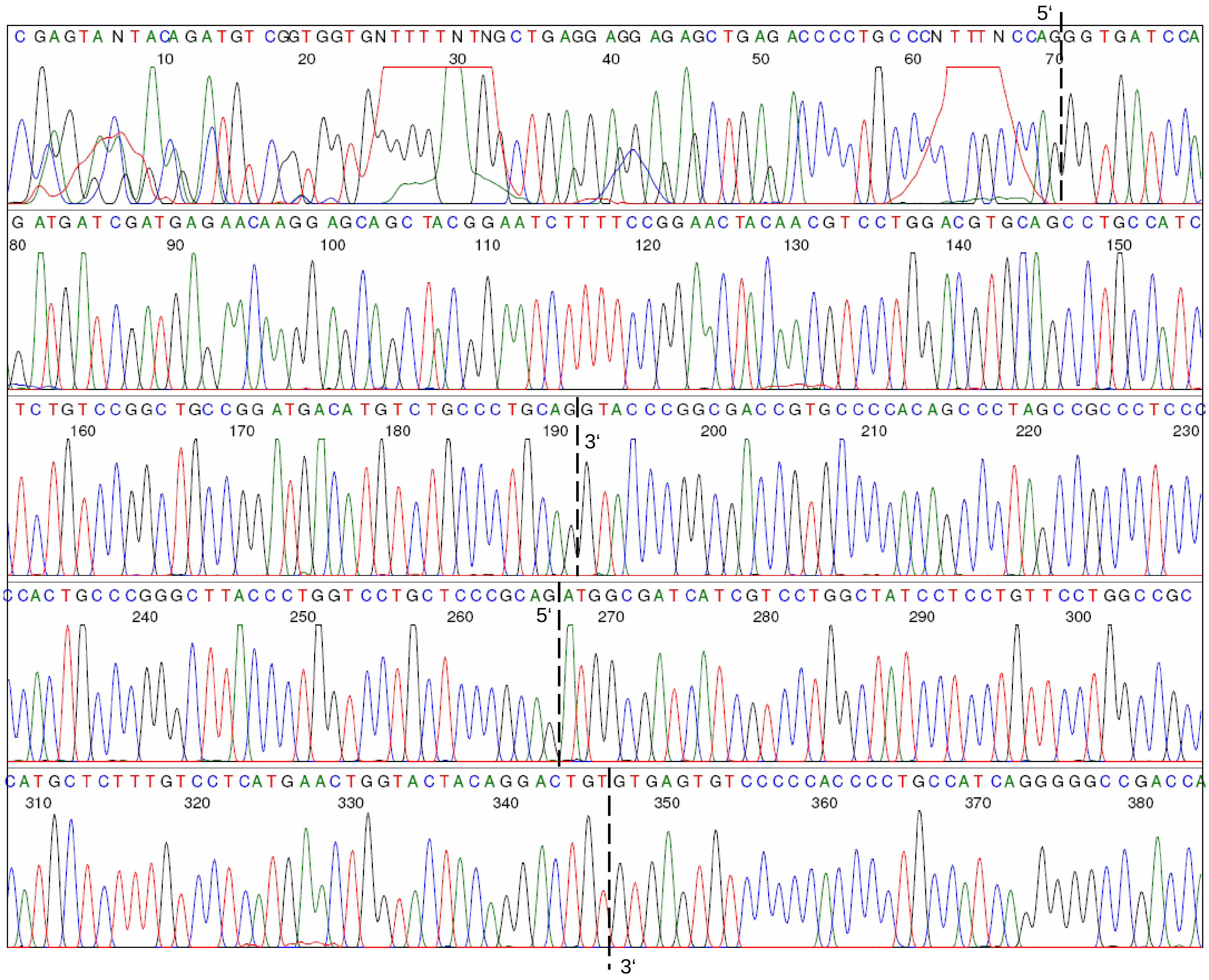

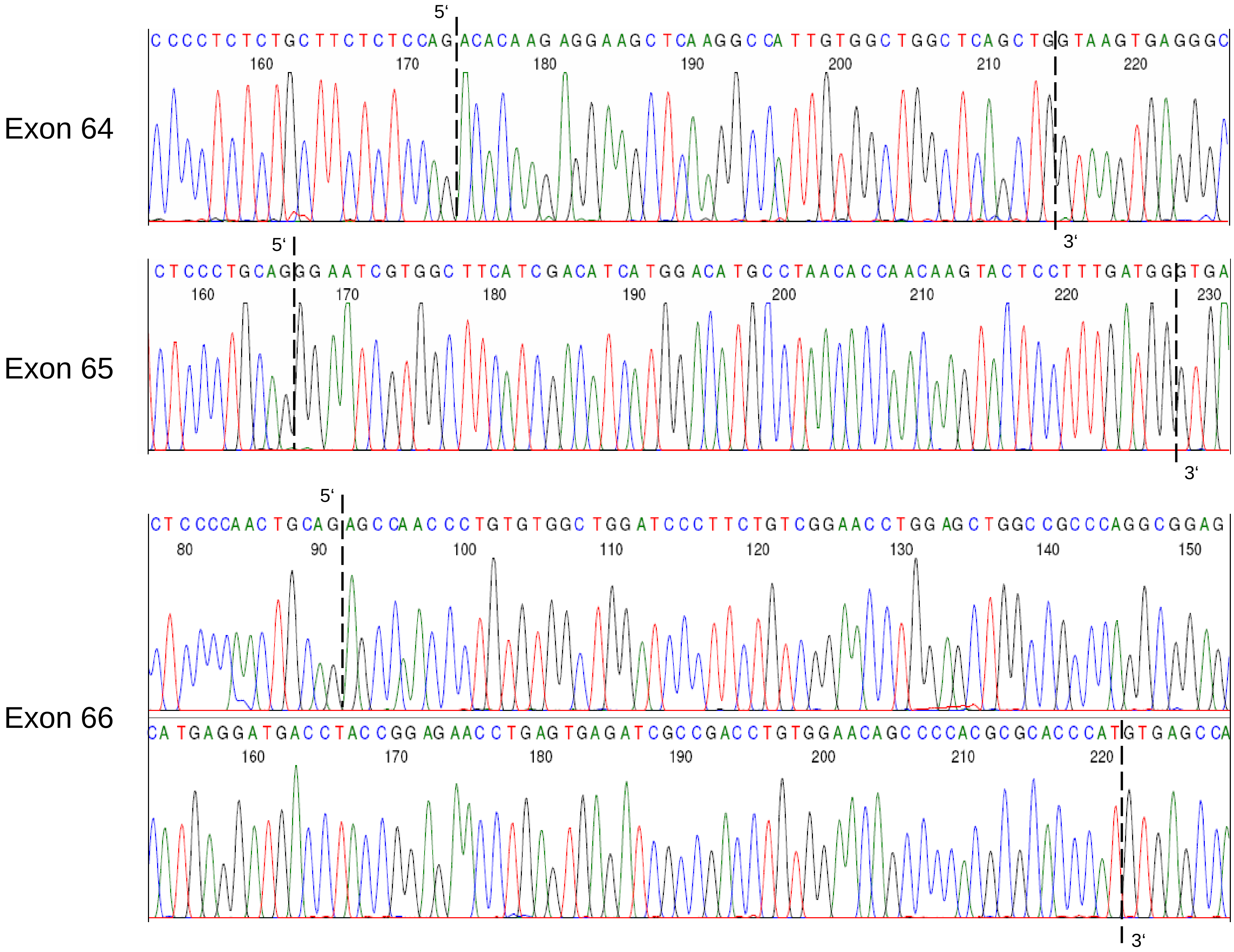
Exon 64
Exon 65
Exon 66

Exon 67

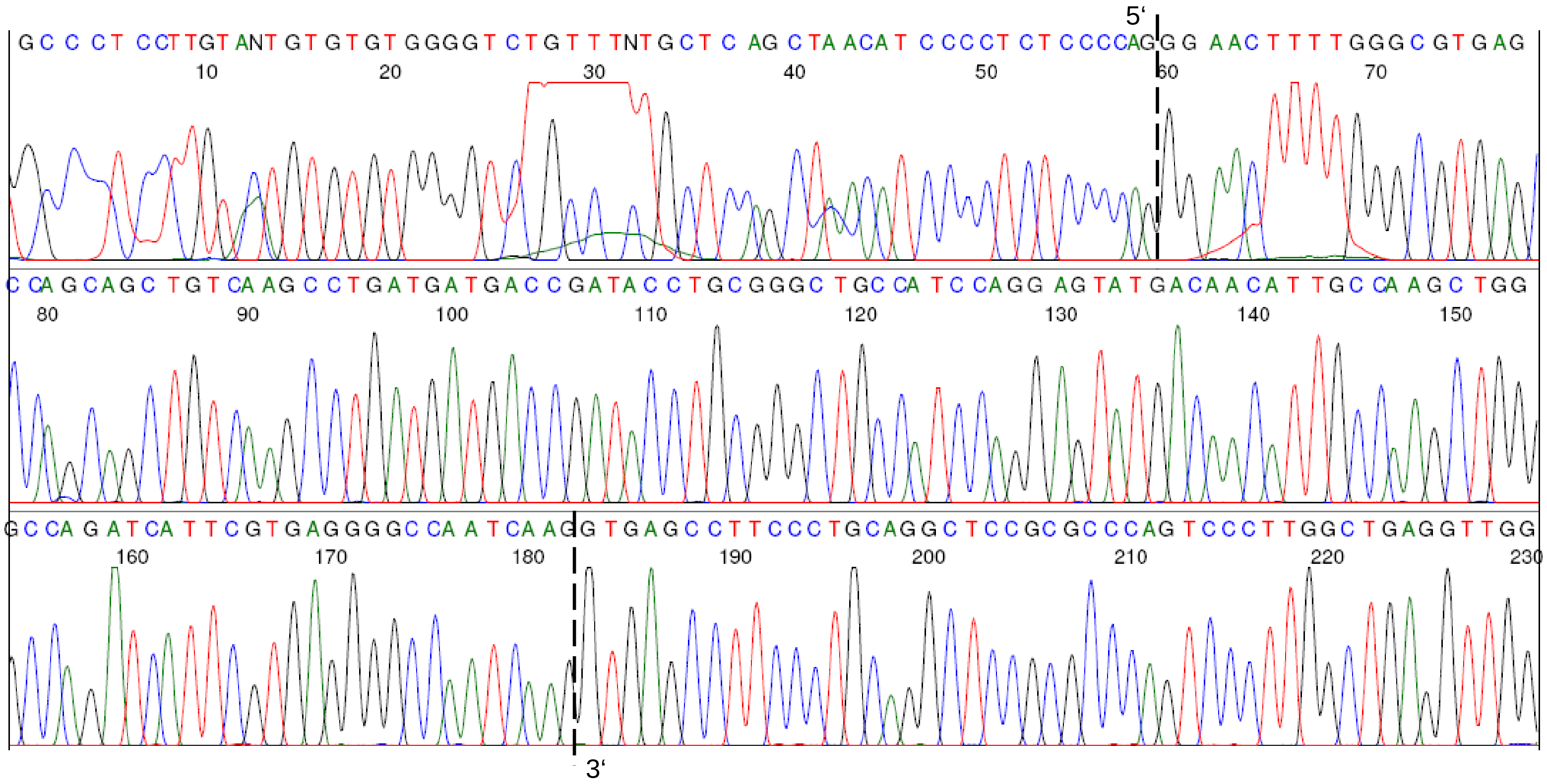

Exon 68

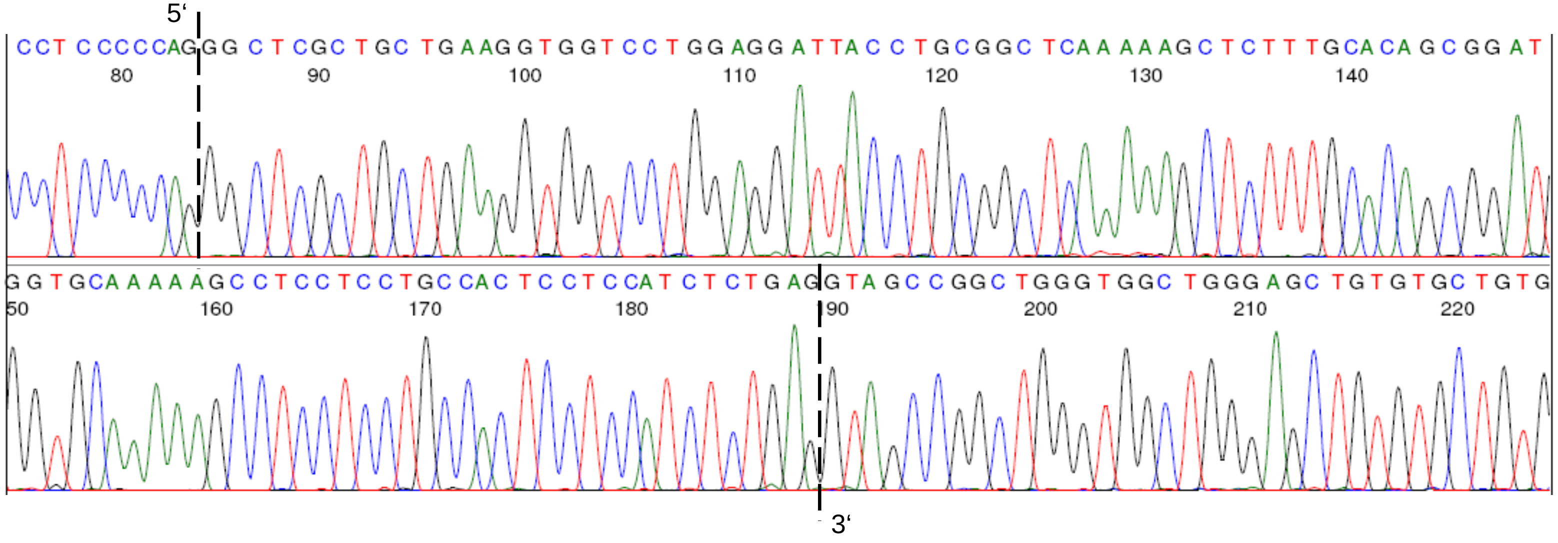

Exon 69

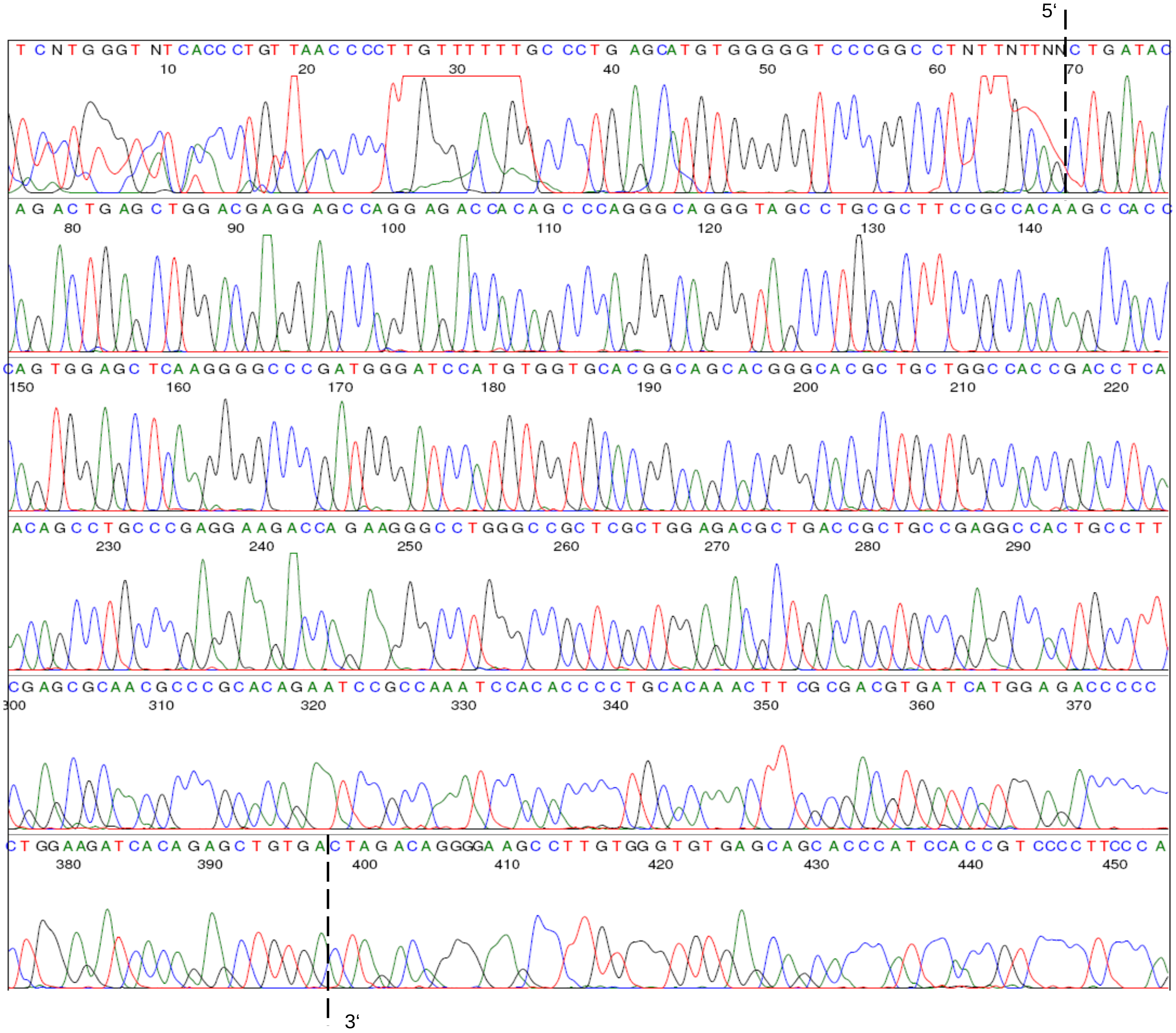

Supplement: Additional data file 8 — Results of mutation screening in patient 1881 in the CDH23 gene (USH1D) (no mutations found). [file gb-2007-8-4-r47-S8.pdf]
