# Supplementary material for: Deafblindness in French Canadians from Quebec: a predominant founder mutation in the USH1C gene provides the first genetic link with the Acadian population
Source: Genome Biol. 2007 Apr 3;8(4):R47. doi: 10.1186/gb-2007-8-4-r47 (PMC1895989; doi:10.1186/gb-2007-8-4-r47)

Deafblindness in French Canadians from Quebec: A predominant founder mutation in the *USH1C* gene provides the first genetic link with the Acadian population

Ebermann et al.

RAW DATA

	page
Haplotypes	
USH1C haplotypes	2 – 23
USH1D haplotypes	24 – 29
USH1 mutations	30 – 36
Genotyping of healthy French Canadian Controls	
Controls for c.216G>A (*USH1C*)	37 – 40
Controls for c.238-239insC (*USH1C*)	41 – 58
Controls for c.496+1G>T (*USH1C*)	59 – 60
Controls for p.R155X (*USH1C*)	61 – 62
Controls for c.748-759+5del (*USH1C*)	63 – 64
Controls for IVS45-9G>A (*CDH23*)	65 – 73
Controls for p.R736X (*CDH23*)	74 – 82
Controls for p.A457V (*MYO7A*)	83 – 91
Controls for p.Q815X (*MYO7A*)	92 – 93
Controls for p.A123D (*USH3A*)	94 – 102
Mutation screening in USH1 genes in patient 1881	103 – 204

Patient 1881, *PCDH15* (*USH1F*)

Exon 2

Exon 3

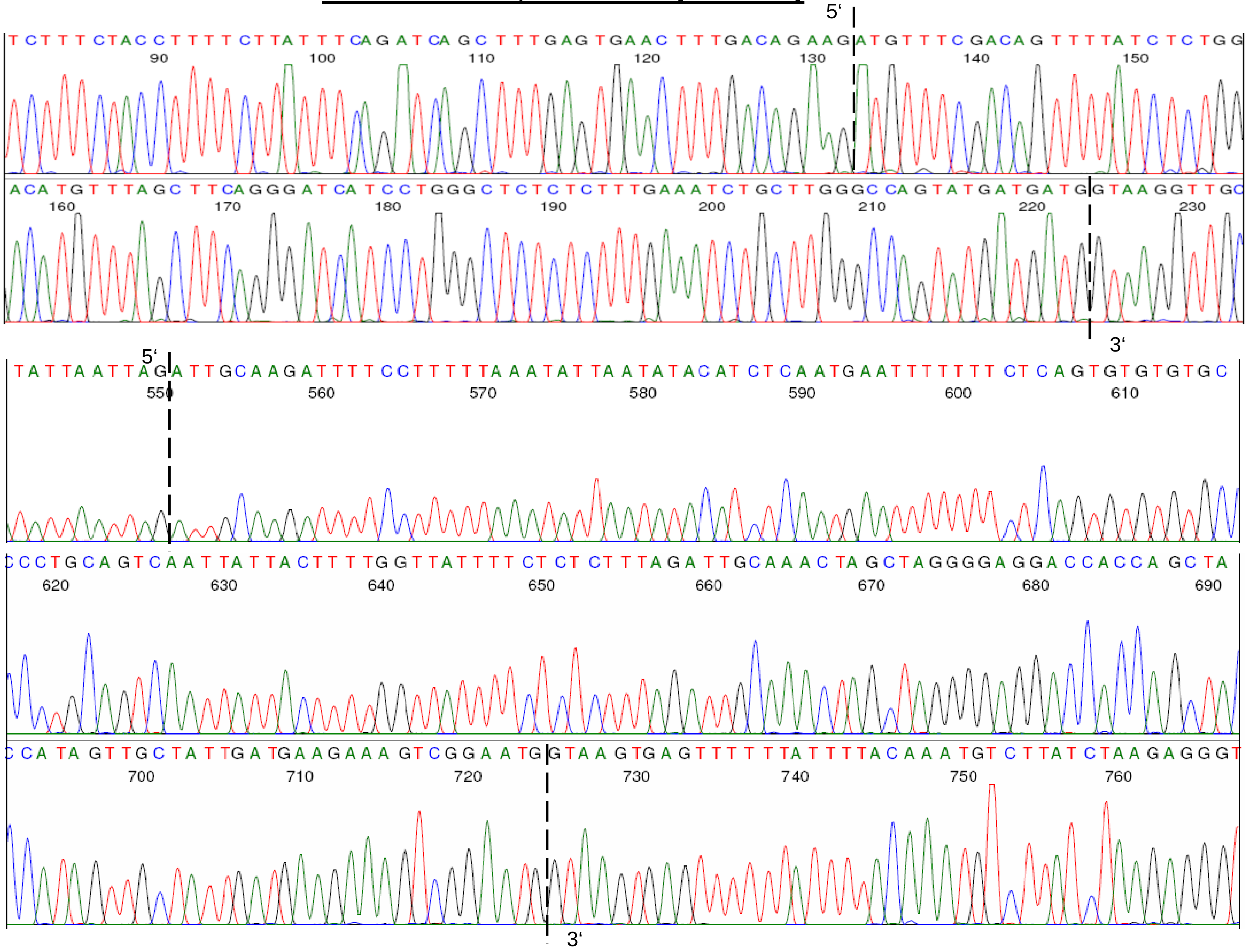

Exon 4

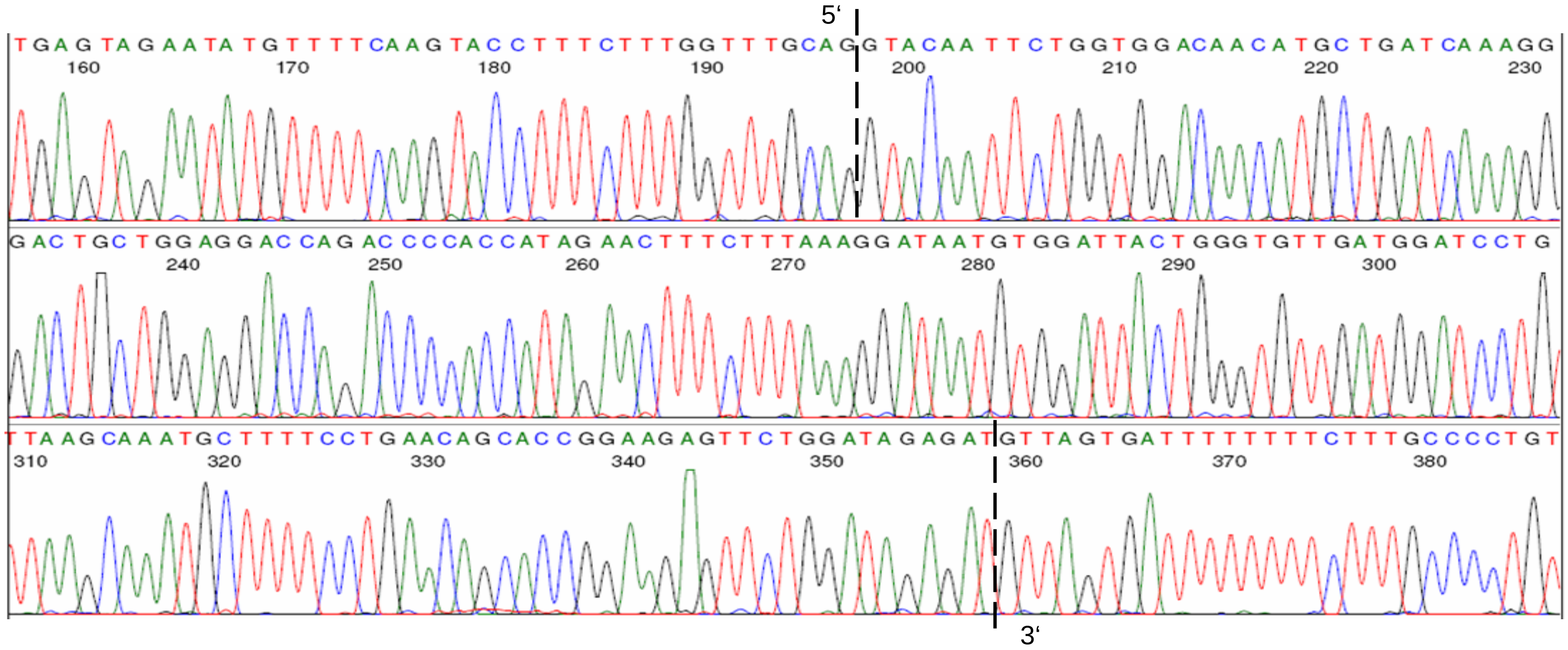

Exon 5

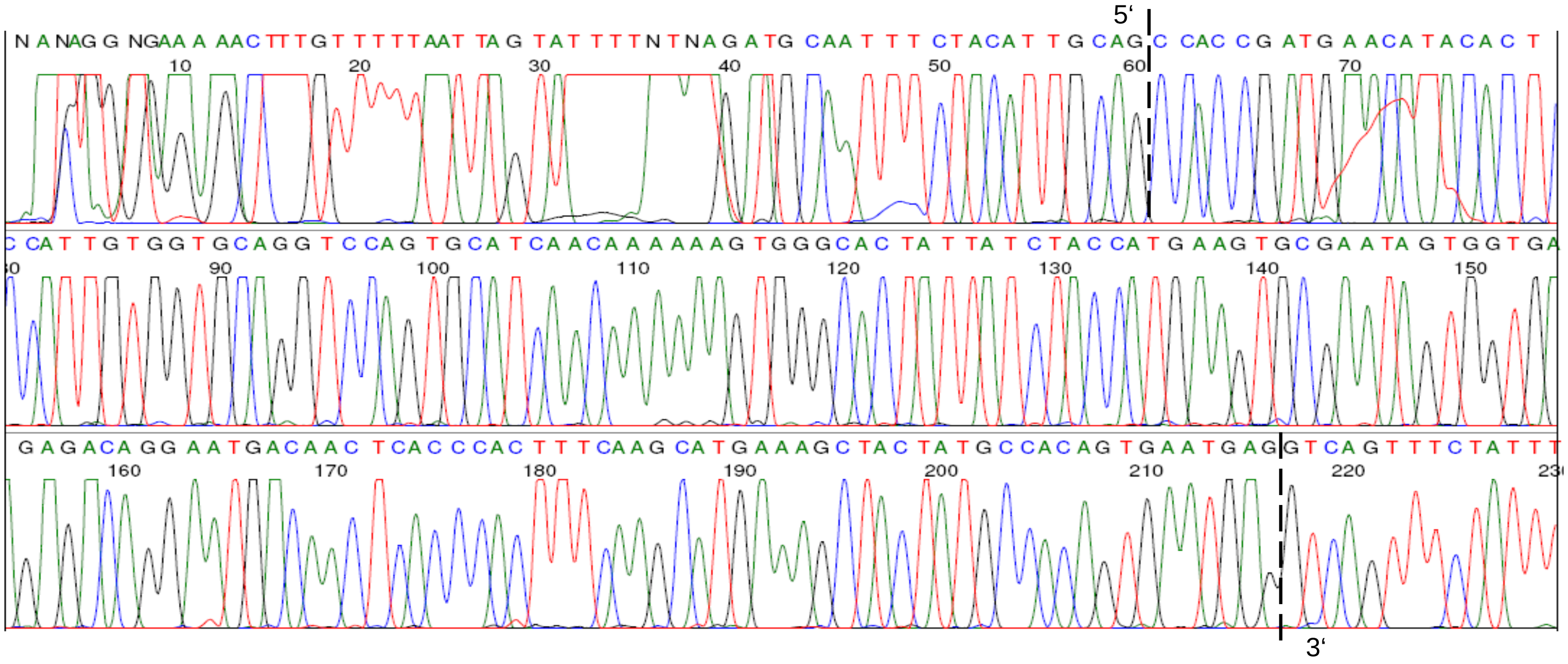

Exon 6

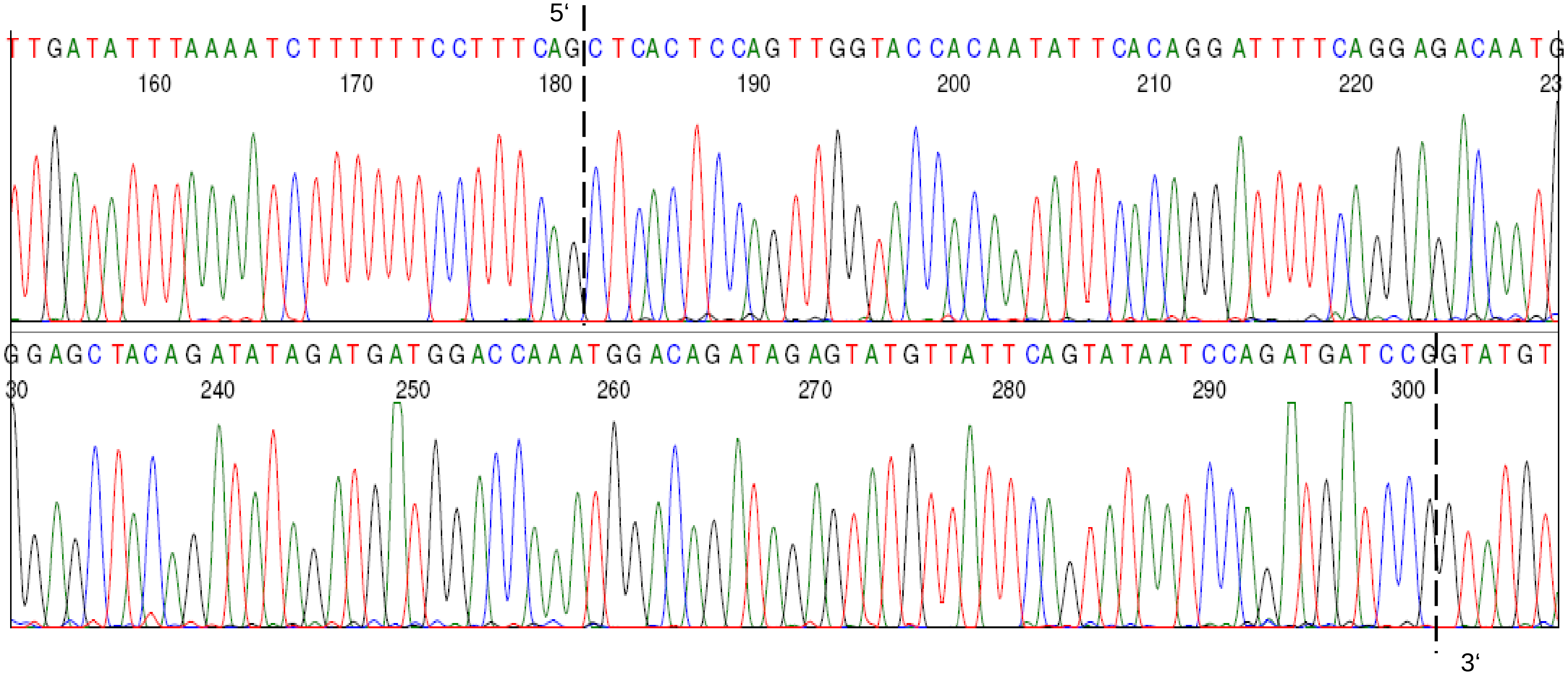
5‘
TTGATATTTAAAATCTTTTTTCCTTTCAGCTCACTCCAGTTGGTACCACAATATTCACAGGATTTTCAGGAGACAATG
GGAGCTACAGATATAGATGATGGACCAAATGGACAGATAGAGTATGTTATTCAGTATAATCCAGATGATCCGGTATGT
3‘

Exon 7

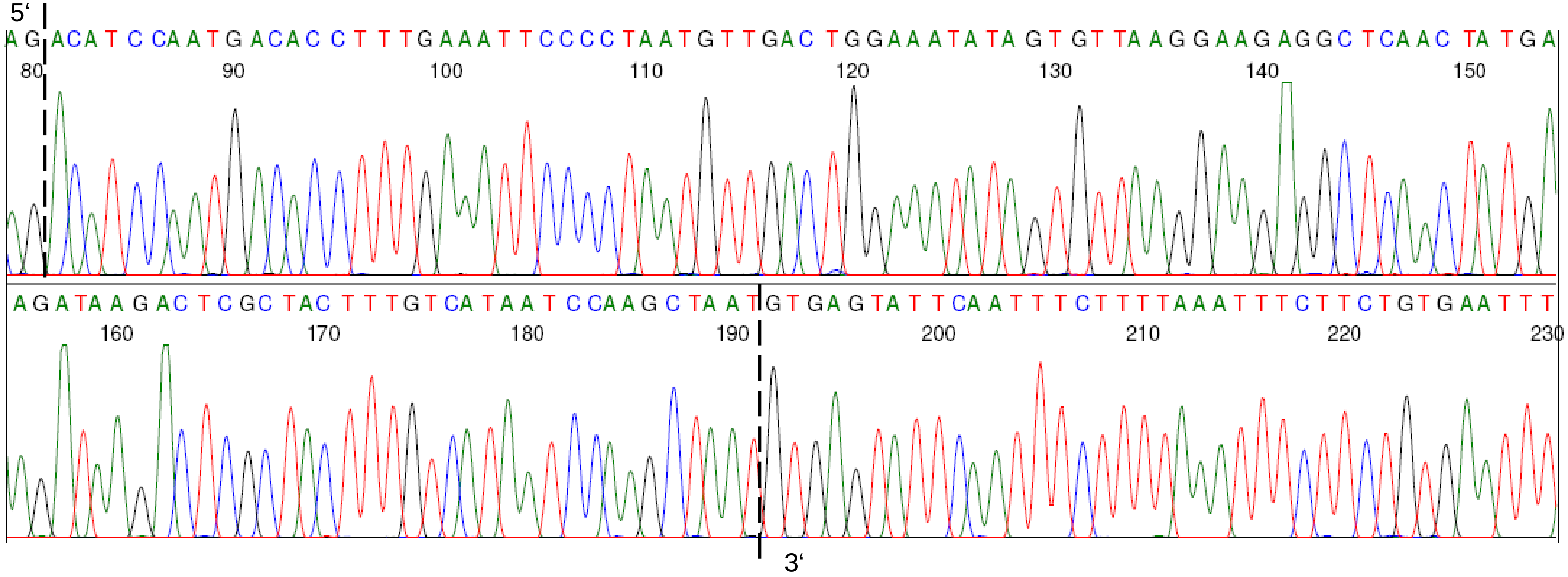
5‘
AGACATCCAATGACACCTTTGAAATTCCCCTAATGTTGACTGGAAATATAGTGTTAAGGAAGAGGCTCAACTATGA
AGATAAGACTCGCTACTTTGTCATAATCCAAGCTAATGTGAGTATTCAATTTCTTTTAAATTTCTTCTGTGAATTT
3‘

Exon 8

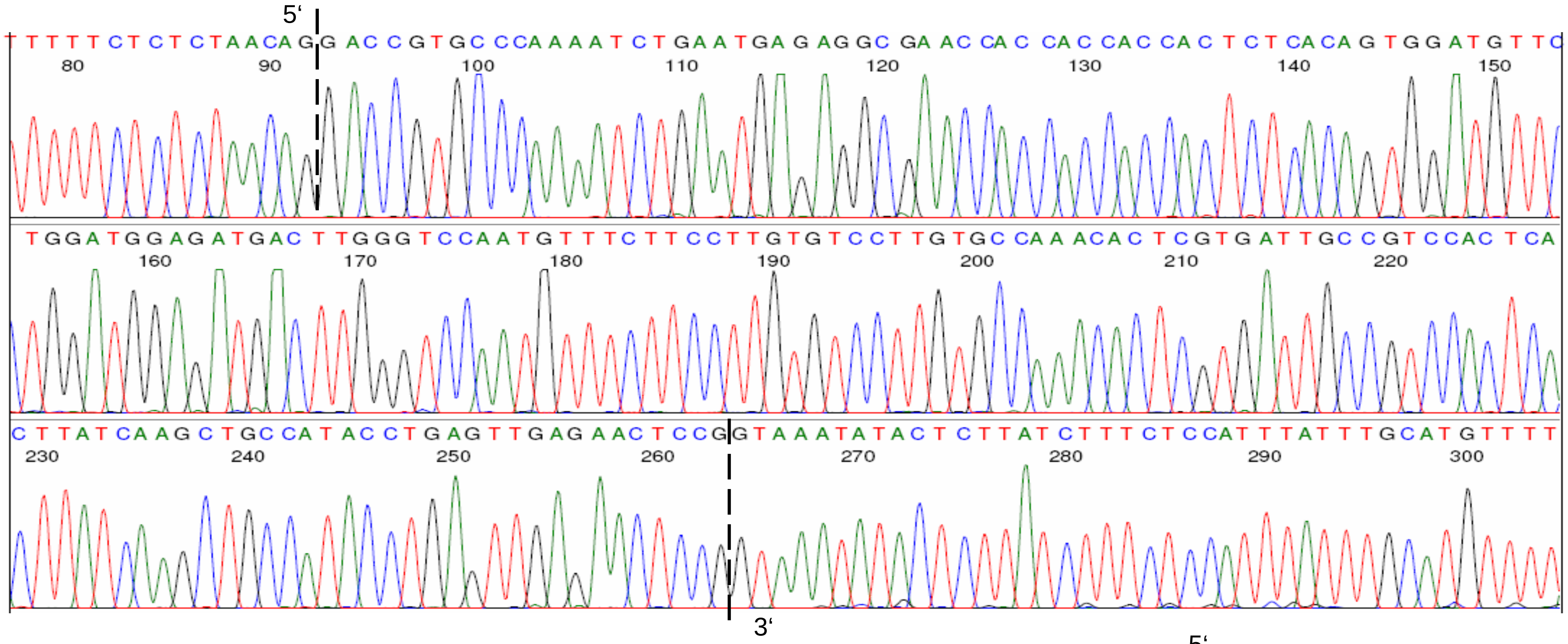

Exon 9

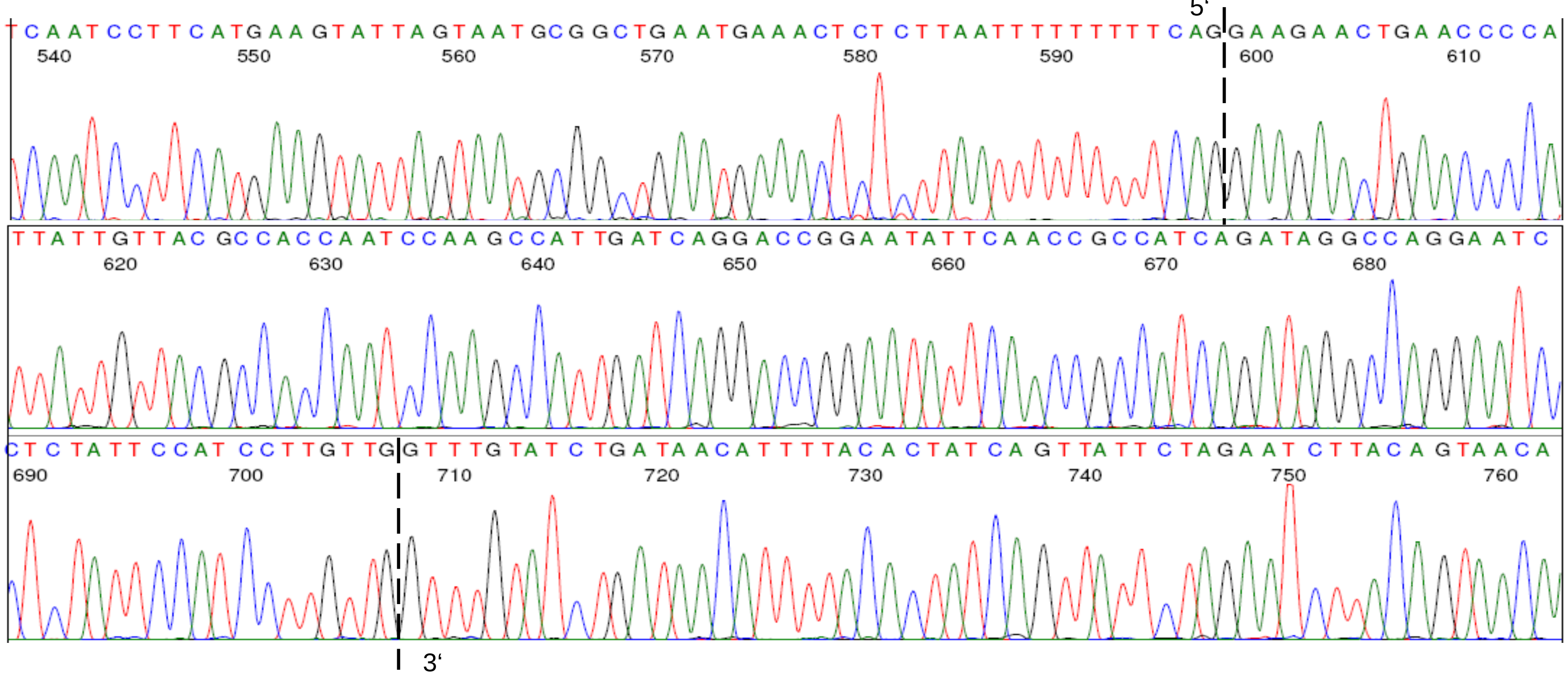

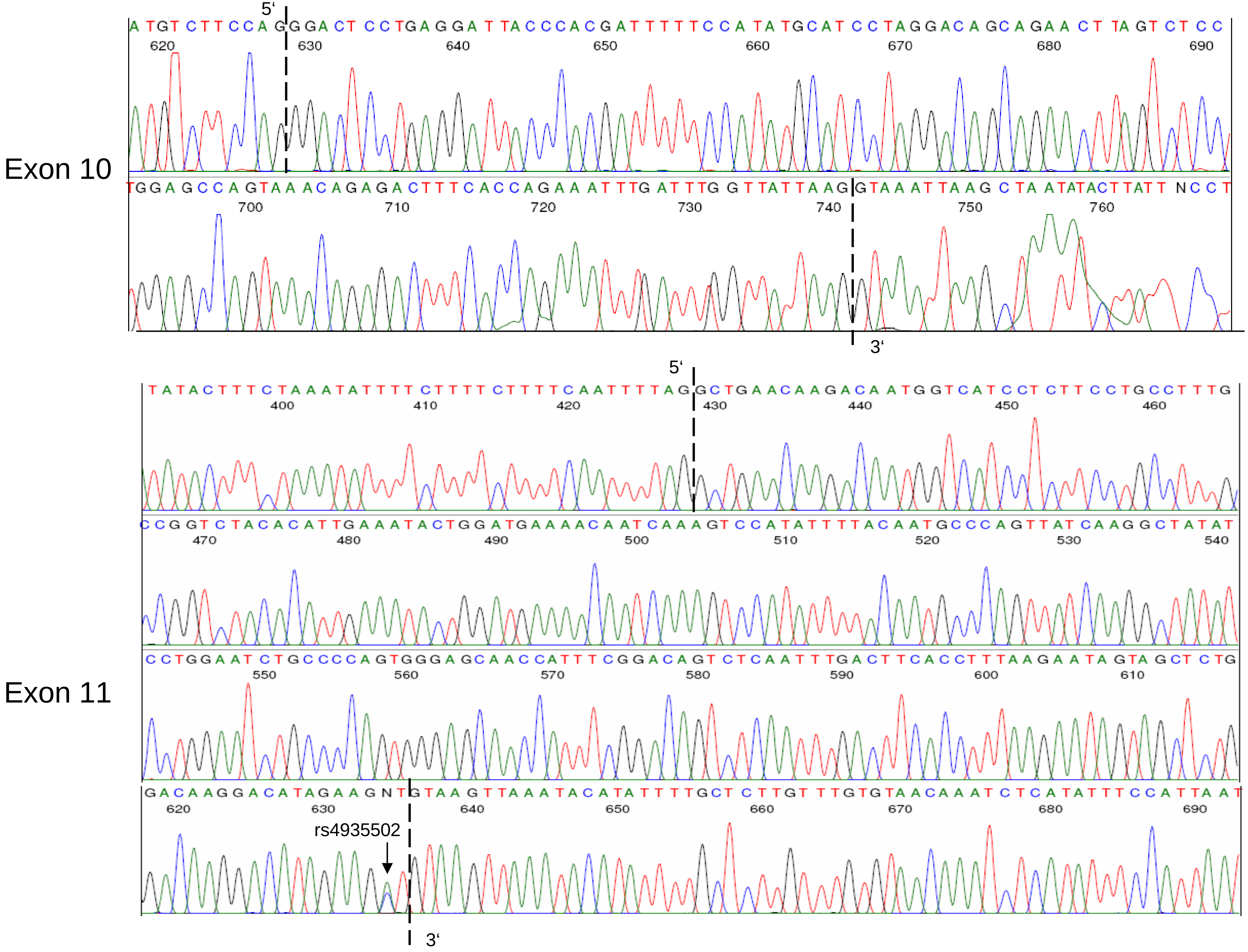
Exon 10
5‘
3‘
Exon 11
5‘
rs4935502
3‘

Exon 12

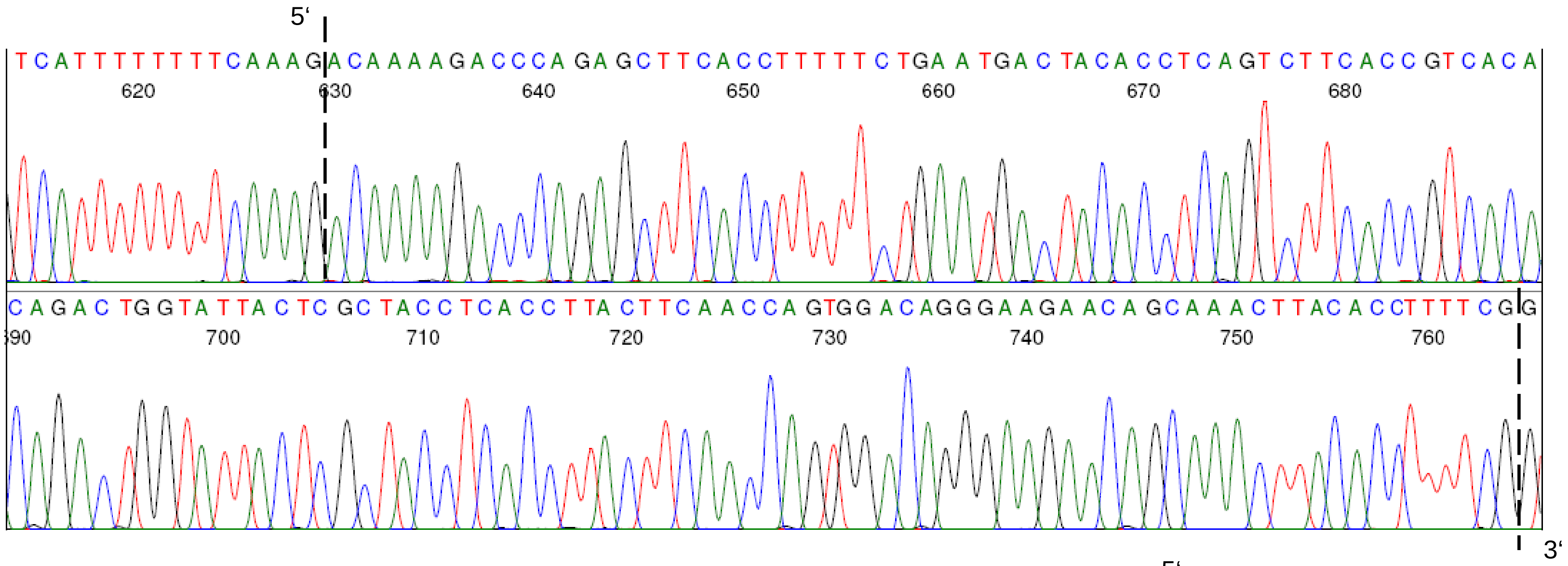

Exon 13

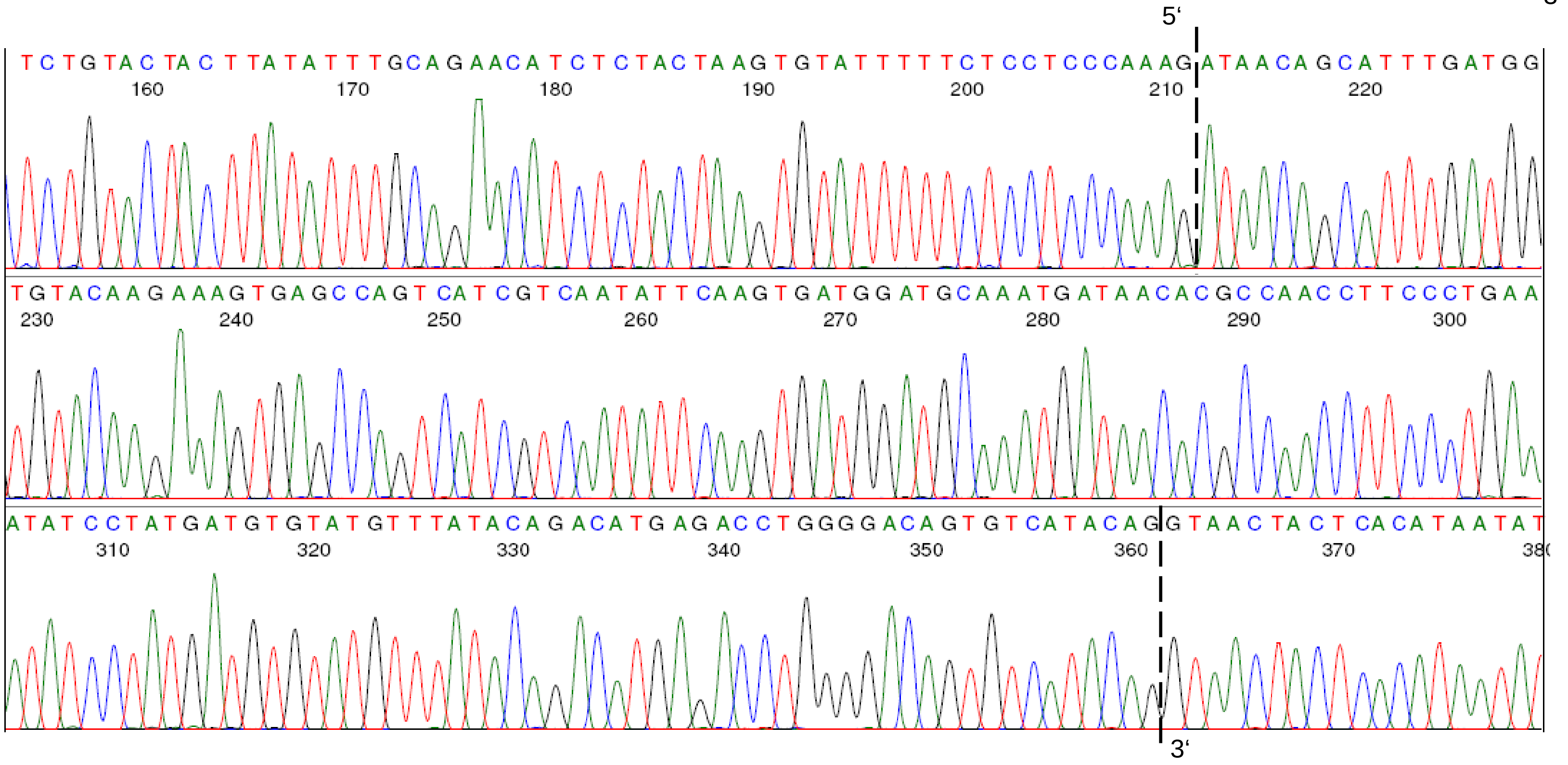

Exon 14

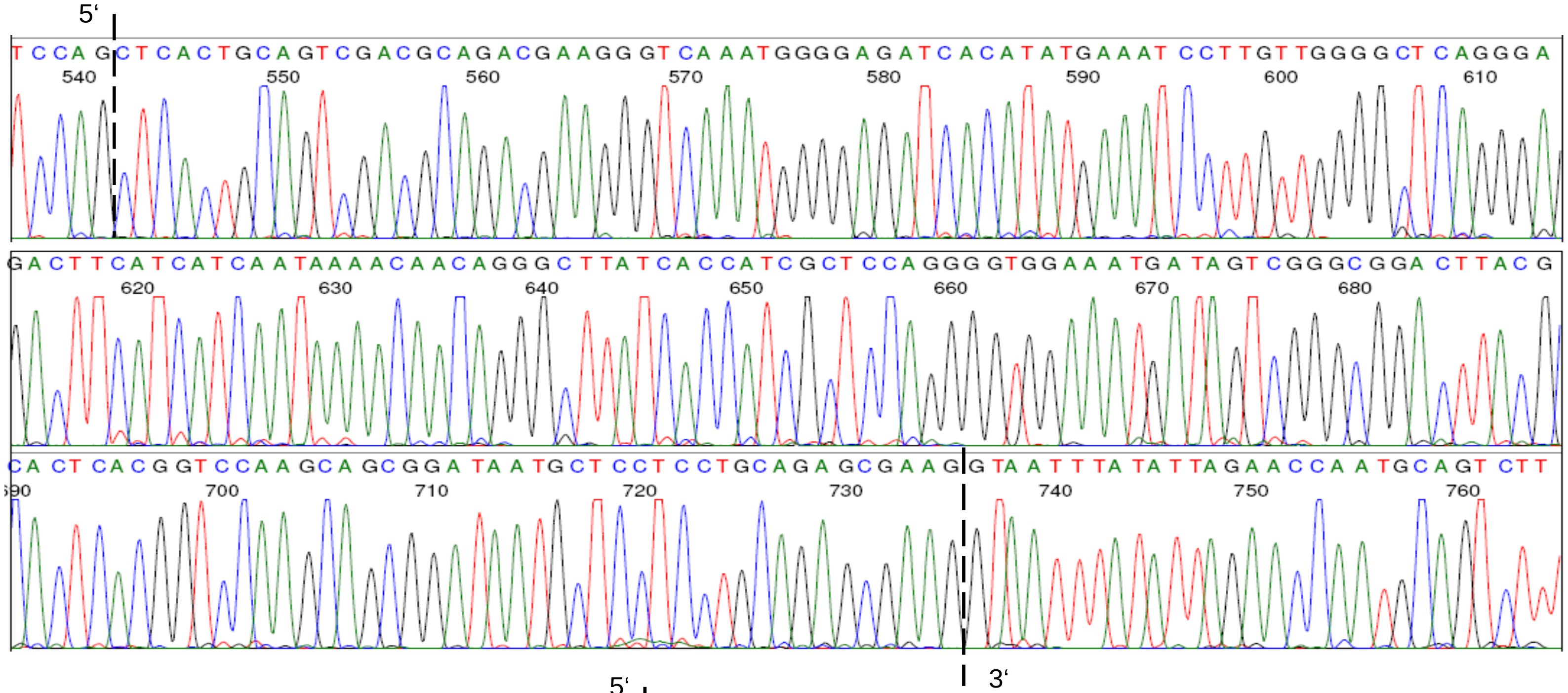

Exon 15

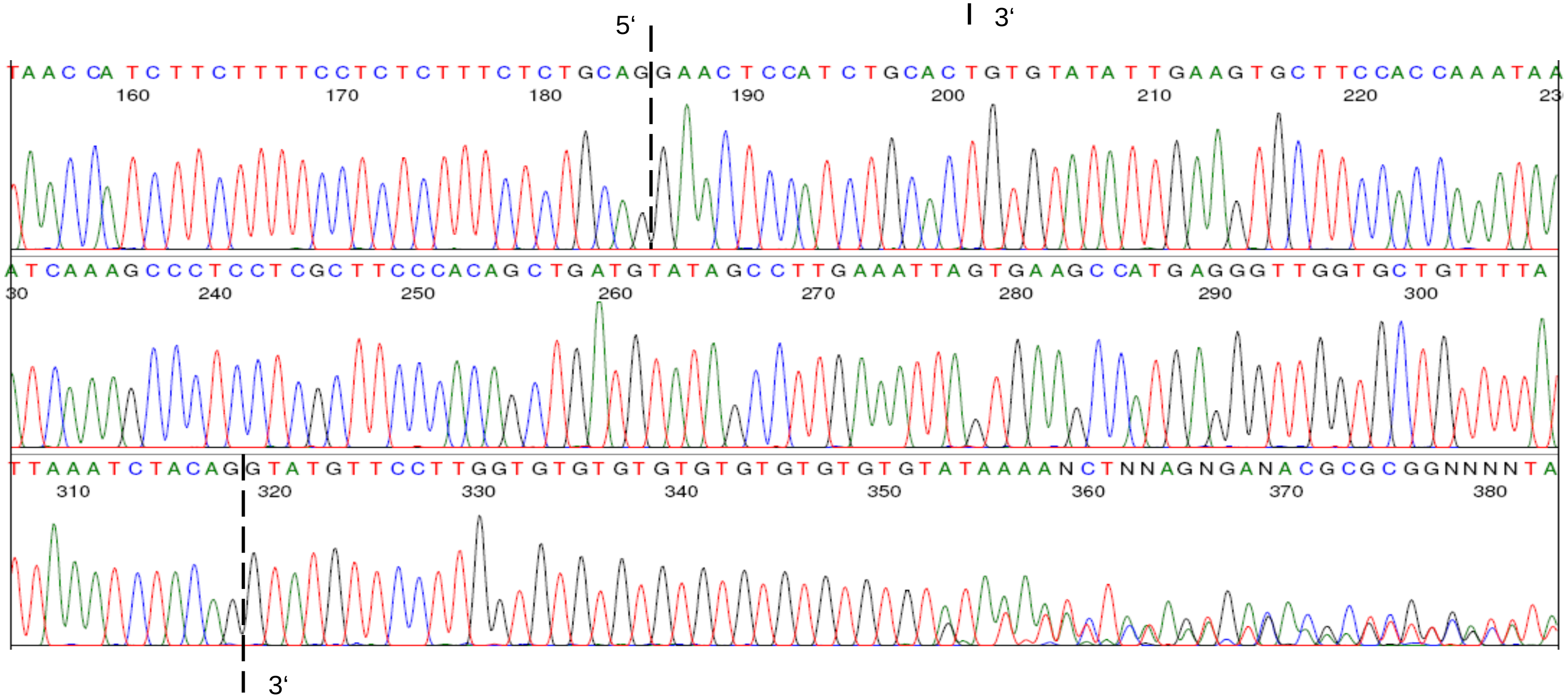

Exon 16

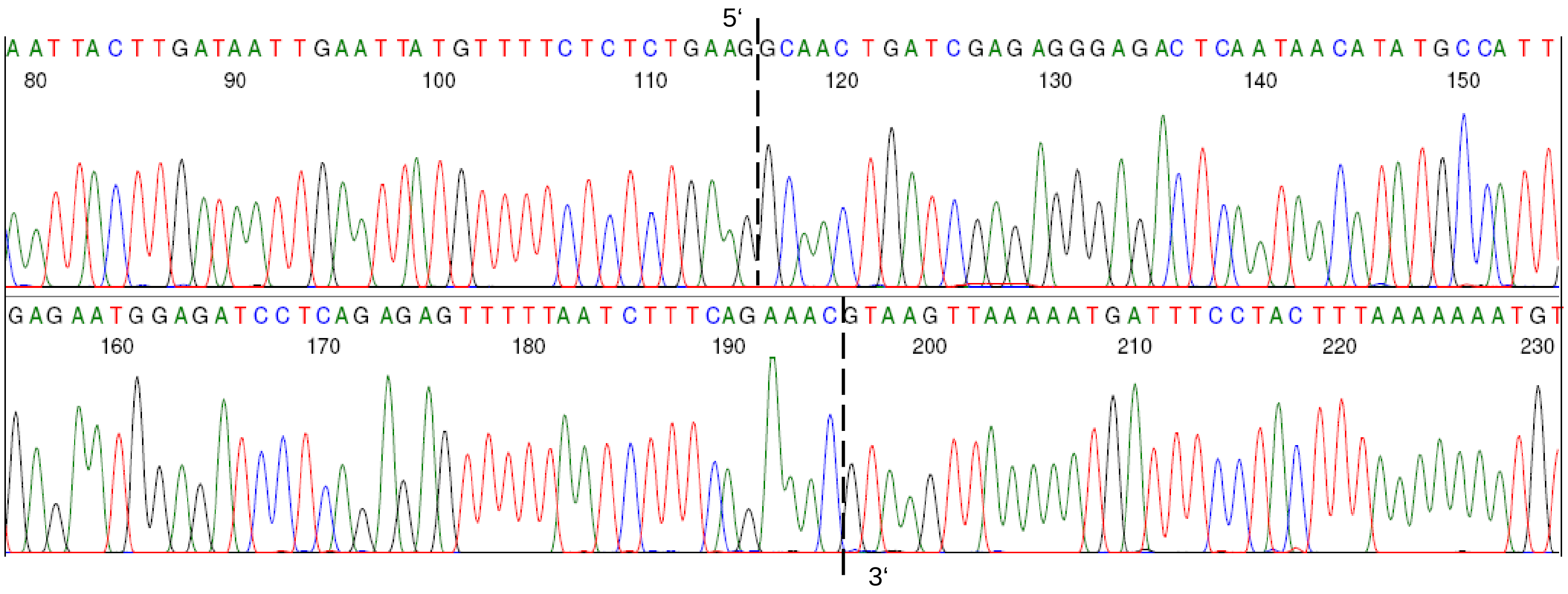

Exon 17

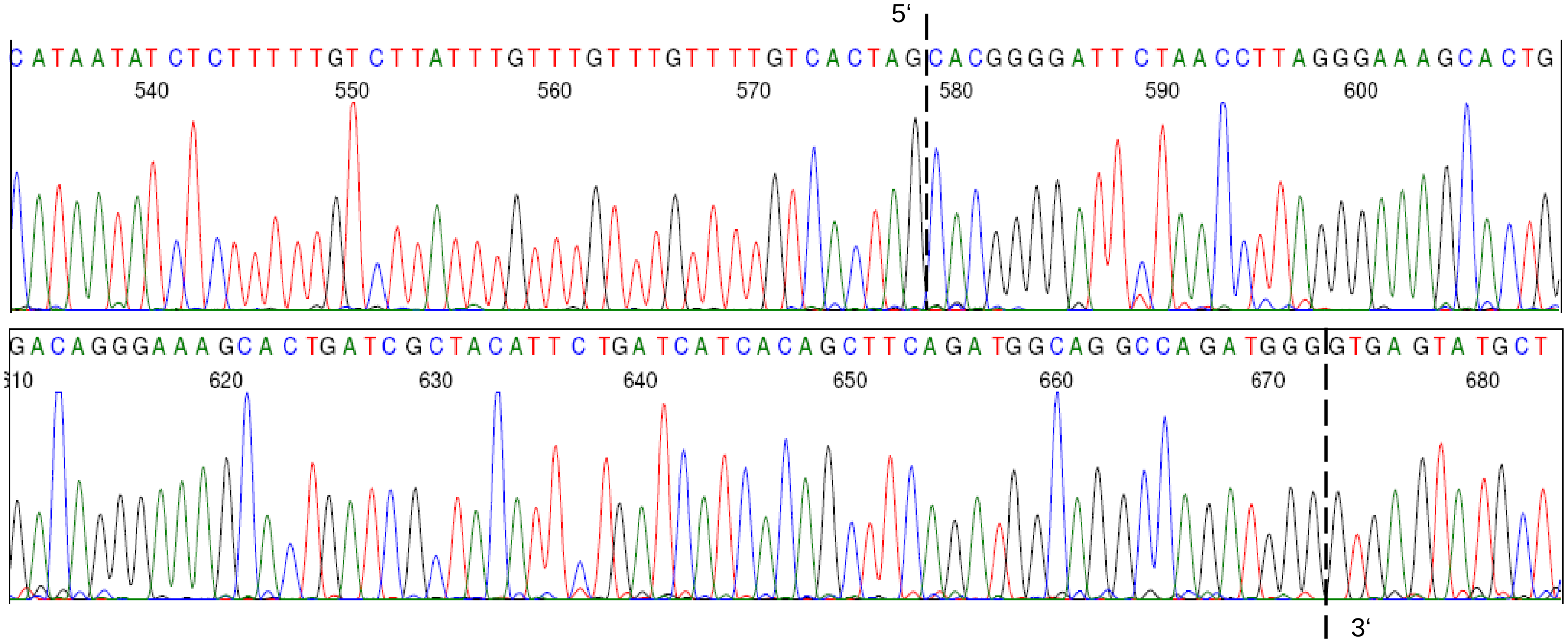

Exon 18

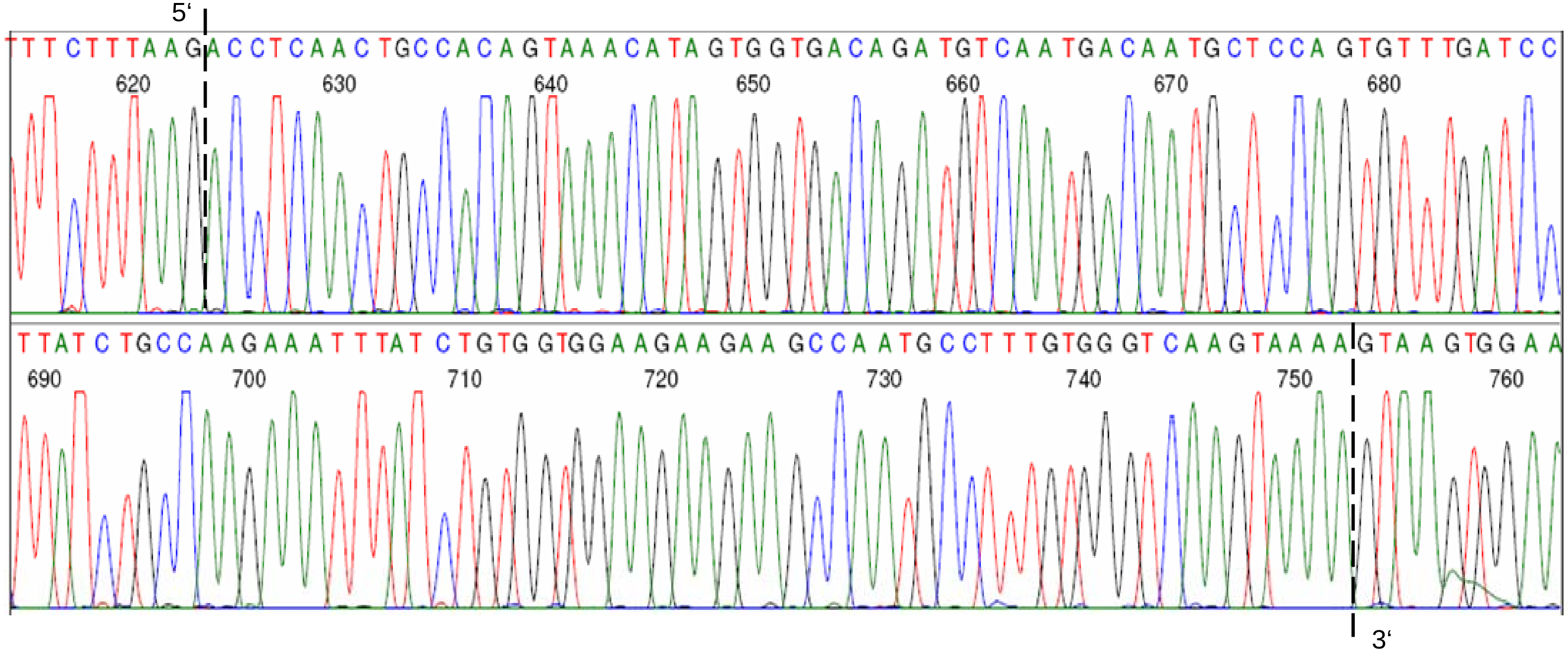

5‘
TTTCTTTAAGACCTCAACTGCCACAGTAAACATAGTGGTGACAGATGTCAATGACAATGCTCCAGTGTTTGATCC
620
630
640
650
660
670
680
TTATCTGCCAAGAAATTTATCTGTGGTGGAAGAAGAAGCCAATGCCTTTGTGGGTCAAGTAAAAGTAAGTGGAA
690
700
710
720
730
740
750
760
3‘

Exon 19

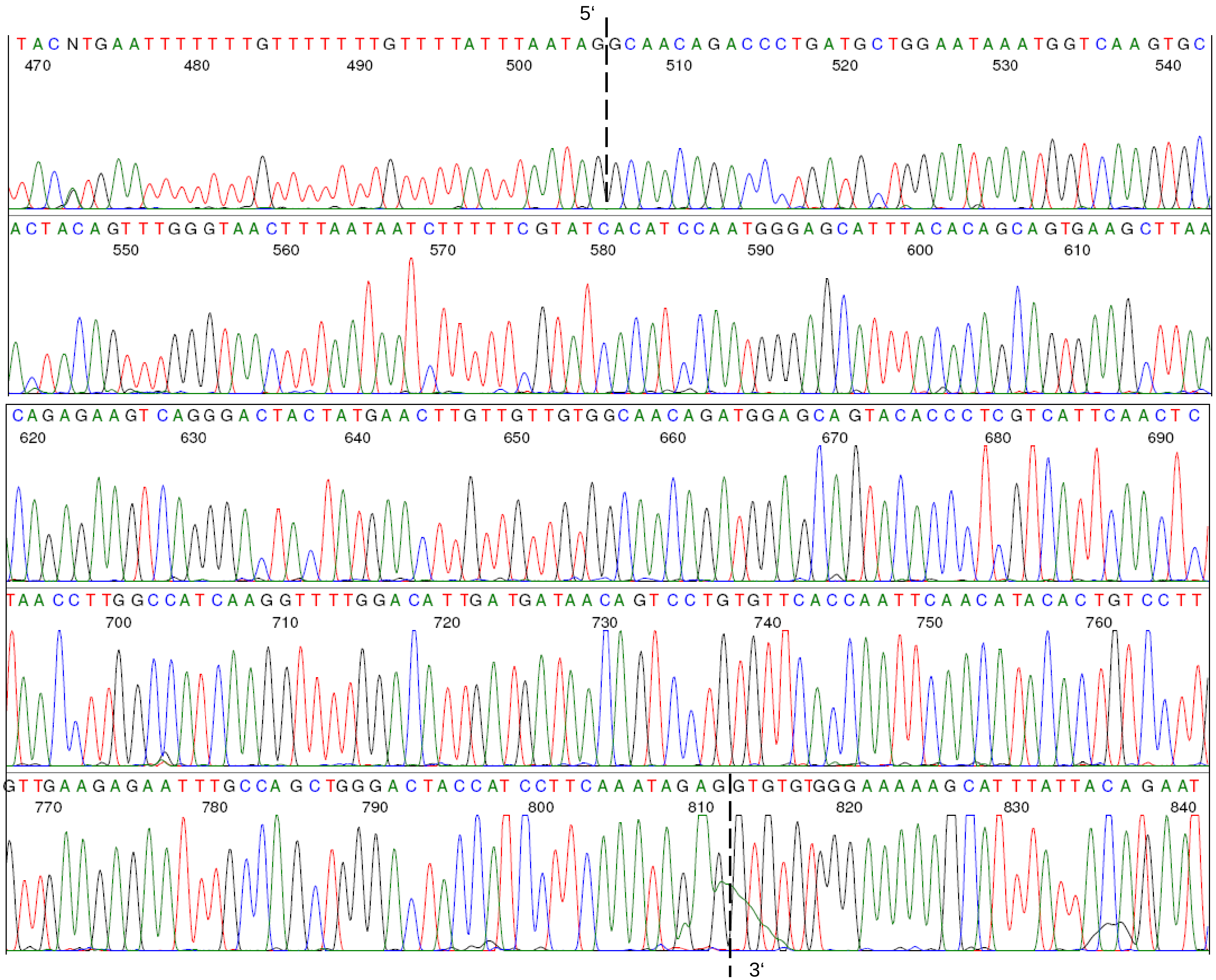

Exon 20

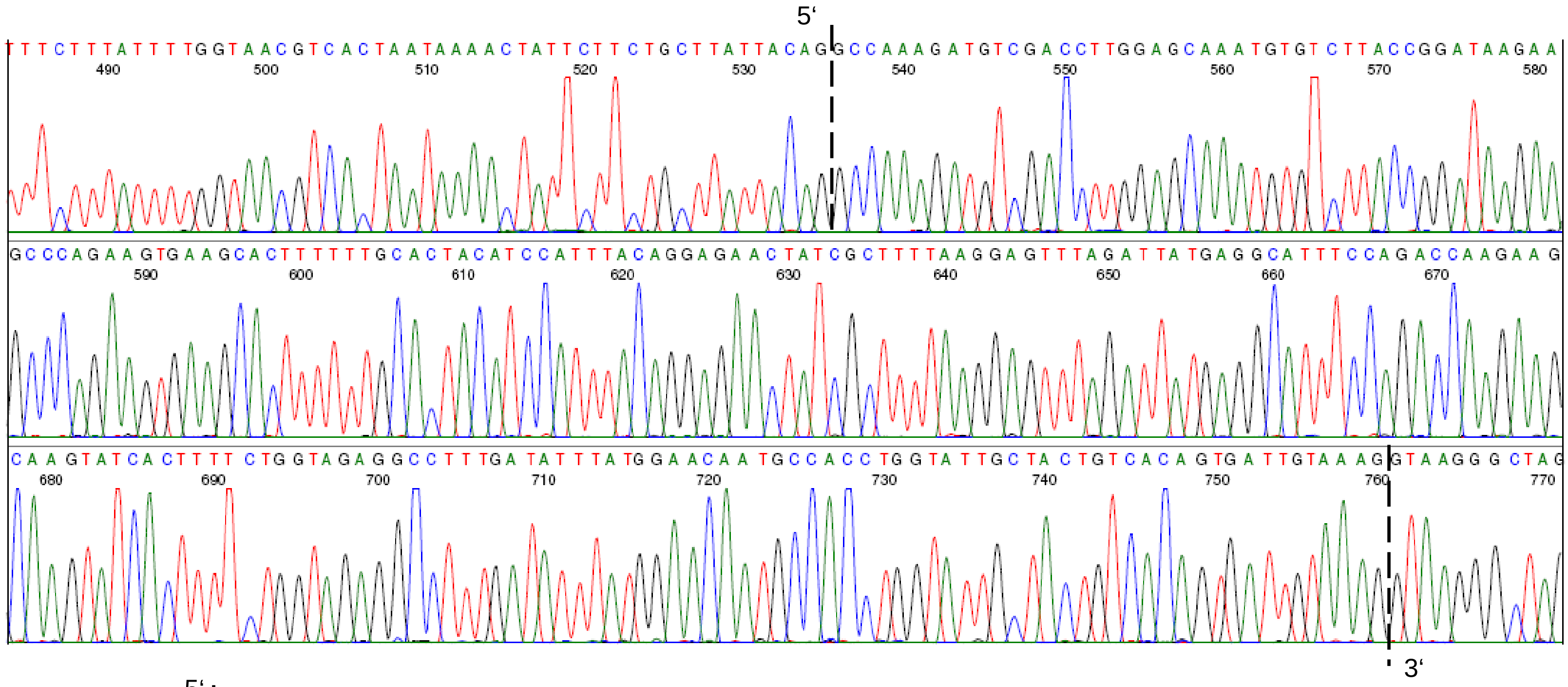

Exon 21

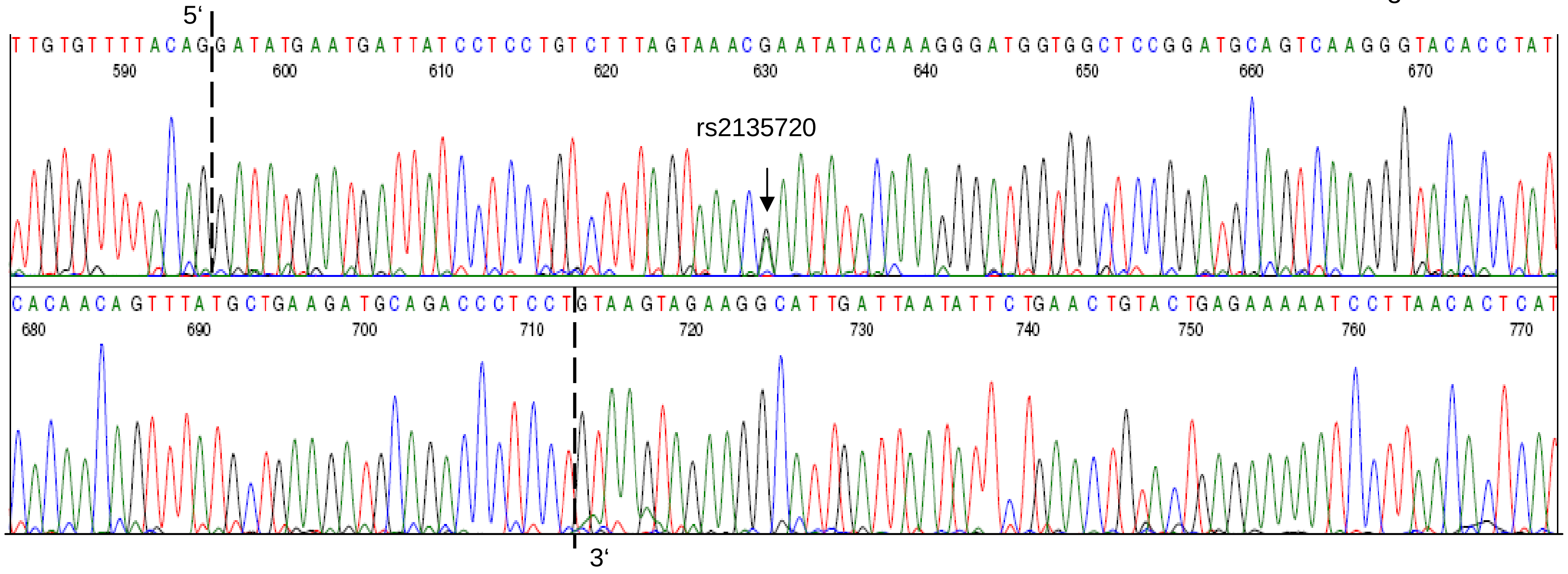

Exon 22

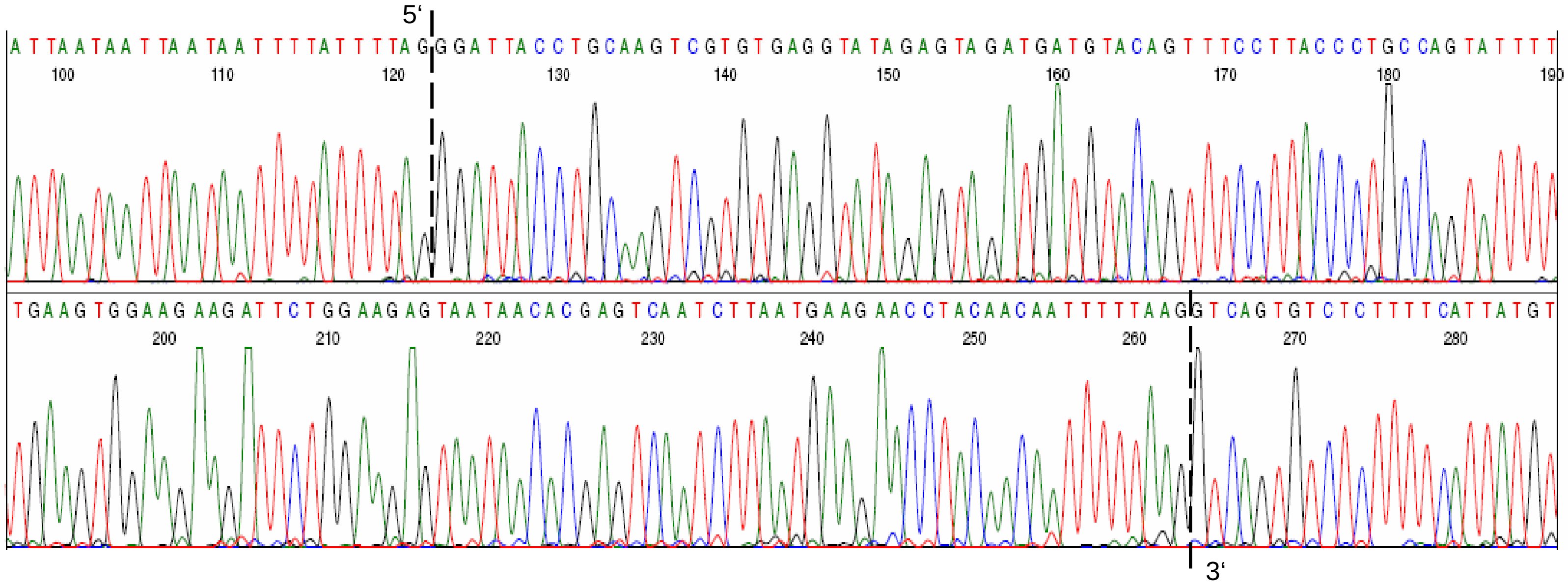

Exon 23

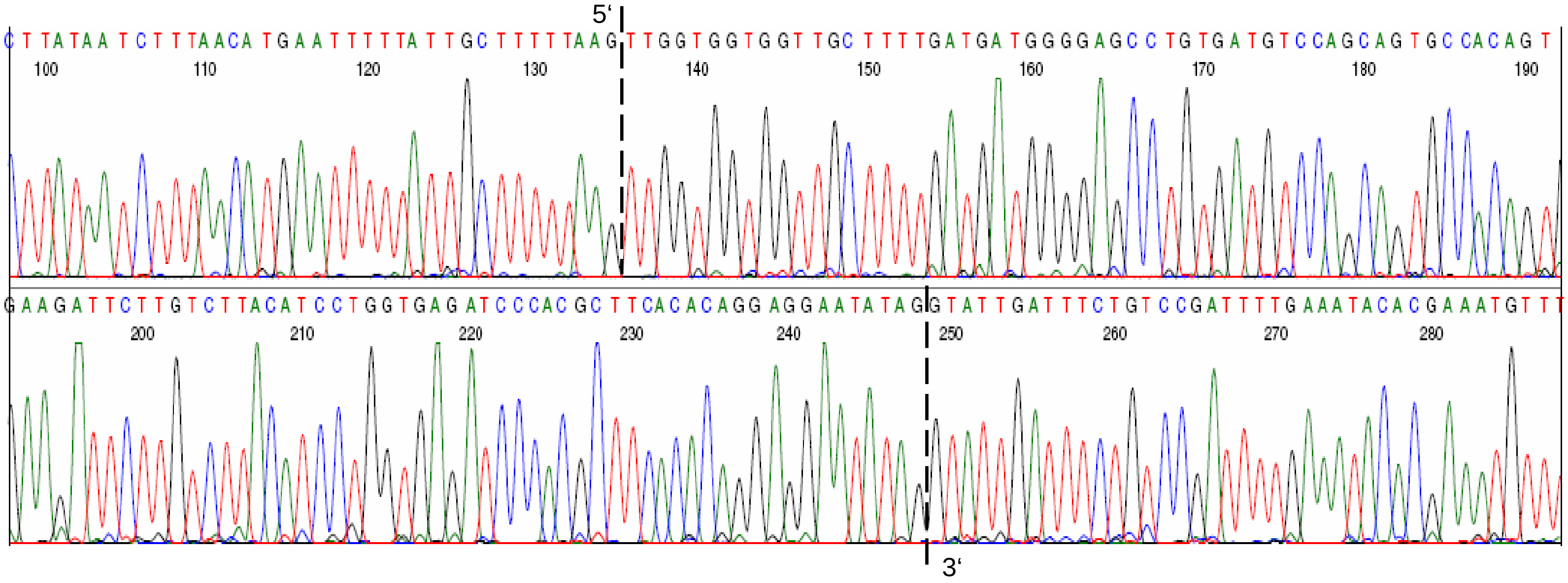

Exon 24

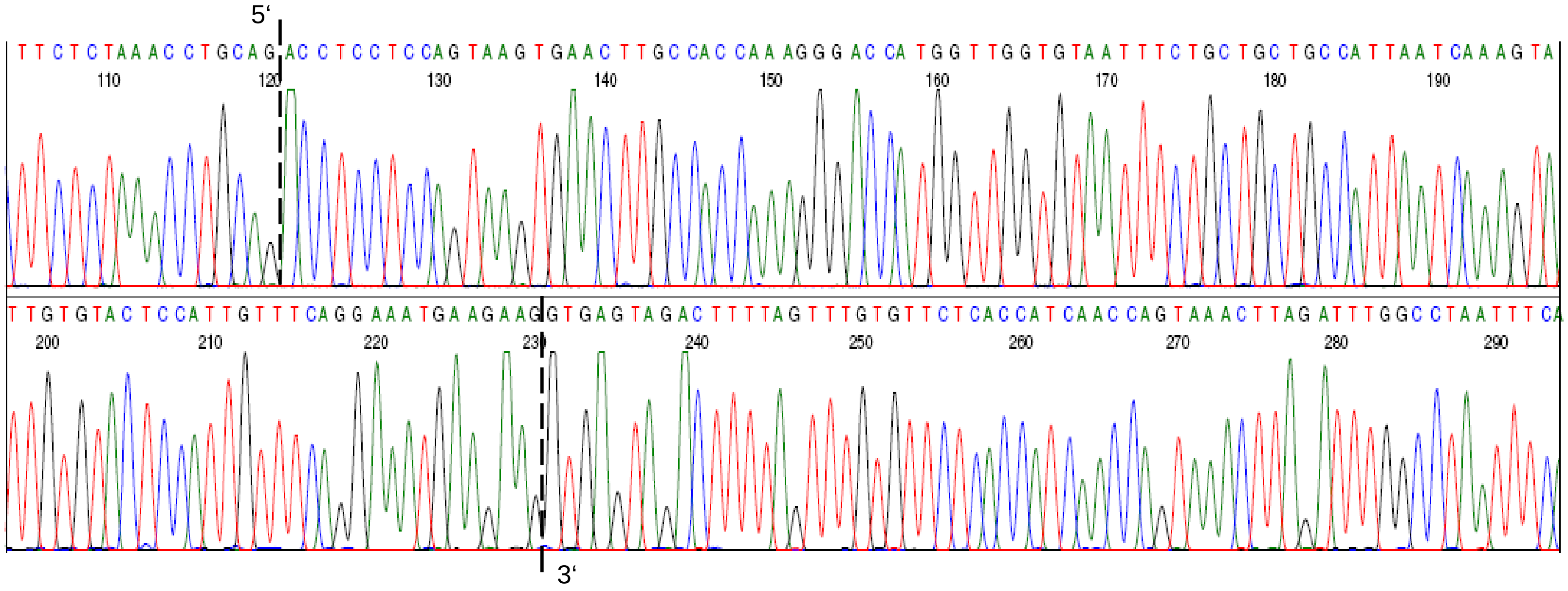

Exon 25

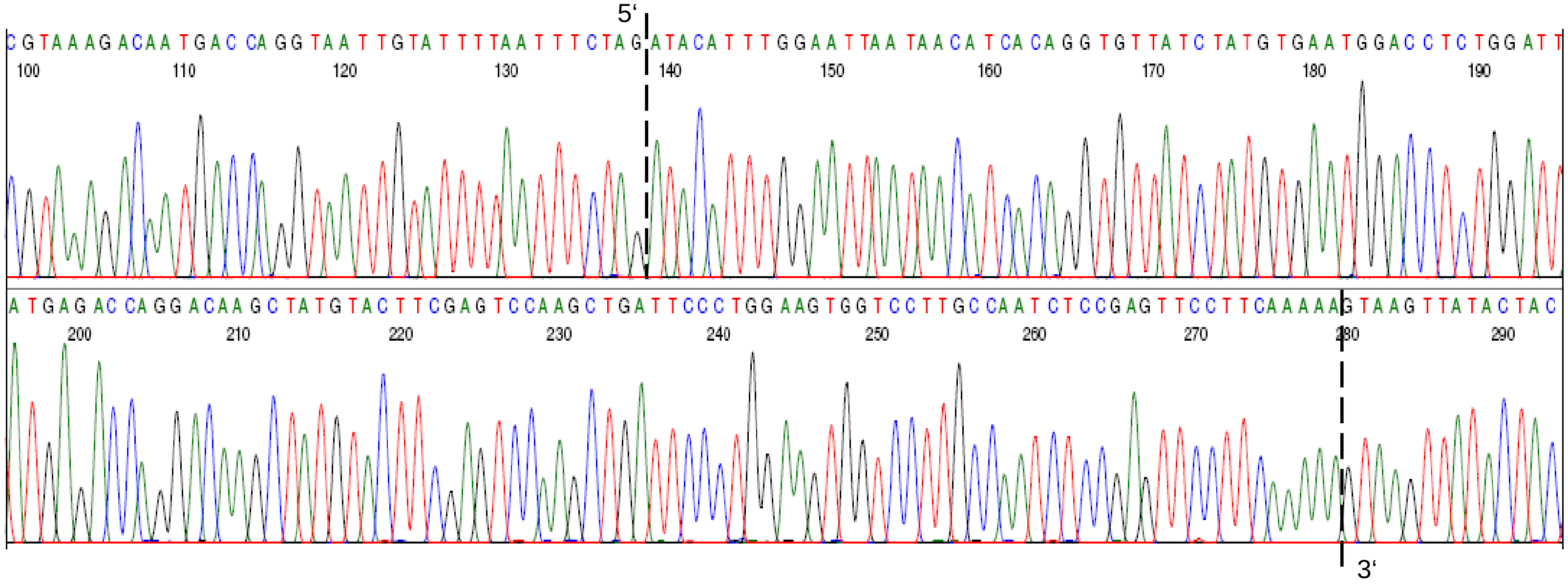

Exon 26

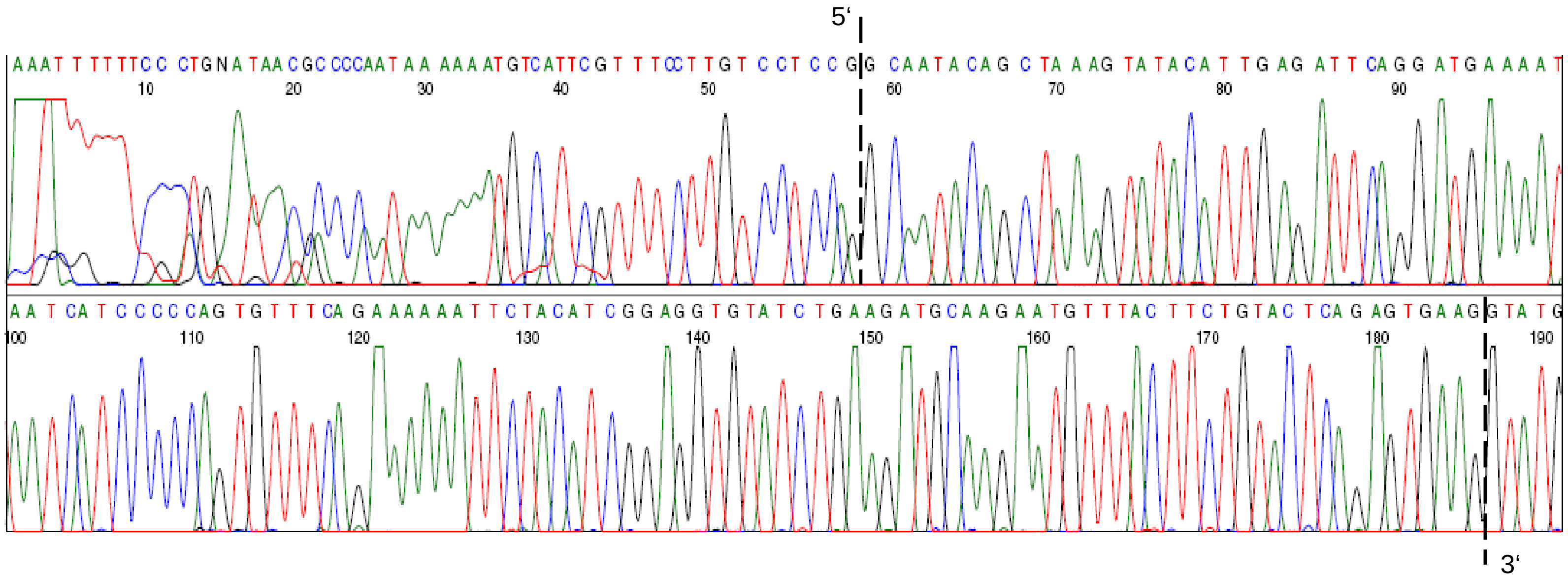

Exon 27

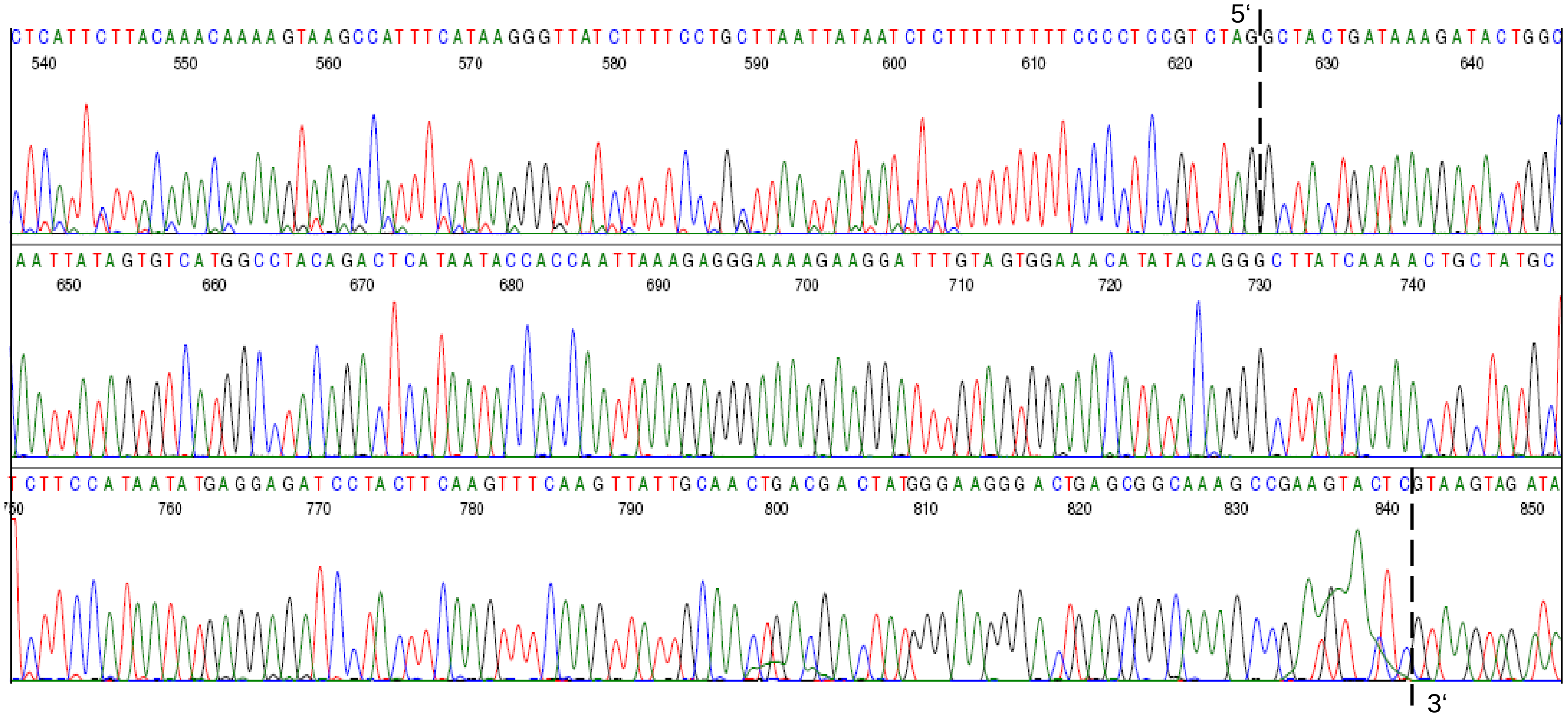

Exon 28

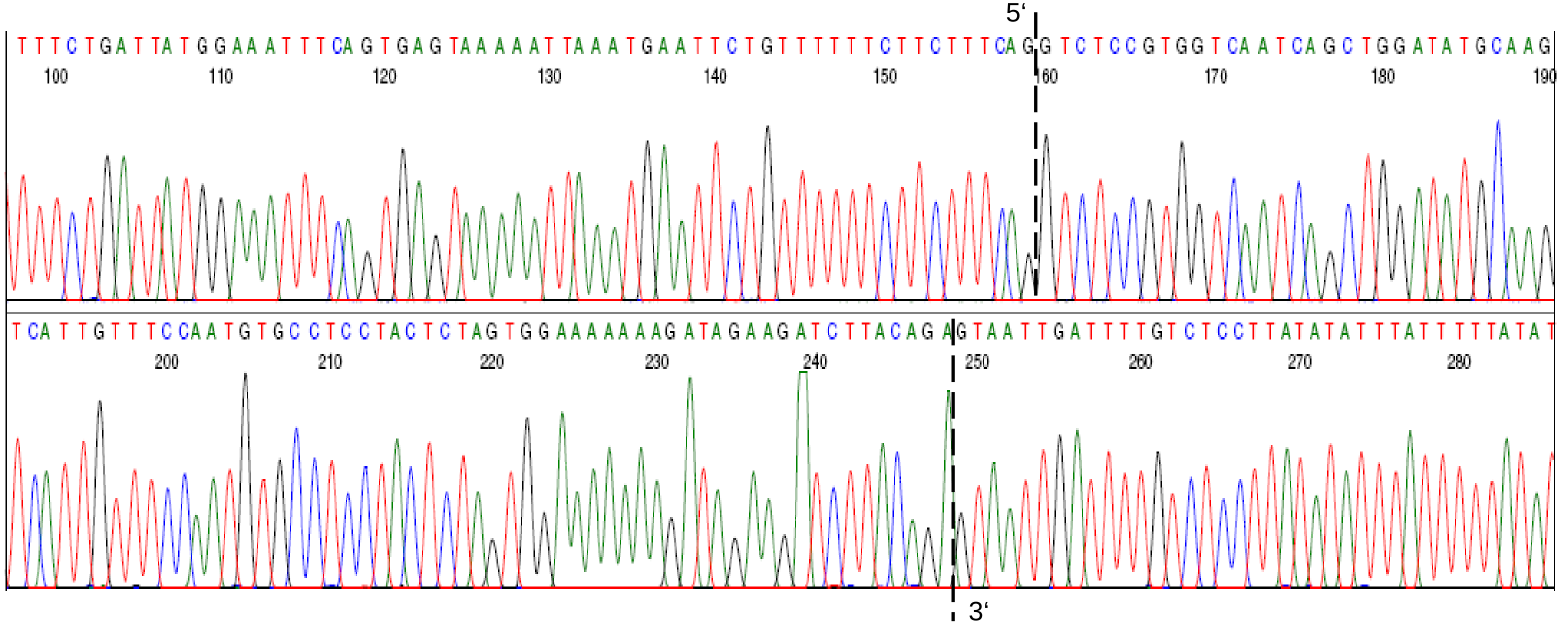

Exon 29

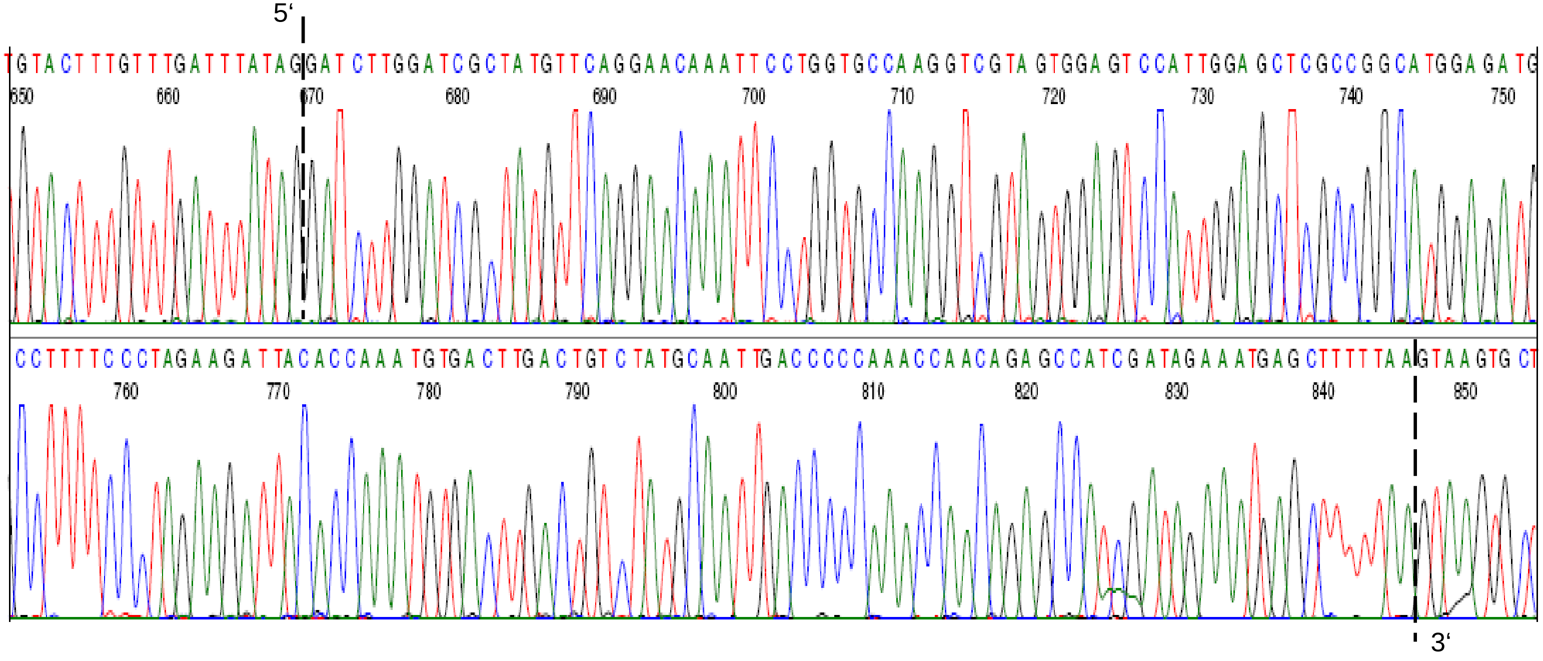

Exon 30

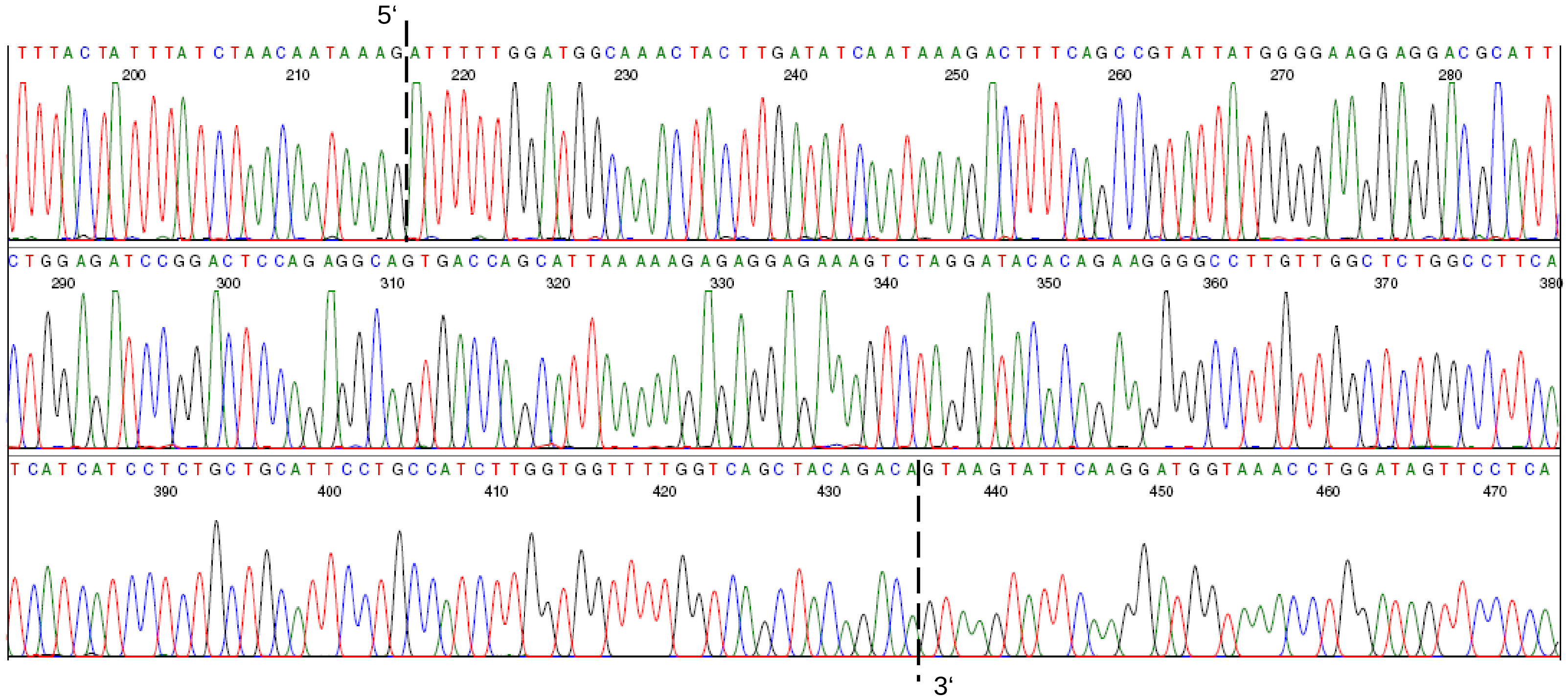

Exon 31

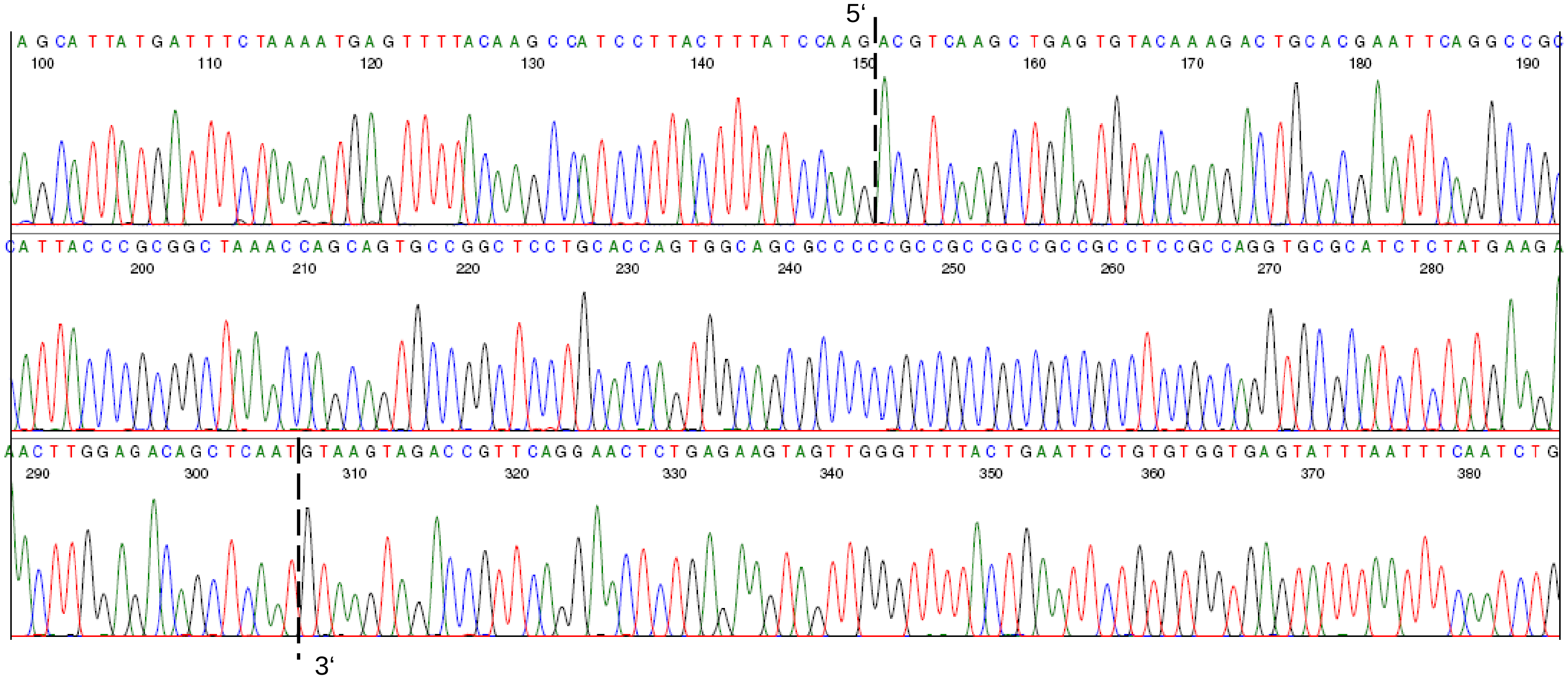

Exon 32

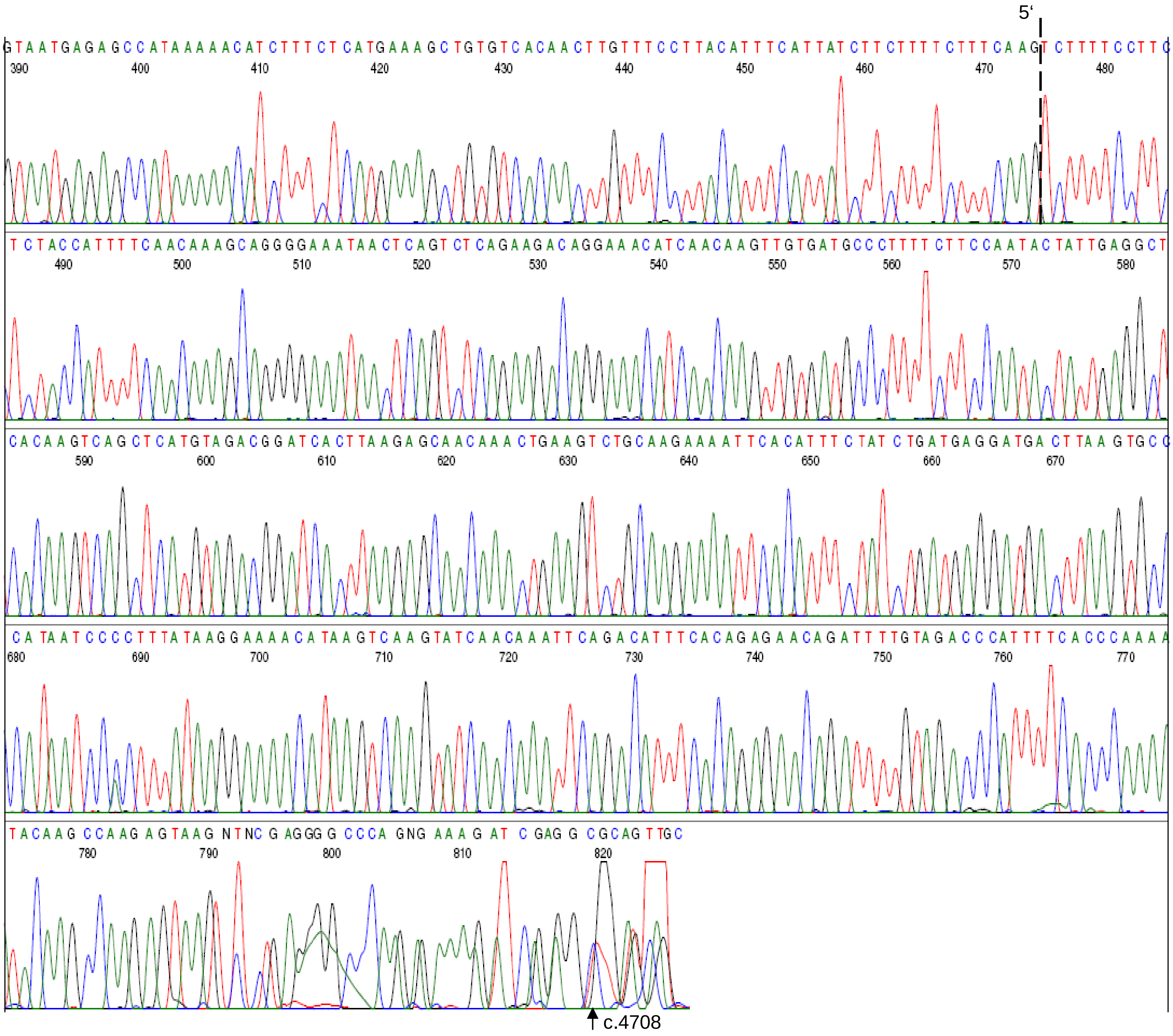
5‘
GTAATGAGAGCCATAAAAACATCTTTCTCATGAAAGCTGTGTCACAACTTGTTTCCTTACATTTCATTATCTTCTTTTCTTTCAAGTCTTTTCCTTC
TCTACCATTTTCAACAAAGCAGGGGAAATAACTCAGTCTCAGAAGACAGGAAACATCAACAAGTTGTGATGCCCTTTTCTTCCAATACTATTGAGGCT
CACAAGTCAGCTCATGTAGACGGATCACTTAAGAGCAACAAACTGAAGTCTGCAAGAAAATTCACATTTCTATCTGATGAGGATGACTTAAGTGCC
CATAATCCCCTTTATAAGGAAAACATAAGTCAAGTATCAACAAATTCAGACATTTCACAGAGAACAGATTTTGTAGACCCATTTTCACCCAAAA
TACAAG CCAAG AGTAAG NTNC G AGGG G CCCA GNG AAA G AT C GAG G CGCAG TTGC
c.4708

Exon 32

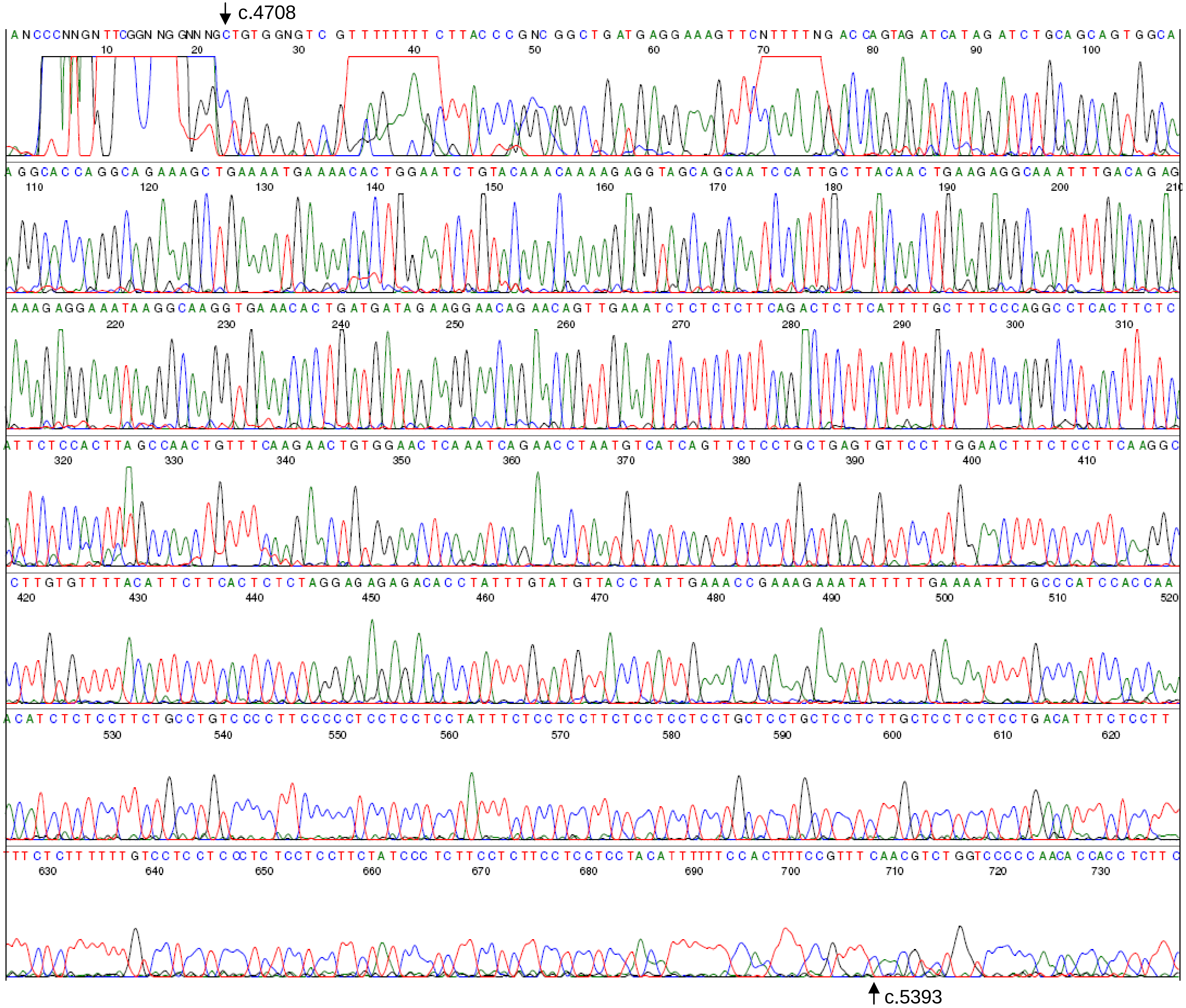

Exon 32

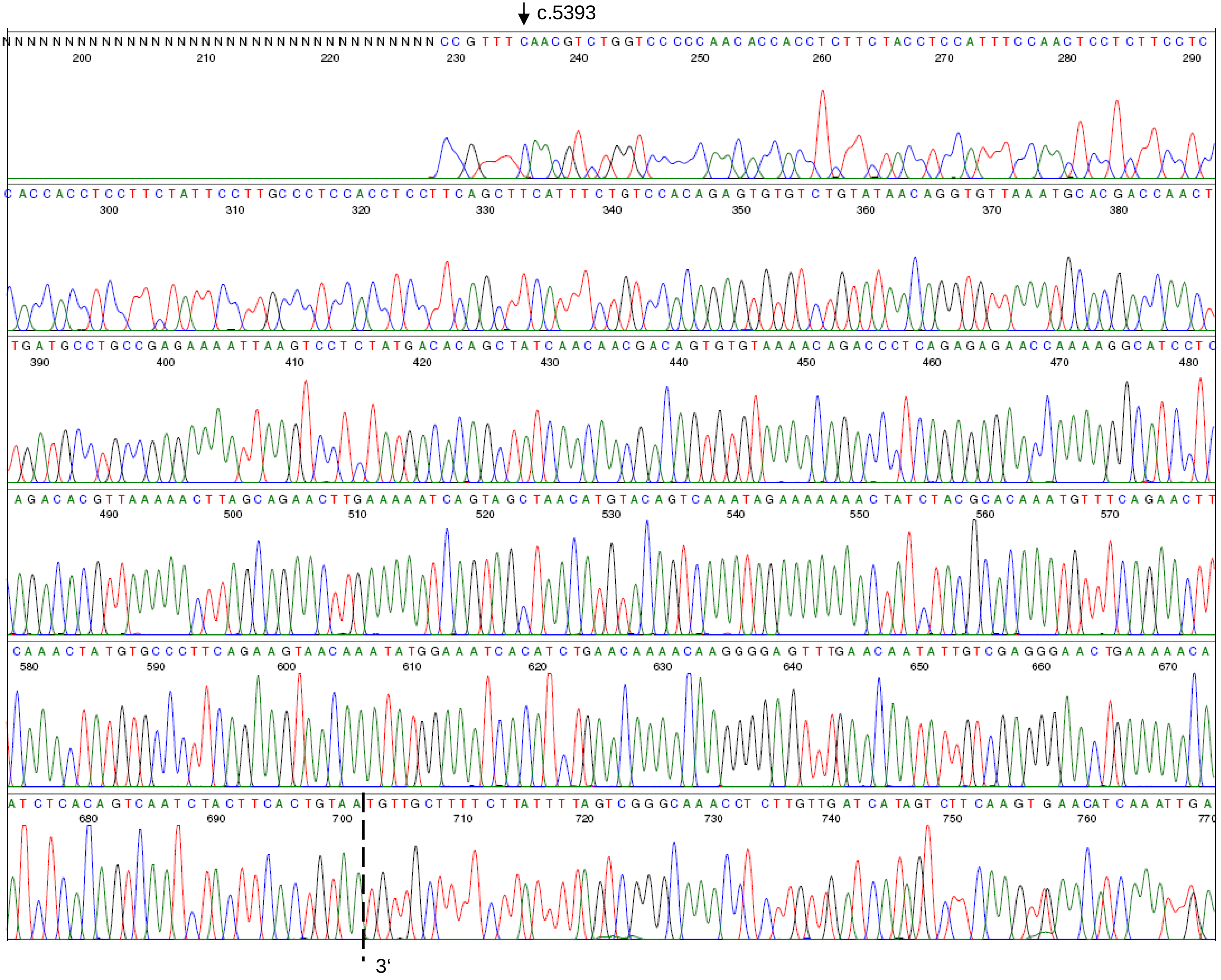

<u>Patient 1881, *SANS* (*USH1G*)</u>

Exon 1

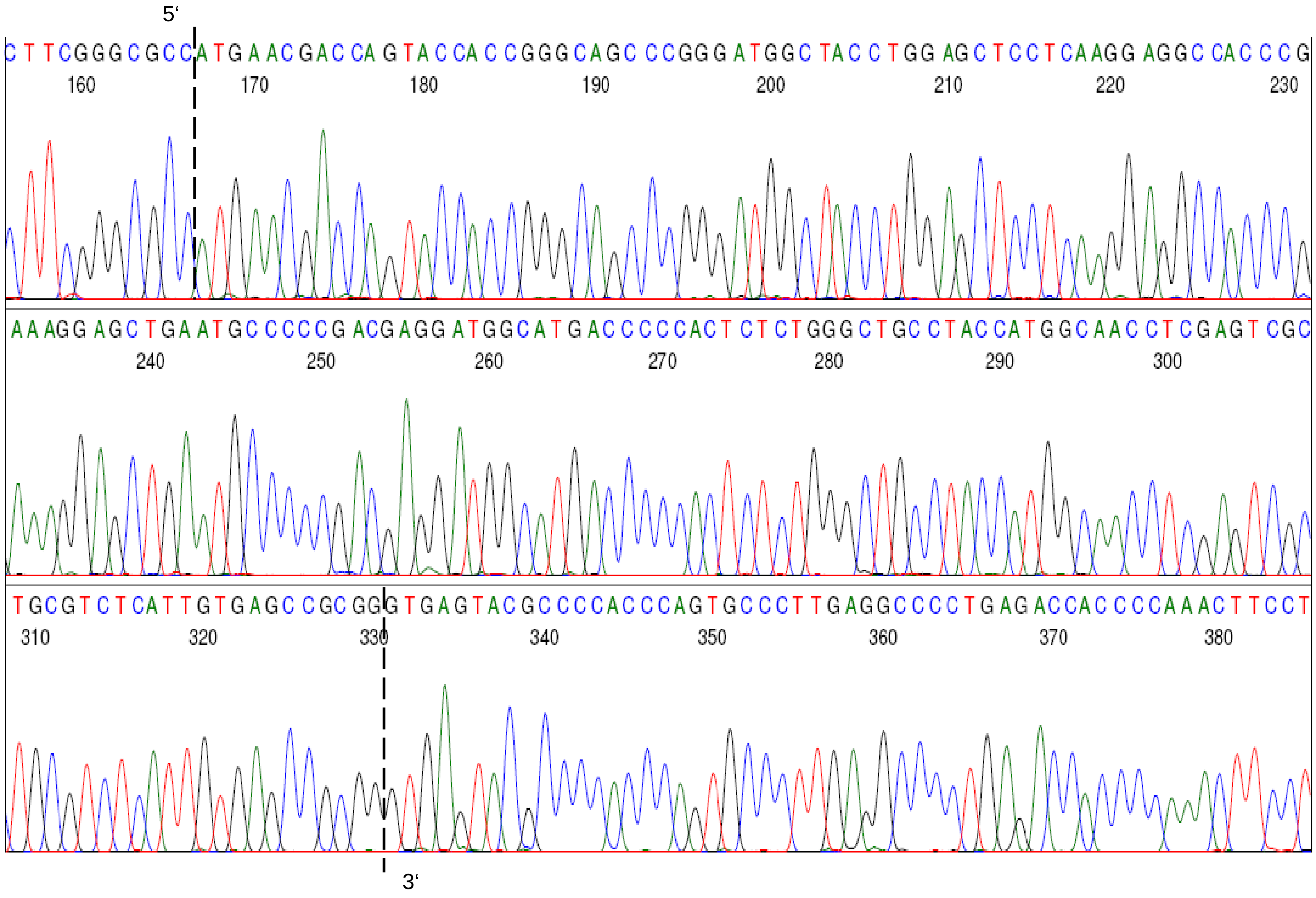

Exon 2

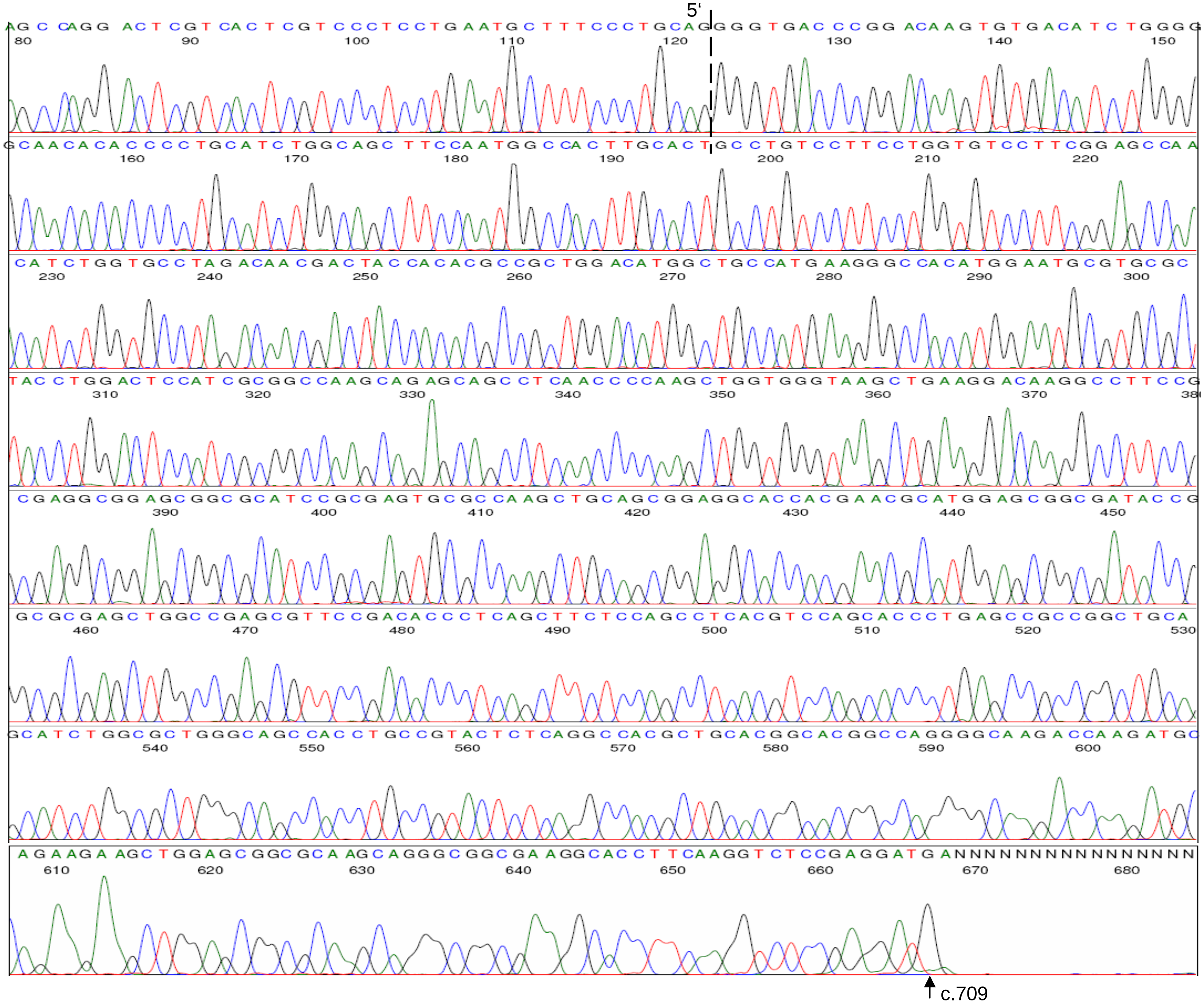

Exon 2

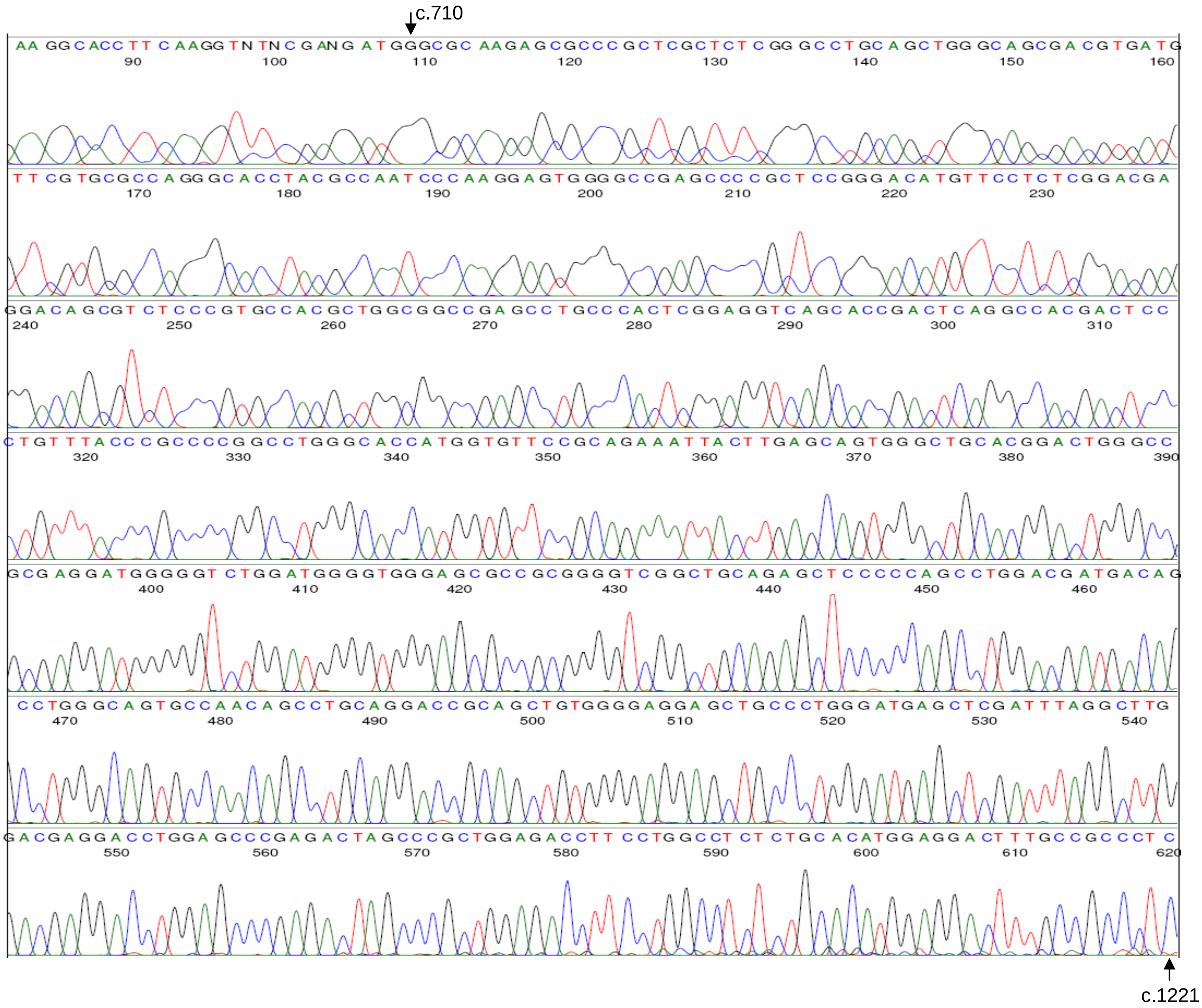

Exon 2

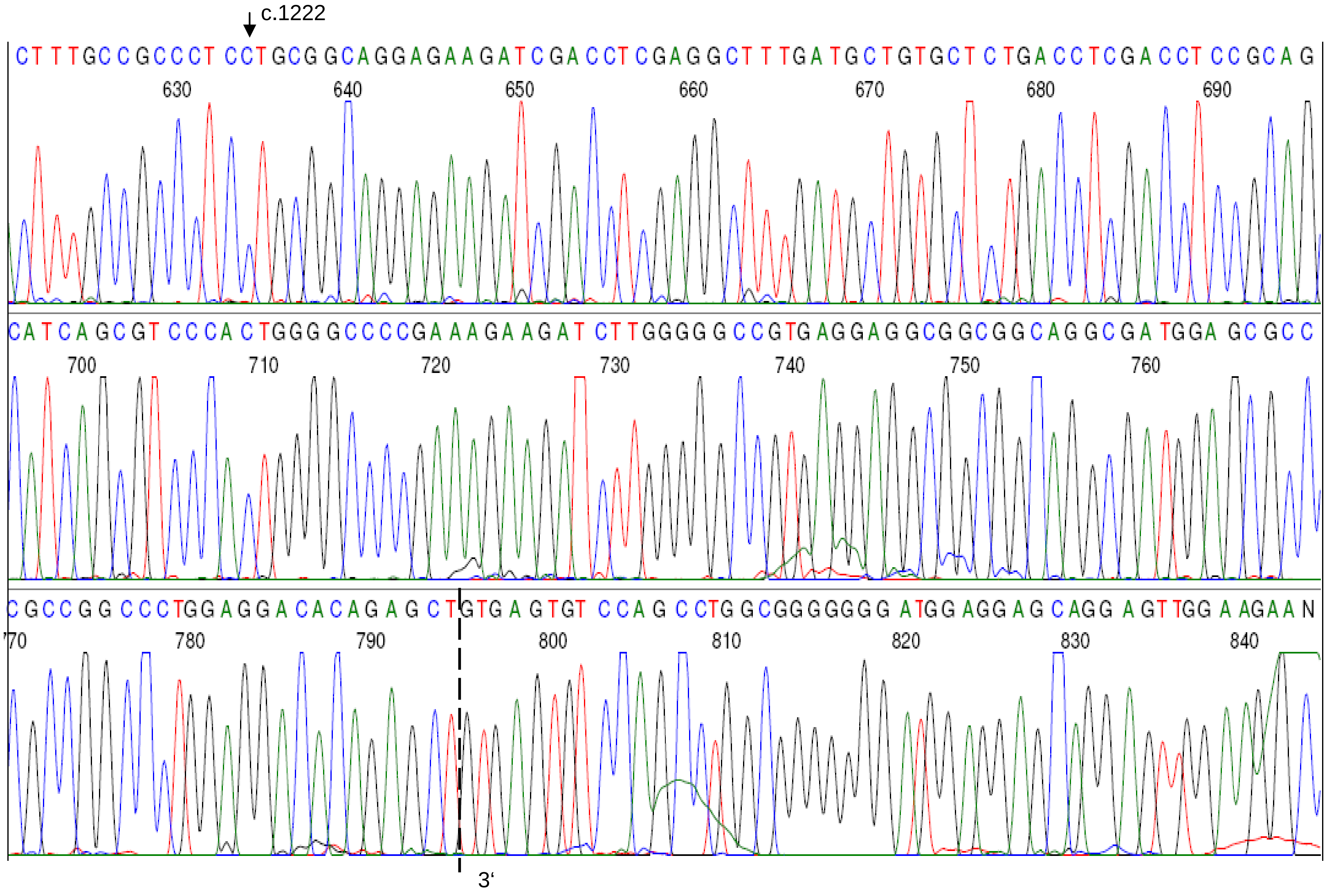

Exon 3

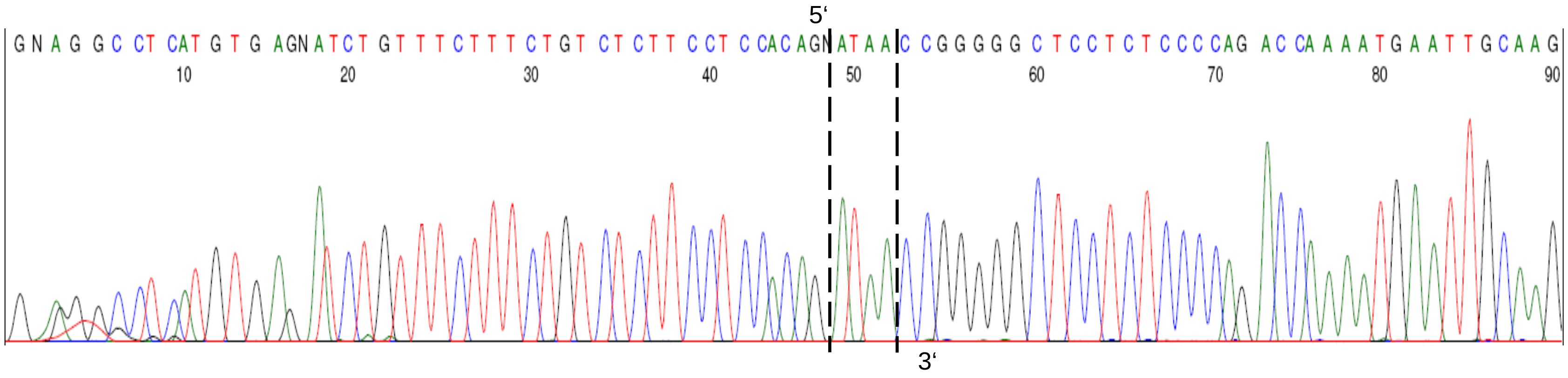
GNAGGCCTCATGTGAGNATCTGTTTCTTTCTGTCTCTTCCTCCACAGNATAACCGGGGGCTCCTCTCCCCAGACCAAAATGAATTGCAAG
10
20
30
40
50
60
70
80
90
5‘
3‘

<u>Patient 1881, *USH3A*</u>

Exon 1

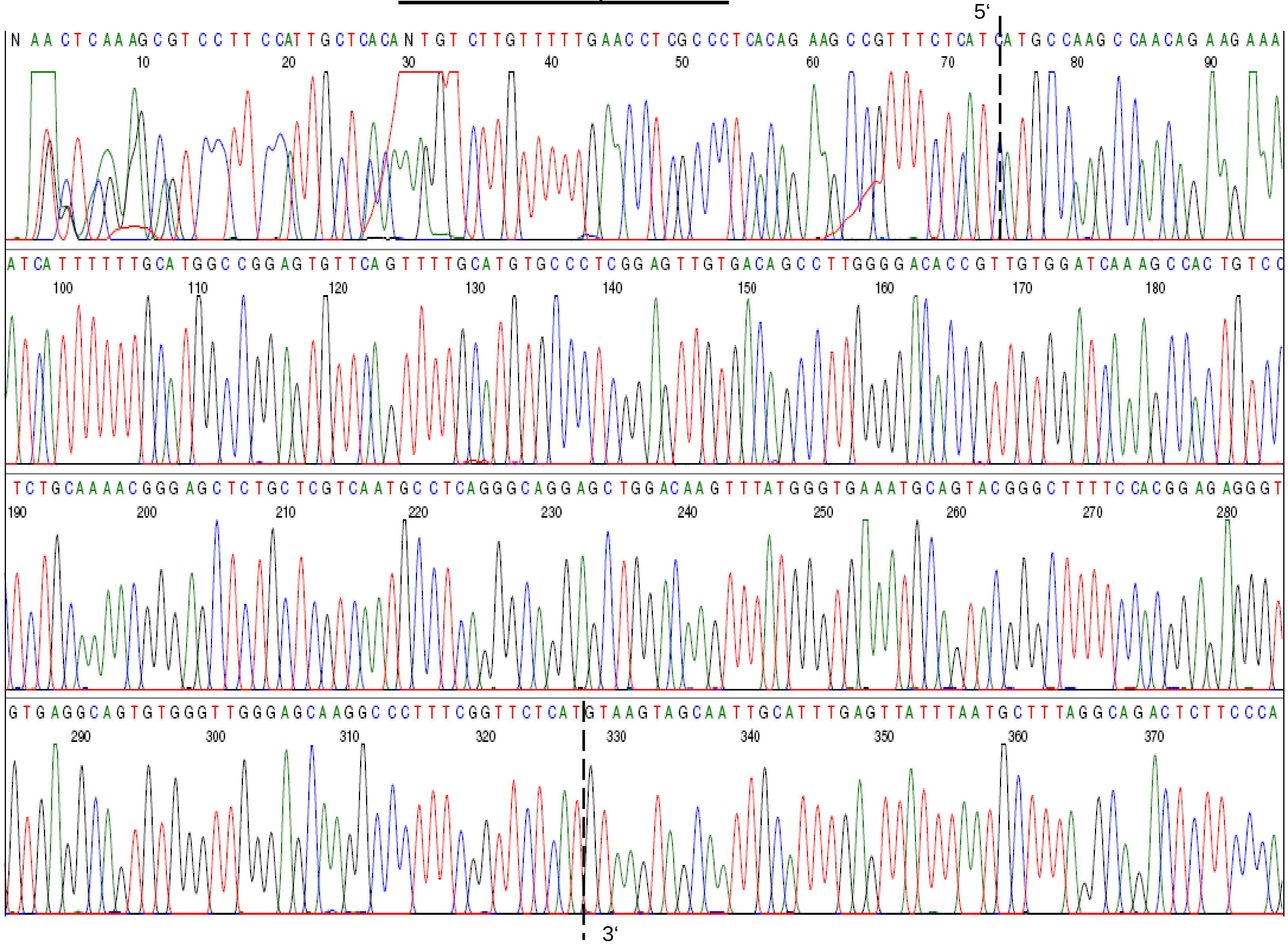

Exon 2

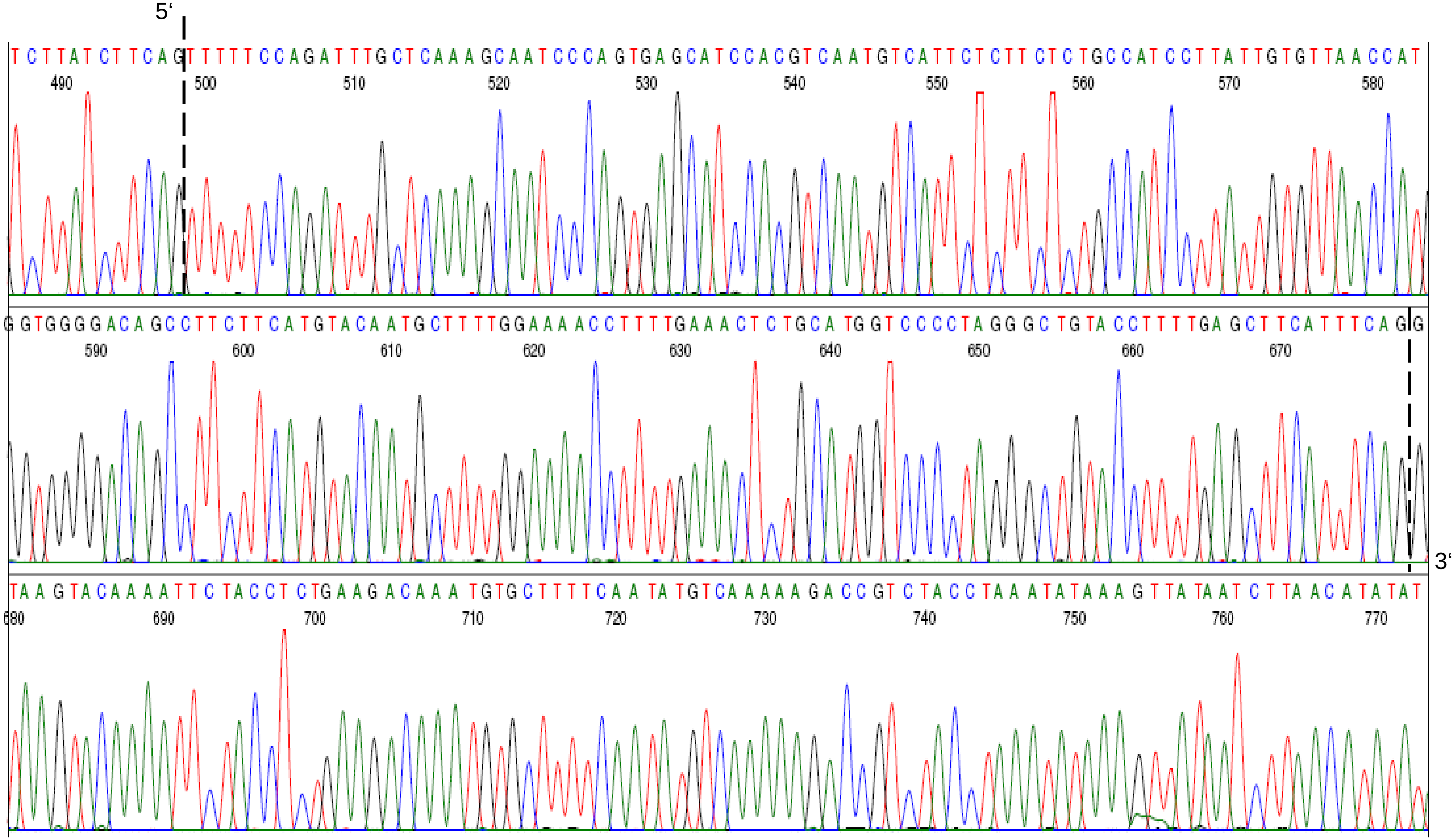

Exon 3

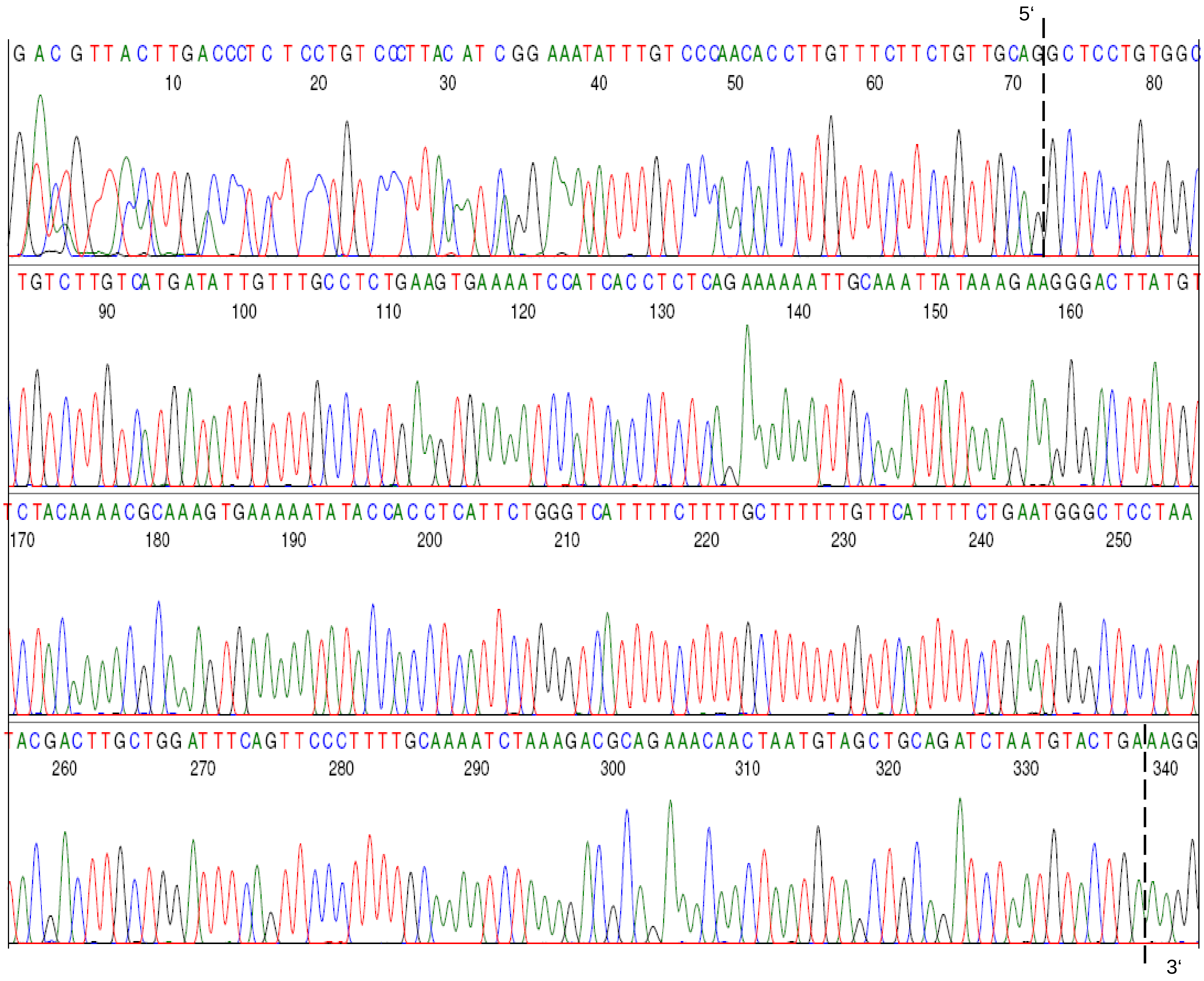

Supplement: Additional data file 9 — Results of mutation screening in patient 1881 in the genes PCDH15 (USH1F), SANS (USH1G) and USH3A (no mutations found). [file gb-2007-8-4-r47-S9.pdf]
